# Suprabasal cells retain progenitor cell identity programs in eosinophilic esophagitis–driven basal cell hyperplasia

**DOI:** 10.1172/jci.insight.171765

**Published:** 2023-10-09

**Authors:** Margarette H. Clevenger, Adam L. Karami, Dustin A. Carlson, Peter J. Kahrilas, Nirmala Gonsalves, John E. Pandolfino, Deborah R. Winter, Kelly A. Whelan, Marie-Pier Tétreault

**Affiliations:** 1 Department of Medicine, Gastroenterology and Hepatology Division, Northwestern University Feinberg School of Medicine, Chicago, Illinois, USA.; 2 Department of Cancer & Cellular Biology, Fels Cancer Institute for Personalized Medicine, Temple University Lewis Katz School of Medicine, Philadelphia, Pennsylvania, USA.; 3 Department of Medicine, Rheumatology Division, Northwestern University Feinberg School of Medicine, Chicago, Illinois, USA.

**Keywords:** Gastroenterology, Inflammation, Allergy, Bioinformatics, Molecular biology

## Abstract

Eosinophilic esophagitis (EoE) is an esophageal immune-mediated disease characterized by eosinophilic inflammation and epithelial remodeling, including basal cell hyperplasia (BCH). Although BCH is known to correlate with disease severity and with persistent symptoms in patients in histological remission, the molecular processes driving BCH remain poorly defined. Here, we demonstrate that BCH is predominantly characterized by an expansion of nonproliferative suprabasal cells that are still committed to early differentiation. Furthermore, we discovered that suprabasal and superficial esophageal epithelial cells retain progenitor identity programs in EoE, evidenced by increased quiescent cell identity scoring and the enrichment of signaling pathways regulating stem cell pluripotency. Enrichment and trajectory analyses identified SOX2 and KLF5 as potential drivers of the increased quiescent identity and epithelial remodeling observed in EoE. Notably, these alterations were not observed in gastroesophageal reflux disease. These findings provide additional insights into the differentiation process in EoE and highlight the distinct characteristics of suprabasal and superficial esophageal epithelial cells in the disease.

## Introduction

Eosinophilic esophagitis (EoE) is an esophageal disease characterized by eosinophilia that results in dysphagia, edema, esophageal stricture, and food impaction due to a type 2 immune response triggered by food allergens. Current management of EoE includes proton pump inhibitors, topical corticosteroids, diet elimination, and dupilumab (1, 2). Despite advancements in EoE treatment, a considerable number of patients face symptom relapse or inadequate response to existing therapies (3, 4), resulting in unfavorable prognosis, diminished quality of life, and substantial healthcare expenses attributed to frequent procedures and lifelong treatment requirements (5). Hence, current efforts to improve therapeutic options focus on the alleviation of symptoms and prevention of complications.

**Figure 1:**
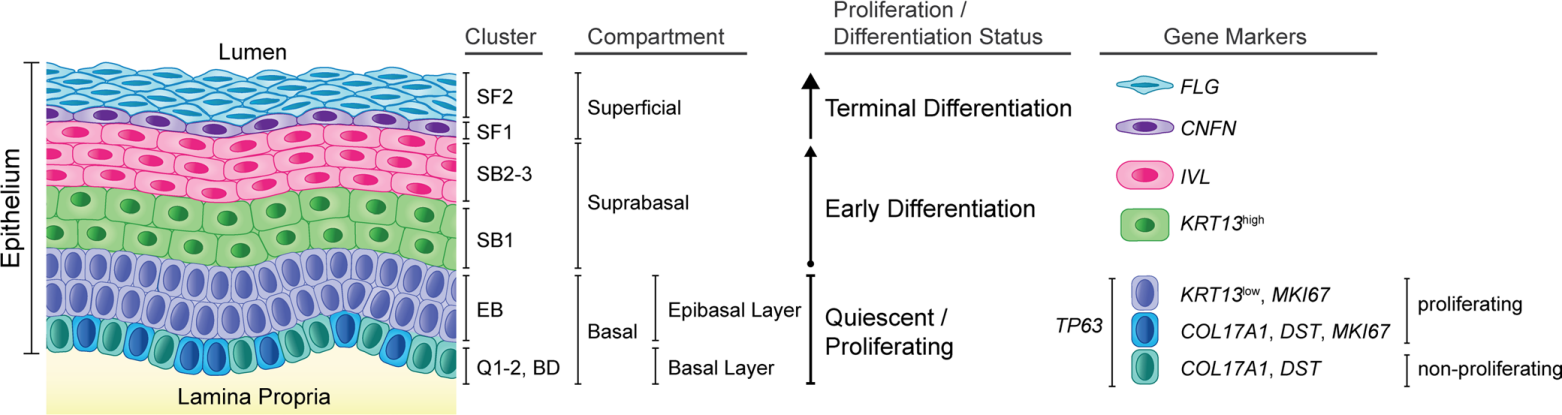
Histology of the human esophageal epithelium. Simplified schematic of the different epithelial compartments of the adult esophagus, summarizing the proliferation/differentiation state, the different epithelial clusters identified by scRNA-Seq analyses, and corresponding gene markers. Papillae are omitted for simplicity.

In the esophagus, primary protection against food antigens passing through the lumen is provided by the stratified squamous epithelial barrier. After arising from the stem cells in the basal compartment, esophageal epithelial cells (EEC) migrate through the suprabasal compartment and initiate an early differentiation process, before reaching the superficial compartment where they complete terminal differentiation and eventually desquamate (Figure 1). Upon damage to the epithelial barrier, a rapid restoration of epithelial homeostasis is achieved through balanced self-renewal and differentiation of stem/progenitor cells. Dysregulated inflammation, aberrant tissue repair mechanisms, or failure to restore homeostasis will ultimately have pathological consequences (6).

Adverse alterations to the esophageal epithelium are a primary driver of EoE (7) and include intraepithelial eosinophilic inflammation, basal cell hyperplasia (BCH), dilatation of intercellular space, and dysregulated terminal differentiation (8, 9). Histologically, BCH is the most prominent epithelial change in EoE and is defined by pathologists as an expansion of EEC within the basal zone (10). Despite the predominant incidence of BCH in EoE, the changes in the molecular and cellular identity occurring in BCH are largely unexplored. Underscoring the importance of understanding the role of BCH in EoE pathogenesis, BCH is linked to disease severity in EoE and directly correlates with persistent symptoms (odds ratio, 2.14; 95% CI, 1.03–4.42; *P* = 0.041) and endoscopic findings (odds ratio, 7.10; 95% CI, 3.12–16.18; *P* < 0.001) in patients in histologic remission (10). While a recent study demonstrated that a ~15% increase in cycling epibasal (*PDPN*^–^) cells contributed to BCH in EoE (8), BCH pervades approximately 65% of the epithelial surface area in patients with EoE (11). This indicates that BCH is associated with additional distinct alterations in EEC characteristics that extend beyond hyperproliferation. Thus, a better molecular characterization of BCH is needed to improve the current understanding of symptom recurrence and persistent endoscopic findings in EoE. This will ultimately guide the development of novel therapeutic approaches for EoE, particularly for cases in which reducing eosinophilic inflammation is not sufficient to restore epithelial tissue integrity or to improve clinical symptoms.

To address this gap in knowledge and investigate more extensively the molecular changes occurring in BCH, we performed single-cell RNA-Seq (scRNA-Seq) of esophageal mucosal biopsies from treatment-naive adult patients with EoE and healthy controls (HC). Our findings reveal that BCH in EoE primarily involves the expansion of nonproliferative suprabasal EEC that are committed to early differentiation while retaining a progenitor cell identity. Through our analysis, we identified the transcription factors (TFs) and regulators of stem cell renewal, SOX2 and KLF5, as the prominent predicted regulators of differentially expressed genes (DEGs) in these atypical early differentiated EEC found in EoE. We further confirmed the increased expression of SOX2 and KLF5, along with their downstream targets, in the early differentiated EEC observed in EoE. Finally, these alterations were not detected in individuals with gastroesophageal reflux disease (GERD).

## Results

### Characterization of esophageal mucosal cell populations in adult EoE.

**Figure 2:**
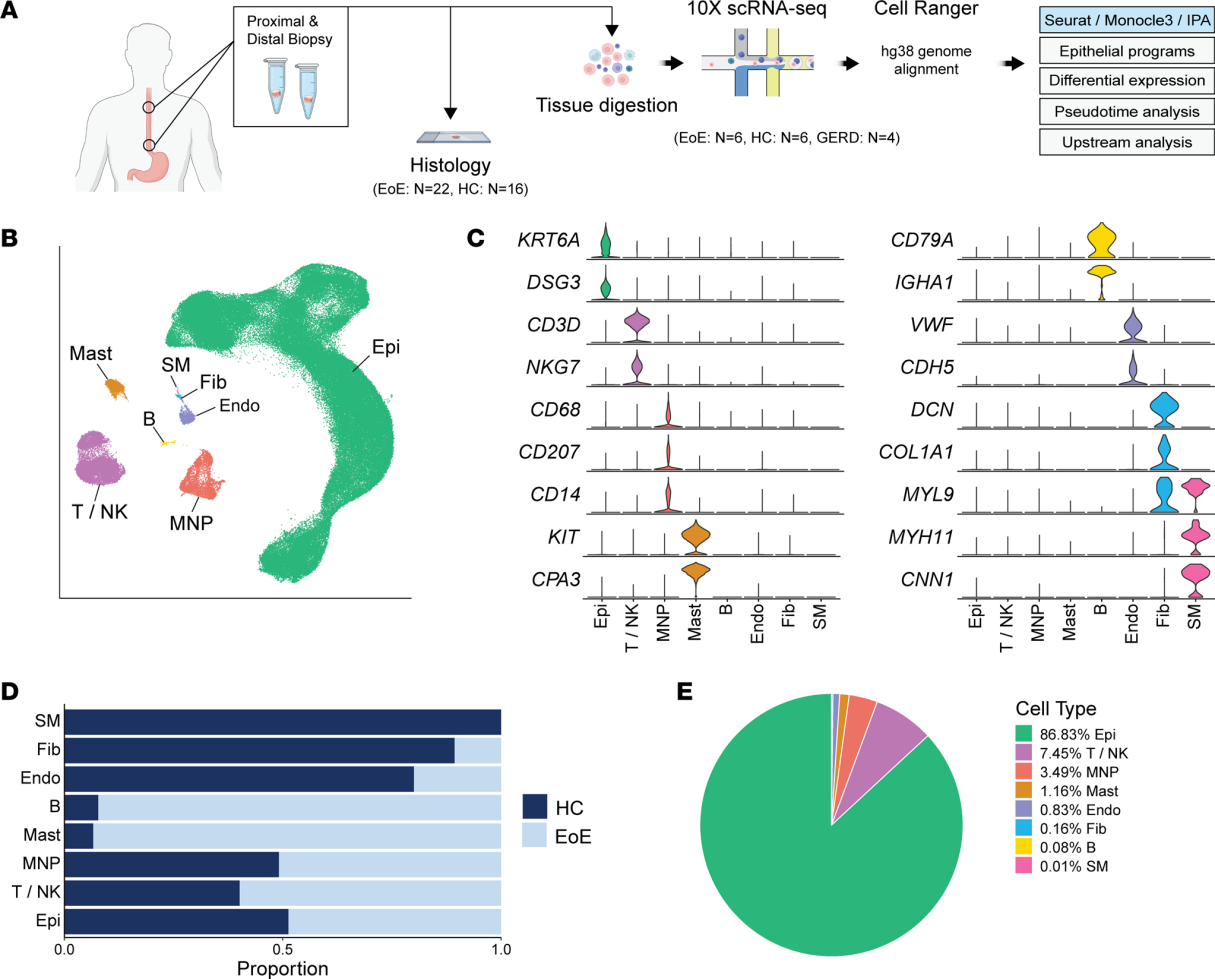
Single-cell transcriptomic landscape of esophageal mucosal cells in EoE and healthy subjects. (**A**) Schematic of study design. (**B**) UMAP embedding of the unsupervised clustering of cells from HC and EoE. (**C**) Violin plots illustrating the expression of established cell type–specific markers. (**D**) Bar plot displaying the frequency of each esophageal cell type in HC and EoE. (**E**) Pie chart demonstrating the proportion of each esophageal cell type. Epi, Epithelial cells; T/NK, T cells/NK cells; MNP, mononuclear phagocytes; Mast, mast cells; B, B cells; Endo, endothelial cells; Fib, fibroblasts; SM, smooth muscle cells.

To characterize the single-cell transcriptomic landscape of the esophageal mucosa in EoE, we obtained proximal and distal biopsies from 6 adults with EoE along with 6 HC (Figure 2A). Histological processing was performed on additional adjacent biopsies (Figure 2A). Immunostaining was conducted on biopsies from 22 additional EoE subjects and 16 HC to validate scRNA-Seq findings. Patient characteristics and demographics are summarized in Table 1. Fresh tissue specimens were digested to generate single-cell suspensions and sequenced using the 10X Genomics platform. After quality control filtering, integration was performed using reciprocal principal component analysis (PCA) dimensional reduction. Uniform Manifold Approximation and Projection (UMAP) for Dimension Reduction embeddings were calculated using the Seurat R package (12), followed by unsupervised graph-based clustering. Clusters were annotated based on established marker genes (Figure 2, B and C) and transcriptional signatures (Supplemental Figure 1A; supplemental material available online with this article; https://doi.org/10.1172/jci.insight.171765DS1).

Within the integrated data set of 151,519 cells, we identified 8 major cell populations: epithelial cells (Epi) (*n* = 131,822), T cells and NK cells (T/NK) (*n* = 11,134), mononuclear phagocytes (MNP) (*n* = 5,211), mast cells (Mast) (*n* = 1,733), B cells (B) (*n* = 116), endothelial cells (Endo) (*n* = 1,239), fibroblasts (Fib) (*n* = 244), and smooth muscle cells (SM) (*n* = 20) (Figure 2B). Representative marker genes used for cell type annotation included *KRT6A* and *DSG3* (Epi); *CD3D* and *NKG7* (T/NK); *CD68*, *CD207*, and *CD14* (MNP); *KIT* and *CPA3* (Mast); *CD79A* and *IGHA1* (B); *VWF* and *CDH5* (Endo); *DCN*, *COL1A1*, and *MYL9* (Fib); and *MYL9*, *MYH11*, and *CNN1* (SM) (Figure 2C). We obtained 85,745 cells from HC and 65,774 from EoE (Supplemental Figure 1B). The distribution of major cell populations was largely similar between the EoE and HC groups (Figure 2D and Supplemental Figure 1C), with EEC being the predominant cell type (Figure 2E).

### Defining EEC clusters in HC and EoE.

**Figure 3:**
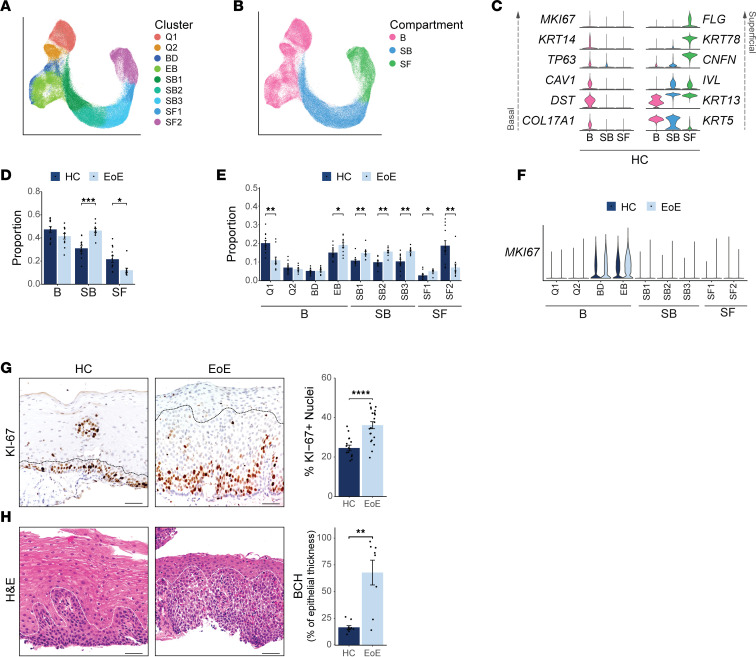
Identification and characterization of human EEC populations and their alterations in EoE. (**A** and **B**) UMAP of the subclustering of epithelial populations, colored by cluster (**A**) or compartment (**B**). (**C**) Violin plots displaying the expression of established marker genes for each EEC compartment in HC. (**D** and **E**) Bar plot showing the proportion of EEC in each compartment (**D**) or cluster (**E**), represented as a fraction of all EEC for each disease condition. (**F**) Violin plot displaying *MKI67* expression across epithelial clusters in HC and EoE. (**G**) Representative immunohistochemistry for KI-67 in the esophageal epithelium and quantification of KI-67^+^ cells from IHC in HC (*n* = 16) and EoE (*n* = 21). The dashed black line outlines the basal compartment (HC) or BCH (EoE), excluding papillae. Scale bar: 100 μm. (**H**) H&E staining of HC and EoE esophageal mucosal sections from patients (HC, *n* = 6; EoE, *n* = 6) in the scRNA-Seq cohort and box plot showing the height of the epithelium occupied by the basal zone, quantified as a function of total epithelial thickness. Scale bar: 100 μm. The dashed white line outlines the basal compartment (HC) or BCH (EoE). For bar plots, data are expressed as mean ± SEM and *P* values were determined using the Wilcoxon signed-ranked test. For **D** and **E**, Benjamini & Hochberg adjustment for multiple comparisons was employed. *P ≤ 0.05, **P ≤ 0.01, ***P ≤ 0.001, ****P ≤ 0.0001. B, Basal; Q, Quiescent; BD, Basal_Dividing; EB, Epibasal; SB, Suprabasal; SF, Superficial.

The prominent representation of EEC in our data set (86.83%) (Figure 2E), a central contributor to EoE pathogenesis (13, 14), enabled high-resolution characterization of their transcriptional changes in EoE. To ensure that UMAP embeddings were assigned based on epithelial subtypes under homeostatic conditions, EEC were reintegrated using anchors identified from HC samples, enabling the representation of HC and EoE EEC within each cluster (Supplemental Figure 2, A and B). Ten epithelial clusters were identified via unsupervised graph-based clustering (Supplemental Figure 2, A–C). To distinguish slow-cycling stem cells in the basal layer from faster-cycling epibasal cells, we performed subclustering of the quiescent (clusters 1 and 2) and dividing clusters (cluster 3, S-phase; clusters 4 and 5, G2/M phase) (Supplemental Figure 3A), as previously described (8). Subclustered cell populations were annotated based on the expression of *KRT13* (15), *DST* (16), and cell cycle markers (Supplemental Figure 3, A and B). Our assignment of cell populations aligns with previous classifications using high/low *PDPN* expression (8) (Supplemental Figure 3C). The basal compartment clusters, Quiescent_1 (Q1), Quiescent_2 (Q2), Basal_Dividing (BD), and Epibasal (EB), were then reassigned within the total esophageal epithelial object (Figure 3A).

To annotate the resulting 9 epithelial clusters and classify them into esophageal epithelial compartments (basal [B], suprabasal [SB], or superficial [SF]), we examined the expression of established marker genes in the HC data set (Figure 3, A–C, and Supplemental Figure 3D). Within the basal compartment, the quiescent (Q) EEC clusters Q1 and Q2 demonstrated elevated expression of quiescence markers *KRT15* and *DST*(7), while the proliferating clusters BD and EB displayed increased expression of the S-phase marker *PCNA* (17, 18) and the G2/mitosis marker *MKI67* (19) (Supplemental Figure 3D). Consistent with the existing literature, basal cells exhibited expression of the TFs *SOX2* (20) and *TP63* (21–23) (Figure 1, Figure 3C, and Supplemental Figure 3D). Suprabasal clusters were identified based on the expression of *KRT13* (*KRT13*^hi^) (24), *IVL* (25), and *SERPINB3* (8) (Figure 1, Figure 3C, and Supplemental Figure 3D). The superficial markers *CNFN* (8, 26), *FLG* (27), and *KRT78* (28) were used to characterize superficial cell clusters (Figure 1, Figure 3C, and Supplemental Figure 3D). Cluster annotation was confirmed using the transcriptional profiles of each HC cell cluster (Supplemental Figure 3E).

### BCH is characterized by the expansion of nonproliferative suprabasal cells committed to early differentiation.

We next examined alterations in the relative representation of EEC compartments between EoE and HC. Surprisingly, we did not observe an expansion of the basal compartment in EoE (Figure 3D). However, analysis of EEC proportions at the cluster level revealed a decrease of the quiescent reserve EEC (Q1) and an increase of the fast-cycling epibasal cells in EoE (Figure 3E). The quantification of Ki-67 staining confirmed the increased proliferation in epibasal cells above the basal layer observed by scRNA-Seq (Figure 3, F and G). This shift in cell proportion confirms the previously reported hyperproliferation in EoE (29, 30). BCH scoring in adjacent esophageal mucosal biopsies from scRNA-Seq patients using EoE-HSS criteria (Figure 3H and Supplemental Figure 4, A–C) revealed an increase greater than 3-fold in the percentage of epithelium thickness occupied by BCH in EoE compared with HC (Figure 3H). These findings suggest that the morphological changes associated with BCH extend higher up in the esophageal epithelium, beyond the detected hyperproliferation, indicating that additional changes in EEC may occur during the development of BCH. To gain further insight into the cell identity of EEC labeled as basal in the histological evaluation of BCH, we examined the changes in cell proportions within different EEC clusters in EoE. Interestingly, we observed an expansion of EEC belonging to the suprabasal clusters, and all these clusters exhibited a nonproliferative phenotype (Figure 3, D–F, and Supplemental Figure 4, D and E). This finding suggests that the EEC identified as basal in the histological assessment of BCH may actually represent nonproliferative differentiated suprabasal cells with an abnormal morphology.

### Increased basal identity marker expression is observed in suprabasal and superficial EEC in EoE.

**Figure 4:**
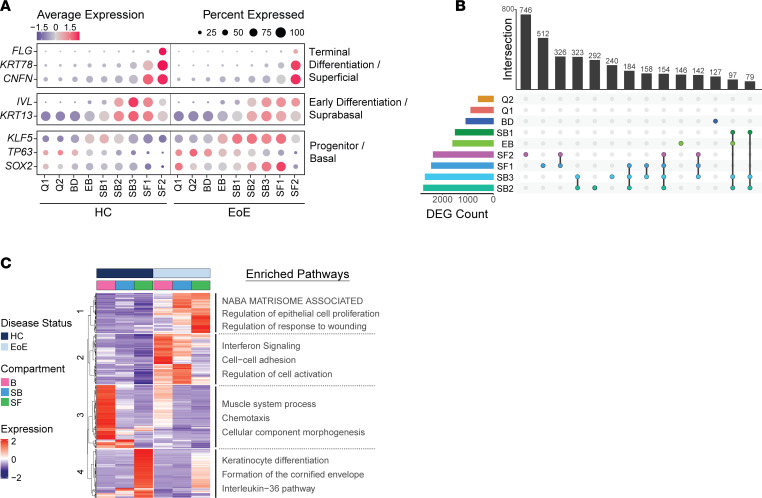
Characterization of the transcriptional changes in the suprabasal and superficial EEC compartments in EoE. (**A**) Cluster average expression *Z* scores of EEC markers in HC and EoE. Color gradient indicates the average gene expression per cluster; dot size indicates the percentage of cells exhibiting gene expression per cluster. (**B**) UpSet plot displaying the number of DEGs per EEC cluster, calculated between EoE and HC. Total DEG counts per cluster and unique DEG intersections are shown as bar plots. Intersections are ordered from greatest to least, with the top 14 visualized. (**C**) Heatmap of log_2_ normalized *Z* score expression of DEGs across all EEC, calculated between EoE and HC (|logFC| > 1, FDR-adjusted *P* < 0.05). Top hierarchical clusters are displayed with enriched pathways. B, Basal; Q, Quiescent; BD, Basal_Dividing; EB, Epibasal; SB, Suprabasal; SF, Superficial.

To gain deeper insights into the alterations in cell identity associated with BCH, we investigated changes in transcriptional profiles within epithelial clusters in EoE. Our analysis initially focused on differentiation markers associated with basal, suprabasal, and superficial cell identities. We observed a substantial decrease in *FLG* expression within the superficial cluster SF2 as well as reduced expression of *CNFN* and *KRT78* in the superficial cluster SF1 in EoE compared with HC (Figure 4A). This loss of terminal differentiation is a well-documented characteristic in EoE (8, 13). Interestingly, we observed that the increased expression of *KRT13* and *IVL*, which occurs during suprabasal commitment, was still present in the suprabasal clusters in EoE as compared with the basal compartment, albeit at slightly lower levels compared with HC (Figure 4A and Supplemental Figure 4D). Surprisingly, in addition to their expected expression in basal clusters, genes associated with basal cells, such as *SOX2*, *KLF5*, and *TP63*, were also expressed throughout the suprabasal and superficial clusters SB1 through SF1 in EoE (Figure 4A). Our analysis reveals that EoE is not solely characterized by a loss of terminal differentiation but rather demonstrates that a majority of EEC still initiate an early differentiation process. Most notably, our analyses unveiled that both early and terminally differentiated EEC retain the expression of genes associated with basal cells in EoE.

### Differential gene expression analysis reveals a dysfunctional differentiation process in EoE.

**Figure 5:**
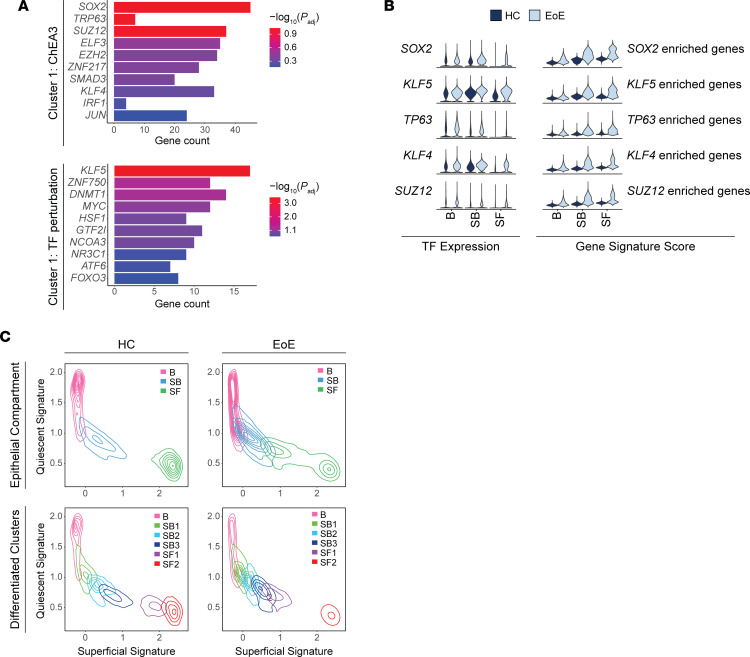
Progenitor-associated transcription factors shift the quiescent/basal/differentiation transition in EoE. (**A**) TF analysis for genes upregulated in hierarchical cluster 1 using the ChEA3 2022 database or the TF perturbations followed by expression database. Color intensity indicates –log_10_ of adjusted *P*. (**B**) Violin plots displaying the expression of TFs identified in **A** and the gene signature scores of the DEGs associated with each TF in EEC compartments in HC and EoE. (**C**) Contour plots showing EEC from HC or EoE plotted along the quiescent gene signature (*y* axis) and the superficial gene signature (*x* axis). Line color indicates cell grouping by EEC compartment, with or without labeling of the suprabasal and superficial clusters. B, Basal; SB, Suprabasal; SF, Superficial.

To gain further insights into the molecular changes associated with EoE, we conducted differential gene expression analysis on EEC in EoE compared with HC per cluster. Our findings further support the pivotal role of suprabasal and superficial EEC in EoE pathogenesis, as we observed the highest number of DEGs in the SB2 and SB3 clusters, followed by SF1 through SF2 (Figure 4B). SB and SF clusters also exhibited the greatest number of overlapping gene changes (Figure 4B) (31). Additionally, greater DEG log_2_ fold-changes (logFC) were detected in SB2 through SF2 compared with other clusters (Supplemental Figure 5A). Pathway enrichment analysis was performed on the hierarchical clustering of the DEGs resulting from the comparison of all EEC in EoE to HC. Given our observation of expanded EEC in SB1 through SF1 in EoE (Figure 3E) and the extensive transcriptional changes observed in the suprabasal and superficial clusters, we hypothesized that the upregulated genes in cluster 1 within the suprabasal and superficial compartments of EoE (Figure 4C) are crucial drivers of BCH. To identify potential upstream regulators of the DEGs within cluster 1, we utilized EnrichR, which maintains updated databases of ChIP-Seq experiments and the Gene Expression Omnibus (GEO) signature of DEGs resulting from TF perturbations (32). Through enrichment analysis against these databases, we identified *SOX2*, *TP63*, and *KLF5*, 3 regulators of stem cell self-renewal in various tissues (21, 22, 33), as the top predicted TFs regulating the DEGs within cluster 1 (Figure 5A). Furthermore, the increased expression of *SOX2*, *TP63*, and *KLF5*, along with their downstream targets, was confirmed in the suprabasal and superficial compartments in EoE (Figure 5B).

### Suprabasal and superficial EEC retain a progenitor-like identity in EoE.

To further explore our hypothesis that suprabasal and superficial EEC maintain an epithelial progenitor-like identity in EoE, we developed 2 gene signatures that capture genes preferentially expressed in either quiescent cells or superficial cells in HC. The signatures were developed to establish a quiescent/basal/differentiation axis in human EEC (Supplemental Tables 1 and 2). Violin plots and contour plots mapping the quiescent signature score (*y* axis) and superficial signature score (*x* axis) by disease condition revealed a distinct separation between the superficial compartment of HC from the basal and suprabasal compartments (Figure 5C and Supplemental Figure 5B). However, in EoE, we observed a notable shift toward decreased superficial score and increased quiescent score in the superficial compartment, which resulted in an overlap between the superficial and suprabasal compartments (Figure 5C and Supplemental Figure 5B). Upon separation of the suprabasal and superficial compartments into epithelial clusters, we observed increased quiescent identity in each suprabasal and superficial cluster in EoE beginning at SB2, with the most dramatic shift in SF1 (Figure 5C and Supplemental Figure 5B). Further supporting maintained progenitor identity in early differentiated EEC in EoE, pathway enrichment analysis of the DEGs in each cluster between EoE and HC predicted the activation of the embryonic stem cell pluripotency pathway in EEC in EoE, with the highest activation scores and pathway coverage in suprabasal and superficial clusters (Supplemental Figure 5C).

**Figure 6:**
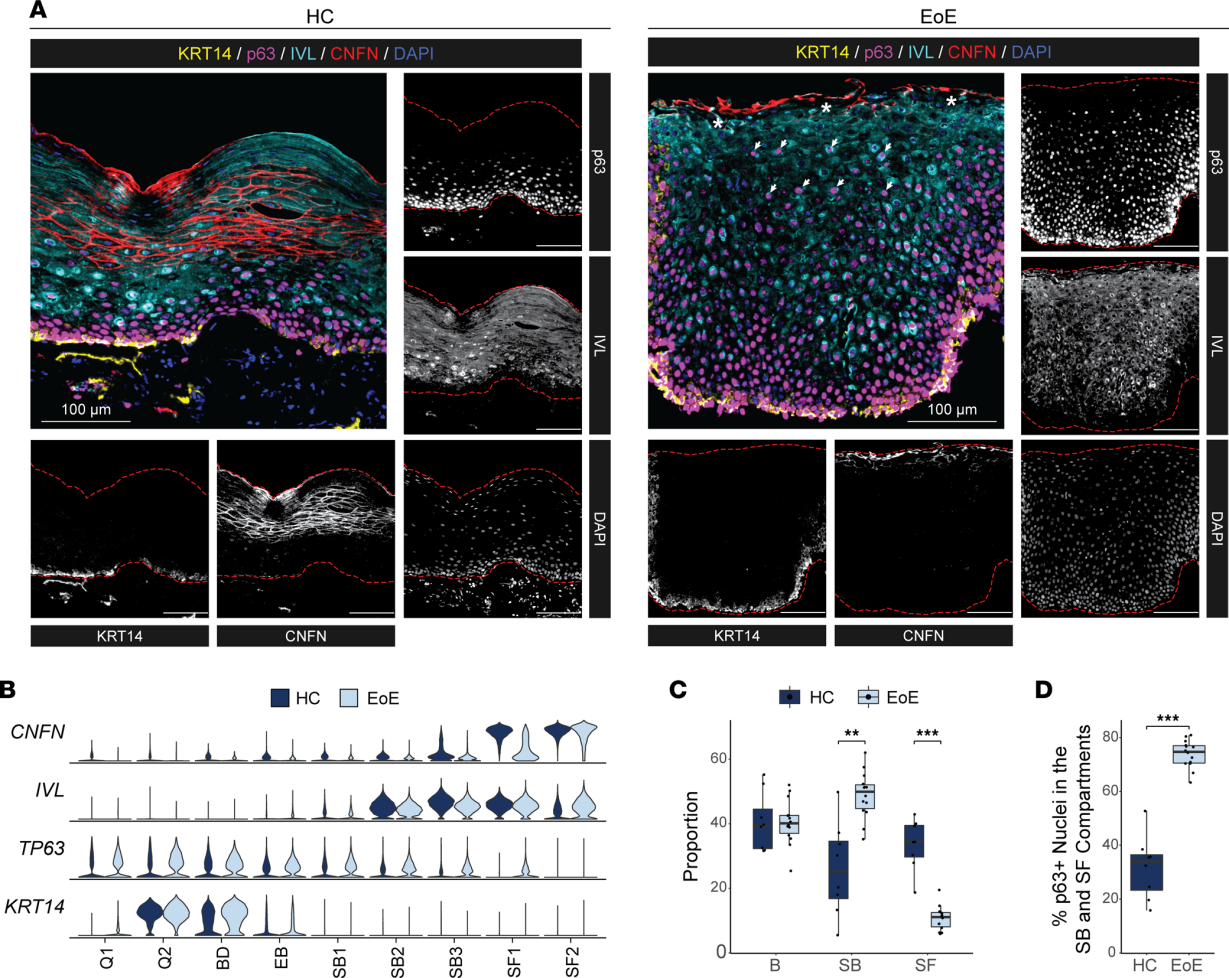
Validation of EoE-associated EEC identity changes identified by scRNA-Seq in esophageal tissue from HC and EoE. (**A**) Representative multispectral fluorescence tissue staining for markers of the epithelial basal (KRT14, yellow; p63, magenta), suprabasal (IVL, cyan), or superficial compartment (CNFN, red) in esophageal mucosal biopsies of HC (*n* = 8) or EoE (*n* = 14). Scale bar: 100 μm. Arrows indicate p63^+^IVL^+^ nuclei in the BCH expanded area; asterisks indicate regions of CNFN loss; red dashed lines indicate outlines of epithelial area. (**B**) Expression of indicated markers of EEC compartments across epithelial clusters in HC and EoE. (**C**) Box plot showing the proportion of cells in the basal (IVL^–^CNFN^–^), suprabasal (IVL^+^CNFN^–^), and superficial (CNFN^+^) compartments identified in multifluorescent staining in **A**, represented as the percentage of all EEC between disease conditions. (**D**) Box plot showing the proportion of p63^+^ nuclei in the suprabasal and superficial compartments identified in multifluorescent staining in **A**. For **C** and **D**, boxes represent quartiles, whiskers indicate minima/maxima, and lines through each box denote the median. All indicated *P* values were determined using Wilcoxon signed-ranked test, with Benjamini & Hochberg adjustment for multiple comparisons applied in **C**. **P ≤ 0.01, ***P ≤ 0.001. B, Basal; Q, Quiescent; BD, Basal_Dividing; EB, Epibasal; SB, Suprabasal; SF, Superficial.

To validate the alterations in the quiescent/basal/differentiation axis, we performed multispectral fluorescence staining on esophageal mucosal sections from HC and EoE using established markers of basal (KRT14, p63), suprabasal (IVL), and superficial (CNFN) cell identity (Figure 6A). For comparison, marker gene expression across clusters in HC or EoE is shown (Figure 6B). We confirmed appropriate expression of the suprabasal marker IVL following exit from the basal compartment in EoE (Figure 6A). The analysis of cell proportions revealed an expanded suprabasal population, a reduced number of superficial cells, and a consistent basal compartment (Figure 6C), which is consistent with our scRNA-Seq findings. Notably, 73.8% of EEC in EoE expressed the basal marker p63 in the suprabasal and superficial compartments, while HC primarily exhibited basal-restricted p63 expression (Figure 6, A and D). Thus, our findings indicate that, despite maintaining the correct spatial organization of suprabasal lineage commitment, most suprabasal and superficial EEC in EoE retain a basal identity**.**

### Pseudotemporal analysis confirms a global differentiation shift toward basal identity in EEC in EoE.

**Figure 7:**
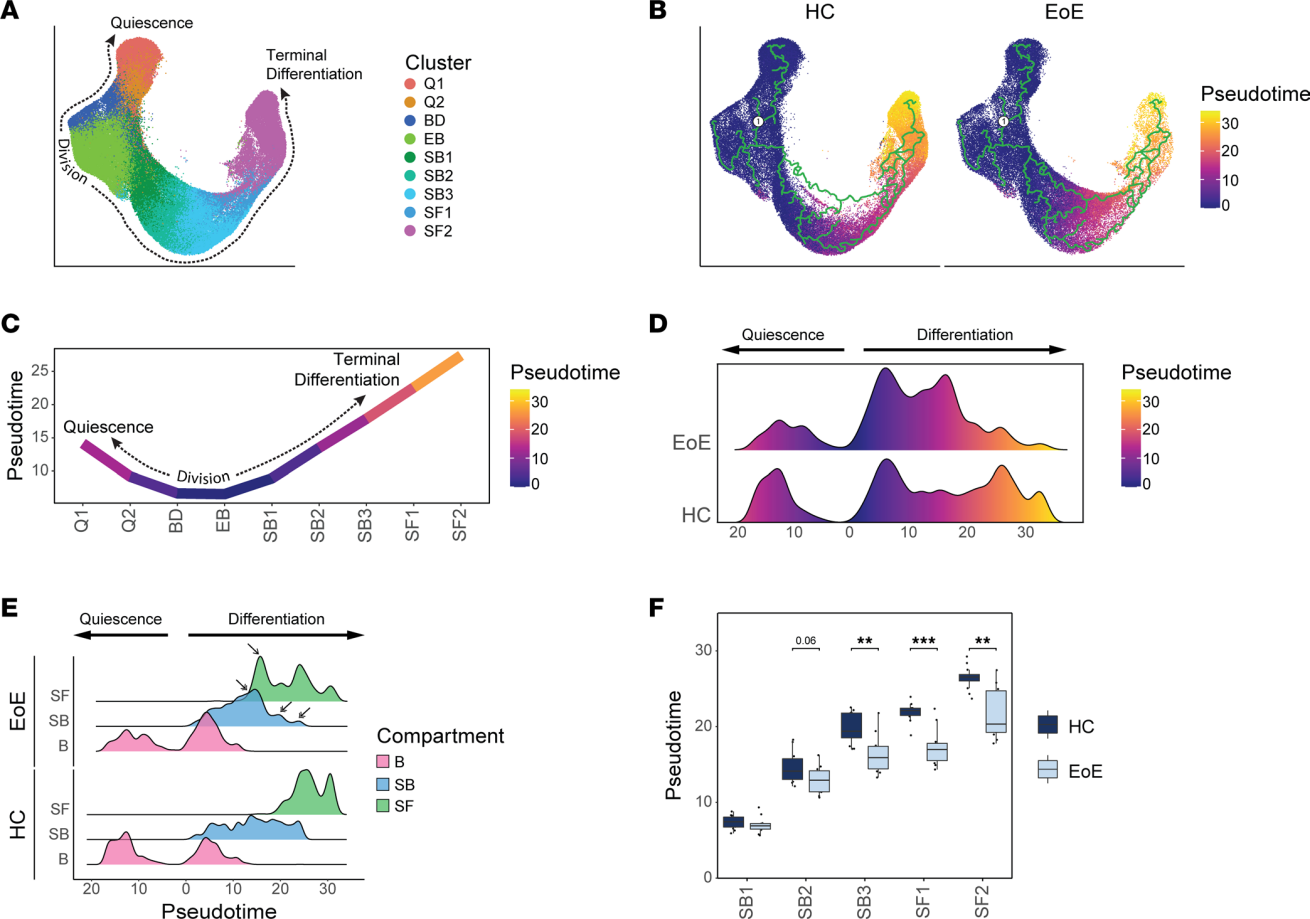
Pseudotemporal trajectory analysis of EEC reveals a shift in global differentiation toward basal identity in EoE. (**A**) UMAP showing merged scRNA-Seq data sets of EEC from HC and EoE, colored by integrated clusters. (**B**) Pseudotime trajectory analysis calculated using HC and EoE samples, with cycling cells as the pseudotime origin. (**C**) Average pseudotime values across epithelial clusters. Pseudotime is rooted in cycling cells and progresses toward either quiescence or terminal differentiation. (**D**) Ridgeline plot illustrating the pseudotime value distribution between EEC in HC and EoE. (**E**) Ridgeline plots depicting pseudotime value distribution between epithelial compartments in HC and EoE. Arrows indicate peaks with differential density between EoE and HC. (**F**) Box plot showing the distribution of pseudotime values for every suprabasal and superficial cell cluster in HC or EoE. Boxes indicate quartiles, whiskers indicate minima/maxima separated by 1.5 times the interquartile range, and lines through each box indicate median pseudotime value. Indicated *P* values were determined using Wilcoxon signed-ranked test with Benjamini & Hochberg adjustment for multiple comparisons. **P ≤ 0.01, ***P ≤ 0.001. B, Basal; Q, Quiescent; BD, Basal_Dividing; EB, Epibasal; SB, Suprabasal; SF, Superficial.

As a complimentary approach to examine changes in cell differentiation, we merged epithelial samples from HC and EoE for pseudotemporal analysis with Monocle3 to examine differences in cell fate trajectories along the course of differentiation (Figure 7A and Supplemental Figure 6A). We followed the established model for EEC ordering and designated S-phase cells that are committed to divide as root cells (26) (Figure 7B and Supplemental Figure 6B), where resulting daughter cells that return to the G0 quiescent reserve (Q1 and Q2) are captured in 1 direction of the trajectory, while daughter cells that commit to differentiation (SB1 through SF2) move in another direction of the trajectory (Figure 7C). Trajectory analysis was performed on healthy and EoE conditions combined (Figure 7B) to allow direct comparison of pseudotime values between conditions. A severe decrease in late pseudotime peaks was observed in EEC in EoE, with a concentration of cells in an intermediate range of pseudotime values instead (Figure 7D). This shift in pseudotime value distribution was consistent across patients with EoE (Supplemental Figure 6C) and was not explained by the decreased frequency of superficial cells (Figure 7E). The comparison of pseudotime densities between epithelial compartments revealed a marked reduction in pseudotime density profiles in the suprabasal and superficial compartments in EoE, compared with HC (Figure 7E). Breakdown of the suprabasal and superficial compartments into component clusters revealed a significant decrease in pseudotime values starting in SB3 in EoE compared with HC (Figure 7F). In fact, hierarchical clustering of mean pseudotime values of each differentiated cluster demonstrated an 87% accuracy in distinguishing EoE from HC using the first 2 dendrogram nodes (Supplemental Figure 6D). This further demonstrates that suprabasal and superficial EEC retain a basal-like identity in EoE, compared with suprabasal and superficial EEC in HC.

**Figure 8:**
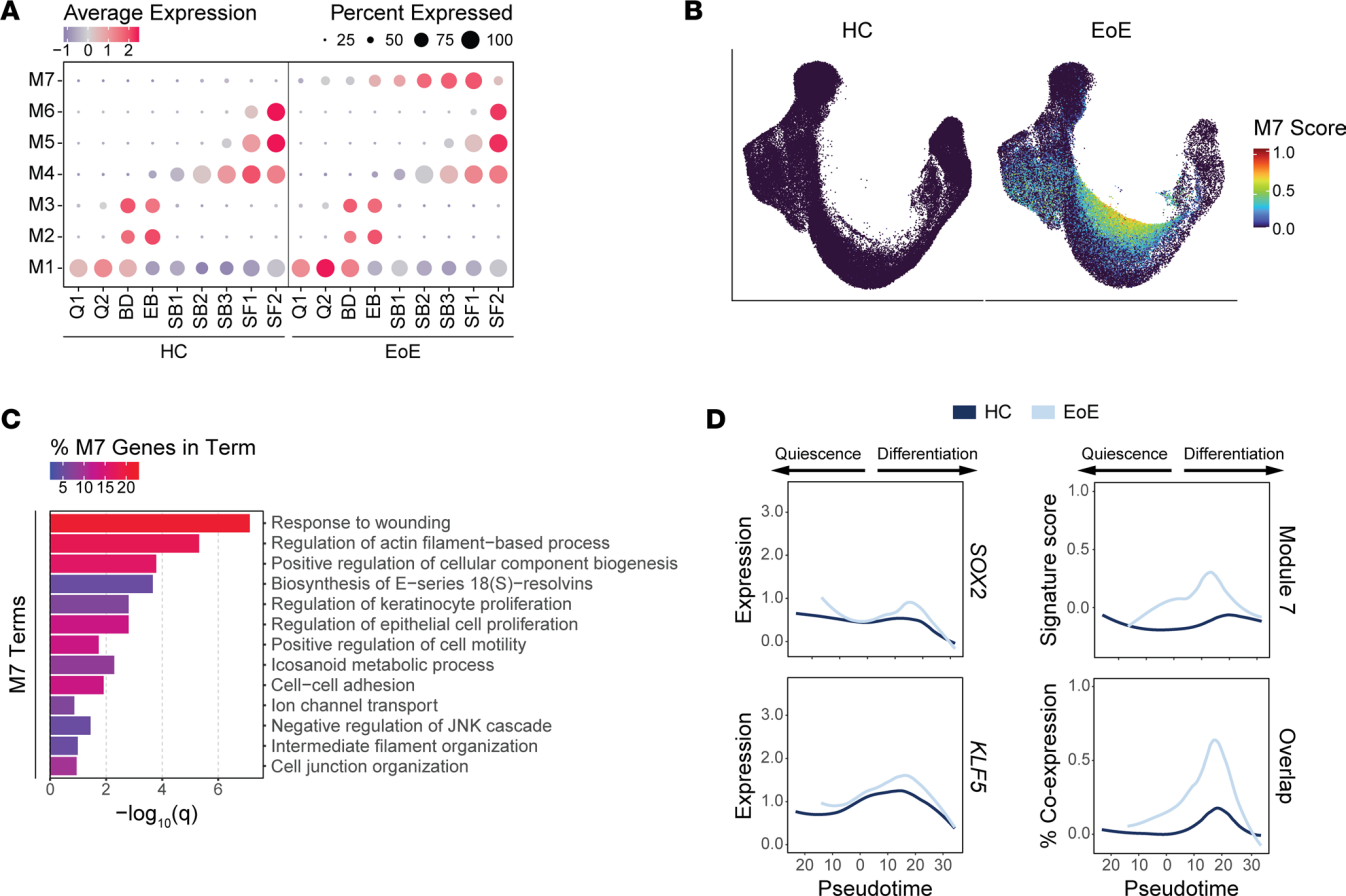
Pseudotemporal trajectory-dependent gene programs are altered in EEC in EoE. (**A**) Average signature score *Z* scores per EoE trajectory–dependent module for each epithelial cluster in HC or EoE. Color intensity indicates relative scoring. (**B**) UMAP of the merged scRNA-Seq data set of EEC from HC or EoE, colored by module 7 gene signature score. (**C**) Enriched pathways for module 7 genes, ranked by *P* value. Color intensity indicates the percentage of module 7 genes along each pathway. (**D**) Expression of SOX2, KLF5, or module 7 genes across EEC and percentage of EEC expressing meaningful levels of all 3 conditions, ordered by pseudotime for both HC and EoE. Lines represent moving averages calculated by LOESS regression. B, Basal; Q, Quiescent; BD, Basal_Dividing; EB, Epibasal; SB, Suprabasal; SF, Superficial.

Next, we identified gene modules that displayed trajectory-dependent gene expression patterns in EoE (Figure 8A, Supplemental Figure 7A, and Supplemental Table 3). Top terms from pathway enrichment analysis are shown for each module in Supplemental Figure 7B. Modules 4, 5, 6, and 7 show different expression patterns between EoE and HC (Supplemental Figure 7C). Modules 4, 5, and 6 were linked to EEC differentiation (Supplemental Figure 7B). Module 7 was particularly interesting as it contained genes with substantially increased expression in EoE in all epithelial clusters (Supplemental Figure 7C; Figure 8, A and B; and Supplemental Table 4), with peak increase in SB2 (Supplemental Figure 7D). Pathway enrichment analysis of module 7 genes identified enriched terms associated with response to wounding, regulation of actin filament-based process, regulation of keratinocyte proliferation, and positive regulation of cell motility (Figure 8C). Mean module 7 gene signature scores show elevated expression along the differentiation trajectory in EoE as compared with HC, peaking at pseudotemporal values representing the differentiated clusters that show earlier pseudotemporal identity in EoE (Figure 8D).

Interestingly, we observed that *SOX2* and *KLF5* expression also increased across a similar pseudotemporal range in EoE compared with HC (Figure 8D). A higher percentage of EEC expressed overlapping *SOX2*, *KLF5*, and module 7 signature scoring in EoE, with the highest level of coexpression within the same range of pseudotemporal values (Figure 8D and Supplemental Figure 7E), suggesting the regulation of module 7 genes by SOX2 and KLF5. All module 7 genes exhibited increased expression in EoE EEC, peaking in clusters SB2 through SF1 (Supplemental Figure 7F). Notably, expression of module 7 genes in EoE peaked in suprabasal and superficial clusters showing aberrant *SOX2* and *KLF5* expression (Supplemental Figure 7F). Furthermore, over 49% of module 7 genes were known epithelial targets of either SOX2, KLF5, or the SOX2-KLF5 interaction (34–37) (Supplemental Figure 7F and Supplemental Tables 5 and 6) with known protein-to-protein interactions (Supplemental Figure 8). This supports our findings regarding the key involvement of SOX2, KLF5, or their interaction in governing the upregulated gene programs identified in the suprabasal and superficial compartments in EoE and suggests that they play a prominent role in disease-associated tissue remodeling.

### SOX2 and KLF5 gene programs are altered in the suprabasal and superficial compartments in EoE.

**Figure 9:**
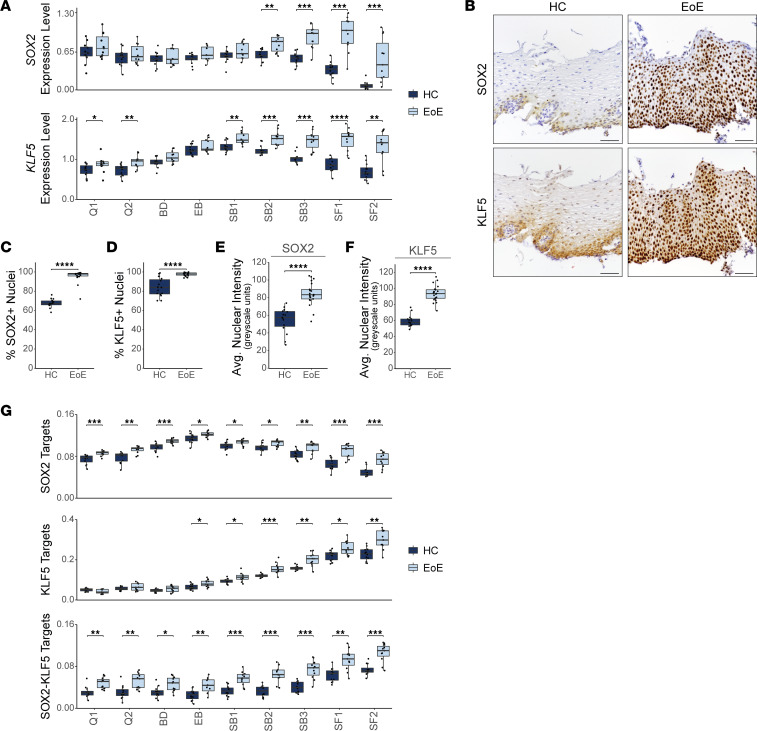
SOX2 and KLF5 gene programs are altered in the suprabasal and superficial compartments in EoE. (**A**) Box plots displaying average *SOX2* and *KLF5* expression for each epithelial cluster in HC and EoE. (**B**) IHC for the indicated proteins in the esophageal epithelium of HC (*n* = 15) and EoE (*n* = 21). Scale bar: 100 μm. (**C** and **D**) Quantification of the positive staining out of total cells for SOX2 (**C**) or KLF5 (**D**). (**E** and **F**) Quantification of the average nuclear intensity across all cells for SOX2 (**E**) or KLF5 (**F**), measured in grayscale units. (**G**) Box plots of average gene signature scores from the transcriptional targets of SOX2, KLF5, or the SOX2-KLF5 interaction in each epithelial cluster in HC and EoE. For all box plots, boxes indicate quartiles, whiskers indicate minima/maxima separated by 1.5 times the interquartile range, and lines through each box indicate median value. Indicated *P* values were determined using Wilcoxon signed-ranked test with Benjamini & Hochberg adjustment for multiple comparisons, applied specifically in **A** and **G**. *P ≤ 0.05, **P ≤ 0.01, ***P ≤ 0.001, ****P ≤ 0.0001. B, Basal; Q, Quiescent; BD, Basal_Dividing; EB, Epibasal; SB, Suprabasal; SF, Superficial.

In addition to our findings that *SOX2* and *KLF5* are coexpressed in EoE, our analysis revealed an increased expression of these TFs in a greater percentage of cells within the suprabasal and superficial EEC clusters in EoE, in comparison with HC (Figure 9A and Supplemental Figure 9, A and B). IHC confirmed increased nuclear expression of SOX2 and KLF5 in suprabasal and superficial EEC in EoE (Figure 9, B–F). Interestingly, KLF5 was recently identified as a SOX2 binding partner, and their interaction led to the acquisition of chromatin binding sites not observed with SOX2 or KLF5 alone (37). Published epithelial-specific SOX2-, KLF5-, or SOX2/KLF5-regulated gene programs (34, 35, 37) were enriched across EEC clusters in EoE, with the most dramatic increase in the suprabasal and superficial clusters (Figure 9G and Supplemental Tables 5 and 7). In total, 1,620 genes known to be regulated by SOX2 and/or KLF5 were significantly upregulated in EEC in EoE (FDR-adjusted *P* < 0.05 and logFC > 0.25), 76.5% of which demonstrated the highest upregulation in the suprabasal and superficial compartments (Supplemental Table 7). Furthermore, in EoE, there was significant dysregulation of 224 genes known to be coregulated by the SOX2-KLF5 interaction (FDR-adjusted *P* < 0.05 and |logFC| > 0.25), with 86.7% of these genes showing upregulation (Supplemental Table 8).

To further investigate the gene targets coregulated by SOX2 and KLF5 displaying elevated expression in EoE, we conducted unsupervised clustering analysis of their expression between HC and EoE compartments (Supplemental Figure 9C). Cluster 1 genes exhibited progressively reduced expression throughout the suprabasal and superficial compartments in HC but showed increased expression in EoE (Supplemental Figure 9C). Enrichment analysis revealed changes related to actin-filament based processes and cell morphogenesis associated with differentiation (Supplemental Figure 9D). Increased cluster 2 gene expression was seen in the suprabasal and superficial compartments in EoE, with relatively low expression in HC (Supplemental Figure 9C). Cluster 2 genes were linked to pathways associated with cell-to-cell junction and actin cytoskeleton organization (Supplemental Figure 9D). These findings demonstrate that the disrupted expression of SOX2 and KLF5, along with their coregulated downstream targets, contribute to epithelial remodeling specifically within the suprabasal and superficial compartments in EoE.

### Dysregulated differentiation and aberrant signaling of progenitor-regulating TFs in EEC are specific to EoE and not observed in GERD.

**Figure 10:**
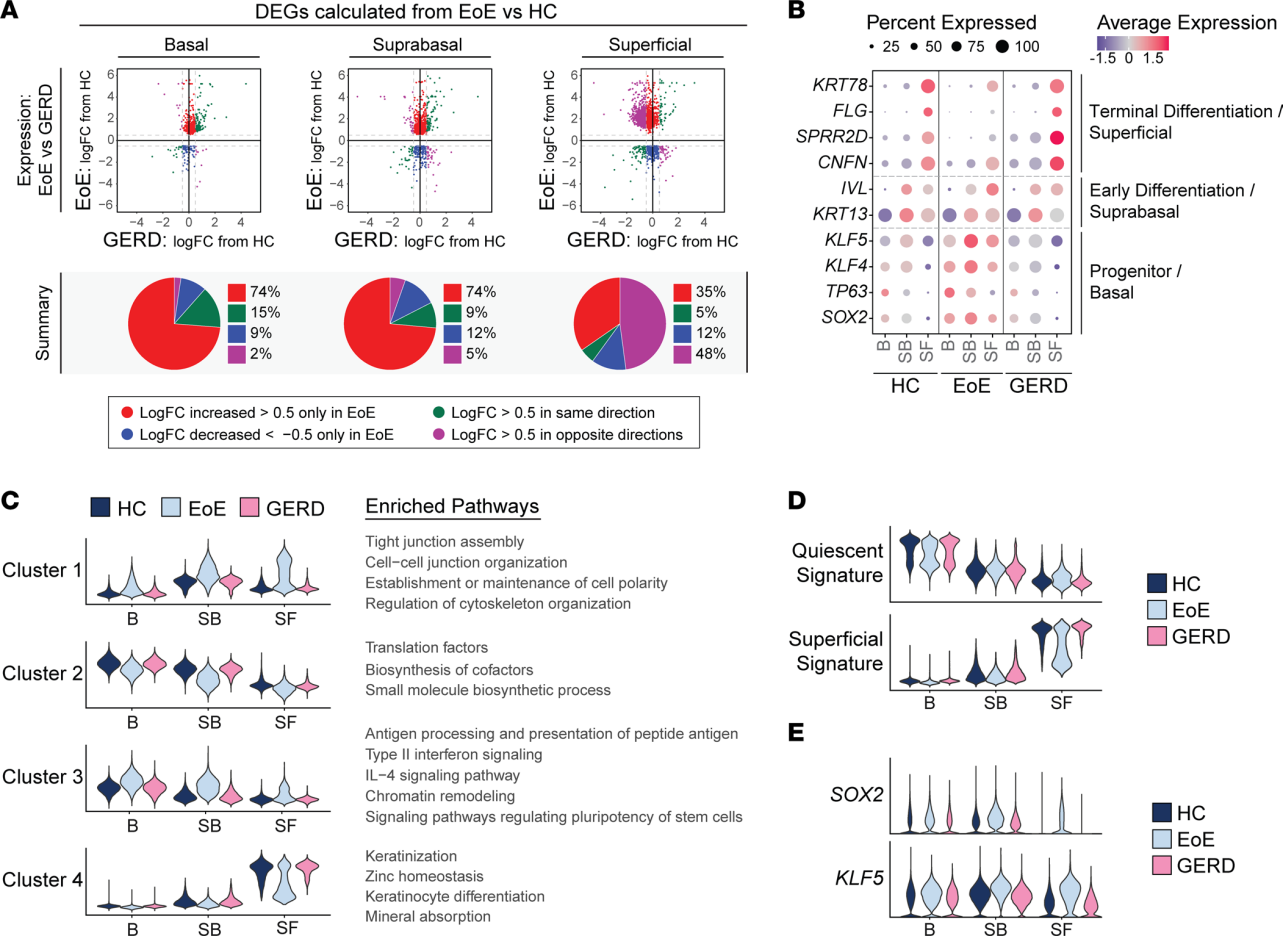
The distinct molecular changes identified at a single-cell level in EEC in EoE are not detected in GERD. (**A**) Scatterplots of DEGs calculated per epithelial compartment between HC and EoE (|logFC| > 0.5, FDR-adjusted *P* < 0.05). Each gene is plotted as logFC in EoE compared with HC (*y* axis) versus logFC in GERD compared with HC (*x* axis). Color indicates the direction and magnitude of logFC in EoE and GERD. Red (logFC > 0.5 only in EoE), blue (logFC < -0.5 only in EoE), green (logFC > 0.5 in EoE and GERD, or logFC < -0.5 in EoE and GERD), and purple (logFC > 0.5 in EoE and < –0.5 in GERD, or logFC < –0.5 in EoE and > 0.5 in GERD). For each compartment, a pie chart summarizes the direction and magnitude of logFCs in EoE and GERD. (**B**) Comparison of average expression *Z* scores of known epithelial transcription factors and differentiation markers in EEC compartments across HC, EoE, and GERD. Color gradient indicates the average gene expression level for each cluster. The dot size corresponds to the percentage of cells within each cluster exhibiting gene expression. (**C**) Violin plots displaying the gene signature scores derived from DEGs specific to suprabasal and superficial compartments in EoE that were hierarchically clustered as shown in Supplemental Figure 10A. Enriched terms associated with each hierarchical cluster are indicated. These scores are shown for each EEC compartment in HC, EoE, and GERD. (**D** and **E**) Violin plots showing quiescent and superficial gene signature scores (**D**) or *SOX2* and *KLF5* expression (**E**) for each EEC compartment in HC, EoE, or GERD. B, Basal; SB, Suprabasal; SF, Superficial; *P*_adj_, FDR-adjusted *P* value.

Given that patients with EoE and GERD present with overlapping symptoms, such as heartburn and dysphagia (38), and that both undergo BCH (39), we next assessed whether the transcriptomic changes observed in EoE are disease specific or influenced by acid reflux. To investigate this, we performed scRNA-Seq on 4 patients with GERD, and we imputed cell identities established from HC and EoE data sets onto GERD EEC. Differential gene expression was calculated between EoE and HC for each epithelial compartment, and logFC from patients with GERD compared with HC was determined for genes significantly altered in EoE (|logFC| > 0.5 and FDR-adjusted *P* < 0.05) within each compartment. As shown in Figure 10A, EEC from GERD and EoE shared only a few genes changing in the same direction. Notably, most EoE DEGs in the basal and suprabasal compartments showed minimal change in GERD (Figure 10A). However, in the superficial compartment, 48% of DEGs displayed opposite changes in GERD (|logFC| > 0.5) (Figure 10A). We next compared known epithelial markers between HC, EoE, and GERD. In contrast to the loss of terminal differentiation observed in EoE, GERD EEC showed the correct expression patterns of early (*KRT13* and *IVL*) and late differentiation markers (*CNFN, SPRR2D, FLG*, and *KRT78*) (Figure 10B).

To comprehensively compare the suprabasal and superficial compartments in EoE and GERD, we calculated differential gene expression between EoE versus HC in these compartments. We then conducted hierarchical clustering of the obtained DEGs and performed pathway enrichment analysis on genes within each hierarchical cluster (Supplemental Figure 10A). Finally, gene signatures were generated from each hierarchical cluster, and scoring was calculated across all HC, EoE, and GERD EEC (Figure 10C). DEGs in clusters 1 and 3 showed increased expression in the suprabasal and superficial compartments in EoE compared with HC but remained unchanged in GERD (Figure 10C and Supplemental Figure 10A). Enriched terms for these DEGs were associated with type II interferon signaling, chromatin remodeling, pluripotency of stem cells, cell junction organization, and cytoskeleton organization (Figure 10C and Supplemental Figure 10A). Similarly, cluster 4 genes related to keratinocyte differentiation showed decreased expression in the superficial compartment in EoE but were not decreased in GERD (Figure 10C and Supplemental Figure 10A).

To assess changes along the quiescent/basal/differentiation axis between GERD, EoE, and HC, we scored EEC using quiescent and superficial gene signatures. In GERD, the superficial compartment demonstrated proper adoption of superficial cell identity and inhibition of basal cell identity, unlike in EoE (Figure 10D and Supplemental Figure 10B). Moreover, the changes observed in the quiescent and superficial cell identity in the clusters SB3-SF2 in EoE were absent in GERD (Supplemental Figure 10B); this was consistent across patients with GERD (Supplemental Figure 10C). Furthermore, we observed no aberrant expression of *SOX2*, *KLF5*, *TP63*, or *KLF4* expression in the suprabasal and superficial compartments in GERD, unlike in EoE (Figure 10, B and E, and Supplemental Figure 10D). Notably, by performing hierarchical clustering of the main features identified in the suprabasal and superficial compartments of patients with EoE, we were able to accurately distinguish healthy individuals and patients with GERD from those with EoE, achieving a 93.3% accuracy at the top-level partition in the dendrogram (Supplemental Figure 11). These findings highlight that the loss of terminal differentiation, the shift toward basal cell identity in the suprabasal and superficial compartments, and the abnormal expression of SOX2 and/or KLF5 are exclusive to EEC in EoE and not attributable to gastric reflux in these patients.

## Discussion

Esophageal homeostasis relies on a careful balance between proliferation, differentiation, and cell death, which is critical for the maintenance of epithelial barrier function. Unfortunately, this process is disrupted in EoE, leading to Th2-mediated eosinophilic inflammation and epithelial remodeling, including loss of differentiation and BCH (9). Understanding the role of BCH in EoE disease progression is essential for improving clinical management and treatment strategies. Previous studies have highlighted the association between BCH and disease severity in patients with EoE (10) and demonstrated that BCH affects > 66% of the esophageal epithelial surface area in these patients (11). Even with treatment, BCH persists in approximately half of patients with EoE and correlates with persistent symptoms and endoscopic findings in histologically inactive patients (10, 11). To investigate the cellular identities and transcriptional processes underlying BCH and altered epithelial differentiation in EoE, we performed scRNA-Seq on esophageal biopsies obtained from adult patients with EoE and HC.

BCH in EoE has been suggested to result from the proliferation of basal cells (29, 40). While we confirmed the expansion of proliferating epibasal cells in EoE, our observations indicate that the morphological changes associated with BCH extend beyond the region of hyperproliferation. Interestingly, our study uncovered that BCH in EoE is marked by the expansion of nonproliferative suprabasal cells. In addition to BCH, EoE is characterized by a more widespread loss of differentiation (8, 13). While we confirmed the reduced expression of terminal differentiation markers in the superficial clusters in EoE, our study revealed a more intricate differentiation dynamic in EoE. Importantly, we demonstrate that suprabasal EEC undergo proper commitment to early differentiation after exiting the basal compartment. Furthermore, we found that suprabasal and superficial EEC in EoE retain a basal-like identity, as supported by earlier pseudotemporal identities and elevated expression of quiescence-associated genes in these epithelial clusters. In a recent study on intestinal injury repair mechanisms, a transient cell population derived from transit amplifying cells exhibited a regenerative stem cell–like transcriptional profile but lacked stem cell capacity (41). The authors coined the term adaptive differentiation to describe this atypical differentiation process that occurred in response to tissue damage to facilitate tissue repair (41). We hypothesize that the enhanced stem-like characteristics observed in suprabasal and superficial compartments in EoE are indicative of an adaptive differentiation process. This process may be triggered by the chronic inflammation in the EoE microenvironment, resembling a tissue-wide wound-healing response. While adaptive differentiation is potentially beneficial for tissue repair in the intestine, further investigation is needed to understand its implications in EoE and its potential contribution to pathology in the presence of chronic inflammation.

Enrichment analysis of upregulated genes in the suprabasal and superficial compartments in EoE revealed the potential regulatory role of SOX2 and KLF5 in BCH, epithelial remodeling, and the maintenance of stem cell identity in differentiated EEC. SOX2 is known for its involvement in stem cell maintenance and self-renewal by suppressing differentiation genes (21, 42, 43). Similarly, KLF5 regulates cell proliferation, migration, differentiation, and stemness (44–46). We confirmed elevated expression of SOX2 and KLF5, which overlapped in suprabasal and superficial EEC in EoE, and observed the upregulation of their target genes. Previous research demonstrates the interaction between SOX2 and KLF5 in the progression from normal tissue to esophageal squamous cell cancer (ESCC), suggesting a potential role in response to tissue injury (37, 47). Pathway analysis of the SOX2/KLF5 targets with increased expression in suprabasal and superficial EEC in EoE revealed terms related to epithelial remodeling. This suggests that SOX2 and KLF5 individually confer a basal identity to suprabasal and superficial EEC in EoE, while their combined signaling regulates gene programs involved in chronic epithelial wound repair. Exploring the mechanistic regulation of the injury response, the development of BCH/adaptive differentiation in EoE, and the upstream factors influencing SOX2 and KLF5 expression in suprabasal and superficial EEC represent complex areas that require further future investigation. Additionally, further studies are needed to explore how available therapeutic interventions can modulate the expression levels of SOX2 and KLF5.

Although our study focused on the interaction between SOX2 and KLF5, we cannot exclude the possibility that SOX2 also interacts with other factors in EoE. For instance, in esophageal and lung squamous cell cancer cell lines, SOX2 and p63 — another TF upregulated in differentiated EEC in EoE — were shown to jointly occupy multiple genomic loci (48). Furthermore, the joint binding of p63, SOX2, and KLF5 was demonstrated to regulate chromatin accessibility, epigenetic modifications, and gene expression in ESCC (40). Furthermore, SOX2 and KLF4 operate as a functional core in pluripotency induction across cells of different origins (49). Thus, additional investigations are needed to explore the interaction of SOX2 with other TFs predicted by our computational analyses in EoE.

Finally, this study also explored the transcriptomic changes at the single-cell level in GERD. Given the overlap in symptoms and histological presentation, particularly the presence of BCH (38), it was crucial to determine whether our findings were exclusive to EoE or applicable to GERD as well. Our results clearly demonstrate that the observed increased basal identity, aberrant SOX2 and KLF5 expression, and abnormal expression of other progenitor-regulating TFs in the suprabasal and superficial compartments are specific to EoE and not present in patients with GERD. Therefore, these changes in EoE cannot be solely attributed to gastric reflux. While our analysis primarily focused on comparing EoE and GERD to HC, the differences in cellular identities and transcriptomics between patients with GERD and HC will be subject to further investigation. It is noteworthy that, while GERD is a risk factor for esophageal cancer development (50), epidemiological studies have not found an association between EoE and esophageal cancer, despite the presence of chronic inflammation (51). In contrast, reflux has been shown to influence other esophageal conditions such as achalasia and scleroderma, which are both associated with a higher susceptibility to esophageal cancer progression (52, 53). Therefore, further exploration of the cellular landscape of EEC in GERD, with a larger cohort of patients, may provide valuable insights into the distinctions among GERD, EoE, achalasia, and scleroderma esophageal diseases and their varying susceptibilities to esophageal cancer progression.

Overall, we believe that our findings will provide future guidance on the development of novel therapeutic approaches for EoE. The development of targeted therapies aiming at promoting proper differentiation of suprabasal cells in EoE could help to restore normal epithelial homeostasis for cases in which the reduction of eosinophilic inflammation is not sufficient to completely restore epithelial tissue integrity or to improve clinical symptoms.

In conclusion, our study uncovered that BCH in EoE is characterized by nonproliferative EEC with a combination of differentiation and stem-like transcriptional features. The involvement of SOX2 and KLF5 as potential key regulators sheds light on the underlying molecular mechanisms driving BCH and adaptive differentiation in EoE. Further exploration of epithelial remodeling and adaptive differentiation holds great promise for advancing our understanding of disease progression and may pave the way for novel therapeutic strategies, particularly for patients who do not respond to conventional antiinflammatory treatments.

## Methods

### Human specimen collection.

HC met asymptomatic criteria including the lack of esophageal symptoms (heartburn, dysphagia, chest pain), history of tobacco use or alcohol dependency, BMI greater than 30 kg/m^2^, or previous treatment with antacids or proton pump inhibitors. Patients with EoE were recruited at the primary visit contingent upon confirmed diagnosis and no history of steroid treatment. Patients with GERD were recruited at the primary visit contingent upon positive Bravo pH testing. Exclusion criteria for EoE and GERD included active severe esophagitis (Los Angeles esophagitis Grade C and above) (54), evidence of mechanical obstruction due to peptic stricture (GERD), long-segment Barrett’s metaplasia, unstable medical illness with ongoing diagnostic workup and treatment, current drug or alcohol abuse or dependency, current neurologic or cognitive impairment that would make the patient an unsuitable candidate for a research trial, severe mental illness, pregnancy and bleeding diathesis, or need for anticoagulation that cannot be stopped for endoscopy. Biospecimen Reporting for improved study quality data including age, sex, and race is detailed in Table 1.

### scRNA-Seq sample preparation, library preparation, and sequencing.

Esophageal mucosal biopsies from the proximal and distal esophagus were processed immediately following collection and treated separately. Tissue was digested in Dispase (Corning) diluted in HBSS containing 10 μM HEPES and 10 μg/mL DNase I at 37°C for 15 minutes with 1,500 rpm agitation, followed by digestion in 0.25% trypsin containing 10 μM HEPES and 10 μg/mL DNase I for 20 minutes at 37°C with agitation. The cell suspension was filtered through a 40 μm strainer followed by 12-minute and 6-minute centrifugation at 500*g* at 4°C. Resuspended pellets were filtered through a 40 μm flowmi filter (SP Bel-Art) and measured for cell count and viability using the Cellometer Auto2000 (Nexcelom Bioscience). All cell suspensions met an 85% minimum viability. In total, 16,000 cells were loaded into the Chromium iX Controller (10X Genomics) on a Chromium Next GEM Chip G (10X Genomics) to capture ~10,000 cells per sample and were processed for encapsulation according to the manufacturer’s protocol. The cDNA and library were generated using the Chromium Next GEM Single Cell 3′ Reagent Kits v3.1 (10X Genomics) and Dual Index Kit TT Set A (10X Genomics) according to the manufacturer’s manual. Quality control was performed by Agilent Bioanalyzer High Sensitivity DNA kit (Agilent Technologies) and Qubit DNA HS assay kit (Invitrogen) for qualitative and quantitative analysis, respectively. The multiplexed libraries were pooled and sequenced on Illumina Novaseq 6000 sequencer (Illumina) with 100 cycle kits using the following read length: 28 bp Read1 for cell barcode and UMI, and 90 bp Read2 for transcript. Library preparation and sequencing was done at Northwestern University NUSeq facility core. The GRCh38 transcriptome was used as a reference for alignment and feature counting using Cell Ranger (V4.0.0/6.0.0/6.1.0, 10X Genomics).

### Data filtering, integration, and clustering.

Filtered matrix files were processed as Seurat objects in the Seurat R package 4.2.0 (55) with a minimum threshold of expression in ≥ 5 cells per gene. Each data set was filtered to exclude cells with total gene counts < 400 and total unique gene counts < 100. Data sets were individually normalized, scaled, and processed to calculate variable features using Seurat’s SCTransform workflow. Stricter quality control filtering was performed across all samples to remove cell populations with low total counts of unique genes or cell populations with high mitochondrial gene percentage (mean > 25%) following integration. Individual filtered samples were then integrated using reverse PCA dimensional reduction. Dimensionality reduction was performed followed by calculation of UMAP embeddings, nearest neighbors, and graph-based clustering. Clusters were annotated according to the expression of known cell-specific gene markers and were confirmed against the transcriptional profiles identified by Seurat’s function FindAllMarkers.

### Epithelial cluster and compartment identification.

Epi were subsetted and reintegrated on a per-sample basis using the Seurat integration pipeline described above. Integration anchors were calculated against HC samples as reference. PCA was performed, and the first 30 PCs were included for downstream analysis. Optimal clustering resolution of 0.5 was determined using Clustree. Quiescent (clusters 1 and 2) and cycling clusters (clusters 3–5) were subclustered to distinguish cycling basal cells (*DST*^+^, *MKI67*^+^) from cycling epibasal cells (*DST*^–^, *KRT13*^lo^, *MKI67*^+^). Epithelial clusters were annotated according to expression of known genes in HC as previously described (26) and confirmed against the transcriptional profiles identified by FindAllMarkers, performed on HC cells. Clusters were combined into parental epithelial compartments (B, SB, SF) based on the expression of established markers (8, 15, 16, 26).

### Cell cycle and proliferation analysis.

Seurat’s function CellCycleScoring (56) was used to assign the cell cycle phase of each cell. Cells exhibiting a weak predicted score for S and G2/M were classified as G0/G1 phase. Expression of the markers *KRT15* and *DST* identified Q1 and Q2 epithelial clusters as quiescent and distinguished the G0 from the G1 phase. During the SCTransform workflow, cell cycle was not regressed, allowing EEC to cluster based on quiescence, S-phase, G2/M-phase, and progressive stages of differentiation, confirmed using the expression of marker genes and cell cycle scoring for each cluster. Cell proportion in each cluster was used to assess proliferation rates.

### Detection of DEGs, gene expression analysis, and gene set enrichment analysis.

Identification of DEGs between cell clusters was performed using FindAllMarkers, with filtering for significantly upregulated genes with |logFC| > 0.25. For differential expression analysis comparing expression profiles between like cell identities across disease conditions, the per-sample population mean gene expression was calculated from the normalized RNA assay. Tested genes were filtered by a lower minimum percentage (min.pct) threshold of 5%–10%, which is the percentage of cells expressing a given gene per cell group. The R package edgeR 3.36.0 (57–59) was utilized to create a DGEList object, followed by calculation of normalization factors and counts per million. The logFC was computed, and significance was determined using the Wilcoxon rank-sum test, with FDR *P* value adjustment to correct for multiple comparisons. DEGs were filtered based on an FDR-adjusted *P* < 0.05 and |log_2_FC| > 0.25, unless a more stringent threshold was specified. To visualize the percentage of cells expressing a gene across clusters, the percentage expression in each cluster was calculated using a minimum expression threshold to filter cells with negligible expression of the gene. Pathway enrichment analyses were performed on DEGs filtered for logFC and significance based on FDR-adjusted *P* value, as mentioned above. The analysis of positively and negatively regulated DEGs was completed using the Ingenuity Pathway Analysis (IPA, Qiagen) software. For the analysis of DEGs changing in only 1 direction, pathway enrichment was performed with either Metascape or ClusterProfiler (60–62).

### TF analysis.

TF analysis was performed using the R package EnrichR 3.1.0 (32, 63, 64). To identify upstream TFs that regulate EoE-specific gene programs, DEGs were calculated across all EEC between disease conditions and filtered for |logFC| > 1 and FDR-adjusted *P* < 0.05. Hierarchical clustering was performed on population *Z* scores of DEGs across healthy and EoE epithelial compartments. Relevant hierarchical clusters were selected and used as input for EnrichR analysis with either the ChEA3 2022 ChIP-Seq database or the TF Perturbations followed by Expression GEO Signature database (https://maayanlab.cloud/Enrichr/#libraries).

### Heatmap visualization, population Z score calculation, and hierarchal clustering.

Gene sets displayed in heatmaps, including gene sets incorporated from external sources, were confirmed as changed in EoE with differential expression testing filtered based on FDR-adjusted *P* < 0.05 and minimum logFC threshold. To calculate population *Z* scores, average population expression values were derived from the normalized RNA assay and scaled by the mean and SD calculated across all populations. All heatmaps show population *Z* scores unless otherwise indicated. Hierarchical clustering using the hclust function from the R Stats package 3.6.2 was performed on population *Z* scores, using the ward.D2 clustering method (65, 66) and the Pearson distance method (67, 68). Heatmaps were generated using the R package Complex Heatmap 2.10.0 (67, 68).

### Gene signature score analysis and functional analysis.

Gene signatures were generated using Seurat’s function AddModuleScore. Quiescent and superficial gene signatures were defined using HC cells from our scRNA-Seq data set. Differential expression analysis was performed comparing either quiescent epithelial clusters (Q1 and Q2) or superficial clusters (SF1-SF2) to the remaining epithelium. DEGs were filtered for FDR-adjusted *P* < 0.05 and ranked by logFC, with the top 100 selected. Quiescent and superficial signature scores were plotted using the ggplot2 R package’s geom_density_2d function (69), with consistent binning applied across all compared conditions. TF-regulated gene signatures were identified via enrichment analysis (EnrichR) (32, 63, 64) or sourced from external experiments (Supplemental Table 5). The data sets included in this analysis were previously published (34, 35, 37). Gene signatures were also calculated from coexpressed gene modules identified using Monocle3 that were also used as input to the stringDB R package (70) to infer protein-to-protein interactions.

### Pseudotime analysis.

Pseudotime analysis was performed using the R package Monocle3 1.0.0 (71–73). Individual samples were log_2_ normalized, scaled, merged using Seurat’s merge function, dimensionality reduced, and batch corrected using the fast mutual nearest neighbors (FMNN) method by individual sample. UMAP embeddings were calculated. A CellDataSet object was created with normalized and scaled counts for 2,000 variable genes and reduction feature loadings calculated by FMNN. Monocle3’s function learn_graph was used to infer a trajectory graph from the UMAP embeddings, with a Euclidean distance ratio of 1, a geodesic distance ratio of 0.5, and a minimum branch length of 10. Cells within the S-phase epithelial cluster were assigned a root state of pseudotime 0. Increasing pseudotime values of cells committed to becoming quiescent are depicted to the left on pseudotime axes, and pseudotime values of cells committed to differentiation are depicted to the right on pseudotime axes.

For EoE samples only, Monocle3’s function graph_test was utilized to identify genes with differential expression along the trajectory. Identified genes were clustered into modules of coexpressed genes with corresponding gene signatures calculated. To determine the most represented module in each cell, each module gene signature was scaled and centered between –2 and 2 across all cells. Each cell was assigned to the module exhibiting the highest scaled scoring. To visualize the expression of genes or signatures across pseudotime-ordered cells, we plotted the gene expression or gene signature score for each cell and calculated local mean expression values using local weighted regression fitting of the data by the locally estimated scatterplot smoothing (LOESS) method. For the calculation of coexpression, cells were assessed on a binary basis for expression of all examined genes or gene signatures (value = 1) or expression of less than all or none of the examined genes or gene signatures (value = 0). The values were plotted for each cell ordered in pseudotime, and local mean values were calculated using the LOESS method.

### Imputation of cell populations in the GERD scRNA-Seq data from the EoE and HC scRNA-Seq data set.

An imputation was performed on each cell in the processed GERD epithelial data set to determine the analogous cell population in the integrated HC and EoE epithelial data set using Seurat’s MapQuery function. Cluster labels were assigned based on the maximum prediction score for each of the query cells.

### IHC and scoring.

Immunostaining was performed on formalin-fixed, paraffin-embedded (FFPE) esophageal mucosal biopsies as previously described (45). Briefly, heat-induced antigen retrieval was performed for 30 minutes in Buffer A (Electron Microscopy Sciences, pH 6). Tissue sections were blocked using 0.3% H*_2_*O_2_, streptavidin/biotin incubation, and Starting Block blocking buffer (Thermo Fisher Scientific). Primary and secondary specific antibodies were added (Supplemental Table 9), and detection was performed as previously described (45). Images were acquired on a Nikon Eclipse Ci microscope with a Nikon DS-Ri2 camera and NIS Elements software. H&E staining was performed by the Robert H. Lurie Comprehensive Cancer Center Pathology Core. Image analysis was performed using Fiji software (74). H&E-stained slides were evaluated for BCH according to EoE-HSS (11). For staining quantification, positive cell fraction was calculated as the percentage of positively stained cells compared with the total cell count. For intensity quantification, nuclei were identified by thresholding, mask conversion, watershed segmentation, and particle analysis, followed by measurement of average inverted intensity (grayscale units) after background subtraction.

### Multispectral fluorescence staining and imaging.

Multispectral fluorescence staining was performed using the Opal 6-Plex Detection kit (Akoya Biosciences) using FFPE tissue sections. Slides were baked at 60°C for 15 minutes and deparaffinized with the Leica Bond Dewax solution (Leica Biosystems), followed by heat-based antigen retrieval using Bond Epitope Retrieval Solution 1 (Leica Biosystems) for 30 minutes. Using the Leica Bond Rx Automated Stainer (Leica Biosystems), slides were incubated with primary antibodies followed by the appropriate secondary horseradish peroxidase–conjugated polymer. Incubation was next performed with a unique Opal dye permitting fluorophore covalent bonding to the horseradish polymer. Heat-based retrieval with Bond Epitope Retrieval 1 (Leica Biosystems) was finally performed for 20 minutes. Slides were subjected to sequential rounds of staining. Primary antibodies, concentrations, and associated fluorophores are detailed in Supplemental Table 9. Sections were counterstained with Spectral DAPI and mounted with ProLong Diamond Antifade Mountant (Thermo Fisher Scientific). Images were acquired using the Vectra3 microscope (Akoya Biosciences) and Phenochart Whole Slide Viewer (Akoya Biosciences). Postacquisition image adjustments were performed using InForm Automated Image Analysis Software (Akoya Biosciences) and Fiji (74).

### Statistics.

Statistical analyses were performed using R version 4.1.1. Descriptive statistics are displayed as mean ± SEM for continuous variables unless otherwise described and as frequency counts for categorical variables. For nonnormally distributed continuous data, Wilcoxon rank-sum test was used. When testing multiple conditions, multiple-comparison adjustment was employed. *P* < 0.05 was considered statistically significant.

### Study approval.

Procedures using human tissue were performed by the Digestive Health Foundation Biorepository with approval from the Northwestern IRB (study STU00208111). Written informed consent was received prior to participation.

### Data availability.

All raw sequencing files and processed barcode and feature matrices used within the article are deposited in NCBI’s GEO database under accession code GSE218607. All supporting analytic code is available at the “scRNA-Human_EoE_Esophagus” repository hosted by the Tetreault Lab on GitHub (https://github.com/Tetreault-Lab/Tetreault-scRNA-Human_EoE_Esophagus-2023; commit ID f51f957). All other supporting data are available within the article, supplement, or Supporting Data Values or from the corresponding author upon reasonable request.

## Author contributions

MHC was involved in designing research studies, conducting experiments, acquiring data, analyzing data, and writing the manuscript. ALK was involved in designing research studies and analyzing data. PJK, DAC, NG, and JEP were involved in designing research studies and writing the manuscript. DRW and KAW were involved in designing research studies, analyzing data, and writing the manuscript. MPT was involved in designing research studies, analyzing data, providing reagents, and writing the manuscript.

## Supplementary Material

Supplemental data

Supplemental tables 1-9

Supporting data values

## Figures and Tables

**Table 1:**
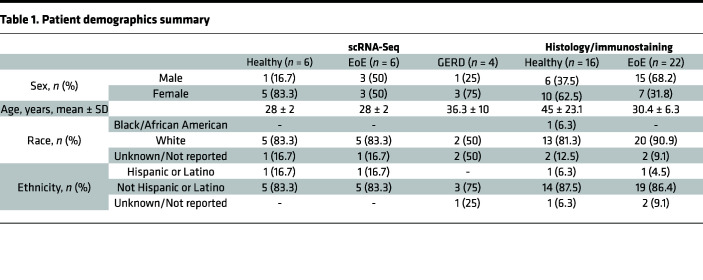
Patient demographics summary

## Version 1 09/06/2023

In-Press Preview

## Version 2 10/09/2023

Electronic publication
