# Supplementary material for: Suprabasal cells retain progenitor cell identity programs in eosinophilic esophagitis–driven basal cell hyperplasia
Source: JCI Insight. 2023 Oct 9;8(19):e171765. doi: 10.1172/jci.insight.171765 (PMC10619442; doi:10.1172/jci.insight.171765)
Supplement: Supplemental data [file jciinsight-8-171765-s078.pdf]

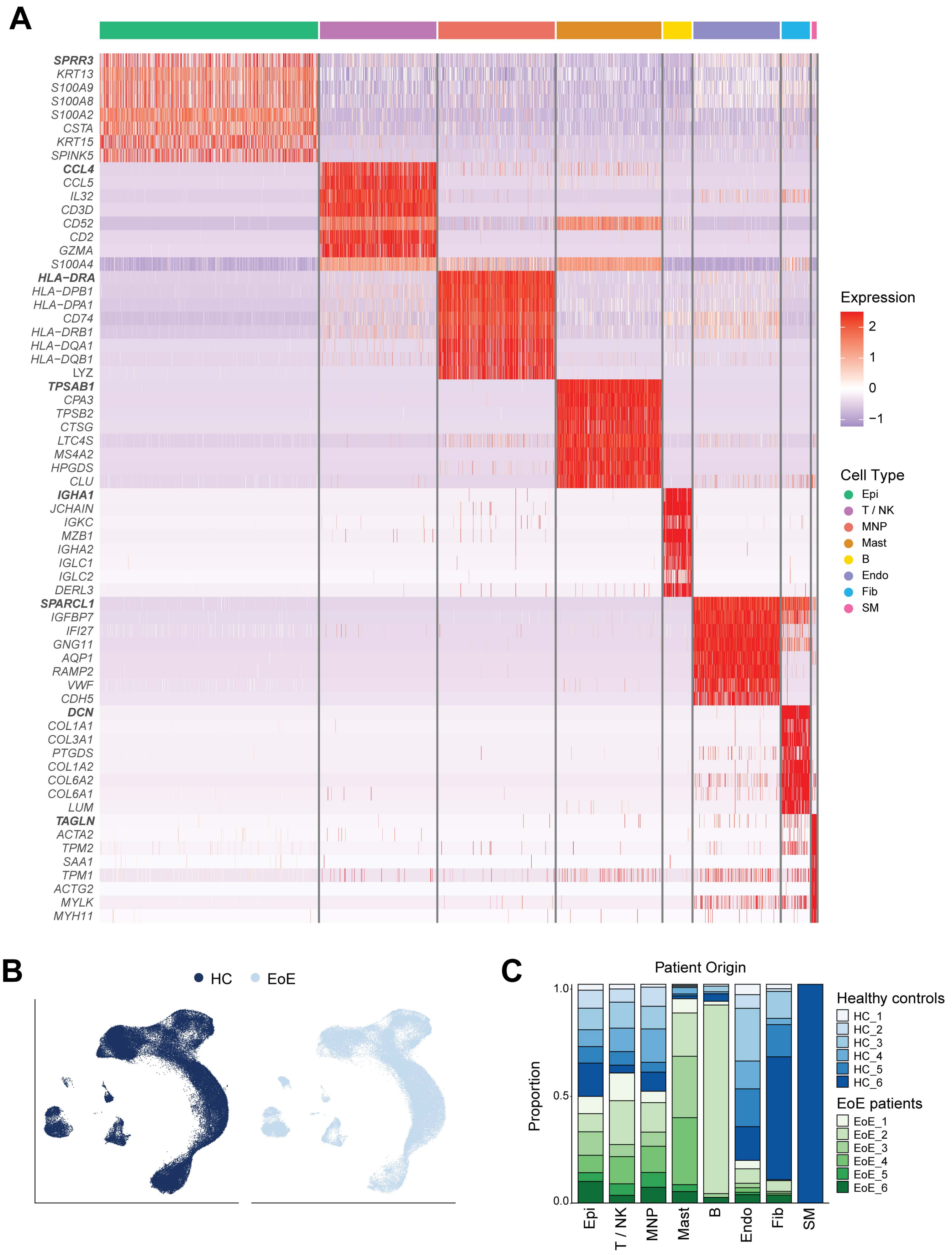

Supplemental Figure 1. Characteristics of esophageal mucosal cells from EoE patients and healthy controls. (**A**) Heatmap of the top 8 genes expressed per cell type. Ranked by average logFC, FDR < 0.05, row-normalized expression z-scores. (**B**) UMAP embedding of scRNA-seq data separated by HC or EoE condition. (**C**) Bar plot depicting the frequency of each esophageal cell type in individual HC and EoE samples. Epithelial cells (Epi), T cells / natural killer cells (T / NK), mononuclear phagocytes (MNP), mast cells (Mast), B cells (B), endothelial cells (Endo), fibroblasts (Fib), or smooth muscle (SM).

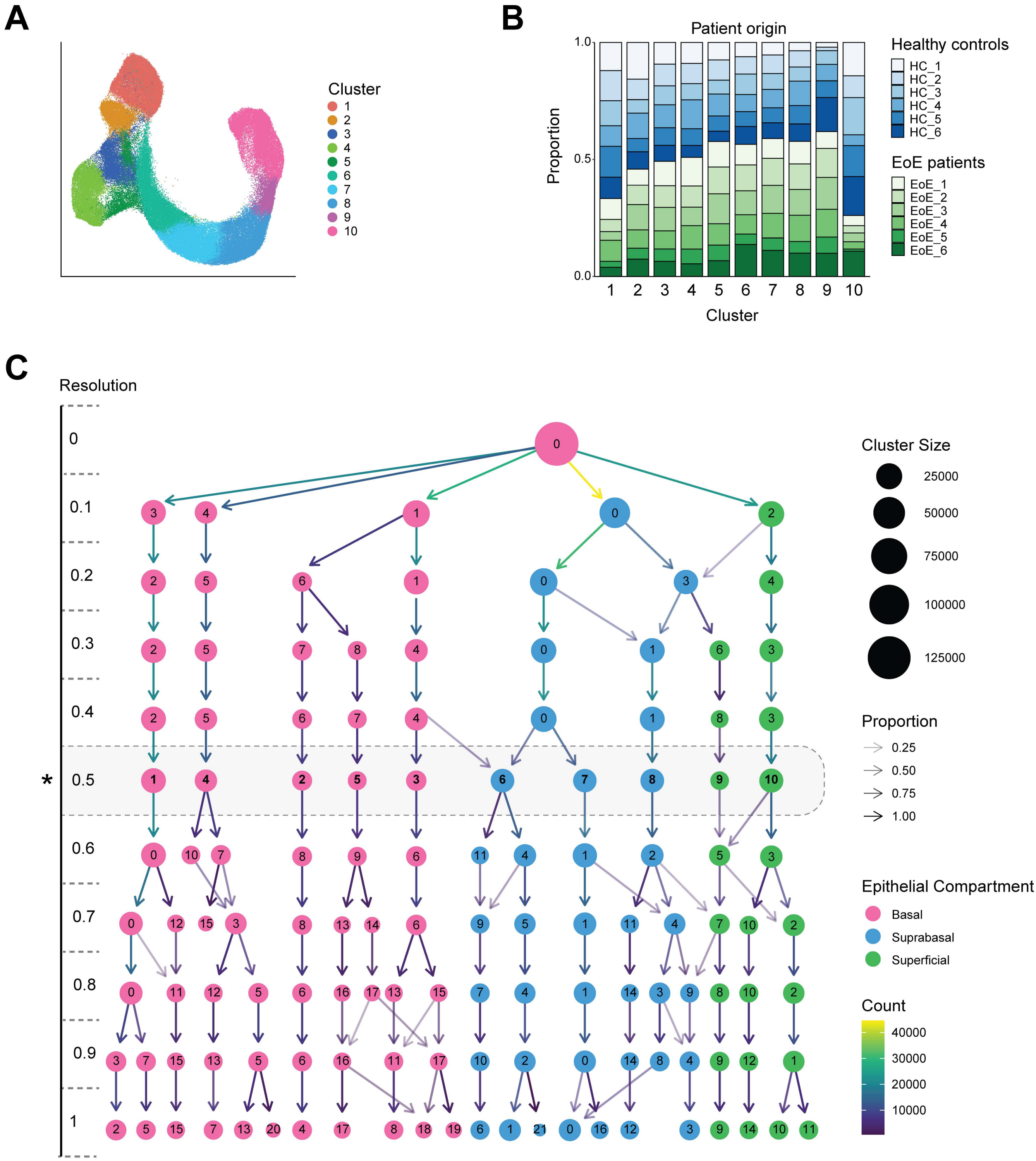

Supplemental Figure 2. Clustering of human esophageal epithelial cells in HC and EoE. (**A**) UMAP of the subclustering of epithelial populations, colored by results of unsupervised graph-based clustering. (**B**) Bar plot depicting the frequency of each epithelial cluster in individual HC and EoE samples. (**C**) Clustering tree of shared nearest neighbor clustering on the epithelial dataset using 30 principal components across resolutions 0-1 with 0.1 increments. Dot size indicates cell count of each cluster. Arrow transparency denotes proportion of cells moving to the indicated cluster in the next resolution increment as function of total cells in the originating cluster. Arrow color indicates cell counts moving to the indicated cluster. Dot color indicates epithelial compartment identity of most cells in each cluster. Star represents the selected resolution.

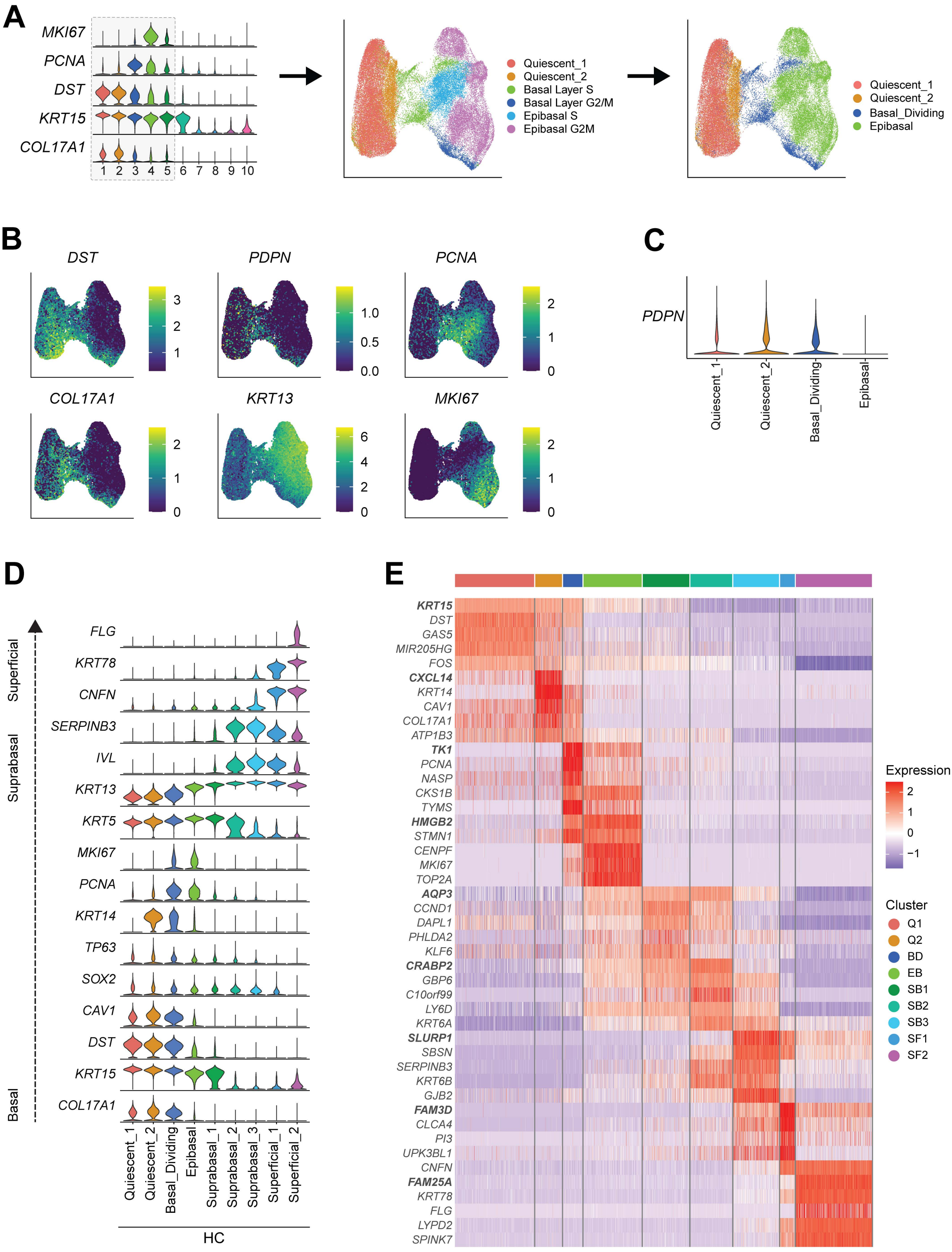

Supplemental Figure 3. Annotation of esophageal epithelial cell clusters. (**A**) Left: Violin plot showing the expression of established markers associated with quiescence and proliferation in each epithelial cluster in healthy controls. Center: UMAP of the subclustering of epithelial clusters 1-5 colored according to basal layer or epibasal layer identity and cell cycle phase. Right: UMAP of the subclustering of epithelial clusters 1-5 colored according to basal layer or epibasal layer identity and quiescent or proliferating state. (**B**) Visualization of the color-coded log1p normalized expression of the epithelial markers *DST*, *COL17A1*, *PDPN*, *KRT13*, *PCNA*, or *MKI67* on the UMAP of the quiescent and proliferating epithelial clusters. (**C**) Violin plot showing *PDPN* expression across identified cell populations within the basal and epibasal layers. (**D**) Violin plot showing expression of established epithelial basal and differentiated markers across annotated epithelial clusters in healthy controls. (**E**) Heatmap of the top 5 genes expressed for each epithelial cluster. Ranked by average logFC, FDR < 0.05, row-normalized expression z-scores. Basal (B), Quiescent (Q), Basal_Dividing (BD), Epibasal (EB), Suprabasal (SB), Superficial (SF).

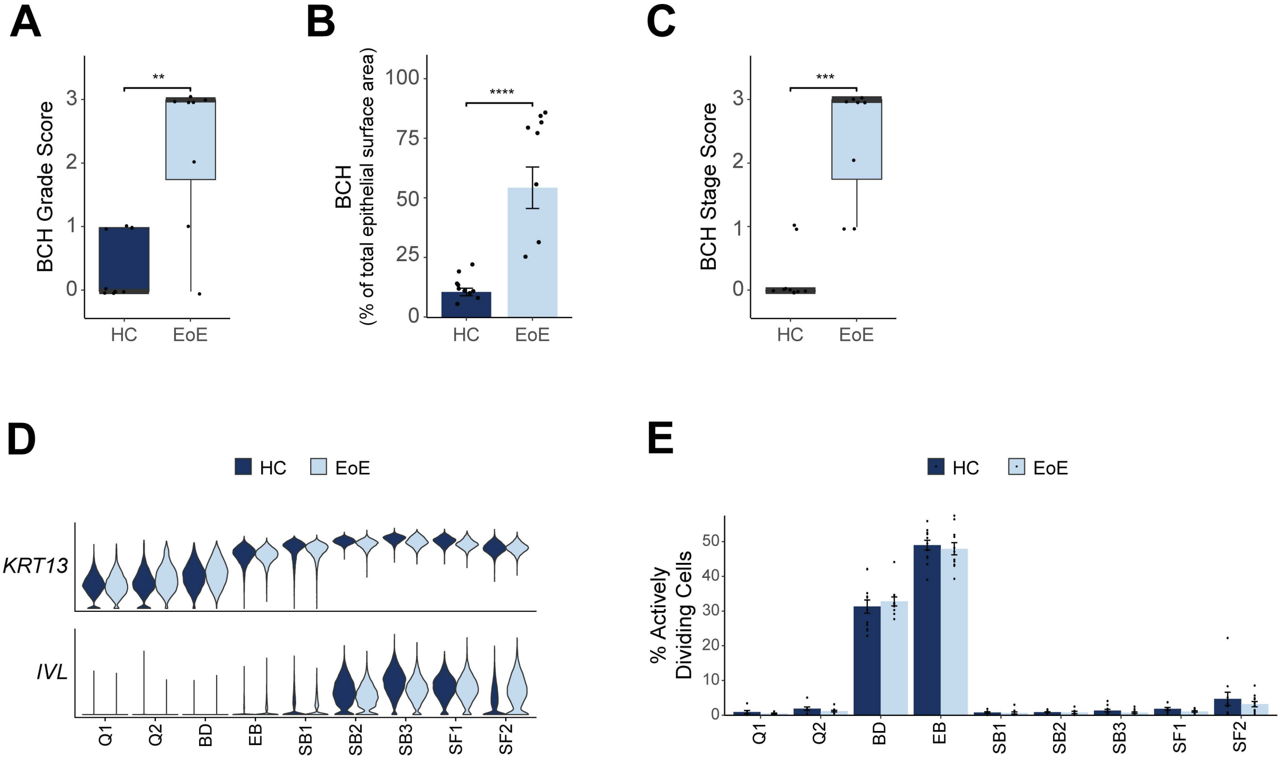

Supplemental Figure 4. BCH scoring and characterization of the proliferation and differentiation states in each esophageal epithelial cell cluster in EoE. (**A-C**) Quantification of BCH grade score (**A**), percentage of BCH out of total epithelial surface area (**B**) and BCH stage score (**C**) was performed on esophageal epithelial sections from HC and EoE samples from the scRNA-seq cohort. (**D**) Violin plots showing the expression of the suprabasal markers *KRT13* or *IVL* in EEC clusters in HC or EoE. (**E**) Bar plot depicting the frequency of actively dividing cells in cell cycle phase G2/M in each EEC cluster in HC and EoE patients. For (**A, C**), boxes represent quartiles, whiskers indicate minima/maxima, lines through each box denote median score. For (**B, E**), data are shown as mean ± SEM. For (**A-C**), indicated *P* values were determined using Wilcoxon signed-ranked test. $^{**}P \leq 0.01$, $^{***}P \leq 0.001$, $^{****}P \leq 0.0001$. Basal (B), Quiescent (Q), Basal_Dividing (BD), Epibasal (EB), Suprabasal (SB), Superficial (SF).

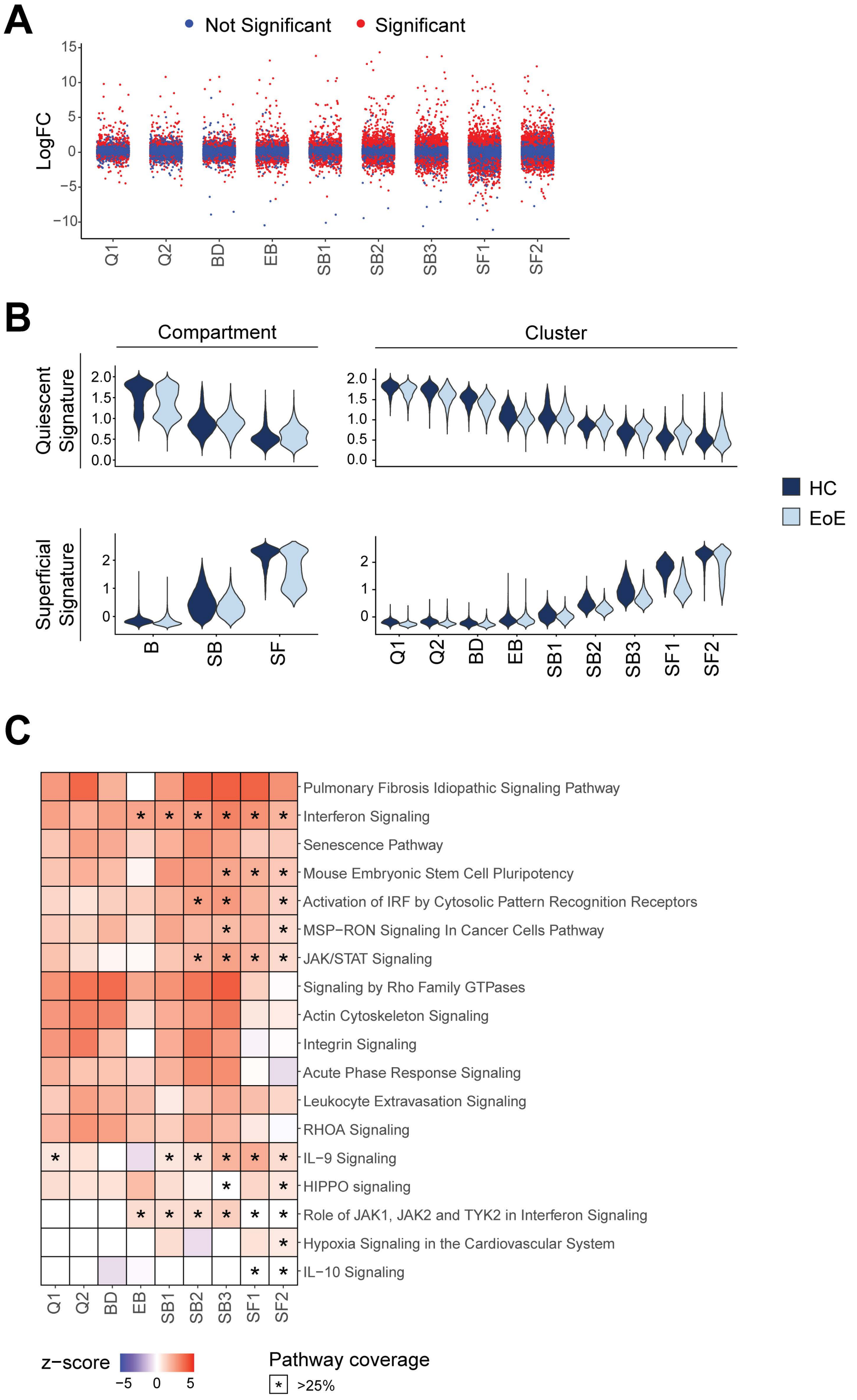

Supplemental Figure 5. Alteration of the quiescent-basal-differentiation transition process in the esophageal epithelium in EoE. (**A**) LogFC of DEGs in each epithelial cluster calculated in EoE patients as compared to HC. Significantly changed genes (FDR < 0.05) are indicated in red. (**B**) Violin plots showing the scores for quiescent gene signature and superficial gene signature between disease states in either epithelial compartments or epithelial cell clusters. (**C**) Ingenuity pathway analysis identified canonical pathways significantly altered in EEC populations in EoE compared to HC. Color intensity indicates activation z-score of each term; red denotes activation, blue denotes inhibition. Stars indicate pathway coverage > 25%. Basal (B), Quiescent (Q), Basal_Dividing (BD), Epibasal (EB), Suprabasal (SB), Superficial (SF).

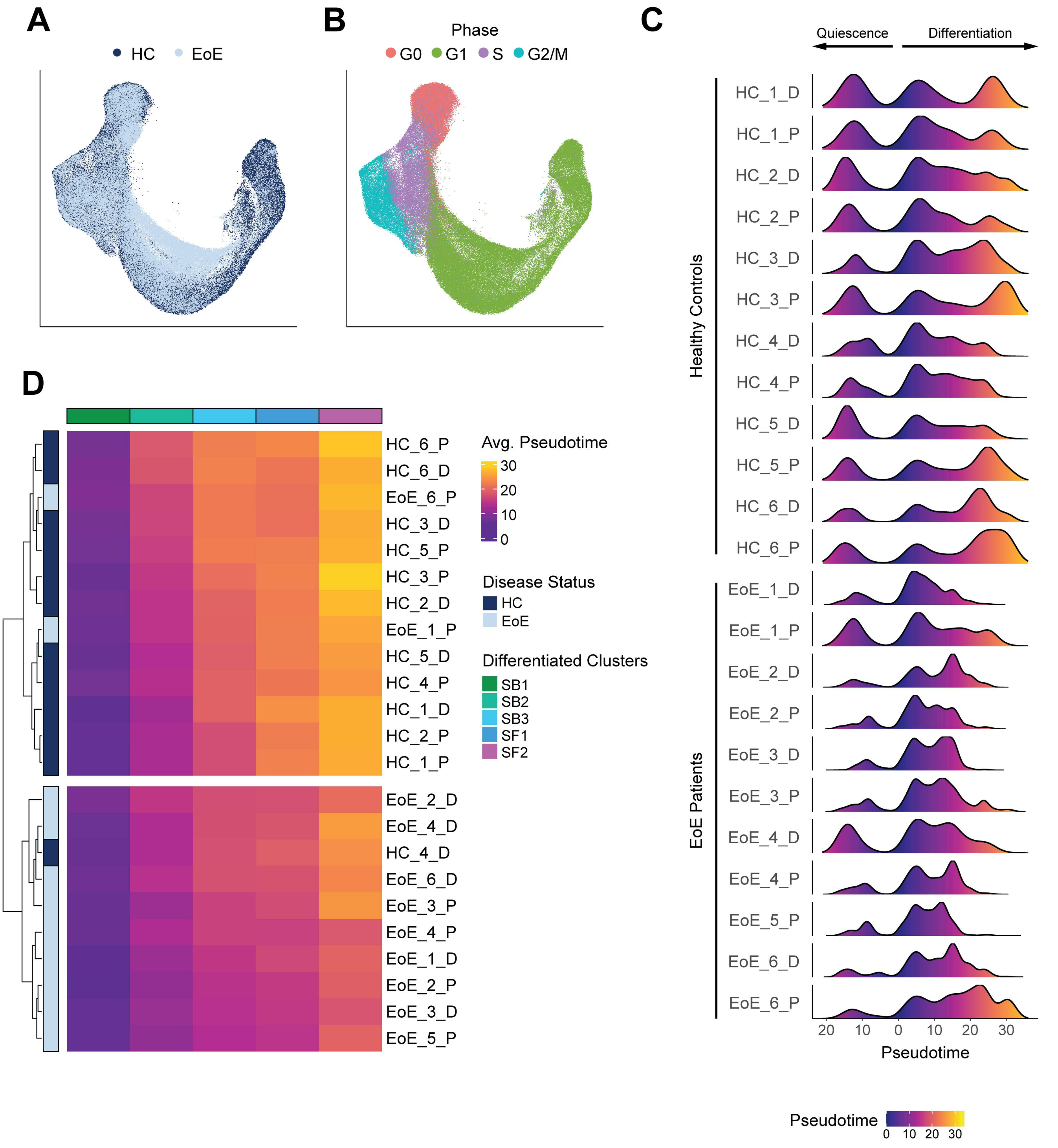

Supplemental Figure 6. Average epithelial cluster pseudotime assignment can distinguish EoE from HC patient samples. (**A**-**B**) UMAP showing merged scRNA-seq datasets of EEC from EoE and HC patients, colored by disease state (**A**) or cell cycle phase (**B**). (**C**) Ridgeline plots showing the distribution of pseudotime values between epithelia of individual samples from HC or EoE patients. (**D**) Heatmap of raw (non-scaled) average pseudotime values for each suprabasal and superficial epithelial cluster in EoE and HC patients, analyzed by hierarchical clustering. The two top-level hierarchical clusters are spatially separated for emphasis. Basal (B), Suprabasal (SB), Superficial (SF).

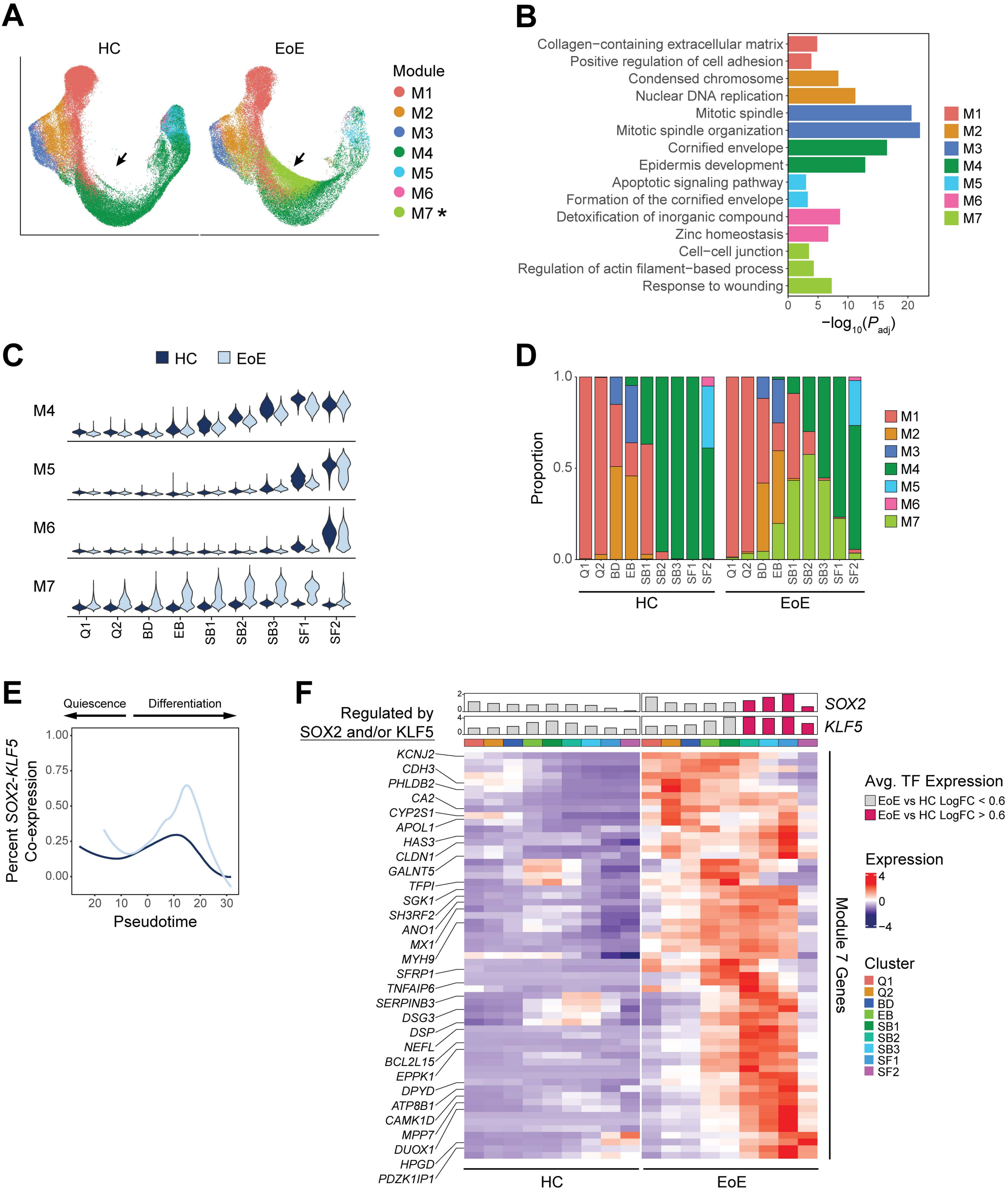

Supplemental Figure 7. Trajectory-dependent gene programs altered in EoE mediate tissue remodeling and may be downstream of SOX2 and/or KLF5. (**A**) UMAP split by disease state, with cells colored by highest-scoring pseudotime-dependent module. Arrows indicate module 7. (**B**) Bar plot showing significantly enriched pathways as identified by pathway enrichment analysis for each EoE-trajectory-dependent gene module. Color indicates associated module. (**C**) Violin plots illustrating the gene signature score of modules that demonstrated significant alterations between HC and EoE epithelial clusters. (**D**) Stacked bar graph depicting the composition of epithelial clusters in EoE and HC, segmented by the proportion of cells exhibiting the highest score for each of the seven EoE-trajectory-dependent modules. (**E**) The percentage of epithelial cells exhibiting co-expression of *SOX2* and *KLF5* is shown in HC and EoE patients across cells ordered by pseudotime. Lines represent moving averages calculated by loess regression across all cells. (**F**) Heatmap of genes in module 7 shown across epithelial clusters, separated by disease state. Data is shown as row-normalized expression z-scores, and the genes known to be regulated by SOX2 and/or KLF5 are annotated. For each epithelial cluster, the mean $\log_2$ gene expression for *SOX2* and *KLF5* is shown in the top annotation as a bar plot. Bar color indicates whether logFC between EoE and HC clusters is above (magenta) or below (grey) 0.6-fold. Basal (B), Quiescent (Q), Basal_Dividing (BD), Epibasal (EB), Suprabasal (SB), Superficial (SF), Module (M), Transcription Factor (TF), adjusted P value (P_{adj}).

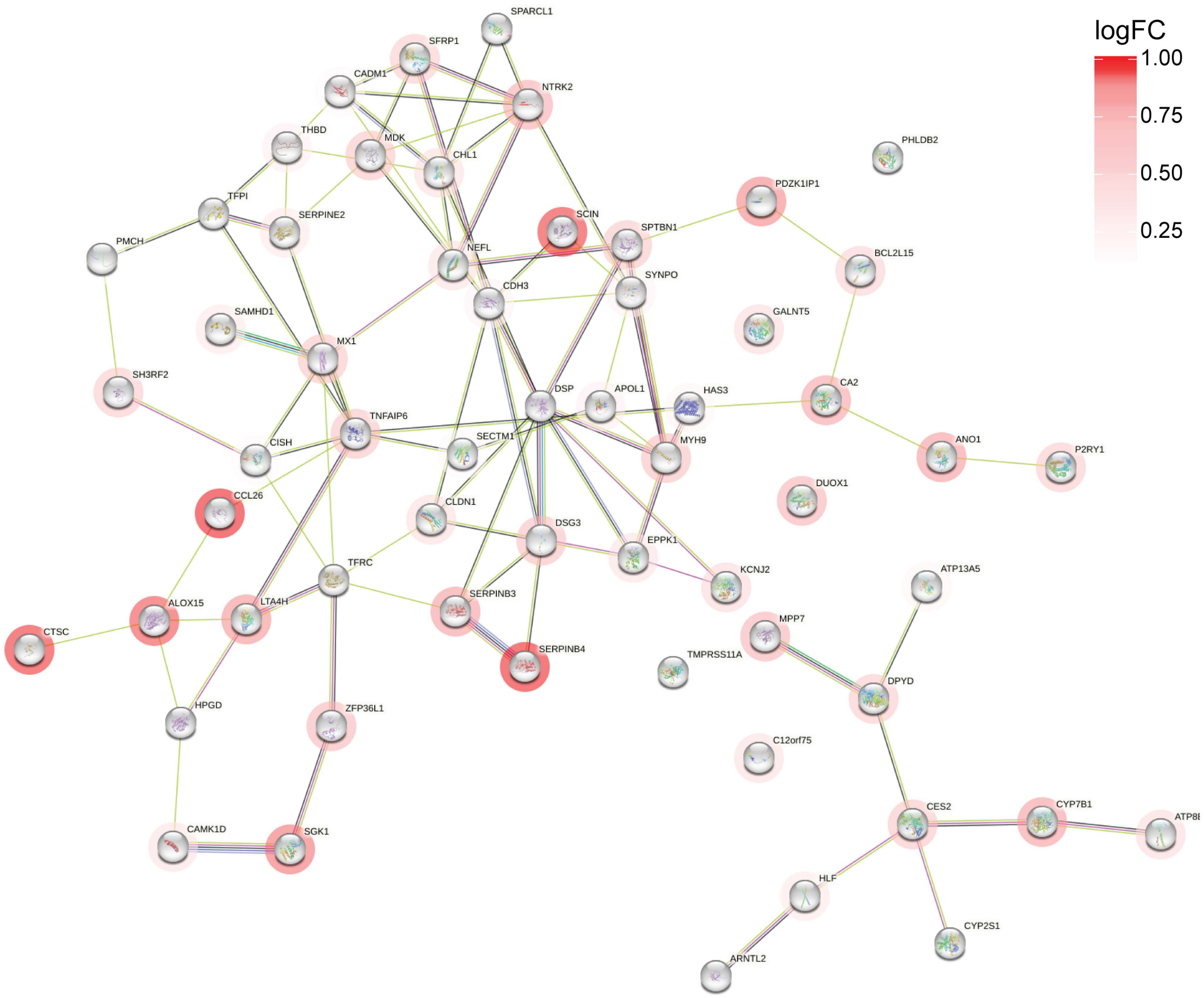

Supplemental Figure 8. Protein-protein interactions in EoE-specific trajectory-dependent genes. Known interactions are mapped between all module 7 gene products. Lines indicate known interactions, halo color intensity indicates logFC between differentiated cells in EoE versus HC.

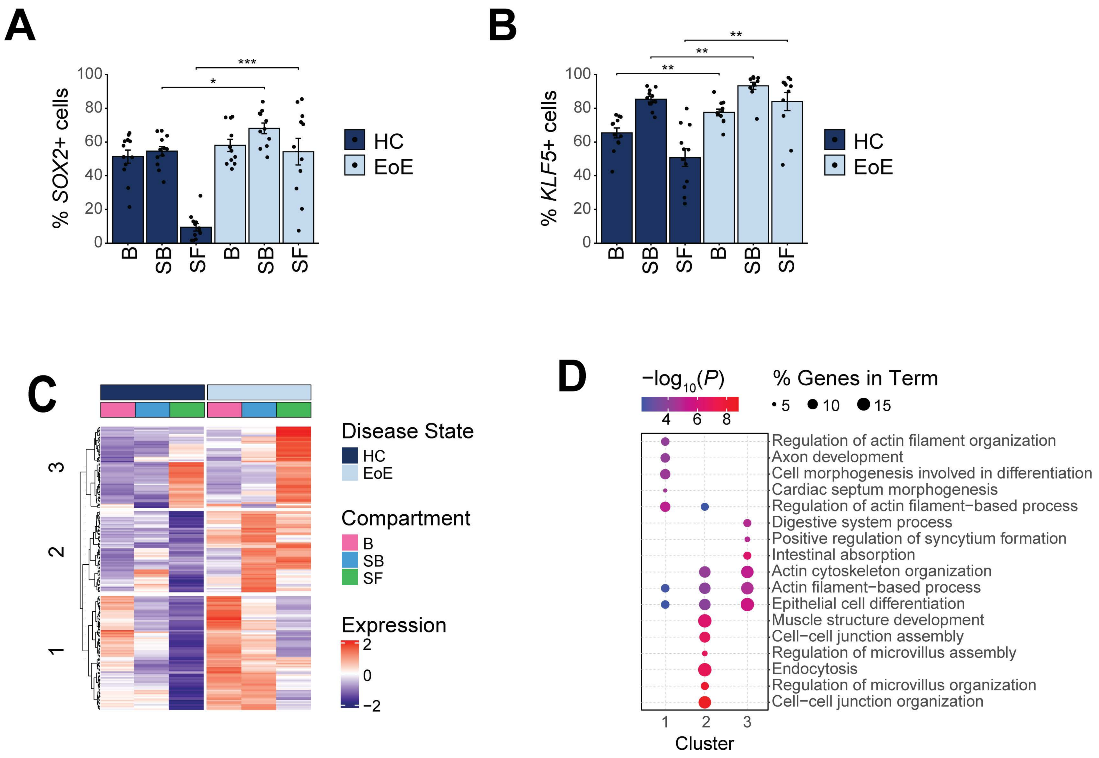

Supplemental Figure 9. SOX2, KLF5 and their transcriptional targets exhibit increased expression in the esophageal epithelial suprabasal and superficial compartment in EoE. (**A**) Bar plots showing the percentage of cells expressing meaningful levels of *SOX2* (**A**) or *KLF5* (**B**) in each epithelial compartment in either HC or EoE. Data are shown as mean ± SEM. Indicated *P* values were determined using Wilcoxon signed-ranked test with Benjamini & Hochberg adjustment for multiple comparisons. $^*P \leq 0.05$, $^{**}P \leq 0.01$, $^{***}P \leq 0.001$. (**C**) Heatmap illustrating the upregulated expression of SOX2-KLF5 co-regulated genes in EoE. Top hierarchal clusters are identified, and their associated enriched terms are each shown in (**D**). Color indicates -$\log_{10}$(*P* value), dot size indicates percentage of genes along each term. Basal (B), Suprabasal (SB), Superficial (SF).

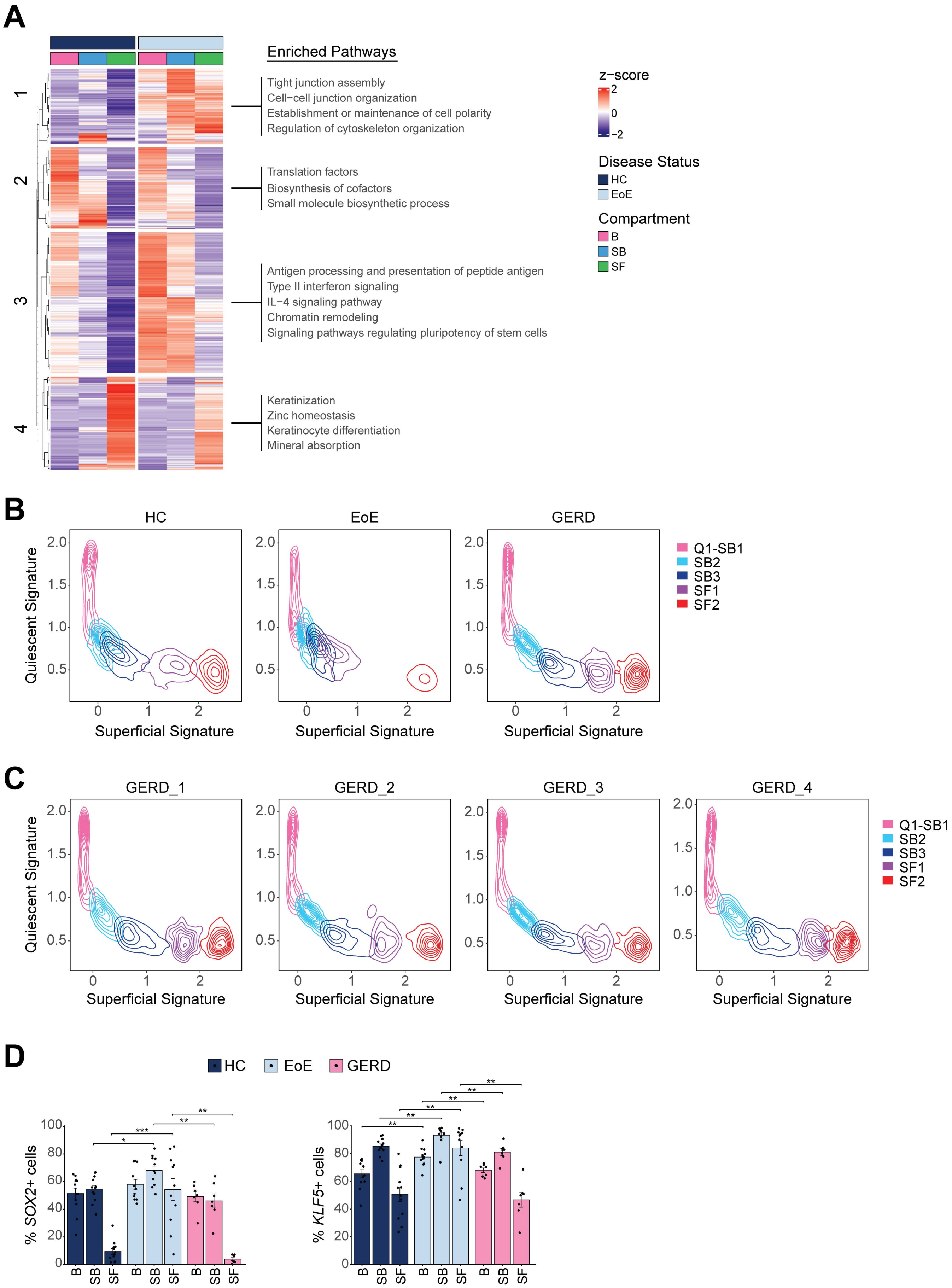

Supplemental Figure 10. Esophageal epithelial suprabasal and superficial compartments in GERD do not demonstrate increased basal-like identity or aberrant expression of basal transcriptional regulators. (**A**) Heatmap illustrating the expression of expression of DEGs calculated between EoE and HC across all EEC in the suprabasal and superficial compartments (|logFC| > 0.25, $P_{adj} < 0.05$). Top hierarchical clusters are identified, and their associated enriched terms are indicated. (**B**) Contour plots showing epithelial cells from HC, EoE or GERD patients plotted along the quiescent gene signature (y-axis) and the superficial gene signature (x-axis). Line color shows cell grouping by indicated epithelial clusters. Clusters are visualized per individual GERD patient in (**C**). (**D**) Bar plots showing the percentage of cells expressing meaningful levels of *SOX2* or *KLF5* in each epithelial compartment in either EoE, GERD or HC. Data are shown as mean ± SEM. Indicated *P* values were determined using Wilcoxon signed-ranked test with Benjamini & Hochberg adjustment for multiple comparisons. **P* ≤ 0.05, ***P* ≤ 0.01, ****P* ≤ 0.001. Basal (B), Quiescent (Q), Suprabasal (SB), Superficial (SF).

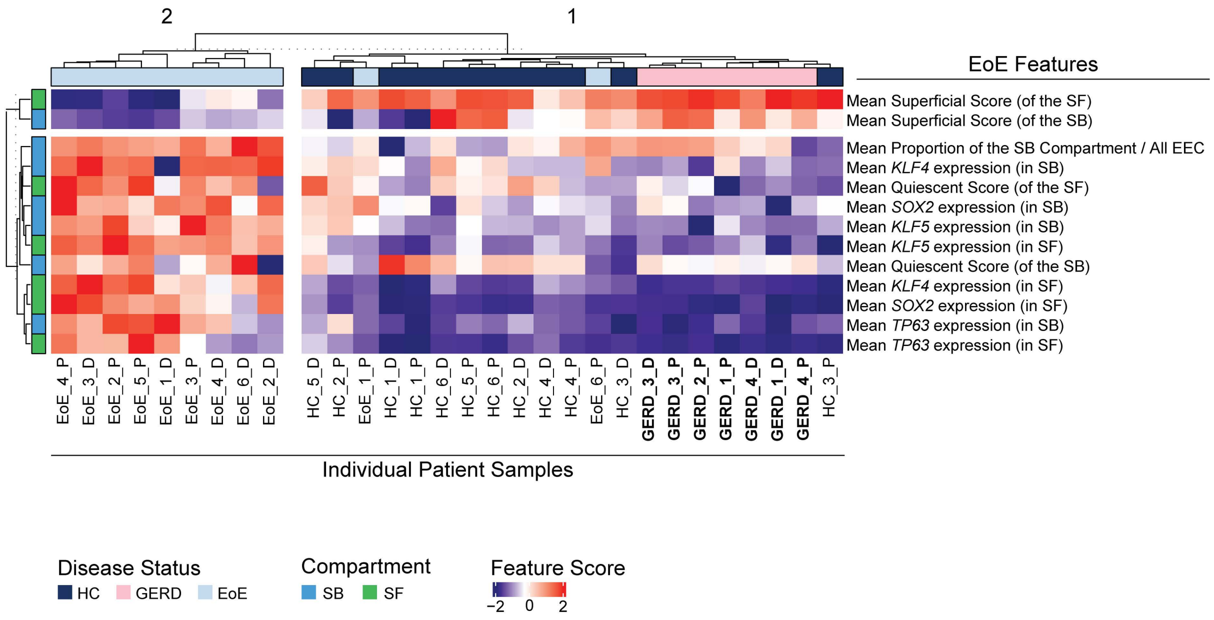

Supplemental Figure 11. Suprabasal and superficial epithelial cell compartments in GERD patients do not demonstrate an impaired basal-differentiation transition. Heatmap of identified EoE features calculated across suprabasal or superficial compartments for HC, EoE, or GERD patients, analyzed by hierarchical clustering. The two top-level hierarchical clusters are spatially separated for emphasis. Feature scoring is shown as row-normalized z-scores, top annotation indicates disease state, left annotation indicates epithelial cell compartment analyzed, columns represent individual patients. Suprabasal (SB), Superficial (SF).
