# Supplementary material for: Suprabasal cells retain progenitor cell identity programs in eosinophilic esophagitis–driven basal cell hyperplasia
Source: JCI Insight. 2023 Oct 9;8(19):e171765. doi: 10.1172/jci.insight.171765 (PMC10619442; doi:10.1172/jci.insight.171765)
Supplement: Supplemental tables 1-9 [file jciinsight-8-171765-s079.pdf]

Supplemental Tables

Clevenger, MH, *et al.* Suprabasal cells retain progenitor cell identity programs in eosinophilic esophagitis-driven basal cell hyperplasia

Table of Contents

Supplemental Table 1. Genes included in the quiescent gene signature. …………………...pg 2

Supplemental Table 2. Genes included in the superficial gene signature. …………………...pg 5

Supplemental Table 3. Genes corresponding to co-expressed modules along the EoE epithelial pseudotemporal trajectory……………………………………………………...…..…pg 8

Supplemental Table 4. Gene expression profile of module 7 genes in EoE vs healthy subjects. ……………………………………………………………………………………………….pg 22

Supplemental Table 5. Identification of SOX2 and/or KLF5 downstream targets from Enrichr databases and literature-based analyses. ……………………………………………………...pg 24

Supplemental Table 6. Module 7 genes known to be regulated by SOX2 and/or KLF5........pg 170

Supplemental Table 7. Gene expression profile of upregulated EoE DEGs known to be regulated by SOX2 and/or KLF5. ....…pg 171

Supplemental Table 8. Gene expression profile of EoE DEGs known to be regulated by the SOX2-KLF5 interaction..…pg 192

Supplemental Table 9. List of antibodies used in the study. ………………………………….pg 197

Supplemental Table 1. Genes included in the quiescent gene signature.

gene	p_val	avg_log2FC	pct.1	pct.2	p_val_adj
DST	0	2.829762397	0.937	0.211	0
KRT15	0	2.749389646	1	0.685	0
KRT19	0	2.027514439	0.99	0.662	0
GAS5	0	1.991027262	0.727	0.641	0
CXCL14	0	1.930840409	0.497	0.095	0
MIR205HG	0	1.681103048	0.728	0.686	0
TXNIP	0	1.679755517	0.787	0.396	0
IL1R2	0	1.641511831	0.619	0.063	0
IL18	0	1.616816891	0.9	0.729	0
ZFP36L2	0	1.608479362	0.823	0.526	0
IFITM3	0	1.606175204	0.858	0.54	0
GPNMB	0	1.602788604	0.741	0.217	0
CAV1	0	1.57258403	0.693	0.127	0
IGFBP5	0	1.564140033	0.586	0.14	0
GPX4	0	1.556344306	0.955	0.852	0
MT-ND2	0	1.541729919	0.95	0.884	0
COL17A1	0	1.504913141	0.68	0.1	0
MALAT1	0	1.497386787	0.975	0.92	0
TSLP	0	1.492823424	0.61	0.088	0
FOS	0	1.469149928	0.977	0.939	0
FTH1	0	1.451440638	0.972	0.929	0
SLC3A2	0	1.428585636	0.811	0.576	0
MT-ND1	0	1.42571156	0.953	0.891	0
MT-ND3	0	1.409695337	0.967	0.922	0
MT-CYB	0	1.374772617	0.979	0.933	0
ATF3	0	1.369038577	0.828	0.673	0
ATP1B3	0	1.315243129	0.871	0.741	0
MT-ATP6	0	1.308089875	0.969	0.922	0
SNHG8	0	1.296775833	0.926	0.798	0
FHL2	0	1.288653373	0.659	0.194	0
MT-CO3	0	1.262818148	0.977	0.944	0
RPL34	0	1.252450795	1	0.994	0
C12orf57	0	1.251320924	0.864	0.712	0
ZFAS1	0	1.239408574	0.965	0.911	0
CH25H	0	1.196781019	0.496	0.051	0
DLK2	0	1.177325313	0.524	0.032	0
NOP53	0	1.162744015	0.822	0.724	0
MT-ND5	0	1.162255222	0.889	0.81	0
SNHG7	0	1.141464301	0.756	0.639	0
ASS1	0	1.110706576	0.554	0.123	0
TSC22D1	0	1.091835855	0.546	0.305	0
NAP1L1	0	1.090727748	0.812	0.664	0
CD81	0	1.085738797	0.753	0.571	0

NPR3	0	1.085345049	0.547	0.171	0
CTNNAL1	0	1.0784772	0.52	0.054	0
THSD4	0	1.075858062	0.567	0.254	0
SNHG29	0	1.070984512	0.734	0.772	0
EEF1D	0	1.069898077	0.98	0.945	0
RPS27	0	1.058941417	1	0.994	0
WNT4	0	1.051182329	0.588	0.419	0
CIRBP	0	1.048393598	0.85	0.799	0
RACK1	0	1.044522976	1	0.99	0
MT-ND4	0	1.044313515	0.963	0.917	0
LAMB3	0	1.044175468	0.558	0.166	0
RPL10	0	1.04270105	1	0.998	0
RPL30	0	1.041514408	1	0.993	0
ITGB1	0	1.038297217	0.623	0.389	0
RPL5	0	1.027958819	1	0.992	0
LIMA1	0	1.019869097	0.605	0.379	0
EPB41L4A-AS1	0	1.015005509	0.646	0.384	0
RPS9	0	0.997767959	1	0.993	0
SLC1A3	0	0.989165554	0.48	0.093	0
RPL13	0	0.986841391	1	0.998	0
SLC7A1	0	0.985424125	0.624	0.378	0
RPS4X	0	0.977786324	1	0.997	0
SLC7A5	0	0.959832268	0.522	0.109	0
RPL12	0	0.954870183	1	0.996	0
APP	0	0.934912332	0.515	0.196	0
RPS3	0	0.933890118	1	0.996	0
EIF3E	0	0.929264383	0.951	0.911	0
ARPC1B	0	0.92852849	0.531	0.218	0
RPL35A	0	0.926635559	1	0.992	0
RPS5	0	0.917666457	1	0.991	0
MT-CO1	0	0.910015039	0.988	0.958	0
RPLP1	0	0.907581937	1	0.999	0
NPM1	0	0.900359763	0.99	0.957	0
RPL37	0	0.895612447	1	0.995	0
RPL10A	0	0.894123777	1	0.991	0
PDLIM1	0	0.892139816	0.832	0.788	0
MTRNR2L8	0	0.887226548	0.519	0.487	0
RPS19	0	0.884909432	1	0.997	0
JUNB	0	0.884094259	0.855	0.863	0
TP53AIP1	0	0.88224727	0.539	0.237	0
MT2A	0	0.876672684	0.81	0.535	0
TIMP1	0	0.87575402	0.462	0.104	0
RPS21	0	0.873160764	0.998	0.988	0
HNRNPA1	0	0.872899675	0.92	0.885	0
RPL3	0	0.869343675	1	0.996	0
PNRC1	0	0.852584474	0.736	0.703	0
RPS15A	0	0.84947814	1	0.992	0

MT-ND4L	0	0.849387461	0.61	0.566	0
RPL18A	0	0.845159597	1	0.996	0
BTF3	0	0.843251407	0.996	0.977	0
RPL11	0	0.841695911	1	0.997	0
KTN1	0	0.83970417	0.826	0.813	0
RPL19	0	0.839507184	1	0.996	0
POLR1D	0	0.839246533	0.838	0.823	0
MT-CO2	0	0.836466386	0.987	0.961	0
EGR1	0	0.835157992	0.808	0.764	0
RPL32	0	0.833150593	1	0.998	0

Supplemental Table 2. Genes included in the superficial gene signature.

gene	p_val	avg_log2FC	pct.1	pct.2	p_val_adj
CRCT1	0	6.663212326	0.998	0.407	0
CNFN	0	5.997242055	1	0.526	0
KRT78	0	5.298534143	0.994	0.27	0
SPRR2D	0	5.277971515	0.848	0.364	0
LYPD2	0	5.240212687	0.992	0.242	0
FAM25A	0	5.213556785	0.968	0.284	0
ECM1	0	5.109232069	0.998	0.372	0
MT1G	0	5.034032823	0.678	0.372	0
SPINK7	0	4.961649141	0.939	0.18	0
SPRR2A	0	4.847879757	0.952	0.567	0
TMPRSS11B	0	4.762628504	0.995	0.135	0
FTH1	0	4.539188087	0.999	0.952	0
MAL	0	4.438547514	1	0.571	0
HOPX	0	4.340963854	0.997	0.708	0
CRISP3	0	4.325306482	0.977	0.083	0
FLG	0	4.244267247	0.598	0.052	0
C15orf48	0	4.127274307	0.971	0.131	0
LCN2	0	4.051987717	0.973	0.343	0
ERO1A	0	4.012610375	0.981	0.314	0
SPRR2E	0	3.965579924	0.749	0.288	0
CRNN	0	3.951263614	0.999	0.386	0
EMP1	0	3.933290949	0.999	0.692	0
PSCA	0	3.913357265	0.941	0.127	0
S100P	0	3.784702209	0.879	0.116	0
MUC21	0	3.777149043	0.994	0.282	0
ADIRF	0	3.75946849	0.97	0.538	0
SPRR3	0	3.743637867	1	0.859	0
PHLDA1	0	3.551732291	0.941	0.11	0
PRSS27	0	3.295137849	0.983	0.179	0
SCEL	0	3.236097127	0.99	0.397	0
MXD1	0	3.197818467	0.97	0.203	0
TGM3	0	3.127514331	0.988	0.308	0
CYSRT1	0	3.101653855	0.981	0.343	0
TMPRSS11E	0	3.056300408	0.976	0.197	0
C4orf3	0	3.050301701	0.993	0.75	0
PPL	0	2.852724672	0.987	0.475	0
MT2A	0	2.842034342	0.718	0.718	0
MT1H	0	2.835812071	0.356	0.113	0
LMO7	0	2.834768659	0.954	0.297	0
AIF1L	0	2.790360446	0.942	0.117	0
SPRR1B	0	2.708646086	0.809	0.324	0
CSTB	0	2.708363743	1	0.949	0

SAMD9	0	2.691807079	0.944	0.317	0
SLURP2	0	2.682477446	0.855	0.289	0
NCCRP1	0	2.681409043	0.976	0.211	0
CAMK2N1	0	2.669605001	0.91	0.027	0
TTC9	0	2.669185763	0.948	0.196	0
EPS8L1	0	2.658087037	0.956	0.113	0
SPINK5	0	2.627968093	0.999	0.692	0
PPDPF	0	2.562754409	0.997	0.881	0
TIMP2	0	2.549920635	0.855	0.033	0
RNASE7	0	2.53043594	0.533	0.024	0
IL1RN	0	2.528853643	0.993	0.576	0
NIBAN2	0	2.474299436	0.818	0.354	0
DUSP5	0	2.450672188	0.845	0.114	0
SAT1	0	2.424408514	0.988	0.789	0
MT1M	0	2.422126718	0.292	0.094	0
S100A9	0	2.421642912	1	0.921	0
MYL12A	0	2.318396676	0.99	0.844	0
IL18	0	2.300159819	0.983	0.784	0
MUC1	0	2.283834402	0.792	0.071	0
TMOD3	0	2.271593495	0.974	0.598	0
C6orf132	0	2.250125477	0.957	0.427	0
FAM3D	0	2.238508466	0.9	0.195	0
ELF3	0	2.224858276	0.957	0.465	0
KRT17	0	2.208476981	0.553	0.312	0
SH3BGRL2	0	2.205070746	0.932	0.185	0
SPNS2	0	2.190006205	0.836	0.033	0
KRT16	0	2.188358007	0.754	0.32	0
A2ML1	0	2.17596011	0.973	0.392	0
ANXA11	0	2.172567648	0.972	0.567	0
MT1E	0	2.16294391	0.577	0.616	0
H2AFJ	0	2.10222954	0.976	0.626	0
GCHFR	0	2.049766	0.862	0.369	0
SFTA2	0	2.045810841	0.877	0.094	0
HILPDA	0	2.042207511	0.575	0.27	0
ADGRF1	0	1.991916337	0.82	0.122	0
ANXA1	0	1.954987483	1	0.969	0
MALL	0	1.94355763	0.973	0.472	0
SLURP1	0	1.937649171	0.918	0.313	0
RAB5IF	0	1.929411281	0.949	0.625	0
LGALS3	0	1.929328711	0.995	0.937	0
TAX1BP1	0	1.913210716	0.967	0.576	0
CLIC3	0	1.869309291	0.959	0.415	0
S100A8	0	1.85301537	1	0.885	0
PRSS3	0	1.828960014	0.862	0.093	0
CD59	0	1.819298232	0.967	0.624	0
CST6	0	1.818308314	0.878	0.119	0
GLUL	0	1.809863604	0.676	0.435	0

HPGD	0	1.807314504	0.912	0.383	0
KLK12	0	1.79835912	0.635	0.175	0
DHRS9	0	1.786528228	0.711	0.018	0
PLAC8	0	1.771809915	0.734	0.2	0
TMEM40	0	1.74106641	0.963	0.511	0
TPM4	0	1.723422329	0.969	0.664	0
SNCG	0	1.687185291	0.721	0.053	0
RIOK3	0	1.652755952	0.952	0.626	0
YOD1	0	1.652293029	0.693	0.078	0
SDCBP2	0	1.650459311	0.91	0.32	0
CD24	0	1.638709797	0.99	0.564	0

Supplemental Table 3. Genes corresponding to co-expressed modules along the EoE epithelial pseudotemporal trajectory.

gene	module	supermodule	dim_1	dim_2	status	p_value	morans_test_statistic	morans_I	q_value
AC020916.1	1	1	1.639280215	-2.488101603	OK	0	251.3780328	0.254997076	0
AC036176.3	1	1	1.684323863	-2.503947617	OK	0	126.1473819	0.127955766	0
ADH1C	1	1	0.319838539	-3.033913674	OK	0	106.8289895	0.108348199	0
ADM	1	1	0.863050229	-3.030575337	OK	0	143.3322682	0.145389691	0
APCDD1	1	1	0.538907961	-2.499797227	OK	0	158.4539216	0.16072909	0
APOBEC3A	1	1	2.508020416	-2.591249706	OK	0	144.5728216	0.146645358	0
AREG	1	1	2.392676488	-2.629367234	OK	0	257.2458014	0.260947948	0
ASS1	1	1	0.560633258	-2.920699002	OK	0	346.705388	0.35169657	0
BASP1	1	1	0.282518283	-3.296340169	OK	0	237.0933783	0.240497285	0
BCAM	1	1	0.468359694	-3.365264447	OK	0	257.5000892	0.261195281	0
BHLHE40	1	1	2.382114068	-2.643254162	OK	0	276.6513126	0.28063536	0
BMP2	1	1	0.852943898	-2.869146766	OK	0	105.7401336	0.10724814	0
BST2	1	1	-0.690088137	-2.770016672	OK	0	152.2795412	0.154464325	0
BTG2	1	1	1.574786977	-2.563254596	OK	0	277.813387	0.281814399	0
C1R	1	1	-0.585594281	-2.930390836	OK	0	251.4742279	0.25508269	0
CALML3	1	1	1.173150763	-1.425154926	OK	0	361.5015277	0.366709242	0
CASC19	1	1	0.111580805	-2.182737948	OK	0	142.600573	0.144642809	0
CAV1	1	1	0.09860243	-2.922177555	OK	0	514.6421679	0.522060707	0
CAVIN2	1	1	0.909654715	-2.925697805	OK	0	119.7009119	0.121415339	0
CCDC80	1	1	0.513993905	-2.903766514	OK	0	139.6095003	0.141598815	0
CCL20	1	1	1.002225228	-2.643324854	OK	9.37E-292	36.48564812	0.036994726	9.48E-292
CCL4	1	1	1.999881879	-4.156357737	OK	3.79E-152	26.2477909	0.026606686	3.81E-152
CCN1	1	1	0.77189231	-2.910440923	OK	0	197.8381453	0.200674161	0
CCND1	1	1	0.636073903	-1.770222904	OK	0	362.9119658	0.368141595	0
CCND2	1	1	0.367253617	-2.54338968	OK	0	234.2296091	0.23759432	0
CD55	1	1	2.35637571	-2.351353289	OK	0	370.4750918	0.375814658	0
CD74	1	1	-0.655046328	-2.785525443	OK	0	191.5775062	0.194331465	0
CDKN1A	1	1	2.458906785	-2.559730412	OK	0	264.6479424	0.268458584	0
CDKN2B	1	1	2.322521821	-2.245069148	OK	0	315.3491555	0.319890431	0
CFB	1	1	-0.672548517	-2.75234151	OK	0	142.8735756	0.144923465	0
CH25H	1	1	-0.303361758	-2.863647701	OK	0	243.1075023	0.246604479	0
CLDN4	1	1	2.610721961	-2.507062437	OK	0	486.9148983	0.493935886	0
CLEC2B	1	1	1.817905859	-4.081781508	OK	0	134.2228228	0.13614883	0
CLU	1	1	-0.615097388	-2.918249787	OK	0	115.1698537	0.116814647	0
COL17A1	1	1	0.254001633	-2.500339271	OK	0	547.7495959	0.555643717	0
CRABP2	1	1	1.807100311	-1.92574084	OK	0	365.6871792	0.370953409	0
CRIP1	1	1	1.011198164	-1.699302198	OK	0	152.03995	0.154224088	0
CRIP2	1	1	1.081225858	-1.483327152	OK	0	139.583941	0.14158715	0
CTNNAL1	1	1	0.117452518	-3.212987961	OK	0	523.8488433	0.531391276	0
CTSG	1	1	1.930303231	-4.230151118	OK	0	69.62924232	0.070601767	0
CXCL14	1	1	1.252691582	-1.564850332	OK	0	277.1710439	0.281144015	0
CXCL2	1	1	1.307683245	-1.749804498	OK	2.79E-184	28.92629484	0.02932487	2.81E-184
CYP2E1	1	1	-0.547377571	-2.791121842	OK	0	67.68390842	0.068647956	0
CYP3A5	1	1	1.863548533	-2.087296845	OK	0	103.0310223	0.104504573	0
DLK2	1	1	0.408009574	-3.390769394	OK	0	412.9989488	0.418934834	0
DST	1	1	0.461137876	-2.536338927	OK	0	568.3045922	0.576500484	0
DUSP2	1	1	1.954672948	-2.62598622	OK	0	51.81895849	0.052553487	0
EDN1	1	1	0.873765824	-3.031929316	OK	0	127.9897084	0.129814816	0
EFNB2	1	1	2.486477033	-2.501721026	OK	0	128.9859157	0.130835068	0
EGR3	1	1	1.452322618	-2.574064376	OK	0	84.38707069	0.08558792	0
EIF1AY	1	1	-0.087112948	-2.366085531	OK	0	70.75675467	0.07176476	0
EMP3	1	1	-0.209373459	-3.079365791	OK	0	256.9533955	0.260638811	0
EPHA2	1	1	2.656547204	-2.423860552	OK	0	246.0033154	0.249543809	0
FABP5	1	1	0.751234681	-2.021072628	OK	0	502.6097048	0.509847502	0
FKBP2	1	1	0.476845965	-3.516573073	OK	0	53.25941835	0.054015331	0
FLNA	1	1	-0.133041009	-2.099577548	OK	0	231.8223223	0.235158029	0
FST	1	1	0.414912657	-3.388290794	OK	0	125.6322119	0.127419657	0
FXYD5	1	1	0.292254106	-3.399874957	OK	0	96.55240375	0.097932855	0
G0S2	1	1	-0.157118663	-2.87777275	OK	0	140.1699029	0.142155865	0
GADD45A	1	1	1.241321043	-2.991256358	OK	0	196.1514741	0.198972735	0
GADD45B	1	1	2.258333341	-1.481868269	OK	0	105.6110451	0.107124251	0
GBP1	1	1	1.11546667	-2.944585206	OK	0	142.4951207	0.144538675	0
GBP4	1	1	-0.714406117	-2.427138926	OK	0	95.03652978	0.096395348	0
GCLC	1	1	0.342774705	-2.376273872	OK	0	203.325739	0.206248864	0
GEM	1	1	1.291326687	-1.651242735	OK	0	116.9290194	0.118554234	0
GLDC	1	1	-0.276514157	-2.633357288	OK	0	157.210424	0.15946526	0
GLUL	1	1	0.434214458	-2.757886411	OK	0	159.2299888	0.161517858	0
HBA2	1	1	2.493381993	-4.069203676	OK	0.000384831	3.363480976	0.003393754	0.000385056
HBB	1	1	2.377574102	-3.734611923	OK	5.27E-50	14.82207236	0.015011786	5.30E-50
HBEGF	1	1	2.164289252	-2.679343821	OK	0	153.9177792	0.156128672	0

HES1	1	1	1.357425228	-2.382722975	OK	0	201.3878748	0.204284314	0
HLA-DRA	1	1	-0.568672403	-2.835550191	OK	9.69E-265	34.7403494	0.035226997	9.80E-265
ID1	1	1	2.581723586	-2.617908122	OK	0	242.6811155	0.246174717	0
ID3	1	1	2.521021262	-2.492029907	OK	0	241.5798604	0.245057319	0
ID4	1	1	1.217843548	-1.420264246	OK	0	202.8877829	0.20580637	0
IFI27	1	1	-0.675456866	-2.200739743	OK	0	181.7270298	0.184339738	0
IFIT3	1	1	-0.703901156	-2.729019286	OK	0	74.26490012	0.075322566	0
IFITM1	1	1	-0.570020303	-2.7569654	OK	0	187.9036526	0.190596143	0
IFITM2	1	1	1.011107982	-1.782858373	OK	0	74.91433667	0.075982435	0
IGFBP3	1	1	0.371005431	-2.700244309	OK	0	306.9375209	0.311350376	0
IGFBP5	1	1	0.588681982	-2.719751121	OK	0	160.1798855	0.162478545	0
IGFBP6	1	1	0.787968048	-2.105188014	OK	0	262.7523086	0.266528557	0
IGFBP7	1	1	-0.346240743	-2.96817881	OK	0	352.2977457	0.357359273	0
IGHA1	1	1	2.557645098	-4.251122923	OK	1.41E-126	23.90349071	0.024215223	1.42E-126
IGKC	1	1	2.54377009	-4.25130731	OK	2.44E-46	14.24399628	0.014430413	2.45E-46
IGLC1	1	1	2.556192414	-4.246733756	OK	2.08E-45	14.09346196	0.014242158	2.09E-45
IGLC2	1	1	2.554935471	-4.255022736	OK	5.26E-49	14.66684552	0.014847509	5.28E-49
IGLC3	1	1	2.555681483	-4.232765646	OK	1.59E-16	8.165986867	0.008246619	1.60E-16
IL18	1	1	0.524979637	-3.263586702	OK	0	296.1337585	0.300398808	0
IL1R2	1	1	-0.234344944	-3.118945004	OK	0	545.0326173	0.552880492	0
IRF1	1	1	-0.604711636	-2.693165304	OK	0	170.8912911	0.173347244	0
ISG15	1	1	-0.662907823	-2.142244817	OK	0	53.05617638	0.053809125	0
ITGA6	1	1	0.254386679	-2.404686691	OK	0	303.19091	0.307556875	0
ITM2A	1	1	1.483513609	-1.334334852	OK	5.07E-44	13.86630512	0.014049758	5.08E-44
ITPKC	1	1	2.682129756	-2.470367552	OK	0	229.6816513	0.232987191	0
JCHAIN	1	1	2.560954109	-4.249203862	OK	3.61E-250	33.76159979	0.034170307	3.65E-250
KCNQ1OT1	1	1	1.962834135	-2.233455183	OK	0	90.04412084	0.09132927	0
KLF2	1	1	2.378366247	-1.980278374	OK	0	126.2714861	0.128081454	0
KLF6	1	1	1.352223173	-2.84309137	OK	0	265.1055937	0.268921095	0
KLK5	1	1	0.547773943	-3.337084921	OK	0	118.4480083	0.120138301	0
KRT14	1	1	1.203801558	-1.93390453	OK	0	517.0799878	0.524536923	0
KRT17	1	1	0.385406092	-2.944312097	OK	0	232.3568361	0.235701003	0
KRT31	1	1	0.948314369	-1.837954284	OK	0	56.15630649	0.056951543	0
KRT8	1	1	1.223441348	-1.860041501	OK	0	223.0145213	0.226223633	0
LAMB3	1	1	0.040065662	-2.469382884	OK	0	415.3611254	0.42134732	0
LDLR	1	1	2.208782212	-2.645735146	OK	0	155.3873989	0.157619287	0
LGALS1	1	1	1.380708233	-1.755956175	OK	2.14E-224	31.95732083	0.032402031	2.16E-224
LGALS7	1	1	1.090734795	-2.069419266	OK	0	126.3137809	0.128126543	0
LGALSL	1	1	2.319630281	-2.184257628	OK	0	335.035367	0.339862205	0
LOXL4	1	1	-0.196646556	-2.750904919	OK	0	248.8545188	0.252411234	0
LSP1	1	1	1.118721039	-1.639778854	OK	8.94E-25	10.21000348	0.01034101	8.96E-25
LTF	1	1	-0.588402733	-2.765226962	OK	0	87.20869164	0.088434068	0
MAFF	1	1	2.511035935	-2.526869656	OK	0	211.3821363	0.21442287	0
MALAT1	1	1	1.566699818	-2.224784733	OK	0	274.782913	0.278732686	0
MAP3K8	1	1	2.390411869	-2.413751961	OK	0	135.6530637	0.137598662	0
MIDN	1	1	2.452559129	-2.541659714	OK	0	233.2363591	0.236593418	0
MT-ND4L	1	1	1.018873767	-2.333582045	OK	0	173.4250491	0.17591813	0
MTRNR2L8	1	1	0.833866731	-2.226135613	OK	0	48.66179031	0.04935217	0
MYADM	1	1	1.113583491	-2.805315138	OK	0	144.0347554	0.146098441	0
MYO1B	1	1	0.028748051	-2.444175841	OK	0	138.4852347	0.140469925	0
NCOA7	1	1	1.155426995	-2.834561767	OK	0	337.5638755	0.342427952	0
NEAT1	1	1	1.83694394	-2.380966427	OK	0	267.4669646	0.271313838	0
NEDD9	1	1	0.333454684	-2.761224748	OK	0	193.8402198	0.196605034	0
NIBAN1	1	1	-0.134676083	-2.076206686	OK	0	123.085989	0.12485015	0
NPPC	1	1	1.228182689	-1.538484813	OK	0	114.497038	0.11605357	0
NR4A1	1	1	1.543684438	-2.590475203	OK	0	169.8232228	0.172263505	0
NR4A2	1	1	1.54458274	-2.543335916	OK	4.35E-307	37.44012295	0.037966936	4.41E-307
NRARP	1	1	2.601736442	-2.467336895	OK	0	394.009364	0.399689073	0
NSUN6	1	1	1.329912082	-2.377381088	OK	0	49.45029147	0.050149525	0
OVOL1	1	1	2.476075903	-2.590224864	OK	0	188.088242	0.190792516	0
PDPN	1	1	-0.196337208	-2.996297182	OK	0	311.1397947	0.31560801	0
PEG3	1	1	0.799546667	-2.360571028	OK	9.24E-221	31.69454113	0.032130556	9.32E-221
PER3	1	1	0.675755561	-1.963496448	OK	0	132.3432152	0.134241907	0
PHGDH	1	1	0.522620664	-3.547712685	OK	0	233.1862418	0.23653939	0
PHLDA2	1	1	2.583079115	-2.493777754	OK	0	345.8296751	0.350813315	0
PLAUR	1	1	2.397017375	-2.617506148	OK	0	90.32190452	0.091612232	0
PLK2	1	1	1.187501684	-2.941412152	OK	0	172.1827367	0.174657582	0
PLK3	1	1	1.848076061	-2.375512125	OK	0	89.0196517	0.090292174	0
POSTN	1	1	-0.368769034	-2.501061918	OK	0	232.6168156	0.235962398	0
PRRX1	1	1	-0.340539917	-2.662184478	OK	0	223.6743965	0.226881018	0
PRSS23	1	1	-0.110737189	-2.750689747	OK	0	184.9629801	0.187602854	0
RAB11FIP1	1	1	2.403470889	-2.333914878	OK	0	462.1266184	0.468790566	0
RAB31	1	1	-0.025321587	-2.504320861	OK	0	94.22632269	0.095572087	0

RAMP1	1	1	0.188344732	-3.315486015	OK	0	317.2659924	0.321820029	0
RHOB	1	1	2.242625371	-1.449702264	OK	0	202.4356987	0.205347422	0
RPS4Y1	1	1	-0.055976137	-2.476873638	OK	0	186.392789	0.189073109	0
RRAD	1	1	2.504522458	-2.524190785	OK	0	279.0862894	0.283105414	0
S100A9	1	1	-0.452807649	-2.634343626	OK	0	389.1506354	0.394745634	0
SAA1	1	1	-0.542092308	-2.953328671	OK	0	186.5676271	0.189238399	0
SERPINB2	1	1	2.394515411	-2.290807606	OK	0	359.4517273	0.364632018	0
SLC1A3	1	1	0.518800721	-2.612765075	OK	0	347.6272537	0.352624721	0
SLC25A25	1	1	2.35760893	-2.665043356	OK	0	123.5270162	0.125297153	0
SLC7A2	1	1	0.472764239	-2.380517484	OK	0	111.0431031	0.112626557	0
SLC7A5	1	1	0.55456169	-2.591320039	OK	0	357.6538348	0.362800445	0
SNHG17	1	1	0.759468571	-2.21122575	OK	0	74.24134721	0.075300069	0
SOCS3	1	1	-0.331208214	-2.883567931	OK	0	140.3157356	0.142328816	0
SOD2	1	1	-0.593009814	-2.747240783	OK	0	188.8917606	0.191607998	0
SOX4	1	1	0.829217896	-2.552506448	OK	0	98.85032033	0.100265606	0
SOX9	1	1	2.555351988	-2.578988077	OK	0	98.2141115	0.099619063	0
SRGN	1	1	1.931935207	-4.225006135	OK	0	48.95780565	0.049640921	0
SYNPO2	1	1	-0.113116547	-2.643561007	OK	0	250.203614	0.253787951	0
SYT8	1	1	1.029980273	-1.718196632	OK	0	81.57041209	0.082734243	0
TIMP1	1	1	-0.365753516	-2.628055813	OK	0	347.6863231	0.352696174	0
TINAGL1	1	1	-0.211673602	-2.832720877	OK	0	168.6046847	0.171013468	0
TNC	1	1	-0.151409372	-2.705799343	OK	0	284.1738482	0.288246259	0
TNFAIP2	1	1	-0.658591493	-2.533924343	OK	0	197.2090193	0.200043031	0
TNFAIP3	1	1	1.433981672	-2.69384432	OK	0	50.76613644	0.051485738	0
TNFRSF12A	1	1	0.076904849	-2.818001153	OK	0	126.3962489	0.12820449	0
TPM2	1	1	0.403067157	-3.443870069	OK	0	192.4588265	0.195212845	0
TPPP3	1	1	1.007442192	-1.700606348	OK	0	89.45077119	0.090727134	0
TPSAB1	1	1	1.956884161	-4.246059717	OK	0	105.468717	0.106962053	0
TPSB2	1	1	1.921123997	-4.242013218	OK	0	113.88851	0.115504233	0
TRIB1	1	1	1.476699785	-2.838272633	OK	0	77.35067692	0.078454578	0
TSLP	1	1	0.266598836	-3.335943909	OK	0	368.0925964	0.373390112	0
TUBB2A	1	1	2.51715614	-2.008536817	OK	0	217.7546552	0.220887095	0
TXNIP	1	1	0.082129554	-2.866490068	OK	0	247.3527351	0.25091322	0
UGCG	1	1	-0.69958578	-2.452220918	OK	0	134.2644889	0.136191135	0
VIM	1	1	1.897630469	-4.184362801	OK	8.36E-207	30.66491519	0.031090937	8.43E-207
WNT10A	1	1	0.45750247	-3.267883422	OK	0	389.7178211	0.39532058	0
XIST	1	1	1.056280569	-2.099429132	OK	0	71.0457907	0.072058953	0
ZNF750	1	1	2.368177668	-2.423241021	OK	0	440.6977967	0.447051918	0
ASF1B	2	2	-4.339882597	-6.223984481	OK	0	303.6197256	0.307970249	0
ATAD2	2	2	-4.257139429	-5.882794859	OK	0	332.4975271	0.337284161	0
ATAD5	2	2	-4.159812673	-5.814746739	OK	0	294.7481882	0.298979108	0
BRCA1	2	2	-4.02936004	-5.897448064	OK	0	324.5089025	0.329167832	0
BRCA2	2	2	-4.240623697	-5.727677585	OK	0	307.555001	0.311968537	0
CARHSP1	2	2	-4.483340009	-6.193916322	OK	0	270.5745973	0.274470824	0
CCDC34	2	2	-5.148061498	-6.522906543	OK	0	265.4406665	0.269261787	0
CDCA5	2	2	-4.89060901	-5.893674852	OK	0	322.045084	0.326657235	0
CDCA7	2	2	-3.511877283	-6.095230581	OK	0	291.3183535	0.29550243	0
CDT1	2	2	-3.491524442	-6.169977667	OK	0	302.2505479	0.306593012	0
CENPK	2	2	-4.805449709	-6.448268177	OK	0	367.7205942	0.373007197	0
CENPM	2	2	-4.802062734	-6.448778869	OK	0	383.4095938	0.388921592	0
CENPU	2	2	-4.124042734	-6.1760664	OK	0	248.5664698	0.252141656	0
CEP78	2	2	-4.318910345	-5.683482887	OK	0	183.1694548	0.185795037	0
CHEK1	2	2	-3.690222486	-6.049997331	OK	0	290.5412327	0.294715062	0
CLSPN	2	2	-4.092672094	-6.093418361	OK	0	389.6023096	0.395192926	0
DEK	2	2	-3.670383676	-6.027088405	OK	0	361.7305588	0.36694275	0
DHFR	2	2	-4.31337808	-6.14951873	OK	0	336.6264085	0.341467552	0
DNMT1	2	2	-3.743537172	-5.96856928	OK	0	320.6282158	0.325245044	0
DUT	2	2	-3.766904577	-6.135896207	OK	0	312.5193279	0.317021209	0
E2F8	2	2	-4.827411875	-5.795890214	OK	0	224.0968609	0.227315581	0
ESCO2	2	2	-4.901314958	-5.854121448	OK	0	417.5632557	0.423550427	0
EZH2	2	2	-4.688194021	-5.789565684	OK	0	212.581927	0.21563699	0
FAM111B	2	2	-4.009516939	-6.045913459	OK	0	420.2372258	0.426279261	0
FANCI	2	2	-4.567585214	-5.804541232	OK	0	306.0426272	0.310436999	0
FBXO5	2	2	-4.943127378	-5.854895832	OK	0	294.1621827	0.298379331	0
FEN1	2	2	-3.990973696	-6.190478088	OK	0	301.9577638	0.306296898	0
GINS2	2	2	-3.501417383	-6.136640312	OK	0	349.9647539	0.354994236	0
GMNN	2	2	-3.658759817	-6.060009958	OK	0	260.1691145	0.263911436	0
HELLS	2	2	-3.728663191	-6.020137788	OK	0	414.2059156	0.420169044	0
HES4	2	2	-4.158427461	-6.237429859	OK	0	260.8087447	0.264562814	0
HIST1H1A	2	2	0.114293293	-8.624274255	OK	0	371.44293	0.376778034	0
HIST1H1B	2	2	0.127776817	-8.609434129	OK	0	447.9944124	0.454435521	0
HIST1H1C	2	2	0.10637565	-8.633271696	OK	0	179.4587549	0.182038482	0
HIST1H1D	2	2	0.135121599	-8.668076994	OK	0	341.452203	0.346366147	0

HIST1H1E	2	2	0.110581831	-8.627090456	OK	0	229.9497091	0.233257254	0
HIST1H2AC	2	2	0.187880472	-8.516907694	OK	0	177.2708799	0.17981758	0
HIST1H2AG	2	2	0.108157054	-8.632150652	OK	0	308.5419311	0.312967688	0
HIST1H2BC	2	2	0.111142234	-8.626455785	OK	0	183.31419	0.18594093	0
HIST1H3B	2	2	0.123162225	-8.601028444	OK	0	388.4850417	0.394043177	0
HIST1H3D	2	2	0.096465961	-8.64129305	OK	0	281.4909056	0.285528032	0
HIST1H3G	2	2	0.083851353	-8.65212536	OK	0	343.3052112	0.348208081	0
HIST1H4C	2	2	0.077952698	-8.66427374	OK	0	330.5816847	0.335344011	0
HIST2H2AC	2	2	0.103010551	-8.651292326	OK	0	184.0538168	0.186696979	0
HSPH1	2	2	-3.67821525	-5.328660609	OK	0	186.3407804	0.189020214	0
LIG1	2	2	-4.11584638	-6.018011333	OK	0	237.5323514	0.240941803	0
LMNB1	2	2	-4.492936357	-5.562939645	OK	0	393.3574489	0.399018086	0
MCM3	2	2	-3.465010389	-6.071926119	OK	0	309.7711593	0.314229662	0
MCM4	2	2	-3.668562158	-5.677072407	OK	0	240.3590917	0.243816915	0
MCM5	2	2	-3.444336637	-6.189079525	OK	0	287.4815364	0.29161554	0
MCM6	2	2	-3.474766954	-6.032500984	OK	0	280.9989135	0.285035061	0
MCM7	2	2	-3.525834307	-6.165759803	OK	0	354.8152696	0.35992284	0
MELK	2	2	-5.142827257	-5.781885864	OK	0	259.7973015	0.263522647	0
MND1	2	2	-4.778887972	-6.44415784	OK	0	321.7540889	0.32636724	0
MNS1	2	2	-4.75841163	-6.368891002	OK	0	303.8454906	0.308196247	0
MSH6	2	2	-3.559077009	-5.851916076	OK	0	223.4767841	0.226689011	0
MYBL2	2	2	-4.730783685	-6.089763166	OK	0	330.5514179	0.335288675	0
NASP	2	2	-3.600200876	-6.051672699	OK	0	322.7272722	0.327376761	0
NCAPD3	2	2	-4.551370367	-5.688102485	OK	0	246.7831849	0.25032704	0
NCAPG2	2	2	-4.677098974	-5.685850503	OK	0	256.4721136	0.260147903	0
ORC6	2	2	-4.097230657	-6.138351919	OK	0	363.8643327	0.369093654	0
PCLAF	2	2	-4.957095369	-6.527775289	OK	0	448.9176111	0.455385143	0
PCNA	2	2	-3.632495149	-6.166114093	OK	0	320.7812437	0.325401937	0
PKMYT1	2	2	-4.581706747	-6.203773023	OK	0	315.0700891	0.319583693	0
PPIF	2	2	-3.486112818	-6.156327726	OK	0	120.3496948	0.122074771	0
PSIP1	2	2	-3.757222875	-6.017235519	OK	0	316.7354083	0.321295216	0
RAD51AP1	2	2	-4.81257604	-6.327401163	OK	0	345.7551801	0.350715451	0
RECQL	2	2	-3.741874918	-5.677526476	OK	0	128.7470146	0.130592743	0
RFC3	2	2	-3.862555727	-5.996248724	OK	0	202.5201102	0.205418977	0
RIF1	2	2	-3.734000429	-5.479385258	OK	0	203.6023775	0.206530179	0
RRM1	2	2	-4.059862837	-5.817682268	OK	0	281.6875114	0.285742545	0
RRM2	2	2	-4.812541708	-6.0115335	OK	0	477.6199408	0.484494517	0
SLC20A1	2	2	-4.178829893	-5.669030072	OK	0	113.0806074	0.114698065	0
SMC1A	2	2	-3.785370096	-5.439705254	OK	0	208.891864	0.211896333	0
SMC2	2	2	-4.432421907	-5.700499059	OK	0	351.0626718	0.356119374	0
TCOF1	2	2	-3.82350181	-5.511555077	OK	0	197.243147	0.200074937	0
TK1	2	2	-4.888381704	-6.520418407	OK	0	432.2123114	0.438437922	0
TMEM106C	2	2	-4.825308069	-6.505574228	OK	0	220.6734587	0.223848049	0
TMPO	2	2	-4.541800245	-5.503974678	OK	0	294.0909353	0.298326686	0
TYMS	2	2	-4.602869734	-6.357121946	OK	0	466.8802178	0.473601802	0
UBE2T	2	2	-5.241653665	-6.156429769	OK	0	350.8187575	0.355865515	0
WDR76	2	2	-3.752479299	-5.981121542	OK	0	237.6101167	0.241014802	0
ZWINT	2	2	-4.903456434	-6.489760877	OK	0	347.7647229	0.352767629	0
ANLN	3	2	-6.648049578	-5.714152695	OK	0	475.0182829	0.481849672	0
ANP32E	3	2	-6.73941873	-6.172750475	OK	0	250.3710827	0.253974298	0
ARHGAP11A	3	2	-6.777717813	-5.838612081	OK	0	324.5657833	0.329220512	0
ARL6IP1	3	2	-6.589393839	-6.875190975	OK	0	225.2103818	0.228451188	0
ASPM	3	2	-7.028236135	-6.294601919	OK	0	518.3509325	0.525807504	0
AURKA	3	2	-7.268601641	-5.791764261	OK	0	334.7481773	0.339551524	0
AURKB	3	2	-6.967200979	-5.388160946	OK	0	402.6692237	0.408447032	0
BIRC5	3	2	-6.51559852	-6.716683151	OK	0	477.3368896	0.484209069	0
BUB1	3	2	-7.06103681	-6.225082876	OK	0	380.6895813	0.386139402	0
BUB1B	3	2	-6.433765634	-5.488328458	OK	0	354.5448231	0.359622181	0
CCNA2	3	2	-6.798074468	-5.643153073	OK	0	488.430511	0.495452876	0
CCNB1	3	2	-6.612406477	-6.834413053	OK	0	419.4382514	0.425475142	0
CCNB2	3	2	-6.590443357	-6.824827911	OK	0	427.368051	0.433512858	0
CDC20	3	2	-6.623996958	-6.819634916	OK	0	440.6831851	0.447020122	0
CDCA3	3	2	-7.240159258	-5.748948337	OK	0	379.5626451	0.385006021	0
CDCA8	3	2	-7.095297083	-5.58951986	OK	0	398.6056673	0.404322532	0
CDK1	3	2	-6.301931127	-5.387701513	OK	0	456.769362	0.463341824	0
CDKN3	3	2	-6.58290027	-6.838282587	OK	0	449.2620406	0.455724218	0
CENPA	3	2	-7.288852915	-6.132091047	OK	0	428.3067258	0.434441798	0
CENPE	3	2	-7.096781	-6.299931528	OK	0	450.2198086	0.456690091	0
CENPF	3	2	-6.81276296	-6.486492159	OK	0	508.4613173	0.515783687	0
CENPN	3	2	-6.086987718	-6.710673095	OK	0	265.6445725	0.269459926	0
CENPW	3	2	-6.137951597	-6.794540645	OK	0	410.6286908	0.416545617	0
CEP55	3	2	-6.93303512	-6.358020784	OK	0	488.8382505	0.495862664	0
CKAP2	3	2	-7.071952089	-5.882383348	OK	0	273.5440429	0.277480308	0

CKAP2L	3	2	-6.44093202	-5.345185162	OK	0	394.9006833	0.400563152	0
CKAP5	3	2	-6.926187261	-6.093642236	OK	0	173.8331852	0.176327414	0
CKS1B	3	2	-5.841136678	-6.277043344	OK	0	315.6493946	0.320196158	0
CKS2	3	2	-7.375025018	-6.045836212	OK	0	265.0567637	0.268872725	0
DBF4	3	2	-6.547476515	-5.658339383	OK	0	220.4418124	0.223608834	0
DDX39A	3	2	-6.257055506	-6.820527794	OK	0	233.5166373	0.236876163	0
DEPDC1	3	2	-7.024569258	-5.820917846	OK	0	429.0195123	0.435177035	0
DIAPH3	3	2	-6.103458627	-5.513811351	OK	0	383.3640675	0.388864589	0
DLGAP5	3	2	-7.136504396	-6.334923031	OK	0	509.9364777	0.517260648	0
DTYMK	3	2	-6.040547117	-6.675614597	OK	0	288.3997887	0.292551862	0
ECT2	3	2	-6.868090376	-6.169924022	OK	0	296.345316	0.300606054	0
FAM83D	3	2	-7.374053224	-5.938319446	OK	0	178.0695124	0.180627389	0
FOXM1	3	2	-6.520667776	-5.68304825	OK	0	270.2818502	0.274153927	0
GTSE1	3	2	-7.221380457	-5.758037092	OK	0	459.0597043	0.46564352	0
H2AFX	3	2	-6.286169275	-5.266152383	OK	0	263.2412019	0.267029414	0
H2AFZ	3	2	-6.008220419	-6.787251474	OK	0	338.9789411	0.343861623	0
HJURP	3	2	-6.466233	-5.389489652	OK	0	334.6720879	0.339459107	0
HMGB2	3	2	-5.926471456	-5.909514429	OK	0	345.8882459	0.350872337	0
HMGB3	3	2	-6.373661264	-6.787762882	OK	0	237.7025782	0.241122218	0
HMMR	3	2	-7.171529039	-6.263320924	OK	0	498.1264527	0.505282954	0
KIF11	3	2	-6.3515179	-5.559972407	OK	0	381.813916	0.387305332	0
KIF20B	3	2	-6.944953188	-6.160136463	OK	0	377.0758759	0.382501862	0
KIF23	3	2	-7.137694582	-5.703711869	OK	0	443.0448743	0.449400458	0
KIF2C	3	2	-6.999280676	-5.816839697	OK	0	400.3925918	0.406130444	0
KIFC1	3	2	-6.399281248	-5.354016544	OK	0	356.6972792	0.361812066	0
KNL1	3	2	-6.758521303	-5.885560514	OK	0	424.8288366	0.430925865	0
KNSTRN	3	2	-7.357202753	-6.137953998	OK	0	257.8392919	0.261533794	0
KPNA2	3	2	-7.380627855	-6.06981969	OK	0	318.2757918	0.322855713	0
LMNB2	3	2	-6.621737703	-5.829898836	OK	0	244.4724342	0.247986341	0
MAD2L1	3	2	-5.760776743	-6.289104225	OK	0	414.4505642	0.420416252	0
MKI67	3	2	-6.593795522	-5.804621221	OK	0	526.9579538	0.53454879	0
MXD3	3	2	-7.073845609	-5.519011857	OK	0	290.628833	0.294788936	0
NCAPD2	3	2	-6.687580332	-6.022724153	OK	0	245.3189966	0.248841529	0
NCAPG	3	2	-6.390696272	-5.46391225	OK	0	408.6318355	0.414496461	0
NDC80	3	2	-6.377324327	-5.320534469	OK	0	404.7600497	0.410566063	0
NUF2	3	2	-6.949828848	-5.769080998	OK	0	462.6247398	0.469268259	0
NUSAP1	3	2	-6.496127828	-5.388550283	OK	0	516.8984844	0.524342178	0
PBK	3	2	-6.360498651	-5.340116145	OK	0	478.6983939	0.485577757	0
PHF19	3	2	-6.142628893	-6.6771369	OK	0	241.2201096	0.244688147	0
PLK1	3	2	-7.373345598	-6.021597387	OK	0	415.7317079	0.421690451	0
PRC1	3	2	-6.78877376	-5.697126629	OK	0	505.0432295	0.512314444	0
PRR11	3	2	-7.028197511	-6.392035963	OK	0	317.0958097	0.321633075	0
PTTG1	3	2	-6.300870642	-6.882250311	OK	0	389.5862866	0.395201744	0
RACGAP1	3	2	-6.878368601	-5.820924999	OK	0	265.8963449	0.269714191	0
SAP30	3	2	-6.628558382	-5.382044079	OK	0	197.6123397	0.20045162	0
SGO1	3	2	-6.403075441	-5.543348433	OK	0	382.9713219	0.38846642	0
SGO2	3	2	-7.080720171	-5.92029071	OK	0	380.4967547	0.385960121	0
SMC4	3	2	-6.675787672	-5.87640238	OK	0	306.5606786	0.31097631	0
SPC25	3	2	-6.340055212	-5.341148497	OK	0	383.6302199	0.389128815	0
SPDL1	3	2	-6.872765764	-5.842988969	OK	0	162.0347877	0.164354662	0
STMN1	3	2	-5.824393019	-6.720142843	OK	0	471.9225507	0.478726733	0
TACC3	3	2	-6.438571199	-5.421845438	OK	0	372.5025199	0.377849588	0
TOP2A	3	2	-6.928583368	-5.701792719	OK	0	513.486736	0.520879003	0
TPX2	3	2	-6.835941061	-5.7866807	OK	0	506.3080196	0.513596154	0
TROAP	3	2	-6.525570616	-6.871572258	OK	0	288.1826309	0.292315512	0
TTK	3	2	-6.795947775	-5.780751468	OK	0	396.9420042	0.402639219	0
TUBA1B	3	2	-5.956985697	-6.755624057	OK	0	321.286051	0.325913918	0
TUBB	3	2	-5.638810381	-6.600142481	OK	0	298.4346713	0.302733052	0
UBE2C	3	2	-7.237405046	-5.704974176	OK	0	441.0608542	0.447406164	0
UBE2S	3	2	-7.350721582	-5.938884498	OK	0	200.7814907	0.203669475	0
A2ML1	4	1	1.891713277	-0.120636465	OK	0	578.0125791	0.586349872	0
A4GALT	4	1	0.016242102	0.500440596	OK	0	285.3128117	0.289413993	0
AC005532.1	4	1	2.78548576	1.009047983	OK	0	280.409881	0.284418872	0
AC008397.1	4	1	0.830541004	1.249374388	OK	0	143.2849463	0.145339239	0
AC068587.4	4	1	0.675012976	1.432837008	OK	0	196.7178579	0.199541358	0
ACPP	4	1	2.342656032	0.278525828	OK	0	199.6201526	0.202486993	0
AGR2	4	1	2.474897281	1.620139597	OK	8.10E-113	22.54169602	0.022852451	8.14E-113
AIFM2	4	1	2.378798023	2.13858795	OK	0	100.4310324	0.101864991	0
AKR1B10	4	1	2.818512574	-0.774807216	OK	0	225.3844686	0.228625234	0
AKR1C1	4	1	2.833771483	-0.828878404	OK	0	84.04776588	0.085246431	0
AKR1C2	4	1	2.859662071	-0.8199389	OK	0	107.320345	0.10885511	0
AKR1C3	4	1	2.872497455	-0.799954893	OK	0	115.1200906	0.116766073	0
AL355312.4	4	1	2.920897022	2.806670187	OK	0	164.3823196	0.166712337	0

ALDH1A1	4	1	1.694270984	-0.671384813	OK	0	166.3984131	0.168780078	0
ALDH3B2	4	1	0.922371399	0.048130034	OK	0	343.2903912	0.348234824	0
ALOX12	4	1	2.88248469	1.174239634	OK	0	235.8135734	0.239172918	0
ALOX15B	4	1	2.391708032	-0.474366905	OK	0	68.19855221	0.069166303	0
ANKRD35	4	1	2.310582772	-0.383000614	OK	0	145.9227264	0.148013409	0
ANXA1	4	1	2.045872585	1.193990706	OK	0	250.8113796	0.254392244	0
ANXA3	4	1	0.921872147	0.816580771	OK	0	380.7749084	0.386262547	0
ASPG	4	1	1.754521981	1.836862562	OK	0	436.7374864	0.443032521	0
ATP13A4	4	1	0.249626294	0.977228163	OK	0	259.4040441	0.263128117	0
ATP1B1	4	1	1.334709123	0.925743578	OK	0	286.7203277	0.290850006	0
BEX4	4	1	2.877670423	1.555563448	OK	0	131.258638	0.133140826	0
BICDL2	4	1	1.032992796	1.797529219	OK	0	418.8785116	0.424916145	0
BNIP3	4	1	2.943675295	1.725236414	OK	0	216.0187422	0.219118728	0
C10orf99	4	1	2.477624074	-0.382632496	OK	0	300.3851886	0.30470966	0
C15orf48	4	1	2.983681694	2.792490004	OK	0	499.9754921	0.507173773	0
C4orf3	4	1	2.075654403	1.675032614	OK	0	349.1688992	0.354198213	0
C8orf82	4	1	2.740573541	-0.103051426	OK	0	129.4587772	0.131316005	0
CALB2	4	1	2.740789906	2.808746813	OK	0	230.5555349	0.233783494	0
CALML5	4	1	1.214829371	1.378087996	OK	0	329.1750199	0.333913761	0
CAPN14	4	1	0.013118998	0.054205893	OK	0	428.6675591	0.434848227	0
CASTOR1	4	1	2.523007528	0.100628851	OK	0	217.3463446	0.220467274	0
CBR3	4	1	2.661850826	-0.336713554	OK	0	226.9621347	0.230228449	0
CCNYL1	4	1	2.590217487	2.890558241	OK	0	88.93180731	0.09019631	0
CD24	4	1	1.88766791	1.186091898	OK	0	620.4954414	0.629444398	0
CDA	4	1	2.855691091	1.064485071	OK	0	294.1648014	0.298380794	0
CDH26	4	1	0.053598539	0.601023196	OK	0	532.2038556	0.539873649	0
CEACAM1	4	1	0.515894607	1.404805659	OK	0	320.9092895	0.325516697	0
CEACAM5	4	1	0.372260944	1.135502814	OK	0	568.2811714	0.576472267	0
CEACAM6	4	1	0.283144251	1.206619261	OK	0	452.4860501	0.45900734	0
CES1	4	1	2.360600368	-0.429948332	OK	0	246.4902038	0.250017345	0
CFD	4	1	1.187712506	1.825720785	OK	0	122.640353	0.124398921	0
CGN	4	1	0.209066883	0.820115565	OK	0	194.8267738	0.197618967	0
CITED4	4	1	2.740355984	-0.243822338	OK	0	325.7651487	0.33045872	0
CLCA4	4	1	0.761373394	0.817988871	OK	0	482.5257374	0.489482999	0
CLDN10	4	1	2.529442683	2.208148478	OK	0	250.1852554	0.253675351	0
CLIC3	4	1	1.230176911	1.439811228	OK	0	614.1757048	0.623036612	0
CNFN	4	1	2.98389031	2.639574526	OK	0	409.1930547	0.415089104	0
CNN3	4	1	2.288358704	0.575354098	OK	0	357.4356518	0.362585813	0
COX7A1	4	1	2.974779621	3.300049303	OK	0	40.77811439	0.041349314	0
CPVL	4	1	1.721601025	-0.703951599	OK	0	100.6883141	0.102125067	0
CRCT1	4	1	3.082562939	2.963946818	OK	0	534.7643131	0.542462813	0
CRISP3	4	1	2.897492186	2.875373362	OK	0	735.9159145	0.7464386	0
CRNN	4	1	2.821413413	1.232228278	OK	0	654.9954888	0.664442004	0
CRYAB	4	1	2.911975161	1.436168192	OK	0	233.3577469	0.236711137	0
CST3	4	1	1.679118053	1.14290428	OK	0	226.8616732	0.230121189	0
CST6	4	1	2.639604107	2.887928007	OK	0	511.3496995	0.518695673	0
CSTA	4	1	2.129539744	1.450359343	OK	0	337.1288839	0.341971213	0
CSTB	4	1	2.192768947	1.327327727	OK	0	319.268586	0.323840315	0
CTNNBIP1	4	1	2.674344794	-0.175788881	OK	0	319.16856	0.323766695	0
CTTNBP2	4	1	1.952348367	-0.34841633	OK	0	341.4477042	0.346364698	0
CWH43	4	1	2.460269347	0.201596258	OK	0	285.178642	0.289265824	0
CXCL17	4	1	0.921782557	1.222795962	OK	0	477.262265	0.484144516	0
CXXC5	4	1	0.79262969	1.69540882	OK	0	230.1887361	0.233499427	0
CYP2C18	4	1	1.678719238	-0.164933921	OK	0	187.6605934	0.190352943	0
CYP4B1	4	1	1.47916241	-0.636459829	OK	0	267.3179709	0.271165681	0
CYP51A1	4	1	-0.950810059	0.937862395	OK	0	198.7919872	0.20165127	0
CYSRT1	4	1	1.584583656	2.066393374	OK	0	625.8978119	0.634925839	0
DDAH2	4	1	0.487227277	0.875252245	OK	0	209.6080141	0.212622787	0
DEFB1	4	1	1.550190524	-0.672664644	OK	0	305.7592893	0.310163766	0
DEGS2	4	1	2.481653825	-0.1639874	OK	0	370.6941318	0.376030087	0
DIO2	4	1	0.314949886	1.053826331	OK	0	444.5257296	0.450923741	0
DMKN	4	1	1.002553598	1.290452956	OK	0	417.0286926	0.423040556	0
DNASE1L3	4	1	2.687181011	-0.693595173	OK	0	60.31481784	0.061170329	0
DSC2	4	1	1.595102743	-0.392863037	OK	0	493.7029115	0.500821855	0
DSG1	4	1	2.346274987	-0.184188368	OK	0	214.1713862	0.217209757	0
DYNLT3	4	1	1.464370028	0.097176073	OK	0	459.416003	0.466040665	0
ECM1	4	1	3.085807577	2.952061175	OK	0	518.096523	0.525563313	0
EGLN3	4	1	0.061429218	0.815330504	OK	0	245.6607025	0.24919467	0
EHD3	4	1	0.093974367	1.610520838	OK	0	191.7791492	0.194533193	0
ELL2	4	1	1.705955402	-0.297456505	OK	0	209.4195313	0.21243179	0
ELOVL4	4	1	0.739471227	0.677093504	OK	0	223.2936189	0.226502083	0
EMP1	4	1	2.829419986	2.286886214	OK	0	443.7120214	0.450108244	0
ENDOU	4	1	2.54721512	0.610316275	OK	0	334.4786733	0.339278169	0

EPB41L3	4	1	2.862268821	1.611585139	OK	0	257.5623751	0.261211827	0
EPHX3	4	1	1.341057495	1.161260126	OK	0	291.4868824	0.29568367	0
EREG	4	1	2.331188337	1.988231657	OK	0	235.7198365	0.239058113	0
ETNK2	4	1	2.509976045	-0.274088384	OK	0	134.7491731	0.136673225	0
EVPL	4	1	0.337948219	0.063773154	OK	0	378.3322097	0.383785382	0
F3	4	1	2.918667093	1.333381651	OK	0	114.480609	0.116096671	0
FAM25A	4	1	2.948396937	3.376667021	OK	0	608.867759	0.61761339	0
FAM3B	4	1	0.796358824	0.809432505	OK	0	584.4285613	0.592859181	0
FAM3D	4	1	2.300568596	1.917214392	OK	0	663.9994596	0.673563586	0
FAM83A	4	1	0.172454015	1.219733237	OK	0	286.1997994	0.290319907	0
FETUB	4	1	0.283221856	0.810892104	OK	0	475.6023852	0.482432717	0
FGF11	4	1	-0.058230265	0.448318957	OK	0	155.6722978	0.157901843	0
FGFBP1	4	1	-0.965856775	0.937648772	OK	0	309.3057201	0.313761821	0
FMO2	4	1	-0.152447089	0.761231898	OK	0	177.0366765	0.179580852	0
FNDC4	4	1	2.759022371	2.182997702	OK	0	306.9385248	0.311293474	0
FUOM	4	1	1.061342136	1.679071902	OK	0	103.8095339	0.105295568	0
GABRP	4	1	0.087931052	0.24921131	OK	0	375.323666	0.380732484	0
GADD45G	4	1	2.479283706	-0.259688856	OK	0	98.44566144	0.099849516	0
GBP6	4	1	1.611602858	-0.215933563	OK	0	512.5143507	0.519903975	0
GCNT3	4	1	0.260955468	0.952297686	OK	0	404.6933837	0.410508647	0
GDPD3	4	1	2.593174473	0.719751356	OK	0	469.1828156	0.47593696	0
GJB2	4	1	1.371929959	0.681958197	OK	0	355.6477572	0.360773205	0
GJB6	4	1	1.207641379	0.704520701	OK	0	402.2779436	0.408076588	0
GLTP	4	1	1.380558357	0.891863345	OK	0	304.6894249	0.309077658	0
GNG4	4	1	2.075480357	1.869416712	OK	0	431.5150323	0.437704767	0
GPR160	4	1	0.062666372	0.886410712	OK	0	167.3407471	0.169732357	0
GPX2	4	1	2.793054715	-0.808768989	OK	0	133.2438973	0.135154468	0
GPX3	4	1	2.613265649	0.909512041	OK	0	283.5345129	0.287605313	0
GRHL3	4	1	1.211949841	-0.140882732	OK	0	251.2249215	0.254839698	0
GSN	4	1	1.752858893	0.796848296	OK	0	376.292767	0.381714297	0
GSTA1	4	1	1.779266194	-0.627222778	OK	0	137.3851127	0.139339134	0
GSTM3	4	1	2.777192608	-0.850568535	OK	0	217.7896179	0.22092253	0
GSTT2B	4	1	2.679171935	2.768601893	OK	0	42.74445998	0.043344181	0
HIF1A	4	1	1.945037738	0.138905524	OK	0	149.890068	0.15204252	0
HMGCS1	4	1	-0.91551946	0.911790846	OK	0	275.885265	0.279857904	0
HMOX2	4	1	1.807944611	2.239630698	OK	0	215.2434171	0.21834002	0
HOPX	4	1	2.457014338	1.924052714	OK	0	471.7445903	0.47854694	0
HPSE	4	1	2.156444327	2.156604765	OK	0	81.75021888	0.08291292	0
HS3ST1	4	1	-0.019545897	0.565165995	OK	0	308.3170718	0.312756955	0
HSPA1A	4	1	2.025708572	-0.889182808	OK	0	220.5169297	0.223689294	0
HSPA1B	4	1	1.987976924	-0.835098745	OK	0	166.0934935	0.168480686	0
IDI1	4	1	-0.993477091	0.983612536	OK	0	154.2160302	0.156430856	0
IFFO2	4	1	0.368857161	-0.038939716	OK	0	285.3620682	0.28947023	0
IFI35	4	1	-0.257952913	0.058422564	OK	0	174.3475072	0.176852125	0
IGFL1	4	1	2.89848663	1.440986632	OK	0	176.6862462	0.179207675	0
IL12A-AS1	4	1	2.602432743	2.423913	OK	0	377.3523034	0.382700199	0
INSIG1	4	1	-0.883813008	0.945471285	OK	0	142.6558754	0.144701404	0
ISG20	4	1	2.134493724	1.835484503	OK	0	260.467267	0.264206833	0
IVL	4	1	2.328467385	0.443246363	OK	0	548.5438079	0.556455175	0
KALRN	4	1	0.312571124	0.790946959	OK	0	192.7387168	0.195502054	0
KCNK7	4	1	2.536251799	-0.579657795	OK	0	292.2925825	0.296501176	0
KIAA1211L	4	1	1.704932646	-0.270336153	OK	0	173.7964577	0.176291552	0
KIF21A	4	1	1.416155771	0.667404173	OK	0	240.6225903	0.244085453	0
KLK10	4	1	0.531004116	1.157840727	OK	0	303.5932559	0.307966784	0
KLK11	4	1	1.636212066	0.60234165	OK	0	442.831086	0.449215493	0
KLK12	4	1	0.345472769	1.598014353	OK	0	623.3935802	0.632373269	0
KLK13	4	1	0.474616066	1.37721443	OK	0	495.5938624	0.502740792	0
KLK6	4	1	0.396938965	1.916517733	OK	0	157.4716702	0.159700176	0
KLK7	4	1	0.513064877	1.071659087	OK	0	394.0594654	0.399727459	0
KLK8	4	1	0.594659612	1.212487219	OK	0	184.9105103	0.187568815	0
KRT24	4	1	0.51401724	1.510684489	OK	0	279.6687924	0.283668688	0
KRT4	4	1	1.33734189	-0.107780458	OK	0	510.4046644	0.517760632	0
KRT6A	4	1	2.299380079	-0.224249126	OK	0	489.094413	0.49614132	0
KRT6B	4	1	2.369384781	-0.192881586	OK	0	379.5929138	0.385063679	0
KRT6C	4	1	2.39508988	-0.196428539	OK	0	295.7432313	0.300000403	0
KRT78	4	1	3.040285841	3.064028738	OK	0	676.0771032	0.685800214	0
KRTDAP	4	1	0.817564123	1.374743937	OK	0	86.4545668	0.087676187	0
LCN2	4	1	0.287852482	2.102612494	OK	0	406.9989418	0.412864953	0
LGALS7B	4	1	0.617081628	-0.052035572	OK	0	297.1630148	0.301437822	0
LINC02487	4	1	2.338762537	2.180424212	OK	0	313.4188888	0.317835254	0
LIPH	4	1	0.696610615	1.137707232	OK	0	328.1725215	0.332871327	0
LPCAT4	4	1	0.042642609	0.442589758	OK	0	224.577213	0.227806447	0
LRRC8A	4	1	0.365402893	0.177682875	OK	0	245.6056258	0.249141065	0

LY6G6C	4	1	0.562588797	1.142948626	OK	0	270.3565806	0.274244555	0
LYPD2	4	1	2.941634432	2.830833433	OK	0	625.1571043	0.634152565	0
LYPD3	4	1	1.610132233	0.842330454	OK	0	476.2371706	0.483102607	0
MAF	4	1	1.601964787	-0.427716018	OK	0	206.0983223	0.209063118	0
MAFB	4	1	2.128156201	-0.676138879	OK	0	232.9020533	0.236253847	0
MAL	4	1	2.821851627	1.165320872	OK	0	597.542185	0.606161367	0
MAL2	4	1	1.590066746	0.476337908	OK	0	473.1410088	0.479962453	0
MBOAT2	4	1	0.428181962	0.63414192	OK	0	367.9772419	0.373278308	0
ME1	4	1	1.418652252	0.551196097	OK	0	214.381397	0.217458054	0
MGLL	4	1	2.435696975	-0.088659765	OK	0	514.0350502	0.521448241	0
MGST1	4	1	2.816961662	-0.860919716	OK	0	145.4212036	0.147509695	0
MIR210HG	4	1	2.529174582	1.486824988	OK	0	188.879432	0.191590452	0
MROH6	4	1	0.1757188	1.771239756	OK	0	189.8738339	0.192598306	0
MSMO1	4	1	-0.964754924	0.951216219	OK	0	167.6398132	0.17004831	0
MT1X	4	1	1.249868617	-0.953428508	OK	0	419.4349072	0.425481502	0
MUC15	4	1	1.499438242	-0.156594278	OK	0	391.3346429	0.396973607	0
MUC21	4	1	2.914217488	1.21543598	OK	0	699.7974673	0.709878134	0
MUC4	4	1	0.076539413	0.971407889	OK	0	241.1563831	0.244620728	0
MYH14	4	1	0.457549438	0.138987063	OK	0	368.9494007	0.374264688	0
NAGK	4	1	2.31937565	0.31576824	OK	0	393.0135785	0.3986788	0
NBEAL2	4	1	0.383789912	0.043907402	OK	0	277.5770228	0.281572603	0
NDUFA4L2	4	1	2.797015444	1.523150919	OK	0	276.55743	0.280534111	0
NQO1	4	1	2.80452408	-0.762233974	OK	0	235.9907708	0.239386545	0
NUAK2	4	1	0.500038013	0.875808714	OK	0	134.9488258	0.136880176	0
NUCB2	4	1	2.366094962	0.279266356	OK	0	442.5090941	0.448889253	0
NUDT8	4	1	2.78912522	-0.128304245	OK	0	362.8969509	0.368127035	0
OAS1	4	1	1.002851867	0.851389407	OK	0	234.2392558	0.237609954	0
PALMD	4	1	2.448580161	0.162383555	OK	0	173.6776309	0.176173171	0
PAQR8	4	1	2.650168554	0.643744467	OK	0	176.6039156	0.179127011	0
PCDH1	4	1	0.242435232	1.470964907	OK	0	248.4782395	0.25204774	0
PDLIM2	4	1	2.020938173	1.288282393	OK	0	267.415289	0.271266086	0
PGD	4	1	0.832945183	-0.035960676	OK	0	352.5557302	0.357635586	0
PHLDB3	4	1	0.859459805	1.56955242	OK	0	104.6262452	0.10612277	0
PI3	4	1	0.651783243	1.354278563	OK	0	407.2748079	0.413141209	0
PIM1	4	1	2.146765963	1.91542816	OK	0	263.4145094	0.267206758	0
PLAC8	4	1	0.144597188	0.950612543	OK	0	419.7971349	0.425848322	0
PLBD1	4	1	1.078558013	1.830584048	OK	0	281.9677579	0.286028024	0
PMM1	4	1	2.155951992	1.748418806	OK	0	207.2343136	0.210215147	0
PPM1K	4	1	1.830320135	-0.580988409	OK	0	138.4886736	0.140473692	0
PPP1R3C	4	1	0.153254465	1.80726528	OK	0	237.4020019	0.240802696	0
PRSS3	4	1	0.542852387	1.180968283	OK	0	629.5136468	0.638585225	0
PTGR1	4	1	2.57114257	-0.548101665	OK	0	281.6081875	0.285663606	0
PTN	4	1	1.697821394	-0.685350181	OK	0	326.1350535	0.330834138	0
RAB10	4	1	1.839724854	0.852697848	OK	0	370.9534977	0.376298335	0
RAET1E	4	1	1.454727665	0.269239901	OK	0	328.9915155	0.333726505	0
RAET1L	4	1	0.805305049	1.064347742	OK	0	355.554163	0.36066827	0
RASGRP1	4	1	-0.114824518	0.029162405	OK	0	246.8680743	0.250418403	0
RBP1	4	1	2.246383802	1.950140951	OK	0	66.85399463	0.067804417	0
RBP7	4	1	2.573333398	1.235962389	OK	0	209.4662368	0.212477003	0
RHCG	4	1	1.694526151	1.07136154	OK	0	628.5327879	0.637593626	0
RHOD	4	1	1.926361099	1.449175833	OK	0	296.7577027	0.301032408	0
RTKN2	4	1	2.170628206	2.034021376	OK	0	206.497866	0.209466094	0
S100A12	4	1	2.697557107	2.427675246	OK	0	288.4841636	0.292621847	0
S100A14	4	1	2.043817178	1.279992579	OK	0	107.595503	0.109126691	0
S100A4	4	1	1.698409871	1.330492972	OK	0	78.06581567	0.079180974	0
S100A6	4	1	2.08193554	1.355507849	OK	0	175.1576173	0.177668345	0
S100A7	4	1	0.50630121	1.987516402	OK	0	182.4658658	0.185082021	0
S100A8	4	1	2.121369497	-1.022029878	OK	0	350.5692893	0.355614645	0
SAMD5	4	1	1.966120139	-0.293299677	OK	0	212.2935678	0.21533272	0
SBSN	4	1	1.093760059	1.207814215	OK	0	506.6516845	0.51395684	0
SCD	4	1	-0.839996084	0.86882448	OK	0	209.145983	0.212152866	0
SCGB1A1	4	1	1.448552803	0.937721251	OK	0	85.4408255	0.086658265	0
SCUBE2	4	1	0.044076935	0.279218672	OK	0	170.772135	0.173220448	0
SDR16C5	4	1	1.681410924	0.284152029	OK	0	386.9208303	0.392497552	0
SERPINB1	4	1	2.124381438	0.752237795	OK	0	461.2169169	0.467864382	0
SERPINB11	4	1	2.617615119	0.656964777	OK	0	75.04807329	0.07611005	0
SERPINB6	4	1	1.261897013	1.348103999	OK	0	302.9232283	0.307286883	0
SF3B3	4	1	2.726533786	1.474830149	OK	0	169.2326086	0.171664326	0
SFTA2	4	1	2.847560183	2.707776545	OK	0	484.0419175	0.490960506	0
SH3GL1	4	1	0.254033223	1.882952212	OK	0	270.8452574	0.274745051	0
SHROOM3	4	1	2.253305808	2.040335177	OK	0	238.2262222	0.241646043	0
SLC26A2	4	1	1.112812982	-0.321353199	OK	0	257.2039339	0.260906123	0
SLC35C1	4	1	0.980233729	1.880276678	OK	0	194.9546313	0.197755254	0

SLPI	4	1	0.859082891	1.300873278	OK	0	544.0574276	0.551905039	0
SLURP1	4	1	2.829994098	1.155082701	OK	0	594.8192823	0.603391556	0
SMAGP	4	1	1.520575122	1.925748347	OK	0	349.3645672	0.354398742	0
SMIM5	4	1	1.426347867	1.57780218	OK	0	487.5480726	0.494576609	0
SNX31	4	1	0.590559558	0.757567404	OK	0	219.0628636	0.222205127	0
SPAG17	4	1	1.73723884	-0.269332887	OK	0	227.284024	0.23054945	0
SPINK5	4	1	1.724913255	0.030583618	OK	0	579.7515218	0.588112212	0
SPINK7	4	1	3.047698752	2.81287813	OK	0	605.8485447	0.614539222	0
SPRR1A	4	1	1.75470765	1.922407149	OK	0	392.3951057	0.398040713	0
SPRR1B	4	1	0.537957654	1.829923628	OK	0	378.2818572	0.383733197	0
SPRR2A	4	1	0.230627493	2.248156546	OK	0	310.9490761	0.31542538	0
SPRR2D	4	1	0.228177324	2.289025305	OK	0	378.9342593	0.384366379	0
SPRR2E	4	1	0.292070941	2.122065543	OK	0	232.6424019	0.235981389	0
SPRR2F	4	1	0.32757105	2.066920756	OK	0	45.12483153	0.04567398	0
SPRR3	4	1	0.452651993	0.557017325	OK	0	489.8141973	0.496876975	0
SQLE	4	1	-0.960737332	0.928280829	OK	0	238.350283	0.241780797	0
SRPX2	4	1	2.283137337	2.374033449	OK	0	261.7557817	0.265483686	0
ST3GAL4	4	1	2.348348991	2.099584578	OK	0	460.4004572	0.466985937	0
STN1	4	1	1.917301074	2.140528677	OK	0	313.9247032	0.318446395	0
SUCO	4	1	2.346720711	0.310316561	OK	0	66.78028391	0.067730809	0
SULT2B1	4	1	1.283354536	0.904190062	OK	0	550.0032649	0.557936022	0
TGM1	4	1	1.230724738	0.828541277	OK	0	437.7703088	0.444079574	0
TGM3	4	1	2.873057739	1.111881255	OK	0	660.8776151	0.670402302	0
TM7SF2	4	1	0.997985192	1.042426108	OK	0	508.0459673	0.515371305	0
TMEM154	4	1	1.304724858	0.400205134	OK	0	331.3103783	0.336083837	0
TMEM184A	4	1	0.453562186	0.099510191	OK	0	257.9570182	0.261668345	0
TMEM265	4	1	1.326204315	1.658581255	OK	0	382.3852645	0.387895067	0
TMEM45B	4	1	0.345423535	0.973878859	OK	0	457.9010382	0.464496985	0
TMEM79	4	1	1.575280324	0.764211176	OK	0	280.1790486	0.284213603	0
TMPRSS11B	4	1	2.823401943	2.631305216	OK	0	713.8195537	0.724078489	0
TP53I3	4	1	2.734518186	0.933127879	OK	0	274.306019	0.278255056	0
TPRG1	4	1	2.007654086	1.882387637	OK	0	530.754327	0.538400801	0
TRNP1	4	1	0.038939015	0.500239848	OK	0	420.8161194	0.426882461	0
TSPAN3	4	1	-0.11121945	0.357102869	OK	0	278.3696823	0.282378671	0
TST	4	1	1.293027536	1.491883753	OK	0	360.9137332	0.366114993	0
TUBA4A	4	1	2.469003097	0.029910563	OK	0	300.8033343	0.305136339	0
UPK1B	4	1	0.215960518	0.89030361	OK	0	147.9459828	0.150052629	0
UPK3BL1	4	1	1.404047862	1.79719591	OK	0	359.6832323	0.364866304	0
VAT1	4	1	0.079907969	1.560358523	OK	0	294.9174343	0.299164305	0
WNK4	4	1	1.57008572	-0.229618313	OK	0	226.5163455	0.229769044	0
WNT5A	4	1	1.877450899	-0.10791278	OK	0	161.7628873	0.164080699	0
ZBED2	4	1	2.914160267	1.823194979	OK	0	136.8986165	0.138827077	0
ZDHHC21	4	1	1.375249133	0.227308749	OK	0	77.28935721	0.07839129	0
ABLIM1	5	1	-0.217179641	3.794879435	OK	0	327.5709265	0.332290726	0
ABLIM3	5	1	1.503047899	4.188347815	OK	0	422.3491115	0.42838339	0
AC004816.1	5	1	1.851444617	3.969703673	OK	0	241.5058012	0.244973242	0
AC006333.2	5	1	2.13527073	4.528072356	OK	0	96.67960308	0.098057408	0
ACADM	5	1	1.650183753	3.311115263	OK	0	177.5233061	0.180075041	0
ADGRF1	5	1	0.548774854	3.839282988	OK	0	319.8729142	0.324472391	0
ADIPOR1	5	1	1.811903492	3.611345766	OK	0	169.9591125	0.172401753	0
ADIRF	5	1	2.665994898	3.774622439	OK	0	311.1721144	0.31565453	0
AFDN	5	1	-0.805897101	3.313125132	OK	0	299.8386758	0.304157696	0
AGFG2	5	1	0.393171415	3.175921915	OK	0	118.2191598	0.11990761	0
AIF1L	5	1	2.708969847	3.520309923	OK	0	559.0065887	0.567028923	0
AL121761.1	5	1	0.752994732	2.716315268	OK	0	390.2613009	0.395876355	0
AL121933.2	5	1	0.371199444	2.82887411	OK	0	116.2867347	0.11787498	0
AL133453.1	5	1	0.284853057	3.984752177	OK	0	164.2049455	0.166556028	0
AL512274.1	5	1	-0.388732537	3.327167509	OK	0	202.180458	0.205083891	0
AL627171.2	5	1	0.660119728	3.4499979	OK	2.57E-119	23.19471124	0.023516268	2.59E-119
ALDH1A3	5	1	0.683515654	4.19603443	OK	0	343.992798	0.348918627	0
ANGPTL4	5	1	0.859332979	2.250810145	OK	0	101.3838166	0.102833744	0
ANXA11	5	1	1.525561587	3.458152769	OK	0	232.8567725	0.236207695	0
ANXA9	5	1	1.158595071	3.677791117	OK	0	569.6644374	0.577825339	0
AP000866.2	5	1	2.281986371	4.074776648	OK	0	282.3087951	0.286275318	0
ARHGAP27	5	1	0.604199723	3.512483118	OK	0	161.8326417	0.164151927	0
ARHGDIB	5	1	1.870288864	3.00214052	OK	0	101.4788249	0.102925561	0
ATP6V1C2	5	1	0.85270694	4.16892433	OK	0	489.3704892	0.496323541	0
AVPI1	5	1	1.252370552	5.156238554	OK	0	86.16357186	0.087393296	0
B3GNT3	5	1	0.95985432	3.224968909	OK	0	409.9786668	0.415810769	0
B3GNT8	5	1	0.83699346	2.476715563	OK	0	131.5788502	0.133463495	0
B4GALT5	5	1	0.114755825	3.200644968	OK	0	121.3325945	0.123070728	0
BAIAP2L2	5	1	2.360195771	3.156896113	OK	0	204.8559365	0.207763538	0
BCAS1	5	1	0.791616612	2.966410635	OK	0	226.2373063	0.229458181	0

BICDL1	5	1	1.411277429	4.53283119	OK	0	89.13088503	0.090402636	0
BX255925.3	5	1	1.477150813	2.671066759	OK	0	227.5627156	0.23083529	0
C15orf62	5	1	1.008478419	3.177142142	OK	0	272.4061085	0.276294131	0
C1orf116	5	1	-0.060886666	3.797162054	OK	0	260.1860847	0.263931602	0
C5orf66-AS1	5	1	2.320603505	4.17116165	OK	0	177.4219913	0.179972482	0
C6orf132	5	1	1.550416485	4.281843184	OK	0	324.951936	0.329633766	0
CAMK2N1	5	1	1.366101042	3.570693968	OK	0	720.0195302	0.730359679	0
CAPN5	5	1	0.659941823	3.13435602	OK	0	206.50282	0.20945108	0
CASP8	5	1	1.744439141	4.65646839	OK	0	51.36895228	0.052097283	0
CCDC127	5	1	1.841410176	3.403882025	OK	0	88.04063806	0.089295861	0
CCNG2	5	1	0.08977522	3.817274569	OK	0	309.511388	0.313960732	0
CD59	5	1	1.786743239	2.361181258	OK	0	285.6603327	0.289773774	0
CD68	5	1	1.048223824	2.470702647	OK	0	146.6964098	0.148797533	0
CDC42EP5	5	1	1.028670431	2.805127142	OK	0	119.8495075	0.121568234	0
CDKN2AIP	5	1	-0.601762756	3.547561644	OK	0	181.2291134	0.183834587	0
CEACAM7	5	1	0.550712541	2.842703341	OK	0	121.9883098	0.123643954	0
CFL2	5	1	2.030292646	3.209030627	OK	1.09E-290	36.41840935	0.036928002	1.10E-290
CKB	5	1	2.001852766	3.755930899	OK	0	120.3262199	0.122051663	0
CLIP1	5	1	-0.859474047	2.905121802	OK	0	309.4371327	0.313895078	0
CPEB4	5	1	-0.231700167	3.266991614	OK	0	169.5389228	0.171972937	0
CPPED1	5	1	2.085446254	3.735492705	OK	0	163.1978539	0.165541678	0
CRYM	5	1	1.917866842	4.557875632	OK	0	364.347147	0.36939243	0
CTSV	5	1	1.107496039	3.798141478	OK	0	224.6085663	0.227822945	0
CTTNBP2NL	5	1	-0.718484386	3.646636484	OK	0	153.6084369	0.155813986	0
CYTOR	5	1	1.116177485	3.802751063	OK	0	142.4010132	0.144432454	0
DDIT4	5	1	0.896957774	2.290771483	OK	0	66.00636315	0.066947302	0
DENND10	5	1	1.980604187	2.828616141	OK	0	144.8079838	0.146886591	0
DHRS9	5	1	1.585832909	4.68468952	OK	0	693.6118164	0.703453951	0
DNAJA4	5	1	-0.55823718	2.998198984	OK	0	74.40412268	0.075464057	0
DNAJC5	5	1	1.229144142	3.947332381	OK	0	138.0881593	0.140069128	0
DOCK9	5	1	-0.415216311	3.633717058	OK	0	144.7966763	0.146873481	0
DTX2	5	1	0.690559522	3.525752543	OK	0	121.0784942	0.122814231	0
DUOX2	5	1	0.207682565	2.870212077	OK	0	90.99370595	0.092289116	0
DUOXA2	5	1	0.234775618	2.687603949	OK	0	97.45812282	0.098785778	0
DUSP4	5	1	1.816091434	4.581932066	OK	0	385.3263131	0.390707734	0
ELF3	5	1	1.002270341	2.629482268	OK	0	306.605412	0.31102224	0
ELF3-AS1	5	1	1.941260234	4.355633734	OK	0	278.6263531	0.282592179	0
EPS8L1	5	1	0.754541815	3.745923041	OK	0	529.1657014	0.536786123	0
EPS8L2	5	1	1.287296996	3.232457159	OK	0	231.9668079	0.235305341	0
ESAM	5	1	1.521073297	4.61438465	OK	0	512.8087214	0.520026763	0
FAM107B	5	1	1.112190858	4.826979636	OK	0	177.8900624	0.180430125	0
FBXO32	5	1	1.164076195	4.145256041	OK	0	223.7851275	0.227001789	0
FBXO34	5	1	-0.494350537	3.499712942	OK	0	138.5374189	0.140525067	0
FBXW11	5	1	-0.573653921	3.765322207	OK	0	83.83945136	0.085037327	0
FCHO2	5	1	-0.793487533	3.428331374	OK	0	219.8072272	0.222968952	0
FRMD4B	5	1	-0.868725284	2.618279932	OK	0	234.6813625	0.238059037	0
FTH1	5	1	2.290752903	4.18931198	OK	0	204.1897415	0.207120341	0
FUT3	5	1	0.442447022	3.280181883	OK	0	413.1890585	0.419137841	0
FUT6	5	1	0.545535073	3.329130648	OK	0	382.9857088	0.3884797	0
GABARAPL2	5	1	2.32674636	4.353869437	OK	0	237.2393712	0.240653954	0
GALNT1	5	1	-0.647503957	3.67525482	OK	0	263.3999098	0.267192499	0
GCC2	5	1	-0.079069659	3.961014269	OK	0	100.5941233	0.102034275	0
GCHFR	5	1	1.234852657	3.585375307	OK	0	226.9033018	0.230167588	0
GFOD2	5	1	0.210803047	3.698657511	OK	0	99.32999089	0.100749341	0
GMDS	5	1	2.136051074	2.485879896	OK	0	239.7198196	0.243167406	0
GNA15	5	1	1.357638404	3.187748907	OK	0	264.9053798	0.26871901	0
GPRC5A	5	1	1.185081646	4.240655897	OK	0	212.3383391	0.215373896	0
GPT2	5	1	1.065448925	3.264035223	OK	0	174.4665032	0.176973331	0
GRHL1	5	1	-0.454951748	2.886757372	OK	0	353.7192684	0.358814243	0
H19	5	1	1.840774671	2.769955157	OK	0	137.5867092	0.139556512	0
H1F0	5	1	0.761057668	2.470832823	OK	0	189.7791318	0.192508179	0
H2AFJ	5	1	2.400450722	4.163906096	OK	0	269.0300459	0.272903699	0
HEBP2	5	1	2.182238952	3.286148547	OK	0	362.6946454	0.367918582	0
HLCS	5	1	2.068586246	4.209116934	OK	0	85.73277037	0.086951492	0
HMOX1	5	1	0.779808358	4.198033331	OK	0	448.5879042	0.454981389	0
HOTAIRM1	5	1	2.21312143	4.560224531	OK	0	95.55791247	0.096924821	0
HS3ST6	5	1	2.336584226	4.240200995	OK	0	144.1704474	0.146235816	0
HSPB8	5	1	0.304159955	3.347195147	OK	0	271.3592445	0.275262104	0
IL1A	5	1	1.546708778	4.849163054	OK	0	257.9598712	0.261592534	0
IL36A	5	1	1.322485909	4.87915325	OK	0	602.4112865	0.610955103	0
IL36RN	5	1	0.264174119	2.66430807	OK	0	221.0506508	0.224127572	0
ITSN2	5	1	-0.669386491	3.280333041	OK	0	203.8113867	0.206742155	0
KATNBL1	5	1	1.925369397	3.441823481	OK	0	164.5362015	0.166900391	0

KAZN	5	1	1.359249339	3.553071974	OK	0	247.0795528	0.250634955	0
KIFC3	5	1	1.571455554	4.702668188	OK	0	263.9422913	0.26769507	0
KRT23	5	1	0.871362833	2.978250025	OK	0	194.875347	0.197610113	0
KRT7	5	1	1.646416262	2.875129698	OK	0	200.9638865	0.203845271	0
KRT80	5	1	-0.295225366	2.774516581	OK	0	433.1171235	0.439350742	0
LEXM	5	1	1.272288517	3.287549494	OK	0	311.254817	0.315633758	0
LGALS3	5	1	2.160169975	4.488221167	OK	0	222.8252583	0.226024261	0
LINC02303	5	1	2.070167199	3.185497282	OK	0	360.8859439	0.365870924	0
LIPE-AS1	5	1	2.196977154	3.69919586	OK	0	105.8625952	0.107358008	0
LMO2	5	1	0.92434612	2.913143633	OK	0	290.3298077	0.294458211	0
LMO7	5	1	-0.664312466	3.485337256	OK	0	335.8673351	0.340706631	0
LPIN2	5	1	0.891191842	4.190340994	OK	0	253.9798394	0.257588518	0
LRG1	5	1	0.588316188	2.90701866	OK	0	63.62816472	0.064533394	0
LY6K	5	1	2.176342145	4.376996992	OK	0	109.3602321	0.110926012	0
MAB21L4	5	1	1.804627076	2.64653158	OK	0	455.2922248	0.461857189	0
MACC1	5	1	-0.330576285	3.60785055	OK	0	166.5992575	0.16899126	0
MALL	5	1	-0.335006341	3.737776755	OK	0	505.074935	0.512359172	0
MAP1LC3A	5	1	2.415848867	4.099238394	OK	0	233.0291241	0.23637113	0
MAPK3	5	1	1.143363163	3.356910704	OK	0	183.2077446	0.185839985	0
MAPK8	5	1	1.43880941	4.940137862	OK	0	77.64106605	0.078747147	0
5-Mar	5	1	2.12173082	4.037055491	OK	0	186.8426922	0.189529083	0
MDFIC	5	1	1.650655583	3.576622485	OK	0	93.29667592	0.094630302	0
METRNL	5	1	1.661725537	3.070854185	OK	0	465.5651301	0.472278351	0
MGAT1	5	1	1.345754907	3.920424937	OK	0	152.810058	0.155004116	0
MPRIP	5	1	1.506429568	4.165702818	OK	0	145.1902765	0.147273551	0
MPZL3	5	1	0.423166976	3.243092535	OK	0	186.8303627	0.189499718	0
MTERF4	5	1	2.188767687	3.515886782	OK	0	73.91733955	0.074971993	0
MUC1	5	1	0.063736156	3.38903427	OK	0	432.0390421	0.438262118	0
MUC20	5	1	1.590833024	2.764943121	OK	0	325.1346702	0.32981643	0
MUC22	5	1	1.379609302	4.635846136	OK	0	304.9171991	0.309142369	0
MXD1	5	1	0.794135206	4.421854018	OK	0	351.7035145	0.356768779	0
MYEOV	5	1	2.141988889	3.434927939	OK	0	254.0325606	0.257679879	0
MYL12A	5	1	2.239586369	3.458912371	OK	0	297.2749322	0.301553588	0
MYO5B	5	1	-0.298074468	2.894899844	OK	0	232.9744069	0.23632279	0
MYO6	5	1	-0.587553366	4.048232077	OK	0	214.7233112	0.21781241	0
NAMPT	5	1	-0.506788477	3.149124144	OK	0	299.2756443	0.303586657	0
NAPRT	5	1	1.497659162	3.19459915	OK	0	262.3857703	0.266163749	0
NCCRP1	5	1	1.198090748	2.270748614	OK	0	630.7482515	0.639842124	0
NDRG2	5	1	0.029266969	3.900654314	OK	0	271.247373	0.275152489	0
NIBAN2	5	1	1.787779943	2.769644736	OK	0	359.8871766	0.365073628	0
NT5C2	5	1	-0.064067706	3.433454989	OK	0	191.4711457	0.194223297	0
PCBP1-AS1	5	1	2.084335581	3.0040493	OK	0	92.19801988	0.093511388	0
PDCD6	5	1	2.024524585	3.328223227	OK	0	280.1072271	0.284140491	0
PHACTR4	5	1	-0.506470307	3.799765108	OK	0	159.9734864	0.162271697	0
PHLDA1	5	1	0.952495844	4.285964011	OK	0	463.6616327	0.470329947	0
PLEKHM1	5	1	0.317323641	3.406489371	OK	0	113.0244293	0.114640891	0
PLIN3	5	1	1.379776642	4.410368918	OK	0	177.4883491	0.180039894	0
PLSCR1	5	1	1.58724703	3.427526472	OK	0	141.7868003	0.143821853	0
PORCN	5	1	1.569070176	4.420709608	OK	0	63.14920094	0.064041155	0
PPDPF	5	1	2.374847547	3.99579191	OK	0	187.5298512	0.190219661	0
PPP1R14B-AS1	5	1	1.82748957	2.408815859	OK	0	171.1926783	0.173650777	0
PRDM1	5	1	-0.24970816	2.77958679	OK	0	346.0109725	0.350989948	0
PRSS22	5	1	0.868888974	2.496704577	OK	0	284.1912347	0.288282608	0
PRSS27	5	1	1.380202786	2.348289965	OK	0	644.7511215	0.654044182	0
PRSS8	5	1	0.802625098	3.794003485	OK	0	116.5088726	0.118178879	0
PSCA	5	1	2.35007991	4.001967429	OK	0	578.3721199	0.586676511	0
PTK6	5	1	0.200677291	3.203274248	OK	0	341.5986134	0.34651898	0
PWWP2B	5	1	1.05733645	3.46356058	OK	0	82.89897147	0.084080905	0
RAB5IF	5	1	2.120733157	2.949410914	OK	0	356.1051098	0.361236098	0
RAB9A	5	1	2.194156662	4.534153937	OK	0	166.2163694	0.168604631	0
RANBP9	5	1	-0.242359623	3.762839316	OK	0	202.9141436	0.205831249	0
RFK	5	1	1.717212037	4.748086928	OK	0	120.8421224	0.122572507	0
RHOF	5	1	1.45730348	3.943708895	OK	0	222.8249627	0.225977392	0
RNF223	5	1	1.050098166	4.393172263	OK	0	340.3687653	0.345251503	0
RNF39	5	1	1.39312096	4.611401556	OK	0	119.0689491	0.120768961	0
S100P	5	1	0.62118359	2.987615584	OK	0	484.7854691	0.491767629	0
SAMD9	5	1	0.108711258	3.801657198	OK	0	311.5740276	0.31606273	0
SAMD9L	5	1	0.061001495	3.980484007	OK	0	49.42289159	0.050122058	0
SAT1	5	1	1.85485901	2.707714079	OK	0	187.8987578	0.190598692	0
SCEL	5	1	-0.199991807	3.73363447	OK	0	581.4354464	0.589822953	0
SCNN1A	5	1	-0.006657585	3.139955996	OK	0	258.2221932	0.261938844	0
SDCBP2	5	1	2.066227571	3.755651472	OK	0	422.0514287	0.42813532	0
SDHA	5	1	1.821547762	3.494743822	OK	0	136.0052107	0.137957321	0

SEC24C	5	1	0.610689596	3.386854647	OK	0	88.05993781	0.089317055	0
SERINC2	5	1	1.802680031	3.99084091	OK	0	193.086298	0.19586232	0
SERPINB13	5	1	-0.932775601	2.497323988	OK	0	412.3513152	0.418295411	0
SGTA	5	1	1.079403967	3.865090369	OK	0	71.36433426	0.07238135	0
SH3BGRL2	5	1	0.390353308	4.000652788	OK	0	472.1057588	0.47890126	0
SLC16A3	5	1	1.170060769	3.080048083	OK	0	192.9056967	0.195649119	0
SLC25A23	5	1	0.346444026	3.508048056	OK	0	179.6251488	0.182207074	0
SLC9A3R2	5	1	2.348631397	4.316080092	OK	0	84.21810082	0.085418461	0
SLK	5	1	-0.810439929	3.252817629	OK	0	321.1278672	0.325754644	0
SLURP2	5	1	1.894557492	2.564851759	OK	0	602.1832332	0.610864315	0
SMIM22	5	1	1.180316732	2.798590659	OK	0	84.16330774	0.085354059	0
SNCG	5	1	2.554073349	3.849249838	OK	0	289.5755367	0.293724234	0
SNX24	5	1	2.420009032	3.822438238	OK	0	207.5905829	0.210575588	0
SNX8	5	1	1.358911381	3.326991556	OK	0	97.14405305	0.098527642	0
SORBS2	5	1	0.78365779	2.884259699	OK	0	61.31761275	0.062185583	0
SORT1	5	1	-0.33216761	2.948586939	OK	0	431.0424572	0.437252073	0
SPAG1	5	1	0.9319648	3.899994372	OK	0	231.9243405	0.235246447	0
SPECC1	5	1	1.182206169	4.404376028	OK	0	215.8631206	0.218954924	0
SPINT1	5	1	1.243958578	3.191831587	OK	0	258.9356485	0.262663718	0
SPNS2	5	1	1.044174031	4.720156668	OK	0	761.2272175	0.772106556	0
SPRR2B	5	1	0.264666096	2.428786276	OK	0	93.96744768	0.095104836	0
SPSB3	5	1	2.275676624	4.12418413	OK	0	134.1963623	0.136121752	0
SRGAP1	5	1	0.717616752	3.138111113	OK	0	162.3382386	0.164656515	0
SRSF5	5	1	1.515917257	3.191328524	OK	0	263.4900155	0.267283833	0
STK39	5	1	-0.456921324	2.962276934	OK	0	272.3405255	0.276259933	0
STRADB	5	1	1.968377606	4.183063505	OK	0	105.2190489	0.106720792	0
SYAP1	5	1	-0.463487014	3.9830842	OK	0	161.8119811	0.164137067	0
TAX1BP1	5	1	-0.754978403	3.994060992	OK	0	344.2932369	0.349254213	0
TGFA	5	1	0.100980893	3.936361788	OK	0	102.6043849	0.104069087	0
TGFB1	5	1	0.698172406	2.553980349	OK	0	130.7056169	0.132577501	0
TIMP2	5	1	2.079420344	4.107531546	OK	0	764.7726239	0.775709043	0
TJP1	5	1	-0.877652987	3.114617823	OK	0	339.8626254	0.344760063	0
TJP3	5	1	0.947804645	2.869461058	OK	0	221.3523421	0.224531575	0
TMEM127	5	1	0.665525303	3.561129568	OK	0	103.4900656	0.104970329	0
TMEM40	5	1	2.52876021	3.960895537	OK	0	485.0987622	0.492094374	0
TMEM8A	5	1	1.090903164	3.311614512	OK	0	104.2874945	0.105774874	0
TMPRSS11D	5	1	-0.065057739	2.814024447	OK	0	302.1537244	0.306502928	0
TMPRSS11E	5	1	0.417922453	3.994251726	OK	0	634.0384728	0.643170651	0
TMPRSS2	5	1	0.472112701	4.025514124	OK	0	389.1246995	0.394696112	0
TP53INP2	5	1	0.531973884	3.888635634	OK	0	304.4155496	0.30877336	0
TRIP10	5	1	0.53034626	3.708186625	OK	0	328.0358406	0.332760368	0
TSPAN6	5	1	1.738747076	2.806931017	OK	0	263.4586299	0.267250206	0
TTC22	5	1	0.462346986	3.264754771	OK	0	177.7604196	0.180313225	0
TTC9	5	1	0.878832143	3.969416617	OK	0	435.4616317	0.441737488	0
TTYH1	5	1	1.15091236	3.766235827	OK	0	344.0595602	0.348940264	0
UACA	5	1	-0.847637757	3.453666209	OK	0	191.9155807	0.19467488	0
UBE2D1	5	1	2.084017888	3.717722891	OK	0	123.5960563	0.125368031	0
UBL3	5	1	-0.261745318	3.669373511	OK	0	216.1318563	0.219240844	0
UPK3B	5	1	2.369172469	3.070439814	OK	0	434.2684569	0.440491682	0
VASN	5	1	0.761317164	3.646884917	OK	0	306.1249153	0.310485614	0
VSIG10	5	1	1.646806256	3.88475561	OK	0	101.6243571	0.103075594	0
VSIG10L	5	1	0.53320779	3.115911959	OK	0	408.1557926	0.41402315	0
VSIG2	5	1	2.261975423	3.558541773	OK	0	609.2245601	0.617911764	0
WDR26	5	1	-0.372366771	3.645667551	OK	0	158.0215038	0.160290493	0
WDR45B	5	1	-0.684584841	3.321651457	OK	0	179.6375217	0.182219819	0
YPEL3	5	1	2.103712932	4.032507895	OK	0	169.9387242	0.172381005	0
ZFAND2A	5	1	2.303498164	4.45271015	OK	7.89E-303	37.17746599	0.037701234	8.00E-303
ZFAND5	5	1	0.997222923	4.390746115	OK	0	220.1789921	0.223347295	0
ZNF101	5	1	2.071026937	4.206876753	OK	0	91.93897505	0.09324362	0
ZNF185	5	1	-0.110329791	3.2754755	OK	0	389.9671791	0.395588249	0
ZNRF1	5	1	2.361412541	4.017040251	OK	0	218.7315085	0.221878561	0
AC023157.3	6	1	0.654476822	5.484818457	OK	0	104.1856252	0.105675341	0
ACOX1	6	1	-0.457640632	4.568180083	OK	0	217.4989344	0.220627276	0
ADIPOR2	6	1	0.631731526	4.765007017	OK	0	179.564683	0.182144731	0
AMOTL2	6	1	-0.269193395	4.415825842	OK	0	69.07158248	0.070054999	0
ANKRD13A	6	1	-0.168756589	4.462661742	OK	0	113.9198441	0.115551767	0
ANKRD37	6	1	0.947839931	5.604867934	OK	0	167.7151696	0.170125335	0
ARHGAP10	6	1	0.234524802	4.402782439	OK	0	207.7814854	0.210769071	0
ARL4C	6	1	0.452182606	4.74882984	OK	0	180.6920978	0.183289042	0
ASCC2	6	1	0.639093847	5.425029753	OK	0	123.0272127	0.124790831	0
ATG9B	6	1	0.574757979	4.585546492	OK	0	537.9230503	0.54557172	0
BPGM	6	1	0.162322596	5.505266188	OK	0	48.0966126	0.04877735	0
C18orf25	6	1	0.468652204	5.148887633	OK	0	201.5397077	0.20443287	0

CDKL5	6	1	-0.206181392	4.554294585	OK	0	91.96738186	0.093274562	0
CFLAR	6	1	0.583018587	5.199415205	OK	0	142.281627	0.144324214	0
CGNL1	6	1	0.820018557	4.883240698	OK	0	545.4976824	0.553241585	0
CHAC1	6	1	-0.014312013	5.097069739	OK	0	280.486403	0.284307749	0
CITED2	6	1	0.685852543	5.244547842	OK	0	129.654138	0.131513978	0
CNST	6	1	0.580457882	5.115193365	OK	0	117.2043621	0.118878763	0
CSNK1E	6	1	0.114267126	4.935920714	OK	0	129.6477081	0.131505603	0
DKK1	6	1	0.659969733	5.518457411	OK	0	336.7215333	0.341410539	0
DUSP14	6	1	0.223446146	5.654047964	OK	0	102.7899874	0.104260715	0
DUSP5	6	1	0.543381975	5.326819418	OK	0	331.9651945	0.3367381	0
ERO1A	6	1	0.795767189	4.576729773	OK	0	347.3011844	0.352302382	0
FAM214A	6	1	0.253162519	4.863839148	OK	0	191.4079091	0.19415462	0
FLG	6	1	0.212209538	5.657109259	OK	0	325.6668067	0.330272629	0
GNE	6	1	-0.184518918	4.501211165	OK	0	98.67681552	0.100074713	0
GPAT3	6	1	-0.100526735	4.944177626	OK	0	312.9460905	0.317342511	0
GRPEL2	6	1	0.849469234	5.27301216	OK	0	311.9734578	0.31644364	0
HCG22	6	1	0.667226777	5.208042143	OK	0	389.00955	0.394543253	0
HILPDA	6	1	0.878430366	5.50162792	OK	0	250.1113345	0.253702216	0
IL1RN	6	1	0.583720103	5.044149397	OK	0	450.6357138	0.457133021	0
ITGB8	6	1	-0.114590689	4.204757689	OK	0	136.1836871	0.138137535	0
KPRP	6	1	0.714850188	5.559823988	OK	0	276.705257	0.280506374	0
KRT16	6	1	-0.253499016	5.099515913	OK	0	250.291273	0.253894899	0
KRTAP3-2	6	1	1.176404372	6.712679861	OK	0	183.2759151	0.185606821	0
LNX1	6	1	0.656699971	4.78874111	OK	0	185.9705633	0.188637032	0
LPIN1	6	1	0.496360198	4.964568137	OK	0	466.4110305	0.473084434	0
MACO1	6	1	0.359179393	4.905659674	OK	0	114.9008094	0.116546607	0
MCU	6	1	0.355279998	4.623193739	OK	0	116.4249278	0.118089442	0
MT1A	6	1	1.2033294	6.768424033	OK	0	76.09390949	0.076930472	0
MT1E	6	1	1.213833824	6.769074438	OK	0	390.9570623	0.39659257	0
MT1F	6	1	1.210290865	6.774556158	OK	0	72.21828667	0.073239868	0
MT1G	6	1	1.228782699	6.795895575	OK	0	285.1050767	0.289200487	0
MT1H	6	1	1.194084392	6.75208473	OK	0	279.0143532	0.282987201	0
MT1M	6	1	1.237856672	6.802793501	OK	0	161.0983657	0.163389607	0
MT2A	6	1	1.250069276	6.821645735	OK	0	466.5079858	0.473235012	0
MYZAP	6	1	0.263877705	4.836771963	OK	0	320.8284676	0.325419829	0
NABP1	6	1	0.434566007	5.238608359	OK	0	127.0786783	0.128899799	0
NEDD4L	6	1	-0.077365383	4.443242071	OK	0	82.95396745	0.084136604	0
NR1D1	6	1	1.081408009	6.453282355	OK	0	96.46358663	0.097840897	0
OXSR1	6	1	-0.255462631	4.587451933	OK	0	137.6766898	0.139651895	0
PADI1	6	1	0.139825896	4.762464522	OK	0	435.3303383	0.441577366	0
PARD6B	6	1	-0.106402441	4.568169592	OK	0	138.7384022	0.140726382	0
PDP1	6	1	-0.109326586	4.360187529	OK	0	65.35033446	0.066279627	0
PDZD2	6	1	-0.122440203	4.343046187	OK	0	78.1201569	0.079233321	0
PELI1	6	1	-0.107826933	4.880102156	OK	0	150.4246262	0.152582908	0
PHACTR2	6	1	-0.07844673	4.995727537	OK	0	203.3841078	0.206309095	0
PPL	6	1	-0.50208877	4.450412749	OK	0	426.5544047	0.432704026	0
PTPN3	6	1	-0.28791736	4.718440054	OK	0	81.25068811	0.082409659	0
QSOX1	6	1	0.291849331	5.440801619	OK	0	294.215965	0.298448385	0
RASGEF1B	6	1	0.600836412	5.473721503	OK	0	135.6035204	0.137538862	0
RIOK3	6	1	-0.624993786	4.121131895	OK	0	330.2011143	0.334958176	0
RMND5A	6	1	0.270897881	4.999139784	OK	0	151.8914784	0.154071822	0
RNASE7	6	1	0.201578096	5.575141905	OK	0	602.393195	0.610915876	0
RNF169	6	1	0.384669051	4.67060089	OK	0	131.59774	0.133484666	0
SASH1	6	1	-0.40657626	4.25782585	OK	0	231.7463291	0.235081072	0
SEC14L1	6	1	0.381332771	4.559777258	OK	0	122.4094454	0.124162302	0
SECISBP2L	6	1	-0.364160165	4.571111677	OK	0	174.6378579	0.177145133	0
SESN2	6	1	0.709717736	5.434245108	OK	0	176.021871	0.178533925	0
SH3D19	6	1	-0.311401709	4.144282339	OK	0	99.98079513	0.101410929	0
SLC20A2	6	1	-0.288973316	4.951071738	OK	0	112.7618792	0.114377453	0
SNX9	6	1	0.589619414	5.014683722	OK	0	213.368937	0.216436584	0
SYNPO2L	6	1	0.628093824	5.540040968	OK	0	228.9929963	0.232065685	0
TCP11L2	6	1	0.793558941	4.927032469	OK	0	275.0539928	0.279000131	0
TEAD1	6	1	-0.364682301	4.406012534	OK	0	137.5743625	0.139547382	0
TICAM1	6	1	0.624626265	4.888031004	OK	0	157.9724732	0.160236809	0
TMOD3	6	1	-0.642777904	4.180425642	OK	0	357.3484758	0.362497716	0
TNIP1	6	1	-0.398778781	4.572251318	OK	0	186.0050496	0.188679079	0
TP53BP2	6	1	0.026254193	4.778003691	OK	0	106.268608	0.107789312	0
TRIM2	6	1	0.020363644	4.501749991	OK	0	176.6294491	0.179154483	0
TUFT1	6	1	-0.253424629	4.955034254	OK	0	107.4780284	0.109015751	0
UBE2G1	6	1	0.755913109	4.739602087	OK	0	158.3813099	0.160656493	0
UPP1	6	1	0.987654746	5.478302	OK	0	276.4968601	0.280478008	0
USP53	6	1	-0.294295415	4.198180197	OK	0	110.5725745	0.112156381	0
USP6NL	6	1	-0.140359505	4.187330244	OK	0	222.892655	0.226095583	0

VPS37B	6	1	-0.272229537	4.806070326	OK	0	195.324401	0.198132008	0
WWC1	6	1	-0.359070047	4.546818732	OK	0	144.8576233	0.146932437	0
YOD1	6	1	0.466948107	5.435899733	OK	0	269.6401227	0.273509689	0
ZNF365	6	1	0.819224112	5.448735236	OK	0	540.6312193	0.548271657	0
ZNF426	6	1	0.161319927	5.103723524	OK	0	153.103905	0.155296108	0
ZNF430	6	1	0.2264985	5.644870756	OK	0	64.31879868	0.065234428	0
ZNF431	6	1	0.287737385	5.576566695	OK	0	177.384566	0.179932189	0
ZNF714	6	1	0.213167504	5.632364271	OK	0	66.0553414	0.066993126	0
ZNF92	6	1	0.212481812	5.639434813	OK	0	78.66965378	0.079788	0
ALOX15	7	1	-0.328905209	-0.238170149	OK	0	434.6651069	0.440929562	0
ANO1	7	1	-0.142525061	-0.502690317	OK	0	320.4936555	0.325110881	0
APOL1	7	1	-0.09544526	-1.116366388	OK	0	136.483189	0.138440431	0
ARNTL2	7	1	-0.198494777	-0.438445808	OK	0	264.2569026	0.268061191	0
ATP13A5	7	1	-0.51693128	-0.850231887	OK	0	130.64077	0.132510822	0
ATP8B1	7	1	0.089923278	-0.575456621	OK	0	230.211679	0.233524191	0
BCL2L15	7	1	-0.499163374	-0.511592628	OK	0	219.2263121	0.222379404	0
C12orf75	7	1	-0.566905841	-1.349287273	OK	0	160.5192575	0.162824817	0
CA2	7	1	-0.109168097	-1.401830198	OK	0	236.2899464	0.239690891	0
CADM1	7	1	-0.27633129	-0.56154895	OK	0	118.3391683	0.120032773	0
CAMK1D	7	1	-0.076294109	-0.479494096	OK	0	217.4202527	0.220546531	0
CCL26	7	1	-0.31754516	-0.543848993	OK	0	302.0231403	0.306374111	0
CDH3	7	1	-0.308561667	-1.404706718	OK	0	180.9037785	0.183503227	0
CES2	7	1	0.447689608	-0.480618717	OK	0	470.9342532	0.477725055	0
CHL1	7	1	-0.380515679	-0.414757253	OK	0	237.1249703	0.240534647	0
CISH	7	1	-0.40716122	-1.215509893	OK	0	69.58338323	0.070574812	0
CLDN1	7	1	0.034546808	-0.951485159	OK	0	201.4444818	0.204342031	0
CTSC	7	1	-0.546894416	-1.069386961	OK	0	293.4937114	0.297720553	0
CYP2S1	7	1	-0.078554555	-0.884851934	OK	0	150.6675981	0.152829389	0
CYP7B1	7	1	-0.493104681	-0.696304084	OK	0	294.3818033	0.298622115	0
DPYD	7	1	-0.291733369	-0.408997537	OK	0	253.6263682	0.257276923	0
DSG3	7	1	0.633452803	-0.60170603	OK	0	372.6045303	0.37797198	0
DSP	7	1	0.566945211	-0.727092745	OK	0	236.2433509	0.239635524	0
DUOX1	7	1	0.201838032	-0.241605522	OK	0	326.2683787	0.330969373	0
EPPK1	7	1	-0.343518361	-0.374264957	OK	0	266.1956611	0.270023892	0
GALNT5	7	1	-0.068886503	-0.770165922	OK	0	214.1991008	0.217279884	0
HAS3	7	1	-0.102325662	-0.901779653	OK	0	143.95017	0.146014356	0
HLF	7	1	-0.349716767	-1.28568411	OK	0	174.4748259	0.176981969	0
HPGD	7	1	-0.614512786	-0.868858816	OK	0	354.9179683	0.360032332	0
KCNJ2	7	1	-0.456494793	-1.242721321	OK	0	130.4389326	0.132310141	0
LINC01497	7	1	-0.586439356	-1.015957834	OK	0	193.505593	0.196287013	0
LTA4H	7	1	0.050769643	-0.249021293	OK	0	348.1073431	0.353123521	0
MDK	7	1	-0.549268469	-1.18397999	OK	0	188.5971158	0.191309067	0
MPP7	7	1	0.160970286	-0.526329757	OK	0	364.668985	0.369924725	0
MX1	7	1	0.046813623	-0.70668626	OK	0	268.5388615	0.272405691	0
MYH9	7	1	0.247705177	-0.853569986	OK	0	257.7729451	0.261484334	0
NEFL	7	1	-0.290648207	-0.313233139	OK	0	177.4029801	0.179948359	0
NTRK2	7	1	-0.4455484	-1.274094106	OK	0	213.3491183	0.216418684	0
P2RY1	7	1	0.238220826	-0.610698225	OK	0	239.3458131	0.242790812	0
PDZK1IP1	7	1	-0.332458004	0.007324456	OK	0	292.720704	0.29693664	0
PHLDB2	7	1	-0.070421621	-1.448131801	OK	0	99.34597926	0.100765568	0
PMCH	7	1	-0.593682631	-0.713761808	OK	0	68.39893734	0.069362999	0
SAMHD1	7	1	-0.542641743	-0.719862463	OK	0	227.8986842	0.231176789	0
SCIN	7	1	-0.492592796	-0.708209755	OK	0	469.4131307	0.476182218	0
SECTM1	7	1	-0.249639257	-0.448442222	OK	0	136.333547	0.138286551	0
SERPINB3	7	1	-0.680836901	-0.759157659	OK	0	480.0374551	0.486955965	0
SERPINB4	7	1	-0.633898958	-0.658208133	OK	0	367.927037	0.373230122	0
SERPINE2	7	1	-0.592544659	-0.915153267	OK	0	174.5971759	0.177105422	0
SFRP1	7	1	-0.557473763	-1.054627182	OK	0	216.6401506	0.219756248	0
SGK1	7	1	-0.502075061	-1.145987274	OK	0	252.2459678	0.25587756	0
SH3RF2	7	1	-0.077612981	-0.703767778	OK	0	244.2873354	0.247803661	0
SLC26A4-AS1	7	1	-0.39774464	-0.35791135	OK	0	218.6808982	0.221817728	0
SPARCL1	7	1	-0.477817878	-1.061317922	OK	0	78.91544755	0.080039264	0
SPTBN1	7	1	0.28666778	-0.717245819	OK	0	247.5870723	0.25115134	0
SYNPO	7	1	-0.298034295	-1.181150676	OK	0	122.6287639	0.124385346	0
TFPI	7	1	-0.345505699	-1.25912595	OK	0	66.9720114	0.067924272	0
TFRC	7	1	0.405159608	-0.763397218	OK	0	175.4500203	0.177971262	0
THBD	7	1	0.039195374	-1.084187748	OK	0	199.7149552	0.202586275	0
TMPRSS11A	7	1	0.105847434	-0.249421121	OK	0	361.7085279	0.36692124	0
TNFAIP6	7	1	-0.588265404	-0.733052971	OK	0	208.3303277	0.211322126	0
ZFP36L1	7	1	0.786903456	-0.87909341	OK	0	378.064425	0.383510027	0

Supplemental Table 4. Gene expression profile of module 7 genes in EoE vs healthy subjects.

gene	meanEoE	meanHC	log2FC	status	p_value	morans_test_statistic	morans_I	q_value
TNFAIP6	0.966075106	3.85E-05	14.61634424	OK	0	208.3303277	0.211322126	0
ALOX15	1.961654088	0.0007248	11.40220098	OK	0	434.6651069	0.440929562	0
PMCH	0.202486736	0.000257418	9.619501201	OK	0	68.39893734	0.069362999	0
CCL26	6.859650624	0.016739443	8.678739727	OK	0	302.0231403	0.306374111	0
SLC26A4-AS1	0.120048362	0.000586635	7.676937321	OK	0	218.6808982	0.221817728	0
ATP13A5	0.15791579	0.001201737	7.037890216	OK	0	130.64077	0.132510822	0
ANO1	0.836764412	0.025489408	5.03685174	OK	0	320.4936555	0.325110881	0
LINC01497	0.661007481	0.02831416	4.545070971	OK	0	193.505593	0.196287013	0
EPPK1	0.26539871	0.01543172	4.104190539	OK	0	266.1956611	0.270023892	0
CA2	1.371843303	0.080025119	4.099518982	OK	0	236.2899464	0.239690891	0
SERPINB4	6.673203074	0.427245606	3.965241846	OK	0	367.927037	0.373230122	0
NEFL	0.280173501	0.018845725	3.894011331	OK	0	177.4029801	0.179948359	0
SYNPO	0.235892522	0.016821057	3.809789454	OK	0	122.6287639	0.124385346	0
SCIN	3.688673568	0.275632567	3.742283858	OK	0	469.4131307	0.476182218	0
BCL2L15	0.386922308	0.030522998	3.664075337	OK	0	219.2263121	0.222379404	0
SFRP1	0.617311839	0.057586647	3.422193231	OK	0	216.6401506	0.219756248	0
CTSC	3.435824221	0.360297523	3.253395592	OK	0	293.4937114	0.297720553	0
CYP7B1	0.957796831	0.114809808	3.06047377	OK	0	294.3818033	0.298622115	0
KCNJ2	0.58025804	0.073125635	2.988245461	OK	0	130.4389326	0.132310141	0
SGK1	2.223951321	0.316300363	2.813758096	OK	0	252.2459678	0.25587756	0
MDK	0.882211769	0.13098338	2.751741244	OK	0	188.5971158	0.191309067	0
NTRK2	0.94801279	0.143535812	2.723495785	OK	0	213.3491183	0.216418684	0
CISH	0.225675308	0.035958991	2.649824121	OK	0	69.58338323	0.070574812	0
CADM1	0.144856241	0.024142202	2.584992696	OK	0	118.3391683	0.120032773	0
HAS3	0.200475746	0.034917142	2.52142031	OK	0	143.95017	0.146014356	0
SECTM1	0.169999339	0.031296673	2.441447924	OK	0	136.333547	0.138286551	0
PHLDB2	0.153398438	0.02979627	2.364080168	OK	0	99.34597926	0.100765568	0
APOL1	0.285596061	0.056620866	2.334570374	OK	0	136.483189	0.138440431	0
CHL1	0.338550396	0.071509407	2.243165654	OK	0	237.1249703	0.240534647	0
DPYD	0.493134501	0.106768169	2.207499592	OK	0	253.6263682	0.257276923	0
DUOX1	1.171431477	0.253832096	2.206326155	OK	0	326.2683787	0.330969373	0
PDZK1IP1	3.354050846	0.74706284	2.166603052	OK	0	292.720704	0.29693664	0
CAMK1D	0.299012821	0.06723627	2.152895754	OK	0	217.4202527	0.220546531	0
SH3RF2	0.653008701	0.161890923	2.012080117	OK	0	244.2873354	0.247803661	0
C12orf75	0.453379065	0.112809698	2.006826682	OK	0	160.5192575	0.162824817	0
GALNT5	0.476173378	0.130597042	1.866364744	OK	0	214.1991008	0.217279884	0
SAMHD1	0.346029726	0.096709186	1.839171147	OK	0	227.8986842	0.231176789	0
LTA4H	1.996930207	0.570936273	1.806382283	OK	0	348.1073431	0.353123521	0
MPP7	1.046935282	0.307842937	1.765905889	OK	0	364.668985	0.369924725	0
SERPINB3	17.48953192	5.324917426	1.71566062	OK	0	480.0374551	0.486955965	0
SERPINE2	0.356731454	0.108896618	1.711879278	OK	0	174.5971759	0.177105422	0
MX1	0.797845799	0.246847702	1.692488724	OK	0	268.5388615	0.272405691	0
MYH9	1.216837583	0.379623725	1.680494553	OK	0	257.7729451	0.261484334	0
ATP8B1	0.488132901	0.158051825	1.626876298	OK	0	230.211679	0.233524191	0
P2RY1	0.656319749	0.21307677	1.623025526	OK	0	239.3458131	0.242790812	0
SPTBN1	0.839628846	0.273834653	1.616446703	OK	0	247.5870723	0.25115134	0
CDH3	0.359101344	0.11811826	1.60415904	OK	0	180.9037785	0.183503227	0
THBD	0.404966324	0.134632233	1.588778084	OK	0	199.7149552	0.202586275	0
HPGD	3.934350587	1.313569137	1.582633386	OK	0	354.9179683	0.360032332	0
TFPI	0.120224359	0.041967708	1.51837767	OK	0	66.9720114	0.067924272	0
HLF	0.498670622	0.178091332	1.485469908	OK	0	174.4748259	0.176981969	0
CLDN1	0.710253872	0.256735722	1.468050745	OK	0	201.4444818	0.204342031	0
SPARCL1	0.13990251	0.054755911	1.353335215	OK	0	78.91544755	0.080039264	0
CYP2S1	0.24700796	0.099058602	1.318203369	OK	0	150.6675981	0.152829389	0
TMPRSS11A	0.897482841	0.377530588	1.249290817	OK	0	361.7085279	0.36692124	0
CES2	2.138066605	0.953794755	1.164556043	OK	0	470.9342532	0.477725055	0
DSG3	4.166980771	1.902058482	1.131440838	OK	0	372.6045303	0.37797198	0
ARNTL2	0.553917381	0.277874538	0.995237163	OK	0	264.2569026	0.268061191	0

DSP	15.0345425	7.578231045	0.988347937	OK	0	236.2433509	0.239635524	0
TFRC	0.392033289	0.209336069	0.90515525	OK	0	175.4500203	0.177971262	0
ZFP36L1	5.059103183	2.971624788	0.767629698	OK	0	378.064425	0.383510027	0

Supplemental Table 5. Identification of SOX2 and/or KLF5 downstream targets from Enrichr databases and literature-based analyses.

gene	source	regulator
CACNA2D4	Enrichr CHEA_2022: SOX2 20726797 ChIP-Seq SW620 Human	SOX2
KIAA1586	Enrichr CHEA_2022: SOX2 20726797 ChIP-Seq SW620 Human	SOX2
ISL1	Enrichr CHEA_2022: SOX2 20726797 ChIP-Seq SW620 Human	SOX2
LARP6	Enrichr CHEA_2022: SOX2 20726797 ChIP-Seq SW620 Human	SOX2
L1CAM	Enrichr CHEA_2022: SOX2 20726797 ChIP-Seq SW620 Human	SOX2
SPINT2	Enrichr CHEA_2022: SOX2 20726797 ChIP-Seq SW620 Human	SOX2
ASPHD1	Enrichr CHEA_2022: SOX2 20726797 ChIP-Seq SW620 Human	SOX2
SPINT1	Enrichr CHEA_2022: SOX2 20726797 ChIP-Seq SW620 Human	SOX2
FEZ2	Enrichr CHEA_2022: SOX2 20726797 ChIP-Seq SW620 Human	SOX2
SCO1	Enrichr CHEA_2022: SOX2 20726797 ChIP-Seq SW620 Human	SOX2
ATF3	Enrichr CHEA_2022: SOX2 20726797 ChIP-Seq SW620 Human	SOX2
SAMD9	Enrichr CHEA_2022: SOX2 20726797 ChIP-Seq SW620 Human	SOX2
HPGD	Enrichr CHEA_2022: SOX2 20726797 ChIP-Seq SW620 Human	SOX2
PON3	Enrichr CHEA_2022: SOX2 20726797 ChIP-Seq SW620 Human	SOX2
CRABP2	Enrichr CHEA_2022: SOX2 20726797 ChIP-Seq SW620 Human	SOX2
TFPI	Enrichr CHEA_2022: SOX2 20726797 ChIP-Seq SW620 Human	SOX2
UACA	Enrichr CHEA_2022: SOX2 20726797 ChIP-Seq SW620 Human	SOX2
SPDEF	Enrichr CHEA_2022: SOX2 20726797 ChIP-Seq SW620 Human	SOX2
TFPT	Enrichr CHEA_2022: SOX2 20726797 ChIP-Seq SW620 Human	SOX2
DPYSL3	Enrichr CHEA_2022: SOX2 20726797 ChIP-Seq SW620 Human	SOX2
ZNF19	Enrichr CHEA_2022: SOX2 20726797 ChIP-Seq SW620 Human	SOX2
JAG1	Enrichr CHEA_2022: SOX2 20726797 ChIP-Seq SW620 Human	SOX2
NEBL	Enrichr CHEA_2022: SOX2 20726797 ChIP-Seq SW620 Human	SOX2
ATP6AP2	Enrichr CHEA_2022: SOX2 20726797 ChIP-Seq SW620 Human	SOX2
ATP6AP1	Enrichr CHEA_2022: SOX2 20726797 ChIP-Seq SW620 Human	SOX2
NR2F2	Enrichr CHEA_2022: SOX2 20726797 ChIP-Seq SW620 Human	SOX2
PBX3	Enrichr CHEA_2022: SOX2 20726797 ChIP-Seq SW620 Human	SOX2
VPS13A	Enrichr CHEA_2022: SOX2 20726797 ChIP-Seq SW620 Human	SOX2
SUPV3L1	Enrichr CHEA_2022: SOX2 20726797 ChIP-Seq SW620 Human	SOX2
VANGL1	Enrichr CHEA_2022: SOX2 20726797 ChIP-Seq SW620 Human	SOX2
BCR	Enrichr CHEA_2022: SOX2 20726797 ChIP-Seq SW620 Human	SOX2
PHF20L1	Enrichr CHEA_2022: SOX2 20726797 ChIP-Seq SW620 Human	SOX2
PPFIBP2	Enrichr CHEA_2022: SOX2 20726797 ChIP-Seq SW620 Human	SOX2
FAM111A	Enrichr CHEA_2022: SOX2 20726797 ChIP-Seq SW620 Human	SOX2
PLSCR1	Enrichr CHEA_2022: SOX2 20726797 ChIP-Seq SW620 Human	SOX2
PKIA	Enrichr CHEA_2022: SOX2 20726797 ChIP-Seq SW620 Human	SOX2
SFXN3	Enrichr CHEA_2022: SOX2 20726797 ChIP-Seq SW620 Human	SOX2
CHST5	Enrichr CHEA_2022: SOX2 20726797 ChIP-Seq SW620 Human	SOX2
PKIG	Enrichr CHEA_2022: SOX2 20726797 ChIP-Seq SW620 Human	SOX2
ATF4	Enrichr CHEA_2022: SOX2 20726797 ChIP-Seq SW620 Human	SOX2
ATF5	Enrichr CHEA_2022: SOX2 20726797 ChIP-Seq SW620 Human	SOX2
CHST4	Enrichr CHEA_2022: SOX2 20726797 ChIP-Seq SW620 Human	SOX2
CDCA2	Enrichr CHEA_2022: SOX2 20726797 ChIP-Seq SW620 Human	SOX2
PDGFC	Enrichr CHEA_2022: SOX2 20726797 ChIP-Seq SW620 Human	SOX2
ZNF22	Enrichr CHEA_2022: SOX2 20726797 ChIP-Seq SW620 Human	SOX2
GATA6	Enrichr CHEA_2022: SOX2 20726797 ChIP-Seq SW620 Human	SOX2
PDGFA	Enrichr CHEA_2022: SOX2 20726797 ChIP-Seq SW620 Human	SOX2
F11R	Enrichr CHEA_2022: SOX2 20726797 ChIP-Seq SW620 Human	SOX2
GATA3	Enrichr CHEA_2022: SOX2 20726797 ChIP-Seq SW620 Human	SOX2
ALKBH8	Enrichr CHEA_2022: SOX2 20726797 ChIP-Seq SW620 Human	SOX2
GATA2	Enrichr CHEA_2022: SOX2 20726797 ChIP-Seq SW620 Human	SOX2
ZNF26	Enrichr CHEA_2022: SOX2 20726797 ChIP-Seq SW620 Human	SOX2
ALKBH6	Enrichr CHEA_2022: SOX2 20726797 ChIP-Seq SW620 Human	SOX2
EPB41L1	Enrichr CHEA_2022: SOX2 20726797 ChIP-Seq SW620 Human	SOX2
ZC3H12A	Enrichr CHEA_2022: SOX2 20726797 ChIP-Seq SW620 Human	SOX2

EPB41L2	Enrichr CHEA_2022: SOX2 20726797 ChIP-Seq SW620 Human	SOX2
FAM110C	Enrichr CHEA_2022: SOX2 20726797 ChIP-Seq SW620 Human	SOX2
NKX3-1	Enrichr CHEA_2022: SOX2 20726797 ChIP-Seq SW620 Human	SOX2
PIP5K1B	Enrichr CHEA_2022: SOX2 20726797 ChIP-Seq SW620 Human	SOX2
PRKACB	Enrichr CHEA_2022: SOX2 20726797 ChIP-Seq SW620 Human	SOX2
ZNF488	Enrichr CHEA_2022: SOX2 20726797 ChIP-Seq SW620 Human	SOX2
DUSP5	Enrichr CHEA_2022: SOX2 20726797 ChIP-Seq SW620 Human	SOX2
FUCA1	Enrichr CHEA_2022: SOX2 20726797 ChIP-Seq SW620 Human	SOX2
JUN	Enrichr CHEA_2022: SOX2 20726797 ChIP-Seq SW620 Human	SOX2
DUSP4	Enrichr CHEA_2022: SOX2 20726797 ChIP-Seq SW620 Human	SOX2
JUP	Enrichr CHEA_2022: SOX2 20726797 ChIP-Seq SW620 Human	SOX2
FN1	Enrichr CHEA_2022: SOX2 20726797 ChIP-Seq SW620 Human	SOX2
DCBLD2	Enrichr CHEA_2022: SOX2 20726797 ChIP-Seq SW620 Human	SOX2
TMEFF1	Enrichr CHEA_2022: SOX2 20726797 ChIP-Seq SW620 Human	SOX2
SLC7A5	Enrichr CHEA_2022: SOX2 20726797 ChIP-Seq SW620 Human	SOX2
CENPF	Enrichr CHEA_2022: SOX2 20726797 ChIP-Seq SW620 Human	SOX2
SLC7A7	Enrichr CHEA_2022: SOX2 20726797 ChIP-Seq SW620 Human	SOX2
SLC7A8	Enrichr CHEA_2022: SOX2 20726797 ChIP-Seq SW620 Human	SOX2
DPYD	Enrichr CHEA_2022: SOX2 20726797 ChIP-Seq SW620 Human	SOX2
FXYD6	Enrichr CHEA_2022: SOX2 20726797 ChIP-Seq SW620 Human	SOX2
RAPGEF4	Enrichr CHEA_2022: SOX2 20726797 ChIP-Seq SW620 Human	SOX2
RAPGEF5	Enrichr CHEA_2022: SOX2 20726797 ChIP-Seq SW620 Human	SOX2
LIMS2	Enrichr CHEA_2022: SOX2 20726797 ChIP-Seq SW620 Human	SOX2
TFCP2L1	Enrichr CHEA_2022: SOX2 20726797 ChIP-Seq SW620 Human	SOX2
ZC3HAV1	Enrichr CHEA_2022: SOX2 20726797 ChIP-Seq SW620 Human	SOX2
SLC7A1	Enrichr CHEA_2022: SOX2 20726797 ChIP-Seq SW620 Human	SOX2
HOXC13	Enrichr CHEA_2022: SOX2 20726797 ChIP-Seq SW620 Human	SOX2
SLC7A2	Enrichr CHEA_2022: SOX2 20726797 ChIP-Seq SW620 Human	SOX2
HOXC10	Enrichr CHEA_2022: SOX2 20726797 ChIP-Seq SW620 Human	SOX2
HLCS	Enrichr CHEA_2022: SOX2 20726797 ChIP-Seq SW620 Human	SOX2
SHH	Enrichr CHEA_2022: SOX2 20726797 ChIP-Seq SW620 Human	SOX2
PANX1	Enrichr CHEA_2022: SOX2 20726797 ChIP-Seq SW620 Human	SOX2
DLGAP1	Enrichr CHEA_2022: SOX2 20726797 ChIP-Seq SW620 Human	SOX2
ZNF468	Enrichr CHEA_2022: SOX2 20726797 ChIP-Seq SW620 Human	SOX2
SLC18A2	Enrichr CHEA_2022: SOX2 20726797 ChIP-Seq SW620 Human	SOX2
PCMTD2	Enrichr CHEA_2022: SOX2 20726797 ChIP-Seq SW620 Human	SOX2
EGLN2	Enrichr CHEA_2022: SOX2 20726797 ChIP-Seq SW620 Human	SOX2
ATAD2	Enrichr CHEA_2022: SOX2 20726797 ChIP-Seq SW620 Human	SOX2
CDKL5	Enrichr CHEA_2022: SOX2 20726797 ChIP-Seq SW620 Human	SOX2
PPARA	Enrichr CHEA_2022: SOX2 20726797 ChIP-Seq SW620 Human	SOX2
DGAT1	Enrichr CHEA_2022: SOX2 20726797 ChIP-Seq SW620 Human	SOX2
SNX25	Enrichr CHEA_2022: SOX2 20726797 ChIP-Seq SW620 Human	SOX2
BGN	Enrichr CHEA_2022: SOX2 20726797 ChIP-Seq SW620 Human	SOX2
ADAM10	Enrichr CHEA_2022: SOX2 20726797 ChIP-Seq SW620 Human	SOX2
SLC39A14	Enrichr CHEA_2022: SOX2 20726797 ChIP-Seq SW620 Human	SOX2
DAB2IP	Enrichr CHEA_2022: SOX2 20726797 ChIP-Seq SW620 Human	SOX2
MYBBP1A	Enrichr CHEA_2022: SOX2 20726797 ChIP-Seq SW620 Human	SOX2
ADAM17	Enrichr CHEA_2022: SOX2 20726797 ChIP-Seq SW620 Human	SOX2
LARS2	Enrichr CHEA_2022: SOX2 20726797 ChIP-Seq SW620 Human	SOX2
APEX2	Enrichr CHEA_2022: SOX2 20726797 ChIP-Seq SW620 Human	SOX2
ETNK1	Enrichr CHEA_2022: SOX2 20726797 ChIP-Seq SW620 Human	SOX2
PPARG	Enrichr CHEA_2022: SOX2 20726797 ChIP-Seq SW620 Human	SOX2
TSR1	Enrichr CHEA_2022: SOX2 20726797 ChIP-Seq SW620 Human	SOX2
LGR6	Enrichr CHEA_2022: SOX2 20726797 ChIP-Seq SW620 Human	SOX2
PPARD	Enrichr CHEA_2022: SOX2 20726797 ChIP-Seq SW620 Human	SOX2
LGR4	Enrichr CHEA_2022: SOX2 20726797 ChIP-Seq SW620 Human	SOX2
OAT	Enrichr CHEA_2022: SOX2 20726797 ChIP-Seq SW620 Human	SOX2
MTMR10	Enrichr CHEA_2022: SOX2 20726797 ChIP-Seq SW620 Human	SOX2
SNX10	Enrichr CHEA_2022: SOX2 20726797 ChIP-Seq SW620 Human	SOX2

SMPD3	Enrichr CHEA_2022: SOX2 20726797 ChIP-Seq SW620 Human	SOX2
WRN	Enrichr CHEA_2022: SOX2 20726797 ChIP-Seq SW620 Human	SOX2
FGFR1OP2	Enrichr CHEA_2022: SOX2 20726797 ChIP-Seq SW620 Human	SOX2
PLAGL1	Enrichr CHEA_2022: SOX2 20726797 ChIP-Seq SW620 Human	SOX2
AGR2	Enrichr CHEA_2022: SOX2 20726797 ChIP-Seq SW620 Human	SOX2
NUDT13	Enrichr CHEA_2022: SOX2 20726797 ChIP-Seq SW620 Human	SOX2
FADS1	Enrichr CHEA_2022: SOX2 20726797 ChIP-Seq SW620 Human	SOX2
MAP3K1	Enrichr CHEA_2022: SOX2 20726797 ChIP-Seq SW620 Human	SOX2
BIK	Enrichr CHEA_2022: SOX2 20726797 ChIP-Seq SW620 Human	SOX2
CORO2A	Enrichr CHEA_2022: SOX2 20726797 ChIP-Seq SW620 Human	SOX2
FBXO17	Enrichr CHEA_2022: SOX2 20726797 ChIP-Seq SW620 Human	SOX2
STX8	Enrichr CHEA_2022: SOX2 20726797 ChIP-Seq SW620 Human	SOX2
ATP11C	Enrichr CHEA_2022: SOX2 20726797 ChIP-Seq SW620 Human	SOX2
SDCBP2	Enrichr CHEA_2022: SOX2 20726797 ChIP-Seq SW620 Human	SOX2
WT1	Enrichr CHEA_2022: SOX2 20726797 ChIP-Seq SW620 Human	SOX2
MFNG	Enrichr CHEA_2022: SOX2 20726797 ChIP-Seq SW620 Human	SOX2
LY6E	Enrichr CHEA_2022: SOX2 20726797 ChIP-Seq SW620 Human	SOX2
PDCD4	Enrichr CHEA_2022: SOX2 20726797 ChIP-Seq SW620 Human	SOX2
PKP4	Enrichr CHEA_2022: SOX2 20726797 ChIP-Seq SW620 Human	SOX2
IL6ST	Enrichr CHEA_2022: SOX2 20726797 ChIP-Seq SW620 Human	SOX2
AGRN	Enrichr CHEA_2022: SOX2 20726797 ChIP-Seq SW620 Human	SOX2
FRK	Enrichr CHEA_2022: SOX2 20726797 ChIP-Seq SW620 Human	SOX2
ZNF430	Enrichr CHEA_2022: SOX2 20726797 ChIP-Seq SW620 Human	SOX2
NIP7	Enrichr CHEA_2022: SOX2 20726797 ChIP-Seq SW620 Human	SOX2
PDCD6	Enrichr CHEA_2022: SOX2 20726797 ChIP-Seq SW620 Human	SOX2
TSPAN31	Enrichr CHEA_2022: SOX2 20726797 ChIP-Seq SW620 Human	SOX2
ATP10B	Enrichr CHEA_2022: SOX2 20726797 ChIP-Seq SW620 Human	SOX2
PLOD2	Enrichr CHEA_2022: SOX2 20726797 ChIP-Seq SW620 Human	SOX2
FRY	Enrichr CHEA_2022: SOX2 20726797 ChIP-Seq SW620 Human	SOX2
EGFR	Enrichr CHEA_2022: SOX2 20726797 ChIP-Seq SW620 Human	SOX2
LAPTM4A	Enrichr CHEA_2022: SOX2 20726797 ChIP-Seq SW620 Human	SOX2
STX5	Enrichr CHEA_2022: SOX2 20726797 ChIP-Seq SW620 Human	SOX2
MAP3K8	Enrichr CHEA_2022: SOX2 20726797 ChIP-Seq SW620 Human	SOX2
ZNF420	Enrichr CHEA_2022: SOX2 20726797 ChIP-Seq SW620 Human	SOX2
NEK6	Enrichr CHEA_2022: SOX2 20726797 ChIP-Seq SW620 Human	SOX2
BNIP3L	Enrichr CHEA_2022: SOX2 20726797 ChIP-Seq SW620 Human	SOX2
SLC9A3R1	Enrichr CHEA_2022: SOX2 20726797 ChIP-Seq SW620 Human	SOX2
PIAS3	Enrichr CHEA_2022: SOX2 20726797 ChIP-Seq SW620 Human	SOX2
FBXO31	Enrichr CHEA_2022: SOX2 20726797 ChIP-Seq SW620 Human	SOX2
FBXO32	Enrichr CHEA_2022: SOX2 20726797 ChIP-Seq SW620 Human	SOX2
FBXO30	Enrichr CHEA_2022: SOX2 20726797 ChIP-Seq SW620 Human	SOX2
LCN2	Enrichr CHEA_2022: SOX2 20726797 ChIP-Seq SW620 Human	SOX2
BMP2K	Enrichr CHEA_2022: SOX2 20726797 ChIP-Seq SW620 Human	SOX2
MNAT1	Enrichr CHEA_2022: SOX2 20726797 ChIP-Seq SW620 Human	SOX2
SLC27A5	Enrichr CHEA_2022: SOX2 20726797 ChIP-Seq SW620 Human	SOX2
OSBPL1A	Enrichr CHEA_2022: SOX2 20726797 ChIP-Seq SW620 Human	SOX2
KCNE3	Enrichr CHEA_2022: SOX2 20726797 ChIP-Seq SW620 Human	SOX2
COL17A1	Enrichr CHEA_2022: SOX2 20726797 ChIP-Seq SW620 Human	SOX2
EIF4EBP1	Enrichr CHEA_2022: SOX2 20726797 ChIP-Seq SW620 Human	SOX2
LY75	Enrichr CHEA_2022: SOX2 20726797 ChIP-Seq SW620 Human	SOX2
MSI2	Enrichr CHEA_2022: SOX2 20726797 ChIP-Seq SW620 Human	SOX2
NHSL1	Enrichr CHEA_2022: SOX2 20726797 ChIP-Seq SW620 Human	SOX2
MED31	Enrichr CHEA_2022: SOX2 20726797 ChIP-Seq SW620 Human	SOX2
FNTA	Enrichr CHEA_2022: SOX2 20726797 ChIP-Seq SW620 Human	SOX2
UROD	Enrichr CHEA_2022: SOX2 20726797 ChIP-Seq SW620 Human	SOX2
NEK1	Enrichr CHEA_2022: SOX2 20726797 ChIP-Seq SW620 Human	SOX2
NEK2	Enrichr CHEA_2022: SOX2 20726797 ChIP-Seq SW620 Human	SOX2
NFATC2IP	Enrichr CHEA_2022: SOX2 20726797 ChIP-Seq SW620 Human	SOX2
SLC2A4RG	Enrichr CHEA_2022: SOX2 20726797 ChIP-Seq SW620 Human	SOX2

ITGA5	Enrichr CHEA_2022: SOX2 20726797 ChIP-Seq SW620 Human	SOX2
ITGA3	Enrichr CHEA_2022: SOX2 20726797 ChIP-Seq SW620 Human	SOX2
TNFRSF19	Enrichr CHEA_2022: SOX2 20726797 ChIP-Seq SW620 Human	SOX2
TNFRSF1B	Enrichr CHEA_2022: SOX2 20726797 ChIP-Seq SW620 Human	SOX2
TMBIM1	Enrichr CHEA_2022: SOX2 20726797 ChIP-Seq SW620 Human	SOX2
DBNDD2	Enrichr CHEA_2022: SOX2 20726797 ChIP-Seq SW620 Human	SOX2
TNFRSF21	Enrichr CHEA_2022: SOX2 20726797 ChIP-Seq SW620 Human	SOX2
LCP1	Enrichr CHEA_2022: SOX2 20726797 ChIP-Seq SW620 Human	SOX2
TMBIM4	Enrichr CHEA_2022: SOX2 20726797 ChIP-Seq SW620 Human	SOX2
PCCA	Enrichr CHEA_2022: SOX2 20726797 ChIP-Seq SW620 Human	SOX2
SP5	Enrichr CHEA_2022: SOX2 20726797 ChIP-Seq SW620 Human	SOX2
OGT	Enrichr CHEA_2022: SOX2 20726797 ChIP-Seq SW620 Human	SOX2
KCNK1	Enrichr CHEA_2022: SOX2 20726797 ChIP-Seq SW620 Human	SOX2
BCAR3	Enrichr CHEA_2022: SOX2 20726797 ChIP-Seq SW620 Human	SOX2
MCFD2	Enrichr CHEA_2022: SOX2 20726797 ChIP-Seq SW620 Human	SOX2
SHMT2	Enrichr CHEA_2022: SOX2 20726797 ChIP-Seq SW620 Human	SOX2
MLLT3	Enrichr CHEA_2022: SOX2 20726797 ChIP-Seq SW620 Human	SOX2
SPATA7	Enrichr CHEA_2022: SOX2 20726797 ChIP-Seq SW620 Human	SOX2
MLLT6	Enrichr CHEA_2022: SOX2 20726797 ChIP-Seq SW620 Human	SOX2
ALAD	Enrichr CHEA_2022: SOX2 20726797 ChIP-Seq SW620 Human	SOX2
RDH13	Enrichr CHEA_2022: SOX2 20726797 ChIP-Seq SW620 Human	SOX2
KIAA1522	Enrichr CHEA_2022: SOX2 20726797 ChIP-Seq SW620 Human	SOX2
BOK	Enrichr CHEA_2022: SOX2 20726797 ChIP-Seq SW620 Human	SOX2
BCAS1	Enrichr CHEA_2022: SOX2 20726797 ChIP-Seq SW620 Human	SOX2
NADK	Enrichr CHEA_2022: SOX2 20726797 ChIP-Seq SW620 Human	SOX2
MBIP	Enrichr CHEA_2022: SOX2 20726797 ChIP-Seq SW620 Human	SOX2
STYK1	Enrichr CHEA_2022: SOX2 20726797 ChIP-Seq SW620 Human	SOX2
GZMB	Enrichr CHEA_2022: SOX2 20726797 ChIP-Seq SW620 Human	SOX2
MID1IP1	Enrichr CHEA_2022: SOX2 20726797 ChIP-Seq SW620 Human	SOX2
MYO10	Enrichr CHEA_2022: SOX2 20726797 ChIP-Seq SW620 Human	SOX2
RNASET2	Enrichr CHEA_2022: SOX2 20726797 ChIP-Seq SW620 Human	SOX2
CABLES1	Enrichr CHEA_2022: SOX2 20726797 ChIP-Seq SW620 Human	SOX2
TM6SF1	Enrichr CHEA_2022: SOX2 20726797 ChIP-Seq SW620 Human	SOX2
TBX3	Enrichr CHEA_2022: SOX2 20726797 ChIP-Seq SW620 Human	SOX2
CFDP1	Enrichr CHEA_2022: SOX2 20726797 ChIP-Seq SW620 Human	SOX2
MYO1C	Enrichr CHEA_2022: SOX2 20726797 ChIP-Seq SW620 Human	SOX2
TSPAN14	Enrichr CHEA_2022: SOX2 20726797 ChIP-Seq SW620 Human	SOX2
MYO1B	Enrichr CHEA_2022: SOX2 20726797 ChIP-Seq SW620 Human	SOX2
TRIM36	Enrichr CHEA_2022: SOX2 20726797 ChIP-Seq SW620 Human	SOX2
KCTD15	Enrichr CHEA_2022: SOX2 20726797 ChIP-Seq SW620 Human	SOX2
KCTD12	Enrichr CHEA_2022: SOX2 20726797 ChIP-Seq SW620 Human	SOX2
TRIM32	Enrichr CHEA_2022: SOX2 20726797 ChIP-Seq SW620 Human	SOX2
PIK3AP1	Enrichr CHEA_2022: SOX2 20726797 ChIP-Seq SW620 Human	SOX2
TRIM34	Enrichr CHEA_2022: SOX2 20726797 ChIP-Seq SW620 Human	SOX2
PRPS2	Enrichr CHEA_2022: SOX2 20726797 ChIP-Seq SW620 Human	SOX2
SRC	Enrichr CHEA_2022: SOX2 20726797 ChIP-Seq SW620 Human	SOX2
MSLN	Enrichr CHEA_2022: SOX2 20726797 ChIP-Seq SW620 Human	SOX2
TRIM31	Enrichr CHEA_2022: SOX2 20726797 ChIP-Seq SW620 Human	SOX2
ITGB5	Enrichr CHEA_2022: SOX2 20726797 ChIP-Seq SW620 Human	SOX2
SRI	Enrichr CHEA_2022: SOX2 20726797 ChIP-Seq SW620 Human	SOX2
TSPAN12	Enrichr CHEA_2022: SOX2 20726797 ChIP-Seq SW620 Human	SOX2
NRGN	Enrichr CHEA_2022: SOX2 20726797 ChIP-Seq SW620 Human	SOX2
ZNRF1	Enrichr CHEA_2022: SOX2 20726797 ChIP-Seq SW620 Human	SOX2
ZNRF2	Enrichr CHEA_2022: SOX2 20726797 ChIP-Seq SW620 Human	SOX2
SRR	Enrichr CHEA_2022: SOX2 20726797 ChIP-Seq SW620 Human	SOX2
HHEX	Enrichr CHEA_2022: SOX2 20726797 ChIP-Seq SW620 Human	SOX2
KIAA1549	Enrichr CHEA_2022: SOX2 20726797 ChIP-Seq SW620 Human	SOX2
TRIM29	Enrichr CHEA_2022: SOX2 20726797 ChIP-Seq SW620 Human	SOX2
PTER	Enrichr CHEA_2022: SOX2 20726797 ChIP-Seq SW620 Human	SOX2

IGF2BP1	Enrichr CHEA_2022: SOX2 20726797 ChIP-Seq SW620 Human	SOX2
ITGB8	Enrichr CHEA_2022: SOX2 20726797 ChIP-Seq SW620 Human	SOX2
ITGAV	Enrichr CHEA_2022: SOX2 20726797 ChIP-Seq SW620 Human	SOX2
FYN	Enrichr CHEA_2022: SOX2 20726797 ChIP-Seq SW620 Human	SOX2
ARL5B	Enrichr CHEA_2022: SOX2 20726797 ChIP-Seq SW620 Human	SOX2
SMARCA1	Enrichr CHEA_2022: SOX2 20726797 ChIP-Seq SW620 Human	SOX2
ACTN4	Enrichr CHEA_2022: SOX2 20726797 ChIP-Seq SW620 Human	SOX2
FBXO3	Enrichr CHEA_2022: SOX2 20726797 ChIP-Seq SW620 Human	SOX2
ARL14	Enrichr CHEA_2022: SOX2 20726797 ChIP-Seq SW620 Human	SOX2
SMARCA2	Enrichr CHEA_2022: SOX2 20726797 ChIP-Seq SW620 Human	SOX2
PPA2	Enrichr CHEA_2022: SOX2 20726797 ChIP-Seq SW620 Human	SOX2
SOAT1	Enrichr CHEA_2022: SOX2 20726797 ChIP-Seq SW620 Human	SOX2
NINJ2	Enrichr CHEA_2022: SOX2 20726797 ChIP-Seq SW620 Human	SOX2
MT1G	Enrichr CHEA_2022: SOX2 20726797 ChIP-Seq SW620 Human	SOX2
MT1H	Enrichr CHEA_2022: SOX2 20726797 ChIP-Seq SW620 Human	SOX2
TRIM16	Enrichr CHEA_2022: SOX2 20726797 ChIP-Seq SW620 Human	SOX2
CCDC82	Enrichr CHEA_2022: SOX2 20726797 ChIP-Seq SW620 Human	SOX2
RIN3	Enrichr CHEA_2022: SOX2 20726797 ChIP-Seq SW620 Human	SOX2
MT1F	Enrichr CHEA_2022: SOX2 20726797 ChIP-Seq SW620 Human	SOX2
SLC23A3	Enrichr CHEA_2022: SOX2 20726797 ChIP-Seq SW620 Human	SOX2
EPAS1	Enrichr CHEA_2022: SOX2 20726797 ChIP-Seq SW620 Human	SOX2
TAGLN	Enrichr CHEA_2022: SOX2 20726797 ChIP-Seq SW620 Human	SOX2
CCDC69	Enrichr CHEA_2022: SOX2 20726797 ChIP-Seq SW620 Human	SOX2
MYCBP2	Enrichr CHEA_2022: SOX2 20726797 ChIP-Seq SW620 Human	SOX2
CPNE8	Enrichr CHEA_2022: SOX2 20726797 ChIP-Seq SW620 Human	SOX2
IL27RA	Enrichr CHEA_2022: SOX2 20726797 ChIP-Seq SW620 Human	SOX2
STS	Enrichr CHEA_2022: SOX2 20726797 ChIP-Seq SW620 Human	SOX2
CDC23	Enrichr CHEA_2022: SOX2 20726797 ChIP-Seq SW620 Human	SOX2
BSG	Enrichr CHEA_2022: SOX2 20726797 ChIP-Seq SW620 Human	SOX2
CSNK1D	Enrichr CHEA_2022: SOX2 20726797 ChIP-Seq SW620 Human	SOX2
PEG10	Enrichr CHEA_2022: SOX2 20726797 ChIP-Seq SW620 Human	SOX2
STK38L	Enrichr CHEA_2022: SOX2 20726797 ChIP-Seq SW620 Human	SOX2
CCDC77	Enrichr CHEA_2022: SOX2 20726797 ChIP-Seq SW620 Human	SOX2
E2F8	Enrichr CHEA_2022: SOX2 20726797 ChIP-Seq SW620 Human	SOX2
EGR3	Enrichr CHEA_2022: SOX2 20726797 ChIP-Seq SW620 Human	SOX2
TGDS	Enrichr CHEA_2022: SOX2 20726797 ChIP-Seq SW620 Human	SOX2
MYO5A	Enrichr CHEA_2022: SOX2 20726797 ChIP-Seq SW620 Human	SOX2
RAB27B	Enrichr CHEA_2022: SOX2 20726797 ChIP-Seq SW620 Human	SOX2
MYO5B	Enrichr CHEA_2022: SOX2 20726797 ChIP-Seq SW620 Human	SOX2
CSNK1E	Enrichr CHEA_2022: SOX2 20726797 ChIP-Seq SW620 Human	SOX2
PTK6	Enrichr CHEA_2022: SOX2 20726797 ChIP-Seq SW620 Human	SOX2
EXT1	Enrichr CHEA_2022: SOX2 20726797 ChIP-Seq SW620 Human	SOX2
PTK2	Enrichr CHEA_2022: SOX2 20726797 ChIP-Seq SW620 Human	SOX2
CNKSR3	Enrichr CHEA_2022: SOX2 20726797 ChIP-Seq SW620 Human	SOX2
BTC	Enrichr CHEA_2022: SOX2 20726797 ChIP-Seq SW620 Human	SOX2
TLCD1	Enrichr CHEA_2022: SOX2 20726797 ChIP-Seq SW620 Human	SOX2
FBXL2	Enrichr CHEA_2022: SOX2 20726797 ChIP-Seq SW620 Human	SOX2
SDC1	Enrichr CHEA_2022: SOX2 20726797 ChIP-Seq SW620 Human	SOX2
MON1B	Enrichr CHEA_2022: SOX2 20726797 ChIP-Seq SW620 Human	SOX2
APOBEC3B	Enrichr CHEA_2022: SOX2 20726797 ChIP-Seq SW620 Human	SOX2
ATP6V1B2	Enrichr CHEA_2022: SOX2 20726797 ChIP-Seq SW620 Human	SOX2
APOBEC3C	Enrichr CHEA_2022: SOX2 20726797 ChIP-Seq SW620 Human	SOX2
LTV1	Enrichr CHEA_2022: SOX2 20726797 ChIP-Seq SW620 Human	SOX2
SLC45A4	Enrichr CHEA_2022: SOX2 20726797 ChIP-Seq SW620 Human	SOX2
WFDC2	Enrichr CHEA_2022: SOX2 20726797 ChIP-Seq SW620 Human	SOX2
MT1X	Enrichr CHEA_2022: SOX2 20726797 ChIP-Seq SW620 Human	SOX2
ICAM2	Enrichr CHEA_2022: SOX2 20726797 ChIP-Seq SW620 Human	SOX2
LGALS4	Enrichr CHEA_2022: SOX2 20726797 ChIP-Seq SW620 Human	SOX2
MT2A	Enrichr CHEA_2022: SOX2 20726797 ChIP-Seq SW620 Human	SOX2

CCDC92	Enrichr CHEA_2022: SOX2 20726797 ChIP-Seq SW620 Human	SOX2
LGALS1	Enrichr CHEA_2022: SOX2 20726797 ChIP-Seq SW620 Human	SOX2
REG4	Enrichr CHEA_2022: SOX2 20726797 ChIP-Seq SW620 Human	SOX2
TNFSF10	Enrichr CHEA_2022: SOX2 20726797 ChIP-Seq SW620 Human	SOX2
LGALS8	Enrichr CHEA_2022: SOX2 20726797 ChIP-Seq SW620 Human	SOX2
CCDC96	Enrichr CHEA_2022: SOX2 20726797 ChIP-Seq SW620 Human	SOX2
SPA17	Enrichr CHEA_2022: SOX2 20726797 ChIP-Seq SW620 Human	SOX2
GABBR1	Enrichr CHEA_2022: SOX2 20726797 ChIP-Seq SW620 Human	SOX2
IL15	Enrichr CHEA_2022: SOX2 20726797 ChIP-Seq SW620 Human	SOX2
SLC10A3	Enrichr CHEA_2022: SOX2 20726797 ChIP-Seq SW620 Human	SOX2
GAB2	Enrichr CHEA_2022: SOX2 20726797 ChIP-Seq SW620 Human	SOX2
GAB1	Enrichr CHEA_2022: SOX2 20726797 ChIP-Seq SW620 Human	SOX2
KLHL3	Enrichr CHEA_2022: SOX2 20726797 ChIP-Seq SW620 Human	SOX2
KLHL5	Enrichr CHEA_2022: SOX2 20726797 ChIP-Seq SW620 Human	SOX2
ZHX1	Enrichr CHEA_2022: SOX2 20726797 ChIP-Seq SW620 Human	SOX2
RAP2A	Enrichr CHEA_2022: SOX2 20726797 ChIP-Seq SW620 Human	SOX2
CTTNBP2NL	Enrichr CHEA_2022: SOX2 20726797 ChIP-Seq SW620 Human	SOX2
BST2	Enrichr CHEA_2022: SOX2 20726797 ChIP-Seq SW620 Human	SOX2
DEPDC7	Enrichr CHEA_2022: SOX2 20726797 ChIP-Seq SW620 Human	SOX2
KITLG	Enrichr CHEA_2022: SOX2 20726797 ChIP-Seq SW620 Human	SOX2
ARHGEF4	Enrichr CHEA_2022: SOX2 20726797 ChIP-Seq SW620 Human	SOX2
ARHGEF3	Enrichr CHEA_2022: SOX2 20726797 ChIP-Seq SW620 Human	SOX2
FJX1	Enrichr CHEA_2022: SOX2 20726797 ChIP-Seq SW620 Human	SOX2
RANBP10	Enrichr CHEA_2022: SOX2 20726797 ChIP-Seq SW620 Human	SOX2
NRN1	Enrichr CHEA_2022: SOX2 20726797 ChIP-Seq SW620 Human	SOX2
NOXO1	Enrichr CHEA_2022: SOX2 20726797 ChIP-Seq SW620 Human	SOX2
PPCS	Enrichr CHEA_2022: SOX2 20726797 ChIP-Seq SW620 Human	SOX2
TFB1M	Enrichr CHEA_2022: SOX2 20726797 ChIP-Seq SW620 Human	SOX2
KALRN	Enrichr CHEA_2022: SOX2 20726797 ChIP-Seq SW620 Human	SOX2
PPP3CA	Enrichr CHEA_2022: SOX2 20726797 ChIP-Seq SW620 Human	SOX2
SEC14L4	Enrichr CHEA_2022: SOX2 20726797 ChIP-Seq SW620 Human	SOX2
RRAS	Enrichr CHEA_2022: SOX2 20726797 ChIP-Seq SW620 Human	SOX2
SMYD3	Enrichr CHEA_2022: SOX2 20726797 ChIP-Seq SW620 Human	SOX2
HTATIP2	Enrichr CHEA_2022: SOX2 20726797 ChIP-Seq SW620 Human	SOX2
PHGDH	Enrichr CHEA_2022: SOX2 20726797 ChIP-Seq SW620 Human	SOX2
NFIL3	Enrichr CHEA_2022: SOX2 20726797 ChIP-Seq SW620 Human	SOX2
PCK2	Enrichr CHEA_2022: SOX2 20726797 ChIP-Seq SW620 Human	SOX2
LYAR	Enrichr CHEA_2022: SOX2 20726797 ChIP-Seq SW620 Human	SOX2
CAP2	Enrichr CHEA_2022: SOX2 20726797 ChIP-Seq SW620 Human	SOX2
GAL	Enrichr CHEA_2022: SOX2 20726797 ChIP-Seq SW620 Human	SOX2
TPST2	Enrichr CHEA_2022: SOX2 20726797 ChIP-Seq SW620 Human	SOX2
TTC9	Enrichr CHEA_2022: SOX2 20726797 ChIP-Seq SW620 Human	SOX2
TTC8	Enrichr CHEA_2022: SOX2 20726797 ChIP-Seq SW620 Human	SOX2
KCNQ1	Enrichr CHEA_2022: SOX2 20726797 ChIP-Seq SW620 Human	SOX2
TPST1	Enrichr CHEA_2022: SOX2 20726797 ChIP-Seq SW620 Human	SOX2
TCEA2	Enrichr CHEA_2022: SOX2 20726797 ChIP-Seq SW620 Human	SOX2
TACC1	Enrichr CHEA_2022: SOX2 20726797 ChIP-Seq SW620 Human	SOX2
PNPLA3	Enrichr CHEA_2022: SOX2 20726797 ChIP-Seq SW620 Human	SOX2
NRP2	Enrichr CHEA_2022: SOX2 20726797 ChIP-Seq SW620 Human	SOX2
CAPG	Enrichr CHEA_2022: SOX2 20726797 ChIP-Seq SW620 Human	SOX2
NUDT4	Enrichr CHEA_2022: SOX2 20726797 ChIP-Seq SW620 Human	SOX2
SPAG16	Enrichr CHEA_2022: SOX2 20726797 ChIP-Seq SW620 Human	SOX2
HERPUD2	Enrichr CHEA_2022: SOX2 20726797 ChIP-Seq SW620 Human	SOX2
AKAP13	Enrichr CHEA_2022: SOX2 20726797 ChIP-Seq SW620 Human	SOX2
AKAP11	Enrichr CHEA_2022: SOX2 20726797 ChIP-Seq SW620 Human	SOX2
PCM1	Enrichr CHEA_2022: SOX2 20726797 ChIP-Seq SW620 Human	SOX2
AKAP12	Enrichr CHEA_2022: SOX2 20726797 ChIP-Seq SW620 Human	SOX2
FAM3C	Enrichr CHEA_2022: SOX2 20726797 ChIP-Seq SW620 Human	SOX2
FAM3B	Enrichr CHEA_2022: SOX2 20726797 ChIP-Seq SW620 Human	SOX2

FAM3D	Enrichr CHEA_2022: SOX2 20726797 ChIP-Seq SW620 Human	SOX2
RARRES1	Enrichr CHEA_2022: SOX2 20726797 ChIP-Seq SW620 Human	SOX2
SPINK5	Enrichr CHEA_2022: SOX2 20726797 ChIP-Seq SW620 Human	SOX2
HS3ST1	Enrichr CHEA_2022: SOX2 20726797 ChIP-Seq SW620 Human	SOX2
UBE2H	Enrichr CHEA_2022: SOX2 20726797 ChIP-Seq SW620 Human	SOX2
MORC4	Enrichr CHEA_2022: SOX2 20726797 ChIP-Seq SW620 Human	SOX2
FAM3A	Enrichr CHEA_2022: SOX2 20726797 ChIP-Seq SW620 Human	SOX2
CACHD1	Enrichr CHEA_2022: SOX2 20726797 ChIP-Seq SW620 Human	SOX2
DNAJB14	Enrichr CHEA_2022: SOX2 20726797 ChIP-Seq SW620 Human	SOX2
BSPRY	Enrichr CHEA_2022: SOX2 20726797 ChIP-Seq SW620 Human	SOX2
CDKAL1	Enrichr CHEA_2022: SOX2 20726797 ChIP-Seq SW620 Human	SOX2
YPEL2	Enrichr CHEA_2022: SOX2 20726797 ChIP-Seq SW620 Human	SOX2
WDFY2	Enrichr CHEA_2022: SOX2 20726797 ChIP-Seq SW620 Human	SOX2
DEGS2	Enrichr CHEA_2022: SOX2 20726797 ChIP-Seq SW620 Human	SOX2
WDFY3	Enrichr CHEA_2022: SOX2 20726797 ChIP-Seq SW620 Human	SOX2
OTUD1	Enrichr CHEA_2022: SOX2 20726797 ChIP-Seq SW620 Human	SOX2
PPIC	Enrichr CHEA_2022: SOX2 20726797 ChIP-Seq SW620 Human	SOX2
PPID	Enrichr CHEA_2022: SOX2 20726797 ChIP-Seq SW620 Human	SOX2
GDA	Enrichr CHEA_2022: SOX2 20726797 ChIP-Seq SW620 Human	SOX2
NAPA	Enrichr CHEA_2022: SOX2 20726797 ChIP-Seq SW620 Human	SOX2
SCNN1A	Enrichr CHEA_2022: SOX2 20726797 ChIP-Seq SW620 Human	SOX2
PRDM1	Enrichr CHEA_2022: SOX2 20726797 ChIP-Seq SW620 Human	SOX2
LOXL4	Enrichr CHEA_2022: SOX2 20726797 ChIP-Seq SW620 Human	SOX2
LOXL2	Enrichr CHEA_2022: SOX2 20726797 ChIP-Seq SW620 Human	SOX2
LOXL3	Enrichr CHEA_2022: SOX2 20726797 ChIP-Seq SW620 Human	SOX2
HSD17B1	Enrichr CHEA_2022: SOX2 20726797 ChIP-Seq SW620 Human	SOX2
HSD17B2	Enrichr CHEA_2022: SOX2 20726797 ChIP-Seq SW620 Human	SOX2
MSX1	Enrichr CHEA_2022: SOX2 20726797 ChIP-Seq SW620 Human	SOX2
TBC1D16	Enrichr CHEA_2022: SOX2 20726797 ChIP-Seq SW620 Human	SOX2
TBC1D17	Enrichr CHEA_2022: SOX2 20726797 ChIP-Seq SW620 Human	SOX2
DENND2D	Enrichr CHEA_2022: SOX2 20726797 ChIP-Seq SW620 Human	SOX2
PCYT1B	Enrichr CHEA_2022: SOX2 20726797 ChIP-Seq SW620 Human	SOX2
SYT1	Enrichr CHEA_2022: SOX2 20726797 ChIP-Seq SW620 Human	SOX2
TIMM44	Enrichr CHEA_2022: SOX2 20726797 ChIP-Seq SW620 Human	SOX2
NAT1	Enrichr CHEA_2022: SOX2 20726797 ChIP-Seq SW620 Human	SOX2
CCNB1IP1	Enrichr CHEA_2022: SOX2 20726797 ChIP-Seq SW620 Human	SOX2
FGF18	Enrichr CHEA_2022: SOX2 20726797 ChIP-Seq SW620 Human	SOX2
ZNF91	Enrichr CHEA_2022: SOX2 20726797 ChIP-Seq SW620 Human	SOX2
GNB2	Enrichr CHEA_2022: SOX2 20726797 ChIP-Seq SW620 Human	SOX2
MKRN2	Enrichr CHEA_2022: SOX2 20726797 ChIP-Seq SW620 Human	SOX2
GNB5	Enrichr CHEA_2022: SOX2 20726797 ChIP-Seq SW620 Human	SOX2
GNAS	Enrichr CHEA_2022: SOX2 20726797 ChIP-Seq SW620 Human	SOX2
MUC20	Enrichr CHEA_2022: SOX2 20726797 ChIP-Seq SW620 Human	SOX2
TCF3	Enrichr CHEA_2022: SOX2 20726797 ChIP-Seq SW620 Human	SOX2
TSNARE1	Enrichr CHEA_2022: SOX2 20726797 ChIP-Seq SW620 Human	SOX2
VIL1	Enrichr CHEA_2022: SOX2 20726797 ChIP-Seq SW620 Human	SOX2
DYRK4	Enrichr CHEA_2022: SOX2 20726797 ChIP-Seq SW620 Human	SOX2
DYRK3	Enrichr CHEA_2022: SOX2 20726797 ChIP-Seq SW620 Human	SOX2
DYRK2	Enrichr CHEA_2022: SOX2 20726797 ChIP-Seq SW620 Human	SOX2
SLC41A2	Enrichr CHEA_2022: SOX2 20726797 ChIP-Seq SW620 Human	SOX2
PLAU	Enrichr CHEA_2022: SOX2 20726797 ChIP-Seq SW620 Human	SOX2
HTRA1	Enrichr CHEA_2022: SOX2 20726797 ChIP-Seq SW620 Human	SOX2
ETS1	Enrichr CHEA_2022: SOX2 20726797 ChIP-Seq SW620 Human	SOX2
ETS2	Enrichr CHEA_2022: SOX2 20726797 ChIP-Seq SW620 Human	SOX2
DAPP1	Enrichr CHEA_2022: SOX2 20726797 ChIP-Seq SW620 Human	SOX2
IGF1R	Enrichr CHEA_2022: SOX2 20726797 ChIP-Seq SW620 Human	SOX2
TGM2	Enrichr CHEA_2022: SOX2 20726797 ChIP-Seq SW620 Human	SOX2
CNN3	Enrichr CHEA_2022: SOX2 20726797 ChIP-Seq SW620 Human	SOX2
ARFGEF1	Enrichr CHEA_2022: SOX2 20726797 ChIP-Seq SW620 Human	SOX2

GGH	Enrichr CHEA_2022: SOX2 20726797 ChIP-Seq SW620 Human	SOX2
CAV1	Enrichr CHEA_2022: SOX2 20726797 ChIP-Seq SW620 Human	SOX2
NAV1	Enrichr CHEA_2022: SOX2 20726797 ChIP-Seq SW620 Human	SOX2
ETV1	Enrichr CHEA_2022: SOX2 20726797 ChIP-Seq SW620 Human	SOX2
NAV2	Enrichr CHEA_2022: SOX2 20726797 ChIP-Seq SW620 Human	SOX2
ETV5	Enrichr CHEA_2022: SOX2 20726797 ChIP-Seq SW620 Human	SOX2
TTC17	Enrichr CHEA_2022: SOX2 20726797 ChIP-Seq SW620 Human	SOX2
ETV6	Enrichr CHEA_2022: SOX2 20726797 ChIP-Seq SW620 Human	SOX2
TAPBP	Enrichr CHEA_2022: SOX2 20726797 ChIP-Seq SW620 Human	SOX2
EPN3	Enrichr CHEA_2022: SOX2 20726797 ChIP-Seq SW620 Human	SOX2
ELF2	Enrichr CHEA_2022: SOX2 20726797 ChIP-Seq SW620 Human	SOX2
ELF3	Enrichr CHEA_2022: SOX2 20726797 ChIP-Seq SW620 Human	SOX2
MANSC1	Enrichr CHEA_2022: SOX2 20726797 ChIP-Seq SW620 Human	SOX2
CUEDC1	Enrichr CHEA_2022: SOX2 20726797 ChIP-Seq SW620 Human	SOX2
FBP1	Enrichr CHEA_2022: SOX2 20726797 ChIP-Seq SW620 Human	SOX2
SHANK2	Enrichr CHEA_2022: SOX2 20726797 ChIP-Seq SW620 Human	SOX2
TTC26	Enrichr CHEA_2022: SOX2 20726797 ChIP-Seq SW620 Human	SOX2
NNMT	Enrichr CHEA_2022: SOX2 20726797 ChIP-Seq SW620 Human	SOX2
TNFAIP8	Enrichr CHEA_2022: SOX2 20726797 ChIP-Seq SW620 Human	SOX2
TNFAIP2	Enrichr CHEA_2022: SOX2 20726797 ChIP-Seq SW620 Human	SOX2
PITPNC1	Enrichr CHEA_2022: SOX2 20726797 ChIP-Seq SW620 Human	SOX2
AGPAT5	Enrichr CHEA_2022: SOX2 20726797 ChIP-Seq SW620 Human	SOX2
CA2	Enrichr CHEA_2022: SOX2 20726797 ChIP-Seq SW620 Human	SOX2
IRAK1	Enrichr CHEA_2022: SOX2 20726797 ChIP-Seq SW620 Human	SOX2
LPXN	Enrichr CHEA_2022: SOX2 20726797 ChIP-Seq SW620 Human	SOX2
ATF7IP	Enrichr CHEA_2022: SOX2 20726797 ChIP-Seq SW620 Human	SOX2
DAPK1	Enrichr CHEA_2022: SOX2 20726797 ChIP-Seq SW620 Human	SOX2
ERCC1	Enrichr CHEA_2022: SOX2 20726797 ChIP-Seq SW620 Human	SOX2
GALE	Enrichr CHEA_2022: SOX2 20726797 ChIP-Seq SW620 Human	SOX2
KRAS	Enrichr CHEA_2022: SOX2 20726797 ChIP-Seq SW620 Human	SOX2
TRIB2	Enrichr CHEA_2022: SOX2 20726797 ChIP-Seq SW620 Human	SOX2
TRIB3	Enrichr CHEA_2022: SOX2 20726797 ChIP-Seq SW620 Human	SOX2
SIGIRR	Enrichr CHEA_2022: SOX2 20726797 ChIP-Seq SW620 Human	SOX2
TRIB1	Enrichr CHEA_2022: SOX2 20726797 ChIP-Seq SW620 Human	SOX2
RND3	Enrichr CHEA_2022: SOX2 20726797 ChIP-Seq SW620 Human	SOX2
NR3C1	Enrichr CHEA_2022: SOX2 20726797 ChIP-Seq SW620 Human	SOX2
POLD4	Enrichr CHEA_2022: SOX2 20726797 ChIP-Seq SW620 Human	SOX2
ACOXL	Enrichr CHEA_2022: SOX2 20726797 ChIP-Seq SW620 Human	SOX2
OXCT1	Enrichr CHEA_2022: SOX2 20726797 ChIP-Seq SW620 Human	SOX2
DIP2C	Enrichr CHEA_2022: SOX2 20726797 ChIP-Seq SW620 Human	SOX2
HOMER3	Enrichr CHEA_2022: SOX2 20726797 ChIP-Seq SW620 Human	SOX2
PCGF6	Enrichr CHEA_2022: SOX2 20726797 ChIP-Seq SW620 Human	SOX2
HUS1	Enrichr CHEA_2022: SOX2 20726797 ChIP-Seq SW620 Human	SOX2
NUP93	Enrichr CHEA_2022: SOX2 20726797 ChIP-Seq SW620 Human	SOX2
CAPN12	Enrichr CHEA_2022: SOX2 20726797 ChIP-Seq SW620 Human	SOX2
DEPDC1B	Enrichr CHEA_2022: SOX2 20726797 ChIP-Seq SW620 Human	SOX2
TEF	Enrichr CHEA_2022: SOX2 20726797 ChIP-Seq SW620 Human	SOX2
RALB	Enrichr CHEA_2022: SOX2 20726797 ChIP-Seq SW620 Human	SOX2
ZNF395	Enrichr CHEA_2022: SOX2 20726797 ChIP-Seq SW620 Human	SOX2
ADARB1	Enrichr CHEA_2022: SOX2 20726797 ChIP-Seq SW620 Human	SOX2
GLS	Enrichr CHEA_2022: SOX2 20726797 ChIP-Seq SW620 Human	SOX2
ASCL2	Enrichr CHEA_2022: SOX2 20726797 ChIP-Seq SW620 Human	SOX2
ADCY7	Enrichr CHEA_2022: SOX2 20726797 ChIP-Seq SW620 Human	SOX2
GNG2	Enrichr CHEA_2022: SOX2 20726797 ChIP-Seq SW620 Human	SOX2
APOL6	Enrichr CHEA_2022: SOX2 20726797 ChIP-Seq SW620 Human	SOX2
GNG4	Enrichr CHEA_2022: SOX2 20726797 ChIP-Seq SW620 Human	SOX2
FLRT3	Enrichr CHEA_2022: SOX2 20726797 ChIP-Seq SW620 Human	SOX2
WDR72	Enrichr CHEA_2022: SOX2 20726797 ChIP-Seq SW620 Human	SOX2
ALOX5	Enrichr CHEA_2022: SOX2 20726797 ChIP-Seq SW620 Human	SOX2

ST3GAL5	Enrichr CHEA_2022: SOX2 20726797 ChIP-Seq SW620 Human	SOX2
PRSS8	Enrichr CHEA_2022: SOX2 20726797 ChIP-Seq SW620 Human	SOX2
LUC7L	Enrichr CHEA_2022: SOX2 20726797 ChIP-Seq SW620 Human	SOX2
NUP88	Enrichr CHEA_2022: SOX2 20726797 ChIP-Seq SW620 Human	SOX2
ST3GAL1	Enrichr CHEA_2022: SOX2 20726797 ChIP-Seq SW620 Human	SOX2
PRSS3	Enrichr CHEA_2022: SOX2 20726797 ChIP-Seq SW620 Human	SOX2
APOL1	Enrichr CHEA_2022: SOX2 20726797 ChIP-Seq SW620 Human	SOX2
FDFT1	Enrichr CHEA_2022: SOX2 20726797 ChIP-Seq SW620 Human	SOX2
ST3GAL4	Enrichr CHEA_2022: SOX2 20726797 ChIP-Seq SW620 Human	SOX2
TAF9B	Enrichr CHEA_2022: SOX2 20726797 ChIP-Seq SW620 Human	SOX2
PRKCH	Enrichr CHEA_2022: SOX2 20726797 ChIP-Seq SW620 Human	SOX2
NDFIP1	Enrichr CHEA_2022: SOX2 20726797 ChIP-Seq SW620 Human	SOX2
PHC3	Enrichr CHEA_2022: SOX2 20726797 ChIP-Seq SW620 Human	SOX2
ICMT	Enrichr CHEA_2022: SOX2 20726797 ChIP-Seq SW620 Human	SOX2
WDR59	Enrichr CHEA_2022: SOX2 20726797 ChIP-Seq SW620 Human	SOX2
STARD13	Enrichr CHEA_2022: SOX2 20726797 ChIP-Seq SW620 Human	SOX2
STARD10	Enrichr CHEA_2022: SOX2 20726797 ChIP-Seq SW620 Human	SOX2
PLK2	Enrichr CHEA_2022: SOX2 20726797 ChIP-Seq SW620 Human	SOX2
PRKCE	Enrichr CHEA_2022: SOX2 20726797 ChIP-Seq SW620 Human	SOX2
PARD3	Enrichr CHEA_2022: SOX2 20726797 ChIP-Seq SW620 Human	SOX2
PRKCA	Enrichr CHEA_2022: SOX2 20726797 ChIP-Seq SW620 Human	SOX2
TERF2	Enrichr CHEA_2022: SOX2 20726797 ChIP-Seq SW620 Human	SOX2
ELL2	Enrichr CHEA_2022: SOX2 20726797 ChIP-Seq SW620 Human	SOX2
ELL3	Enrichr CHEA_2022: SOX2 20726797 ChIP-Seq SW620 Human	SOX2
COQ10A	Enrichr CHEA_2022: SOX2 20726797 ChIP-Seq SW620 Human	SOX2
EHD2	Enrichr CHEA_2022: SOX2 20726797 ChIP-Seq SW620 Human	SOX2
SQLE	Enrichr CHEA_2022: SOX2 20726797 ChIP-Seq SW620 Human	SOX2
GJB3	Enrichr CHEA_2022: SOX2 20726797 ChIP-Seq SW620 Human	SOX2
GJB2	Enrichr CHEA_2022: SOX2 20726797 ChIP-Seq SW620 Human	SOX2
TCN1	Enrichr CHEA_2022: SOX2 20726797 ChIP-Seq SW620 Human	SOX2
SNAI2	Enrichr CHEA_2022: SOX2 20726797 ChIP-Seq SW620 Human	SOX2
PRKD2	Enrichr CHEA_2022: SOX2 20726797 ChIP-Seq SW620 Human	SOX2
GNE	Enrichr CHEA_2022: SOX2 20726797 ChIP-Seq SW620 Human	SOX2
CFB	Enrichr CHEA_2022: SOX2 20726797 ChIP-Seq SW620 Human	SOX2
FANK1	Enrichr CHEA_2022: SOX2 20726797 ChIP-Seq SW620 Human	SOX2
CFH	Enrichr CHEA_2022: SOX2 20726797 ChIP-Seq SW620 Human	SOX2
PHYHIPL	Enrichr CHEA_2022: SOX2 20726797 ChIP-Seq SW620 Human	SOX2
GNS	Enrichr CHEA_2022: SOX2 20726797 ChIP-Seq SW620 Human	SOX2
ELK3	Enrichr CHEA_2022: SOX2 20726797 ChIP-Seq SW620 Human	SOX2
PLAC8	Enrichr CHEA_2022: SOX2 20726797 ChIP-Seq SW620 Human	SOX2
NUP62	Enrichr CHEA_2022: SOX2 20726797 ChIP-Seq SW620 Human	SOX2
AP1S3	Enrichr CHEA_2022: SOX2 20726797 ChIP-Seq SW620 Human	SOX2
CEP72	Enrichr CHEA_2022: SOX2 20726797 ChIP-Seq SW620 Human	SOX2
AP1S2	Enrichr CHEA_2022: SOX2 20726797 ChIP-Seq SW620 Human	SOX2
PROM2	Enrichr CHEA_2022: SOX2 20726797 ChIP-Seq SW620 Human	SOX2
PROM1	Enrichr CHEA_2022: SOX2 20726797 ChIP-Seq SW620 Human	SOX2
ZNF367	Enrichr CHEA_2022: SOX2 20726797 ChIP-Seq SW620 Human	SOX2
SHPRH	Enrichr CHEA_2022: SOX2 20726797 ChIP-Seq SW620 Human	SOX2
PHF10	Enrichr CHEA_2022: SOX2 20726797 ChIP-Seq SW620 Human	SOX2
PHF13	Enrichr CHEA_2022: SOX2 20726797 ChIP-Seq SW620 Human	SOX2
GRTP1	Enrichr CHEA_2022: SOX2 20726797 ChIP-Seq SW620 Human	SOX2
CGN	Enrichr CHEA_2022: SOX2 20726797 ChIP-Seq SW620 Human	SOX2
TCP1	Enrichr CHEA_2022: SOX2 20726797 ChIP-Seq SW620 Human	SOX2
NFATC3	Enrichr CHEA_2022: SOX2 20726797 ChIP-Seq SW620 Human	SOX2
ASH1L	Enrichr CHEA_2022: SOX2 20726797 ChIP-Seq SW620 Human	SOX2
WDR35	Enrichr CHEA_2022: SOX2 20726797 ChIP-Seq SW620 Human	SOX2
TUBB2B	Enrichr CHEA_2022: SOX2 20726797 ChIP-Seq SW620 Human	SOX2
HLA-DQB1	Enrichr CHEA_2022: SOX2 20726797 ChIP-Seq SW620 Human	SOX2
PHF14	Enrichr CHEA_2022: SOX2 20726797 ChIP-Seq SW620 Human	SOX2

RARA	Enrichr CHEA_2022: SOX2 20726797 ChIP-Seq SW620 Human	SOX2
MGLL	Enrichr CHEA_2022: SOX2 20726797 ChIP-Seq SW620 Human	SOX2
GAS6	Enrichr CHEA_2022: SOX2 20726797 ChIP-Seq SW620 Human	SOX2
BTG3	Enrichr CHEA_2022: SOX2 20726797 ChIP-Seq SW620 Human	SOX2
BTG2	Enrichr CHEA_2022: SOX2 20726797 ChIP-Seq SW620 Human	SOX2
BTG1	Enrichr CHEA_2022: SOX2 20726797 ChIP-Seq SW620 Human	SOX2
PRDM11	Enrichr CHEA_2022: SOX2 20726797 ChIP-Seq SW620 Human	SOX2
TIMP4	Enrichr CHEA_2022: SOX2 20726797 ChIP-Seq SW620 Human	SOX2
PBK	Enrichr CHEA_2022: SOX2 20726797 ChIP-Seq SW620 Human	SOX2
PLLP	Enrichr CHEA_2022: SOX2 20726797 ChIP-Seq SW620 Human	SOX2
TIMP2	Enrichr CHEA_2022: SOX2 20726797 ChIP-Seq SW620 Human	SOX2
TEAD2	Enrichr CHEA_2022: SOX2 20726797 ChIP-Seq SW620 Human	SOX2
TEAD3	Enrichr CHEA_2022: SOX2 20726797 ChIP-Seq SW620 Human	SOX2
AP1M2	Enrichr CHEA_2022: SOX2 20726797 ChIP-Seq SW620 Human	SOX2
TEAD4	Enrichr CHEA_2022: SOX2 20726797 ChIP-Seq SW620 Human	SOX2
CEP57	Enrichr CHEA_2022: SOX2 20726797 ChIP-Seq SW620 Human	SOX2
FARP2	Enrichr CHEA_2022: SOX2 20726797 ChIP-Seq SW620 Human	SOX2
CDKN2C	Enrichr CHEA_2022: SOX2 20726797 ChIP-Seq SW620 Human	SOX2
MBNL2	Enrichr CHEA_2022: SOX2 20726797 ChIP-Seq SW620 Human	SOX2
YTHDF3	Enrichr CHEA_2022: SOX2 20726797 ChIP-Seq SW620 Human	SOX2
MGMT	Enrichr CHEA_2022: SOX2 20726797 ChIP-Seq SW620 Human	SOX2
CDKN2B	Enrichr CHEA_2022: SOX2 20726797 ChIP-Seq SW620 Human	SOX2
G0S2	Enrichr CHEA_2022: SOX2 20726797 ChIP-Seq SW620 Human	SOX2
NR1H3	Enrichr CHEA_2022: SOX2 20726797 ChIP-Seq SW620 Human	SOX2
ELP3	Enrichr CHEA_2022: SOX2 20726797 ChIP-Seq SW620 Human	SOX2
NFIA	Enrichr CHEA_2022: SOX2 20726797 ChIP-Seq SW620 Human	SOX2
DHRS2	Enrichr CHEA_2022: SOX2 20726797 ChIP-Seq SW620 Human	SOX2
DHRS3	Enrichr CHEA_2022: SOX2 20726797 ChIP-Seq SW620 Human	SOX2
FARP1	Enrichr CHEA_2022: SOX2 20726797 ChIP-Seq SW620 Human	SOX2
ZNF33B	Enrichr CHEA_2022: SOX2 20726797 ChIP-Seq SW620 Human	SOX2
DHRS9	Enrichr CHEA_2022: SOX2 20726797 ChIP-Seq SW620 Human	SOX2
NFIB	Enrichr CHEA_2022: SOX2 20726797 ChIP-Seq SW620 Human	SOX2
PLEKHO1	Enrichr CHEA_2022: SOX2 20726797 ChIP-Seq SW620 Human	SOX2
SMOC2	Enrichr CHEA_2022: SOX2 20726797 ChIP-Seq SW620 Human	SOX2
MAP1B	Enrichr CHEA_2022: SOX2 20726797 ChIP-Seq SW620 Human	SOX2
NUCB2	Enrichr CHEA_2022: SOX2 20726797 ChIP-Seq SW620 Human	SOX2
ZNF331	Enrichr CHEA_2022: SOX2 20726797 ChIP-Seq SW620 Human	SOX2
PHF6	Enrichr CHEA_2022: SOX2 20726797 ChIP-Seq SW620 Human	SOX2
CDKN1C	Enrichr CHEA_2022: SOX2 20726797 ChIP-Seq SW620 Human	SOX2
GRN	Enrichr CHEA_2022: SOX2 20726797 ChIP-Seq SW620 Human	SOX2
PYGB	Enrichr CHEA_2022: SOX2 20726797 ChIP-Seq SW620 Human	SOX2
SETD7	Enrichr CHEA_2022: SOX2 20726797 ChIP-Seq SW620 Human	SOX2
PYGL	Enrichr CHEA_2022: SOX2 20726797 ChIP-Seq SW620 Human	SOX2
STON2	Enrichr CHEA_2022: SOX2 20726797 ChIP-Seq SW620 Human	SOX2
PDF	Enrichr CHEA_2022: SOX2 20726797 ChIP-Seq SW620 Human	SOX2
PPP1R2	Enrichr CHEA_2022: SOX2 20726797 ChIP-Seq SW620 Human	SOX2
UBL3	Enrichr CHEA_2022: SOX2 20726797 ChIP-Seq SW620 Human	SOX2
ASH2L	Enrichr CHEA_2022: SOX2 20726797 ChIP-Seq SW620 Human	SOX2
CKB	Enrichr CHEA_2022: SOX2 20726797 ChIP-Seq SW620 Human	SOX2
SLC17A5	Enrichr CHEA_2022: SOX2 20726797 ChIP-Seq SW620 Human	SOX2
GSN	Enrichr CHEA_2022: SOX2 20726797 ChIP-Seq SW620 Human	SOX2
TLE4	Enrichr CHEA_2022: SOX2 20726797 ChIP-Seq SW620 Human	SOX2
TLE1	Enrichr CHEA_2022: SOX2 20726797 ChIP-Seq SW620 Human	SOX2
TPM2	Enrichr CHEA_2022: SOX2 20726797 ChIP-Seq SW620 Human	SOX2
VRK2	Enrichr CHEA_2022: SOX2 20726797 ChIP-Seq SW620 Human	SOX2
VRK3	Enrichr CHEA_2022: SOX2 20726797 ChIP-Seq SW620 Human	SOX2
HIPK2	Enrichr CHEA_2022: SOX2 20726797 ChIP-Seq SW620 Human	SOX2
RAB30	Enrichr CHEA_2022: SOX2 20726797 ChIP-Seq SW620 Human	SOX2
NRBP2	Enrichr CHEA_2022: SOX2 20726797 ChIP-Seq SW620 Human	SOX2

FAM91A1	Enrichr CHEA_2022: SOX2 20726797 ChIP-Seq SW620 Human	SOX2
SLC7A6OS	Enrichr CHEA_2022: SOX2 20726797 ChIP-Seq SW620 Human	SOX2
RAB31	Enrichr CHEA_2022: SOX2 20726797 ChIP-Seq SW620 Human	SOX2
PSAT1	Enrichr CHEA_2022: SOX2 20726797 ChIP-Seq SW620 Human	SOX2
PLXNB2	Enrichr CHEA_2022: SOX2 20726797 ChIP-Seq SW620 Human	SOX2
CSPG5	Enrichr CHEA_2022: SOX2 20726797 ChIP-Seq SW620 Human	SOX2
CNIH3	Enrichr CHEA_2022: SOX2 20726797 ChIP-Seq SW620 Human	SOX2
TCTA	Enrichr CHEA_2022: SOX2 20726797 ChIP-Seq SW620 Human	SOX2
PPIL4	Enrichr CHEA_2022: SOX2 20726797 ChIP-Seq SW620 Human	SOX2
FKBP7	Enrichr CHEA_2022: SOX2 20726797 ChIP-Seq SW620 Human	SOX2
GOLGA8A	Enrichr CHEA_2022: SOX2 20726797 ChIP-Seq SW620 Human	SOX2
GTF2I	Enrichr CHEA_2022: SOX2 20726797 ChIP-Seq SW620 Human	SOX2
RNMT	Enrichr CHEA_2022: SOX2 20726797 ChIP-Seq SW620 Human	SOX2
PROS1	Enrichr CHEA_2022: SOX2 20726797 ChIP-Seq SW620 Human	SOX2
TCEAL1	Enrichr CHEA_2022: SOX2 20726797 ChIP-Seq SW620 Human	SOX2
PAPSS1	Enrichr CHEA_2022: SOX2 20726797 ChIP-Seq SW620 Human	SOX2
PAPSS2	Enrichr CHEA_2022: SOX2 20726797 ChIP-Seq SW620 Human	SOX2
FGD5	Enrichr CHEA_2022: SOX2 20726797 ChIP-Seq SW620 Human	SOX2
TCEAL8	Enrichr CHEA_2022: SOX2 20726797 ChIP-Seq SW620 Human	SOX2
ABLIM1	Enrichr CHEA_2022: SOX2 20726797 ChIP-Seq SW620 Human	SOX2
ABLIM3	Enrichr CHEA_2022: SOX2 20726797 ChIP-Seq SW620 Human	SOX2
CLCC1	Enrichr CHEA_2022: SOX2 20726797 ChIP-Seq SW620 Human	SOX2
TCEAL4	Enrichr CHEA_2022: SOX2 20726797 ChIP-Seq SW620 Human	SOX2
RAB25	Enrichr CHEA_2022: SOX2 20726797 ChIP-Seq SW620 Human	SOX2
SLC39A6	Enrichr CHEA_2022: SOX2 20726797 ChIP-Seq SW620 Human	SOX2
TXNL4B	Enrichr CHEA_2022: SOX2 20726797 ChIP-Seq SW620 Human	SOX2
ZNF302	Enrichr CHEA_2022: SOX2 20726797 ChIP-Seq SW620 Human	SOX2
SIAE	Enrichr CHEA_2022: SOX2 20726797 ChIP-Seq SW620 Human	SOX2
KCMF1	Enrichr CHEA_2022: SOX2 20726797 ChIP-Seq SW620 Human	SOX2
SERF1A	Enrichr CHEA_2022: SOX2 20726797 ChIP-Seq SW620 Human	SOX2
TRAPPC2	Enrichr CHEA_2022: SOX2 20726797 ChIP-Seq SW620 Human	SOX2
OSBPL5	Enrichr CHEA_2022: SOX2 20726797 ChIP-Seq SW620 Human	SOX2
FRMD4B	Enrichr CHEA_2022: SOX2 20726797 ChIP-Seq SW620 Human	SOX2
TFDP1	Enrichr CHEA_2022: SOX2 20726797 ChIP-Seq SW620 Human	SOX2
FRMD4A	Enrichr CHEA_2022: SOX2 20726797 ChIP-Seq SW620 Human	SOX2
TPP2	Enrichr CHEA_2022: SOX2 20726797 ChIP-Seq SW620 Human	SOX2
LHX6	Enrichr CHEA_2022: SOX2 20726797 ChIP-Seq SW620 Human	SOX2
RAB3D	Enrichr CHEA_2022: SOX2 20726797 ChIP-Seq SW620 Human	SOX2
SLC26A2	Enrichr CHEA_2022: SOX2 20726797 ChIP-Seq SW620 Human	SOX2
CBFB	Enrichr CHEA_2022: SOX2 20726797 ChIP-Seq SW620 Human	SOX2
NPEPPS	Enrichr CHEA_2022: SOX2 20726797 ChIP-Seq SW620 Human	SOX2
CDC14B	Enrichr CHEA_2022: SOX2 20726797 ChIP-Seq SW620 Human	SOX2
TM7SF2	Enrichr CHEA_2022: SOX2 20726797 ChIP-Seq SW620 Human	SOX2
TM7SF3	Enrichr CHEA_2022: SOX2 20726797 ChIP-Seq SW620 Human	SOX2
PLXNA3	Enrichr CHEA_2022: SOX2 20726797 ChIP-Seq SW620 Human	SOX2
PI3	Enrichr CHEA_2022: SOX2 20726797 ChIP-Seq SW620 Human	SOX2
PLXNA2	Enrichr CHEA_2022: SOX2 20726797 ChIP-Seq SW620 Human	SOX2
PAPOLA	Enrichr CHEA_2022: SOX2 20726797 ChIP-Seq SW620 Human	SOX2
PLXNA1	Enrichr CHEA_2022: SOX2 20726797 ChIP-Seq SW620 Human	SOX2
PLS3	Enrichr CHEA_2022: SOX2 20726797 ChIP-Seq SW620 Human	SOX2
MAP2K6	Enrichr CHEA_2022: SOX2 20726797 ChIP-Seq SW620 Human	SOX2
CDADC1	Enrichr CHEA_2022: SOX2 20726797 ChIP-Seq SW620 Human	SOX2
RFC4	Enrichr CHEA_2022: SOX2 20726797 ChIP-Seq SW620 Human	SOX2
XCL1	Enrichr CHEA_2022: SOX2 20726797 ChIP-Seq SW620 Human	SOX2
KBTBD11	Enrichr CHEA_2022: SOX2 20726797 ChIP-Seq SW620 Human	SOX2
IRAK1BP1	Enrichr CHEA_2022: SOX2 20726797 ChIP-Seq SW620 Human	SOX2
DAB2	Enrichr CHEA_2022: SOX2 20726797 ChIP-Seq SW620 Human	SOX2
MFAP2	Enrichr CHEA_2022: SOX2 20726797 ChIP-Seq SW620 Human	SOX2
ESPL1	Enrichr CHEA_2022: SOX2 20726797 ChIP-Seq SW620 Human	SOX2

BCL6	Enrichr CHEA_2022: SOX2 20726797 ChIP-Seq SW620 Human	SOX2
DNAJC6	Enrichr CHEA_2022: SOX2 20726797 ChIP-Seq SW620 Human	SOX2
DNAJC5	Enrichr CHEA_2022: SOX2 20726797 ChIP-Seq SW620 Human	SOX2
PIR	Enrichr CHEA_2022: SOX2 20726797 ChIP-Seq SW620 Human	SOX2
XCL2	Enrichr CHEA_2022: SOX2 20726797 ChIP-Seq SW620 Human	SOX2
LAT	Enrichr CHEA_2022: SOX2 20726797 ChIP-Seq SW620 Human	SOX2
BMPR2	Enrichr CHEA_2022: SOX2 20726797 ChIP-Seq SW620 Human	SOX2
CCDC25	Enrichr CHEA_2022: SOX2 20726797 ChIP-Seq SW620 Human	SOX2
HMMR	Enrichr CHEA_2022: SOX2 20726797 ChIP-Seq SW620 Human	SOX2
ZC3H8	Enrichr CHEA_2022: SOX2 20726797 ChIP-Seq SW620 Human	SOX2
UBR1	Enrichr CHEA_2022: SOX2 20726797 ChIP-Seq SW620 Human	SOX2
CYP3A5	Enrichr CHEA_2022: SOX2 20726797 ChIP-Seq SW620 Human	SOX2
CYP3A7	Enrichr CHEA_2022: SOX2 20726797 ChIP-Seq SW620 Human	SOX2
LBH	Enrichr CHEA_2022: SOX2 20726797 ChIP-Seq SW620 Human	SOX2
PSTPIP2	Enrichr CHEA_2022: SOX2 20726797 ChIP-Seq SW620 Human	SOX2
P2RY2	Enrichr CHEA_2022: SOX2 20726797 ChIP-Seq SW620 Human	SOX2
DVL2	Enrichr CHEA_2022: SOX2 20726797 ChIP-Seq SW620 Human	SOX2
PHACTR3	Enrichr CHEA_2022: SOX2 20726797 ChIP-Seq SW620 Human	SOX2
PHACTR2	Enrichr CHEA_2022: SOX2 20726797 ChIP-Seq SW620 Human	SOX2
DNAJB9	Enrichr CHEA_2022: SOX2 20726797 ChIP-Seq SW620 Human	SOX2
SERAC1	Enrichr CHEA_2022: SOX2 20726797 ChIP-Seq SW620 Human	SOX2
EMILIN2	Enrichr CHEA_2022: SOX2 20726797 ChIP-Seq SW620 Human	SOX2
CCDC34	Enrichr CHEA_2022: SOX2 20726797 ChIP-Seq SW620 Human	SOX2
TPPP	Enrichr CHEA_2022: SOX2 20726797 ChIP-Seq SW620 Human	SOX2
CEP170	Enrichr CHEA_2022: SOX2 20726797 ChIP-Seq SW620 Human	SOX2
ADSL	Enrichr CHEA_2022: SOX2 20726797 ChIP-Seq SW620 Human	SOX2
PLEKHG6	Enrichr CHEA_2022: SOX2 20726797 ChIP-Seq SW620 Human	SOX2
KRR1	Enrichr CHEA_2022: SOX2 20726797 ChIP-Seq SW620 Human	SOX2
COBL	Enrichr CHEA_2022: SOX2 20726797 ChIP-Seq SW620 Human	SOX2
FBXL16	Enrichr CHEA_2022: SOX2 20726797 ChIP-Seq SW620 Human	SOX2
LEMD1	Enrichr CHEA_2022: SOX2 20726797 ChIP-Seq SW620 Human	SOX2
LCK	Enrichr CHEA_2022: SOX2 20726797 ChIP-Seq SW620 Human	SOX2
P2RX5	Enrichr CHEA_2022: SOX2 20726797 ChIP-Seq SW620 Human	SOX2
RAB15	Enrichr CHEA_2022: SOX2 20726797 ChIP-Seq SW620 Human	SOX2
APOC1	Enrichr CHEA_2022: SOX2 20726797 ChIP-Seq SW620 Human	SOX2
KIAA0586	Enrichr CHEA_2022: SOX2 20726797 ChIP-Seq SW620 Human	SOX2
DNAJA4	Enrichr CHEA_2022: SOX2 20726797 ChIP-Seq SW620 Human	SOX2
POLR1D	Enrichr CHEA_2022: SOX2 20726797 ChIP-Seq SW620 Human	SOX2
APOC2	Enrichr CHEA_2022: SOX2 20726797 ChIP-Seq SW620 Human	SOX2
POLR1E	Enrichr CHEA_2022: SOX2 20726797 ChIP-Seq SW620 Human	SOX2
CCDC66	Enrichr CHEA_2022: SOX2 20726797 ChIP-Seq SW620 Human	SOX2
PLEKHG1	Enrichr CHEA_2022: SOX2 20726797 ChIP-Seq SW620 Human	SOX2
HDAC4	Enrichr CHEA_2022: SOX2 20726797 ChIP-Seq SW620 Human	SOX2
CEBPD	Enrichr CHEA_2022: SOX2 20726797 ChIP-Seq SW620 Human	SOX2
MCM8	Enrichr CHEA_2022: SOX2 20726797 ChIP-Seq SW620 Human	SOX2
WDR4	Enrichr CHEA_2022: SOX2 20726797 ChIP-Seq SW620 Human	SOX2
TTL	Enrichr CHEA_2022: SOX2 20726797 ChIP-Seq SW620 Human	SOX2
THSD1	Enrichr CHEA_2022: SOX2 20726797 ChIP-Seq SW620 Human	SOX2
NOB1	Enrichr CHEA_2022: SOX2 20726797 ChIP-Seq SW620 Human	SOX2
TTK	Enrichr CHEA_2022: SOX2 20726797 ChIP-Seq SW620 Human	SOX2
HDAC9	Enrichr CHEA_2022: SOX2 20726797 ChIP-Seq SW620 Human	SOX2
SYNE2	Enrichr CHEA_2022: SOX2 20726797 ChIP-Seq SW620 Human	SOX2
THSD4	Enrichr CHEA_2022: SOX2 20726797 ChIP-Seq SW620 Human	SOX2
SCRN2	Enrichr CHEA_2022: SOX2 20726797 ChIP-Seq SW620 Human	SOX2
SCRN3	Enrichr CHEA_2022: SOX2 20726797 ChIP-Seq SW620 Human	SOX2
UGT1A6	Enrichr CHEA_2022: SOX2 20726797 ChIP-Seq SW620 Human	SOX2
CCDC51	Enrichr CHEA_2022: SOX2 20726797 ChIP-Seq SW620 Human	SOX2
UGT1A8	Enrichr CHEA_2022: SOX2 20726797 ChIP-Seq SW620 Human	SOX2
IFRD1	Enrichr CHEA_2022: SOX2 20726797 ChIP-Seq SW620 Human	SOX2

IL1R2	Enrichr CHEA_2022: SOX2 20726797 ChIP-Seq SW620 Human	SOX2
KRT7	Enrichr CHEA_2022: SOX2 20726797 ChIP-Seq SW620 Human	SOX2
CRNKL1	Enrichr CHEA_2022: SOX2 20726797 ChIP-Seq SW620 Human	SOX2
IL23A	Enrichr CHEA_2022: SOX2 20726797 ChIP-Seq SW620 Human	SOX2
KATNAL1	Enrichr CHEA_2022: SOX2 20726797 ChIP-Seq SW620 Human	SOX2
NEIL2	Enrichr CHEA_2022: SOX2 20726797 ChIP-Seq SW620 Human	SOX2
OBSL1	Enrichr CHEA_2022: SOX2 20726797 ChIP-Seq SW620 Human	SOX2
DCP1B	Enrichr CHEA_2022: SOX2 20726797 ChIP-Seq SW620 Human	SOX2
IFITM1	Enrichr CHEA_2022: SOX2 20726797 ChIP-Seq SW620 Human	SOX2
CBLC	Enrichr CHEA_2022: SOX2 20726797 ChIP-Seq SW620 Human	SOX2
CALML4	Enrichr CHEA_2022: SOX2 20726797 ChIP-Seq SW620 Human	SOX2
AQP5	Enrichr CHEA_2022: SOX2 20726797 ChIP-Seq SW620 Human	SOX2
AQP3	Enrichr CHEA_2022: SOX2 20726797 ChIP-Seq SW620 Human	SOX2
APCDD1	Enrichr CHEA_2022: SOX2 20726797 ChIP-Seq SW620 Human	SOX2
MTHFD1L	Enrichr CHEA_2022: SOX2 20726797 ChIP-Seq SW620 Human	SOX2
STXBP6	Enrichr CHEA_2022: SOX2 20726797 ChIP-Seq SW620 Human	SOX2
RFFL	Enrichr CHEA_2022: SOX2 20726797 ChIP-Seq SW620 Human	SOX2
HLA-E	Enrichr CHEA_2022: SOX2 20726797 ChIP-Seq SW620 Human	SOX2
VASP	Enrichr CHEA_2022: SOX2 20726797 ChIP-Seq SW620 Human	SOX2
VNN1	Enrichr CHEA_2022: SOX2 20726797 ChIP-Seq SW620 Human	SOX2
SMTN	Enrichr CHEA_2022: SOX2 20726797 ChIP-Seq SW620 Human	SOX2
SPRR1A	Enrichr CHEA_2022: SOX2 20726797 ChIP-Seq SW620 Human	SOX2
RPS23	Enrichr CHEA_2022: SOX2 20726797 ChIP-Seq SW620 Human	SOX2
SIM2	Enrichr CHEA_2022: SOX2 20726797 ChIP-Seq SW620 Human	SOX2
SPRR1B	Enrichr CHEA_2022: SOX2 20726797 ChIP-Seq SW620 Human	SOX2
CFTR	Enrichr CHEA_2022: SOX2 20726797 ChIP-Seq SW620 Human	SOX2
IL1RN	Enrichr CHEA_2022: SOX2 20726797 ChIP-Seq SW620 Human	SOX2
MAGED2	Enrichr CHEA_2022: SOX2 20726797 ChIP-Seq SW620 Human	SOX2
DCUN1D4	Enrichr CHEA_2022: SOX2 20726797 ChIP-Seq SW620 Human	SOX2
FAM89A	Enrichr CHEA_2022: SOX2 20726797 ChIP-Seq SW620 Human	SOX2
PPL	Enrichr CHEA_2022: SOX2 20726797 ChIP-Seq SW620 Human	SOX2
IFIT2	Enrichr CHEA_2022: SOX2 20726797 ChIP-Seq SW620 Human	SOX2
IFIT1	Enrichr CHEA_2022: SOX2 20726797 ChIP-Seq SW620 Human	SOX2
TRPM2	Enrichr CHEA_2022: SOX2 20726797 ChIP-Seq SW620 Human	SOX2
PDK4	Enrichr CHEA_2022: SOX2 20726797 ChIP-Seq SW620 Human	SOX2
PDK1	Enrichr CHEA_2022: SOX2 20726797 ChIP-Seq SW620 Human	SOX2
RALGPS2	Enrichr CHEA_2022: SOX2 20726797 ChIP-Seq SW620 Human	SOX2
TRPM4	Enrichr CHEA_2022: SOX2 20726797 ChIP-Seq SW620 Human	SOX2
PDK3	Enrichr CHEA_2022: SOX2 20726797 ChIP-Seq SW620 Human	SOX2
PSENEN	Enrichr CHEA_2022: SOX2 20726797 ChIP-Seq SW620 Human	SOX2
EDN1	Enrichr CHEA_2022: SOX2 20726797 ChIP-Seq SW620 Human	SOX2
HEG1	Enrichr CHEA_2022: SOX2 20726797 ChIP-Seq SW620 Human	SOX2
UBL4A	Enrichr CHEA_2022: SOX2 20726797 ChIP-Seq SW620 Human	SOX2
MLF1	Enrichr CHEA_2022: SOX2 20726797 ChIP-Seq SW620 Human	SOX2
MTSS1	Enrichr CHEA_2022: SOX2 20726797 ChIP-Seq SW620 Human	SOX2
CLDN11	Enrichr CHEA_2022: SOX2 20726797 ChIP-Seq SW620 Human	SOX2
KBTBD7	Enrichr CHEA_2022: SOX2 20726797 ChIP-Seq SW620 Human	SOX2
P4HA2	Enrichr CHEA_2022: SOX2 20726797 ChIP-Seq SW620 Human	SOX2
VWA1	Enrichr CHEA_2022: SOX2 20726797 ChIP-Seq SW620 Human	SOX2
TLR4	Enrichr CHEA_2022: SOX2 20726797 ChIP-Seq SW620 Human	SOX2
RAB7B	Enrichr CHEA_2022: SOX2 20726797 ChIP-Seq SW620 Human	SOX2
SLC44A4	Enrichr CHEA_2022: SOX2 20726797 ChIP-Seq SW620 Human	SOX2
CITED1	Enrichr CHEA_2022: SOX2 20726797 ChIP-Seq SW620 Human	SOX2
RTN4R	Enrichr CHEA_2022: SOX2 20726797 ChIP-Seq SW620 Human	SOX2
SLC44A2	Enrichr CHEA_2022: SOX2 20726797 ChIP-Seq SW620 Human	SOX2
FAM43A	Enrichr CHEA_2022: SOX2 20726797 ChIP-Seq SW620 Human	SOX2
CAB39L	Enrichr CHEA_2022: SOX2 20726797 ChIP-Seq SW620 Human	SOX2
SLC44A1	Enrichr CHEA_2022: SOX2 20726797 ChIP-Seq SW620 Human	SOX2
CHRAC1	Enrichr CHEA_2022: SOX2 20726797 ChIP-Seq SW620 Human	SOX2

SLC22A15	Enrichr CHEA_2022: SOX2 20726797 ChIP-Seq SW620 Human	SOX2
UGCG	Enrichr CHEA_2022: SOX2 20726797 ChIP-Seq SW620 Human	SOX2
SLC22A18	Enrichr CHEA_2022: SOX2 20726797 ChIP-Seq SW620 Human	SOX2
C1QBP	Enrichr CHEA_2022: SOX2 20726797 ChIP-Seq SW620 Human	SOX2
TMED3	Enrichr CHEA_2022: SOX2 20726797 ChIP-Seq SW620 Human	SOX2
TMED8	Enrichr CHEA_2022: SOX2 20726797 ChIP-Seq SW620 Human	SOX2
TMED5	Enrichr CHEA_2022: SOX2 20726797 ChIP-Seq SW620 Human	SOX2
MYEF2	Enrichr CHEA_2022: SOX2 20726797 ChIP-Seq SW620 Human	SOX2
PARP4	Enrichr CHEA_2022: SOX2 20726797 ChIP-Seq SW620 Human	SOX2
CBR1	Enrichr CHEA_2022: SOX2 20726797 ChIP-Seq SW620 Human	SOX2
PARP2	Enrichr CHEA_2022: SOX2 20726797 ChIP-Seq SW620 Human	SOX2
METTL3	Enrichr CHEA_2022: SOX2 20726797 ChIP-Seq SW620 Human	SOX2
PROX1	Enrichr CHEA_2022: SOX2 20726797 ChIP-Seq SW620 Human	SOX2
SORBS1	Enrichr CHEA_2022: SOX2 20726797 ChIP-Seq SW620 Human	SOX2
QPCT	Enrichr CHEA_2022: SOX2 20726797 ChIP-Seq SW620 Human	SOX2
SORBS2	Enrichr CHEA_2022: SOX2 20726797 ChIP-Seq SW620 Human	SOX2
RAB11A	Enrichr CHEA_2022: SOX2 20726797 ChIP-Seq SW620 Human	SOX2
GFRA3	Enrichr CHEA_2022: SOX2 20726797 ChIP-Seq SW620 Human	SOX2
LIMA1	Enrichr CHEA_2022: SOX2 20726797 ChIP-Seq SW620 Human	SOX2
PARP9	Enrichr CHEA_2022: SOX2 20726797 ChIP-Seq SW620 Human	SOX2
PDP2	Enrichr CHEA_2022: SOX2 20726797 ChIP-Seq SW620 Human	SOX2
ALDH5A1	Enrichr CHEA_2022: SOX2 20726797 ChIP-Seq SW620 Human	SOX2
GDPD1	Enrichr CHEA_2022: SOX2 20726797 ChIP-Seq SW620 Human	SOX2
RAB9A	Enrichr CHEA_2022: SOX2 20726797 ChIP-Seq SW620 Human	SOX2
CSTB	Enrichr CHEA_2022: SOX2 20726797 ChIP-Seq SW620 Human	SOX2
GBE1	Enrichr CHEA_2022: SOX2 20726797 ChIP-Seq SW620 Human	SOX2
SIPA1L3	Enrichr CHEA_2022: SOX2 20726797 ChIP-Seq SW620 Human	SOX2
CST7	Enrichr CHEA_2022: SOX2 20726797 ChIP-Seq SW620 Human	SOX2
ROBO1	Enrichr CHEA_2022: SOX2 20726797 ChIP-Seq SW620 Human	SOX2
CST6	Enrichr CHEA_2022: SOX2 20726797 ChIP-Seq SW620 Human	SOX2
NKAP	Enrichr CHEA_2022: SOX2 20726797 ChIP-Seq SW620 Human	SOX2
RPAIN	Enrichr CHEA_2022: SOX2 20726797 ChIP-Seq SW620 Human	SOX2
SIPA1L1	Enrichr CHEA_2022: SOX2 20726797 ChIP-Seq SW620 Human	SOX2
SIPA1L2	Enrichr CHEA_2022: SOX2 20726797 ChIP-Seq SW620 Human	SOX2
SOSTDC1	Enrichr CHEA_2022: SOX2 20726797 ChIP-Seq SW620 Human	SOX2
LMO2	Enrichr CHEA_2022: SOX2 20726797 ChIP-Seq SW620 Human	SOX2
GABRP	Enrichr CHEA_2022: SOX2 20726797 ChIP-Seq SW620 Human	SOX2
LMO4	Enrichr CHEA_2022: SOX2 20726797 ChIP-Seq SW620 Human	SOX2
TRPV2	Enrichr CHEA_2022: SOX2 20726797 ChIP-Seq SW620 Human	SOX2
GRHL2	Enrichr CHEA_2022: SOX2 20726797 ChIP-Seq SW620 Human	SOX2
ATP1B1	Enrichr CHEA_2022: SOX2 20726797 ChIP-Seq SW620 Human	SOX2
TATDN1	Enrichr CHEA_2022: SOX2 20726797 ChIP-Seq SW620 Human	SOX2
AIG1	Enrichr CHEA_2022: SOX2 20726797 ChIP-Seq SW620 Human	SOX2
GRHL1	Enrichr CHEA_2022: SOX2 20726797 ChIP-Seq SW620 Human	SOX2
BAMBI	Enrichr CHEA_2022: SOX2 20726797 ChIP-Seq SW620 Human	SOX2
FANCD2	Enrichr CHEA_2022: SOX2 20726797 ChIP-Seq SW620 Human	SOX2
TRPV6	Enrichr CHEA_2022: SOX2 20726797 ChIP-Seq SW620 Human	SOX2
CRYL1	Enrichr CHEA_2022: SOX2 20726797 ChIP-Seq SW620 Human	SOX2
CHMP4B	Enrichr CHEA_2022: SOX2 20726797 ChIP-Seq SW620 Human	SOX2
MYADM	Enrichr CHEA_2022: SOX2 20726797 ChIP-Seq SW620 Human	SOX2
BRWD1	Enrichr CHEA_2022: SOX2 20726797 ChIP-Seq SW620 Human	SOX2
LRP10	Enrichr CHEA_2022: SOX2 20726797 ChIP-Seq SW620 Human	SOX2
MPHOSPH6	Enrichr CHEA_2022: SOX2 20726797 ChIP-Seq SW620 Human	SOX2
XPR1	Enrichr CHEA_2022: SOX2 20726797 ChIP-Seq SW620 Human	SOX2
PVR	Enrichr CHEA_2022: SOX2 20726797 ChIP-Seq SW620 Human	SOX2
FBLN1	Enrichr CHEA_2022: SOX2 20726797 ChIP-Seq SW620 Human	SOX2
NKD1	Enrichr CHEA_2022: SOX2 20726797 ChIP-Seq SW620 Human	SOX2
COMT	Enrichr CHEA_2022: SOX2 20726797 ChIP-Seq SW620 Human	SOX2
CNDP2	Enrichr CHEA_2022: SOX2 20726797 ChIP-Seq SW620 Human	SOX2

HOXA13	Enrichr CHEA_2022: SOX2 20726797 ChIP-Seq SW620 Human	SOX2
LMNA	Enrichr CHEA_2022: SOX2 20726797 ChIP-Seq SW620 Human	SOX2
DOCK11	Enrichr CHEA_2022: SOX2 20726797 ChIP-Seq SW620 Human	SOX2
NT5E	Enrichr CHEA_2022: SOX2 20726797 ChIP-Seq SW620 Human	SOX2
PURB	Enrichr CHEA_2022: SOX2 20726797 ChIP-Seq SW620 Human	SOX2
KISS1R	Enrichr CHEA_2022: SOX2 20726797 ChIP-Seq SW620 Human	SOX2
GAS2L1	Enrichr CHEA_2022: SOX2 20726797 ChIP-Seq SW620 Human	SOX2
EIF2S3	Enrichr CHEA_2022: SOX2 20726797 ChIP-Seq SW620 Human	SOX2
EIF2S1	Enrichr CHEA_2022: SOX2 20726797 ChIP-Seq SW620 Human	SOX2
DIAPH1	Enrichr CHEA_2022: SOX2 20726797 ChIP-Seq SW620 Human	SOX2
EFNA4	Enrichr CHEA_2022: SOX2 20726797 ChIP-Seq SW620 Human	SOX2
PRPF8	Enrichr CHEA_2022: SOX2 20726797 ChIP-Seq SW620 Human	SOX2
COL6A1	Enrichr CHEA_2022: SOX2 20726797 ChIP-Seq SW620 Human	SOX2
DDAH1	Enrichr CHEA_2022: SOX2 20726797 ChIP-Seq SW620 Human	SOX2
CDH13	Enrichr CHEA_2022: SOX2 20726797 ChIP-Seq SW620 Human	SOX2
FGFR1	Enrichr CHEA_2022: SOX2 20726797 ChIP-Seq SW620 Human	SOX2
CHMP2A	Enrichr CHEA_2022: SOX2 20726797 ChIP-Seq SW620 Human	SOX2
FGFR3	Enrichr CHEA_2022: SOX2 20726797 ChIP-Seq SW620 Human	SOX2
ZCCHC2	Enrichr CHEA_2022: SOX2 20726797 ChIP-Seq SW620 Human	SOX2
FGFR2	Enrichr CHEA_2022: SOX2 20726797 ChIP-Seq SW620 Human	SOX2
PXK	Enrichr CHEA_2022: SOX2 20726797 ChIP-Seq SW620 Human	SOX2
LZIC	Enrichr CHEA_2022: SOX2 20726797 ChIP-Seq SW620 Human	SOX2
RASEF	Enrichr CHEA_2022: SOX2 20726797 ChIP-Seq SW620 Human	SOX2
SPPL2A	Enrichr CHEA_2022: SOX2 20726797 ChIP-Seq SW620 Human	SOX2
RBMX2	Enrichr CHEA_2022: SOX2 20726797 ChIP-Seq SW620 Human	SOX2
EME2	Enrichr CHEA_2022: SOX2 20726797 ChIP-Seq SW620 Human	SOX2
PMAIP1	Enrichr CHEA_2022: SOX2 20726797 ChIP-Seq SW620 Human	SOX2
MLKL	Enrichr CHEA_2022: SOX2 20726797 ChIP-Seq SW620 Human	SOX2
SACS	Enrichr CHEA_2022: SOX2 20726797 ChIP-Seq SW620 Human	SOX2
CYP2J2	Enrichr CHEA_2022: SOX2 20726797 ChIP-Seq SW620 Human	SOX2
CBX6	Enrichr CHEA_2022: SOX2 20726797 ChIP-Seq SW620 Human	SOX2
CBX4	Enrichr CHEA_2022: SOX2 20726797 ChIP-Seq SW620 Human	SOX2
CBX2	Enrichr CHEA_2022: SOX2 20726797 ChIP-Seq SW620 Human	SOX2
PARVB	Enrichr CHEA_2022: SOX2 20726797 ChIP-Seq SW620 Human	SOX2
IQCE	Enrichr CHEA_2022: SOX2 20726797 ChIP-Seq SW620 Human	SOX2
OAS1	Enrichr CHEA_2022: SOX2 20726797 ChIP-Seq SW620 Human	SOX2
DNM1	Enrichr CHEA_2022: SOX2 20726797 ChIP-Seq SW620 Human	SOX2
RBKS	Enrichr CHEA_2022: SOX2 20726797 ChIP-Seq SW620 Human	SOX2
RASA1	Enrichr CHEA_2022: SOX2 20726797 ChIP-Seq SW620 Human	SOX2
RABEP1	Enrichr CHEA_2022: SOX2 20726797 ChIP-Seq SW620 Human	SOX2
RASA4	Enrichr CHEA_2022: SOX2 20726797 ChIP-Seq SW620 Human	SOX2
OAS3	Enrichr CHEA_2022: SOX2 20726797 ChIP-Seq SW620 Human	SOX2
AKT1S1	Enrichr CHEA_2022: SOX2 20726797 ChIP-Seq SW620 Human	SOX2
BLMH	Enrichr CHEA_2022: SOX2 20726797 ChIP-Seq SW620 Human	SOX2
DNASE2	Enrichr CHEA_2022: SOX2 20726797 ChIP-Seq SW620 Human	SOX2
TERF2IP	Enrichr CHEA_2022: SOX2 20726797 ChIP-Seq SW620 Human	SOX2
TRPS1	Enrichr CHEA_2022: SOX2 20726797 ChIP-Seq SW620 Human	SOX2
SLC40A1	Enrichr CHEA_2022: SOX2 20726797 ChIP-Seq SW620 Human	SOX2
ERICH1	Enrichr CHEA_2022: SOX2 20726797 ChIP-Seq SW620 Human	SOX2
FAM83H	Enrichr CHEA_2022: SOX2 20726797 ChIP-Seq SW620 Human	SOX2
WNT11	Enrichr CHEA_2022: SOX2 20726797 ChIP-Seq SW620 Human	SOX2
CASP6	Enrichr CHEA_2022: SOX2 20726797 ChIP-Seq SW620 Human	SOX2
CASP7	Enrichr CHEA_2022: SOX2 20726797 ChIP-Seq SW620 Human	SOX2
CASP4	Enrichr CHEA_2022: SOX2 20726797 ChIP-Seq SW620 Human	SOX2
SNAPC5	Enrichr CHEA_2022: SOX2 20726797 ChIP-Seq SW620 Human	SOX2
FAM83A	Enrichr CHEA_2022: SOX2 20726797 ChIP-Seq SW620 Human	SOX2
MYEOV	Enrichr CHEA_2022: SOX2 20726797 ChIP-Seq SW620 Human	SOX2
SNAPC1	Enrichr CHEA_2022: SOX2 20726797 ChIP-Seq SW620 Human	SOX2
LONRF1	Enrichr CHEA_2022: SOX2 20726797 ChIP-Seq SW620 Human	SOX2

FAM83B	Enrichr CHEA_2022: SOX2 20726797 ChIP-Seq SW620 Human	SOX2
RHBDF1	Enrichr CHEA_2022: SOX2 20726797 ChIP-Seq SW620 Human	SOX2
IL15RA	Enrichr CHEA_2022: SOX2 20726797 ChIP-Seq SW620 Human	SOX2
TMEM30B	Enrichr CHEA_2022: SOX2 20726797 ChIP-Seq SW620 Human	SOX2
PLAUR	Enrichr CHEA_2022: SOX2 20726797 ChIP-Seq SW620 Human	SOX2
LSR	Enrichr CHEA_2022: SOX2 20726797 ChIP-Seq SW620 Human	SOX2
ANXA10	Enrichr CHEA_2022: SOX2 20726797 ChIP-Seq SW620 Human	SOX2
TIAM1	Enrichr CHEA_2022: SOX2 20726797 ChIP-Seq SW620 Human	SOX2
MLPH	Enrichr CHEA_2022: SOX2 20726797 ChIP-Seq SW620 Human	SOX2
TRAPPC6A	Enrichr CHEA_2022: SOX2 20726797 ChIP-Seq SW620 Human	SOX2
ALDH1A1	Enrichr CHEA_2022: SOX2 20726797 ChIP-Seq SW620 Human	SOX2
PPP2R2C	Enrichr CHEA_2022: SOX2 20726797 ChIP-Seq SW620 Human	SOX2
ALDH1A3	Enrichr CHEA_2022: SOX2 20726797 ChIP-Seq SW620 Human	SOX2
DDIT4	Enrichr CHEA_2022: SOX2 20726797 ChIP-Seq SW620 Human	SOX2
DDIT3	Enrichr CHEA_2022: SOX2 20726797 ChIP-Seq SW620 Human	SOX2
CLOCK	Enrichr CHEA_2022: SOX2 20726797 ChIP-Seq SW620 Human	SOX2
SIX1	Enrichr CHEA_2022: SOX2 20726797 ChIP-Seq SW620 Human	SOX2
THBS1	Enrichr CHEA_2022: SOX2 20726797 ChIP-Seq SW620 Human	SOX2
OASL	Enrichr CHEA_2022: SOX2 20726797 ChIP-Seq SW620 Human	SOX2
GNA14	Enrichr CHEA_2022: SOX2 20726797 ChIP-Seq SW620 Human	SOX2
GNA15	Enrichr CHEA_2022: SOX2 20726797 ChIP-Seq SW620 Human	SOX2
HEPH	Enrichr CHEA_2022: SOX2 20726797 ChIP-Seq SW620 Human	SOX2
ARHGDIB	Enrichr CHEA_2022: SOX2 20726797 ChIP-Seq SW620 Human	SOX2
LEPROTL1	Enrichr CHEA_2022: SOX2 20726797 ChIP-Seq SW620 Human	SOX2
RPS6KA2	Enrichr CHEA_2022: SOX2 20726797 ChIP-Seq SW620 Human	SOX2
B3GALNT1	Enrichr CHEA_2022: SOX2 20726797 ChIP-Seq SW620 Human	SOX2
GDAP1	Enrichr CHEA_2022: SOX2 20726797 ChIP-Seq SW620 Human	SOX2
WDHD1	Enrichr CHEA_2022: SOX2 20726797 ChIP-Seq SW620 Human	SOX2
PSMB9	Enrichr CHEA_2022: SOX2 20726797 ChIP-Seq SW620 Human	SOX2
EML4	Enrichr CHEA_2022: SOX2 20726797 ChIP-Seq SW620 Human	SOX2
MYL5	Enrichr CHEA_2022: SOX2 20726797 ChIP-Seq SW620 Human	SOX2
RBP4	Enrichr CHEA_2022: SOX2 20726797 ChIP-Seq SW620 Human	SOX2
RBP1	Enrichr CHEA_2022: SOX2 20726797 ChIP-Seq SW620 Human	SOX2
LETMD1	Enrichr CHEA_2022: SOX2 20726797 ChIP-Seq SW620 Human	SOX2
LEF1	Enrichr CHEA_2022: SOX2 20726797 ChIP-Seq SW620 Human	SOX2
CGGBP1	Enrichr CHEA_2022: SOX2 20726797 ChIP-Seq SW620 Human	SOX2
PDXK	Enrichr CHEA_2022: SOX2 20726797 ChIP-Seq SW620 Human	SOX2
ZNF274	Enrichr CHEA_2022: SOX2 20726797 ChIP-Seq SW620 Human	SOX2
ZNF273	Enrichr CHEA_2022: SOX2 20726797 ChIP-Seq SW620 Human	SOX2
IER5L	Enrichr CHEA_2022: SOX2 20726797 ChIP-Seq SW620 Human	SOX2
FZD10	Enrichr CHEA_2022: SOX2 20726797 ChIP-Seq SW620 Human	SOX2
ARAF	Enrichr CHEA_2022: SOX2 20726797 ChIP-Seq SW620 Human	SOX2
CLDN3	Enrichr CHEA_2022: SOX2 20726797 ChIP-Seq SW620 Human	SOX2
CLDN1	Enrichr CHEA_2022: SOX2 20726797 ChIP-Seq SW620 Human	SOX2
KRTCAP3	Enrichr CHEA_2022: SOX2 20726797 ChIP-Seq SW620 Human	SOX2
PREX1	Enrichr CHEA_2022: SOX2 20726797 ChIP-Seq SW620 Human	SOX2
DNTTIP1	Enrichr CHEA_2022: SOX2 20726797 ChIP-Seq SW620 Human	SOX2
SNCG	Enrichr CHEA_2022: SOX2 20726797 ChIP-Seq SW620 Human	SOX2
STAT6	Enrichr CHEA_2022: SOX2 20726797 ChIP-Seq SW620 Human	SOX2
RABAC1	Enrichr CHEA_2022: SOX2 20726797 ChIP-Seq SW620 Human	SOX2
GBP3	Enrichr CHEA_2022: SOX2 20726797 ChIP-Seq SW620 Human	SOX2
GBP2	Enrichr CHEA_2022: SOX2 20726797 ChIP-Seq SW620 Human	SOX2
HES6	Enrichr CHEA_2022: SOX2 20726797 ChIP-Seq SW620 Human	SOX2
NGFR	Enrichr CHEA_2022: SOX2 20726797 ChIP-Seq SW620 Human	SOX2
MYH7B	Enrichr CHEA_2022: SOX2 20726797 ChIP-Seq SW620 Human	SOX2
KLF12	Enrichr CHEA_2022: SOX2 20726797 ChIP-Seq SW620 Human	SOX2
ZNF264	Enrichr CHEA_2022: SOX2 20726797 ChIP-Seq SW620 Human	SOX2
GABARAPL1	Enrichr CHEA_2022: SOX2 20726797 ChIP-Seq SW620 Human	SOX2
FIBCD1	Enrichr CHEA_2022: SOX2 20726797 ChIP-Seq SW620 Human	SOX2

LATS2	Enrichr CHEA_2022: SOX2 20726797 ChIP-Seq SW620 Human	SOX2
MLLT11	Enrichr CHEA_2022: SOX2 20726797 ChIP-Seq SW620 Human	SOX2
IL22RA1	Enrichr CHEA_2022: SOX2 20726797 ChIP-Seq SW620 Human	SOX2
PRKAR1B	Enrichr CHEA_2022: SOX2 20726797 ChIP-Seq SW620 Human	SOX2
VAMP1	Enrichr CHEA_2022: SOX2 20726797 ChIP-Seq SW620 Human	SOX2
PADI2	Enrichr CHEA_2022: SOX2 20726797 ChIP-Seq SW620 Human	SOX2
VAMP3	Enrichr CHEA_2022: SOX2 20726797 ChIP-Seq SW620 Human	SOX2
MCPH1	Enrichr CHEA_2022: SOX2 20726797 ChIP-Seq SW620 Human	SOX2
PADI1	Enrichr CHEA_2022: SOX2 20726797 ChIP-Seq SW620 Human	SOX2
LXN	Enrichr CHEA_2022: SOX2 20726797 ChIP-Seq SW620 Human	SOX2
SCHIP1	Enrichr CHEA_2022: SOX2 20726797 ChIP-Seq SW620 Human	SOX2
UXS1	Enrichr CHEA_2022: SOX2 20726797 ChIP-Seq SW620 Human	SOX2
MRPL13	Enrichr CHEA_2022: SOX2 20726797 ChIP-Seq SW620 Human	SOX2
TMEM92	Enrichr CHEA_2022: SOX2 20726797 ChIP-Seq SW620 Human	SOX2
MYLK	Enrichr CHEA_2022: SOX2 20726797 ChIP-Seq SW620 Human	SOX2
LIPG	Enrichr CHEA_2022: SOX2 20726797 ChIP-Seq SW620 Human	SOX2
NETO2	Enrichr CHEA_2022: SOX2 20726797 ChIP-Seq SW620 Human	SOX2
GRPEL2	Enrichr CHEA_2022: SOX2 20726797 ChIP-Seq SW620 Human	SOX2
IL20RA	Enrichr CHEA_2022: SOX2 20726797 ChIP-Seq SW620 Human	SOX2
KCNMB4	Enrichr CHEA_2022: SOX2 20726797 ChIP-Seq SW620 Human	SOX2
ZBTB7C	Enrichr CHEA_2022: SOX2 20726797 ChIP-Seq SW620 Human	SOX2
AMZ2	Enrichr CHEA_2022: SOX2 20726797 ChIP-Seq SW620 Human	SOX2
RHBDL2	Enrichr CHEA_2022: SOX2 20726797 ChIP-Seq SW620 Human	SOX2
ANKIB1	Enrichr CHEA_2022: SOX2 20726797 ChIP-Seq SW620 Human	SOX2
HACE1	Enrichr CHEA_2022: SOX2 20726797 ChIP-Seq SW620 Human	SOX2
UPF2	Enrichr CHEA_2022: SOX2 20726797 ChIP-Seq SW620 Human	SOX2
LYN	Enrichr CHEA_2022: SOX2 20726797 ChIP-Seq SW620 Human	SOX2
FGFBP1	Enrichr CHEA_2022: SOX2 20726797 ChIP-Seq SW620 Human	SOX2
RIPK3	Enrichr CHEA_2022: SOX2 20726797 ChIP-Seq SW620 Human	SOX2
NSUN4	Enrichr CHEA_2022: SOX2 20726797 ChIP-Seq SW620 Human	SOX2
EMP3	Enrichr CHEA_2022: SOX2 20726797 ChIP-Seq SW620 Human	SOX2
FAHD2A	Enrichr CHEA_2022: SOX2 20726797 ChIP-Seq SW620 Human	SOX2
TMCO4	Enrichr CHEA_2022: SOX2 20726797 ChIP-Seq SW620 Human	SOX2
TMC5	Enrichr CHEA_2022: SOX2 20726797 ChIP-Seq SW620 Human	SOX2
LYZ	Enrichr CHEA_2022: SOX2 20726797 ChIP-Seq SW620 Human	SOX2
TMC4	Enrichr CHEA_2022: SOX2 20726797 ChIP-Seq SW620 Human	SOX2
GPRC5A	Enrichr CHEA_2022: SOX2 20726797 ChIP-Seq SW620 Human	SOX2
LETM2	Enrichr CHEA_2022: SOX2 20726797 ChIP-Seq SW620 Human	SOX2
PIGA	Enrichr CHEA_2022: SOX2 20726797 ChIP-Seq SW620 Human	SOX2
PIGK	Enrichr CHEA_2022: SOX2 20726797 ChIP-Seq SW620 Human	SOX2
ZNF239	Enrichr CHEA_2022: SOX2 20726797 ChIP-Seq SW620 Human	SOX2
ZNF232	Enrichr CHEA_2022: SOX2 20726797 ChIP-Seq SW620 Human	SOX2
ATF7IP2	Enrichr CHEA_2022: SOX2 20726797 ChIP-Seq SW620 Human	SOX2
ACAT1	Enrichr CHEA_2022: SOX2 20726797 ChIP-Seq SW620 Human	SOX2
IQGAP1	Enrichr CHEA_2022: SOX2 20726797 ChIP-Seq SW620 Human	SOX2
LITAF	Enrichr CHEA_2022: SOX2 20726797 ChIP-Seq SW620 Human	SOX2
IQGAP2	Enrichr CHEA_2022: SOX2 20726797 ChIP-Seq SW620 Human	SOX2
MRPL38	Enrichr CHEA_2022: SOX2 20726797 ChIP-Seq SW620 Human	SOX2
LRRC1	Enrichr CHEA_2022: SOX2 20726797 ChIP-Seq SW620 Human	SOX2
AREG	Enrichr CHEA_2022: SOX2 20726797 ChIP-Seq SW620 Human	SOX2
LRRC34	Enrichr CHEA_2022: SOX2 20726797 ChIP-Seq SW620 Human	SOX2
HSD11B2	Enrichr CHEA_2022: SOX2 20726797 ChIP-Seq SW620 Human	SOX2
UGT8	Enrichr CHEA_2022: SOX2 20726797 ChIP-Seq SW620 Human	SOX2
MYO6	Enrichr CHEA_2022: SOX2 20726797 ChIP-Seq SW620 Human	SOX2
PACSIN3	Enrichr CHEA_2022: SOX2 20726797 ChIP-Seq SW620 Human	SOX2
SH3BGRL3	Enrichr CHEA_2022: SOX2 20726797 ChIP-Seq SW620 Human	SOX2
STAP2	Enrichr CHEA_2022: SOX2 20726797 ChIP-Seq SW620 Human	SOX2
CTBS	Enrichr CHEA_2022: SOX2 20726797 ChIP-Seq SW620 Human	SOX2
CGNL1	Enrichr CHEA_2022: SOX2 20726797 ChIP-Seq SW620 Human	SOX2

ELOVL1	Enrichr CHEA_2022: SOX2 20726797 ChIP-Seq SW620 Human	SOX2
GPR37	Enrichr CHEA_2022: SOX2 20726797 ChIP-Seq SW620 Human	SOX2
NANOS1	Enrichr CHEA_2022: SOX2 20726797 ChIP-Seq SW620 Human	SOX2
AADAT	Enrichr CHEA_2022: SOX2 20726797 ChIP-Seq SW620 Human	SOX2
CSNK2A2	Enrichr CHEA_2022: SOX2 20726797 ChIP-Seq SW620 Human	SOX2
ELOVL5	Enrichr CHEA_2022: SOX2 20726797 ChIP-Seq SW620 Human	SOX2
ARG2	Enrichr CHEA_2022: SOX2 20726797 ChIP-Seq SW620 Human	SOX2
STAT3	Enrichr CHEA_2022: SOX2 20726797 ChIP-Seq SW620 Human	SOX2
TMEM62	Enrichr CHEA_2022: SOX2 20726797 ChIP-Seq SW620 Human	SOX2
TRNT1	Enrichr CHEA_2022: SOX2 20726797 ChIP-Seq SW620 Human	SOX2
ELOVL7	Enrichr CHEA_2022: SOX2 20726797 ChIP-Seq SW620 Human	SOX2
RNASE4	Enrichr CHEA_2022: SOX2 20726797 ChIP-Seq SW620 Human	SOX2
TMEM65	Enrichr CHEA_2022: SOX2 20726797 ChIP-Seq SW620 Human	SOX2
LRG1	Enrichr CHEA_2022: SOX2 20726797 ChIP-Seq SW620 Human	SOX2
F5	Enrichr CHEA_2022: SOX2 20726797 ChIP-Seq SW620 Human	SOX2
FABP5	Enrichr CHEA_2022: SOX2 20726797 ChIP-Seq SW620 Human	SOX2
MRPL52	Enrichr CHEA_2022: SOX2 20726797 ChIP-Seq SW620 Human	SOX2
AADAC	Enrichr CHEA_2022: SOX2 20726797 ChIP-Seq SW620 Human	SOX2
PELI1	Enrichr CHEA_2022: SOX2 20726797 ChIP-Seq SW620 Human	SOX2
GOPC	Enrichr CHEA_2022: SOX2 20726797 ChIP-Seq SW620 Human	SOX2
ZNF697	Enrichr CHEA_2022: SOX2 20726797 ChIP-Seq SW620 Human	SOX2
ZNF211	Enrichr CHEA_2022: SOX2 20726797 ChIP-Seq SW620 Human	SOX2
PIGV	Enrichr CHEA_2022: SOX2 20726797 ChIP-Seq SW620 Human	SOX2
RNASEL	Enrichr CHEA_2022: SOX2 20726797 ChIP-Seq SW620 Human	SOX2
QPRT	Enrichr CHEA_2022: SOX2 20726797 ChIP-Seq SW620 Human	SOX2
F13A1	Enrichr CHEA_2022: SOX2 20726797 ChIP-Seq SW620 Human	SOX2
TMEM51	Enrichr CHEA_2022: SOX2 20726797 ChIP-Seq SW620 Human	SOX2
TMEM54	Enrichr CHEA_2022: SOX2 20726797 ChIP-Seq SW620 Human	SOX2
JPH1	Enrichr CHEA_2022: SOX2 20726797 ChIP-Seq SW620 Human	SOX2
PTGS1	Enrichr CHEA_2022: SOX2 20726797 ChIP-Seq SW620 Human	SOX2
PTGS2	Enrichr CHEA_2022: SOX2 20726797 ChIP-Seq SW620 Human	SOX2
NSDHL	Enrichr CHEA_2022: SOX2 20726797 ChIP-Seq SW620 Human	SOX2
HEY1	Enrichr CHEA_2022: SOX2 20726797 ChIP-Seq SW620 Human	SOX2
TMEM47	Enrichr CHEA_2022: SOX2 20726797 ChIP-Seq SW620 Human	SOX2
ZNF207	Enrichr CHEA_2022: SOX2 20726797 ChIP-Seq SW620 Human	SOX2
MYLIP	Enrichr CHEA_2022: SOX2 20726797 ChIP-Seq SW620 Human	SOX2
AGTPBP1	Enrichr CHEA_2022: SOX2 20726797 ChIP-Seq SW620 Human	SOX2
SEC24B	Enrichr CHEA_2022: SOX2 20726797 ChIP-Seq SW620 Human	SOX2
GK	Enrichr CHEA_2022: SOX2 20726797 ChIP-Seq SW620 Human	SOX2
S100A3	Enrichr CHEA_2022: SOX2 20726797 ChIP-Seq SW620 Human	SOX2
BRPF3	Enrichr CHEA_2022: SOX2 20726797 ChIP-Seq SW620 Human	SOX2
S100A2	Enrichr CHEA_2022: SOX2 20726797 ChIP-Seq SW620 Human	SOX2
CRYBB2	Enrichr CHEA_2022: SOX2 20726797 ChIP-Seq SW620 Human	SOX2
ZBTB10	Enrichr CHEA_2022: SOX2 20726797 ChIP-Seq SW620 Human	SOX2
SAP30	Enrichr CHEA_2022: SOX2 20726797 ChIP-Seq SW620 Human	SOX2
MAPKAPK3	Enrichr CHEA_2022: SOX2 20726797 ChIP-Seq SW620 Human	SOX2
RIMBP2	Enrichr CHEA_2022: SOX2 20726797 ChIP-Seq SW620 Human	SOX2
SQSTM1	Enrichr CHEA_2022: SOX2 20726797 ChIP-Seq SW620 Human	SOX2
SEC24D	Enrichr CHEA_2022: SOX2 20726797 ChIP-Seq SW620 Human	SOX2
ROR1	Enrichr CHEA_2022: SOX2 20726797 ChIP-Seq SW620 Human	SOX2
UTRN	Enrichr CHEA_2022: SOX2 20726797 ChIP-Seq SW620 Human	SOX2
DSC2	Enrichr CHEA_2022: SOX2 20726797 ChIP-Seq SW620 Human	SOX2
UGT1A10	Enrichr CHEA_2022: SOX2 20726797 ChIP-Seq SW620 Human	SOX2
HDDC2	Enrichr CHEA_2022: SOX2 20726797 ChIP-Seq SW620 Human	SOX2
TMF1	Enrichr CHEA_2022: SOX2 20726797 ChIP-Seq SW620 Human	SOX2
UBE2D3	Enrichr CHEA_2022: SOX2 20726797 ChIP-Seq SW620 Human	SOX2
HP	Enrichr CHEA_2022: SOX2 20726797 ChIP-Seq SW620 Human	SOX2
UCP2	Enrichr CHEA_2022: SOX2 20726797 ChIP-Seq SW620 Human	SOX2
DEFB1	Enrichr CHEA_2022: SOX2 20726797 ChIP-Seq SW620 Human	SOX2

CXCL16	Enrichr CHEA_2022: SOX2 20726797 ChIP-Seq SW620 Human	SOX2
SH3BP2	Enrichr CHEA_2022: SOX2 20726797 ChIP-Seq SW620 Human	SOX2
HAS2	Enrichr CHEA_2022: SOX2 20726797 ChIP-Seq SW620 Human	SOX2
SH3BP5	Enrichr CHEA_2022: SOX2 20726797 ChIP-Seq SW620 Human	SOX2
HAS3	Enrichr CHEA_2022: SOX2 20726797 ChIP-Seq SW620 Human	SOX2
SLC16A9	Enrichr CHEA_2022: SOX2 20726797 ChIP-Seq SW620 Human	SOX2
SLC16A4	Enrichr CHEA_2022: SOX2 20726797 ChIP-Seq SW620 Human	SOX2
PITX2	Enrichr CHEA_2022: SOX2 20726797 ChIP-Seq SW620 Human	SOX2
SLC16A5	Enrichr CHEA_2022: SOX2 20726797 ChIP-Seq SW620 Human	SOX2
ATP9A	Enrichr CHEA_2022: SOX2 20726797 ChIP-Seq SW620 Human	SOX2
SLC16A6	Enrichr CHEA_2022: SOX2 20726797 ChIP-Seq SW620 Human	SOX2
SLC38A2	Enrichr CHEA_2022: SOX2 20726797 ChIP-Seq SW620 Human	SOX2
TNFRSF10B	Enrichr CHEA_2022: SOX2 20726797 ChIP-Seq SW620 Human	SOX2
FRMD6	Enrichr CHEA_2022: SOX2 20726797 ChIP-Seq SW620 Human	SOX2
CDC42EP5	Enrichr CHEA_2022: SOX2 20726797 ChIP-Seq SW620 Human	SOX2
CDC42EP4	Enrichr CHEA_2022: SOX2 20726797 ChIP-Seq SW620 Human	SOX2
BIRC5	Enrichr CHEA_2022: SOX2 20726797 ChIP-Seq SW620 Human	SOX2
FSCN1	Enrichr CHEA_2022: SOX2 20726797 ChIP-Seq SW620 Human	SOX2
CDC42EP2	Enrichr CHEA_2022: SOX2 20726797 ChIP-Seq SW620 Human	SOX2
ZNF655	Enrichr CHEA_2022: SOX2 20726797 ChIP-Seq SW620 Human	SOX2
ZNF654	Enrichr CHEA_2022: SOX2 20726797 ChIP-Seq SW620 Human	SOX2
LRRC57	Enrichr CHEA_2022: SOX2 20726797 ChIP-Seq SW620 Human	SOX2
PFKFB4	Enrichr CHEA_2022: SOX2 20726797 ChIP-Seq SW620 Human	SOX2
GPR87	Enrichr CHEA_2022: SOX2 20726797 ChIP-Seq SW620 Human	SOX2
TACSTD2	Enrichr CHEA_2022: SOX2 20726797 ChIP-Seq SW620 Human	SOX2
SAT2	Enrichr CHEA_2022: SOX2 20726797 ChIP-Seq SW620 Human	SOX2
HNMT	Enrichr CHEA_2022: SOX2 20726797 ChIP-Seq SW620 Human	SOX2
GLIS2	Enrichr CHEA_2022: SOX2 20726797 ChIP-Seq SW620 Human	SOX2
PORCN	Enrichr CHEA_2022: SOX2 20726797 ChIP-Seq SW620 Human	SOX2
SESN2	Enrichr CHEA_2022: SOX2 20726797 ChIP-Seq SW620 Human	SOX2
SESN3	Enrichr CHEA_2022: SOX2 20726797 ChIP-Seq SW620 Human	SOX2
LEPR	Enrichr CHEA_2022: SOX2 20726797 ChIP-Seq SW620 Human	SOX2
MBP	Enrichr CHEA_2022: SOX2 20726797 ChIP-Seq SW620 Human	SOX2
SLC38A5	Enrichr CHEA_2022: SOX2 20726797 ChIP-Seq SW620 Human	SOX2
DGCR2	Enrichr CHEA_2022: SOX2 20726797 ChIP-Seq SW620 Human	SOX2
CYB5B	Enrichr CHEA_2022: SOX2 20726797 ChIP-Seq SW620 Human	SOX2
SETDB2	Enrichr CHEA_2022: SOX2 20726797 ChIP-Seq SW620 Human	SOX2
MCC	Enrichr CHEA_2022: SOX2 20726797 ChIP-Seq SW620 Human	SOX2
CYB5A	Enrichr CHEA_2022: SOX2 20726797 ChIP-Seq SW620 Human	SOX2
QKI	Enrichr CHEA_2022: SOX2 20726797 ChIP-Seq SW620 Human	SOX2
UST	Enrichr CHEA_2022: SOX2 20726797 ChIP-Seq SW620 Human	SOX2
AAK1	Enrichr CHEA_2022: SOX2 20726797 ChIP-Seq SW620 Human	SOX2
SLC25A1	Enrichr CHEA_2022: SOX2 20726797 ChIP-Seq SW620 Human	SOX2
SLC25A4	Enrichr CHEA_2022: SOX2 20726797 ChIP-Seq SW620 Human	SOX2
RAP1GAP	Enrichr CHEA_2022: SOX2 20726797 ChIP-Seq SW620 Human	SOX2
PELP1	Enrichr CHEA_2022: SOX2 20726797 ChIP-Seq SW620 Human	SOX2
FCHO2	Enrichr CHEA_2022: SOX2 20726797 ChIP-Seq SW620 Human	SOX2
DSP	Enrichr CHEA_2022: SOX2 20726797 ChIP-Seq SW620 Human	SOX2
NDUFA8	Enrichr CHEA_2022: SOX2 20726797 ChIP-Seq SW620 Human	SOX2
SEC22A	Enrichr CHEA_2022: SOX2 20726797 ChIP-Seq SW620 Human	SOX2
DST	Enrichr CHEA_2022: SOX2 20726797 ChIP-Seq SW620 Human	SOX2
NDUFA3	Enrichr CHEA_2022: SOX2 20726797 ChIP-Seq SW620 Human	SOX2
LYSMD2	Enrichr CHEA_2022: SOX2 20726797 ChIP-Seq SW620 Human	SOX2
TPD52L1	Enrichr CHEA_2022: SOX2 20726797 ChIP-Seq SW620 Human	SOX2
PPM1A	Enrichr CHEA_2022: SOX2 20726797 ChIP-Seq SW620 Human	SOX2
ZNF618	Enrichr CHEA_2022: SOX2 20726797 ChIP-Seq SW620 Human	SOX2
MET	Enrichr CHEA_2022: SOX2 20726797 ChIP-Seq SW620 Human	SOX2
SEC22C	Enrichr CHEA_2022: SOX2 20726797 ChIP-Seq SW620 Human	SOX2
SLC25A14	Enrichr CHEA_2022: SOX2 20726797 ChIP-Seq SW620 Human	SOX2

EPHB6	Enrichr CHEA_2022: SOX2 20726797 ChIP-Seq SW620 Human	SOX2
FHL1	Enrichr CHEA_2022: SOX2 20726797 ChIP-Seq SW620 Human	SOX2
PIK3CD	Enrichr CHEA_2022: SOX2 20726797 ChIP-Seq SW620 Human	SOX2
PTPRZ1	Enrichr CHEA_2022: SOX2 20726797 ChIP-Seq SW620 Human	SOX2
SLITRK6	Enrichr CHEA_2022: SOX2 20726797 ChIP-Seq SW620 Human	SOX2
UPP1	Enrichr CHEA_2022: SOX2 20726797 ChIP-Seq SW620 Human	SOX2
EPHB2	Enrichr CHEA_2022: SOX2 20726797 ChIP-Seq SW620 Human	SOX2
EPHB4	Enrichr CHEA_2022: SOX2 20726797 ChIP-Seq SW620 Human	SOX2
ACVR1	Enrichr CHEA_2022: SOX2 20726797 ChIP-Seq SW620 Human	SOX2
ZFP1	Enrichr CHEA_2022: SOX2 20726797 ChIP-Seq SW620 Human	SOX2
SLC12A2	Enrichr CHEA_2022: SOX2 20726797 ChIP-Seq SW620 Human	SOX2
PLEKHA6	Enrichr CHEA_2022: SOX2 20726797 ChIP-Seq SW620 Human	SOX2
TM4SF4	Enrichr CHEA_2022: SOX2 20726797 ChIP-Seq SW620 Human	SOX2
EREG	Enrichr CHEA_2022: SOX2 20726797 ChIP-Seq SW620 Human	SOX2
PPP1R3D	Enrichr CHEA_2022: SOX2 20726797 ChIP-Seq SW620 Human	SOX2
IFI27	Enrichr CHEA_2022: SOX2 20726797 ChIP-Seq SW620 Human	SOX2
LACTB2	Enrichr CHEA_2022: SOX2 20726797 ChIP-Seq SW620 Human	SOX2
MXRA7	Enrichr CHEA_2022: SOX2 20726797 ChIP-Seq SW620 Human	SOX2
COL9A3	Enrichr CHEA_2022: SOX2 20726797 ChIP-Seq SW620 Human	SOX2
ALPK3	Enrichr CHEA_2022: SOX2 20726797 ChIP-Seq SW620 Human	SOX2
EPHA1	Enrichr CHEA_2022: SOX2 20726797 ChIP-Seq SW620 Human	SOX2
EPHA4	Enrichr CHEA_2022: SOX2 20726797 ChIP-Seq SW620 Human	SOX2
TMEM87A	Enrichr CHEA_2022: SOX2 20726797 ChIP-Seq SW620 Human	SOX2
SATB1	Enrichr CHEA_2022: SOX2 20726797 ChIP-Seq SW620 Human	SOX2
KDELR2	Enrichr CHEA_2022: SOX2 20726797 ChIP-Seq SW620 Human	SOX2
PEX11A	Enrichr CHEA_2022: SOX2 20726797 ChIP-Seq SW620 Human	SOX2
PEX11B	Enrichr CHEA_2022: SOX2 20726797 ChIP-Seq SW620 Human	SOX2
LRP4	Enrichr CHEA_2022: SOX2 20726797 ChIP-Seq SW620 Human	SOX2
PRCP	Enrichr CHEA_2022: SOX2 20726797 ChIP-Seq SW620 Human	SOX2
CYB5D1	Enrichr CHEA_2022: SOX2 20726797 ChIP-Seq SW620 Human	SOX2
UBE2L6	Enrichr CHEA_2022: SOX2 20726797 ChIP-Seq SW620 Human	SOX2
LRP8	Enrichr CHEA_2022: SOX2 20726797 ChIP-Seq SW620 Human	SOX2
NTN4	Enrichr CHEA_2022: SOX2 20726797 ChIP-Seq SW620 Human	SOX2
CYB5D2	Enrichr CHEA_2022: SOX2 20726797 ChIP-Seq SW620 Human	SOX2
BAIAP2L1	Enrichr CHEA_2022: SOX2 20726797 ChIP-Seq SW620 Human	SOX2
RAB11FIP1	Enrichr CHEA_2022: SOX2 20726797 ChIP-Seq SW620 Human	SOX2
EXOSC6	Enrichr CHEA_2022: SOX2 20726797 ChIP-Seq SW620 Human	SOX2
PARD6B	Enrichr CHEA_2022: SOX2 20726797 ChIP-Seq SW620 Human	SOX2
BAIAP2L2	Enrichr CHEA_2022: SOX2 20726797 ChIP-Seq SW620 Human	SOX2
CHORDC1	Enrichr CHEA_2022: SOX2 20726797 ChIP-Seq SW620 Human	SOX2
TGOLN2	Enrichr CHEA_2022: SOX2 20726797 ChIP-Seq SW620 Human	SOX2
POF1B	Enrichr CHEA_2022: SOX2 20726797 ChIP-Seq SW620 Human	SOX2
RAB11FIP4	Enrichr CHEA_2022: SOX2 20726797 ChIP-Seq SW620 Human	SOX2
WWTR1	Enrichr CHEA_2022: SOX2 20726797 ChIP-Seq SW620 Human	SOX2
PAQR5	Enrichr CHEA_2022: SOX2 20726797 ChIP-Seq SW620 Human	SOX2
FOXF2	Enrichr CHEA_2022: SOX2 20726797 ChIP-Seq SW620 Human	SOX2
ST6GAL1	Enrichr CHEA_2022: SOX2 20726797 ChIP-Seq SW620 Human	SOX2
PAQR3	Enrichr CHEA_2022: SOX2 20726797 ChIP-Seq SW620 Human	SOX2
PLA2G4A	Enrichr CHEA_2022: SOX2 20726797 ChIP-Seq SW620 Human	SOX2
HSPA12A	Enrichr CHEA_2022: SOX2 20726797 ChIP-Seq SW620 Human	SOX2
IFI44	Enrichr CHEA_2022: SOX2 20726797 ChIP-Seq SW620 Human	SOX2
PIK3R1	Enrichr CHEA_2022: SOX2 20726797 ChIP-Seq SW620 Human	SOX2
TAGLN2	Enrichr CHEA_2022: SOX2 20726797 ChIP-Seq SW620 Human	SOX2
RGL2	Enrichr CHEA_2022: SOX2 20726797 ChIP-Seq SW620 Human	SOX2
RGL1	Enrichr CHEA_2022: SOX2 20726797 ChIP-Seq SW620 Human	SOX2
SPON2	Enrichr CHEA_2022: SOX2 20726797 ChIP-Seq SW620 Human	SOX2
IFI35	Enrichr CHEA_2022: SOX2 20726797 ChIP-Seq SW620 Human	SOX2
PRF1	Enrichr CHEA_2022: SOX2 20726797 ChIP-Seq SW620 Human	SOX2
BRCC3	Enrichr CHEA_2022: SOX2 20726797 ChIP-Seq SW620 Human	SOX2

PELO	Enrichr CHEA_2022: SOX2 20726797 ChIP-Seq SW620 Human	SOX2
RASSF3	Enrichr CHEA_2022: SOX2 20726797 ChIP-Seq SW620 Human	SOX2
RASSF5	Enrichr CHEA_2022: SOX2 20726797 ChIP-Seq SW620 Human	SOX2
RASSF6	Enrichr CHEA_2022: SOX2 20726797 ChIP-Seq SW620 Human	SOX2
MICAL2	Enrichr CHEA_2022: SOX2 20726797 ChIP-Seq SW620 Human	SOX2
TSPAN8	Enrichr CHEA_2022: SOX2 20726797 ChIP-Seq SW620 Human	SOX2
ANKH	Enrichr CHEA_2022: SOX2 20726797 ChIP-Seq SW620 Human	SOX2
ARSJ	Enrichr CHEA_2022: SOX2 20726797 ChIP-Seq SW620 Human	SOX2
TSPAN6	Enrichr CHEA_2022: SOX2 20726797 ChIP-Seq SW620 Human	SOX2
ZBED2	Enrichr CHEA_2022: SOX2 20726797 ChIP-Seq SW620 Human	SOX2
KIF21B	Enrichr CHEA_2022: SOX2 20726797 ChIP-Seq SW620 Human	SOX2
TSPAN3	Enrichr CHEA_2022: SOX2 20726797 ChIP-Seq SW620 Human	SOX2
CD14	Enrichr CHEA_2022: SOX2 20726797 ChIP-Seq SW620 Human	SOX2
TSPAN1	Enrichr CHEA_2022: SOX2 20726797 ChIP-Seq SW620 Human	SOX2
ACAD11	Enrichr CHEA_2022: SOX2 20726797 ChIP-Seq SW620 Human	SOX2
PGBD1	Enrichr CHEA_2022: SOX2 20726797 ChIP-Seq SW620 Human	SOX2
ARSD	Enrichr CHEA_2022: SOX2 20726797 ChIP-Seq SW620 Human	SOX2
CTSB	Enrichr CHEA_2022: SOX2 20726797 ChIP-Seq SW620 Human	SOX2
NBPF3	Enrichr CHEA_2022: SOX2 20726797 ChIP-Seq SW620 Human	SOX2
FOXD1	Enrichr CHEA_2022: SOX2 20726797 ChIP-Seq SW620 Human	SOX2
TNFRSF12A	Enrichr CHEA_2022: SOX2 20726797 ChIP-Seq SW620 Human	SOX2
MMEL1	Enrichr CHEA_2022: SOX2 20726797 ChIP-Seq SW620 Human	SOX2
KLK10	Enrichr CHEA_2022: SOX2 20726797 ChIP-Seq SW620 Human	SOX2
KLK11	Enrichr CHEA_2022: SOX2 20726797 ChIP-Seq SW620 Human	SOX2
RGMA	Enrichr CHEA_2022: SOX2 20726797 ChIP-Seq SW620 Human	SOX2
ENAH	Enrichr CHEA_2022: SOX2 20726797 ChIP-Seq SW620 Human	SOX2
NR5A2	Enrichr CHEA_2022: SOX2 20726797 ChIP-Seq SW620 Human	SOX2
PTPRF	Enrichr CHEA_2022: SOX2 20726797 ChIP-Seq SW620 Human	SOX2
SERGEF	Enrichr CHEA_2022: SOX2 20726797 ChIP-Seq SW620 Human	SOX2
TH	Enrichr CHEA_2022: SOX2 20726797 ChIP-Seq SW620 Human	SOX2
PTPRB	Enrichr CHEA_2022: SOX2 20726797 ChIP-Seq SW620 Human	SOX2
VSNL1	Enrichr CHEA_2022: SOX2 20726797 ChIP-Seq SW620 Human	SOX2
ID2	Enrichr CHEA_2022: SOX2 20726797 ChIP-Seq SW620 Human	SOX2
MKX	Enrichr CHEA_2022: SOX2 20726797 ChIP-Seq SW620 Human	SOX2
CD24	Enrichr CHEA_2022: SOX2 20726797 ChIP-Seq SW620 Human	SOX2
FOXC1	Enrichr CHEA_2022: SOX2 20726797 ChIP-Seq SW620 Human	SOX2
CETN2	Enrichr CHEA_2022: SOX2 20726797 ChIP-Seq SW620 Human	SOX2
PTPRO	Enrichr CHEA_2022: SOX2 20726797 ChIP-Seq SW620 Human	SOX2
INTS10	Enrichr CHEA_2022: SOX2 20726797 ChIP-Seq SW620 Human	SOX2
TMPRSS4	Enrichr CHEA_2022: SOX2 20726797 ChIP-Seq SW620 Human	SOX2
CTSZ	Enrichr CHEA_2022: SOX2 20726797 ChIP-Seq SW620 Human	SOX2
KIF15	Enrichr CHEA_2022: SOX2 20726797 ChIP-Seq SW620 Human	SOX2
KIF14	Enrichr CHEA_2022: SOX2 20726797 ChIP-Seq SW620 Human	SOX2
HNF4G	Enrichr CHEA_2022: SOX2 20726797 ChIP-Seq SW620 Human	SOX2
TMPRSS3	Enrichr CHEA_2022: SOX2 20726797 ChIP-Seq SW620 Human	SOX2
PCSK9	Enrichr CHEA_2022: SOX2 20726797 ChIP-Seq SW620 Human	SOX2
NEDD9	Enrichr CHEA_2022: SOX2 20726797 ChIP-Seq SW620 Human	SOX2
PCSK6	Enrichr CHEA_2022: SOX2 20726797 ChIP-Seq SW620 Human	SOX2
CTSS	Enrichr CHEA_2022: SOX2 20726797 ChIP-Seq SW620 Human	SOX2
PTPRH	Enrichr CHEA_2022: SOX2 20726797 ChIP-Seq SW620 Human	SOX2
ACACA	Enrichr CHEA_2022: SOX2 20726797 ChIP-Seq SW620 Human	SOX2
SNX5	Enrichr CHEA_2022: SOX2 20726797 ChIP-Seq SW620 Human	SOX2
SOCS2	Enrichr CHEA_2022: SOX2 20726797 ChIP-Seq SW620 Human	SOX2
SNX1	Enrichr CHEA_2022: SOX2 20726797 ChIP-Seq SW620 Human	SOX2
IDS	Enrichr CHEA_2022: SOX2 20726797 ChIP-Seq SW620 Human	SOX2
CTSL	Enrichr CHEA_2022: SOX2 20726797 ChIP-Seq SW620 Human	SOX2
STK39	Enrichr CHEA_2022: SOX2 20726797 ChIP-Seq SW620 Human	SOX2
CTSH	Enrichr CHEA_2022: SOX2 20726797 ChIP-Seq SW620 Human	SOX2
LMCD1	Enrichr CHEA_2022: SOX2 20726797 ChIP-Seq SW620 Human	SOX2

SNX9	Enrichr CHEA_2022: SOX2 20726797 ChIP-Seq SW620 Human	SOX2
CTSD	Enrichr CHEA_2022: SOX2 20726797 ChIP-Seq SW620 Human	SOX2
SOCS5	Enrichr CHEA_2022: SOX2 20726797 ChIP-Seq SW620 Human	SOX2
SOCS6	Enrichr CHEA_2022: SOX2 20726797 ChIP-Seq SW620 Human	SOX2
MMD	Enrichr CHEA_2022: SOX2 20726797 ChIP-Seq SW620 Human	SOX2
BCAP31	Enrichr CHEA_2022: SOX2 20726797 ChIP-Seq SW620 Human	SOX2
MME	Enrichr CHEA_2022: SOX2 20726797 ChIP-Seq SW620 Human	SOX2
AP2B1	Enrichr CHEA_2022: SOX2 20726797 ChIP-Seq SW620 Human	SOX2
UBE2E2	Enrichr CHEA_2022: SOX2 20726797 ChIP-Seq SW620 Human	SOX2
MIB1	Enrichr CHEA_2022: SOX2 20726797 ChIP-Seq SW620 Human	SOX2
FKBP1B	Enrichr CHEA_2022: SOX2 20726797 ChIP-Seq SW620 Human	SOX2
NEDD1	Enrichr CHEA_2022: SOX2 20726797 ChIP-Seq SW620 Human	SOX2
STK17B	Enrichr CHEA_2022: SOX2 20726797 ChIP-Seq SW620 Human	SOX2
CCNE2	Enrichr CHEA_2022: SOX2 20726797 ChIP-Seq SW620 Human	SOX2
CCNE1	Enrichr CHEA_2022: SOX2 20726797 ChIP-Seq SW620 Human	SOX2
EPPK1	Enrichr CHEA_2022: SOX2 20726797 ChIP-Seq SW620 Human	SOX2
IL17D	Enrichr CHEA_2022: SOX2 20726797 ChIP-Seq SW620 Human	SOX2
CD44	Enrichr CHEA_2022: SOX2 20726797 ChIP-Seq SW620 Human	SOX2
NPDC1	Enrichr CHEA_2022: SOX2 20726797 ChIP-Seq SW620 Human	SOX2
DBN1	Enrichr CHEA_2022: SOX2 20726797 ChIP-Seq SW620 Human	SOX2
FOXA2	Enrichr CHEA_2022: SOX2 20726797 ChIP-Seq SW620 Human	SOX2
ERRFI1	Enrichr CHEA_2022: SOX2 20726797 ChIP-Seq SW620 Human	SOX2
FOXA1	Enrichr CHEA_2022: SOX2 20726797 ChIP-Seq SW620 Human	SOX2
FKBP11	Enrichr CHEA_2022: SOX2 20726797 ChIP-Seq SW620 Human	SOX2
SLC43A3	Enrichr CHEA_2022: SOX2 20726797 ChIP-Seq SW620 Human	SOX2
SLC43A1	Enrichr CHEA_2022: SOX2 20726797 ChIP-Seq SW620 Human	SOX2
FKBP14	Enrichr CHEA_2022: SOX2 20726797 ChIP-Seq SW620 Human	SOX2
AKR1B1	Enrichr CHEA_2022: SOX2 20726797 ChIP-Seq SW620 Human	SOX2
FHOD3	Enrichr CHEA_2022: SOX2 20726797 ChIP-Seq SW620 Human	SOX2
WASL	Enrichr CHEA_2022: SOX2 20726797 ChIP-Seq SW620 Human	SOX2
DERL1	Enrichr CHEA_2022: SOX2 20726797 ChIP-Seq SW620 Human	SOX2
ZDHHC2	Enrichr CHEA_2022: SOX2 20726797 ChIP-Seq SW620 Human	SOX2
CRIP1	Enrichr CHEA_2022: SOX2 20726797 ChIP-Seq SW620 Human	SOX2
CRIP2	Enrichr CHEA_2022: SOX2 20726797 ChIP-Seq SW620 Human	SOX2
SCML1	Enrichr CHEA_2022: SOX2 20726797 ChIP-Seq SW620 Human	SOX2
PGRMC2	Enrichr CHEA_2022: SOX2 20726797 ChIP-Seq SW620 Human	SOX2
SPTLC2	Enrichr CHEA_2022: SOX2 20726797 ChIP-Seq SW620 Human	SOX2
CAMK2N1	Enrichr CHEA_2022: SOX2 20726797 ChIP-Seq SW620 Human	SOX2
SERTAD2	Enrichr CHEA_2022: SOX2 20726797 ChIP-Seq SW620 Human	SOX2
SERTAD3	Enrichr CHEA_2022: SOX2 20726797 ChIP-Seq SW620 Human	SOX2
ENC1	Enrichr CHEA_2022: SOX2 20726797 ChIP-Seq SW620 Human	SOX2
SERTAD1	Enrichr CHEA_2022: SOX2 20726797 ChIP-Seq SW620 Human	SOX2
CD59	Enrichr CHEA_2022: SOX2 20726797 ChIP-Seq SW620 Human	SOX2
CD55	Enrichr CHEA_2022: SOX2 20726797 ChIP-Seq SW620 Human	SOX2
NPNT	Enrichr CHEA_2022: SOX2 20726797 ChIP-Seq SW620 Human	SOX2
G6PD	Enrichr CHEA_2022: SOX2 20726797 ChIP-Seq SW620 Human	SOX2
CRIM1	Enrichr CHEA_2022: SOX2 20726797 ChIP-Seq SW620 Human	SOX2
MID1	Enrichr CHEA_2022: SOX2 20726797 ChIP-Seq SW620 Human	SOX2
TANC2	Enrichr CHEA_2022: SOX2 20726797 ChIP-Seq SW620 Human	SOX2
XK	Enrichr CHEA_2022: SOX2 20726797 ChIP-Seq SW620 Human	SOX2
IVNS1ABP	Enrichr CHEA_2022: SOX2 20726797 ChIP-Seq SW620 Human	SOX2
CD82	Enrichr CHEA_2022: SOX2 20726797 ChIP-Seq SW620 Human	SOX2
SLC1A1	Enrichr CHEA_2022: SOX2 20726797 ChIP-Seq SW620 Human	SOX2
AURKB	Enrichr CHEA_2022: SOX2 20726797 ChIP-Seq SW620 Human	SOX2
RXRA	Enrichr CHEA_2022: SOX2 20726797 ChIP-Seq SW620 Human	SOX2
RAB40B	Enrichr CHEA_2022: SOX2 20726797 ChIP-Seq SW620 Human	SOX2
SH3TC2	Enrichr CHEA_2022: SOX2 20726797 ChIP-Seq SW620 Human	SOX2
HSF2	Enrichr CHEA_2022: SOX2 20726797 ChIP-Seq SW620 Human	SOX2
NPHP3	Enrichr CHEA_2022: SOX2 20726797 ChIP-Seq SW620 Human	SOX2

CCRL2	Enrichr CHEA_2022: SOX2 20726797 ChIP-Seq SW620 Human	SOX2
GPC4	Enrichr CHEA_2022: SOX2 20726797 ChIP-Seq SW620 Human	SOX2
PAK6	Enrichr CHEA_2022: SOX2 20726797 ChIP-Seq SW620 Human	SOX2
MICB	Enrichr CHEA_2022: SOX2 20726797 ChIP-Seq SW620 Human	SOX2
SLC30A7	Enrichr CHEA_2022: SOX2 20726797 ChIP-Seq SW620 Human	SOX2
INHBB	Enrichr CHEA_2022: SOX2 20726797 ChIP-Seq SW620 Human	SOX2
ISG15	Enrichr CHEA_2022: SOX2 20726797 ChIP-Seq SW620 Human	SOX2
SSH3	Enrichr CHEA_2022: SOX2 20726797 ChIP-Seq SW620 Human	SOX2
PAN3	Enrichr CHEA_2022: SOX2 20726797 ChIP-Seq SW620 Human	SOX2
QDPR	Enrichr CHEA_2022: SOX2 20726797 ChIP-Seq SW620 Human	SOX2
PROCR	Enrichr CHEA_2022: SOX2 20726797 ChIP-Seq SW620 Human	SOX2
GEMIN7	Enrichr CHEA_2022: SOX2 20726797 ChIP-Seq SW620 Human	SOX2
TAB3	Enrichr CHEA_2022: SOX2 20726797 ChIP-Seq SW620 Human	SOX2
GCLM	Enrichr CHEA_2022: SOX2 20726797 ChIP-Seq SW620 Human	SOX2
BLVRB	Enrichr CHEA_2022: SOX2 20726797 ChIP-Seq SW620 Human	SOX2
ARHGEF10	Enrichr CHEA_2022: SOX2 20726797 ChIP-Seq SW620 Human	SOX2
LRRFIP1	Enrichr CHEA_2022: SOX2 20726797 ChIP-Seq SW620 Human	SOX2
KLHL15	Enrichr CHEA_2022: SOX2 20726797 ChIP-Seq SW620 Human	SOX2
WWC1	Enrichr CHEA_2022: SOX2 20726797 ChIP-Seq SW620 Human	SOX2
WWC2	Enrichr CHEA_2022: SOX2 20726797 ChIP-Seq SW620 Human	SOX2
RGS3	Enrichr CHEA_2022: SOX2 20726797 ChIP-Seq SW620 Human	SOX2
CALD1	Enrichr CHEA_2022: SOX2 20726797 ChIP-Seq SW620 Human	SOX2
RGS2	Enrichr CHEA_2022: SOX2 20726797 ChIP-Seq SW620 Human	SOX2
NDEL1	Enrichr CHEA_2022: SOX2 20726797 ChIP-Seq SW620 Human	SOX2
CD99	Enrichr CHEA_2022: SOX2 20726797 ChIP-Seq SW620 Human	SOX2
MMP7	Enrichr CHEA_2022: SOX2 20726797 ChIP-Seq SW620 Human	SOX2
GALNT6	Enrichr CHEA_2022: SOX2 20726797 ChIP-Seq SW620 Human	SOX2
GALNT5	Enrichr CHEA_2022: SOX2 20726797 ChIP-Seq SW620 Human	SOX2
GALNT4	Enrichr CHEA_2022: SOX2 20726797 ChIP-Seq SW620 Human	SOX2
GALNT3	Enrichr CHEA_2022: SOX2 20726797 ChIP-Seq SW620 Human	SOX2
HMGA2	Enrichr CHEA_2022: SOX2 20726797 ChIP-Seq SW620 Human	SOX2
GULP1	Enrichr CHEA_2022: SOX2 20726797 ChIP-Seq SW620 Human	SOX2
BMP7	Enrichr CHEA_2022: SOX2 20726797 ChIP-Seq SW620 Human	SOX2
BMP5	Enrichr CHEA_2022: SOX2 20726797 ChIP-Seq SW620 Human	SOX2
IRF1	Enrichr CHEA_2022: SOX2 20726797 ChIP-Seq SW620 Human	SOX2
BMP4	Enrichr CHEA_2022: SOX2 20726797 ChIP-Seq SW620 Human	SOX2
BMP2	Enrichr CHEA_2022: SOX2 20726797 ChIP-Seq SW620 Human	SOX2
MMRN2	Enrichr CHEA_2022: SOX2 20726797 ChIP-Seq SW620 Human	SOX2
RBM20	Enrichr CHEA_2022: SOX2 20726797 ChIP-Seq SW620 Human	SOX2
IRF6	Enrichr CHEA_2022: SOX2 20726797 ChIP-Seq SW620 Human	SOX2
IRF7	Enrichr CHEA_2022: SOX2 20726797 ChIP-Seq SW620 Human	SOX2
TMEM45A	Enrichr CHEA_2022: SOX2 20726797 ChIP-Seq SW620 Human	SOX2
TMEM45B	Enrichr CHEA_2022: SOX2 20726797 ChIP-Seq SW620 Human	SOX2
PSMD11	Enrichr CHEA_2022: SOX2 20726797 ChIP-Seq SW620 Human	SOX2
RTN3	Enrichr CHEA_2022: SOX2 20726797 ChIP-Seq SW620 Human	SOX2
CHD2	Enrichr CHEA_2022: SOX2 20726797 ChIP-Seq SW620 Human	SOX2
FUT2	Enrichr CHEA_2022: SOX2 20726797 ChIP-Seq SW620 Human	SOX2
AFF1	Enrichr CHEA_2022: SOX2 20726797 ChIP-Seq SW620 Human	SOX2
PDLIM1	Enrichr CHEA_2022: SOX2 20726797 ChIP-Seq SW620 Human	SOX2
CALB1	Enrichr CHEA_2022: SOX2 20726797 ChIP-Seq SW620 Human	SOX2
PDLIM4	Enrichr CHEA_2022: SOX2 20726797 ChIP-Seq SW620 Human	SOX2
MARVELD1	Enrichr CHEA_2022: SOX2 20726797 ChIP-Seq SW620 Human	SOX2
CALB2	Enrichr CHEA_2022: SOX2 20726797 ChIP-Seq SW620 Human	SOX2
FUT8	Enrichr CHEA_2022: SOX2 20726797 ChIP-Seq SW620 Human	SOX2
ISOC2	Enrichr CHEA_2022: SOX2 20726797 ChIP-Seq SW620 Human	SOX2
PDLIM5	Enrichr CHEA_2022: SOX2 20726797 ChIP-Seq SW620 Human	SOX2
WNT3	Enrichr CHEA_2022: SOX2 20726797 ChIP-Seq SW620 Human	SOX2
DDX17	Enrichr CHEA_2022: SOX2 20726797 ChIP-Seq SW620 Human	SOX2
TBC1D9	Enrichr CHEA_2022: SOX2 20726797 ChIP-Seq SW620 Human	SOX2

ATP2B4	Enrichr CHEA_2022: SOX2 20726797 ChIP-Seq SW620 Human	SOX2
FNDC3A	Enrichr CHEA_2022: SOX2 20726797 ChIP-Seq SW620 Human	SOX2
FNDC3B	Enrichr CHEA_2022: SOX2 20726797 ChIP-Seq SW620 Human	SOX2
ATP2B1	Enrichr CHEA_2022: SOX2 20726797 ChIP-Seq SW620 Human	SOX2
TBC1D1	Enrichr CHEA_2022: SOX2 20726797 ChIP-Seq SW620 Human	SOX2
TBC1D2	Enrichr CHEA_2022: SOX2 20726797 ChIP-Seq SW620 Human	SOX2
PTHLH	Enrichr CHEA_2022: SOX2 20726797 ChIP-Seq SW620 Human	SOX2
SBK1	Enrichr CHEA_2022: SOX2 20726797 ChIP-Seq SW620 Human	SOX2
TBC1D4	Enrichr CHEA_2022: SOX2 20726797 ChIP-Seq SW620 Human	SOX2
TBC1D5	Enrichr CHEA_2022: SOX2 20726797 ChIP-Seq SW620 Human	SOX2
LNX2	Enrichr CHEA_2022: SOX2 20726797 ChIP-Seq SW620 Human	SOX2
FAM50A	Enrichr CHEA_2022: SOX2 20726797 ChIP-Seq SW620 Human	SOX2
LNX1	Enrichr CHEA_2022: SOX2 20726797 ChIP-Seq SW620 Human	SOX2
S100P	Enrichr CHEA_2022: SOX2 20726797 ChIP-Seq SW620 Human	SOX2
CHURC1	Enrichr CHEA_2022: SOX2 20726797 ChIP-Seq SW620 Human	SOX2
ZP3	Enrichr CHEA_2022: SOX2 20726797 ChIP-Seq SW620 Human	SOX2
CLUAP1	Enrichr CHEA_2022: SOX2 20726797 ChIP-Seq SW620 Human	SOX2
SCARB2	Enrichr CHEA_2022: SOX2 20726797 ChIP-Seq SW620 Human	SOX2
DCTN6	Enrichr CHEA_2022: SOX2 20726797 ChIP-Seq SW620 Human	SOX2
DDX28	Enrichr CHEA_2022: SOX2 20726797 ChIP-Seq SW620 Human	SOX2
LAMA3	Enrichr CHEA_2022: SOX2 20726797 ChIP-Seq SW620 Human	SOX2
MVP	Enrichr CHEA_2022: SOX2 20726797 ChIP-Seq SW620 Human	SOX2
ZNF292	Enrichr CHEA_2022: SOX2 20726797 ChIP-Seq SW620 Human	SOX2
CRMP1	Enrichr CHEA_2022: SOX2 20726797 ChIP-Seq SW620 Human	SOX2
ATP2A3	Enrichr CHEA_2022: SOX2 20726797 ChIP-Seq SW620 Human	SOX2
ATP2A2	Enrichr CHEA_2022: SOX2 20726797 ChIP-Seq SW620 Human	SOX2
RPGR	Enrichr CHEA_2022: SOX2 20726797 ChIP-Seq SW620 Human	SOX2
CLN6	Enrichr CHEA_2022: SOX2 20726797 ChIP-Seq SW620 Human	SOX2
CLMN	Enrichr CHEA_2022: SOX2 20726797 ChIP-Seq SW620 Human	SOX2
EPS8L1	Enrichr CHEA_2022: SOX2 20726797 ChIP-Seq SW620 Human	SOX2
SCN5A	Enrichr CHEA_2022: SOX2 20726797 ChIP-Seq SW620 Human	SOX2
EPS8L2	Enrichr CHEA_2022: SOX2 20726797 ChIP-Seq SW620 Human	SOX2
NPTN	Enrichr CHEA_2022: SOX2 20726797 ChIP-Seq SW620 Human	SOX2
ACOT9	Enrichr CHEA_2022: SOX2 20726797 ChIP-Seq SW620 Human	SOX2
MX1	Enrichr CHEA_2022: SOX2 20726797 ChIP-Seq SW620 Human	SOX2
GLCE	Enrichr CHEA_2022: SOX2 20726797 ChIP-Seq SW620 Human	SOX2
RPL23	Enrichr CHEA_2022: SOX2 20726797 ChIP-Seq SW620 Human	SOX2
SYTL2	Enrichr CHEA_2022: SOX2 20726797 ChIP-Seq SW620 Human	SOX2
UPF3B	Enrichr CHEA_2022: SOX2 20726797 ChIP-Seq SW620 Human	SOX2
CHFR	Enrichr CHEA_2022: SOX2 20726797 ChIP-Seq SW620 Human	SOX2
DYNC1LI1	Enrichr CHEA_2022: SOX2 20726797 ChIP-Seq SW620 Human	SOX2
GALNS	Enrichr CHEA_2022: SOX2 20726797 ChIP-Seq SW620 Human	SOX2
CPEB2	Enrichr CHEA_2022: SOX2 20726797 ChIP-Seq SW620 Human	SOX2
GPR160	Enrichr CHEA_2022: SOX2 20726797 ChIP-Seq SW620 Human	SOX2
ASB9	Enrichr CHEA_2022: SOX2 20726797 ChIP-Seq SW620 Human	SOX2
TRIP6	Enrichr CHEA_2022: SOX2 20726797 ChIP-Seq SW620 Human	SOX2
VIM	Enrichr CHEA_2022: SOX2 20726797 ChIP-Seq SW620 Human	SOX2
HSPA1A	Enrichr CHEA_2022: SOX2 20726797 ChIP-Seq SW620 Human	SOX2
ASF1A	Enrichr CHEA_2022: SOX2 20726797 ChIP-Seq SW620 Human	SOX2
ACOT2	Enrichr CHEA_2022: SOX2 20726797 ChIP-Seq SW620 Human	SOX2
ASB4	Enrichr CHEA_2022: SOX2 20726797 ChIP-Seq SW620 Human	SOX2
CPVL	Enrichr CHEA_2022: SOX2 20726797 ChIP-Seq SW620 Human	SOX2
HSPA1B	Enrichr CHEA_2022: SOX2 20726797 ChIP-Seq SW620 Human	SOX2
PARG	Enrichr CHEA_2022: SOX2 20726797 ChIP-Seq SW620 Human	SOX2
SF3B3	Enrichr CHEA_2022: SOX2 20726797 ChIP-Seq SW620 Human	SOX2
EHF	Enrichr CHEA_2022: SOX2 20726797 ChIP-Seq SW620 Human	SOX2
MAML3	Enrichr CHEA_2022: SOX2 20726797 ChIP-Seq SW620 Human	SOX2
AHNAK	Enrichr CHEA_2022: SOX2 20726797 ChIP-Seq SW620 Human	SOX2
SERPINE1	Enrichr CHEA_2022: SOX2 20726797 ChIP-Seq SW620 Human	SOX2

THYN1	Enrichr CHEA_2022: SOX2 20726797 ChIP-Seq SW620 Human	SOX2
ASAH1	Enrichr CHEA_2022: SOX2 20726797 ChIP-Seq SW620 Human	SOX2
PCMT1	Enrichr CHEA_2022: SOX2 20726797 ChIP-Seq SW620 Human	SOX2
CREB3L1	Enrichr CHEA_2022: SOX2 20726797 ChIP-Seq SW620 Human	SOX2
MYB	Enrichr CHEA_2022: SOX2 20726797 ChIP-Seq SW620 Human	SOX2
CHST13	Enrichr CHEA_2022: SOX2 20726797 ChIP-Seq SW620 Human	SOX2
CHST11	Enrichr CHEA_2022: SOX2 20726797 ChIP-Seq SW620 Human	SOX2
S100A14	Enrichr CHEA_2022: SOX2 20726797 ChIP-Seq SW620 Human	SOX2
SERPINF1	Enrichr CHEA_2022: SOX2 20726797 ChIP-Seq SW620 Human	SOX2
HOXA2	Enrichr CHEA_2022: SOX2 20726797 ChIP-Seq SW620 Human	SOX2
CYP1B1	Enrichr CHEA_2022: SOX2 20726797 ChIP-Seq SW620 Human	SOX2
HOXA7	Enrichr CHEA_2022: SOX2 20726797 ChIP-Seq SW620 Human	SOX2
HOXA5	Enrichr CHEA_2022: SOX2 20726797 ChIP-Seq SW620 Human	SOX2
F8A1	Enrichr CHEA_2022: SOX2 20726797 ChIP-Seq SW620 Human	SOX2
WNT10A	Enrichr CHEA_2022: SOX2 20726797 ChIP-Seq SW620 Human	SOX2
FZD3	Enrichr CHEA_2022: SOX2 20726797 ChIP-Seq SW620 Human	SOX2
FZD6	Enrichr CHEA_2022: SOX2 20726797 ChIP-Seq SW620 Human	SOX2
ST14	Enrichr CHEA_2022: SOX2 20726797 ChIP-Seq SW620 Human	SOX2
RAB3IP	Enrichr CHEA_2022: SOX2 20726797 ChIP-Seq SW620 Human	SOX2
FZD7	Enrichr CHEA_2022: SOX2 20726797 ChIP-Seq SW620 Human	SOX2
SLC16A10	Enrichr CHEA_2022: SOX2 20726797 ChIP-Seq SW620 Human	SOX2
GPR137B	Enrichr CHEA_2022: SOX2 20726797 ChIP-Seq SW620 Human	SOX2
SULT2B1	Enrichr CHEA_2022: SOX2 20726797 ChIP-Seq SW620 Human	SOX2
SLC6A8	Enrichr CHEA_2022: SOX2 20726797 ChIP-Seq SW620 Human	SOX2
BIN3	Enrichr CHEA_2022: SOX2 20726797 ChIP-Seq SW620 Human	SOX2
HOXB3	Enrichr CHEA_2022: SOX2 20726797 ChIP-Seq SW620 Human	SOX2
SSPN	Enrichr CHEA_2022: SOX2 20726797 ChIP-Seq SW620 Human	SOX2
HBP1	Enrichr CHEA_2022: SOX2 20726797 ChIP-Seq SW620 Human	SOX2
HOXB9	Enrichr CHEA_2022: SOX2 20726797 ChIP-Seq SW620 Human	SOX2
HSPA4L	Enrichr CHEA_2022: SOX2 20726797 ChIP-Seq SW620 Human	SOX2
RPL5	Enrichr CHEA_2022: SOX2 20726797 ChIP-Seq SW620 Human	SOX2
CDC42SE2	Enrichr CHEA_2022: SOX2 20726797 ChIP-Seq SW620 Human	SOX2
ZNF134	Enrichr CHEA_2022: SOX2 20726797 ChIP-Seq SW620 Human	SOX2
STMN3	Enrichr CHEA_2022: SOX2 20726797 ChIP-Seq SW620 Human	SOX2
ENO3	Enrichr CHEA_2022: SOX2 20726797 ChIP-Seq SW620 Human	SOX2
PRDX4	Enrichr CHEA_2022: SOX2 20726797 ChIP-Seq SW620 Human	SOX2
ZXDB	Enrichr CHEA_2022: SOX2 20726797 ChIP-Seq SW620 Human	SOX2
STMN1	Enrichr CHEA_2022: SOX2 20726797 ChIP-Seq SW620 Human	SOX2
RPL37	Enrichr CHEA_2022: SOX2 20726797 ChIP-Seq SW620 Human	SOX2
PLCG1	Enrichr CHEA_2022: SOX2 20726797 ChIP-Seq SW620 Human	SOX2
IER5	Enrichr CHEA_2022: SOX2 20726797 ChIP-Seq SW620 Human	SOX2
ZNF124	Enrichr CHEA_2022: SOX2 20726797 ChIP-Seq SW620 Human	SOX2
BLCAP	Enrichr CHEA_2022: SOX2 20726797 ChIP-Seq SW620 Human	SOX2
ANXA4	Enrichr CHEA_2022: SOX2 20726797 ChIP-Seq SW620 Human	SOX2
CCL20	Enrichr CHEA_2022: SOX2 20726797 ChIP-Seq SW620 Human	SOX2
ANXA5	Enrichr CHEA_2022: SOX2 20726797 ChIP-Seq SW620 Human	SOX2
TESC	Enrichr CHEA_2022: SOX2 20726797 ChIP-Seq SW620 Human	SOX2
PAX6	Enrichr CHEA_2022: SOX2 20726797 ChIP-Seq SW620 Human	SOX2
TIMM22	Enrichr CHEA_2022: SOX2 20726797 ChIP-Seq SW620 Human	SOX2
CARD6	Enrichr CHEA_2022: SOX2 20726797 ChIP-Seq SW620 Human	SOX2
ITPKA	Enrichr CHEA_2022: SOX2 20726797 ChIP-Seq SW620 Human	SOX2
GIPC2	Enrichr CHEA_2022: SOX2 20726797 ChIP-Seq SW620 Human	SOX2
JDP2	Enrichr CHEA_2022: SOX2 20726797 ChIP-Seq SW620 Human	SOX2
RNF141	Enrichr CHEA_2022: SOX2 20726797 ChIP-Seq SW620 Human	SOX2
REN	Enrichr CHEA_2022: SOX2 20726797 ChIP-Seq SW620 Human	SOX2
ADA	Enrichr CHEA_2022: SOX2 20726797 ChIP-Seq SW620 Human	SOX2
SERPINA1	Enrichr CHEA_2022: SOX2 20726797 ChIP-Seq SW620 Human	SOX2
CLTA	Enrichr CHEA_2022: SOX2 20726797 ChIP-Seq SW620 Human	SOX2
GLG1	Enrichr CHEA_2022: SOX2 20726797 ChIP-Seq SW620 Human	SOX2

RNF157	Enrichr CHEA_2022: SOX2 20726797 ChIP-Seq SW620 Human	SOX2
CSRP2	Enrichr CHEA_2022: SOX2 20726797 ChIP-Seq SW620 Human	SOX2
TRIM4	Enrichr CHEA_2022: SOX2 20726797 ChIP-Seq SW620 Human	SOX2
GPRASP2	Enrichr CHEA_2022: SOX2 20726797 ChIP-Seq SW620 Human	SOX2
ZNF589	Enrichr CHEA_2022: SOX2 20726797 ChIP-Seq SW620 Human	SOX2
TIGD2	Enrichr CHEA_2022: SOX2 20726797 ChIP-Seq SW620 Human	SOX2
ZNF587	Enrichr CHEA_2022: SOX2 20726797 ChIP-Seq SW620 Human	SOX2
ZNF586	Enrichr CHEA_2022: SOX2 20726797 ChIP-Seq SW620 Human	SOX2
WWOX	Enrichr CHEA_2022: SOX2 20726797 ChIP-Seq SW620 Human	SOX2
SERPINB5	Enrichr CHEA_2022: SOX2 20726797 ChIP-Seq SW620 Human	SOX2
SF3A3	Enrichr CHEA_2022: SOX2 20726797 ChIP-Seq SW620 Human	SOX2
SERPINB3	Enrichr CHEA_2022: SOX2 20726797 ChIP-Seq SW620 Human	SOX2
IFNGR1	Enrichr CHEA_2022: SOX2 20726797 ChIP-Seq SW620 Human	SOX2
TMOD3	Enrichr CHEA_2022: SOX2 20726797 ChIP-Seq SW620 Human	SOX2
WWP1	Enrichr CHEA_2022: SOX2 20726797 ChIP-Seq SW620 Human	SOX2
LAPTM5	Enrichr CHEA_2022: SOX2 20726797 ChIP-Seq SW620 Human	SOX2
EN2	Enrichr CHEA_2022: SOX2 20726797 ChIP-Seq SW620 Human	SOX2
ADAT1	Enrichr CHEA_2022: SOX2 20726797 ChIP-Seq SW620 Human	SOX2
GTF2H2	Enrichr CHEA_2022: SOX2 20726797 ChIP-Seq SW620 Human	SOX2
NMNAT3	Enrichr CHEA_2022: SOX2 20726797 ChIP-Seq SW620 Human	SOX2
TTC7B	Enrichr CHEA_2022: SOX2 20726797 ChIP-Seq SW620 Human	SOX2
SERPINB8	Enrichr CHEA_2022: SOX2 20726797 ChIP-Seq SW620 Human	SOX2
SULF2	Enrichr CHEA_2022: SOX2 20726797 ChIP-Seq SW620 Human	SOX2
TMEM154	Enrichr CHEA_2022: SOX2 20726797 ChIP-Seq SW620 Human	SOX2
COL4A2	Enrichr CHEA_2022: SOX2 20726797 ChIP-Seq SW620 Human	SOX2
RFWD3	Enrichr CHEA_2022: SOX2 20726797 ChIP-Seq SW620 Human	SOX2
MSRB3	Enrichr CHEA_2022: SOX2 20726797 ChIP-Seq SW620 Human	SOX2
NOTUM	Enrichr CHEA_2022: SOX2 20726797 ChIP-Seq SW620 Human	SOX2
HBEGF	Enrichr CHEA_2022: SOX2 20726797 ChIP-Seq SW620 Human	SOX2
BSCL2	Enrichr CHEA_2022: SOX2 20726797 ChIP-Seq SW620 Human	SOX2
ZNF573	Enrichr CHEA_2022: SOX2 20726797 ChIP-Seq SW620 Human	SOX2
CCL14	Enrichr CHEA_2022: SOX2 20726797 ChIP-Seq SW620 Human	SOX2
PFAS	Enrichr CHEA_2022: SOX2 20726797 ChIP-Seq SW620 Human	SOX2
ZNF572	Enrichr CHEA_2022: SOX2 20726797 ChIP-Seq SW620 Human	SOX2
OGG1	Enrichr CHEA_2022: SOX2 20726797 ChIP-Seq SW620 Human	SOX2
TGFB1I1	Enrichr CHEA_2022: SOX2 20726797 ChIP-Seq SW620 Human	SOX2
TMEM141	Enrichr CHEA_2022: SOX2 20726797 ChIP-Seq SW620 Human	SOX2
PRSS23	Enrichr CHEA_2022: SOX2 20726797 ChIP-Seq SW620 Human	SOX2
RNF135	Enrichr CHEA_2022: SOX2 20726797 ChIP-Seq SW620 Human	SOX2
FAM102B	Enrichr CHEA_2022: SOX2 20726797 ChIP-Seq SW620 Human	SOX2
RNF139	Enrichr CHEA_2022: SOX2 20726797 ChIP-Seq SW620 Human	SOX2
CHN2	Enrichr CHEA_2022: SOX2 20726797 ChIP-Seq SW620 Human	SOX2
METTL7A	Enrichr CHEA_2022: SOX2 20726797 ChIP-Seq SW620 Human	SOX2
CHN1	Enrichr CHEA_2022: SOX2 20726797 ChIP-Seq SW620 Human	SOX2
GCNT3	Enrichr CHEA_2022: SOX2 20726797 ChIP-Seq SW620 Human	SOX2
ANXA9	Enrichr CHEA_2022: SOX2 20726797 ChIP-Seq SW620 Human	SOX2
RIOK2	Enrichr CHEA_2022: SOX2 20726797 ChIP-Seq SW620 Human	SOX2
ANKRD50	Enrichr CHEA_2022: SOX2 20726797 ChIP-Seq SW620 Human	SOX2
ZNF567	Enrichr CHEA_2022: SOX2 20726797 ChIP-Seq SW620 Human	SOX2
BRD7	Enrichr CHEA_2022: SOX2 20726797 ChIP-Seq SW620 Human	SOX2
TNKS1BP1	Enrichr CHEA_2022: SOX2 20726797 ChIP-Seq SW620 Human	SOX2
ZNF565	Enrichr CHEA_2022: SOX2 20726797 ChIP-Seq SW620 Human	SOX2
RNF43	Enrichr CHEA_2022: SOX2 20726797 ChIP-Seq SW620 Human	SOX2
SEMA4B	Enrichr CHEA_2022: SOX2 20726797 ChIP-Seq SW620 Human	SOX2
PHLDA1	Enrichr CHEA_2022: SOX2 20726797 ChIP-Seq SW620 Human	SOX2
BRF2	Enrichr CHEA_2022: SOX2 20726797 ChIP-Seq SW620 Human	SOX2
SEMA4F	Enrichr CHEA_2022: SOX2 20726797 ChIP-Seq SW620 Human	SOX2
SEMA4C	Enrichr CHEA_2022: SOX2 20726797 ChIP-Seq SW620 Human	SOX2
PLCL2	Enrichr CHEA_2022: SOX2 20726797 ChIP-Seq SW620 Human	SOX2

SEMA4D	Enrichr CHEA_2022: SOX2 20726797 ChIP-Seq SW620 Human	SOX2
GPT2	Enrichr CHEA_2022: SOX2 20726797 ChIP-Seq SW620 Human	SOX2
FAM24B	Enrichr CHEA_2022: SOX2 20726797 ChIP-Seq SW620 Human	SOX2
CYBRD1	Enrichr CHEA_2022: SOX2 20726797 ChIP-Seq SW620 Human	SOX2
SEMA4G	Enrichr CHEA_2022: SOX2 20726797 ChIP-Seq SW620 Human	SOX2
CNOT7	Enrichr CHEA_2022: SOX2 20726797 ChIP-Seq SW620 Human	SOX2
SYPL1	Enrichr CHEA_2022: SOX2 20726797 ChIP-Seq SW620 Human	SOX2
RNF183	Enrichr CHEA_2022: SOX2 20726797 ChIP-Seq SW620 Human	SOX2
TUSC1	Enrichr CHEA_2022: SOX2 20726797 ChIP-Seq SW620 Human	SOX2
ANKRD22	Enrichr CHEA_2022: SOX2 20726797 ChIP-Seq SW620 Human	SOX2
ZNF559	Enrichr CHEA_2022: SOX2 20726797 ChIP-Seq SW620 Human	SOX2
TNIK	Enrichr CHEA_2022: SOX2 20726797 ChIP-Seq SW620 Human	SOX2
SLC28A3	Enrichr CHEA_2022: SOX2 20726797 ChIP-Seq SW620 Human	SOX2
RNF32	Enrichr CHEA_2022: SOX2 20726797 ChIP-Seq SW620 Human	SOX2
DGKH	Enrichr CHEA_2022: SOX2 20726797 ChIP-Seq SW620 Human	SOX2
ZNF552	Enrichr CHEA_2022: SOX2 20726797 ChIP-Seq SW620 Human	SOX2
ZNF551	Enrichr CHEA_2022: SOX2 20726797 ChIP-Seq SW620 Human	SOX2
COL16A1	Enrichr CHEA_2022: SOX2 20726797 ChIP-Seq SW620 Human	SOX2
PHLDB2	Enrichr CHEA_2022: SOX2 20726797 ChIP-Seq SW620 Human	SOX2
ZNF550	Enrichr CHEA_2022: SOX2 20726797 ChIP-Seq SW620 Human	SOX2
ANKRD37	Enrichr CHEA_2022: SOX2 20726797 ChIP-Seq SW620 Human	SOX2
AHR	Enrichr CHEA_2022: SOX2 20726797 ChIP-Seq SW620 Human	SOX2
METRNL	Enrichr CHEA_2022: SOX2 20726797 ChIP-Seq SW620 Human	SOX2
TANK	Enrichr CHEA_2022: SOX2 20726797 ChIP-Seq SW620 Human	SOX2
PYCARD	Enrichr CHEA_2022: SOX2 20726797 ChIP-Seq SW620 Human	SOX2
AP3M2	Enrichr CHEA_2022: SOX2 20726797 ChIP-Seq SW620 Human	SOX2
ZFP36	Enrichr CHEA_2022: SOX2 20726797 ChIP-Seq SW620 Human	SOX2
TUBB6	Enrichr CHEA_2022: SOX2 20726797 ChIP-Seq SW620 Human	SOX2
ZFP30	Enrichr CHEA_2022: SOX2 20726797 ChIP-Seq SW620 Human	SOX2
GRB14	Enrichr CHEA_2022: SOX2 20726797 ChIP-Seq SW620 Human	SOX2
ZNF548	Enrichr CHEA_2022: SOX2 20726797 ChIP-Seq SW620 Human	SOX2
ACSS1	Enrichr CHEA_2022: SOX2 20726797 ChIP-Seq SW620 Human	SOX2
IGFBP2	Enrichr CHEA_2022: SOX2 20726797 ChIP-Seq SW620 Human	SOX2
BIVM	Enrichr CHEA_2022: SOX2 20726797 ChIP-Seq SW620 Human	SOX2
F2R	Enrichr CHEA_2022: SOX2 20726797 ChIP-Seq SW620 Human	SOX2
RNF170	Enrichr CHEA_2022: SOX2 20726797 ChIP-Seq SW620 Human	SOX2
ADRA2A	Enrichr CHEA_2022: SOX2 20726797 ChIP-Seq SW620 Human	SOX2
DUSP23	Enrichr CHEA_2022: SOX2 20726797 ChIP-Seq SW620 Human	SOX2
ARMCX3	Enrichr CHEA_2022: SOX2 20726797 ChIP-Seq SW620 Human	SOX2
TRAF5	Enrichr CHEA_2022: SOX2 20726797 ChIP-Seq SW620 Human	SOX2
RHEB	Enrichr CHEA_2022: SOX2 20726797 ChIP-Seq SW620 Human	SOX2
TRAF4	Enrichr CHEA_2022: SOX2 20726797 ChIP-Seq SW620 Human	SOX2
ARMCX6	Enrichr CHEA_2022: SOX2 20726797 ChIP-Seq SW620 Human	SOX2
DGKQ	Enrichr CHEA_2022: SOX2 20726797 ChIP-Seq SW620 Human	SOX2
ILDR1	Enrichr CHEA_2022: SOX2 20726797 ChIP-Seq SW620 Human	SOX2
ERF	Enrichr CHEA_2022: SOX2 20726797 ChIP-Seq SW620 Human	SOX2
ESAM	Enrichr CHEA_2022: SOX2 20726797 ChIP-Seq SW620 Human	SOX2
RLN2	Enrichr CHEA_2022: SOX2 20726797 ChIP-Seq SW620 Human	SOX2
ZNF530	Enrichr CHEA_2022: SOX2 20726797 ChIP-Seq SW620 Human	SOX2
IRS1	Enrichr CHEA_2022: SOX2 20726797 ChIP-Seq SW620 Human	SOX2
FMR1	Enrichr CHEA_2022: SOX2 20726797 ChIP-Seq SW620 Human	SOX2
BRI3	Enrichr CHEA_2022: SOX2 20726797 ChIP-Seq SW620 Human	SOX2
AK5	Enrichr CHEA_2022: SOX2 20726797 ChIP-Seq SW620 Human	SOX2
MYOM3	Enrichr CHEA_2022: SOX2 20726797 ChIP-Seq SW620 Human	SOX2
ABHD12	Enrichr CHEA_2022: SOX2 20726797 ChIP-Seq SW620 Human	SOX2
BAG4	Enrichr CHEA_2022: SOX2 20726797 ChIP-Seq SW620 Human	SOX2
ABHD10	Enrichr CHEA_2022: SOX2 20726797 ChIP-Seq SW620 Human	SOX2
ZNF529	Enrichr CHEA_2022: SOX2 20726797 ChIP-Seq SW620 Human	SOX2
ANKRD10	Enrichr CHEA_2022: SOX2 20726797 ChIP-Seq SW620 Human	SOX2

SLC37A1	Enrichr CHEA_2022: SOX2 20726797 ChIP-Seq SW620 Human	SOX2
GPX3	Enrichr CHEA_2022: SOX2 20726797 ChIP-Seq SW620 Human	SOX2
GPX2	Enrichr CHEA_2022: SOX2 20726797 ChIP-Seq SW620 Human	SOX2
DTX3L	Enrichr CHEA_2022: SOX2 20726797 ChIP-Seq SW620 Human	SOX2
GPX7	Enrichr CHEA_2022: SOX2 20726797 ChIP-Seq SW620 Human	SOX2
SMARCE1	Enrichr CHEA_2022: SOX2 20726797 ChIP-Seq SW620 Human	SOX2
CFLAR	Enrichr CHEA_2022: SOX2 20726797 ChIP-Seq SW620 Human	SOX2
ASNS	Enrichr CHEA_2022: SOX2 20726797 ChIP-Seq SW620 Human	SOX2
PTPN13	Enrichr CHEA_2022: SOX2 20726797 ChIP-Seq SW620 Human	SOX2
CDC42BPA	Enrichr CHEA_2022: SOX2 20726797 ChIP-Seq SW620 Human	SOX2
TPCN2	Enrichr CHEA_2022: SOX2 20726797 ChIP-Seq SW620 Human	SOX2
CUL4A	Enrichr CHEA_2022: SOX2 20726797 ChIP-Seq SW620 Human	SOX2
SERPINI1	Enrichr CHEA_2022: SOX2 20726797 ChIP-Seq SW620 Human	SOX2
NES	Enrichr CHEA_2022: SOX2 20726797 ChIP-Seq SW620 Human	SOX2
SERINC2	Enrichr CHEA_2022: SOX2 20726797 ChIP-Seq SW620 Human	SOX2
ZNF512	Enrichr CHEA_2022: SOX2 20726797 ChIP-Seq SW620 Human	SOX2
EIF4E2	Enrichr CHEA_2022: SOX2 20726797 ChIP-Seq SW620 Human	SOX2
METAP1	Enrichr CHEA_2022: SOX2 20726797 ChIP-Seq SW620 Human	SOX2
METAP2	Enrichr CHEA_2022: SOX2 20726797 ChIP-Seq SW620 Human	SOX2
SERINC5	Enrichr CHEA_2022: SOX2 20726797 ChIP-Seq SW620 Human	SOX2
TNKS	Enrichr CHEA_2022: SOX2 20726797 ChIP-Seq SW620 Human	SOX2
ITPR2	Enrichr CHEA_2022: SOX2 20726797 ChIP-Seq SW620 Human	SOX2
ITPR3	Enrichr CHEA_2022: SOX2 20726797 ChIP-Seq SW620 Human	SOX2
DUSP14	Enrichr CHEA_2022: SOX2 20726797 ChIP-Seq SW620 Human	SOX2
RAD21	Enrichr CHEA_2022: SOX2 20726797 ChIP-Seq SW620 Human	SOX2
PSAP	Enrichr CHEA_2022: SOX2 20726797 ChIP-Seq SW620 Human	SOX2
WASF1	Enrichr CHEA_2022: SOX2 20726797 ChIP-Seq SW620 Human	SOX2
SKIL	Enrichr CHEA_2022: SOX2 20726797 ChIP-Seq SW620 Human	SOX2
WASF3	Enrichr CHEA_2022: SOX2 20726797 ChIP-Seq SW620 Human	SOX2
IRX2	Enrichr CHEA_2022: SOX2 20726797 ChIP-Seq SW620 Human	SOX2
SPAG7	Enrichr CHEA_2022: SOX2 20726797 ChIP-Seq SW620 Human	SOX2
SPAG4	Enrichr CHEA_2022: SOX2 20726797 ChIP-Seq SW620 Human	SOX2
AMT	Enrichr CHEA_2022: SOX2 20726797 ChIP-Seq SW620 Human	SOX2
MND1	Enrichr CHEA_2022: SOX2 20726797 ChIP-Seq SW620 Human	SOX2
RAD50	Enrichr CHEA_2022: SOX2 20726797 ChIP-Seq SW620 Human	SOX2
RNF20	Enrichr CHEA_2022: SOX2 20726797 ChIP-Seq SW620 Human	SOX2
KLF2	Enrichr CHEA_2022: SOX2 20726797 ChIP-Seq SW620 Human	SOX2
ZFP90	Enrichr CHEA_2022: SOX2 20726797 ChIP-Seq SW620 Human	SOX2
RAD52	Enrichr CHEA_2022: SOX2 20726797 ChIP-Seq SW620 Human	SOX2
MGAT4B	Enrichr CHEA_2022: SOX2 20726797 ChIP-Seq SW620 Human	SOX2
ASPH	Enrichr CHEA_2022: SOX2 20726797 ChIP-Seq SW620 Human	SOX2
CD109	Enrichr CHEA_2022: SOX2 20726797 ChIP-Seq SW620 Human	SOX2
EVL	Enrichr CHEA_2022: SOX2 20726797 ChIP-Seq SW620 Human	SOX2
CLEC2D	Enrichr CHEA_2022: SOX2 20726797 ChIP-Seq SW620 Human	SOX2
SMPDL3B	Enrichr CHEA_2022: SOX2 20726797 ChIP-Seq SW620 Human	SOX2
FOLR1	Enrichr CHEA_2022: SOX2 20726797 ChIP-Seq SW620 Human	SOX2
SEMA3A	Enrichr CHEA_2022: SOX2 20726797 ChIP-Seq SW620 Human	SOX2
LSP1	Enrichr CHEA_2022: SOX2 20726797 ChIP-Seq SW620 Human	SOX2
SEMA3C	Enrichr CHEA_2022: SOX2 20726797 ChIP-Seq SW620 Human	SOX2
SEMA3F	Enrichr CHEA_2022: SOX2 20726797 ChIP-Seq SW620 Human	SOX2
SGCE	Enrichr CHEA_2022: SOX2 20726797 ChIP-Seq SW620 Human	SOX2
OPA1	Enrichr CHEA_2022: SOX2 20726797 ChIP-Seq SW620 Human	SOX2
OPA3	Enrichr CHEA_2022: SOX2 20726797 ChIP-Seq SW620 Human	SOX2
NHS	Enrichr CHEA_2022: SOX2 20726797 ChIP-Seq SW620 Human	SOX2
PTPRN2	Enrichr CHEA_2022: SOX2 20726797 ChIP-Seq SW620 Human	SOX2
CDON	Enrichr CHEA_2022: SOX2 20726797 ChIP-Seq SW620 Human	SOX2
SS18L1	Enrichr CHEA_2022: SOX2 20726797 ChIP-Seq SW620 Human	SOX2
SP110	Enrichr CHEA_2022: SOX2 20726797 ChIP-Seq SW620 Human	SOX2
ACSL1	Enrichr CHEA_2022: SOX2 20726797 ChIP-Seq SW620 Human	SOX2

RUNX1	Enrichr CHEA_2022: SOX2 20726797 ChIP-Seq SW620 Human	SOX2
RECQL	Enrichr CHEA_2022: SOX2 20726797 ChIP-Seq SW620 Human	SOX2
ACSL5	Enrichr CHEA_2022: SOX2 20726797 ChIP-Seq SW620 Human	SOX2
RUNX3	Enrichr CHEA_2022: SOX2 20726797 ChIP-Seq SW620 Human	SOX2
MTUS1	Enrichr CHEA_2022: SOX2 20726797 ChIP-Seq SW620 Human	SOX2
RUNX2	Enrichr CHEA_2022: SOX2 20726797 ChIP-Seq SW620 Human	SOX2
MALL	Enrichr CHEA_2022: SOX2 20726797 ChIP-Seq SW620 Human	SOX2
RHEBL1	Enrichr CHEA_2022: SOX2 20726797 ChIP-Seq SW620 Human	SOX2
XRN2	Enrichr CHEA_2022: SOX2 20726797 ChIP-Seq SW620 Human	SOX2
RRAGD	Enrichr CHEA_2022: SOX2 20726797 ChIP-Seq SW620 Human	SOX2
TMCC1	Enrichr CHEA_2022: SOX2 20726797 ChIP-Seq SW620 Human	SOX2
GUF1	Enrichr CHEA_2022: SOX2 20726797 ChIP-Seq SW620 Human	SOX2
TFAM	Enrichr CHEA_2022: SOX2 20726797 ChIP-Seq SW620 Human	SOX2
PFN2	Enrichr CHEA_2022: SOX2 20726797 ChIP-Seq SW620 Human	SOX2
SPRR3	Enrichr CHEA_2022: SOX2 20726797 ChIP-Seq SW620 Human	SOX2
GLS2	Enrichr CHEA_2022: SOX2 20726797 ChIP-Seq SW620 Human	SOX2
CMAS	Enrichr CHEA_2022: SOX2 20726797 ChIP-Seq SW620 Human	SOX2
VIPR1	Enrichr CHEA_2022: SOX2 20726797 ChIP-Seq SW620 Human	SOX2
GLRX	Enrichr CHEA_2022: SOX2 20726797 ChIP-Seq SW620 Human	SOX2
RAD54B	Enrichr CHEA_2022: SOX2 20726797 ChIP-Seq SW620 Human	SOX2
ANTXR2	Enrichr CHEA_2022: SOX2 20726797 ChIP-Seq SW620 Human	SOX2
CARHSP1	Enrichr CHEA_2022: SOX2 20726797 ChIP-Seq SW620 Human	SOX2
ANTXR1	Enrichr CHEA_2022: SOX2 20726797 ChIP-Seq SW620 Human	SOX2
CHAC1	Enrichr CHEA_2022: SOX2 20726797 ChIP-Seq SW620 Human	SOX2
MAL2	Enrichr CHEA_2022: SOX2 20726797 ChIP-Seq SW620 Human	SOX2
RABGAP1L	Enrichr CHEA_2022: SOX2 20726797 ChIP-Seq SW620 Human	SOX2
AQR	Enrichr CHEA_2022: SOX2 20726797 ChIP-Seq SW620 Human	SOX2
LIX1L	Enrichr CHEA_2022: SOX2 20726797 ChIP-Seq SW620 Human	SOX2
GADD45B	Enrichr CHEA_2022: SOX2 20726797 ChIP-Seq SW620 Human	SOX2
GADD45A	Enrichr CHEA_2022: SOX2 20726797 ChIP-Seq SW620 Human	SOX2
SLC11A2	Enrichr CHEA_2022: SOX2 20726797 ChIP-Seq SW620 Human	SOX2
ACADSB	Enrichr CHEA_2022: SOX2 20726797 ChIP-Seq SW620 Human	SOX2
OVOL1	Enrichr CHEA_2022: SOX2 20726797 ChIP-Seq SW620 Human	SOX2
IL17RD	Enrichr CHEA_2022: SOX2 20726797 ChIP-Seq SW620 Human	SOX2
PARP14	Enrichr CHEA_2022: SOX2 20726797 ChIP-Seq SW620 Human	SOX2
INPP4B	Enrichr CHEA_2022: SOX2 20726797 ChIP-Seq SW620 Human	SOX2
PARP12	Enrichr CHEA_2022: SOX2 20726797 ChIP-Seq SW620 Human	SOX2
TSHZ1	Enrichr CHEA_2022: SOX2 20726797 ChIP-Seq SW620 Human	SOX2
TRMT12	Enrichr CHEA_2022: SOX2 20726797 ChIP-Seq SW620 Human	SOX2
ITM2C	Enrichr CHEA_2022: SOX2 20726797 ChIP-Seq SW620 Human	SOX2
CDS1	Enrichr CHEA_2022: SOX2 20726797 ChIP-Seq SW620 Human	SOX2
BEX2	Enrichr CHEA_2022: SOX2 20726797 ChIP-Seq SW620 Human	SOX2
ACSM3	Enrichr CHEA_2022: SOX2 20726797 ChIP-Seq SW620 Human	SOX2
MGST2	Enrichr CHEA_2022: SOX2 20726797 ChIP-Seq SW620 Human	SOX2
DIXDC1	Enrichr CHEA_2022: SOX2 20726797 ChIP-Seq SW620 Human	SOX2
EPM2AIP1	Enrichr CHEA_2022: SOX2 20726797 ChIP-Seq SW620 Human	SOX2
SOX2	Enrichr CHEA_2022: SOX2 20726797 ChIP-Seq SW620 Human	SOX2
INPP5F	Enrichr CHEA_2022: SOX2 20726797 ChIP-Seq SW620 Human	SOX2
ASL	Enrichr CHEA_2022: SOX2 20726797 ChIP-Seq SW620 Human	SOX2
ARL6IP5	Enrichr CHEA_2022: SOX2 20726797 ChIP-Seq SW620 Human	SOX2
TREX1	Enrichr CHEA_2022: SOX2 20726797 ChIP-Seq SW620 Human	SOX2
GLUL	Enrichr CHEA_2022: SOX2 20726797 ChIP-Seq SW620 Human	SOX2
GOLGA7	Enrichr CHEA_2022: SOX2 20726797 ChIP-Seq SW620 Human	SOX2
TNS4	Enrichr CHEA_2022: SOX2 20726797 ChIP-Seq SW620 Human	SOX2
SOX8	Enrichr CHEA_2022: SOX2 20726797 ChIP-Seq SW620 Human	SOX2
TNS3	Enrichr CHEA_2022: SOX2 20726797 ChIP-Seq SW620 Human	SOX2
CDR2	Enrichr CHEA_2022: SOX2 20726797 ChIP-Seq SW620 Human	SOX2
NMB	Enrichr CHEA_2022: SOX2 20726797 ChIP-Seq SW620 Human	SOX2
TSC22D2	Enrichr CHEA_2022: SOX2 20726797 ChIP-Seq SW620 Human	SOX2

NMI	Enrichr CHEA_2022: SOX2 20726797 ChIP-Seq SW620 Human	SOX2
NME3	Enrichr CHEA_2022: SOX2 20726797 ChIP-Seq SW620 Human	SOX2
TFEB	Enrichr CHEA_2022: SOX2 20726797 ChIP-Seq SW620 Human	SOX2
KRT13	Enrichr CHEA_2022: SOX2 20726797 ChIP-Seq SW620 Human	SOX2
NME4	Enrichr CHEA_2022: SOX2 20726797 ChIP-Seq SW620 Human	SOX2
TUBE1	Enrichr CHEA_2022: SOX2 20726797 ChIP-Seq SW620 Human	SOX2
GNG12	Enrichr CHEA_2022: SOX2 20726797 ChIP-Seq SW620 Human	SOX2
PDZK1IP1	Enrichr CHEA_2022: SOX2 20726797 ChIP-Seq SW620 Human	SOX2
RAD51C	Enrichr CHEA_2022: SOX2 20726797 ChIP-Seq SW620 Human	SOX2
RHOQ	Enrichr CHEA_2022: SOX2 20726797 ChIP-Seq SW620 Human	SOX2
CEACAM1	Enrichr CHEA_2022: SOX2 20726797 ChIP-Seq SW620 Human	SOX2
NME7	Enrichr CHEA_2022: SOX2 20726797 ChIP-Seq SW620 Human	SOX2
R3HCC1	Enrichr CHEA_2022: SOX2 20726797 ChIP-Seq SW620 Human	SOX2
KRT17	Enrichr CHEA_2022: SOX2 20726797 ChIP-Seq SW620 Human	SOX2
RPUSD3	Enrichr CHEA_2022: SOX2 20726797 ChIP-Seq SW620 Human	SOX2
CEACAM6	Enrichr CHEA_2022: SOX2 20726797 ChIP-Seq SW620 Human	SOX2
RPUSD4	Enrichr CHEA_2022: SOX2 20726797 ChIP-Seq SW620 Human	SOX2
ATM	Enrichr CHEA_2022: SOX2 20726797 ChIP-Seq SW620 Human	SOX2
TFF3	Enrichr CHEA_2022: SOX2 20726797 ChIP-Seq SW620 Human	SOX2
CAMK1	Enrichr CHEA_2022: SOX2 20726797 ChIP-Seq SW620 Human	SOX2
TFF1	Enrichr CHEA_2022: SOX2 20726797 ChIP-Seq SW620 Human	SOX2
SGK2	Enrichr CHEA_2022: SOX2 20726797 ChIP-Seq SW620 Human	SOX2
AKR1B10	Enrichr CHEA_2022: SOX2 20726797 ChIP-Seq SW620 Human	SOX2
RHOU	Enrichr CHEA_2022: SOX2 20726797 ChIP-Seq SW620 Human	SOX2
TRAM2	Enrichr CHEA_2022: SOX2 20726797 ChIP-Seq SW620 Human	SOX2
CLIC5	Enrichr CHEA_2022: SOX2 20726797 ChIP-Seq SW620 Human	SOX2
CLIC4	Enrichr CHEA_2022: SOX2 20726797 ChIP-Seq SW620 Human	SOX2
MAOB	Enrichr CHEA_2022: SOX2 20726797 ChIP-Seq SW620 Human	SOX2
MAOA	Enrichr CHEA_2022: SOX2 20726797 ChIP-Seq SW620 Human	SOX2
IGSF3	Enrichr CHEA_2022: SOX2 20726797 ChIP-Seq SW620 Human	SOX2
KLK6	Enrichr CHEA_2022: SOX2 20726797 ChIP-Seq SW620 Human	SOX2
STC2	Enrichr CHEA_2022: SOX2 20726797 ChIP-Seq SW620 Human	SOX2
VLDLR	Enrichr CHEA_2022: SOX2 20726797 ChIP-Seq SW620 Human	SOX2
KRT20	Enrichr CHEA_2022: SOX2 20726797 ChIP-Seq SW620 Human	SOX2
NNT	Enrichr CHEA_2022: SOX2 20726797 ChIP-Seq SW620 Human	SOX2
HINT3	Enrichr CHEA_2022: SOX2 20726797 ChIP-Seq SW620 Human	SOX2
PITRM1	Enrichr CHEA_2022: SOX2 20726797 ChIP-Seq SW620 Human	SOX2
CLIC3	Enrichr CHEA_2022: SOX2 20726797 ChIP-Seq SW620 Human	SOX2
ARNT2	Enrichr CHEA_2022: SOX2 20726797 ChIP-Seq SW620 Human	SOX2
PRMT7	Enrichr CHEA_2022: SOX2 20726797 ChIP-Seq SW620 Human	SOX2
PRMT5	Enrichr CHEA_2022: SOX2 20726797 ChIP-Seq SW620 Human	SOX2
SCD5	Enrichr CHEA_2022: SOX2 20726797 ChIP-Seq SW620 Human	SOX2
RSL1D1	Enrichr CHEA_2022: SOX2 20726797 ChIP-Seq SW620 Human	SOX2
TJP2	Enrichr CHEA_2022: SOX2 20726797 ChIP-Seq SW620 Human	SOX2
TJP1	Enrichr CHEA_2022: SOX2 20726797 ChIP-Seq SW620 Human	SOX2
DNASE1L1	Enrichr CHEA_2022: SOX2 20726797 ChIP-Seq SW620 Human	SOX2
TJP3	Enrichr CHEA_2022: SOX2 20726797 ChIP-Seq SW620 Human	SOX2
DCP2	Enrichr CHEA_2022: SOX2 20726797 ChIP-Seq SW620 Human	SOX2
GDI1	Enrichr CHEA_2022: SOX2 20726797 ChIP-Seq SW620 Human	SOX2
PRIM1	Enrichr CHEA_2022: SOX2 20726797 ChIP-Seq SW620 Human	SOX2
HCCS	Enrichr CHEA_2022: SOX2 20726797 ChIP-Seq SW620 Human	SOX2
TRAK2	Enrichr CHEA_2022: SOX2 20726797 ChIP-Seq SW620 Human	SOX2
TRAK1	Enrichr CHEA_2022: SOX2 20726797 ChIP-Seq SW620 Human	SOX2
GYS1	Enrichr CHEA_2022: SOX2 20726797 ChIP-Seq SW620 Human	SOX2
NPW	Enrichr CHEA_2022: SOX2 20726797 ChIP-Seq SW620 Human	SOX2
MAP4K4	Enrichr CHEA_2022: SOX2 20726797 ChIP-Seq SW620 Human	SOX2
GDF11	Enrichr CHEA_2022: SOX2 20726797 ChIP-Seq SW620 Human	SOX2
CA13	Enrichr CHEA_2022: SOX2 20726797 ChIP-Seq SW620 Human	SOX2
BTNL9	Enrichr CHEA_2022: SOX2 20726797 ChIP-Seq SW620 Human	SOX2

CA12	Enrichr CHEA_2022: SOX2 20726797 ChIP-Seq SW620 Human	SOX2
TGFB2	Enrichr CHEA_2022: SOX2 20726797 ChIP-Seq SW620 Human	SOX2
KLRC4	Enrichr CHEA_2022: SOX2 20726797 ChIP-Seq SW620 Human	SOX2
EPDR1	Enrichr CHEA_2022: SOX2 20726797 ChIP-Seq SW620 Human	SOX2
ARID3A	Enrichr CHEA_2022: SOX2 20726797 ChIP-Seq SW620 Human	SOX2
ARHGAP27	Enrichr CHEA_2022: SOX2 20726797 ChIP-Seq SW620 Human	SOX2
BATF	Enrichr CHEA_2022: SOX2 20726797 ChIP-Seq SW620 Human	SOX2
ZNFX1	Enrichr CHEA_2022: SOX2 20726797 ChIP-Seq SW620 Human	SOX2
GNPDA2	Enrichr CHEA_2022: SOX2 20726797 ChIP-Seq SW620 Human	SOX2
AXL	Enrichr CHEA_2022: SOX2 20726797 ChIP-Seq SW620 Human	SOX2
TMEM139	Enrichr CHEA_2022: SOX2 20726797 ChIP-Seq SW620 Human	SOX2
FAS	Enrichr CHEA_2022: SOX2 20726797 ChIP-Seq SW620 Human	SOX2
ELMO3	Enrichr CHEA_2022: SOX2 20726797 ChIP-Seq SW620 Human	SOX2
ABCG1	Enrichr CHEA_2022: SOX2 20726797 ChIP-Seq SW620 Human	SOX2
GOLT1A	Enrichr CHEA_2022: SOX2 20726797 ChIP-Seq SW620 Human	SOX2
SCG2	Enrichr CHEA_2022: SOX2 20726797 ChIP-Seq SW620 Human	SOX2
STEAP2	Enrichr CHEA_2022: SOX2 20726797 ChIP-Seq SW620 Human	SOX2
ABCG2	Enrichr CHEA_2022: SOX2 20726797 ChIP-Seq SW620 Human	SOX2
FAIM	Enrichr CHEA_2022: SOX2 20726797 ChIP-Seq SW620 Human	SOX2
STEAP1	Enrichr CHEA_2022: SOX2 20726797 ChIP-Seq SW620 Human	SOX2
GALNT12	Enrichr CHEA_2022: SOX2 20726797 ChIP-Seq SW620 Human	SOX2
GALNT14	Enrichr CHEA_2022: SOX2 20726797 ChIP-Seq SW620 Human	SOX2
SLFN5	Enrichr CHEA_2022: SOX2 20726797 ChIP-Seq SW620 Human	SOX2
HDLBP	Enrichr CHEA_2022: SOX2 20726797 ChIP-Seq SW620 Human	SOX2
IFI6	Enrichr CHEA_2022: SOX2 20726797 ChIP-Seq SW620 Human	SOX2
MANEA	Enrichr CHEA_2022: SOX2 20726797 ChIP-Seq SW620 Human	SOX2
GALNT10	Enrichr CHEA_2022: SOX2 20726797 ChIP-Seq SW620 Human	SOX2
ZFP36L2	Enrichr CHEA_2022: SOX2 20726797 ChIP-Seq SW620 Human	SOX2
SCEL	Enrichr CHEA_2022: SOX2 20726797 ChIP-Seq SW620 Human	SOX2
KRT6A	Enrichr CHEA_2022: SOX2 20726797 ChIP-Seq SW620 Human	SOX2
TMEM125	Enrichr CHEA_2022: SOX2 20726797 ChIP-Seq SW620 Human	SOX2
MYH14	Enrichr CHEA_2022: SOX2 20726797 ChIP-Seq SW620 Human	SOX2
MYH10	Enrichr CHEA_2022: SOX2 20726797 ChIP-Seq SW620 Human	SOX2
CLASP2	Enrichr CHEA_2022: SOX2 20726797 ChIP-Seq SW620 Human	SOX2
OPN3	Enrichr CHEA_2022: SOX2 20726797 ChIP-Seq SW620 Human	SOX2
USP47	Enrichr CHEA_2022: SOX2 20726797 ChIP-Seq SW620 Human	SOX2
SMAD3	Enrichr CHEA_2022: SOX2 20726797 ChIP-Seq SW620 Human	SOX2
PDIA3	Enrichr CHEA_2022: SOX2 20726797 ChIP-Seq SW620 Human	SOX2
SMAD5	Enrichr CHEA_2022: SOX2 20726797 ChIP-Seq SW620 Human	SOX2
ARID5B	Enrichr CHEA_2022: SOX2 20726797 ChIP-Seq SW620 Human	SOX2
DNAJC12	Enrichr CHEA_2022: SOX2 20726797 ChIP-Seq SW620 Human	SOX2
ASPSCR1	Enrichr CHEA_2022: SOX2 20726797 ChIP-Seq SW620 Human	SOX2
CTPS2	Enrichr CHEA_2022: SOX2 20726797 ChIP-Seq SW620 Human	SOX2
CDYL	Enrichr CHEA_2022: SOX2 20726797 ChIP-Seq SW620 Human	SOX2
SMAD7	Enrichr CHEA_2022: SOX2 20726797 ChIP-Seq SW620 Human	SOX2
UPK3B	Enrichr CHEA_2022: SOX2 20726797 ChIP-Seq SW620 Human	SOX2
SMAD6	Enrichr CHEA_2022: SOX2 20726797 ChIP-Seq SW620 Human	SOX2
DNAJC18	Enrichr CHEA_2022: SOX2 20726797 ChIP-Seq SW620 Human	SOX2
SDCBP	Enrichr CHEA_2022: SOX2 20726797 ChIP-Seq SW620 Human	SOX2
PDIA6	Enrichr CHEA_2022: SOX2 20726797 ChIP-Seq SW620 Human	SOX2
ALDH3A2	Enrichr CHEA_2022: SOX2 20726797 ChIP-Seq SW620 Human	SOX2
PREPL	Enrichr CHEA_2022: SOX2 20726797 ChIP-Seq SW620 Human	SOX2
DLG4	Enrichr CHEA_2022: SOX2 20726797 ChIP-Seq SW620 Human	SOX2
CHMP7	Enrichr CHEA_2022: SOX2 20726797 ChIP-Seq SW620 Human	SOX2
ATP6V1A	Enrichr CHEA_2022: SOX2 20726797 ChIP-Seq SW620 Human	SOX2
DOCK4	Enrichr CHEA_2022: SOX2 20726797 ChIP-Seq SW620 Human	SOX2
USP54	Enrichr CHEA_2022: SOX2 20726797 ChIP-Seq SW620 Human	SOX2
SH3KBP1	Enrichr CHEA_2022: SOX2 20726797 ChIP-Seq SW620 Human	SOX2
SLC2A3	Enrichr CHEA_2022: SOX2 20726797 ChIP-Seq SW620 Human	SOX2

ARHGAP18	Enrichr CHEA_2022: SOX2 20726797 ChIP-Seq SW620 Human	SOX2
PLCXD2	Enrichr CHEA_2022: SOX2 20726797 ChIP-Seq SW620 Human	SOX2
MYL6B	Enrichr CHEA_2022: SOX2 20726797 ChIP-Seq SW620 Human	SOX2
ARHGAP23	Enrichr CHEA_2022: SOX2 20726797 ChIP-Seq SW620 Human	SOX2
PODXL	Enrichr CHEA_2022: SOX2 20726797 ChIP-Seq SW620 Human	SOX2
MAN2A2	Enrichr CHEA_2022: SOX2 20726797 ChIP-Seq SW620 Human	SOX2
TMEM107	Enrichr CHEA_2022: SOX2 20726797 ChIP-Seq SW620 Human	SOX2
ALDH3B1	Enrichr CHEA_2022: SOX2 20726797 ChIP-Seq SW620 Human	SOX2
COBLL1	Enrichr CHEA_2022: SOX2 20726797 ChIP-Seq SW620 Human	SOX2
PSPH	Enrichr CHEA_2022: SOX2 20726797 ChIP-Seq SW620 Human	SOX2
USP25	Enrichr CHEA_2022: SOX2 20726797 ChIP-Seq SW620 Human	SOX2
ABCC5	Enrichr CHEA_2022: SOX2 20726797 ChIP-Seq SW620 Human	SOX2
ABCC3	Enrichr CHEA_2022: SOX2 20726797 ChIP-Seq SW620 Human	SOX2
HSPA5	Enrichr CHEA_2022: SOX2 20726797 ChIP-Seq SW620 Human	SOX2
SMURF1	Enrichr CHEA_2022: SOX2 20726797 ChIP-Seq SW620 Human	SOX2
HSPA2	Enrichr CHEA_2022: SOX2 20726797 ChIP-Seq SW620 Human	SOX2
NFKBIZ	Enrichr CHEA_2022: SOX2 20726797 ChIP-Seq SW620 Human	SOX2
TGFBR3	Enrichr CHEA_2022: SOX2 20726797 ChIP-Seq SW620 Human	SOX2
NAP1L5	Enrichr CHEA_2022: SOX2 20726797 ChIP-Seq SW620 Human	SOX2
DDX19A	Enrichr CHEA_2022: SOX2 20726797 ChIP-Seq SW620 Human	SOX2
DDX19B	Enrichr CHEA_2022: SOX2 20726797 ChIP-Seq SW620 Human	SOX2
MEIS2	Enrichr CHEA_2022: SOX2 20726797 ChIP-Seq SW620 Human	SOX2
MNS1	Enrichr CHEA_2022: SOX2 20726797 ChIP-Seq SW620 Human	SOX2
PRTFDC1	Enrichr CHEA_2022: SOX2 20726797 ChIP-Seq SW620 Human	SOX2
B4GALT1	Enrichr CHEA_2022: SOX2 20726797 ChIP-Seq SW620 Human	SOX2
MCTP1	Enrichr CHEA_2022: SOX2 20726797 ChIP-Seq SW620 Human	SOX2
ABCB1	Enrichr CHEA_2022: SOX2 20726797 ChIP-Seq SW620 Human	SOX2
NID1	Enrichr CHEA_2022: SOX2 20726797 ChIP-Seq SW620 Human	SOX2
MTIF3	Enrichr CHEA_2022: SOX2 20726797 ChIP-Seq SW620 Human	SOX2
CXCL1	Enrichr CHEA_2022: SOX2 20726797 ChIP-Seq SW620 Human	SOX2
DTX4	Enrichr CHEA_2022: SOX2 20726797 ChIP-Seq SW620 Human	SOX2
ELAVL2	Enrichr CHEA_2022: SOX2 20726797 ChIP-Seq SW620 Human	SOX2
CXCL3	Enrichr CHEA_2022: SOX2 20726797 ChIP-Seq SW620 Human	SOX2
MOSPD1	Enrichr CHEA_2022: SOX2 20726797 ChIP-Seq SW620 Human	SOX2
CXCL6	Enrichr CHEA_2022: SOX2 20726797 ChIP-Seq SW620 Human	SOX2
DPP4	Enrichr CHEA_2022: SOX2 20726797 ChIP-Seq SW620 Human	SOX2
DPP7	Enrichr CHEA_2022: SOX2 20726797 ChIP-Seq SW620 Human	SOX2
NRIP1	Enrichr CHEA_2022: SOX2 20726797 ChIP-Seq SW620 Human	SOX2
NRIP3	Enrichr CHEA_2022: SOX2 20726797 ChIP-Seq SW620 Human	SOX2
ERBB2	Enrichr CHEA_2022: SOX2 20726797 ChIP-Seq SW620 Human	SOX2
PDE4A	Enrichr CHEA_2022: SOX2 20726797 ChIP-Seq SW620 Human	SOX2
USP31	Enrichr CHEA_2022: SOX2 20726797 ChIP-Seq SW620 Human	SOX2
TCFL5	Enrichr CHEA_2022: SOX2 20726797 ChIP-Seq SW620 Human	SOX2
ZNF185	Enrichr CHEA_2022: SOX2 20726797 ChIP-Seq SW620 Human	SOX2
PDE4DIP	Enrichr CHEA_2022: SOX2 20726797 ChIP-Seq SW620 Human	SOX2
RWDD1	Enrichr CHEA_2022: SOX2 20726797 ChIP-Seq SW620 Human	SOX2
PDE4D	Enrichr CHEA_2022: SOX2 20726797 ChIP-Seq SW620 Human	SOX2
ZNF182	Enrichr CHEA_2022: SOX2 20726797 ChIP-Seq SW620 Human	SOX2
KCTD1	Enrichr CHEA_2022: SOX2 20726797 ChIP-Seq SW620 Human	SOX2
KCTD6	Enrichr CHEA_2022: SOX2 20726797 ChIP-Seq SW620 Human	SOX2
SGPP2	Enrichr CHEA_2022: SOX2 20726797 ChIP-Seq SW620 Human	SOX2
CLDN7	Enrichr CHEA_2022: SOX2 20726797 ChIP-Seq SW620 Human	SOX2
FMNL2	Enrichr CHEA_2022: SOX2 20726797 ChIP-Seq SW620 Human	SOX2
CLDN4	Enrichr CHEA_2022: SOX2 20726797 ChIP-Seq SW620 Human	SOX2
PALLD	Enrichr CHEA_2022: SOX2 20726797 ChIP-Seq SW620 Human	SOX2
COL8A1	Enrichr CHEA_2022: SOX2 20726797 ChIP-Seq SW620 Human	SOX2
HKDC1	Enrichr CHEA_2022: SOX2 20726797 ChIP-Seq SW620 Human	SOX2
VDAC3	Enrichr CHEA_2022: SOX2 20726797 ChIP-Seq SW620 Human	SOX2
SPRY2	Enrichr CHEA_2022: SOX2 20726797 ChIP-Seq SW620 Human	SOX2

SSBP2	Enrichr CHEA_2022: SOX2 20726797 ChIP-Seq SW620 Human	SOX2
B4GALT6	Enrichr CHEA_2022: SOX2 20726797 ChIP-Seq SW620 Human	SOX2
CAMK2D	Enrichr CHEA_2022: SOX2 20726797 ChIP-Seq SW620 Human	SOX2
TSEN34	Enrichr CHEA_2022: SOX2 20726797 ChIP-Seq SW620 Human	SOX2
STEAP4	Enrichr CHEA_2022: SOX2 20726797 ChIP-Seq SW620 Human	SOX2
ZNF174	Enrichr CHEA_2022: SOX2 20726797 ChIP-Seq SW620 Human	SOX2
USP11	Enrichr CHEA_2022: SOX2 20726797 ChIP-Seq SW620 Human	SOX2
USP12	Enrichr CHEA_2022: SOX2 20726797 ChIP-Seq SW620 Human	SOX2
USP13	Enrichr CHEA_2022: SOX2 20726797 ChIP-Seq SW620 Human	SOX2
CD163L1	Enrichr CHEA_2022: SOX2 20726797 ChIP-Seq SW620 Human	SOX2
HSPD1	Enrichr CHEA_2022: SOX2 20726797 ChIP-Seq SW620 Human	SOX2
USP18	Enrichr CHEA_2022: SOX2 20726797 ChIP-Seq SW620 Human	SOX2
SH3YL1	Enrichr CHEA_2022: SOX2 20726797 ChIP-Seq SW620 Human	SOX2
NT5DC1	Enrichr CHEA_2022: SOX2 20726797 ChIP-Seq SW620 Human	SOX2
PRRG4	Enrichr CHEA_2022: SOX2 20726797 ChIP-Seq SW620 Human	SOX2
POLB	Enrichr CHEA_2022: SOX2 20726797 ChIP-Seq SW620 Human	SOX2
VSIG2	Enrichr CHEA_2022: SOX2 20726797 ChIP-Seq SW620 Human	SOX2
VSIG1	Enrichr CHEA_2022: SOX2 20726797 ChIP-Seq SW620 Human	SOX2
CCNJL	Enrichr TF Perturbations followed by expression: KLF5 20726797 ChIP-Seq SW620 Human	KLF5
RBFOX3	Enrichr TF Perturbations followed by expression: KLF5 20726797 ChIP-Seq SW620 Human	KLF5
SP110	Enrichr TF Perturbations followed by expression: KLF5 20726797 ChIP-Seq SW620 Human	KLF5
HOMER2	Enrichr TF Perturbations followed by expression: KLF5 20726797 ChIP-Seq SW620 Human	KLF5
KRT4	Enrichr TF Perturbations followed by expression: KLF5 20726797 ChIP-Seq SW620 Human	KLF5
ARL14	Enrichr TF Perturbations followed by expression: KLF5 20726797 ChIP-Seq SW620 Human	KLF5
CKMT1A	Enrichr TF Perturbations followed by expression: KLF5 20726797 ChIP-Seq SW620 Human	KLF5
KRT7	Enrichr TF Perturbations followed by expression: KLF5 20726797 ChIP-Seq SW620 Human	KLF5
L1CAM	Enrichr TF Perturbations followed by expression: KLF5 20726797 ChIP-Seq SW620 Human	KLF5
MALL	Enrichr TF Perturbations followed by expression: KLF5 20726797 ChIP-Seq SW620 Human	KLF5
CAPN13	Enrichr TF Perturbations followed by expression: KLF5 20726797 ChIP-Seq SW620 Human	KLF5
LGALS12	Enrichr TF Perturbations followed by expression: KLF5 20726797 ChIP-Seq SW620 Human	KLF5
MYL9	Enrichr TF Perturbations followed by expression: KLF5 20726797 ChIP-Seq SW620 Human	KLF5
VIP	Enrichr TF Perturbations followed by expression: KLF5 20726797 ChIP-Seq SW620 Human	KLF5
EPHA4	Enrichr TF Perturbations followed by expression: KLF5 20726797 ChIP-Seq SW620 Human	KLF5
CHIC1	Enrichr TF Perturbations followed by expression: KLF5 20726797 ChIP-Seq SW620 Human	KLF5
SYNPO2	Enrichr TF Perturbations followed by expression: KLF5 20726797 ChIP-Seq SW620 Human	KLF5
MST1R	Enrichr TF Perturbations followed by expression: KLF5 20726797 ChIP-Seq SW620 Human	KLF5
CRACR2A	Enrichr TF Perturbations followed by expression: KLF5 20726797 ChIP-Seq SW620 Human	KLF5
MUC1	Enrichr TF Perturbations followed by expression: KLF5 20726797 ChIP-Seq SW620 Human	KLF5
STRIP2	Enrichr TF Perturbations followed by expression: KLF5 20726797 ChIP-Seq SW620 Human	KLF5
GNG3	Enrichr TF Perturbations followed by expression: KLF5 20726797 ChIP-Seq SW620 Human	KLF5
CREB3L4	Enrichr TF Perturbations followed by expression: KLF5 20726797 ChIP-Seq SW620 Human	KLF5
SV2C	Enrichr TF Perturbations followed by expression: KLF5 20726797 ChIP-Seq SW620 Human	KLF5
GMDS	Enrichr TF Perturbations followed by expression: KLF5 20726797 ChIP-Seq SW620 Human	KLF5
SPOCK3	Enrichr TF Perturbations followed by expression: KLF5 20726797 ChIP-Seq SW620 Human	KLF5
S100A14	Enrichr TF Perturbations followed by expression: KLF5 20726797 ChIP-Seq SW620 Human	KLF5
SUSD4	Enrichr TF Perturbations followed by expression: KLF5 20726797 ChIP-Seq SW620 Human	KLF5
SPOCK2	Enrichr TF Perturbations followed by expression: KLF5 20726797 ChIP-Seq SW620 Human	KLF5
MUC4	Enrichr TF Perturbations followed by expression: KLF5 20726797 ChIP-Seq SW620 Human	KLF5
SLC12A8	Enrichr TF Perturbations followed by expression: KLF5 20726797 ChIP-Seq SW620 Human	KLF5
PPARGC1B	Enrichr TF Perturbations followed by expression: KLF5 20726797 ChIP-Seq SW620 Human	KLF5
FZD3	Enrichr TF Perturbations followed by expression: KLF5 20726797 ChIP-Seq SW620 Human	KLF5
PAQR6	Enrichr TF Perturbations followed by expression: KLF5 20726797 ChIP-Seq SW620 Human	KLF5
RAB27A	Enrichr TF Perturbations followed by expression: KLF5 20726797 ChIP-Seq SW620 Human	KLF5
CMBL	Enrichr TF Perturbations followed by expression: KLF5 20726797 ChIP-Seq SW620 Human	KLF5
ANK2	Enrichr TF Perturbations followed by expression: KLF5 20726797 ChIP-Seq SW620 Human	KLF5
FA2H	Enrichr TF Perturbations followed by expression: KLF5 20726797 ChIP-Seq SW620 Human	KLF5
CNKSR2	Enrichr TF Perturbations followed by expression: KLF5 20726797 ChIP-Seq SW620 Human	KLF5
POR	Enrichr TF Perturbations followed by expression: KLF5 20726797 ChIP-Seq SW620 Human	KLF5
RIPPLY3	Enrichr TF Perturbations followed by expression: KLF5 20726797 ChIP-Seq SW620 Human	KLF5

PPP1R1B	Enrichr TF Perturbations followed by expression: KLF5 20726797 ChIP-Seq SW620 Human	KLF5
SNHG11	Enrichr TF Perturbations followed by expression: KLF5 20726797 ChIP-Seq SW620 Human	KLF5
SPATS2L	Enrichr TF Perturbations followed by expression: KLF5 20726797 ChIP-Seq SW620 Human	KLF5
IGSF9B	Enrichr TF Perturbations followed by expression: KLF5 20726797 ChIP-Seq SW620 Human	KLF5
XKR4	Enrichr TF Perturbations followed by expression: KLF5 20726797 ChIP-Seq SW620 Human	KLF5
PTGES	Enrichr TF Perturbations followed by expression: KLF5 20726797 ChIP-Seq SW620 Human	KLF5
GLDC	Enrichr TF Perturbations followed by expression: KLF5 20726797 ChIP-Seq SW620 Human	KLF5
ACSM3	Enrichr TF Perturbations followed by expression: KLF5 20726797 ChIP-Seq SW620 Human	KLF5
NECAB1	Enrichr TF Perturbations followed by expression: KLF5 20726797 ChIP-Seq SW620 Human	KLF5
HHIP	Enrichr TF Perturbations followed by expression: KLF5 20726797 ChIP-Seq SW620 Human	KLF5
NAPEPLD	Enrichr TF Perturbations followed by expression: KLF5 20726797 ChIP-Seq SW620 Human	KLF5
WFDC2	Enrichr TF Perturbations followed by expression: KLF5 20726797 ChIP-Seq SW620 Human	KLF5
RAPGEFL1	Enrichr TF Perturbations followed by expression: KLF5 20726797 ChIP-Seq SW620 Human	KLF5
SESTD1	Enrichr TF Perturbations followed by expression: KLF5 20726797 ChIP-Seq SW620 Human	KLF5
GATA2	Enrichr TF Perturbations followed by expression: KLF5 20726797 ChIP-Seq SW620 Human	KLF5
SOX2	Enrichr TF Perturbations followed by expression: KLF5 20726797 ChIP-Seq SW620 Human	KLF5
TSPAN8	Enrichr TF Perturbations followed by expression: KLF5 20726797 ChIP-Seq SW620 Human	KLF5
REG4	Enrichr TF Perturbations followed by expression: KLF5 20726797 ChIP-Seq SW620 Human	KLF5
FFAR4	Enrichr TF Perturbations followed by expression: KLF5 20726797 ChIP-Seq SW620 Human	KLF5
TNFSF10	Enrichr TF Perturbations followed by expression: KLF5 20726797 ChIP-Seq SW620 Human	KLF5
CD14	Enrichr TF Perturbations followed by expression: KLF5 20726797 ChIP-Seq SW620 Human	KLF5
TSPAN2	Enrichr TF Perturbations followed by expression: KLF5 20726797 ChIP-Seq SW620 Human	KLF5
PROM1	Enrichr TF Perturbations followed by expression: KLF5 20726797 ChIP-Seq SW620 Human	KLF5
LRRC26	Enrichr TF Perturbations followed by expression: KLF5 20726797 ChIP-Seq SW620 Human	KLF5
PCP4L1	Enrichr TF Perturbations followed by expression: KLF5 20726797 ChIP-Seq SW620 Human	KLF5
GSDMC	Enrichr TF Perturbations followed by expression: KLF5 20726797 ChIP-Seq SW620 Human	KLF5
TESC	Enrichr TF Perturbations followed by expression: KLF5 20726797 ChIP-Seq SW620 Human	KLF5
GP2	Enrichr TF Perturbations followed by expression: KLF5 20726797 ChIP-Seq SW620 Human	KLF5
TMC5	Enrichr TF Perturbations followed by expression: KLF5 20726797 ChIP-Seq SW620 Human	KLF5
SYN2	Enrichr TF Perturbations followed by expression: KLF5 20726797 ChIP-Seq SW620 Human	KLF5
GPRC5A	Enrichr TF Perturbations followed by expression: KLF5 20726797 ChIP-Seq SW620 Human	KLF5
TTLL7	Enrichr TF Perturbations followed by expression: KLF5 20726797 ChIP-Seq SW620 Human	KLF5
TOX3	Enrichr TF Perturbations followed by expression: KLF5 20726797 ChIP-Seq SW620 Human	KLF5
TPPP3	Enrichr TF Perturbations followed by expression: KLF5 20726797 ChIP-Seq SW620 Human	KLF5
ARHGEF4	Enrichr TF Perturbations followed by expression: KLF5 20726797 ChIP-Seq SW620 Human	KLF5
TFF3	Enrichr TF Perturbations followed by expression: KLF5 20726797 ChIP-Seq SW620 Human	KLF5
RET	Enrichr TF Perturbations followed by expression: KLF5 20726797 ChIP-Seq SW620 Human	KLF5
KLK1	Enrichr TF Perturbations followed by expression: KLF5 20726797 ChIP-Seq SW620 Human	KLF5
TMPRSS4	Enrichr TF Perturbations followed by expression: KLF5 20726797 ChIP-Seq SW620 Human	KLF5
NR1I3	Enrichr TF Perturbations followed by expression: KLF5 20726797 ChIP-Seq SW620 Human	KLF5
TMPRSS2	Enrichr TF Perturbations followed by expression: KLF5 20726797 ChIP-Seq SW620 Human	KLF5
HNF4G	Enrichr TF Perturbations followed by expression: KLF5 20726797 ChIP-Seq SW620 Human	KLF5
SLC7A14	Enrichr TF Perturbations followed by expression: KLF5 20726797 ChIP-Seq SW620 Human	KLF5
HEPACAM2	Enrichr TF Perturbations followed by expression: KLF5 20726797 ChIP-Seq SW620 Human	KLF5
HIF3A	Enrichr TF Perturbations followed by expression: KLF5 20726797 ChIP-Seq SW620 Human	KLF5
KRT23	Enrichr TF Perturbations followed by expression: KLF5 20726797 ChIP-Seq SW620 Human	KLF5
KIF19	Enrichr TF Perturbations followed by expression: KLF5 20726797 ChIP-Seq SW620 Human	KLF5
ACACB	Enrichr TF Perturbations followed by expression: KLF5 20726797 ChIP-Seq SW620 Human	KLF5
HSD11B2	Enrichr TF Perturbations followed by expression: KLF5 20726797 ChIP-Seq SW620 Human	KLF5
SULT1C2	Enrichr TF Perturbations followed by expression: KLF5 20726797 ChIP-Seq SW620 Human	KLF5
UCHL1	Enrichr TF Perturbations followed by expression: KLF5 20726797 ChIP-Seq SW620 Human	KLF5
MECOM	Enrichr TF Perturbations followed by expression: KLF5 20726797 ChIP-Seq SW620 Human	KLF5
RNF150	Enrichr TF Perturbations followed by expression: KLF5 20726797 ChIP-Seq SW620 Human	KLF5
QSOX1	Enrichr TF Perturbations followed by expression: KLF5 20726797 ChIP-Seq SW620 Human	KLF5
PLLP	Enrichr TF Perturbations followed by expression: KLF5 20726797 ChIP-Seq SW620 Human	KLF5
RAB6B	Enrichr TF Perturbations followed by expression: KLF5 20726797 ChIP-Seq SW620 Human	KLF5
CTSE	Enrichr TF Perturbations followed by expression: KLF5 20726797 ChIP-Seq SW620 Human	KLF5
DGAT2	Enrichr TF Perturbations followed by expression: KLF5 20726797 ChIP-Seq SW620 Human	KLF5
GSTO1	Enrichr TF Perturbations followed by expression: KLF5 20726797 ChIP-Seq SW620 Human	KLF5

ANO9	Enrichr TF Perturbations followed by expression: KLF5 20726797 ChIP-Seq SW620 Human	KLF5
SYP	Enrichr TF Perturbations followed by expression: KLF5 20726797 ChIP-Seq SW620 Human	KLF5
SORBS1	Enrichr TF Perturbations followed by expression: KLF5 20726797 ChIP-Seq SW620 Human	KLF5
SLC35G2	Enrichr TF Perturbations followed by expression: KLF5 20726797 ChIP-Seq SW620 Human	KLF5
F3	Enrichr TF Perturbations followed by expression: KLF5 20726797 ChIP-Seq SW620 Human	KLF5
CLIP4	Enrichr TF Perturbations followed by expression: KLF5 20726797 ChIP-Seq SW620 Human	KLF5
DHRS9	Enrichr TF Perturbations followed by expression: KLF5 20726797 ChIP-Seq SW620 Human	KLF5
KCNQ1	Enrichr TF Perturbations followed by expression: KLF5 20726797 ChIP-Seq SW620 Human	KLF5
FABP7	Enrichr TF Perturbations followed by expression: KLF5 20726797 ChIP-Seq SW620 Human	KLF5
PPARG	Enrichr TF Perturbations followed by expression: KLF5 20726797 ChIP-Seq SW620 Human	KLF5
B3GNT3	Enrichr TF Perturbations followed by expression: KLF5 20726797 ChIP-Seq SW620 Human	KLF5
ENGASE	Enrichr TF Perturbations followed by expression: KLF5 20726797 ChIP-Seq SW620 Human	KLF5
SERPINB11	Enrichr TF Perturbations followed by expression: KLF5 20726797 ChIP-Seq SW620 Human	KLF5
PRSS27	Enrichr TF Perturbations followed by expression: KLF5 20726797 ChIP-Seq SW620 Human	KLF5
SH3RF2	Enrichr TF Perturbations followed by expression: KLF5 20726797 ChIP-Seq SW620 Human	KLF5
SMPD3	Enrichr TF Perturbations followed by expression: KLF5 20726797 ChIP-Seq SW620 Human	KLF5
ADAM28	Enrichr TF Perturbations followed by expression: KLF5 20726797 ChIP-Seq SW620 Human	KLF5
SULT1E1	Enrichr TF Perturbations followed by expression: KLF5 20726797 ChIP-Seq SW620 Human	KLF5
NEFL	Enrichr TF Perturbations followed by expression: KLF5 20726797 ChIP-Seq SW620 Human	KLF5
MKRN2OS	Enrichr TF Perturbations followed by expression: KLF5 20726797 ChIP-Seq SW620 Human	KLF5
FAM3B	Enrichr TF Perturbations followed by expression: KLF5 20726797 ChIP-Seq SW620 Human	KLF5
ENPP2	Enrichr TF Perturbations followed by expression: KLF5 20726797 ChIP-Seq SW620 Human	KLF5
GPA33	Enrichr TF Perturbations followed by expression: KLF5 20726797 ChIP-Seq SW620 Human	KLF5
MGAT3	Enrichr TF Perturbations followed by expression: KLF5 20726797 ChIP-Seq SW620 Human	KLF5
GSN	Enrichr TF Perturbations followed by expression: KLF5 20726797 ChIP-Seq SW620 Human	KLF5
ABCG8	Enrichr TF Perturbations followed by expression: KLF5 20726797 ChIP-Seq SW620 Human	KLF5
GABRP	Enrichr TF Perturbations followed by expression: KLF5 20726797 ChIP-Seq SW620 Human	KLF5
NPNT	Enrichr TF Perturbations followed by expression: KLF5 20726797 ChIP-Seq SW620 Human	KLF5
GOLM1	Enrichr TF Perturbations followed by expression: KLF5 20726797 ChIP-Seq SW620 Human	KLF5
AGT	Enrichr TF Perturbations followed by expression: KLF5 20726797 ChIP-Seq SW620 Human	KLF5
TC2N	Enrichr TF Perturbations followed by expression: KLF5 20726797 ChIP-Seq SW620 Human	KLF5
ERN2	Enrichr TF Perturbations followed by expression: KLF5 20726797 ChIP-Seq SW620 Human	KLF5
OLFM4	Enrichr TF Perturbations followed by expression: KLF5 20726797 ChIP-Seq SW620 Human	KLF5
PCP4	Enrichr TF Perturbations followed by expression: KLF5 20726797 ChIP-Seq SW620 Human	KLF5
GSTA4	Enrichr TF Perturbations followed by expression: KLF5 20726797 ChIP-Seq SW620 Human	KLF5
GSTA3	Enrichr TF Perturbations followed by expression: KLF5 20726797 ChIP-Seq SW620 Human	KLF5
S100A6	Enrichr TF Perturbations followed by expression: KLF5 20726797 ChIP-Seq SW620 Human	KLF5
LY6D	Enrichr TF Perturbations followed by expression: KLF5 20726797 ChIP-Seq SW620 Human	KLF5
ABCG5	Enrichr TF Perturbations followed by expression: KLF5 20726797 ChIP-Seq SW620 Human	KLF5
CHRNA3	Enrichr TF Perturbations followed by expression: KLF5 20726797 ChIP-Seq SW620 Human	KLF5
TNXB	Enrichr TF Perturbations followed by expression: KLF5 20726797 ChIP-Seq SW620 Human	KLF5
DGKB	Enrichr TF Perturbations followed by expression: KLF5 20726797 ChIP-Seq SW620 Human	KLF5
SCNN1A	Enrichr TF Perturbations followed by expression: KLF5 20726797 ChIP-Seq SW620 Human	KLF5
BEST2	Enrichr TF Perturbations followed by expression: KLF5 20726797 ChIP-Seq SW620 Human	KLF5
CAPN8	Enrichr TF Perturbations followed by expression: KLF5 20726797 ChIP-Seq SW620 Human	KLF5
ARHGAP40	Enrichr TF Perturbations followed by expression: KLF5 20726797 ChIP-Seq SW620 Human	KLF5
ASB11	Enrichr TF Perturbations followed by expression: KLF5 20726797 ChIP-Seq SW620 Human	KLF5
CAPN5	Enrichr TF Perturbations followed by expression: KLF5 20726797 ChIP-Seq SW620 Human	KLF5
GPC3	Enrichr TF Perturbations followed by expression: KLF5 20726797 ChIP-Seq SW620 Human	KLF5
PDE8B	Enrichr TF Perturbations followed by expression: KLF5 20726797 ChIP-Seq SW620 Human	KLF5
MYH11	Enrichr TF Perturbations followed by expression: KLF5 20726797 ChIP-Seq SW620 Human	KLF5
KCNH2	Enrichr TF Perturbations followed by expression: KLF5 20726797 ChIP-Seq SW620 Human	KLF5
SLC39A4	Enrichr TF Perturbations followed by expression: KLF5 20726797 ChIP-Seq SW620 Human	KLF5
IGFBP2	Enrichr TF Perturbations followed by expression: KLF5 20726797 ChIP-Seq SW620 Human	KLF5
LYNX1	Enrichr TF Perturbations followed by expression: KLF5 20726797 ChIP-Seq SW620 Human	KLF5
PSAPL1	Enrichr TF Perturbations followed by expression: KLF5 20726797 ChIP-Seq SW620 Human	KLF5
SYT1	Enrichr TF Perturbations followed by expression: KLF5 20726797 ChIP-Seq SW620 Human	KLF5
B3GALT5	Enrichr TF Perturbations followed by expression: KLF5 20726797 ChIP-Seq SW620 Human	KLF5
CRNDE	Enrichr TF Perturbations followed by expression: KLF5 20726797 ChIP-Seq SW620 Human	KLF5

SYT8	Enrichr TF Perturbations followed by expression: KLF5 20726797 ChIP-Seq SW620 Human	KLF5
ADRA2A	Enrichr TF Perturbations followed by expression: KLF5 20726797 ChIP-Seq SW620 Human	KLF5
TMIE	Enrichr TF Perturbations followed by expression: KLF5 20726797 ChIP-Seq SW620 Human	KLF5
REEP6	Enrichr TF Perturbations followed by expression: KLF5 20726797 ChIP-Seq SW620 Human	KLF5
MAL	Enrichr TF Perturbations followed by expression: KLF5 20726797 ChIP-Seq SW620 Human	KLF5
HPSE	Enrichr TF Perturbations followed by expression: KLF5 20726797 ChIP-Seq SW620 Human	KLF5
SPIRE2	Enrichr TF Perturbations followed by expression: KLF5 20726797 ChIP-Seq SW620 Human	KLF5
MUC20	Enrichr TF Perturbations followed by expression: KLF5 20726797 ChIP-Seq SW620 Human	KLF5
CDH19	Enrichr TF Perturbations followed by expression: KLF5 20726797 ChIP-Seq SW620 Human	KLF5
FGFR3	Enrichr TF Perturbations followed by expression: KLF5 20726797 ChIP-Seq SW620 Human	KLF5
CERS3	Enrichr TF Perturbations followed by expression: KLF5 20726797 ChIP-Seq SW620 Human	KLF5
RAB3C	Enrichr TF Perturbations followed by expression: KLF5 20726797 ChIP-Seq SW620 Human	KLF5
KCNE3	Enrichr TF Perturbations followed by expression: KLF5 20726797 ChIP-Seq SW620 Human	KLF5
KCNE4	Enrichr TF Perturbations followed by expression: KLF5 20726797 ChIP-Seq SW620 Human	KLF5
UPK2	Enrichr TF Perturbations followed by expression: KLF5 20726797 ChIP-Seq SW620 Human	KLF5
TACSTD2	Enrichr TF Perturbations followed by expression: KLF5 20726797 ChIP-Seq SW620 Human	KLF5
FAM107A	Enrichr TF Perturbations followed by expression: KLF5 20726797 ChIP-Seq SW620 Human	KLF5
PPP1R9A	Enrichr TF Perturbations followed by expression: KLF5 20726797 ChIP-Seq SW620 Human	KLF5
GPR83	Enrichr TF Perturbations followed by expression: KLF5 20726797 ChIP-Seq SW620 Human	KLF5
PLCXD3	Enrichr TF Perturbations followed by expression: KLF5 20726797 ChIP-Seq SW620 Human	KLF5
ELFN1	Enrichr TF Perturbations followed by expression: KLF5 20726797 ChIP-Seq SW620 Human	KLF5
ACTG2	Enrichr TF Perturbations followed by expression: KLF5 20726797 ChIP-Seq SW620 Human	KLF5
EFEMP1	Enrichr TF Perturbations followed by expression: KLF5 20726797 ChIP-Seq SW620 Human	KLF5
CNN1	Enrichr TF Perturbations followed by expression: KLF5 20726797 ChIP-Seq SW620 Human	KLF5
ALDH3B2	Enrichr TF Perturbations followed by expression: KLF5 20726797 ChIP-Seq SW620 Human	KLF5
B3GALT2	Enrichr TF Perturbations followed by expression: KLF5 20726797 ChIP-Seq SW620 Human	KLF5
CHAD	Enrichr TF Perturbations followed by expression: KLF5 20726797 ChIP-Seq SW620 Human	KLF5
LEPR	Enrichr TF Perturbations followed by expression: KLF5 20726797 ChIP-Seq SW620 Human	KLF5
PRODH	Enrichr TF Perturbations followed by expression: KLF5 20726797 ChIP-Seq SW620 Human	KLF5
EFHD1	Enrichr TF Perturbations followed by expression: KLF5 20726797 ChIP-Seq SW620 Human	KLF5
SH3GL2	Enrichr TF Perturbations followed by expression: KLF5 20726797 ChIP-Seq SW620 Human	KLF5
SLC37A1	Enrichr TF Perturbations followed by expression: KLF5 20726797 ChIP-Seq SW620 Human	KLF5
GALNT7	Enrichr TF Perturbations followed by expression: KLF5 20726797 ChIP-Seq SW620 Human	KLF5
GALNT5	Enrichr TF Perturbations followed by expression: KLF5 20726797 ChIP-Seq SW620 Human	KLF5
HMGCS2	Enrichr TF Perturbations followed by expression: KLF5 20726797 ChIP-Seq SW620 Human	KLF5
GALNT4	Enrichr TF Perturbations followed by expression: KLF5 20726797 ChIP-Seq SW620 Human	KLF5
PEG3	Enrichr TF Perturbations followed by expression: KLF5 20726797 ChIP-Seq SW620 Human	KLF5
MYOCD	Enrichr TF Perturbations followed by expression: KLF5 20726797 ChIP-Seq SW620 Human	KLF5
CADPS	Enrichr TF Perturbations followed by expression: KLF5 20726797 ChIP-Seq SW620 Human	KLF5
TNFRSF19	Enrichr TF Perturbations followed by expression: KLF5 20726797 ChIP-Seq SW620 Human	KLF5
INMT	Enrichr TF Perturbations followed by expression: KLF5 20726797 ChIP-Seq SW620 Human	KLF5
ELF3	Enrichr TF Perturbations followed by expression: KLF5 20726797 ChIP-Seq SW620 Human	KLF5
TNIP3	Enrichr TF Perturbations followed by expression: KLF5 20726797 ChIP-Seq SW620 Human	KLF5
GNAO1	Enrichr TF Perturbations followed by expression: KLF5 20726797 ChIP-Seq SW620 Human	KLF5
DSG3	Enrichr TF Perturbations followed by expression: KLF5 20726797 ChIP-Seq SW620 Human	KLF5
SOWAHA	Enrichr TF Perturbations followed by expression: KLF5 20726797 ChIP-Seq SW620 Human	KLF5
SNAP25	Enrichr TF Perturbations followed by expression: KLF5 20726797 ChIP-Seq SW620 Human	KLF5
ST6GALNAC1	Enrichr TF Perturbations followed by expression: KLF5 20726797 ChIP-Seq SW620 Human	KLF5
ST6GALNAC2	Enrichr TF Perturbations followed by expression: KLF5 20726797 ChIP-Seq SW620 Human	KLF5
RTN1	Enrichr TF Perturbations followed by expression: KLF5 20726797 ChIP-Seq SW620 Human	KLF5
SMIM6	Enrichr TF Perturbations followed by expression: KLF5 20726797 ChIP-Seq SW620 Human	KLF5
CRYBG2	Enrichr TF Perturbations followed by expression: KLF5 20726797 ChIP-Seq SW620 Human	KLF5
TTC22	Enrichr TF Perturbations followed by expression: KLF5 20726797 ChIP-Seq SW620 Human	KLF5
TNFAIP8	Enrichr TF Perturbations followed by expression: KLF5 20726797 ChIP-Seq SW620 Human	KLF5
NPR3	Enrichr TF Perturbations followed by expression: KLF5 20726797 ChIP-Seq SW620 Human	KLF5
ITPR2	Enrichr TF Perturbations followed by expression: KLF5 20726797 ChIP-Seq SW620 Human	KLF5
FBXO44	Enrichr TF Perturbations followed by expression: KLF5 20726797 ChIP-Seq SW620 Human	KLF5
RASGEF1A	Enrichr TF Perturbations followed by expression: KLF5 20726797 ChIP-Seq SW620 Human	KLF5
FUT8	Enrichr TF Perturbations followed by expression: KLF5 20726797 ChIP-Seq SW620 Human	KLF5

UPK1B	Enrichr TF Perturbations followed by expression: KLF5 20726797 ChIP-Seq SW620 Human	KLF5
RDH16	Enrichr TF Perturbations followed by expression: KLF5 20726797 ChIP-Seq SW620 Human	KLF5
CPXM2	Enrichr TF Perturbations followed by expression: KLF5 20726797 ChIP-Seq SW620 Human	KLF5
SYMPK	Enrichr TF Perturbations followed by expression: KLF5 20726797 ChIP-Seq SW620 Human	KLF5
CASP1	Enrichr TF Perturbations followed by expression: KLF5 20726797 ChIP-Seq SW620 Human	KLF5
FAM83E	Enrichr TF Perturbations followed by expression: KLF5 20726797 ChIP-Seq SW620 Human	KLF5
PRPH	Enrichr TF Perturbations followed by expression: KLF5 20726797 ChIP-Seq SW620 Human	KLF5
WNT4	Enrichr TF Perturbations followed by expression: KLF5 20726797 ChIP-Seq SW620 Human	KLF5
HGFAC	Enrichr TF Perturbations followed by expression: KLF5 20726797 ChIP-Seq SW620 Human	KLF5
TBX1	Enrichr TF Perturbations followed by expression: KLF5 20726797 ChIP-Seq SW620 Human	KLF5
CAMK1D	Enrichr TF Perturbations followed by expression: KLF5 20726797 ChIP-Seq SW620 Human	KLF5
MUC5B	Enrichr TF Perturbations followed by expression: KLF5 20726797 ChIP-Seq SW620 Human	KLF5
STYK1	Enrichr TF Perturbations followed by expression: KLF5 20726797 ChIP-Seq SW620 Human	KLF5
DCXR	Enrichr TF Perturbations followed by expression: KLF5 20726797 ChIP-Seq SW620 Human	KLF5
SYT16	Enrichr TF Perturbations followed by expression: KLF5 20726797 ChIP-Seq SW620 Human	KLF5
MCOLN2	Enrichr TF Perturbations followed by expression: KLF5 20726797 ChIP-Seq SW620 Human	KLF5
KLF5	Enrichr TF Perturbations followed by expression: KLF5 20726797 ChIP-Seq SW620 Human	KLF5
HTR3A	Enrichr TF Perturbations followed by expression: KLF5 20726797 ChIP-Seq SW620 Human	KLF5
HSH2D	Enrichr TF Perturbations followed by expression: KLF5 20726797 ChIP-Seq SW620 Human	KLF5
TST	Enrichr TF Perturbations followed by expression: KLF5 20726797 ChIP-Seq SW620 Human	KLF5
P2RX1	Enrichr TF Perturbations followed by expression: KLF5 20726797 ChIP-Seq SW620 Human	KLF5
LTF	Enrichr TF Perturbations followed by expression: KLF5 20726797 ChIP-Seq SW620 Human	KLF5
ASPA	Enrichr TF Perturbations followed by expression: KLF5 20726797 ChIP-Seq SW620 Human	KLF5
SLC24A3	Enrichr TF Perturbations followed by expression: KLF5 20726797 ChIP-Seq SW620 Human	KLF5
SH2D4A	Enrichr TF Perturbations followed by expression: KLF5 20726797 ChIP-Seq SW620 Human	KLF5
MSLN	Enrichr TF Perturbations followed by expression: KLF5 20726797 ChIP-Seq SW620 Human	KLF5
TRIM31	Enrichr TF Perturbations followed by expression: KLF5 20726797 ChIP-Seq SW620 Human	KLF5
PSCA	Enrichr TF Perturbations followed by expression: KLF5 20726797 ChIP-Seq SW620 Human	KLF5
SEMA3C	Enrichr TF Perturbations followed by expression: KLF5 20726797 ChIP-Seq SW620 Human	KLF5
ATP2A3	Enrichr TF Perturbations followed by expression: KLF5 20726797 ChIP-Seq SW620 Human	KLF5
HSD17B14	Enrichr TF Perturbations followed by expression: KLF5 20726797 ChIP-Seq SW620 Human	KLF5
CLCN2	Enrichr TF Perturbations followed by expression: KLF5 20726797 ChIP-Seq SW620 Human	KLF5
TPRN	Enrichr TF Perturbations followed by expression: KLF5 20726797 ChIP-Seq SW620 Human	KLF5
PTPRZ1	Enrichr TF Perturbations followed by expression: KLF5 20726797 ChIP-Seq SW620 Human	KLF5
NT5DC3	Enrichr TF Perturbations followed by expression: KLF5 20726797 ChIP-Seq SW620 Human	KLF5
VSIG1	Enrichr TF Perturbations followed by expression: KLF5 20726797 ChIP-Seq SW620 Human	KLF5
NOS1	Enrichr TF Perturbations followed by expression: KLF5 20726797 ChIP-Seq SW620 Human	KLF5
DLGAP1	SOX2-KLF5 coregulated genes from PMID: 33972779	SOX2 + KLF[illegible]
SLC35E4	SOX2-KLF5 coregulated genes from PMID: 33972779	SOX2 + KLF[illegible]
USP12	SOX2-KLF5 coregulated genes from PMID: 33972779	SOX2 + KLF[illegible]
CARD10	SOX2-KLF5 coregulated genes from PMID: 33972779	SOX2 + KLF[illegible]
KLF5	SOX2-KLF5 coregulated genes from PMID: 33972779	SOX2 + KLF[illegible]
CUBN	SOX2-KLF5 coregulated genes from PMID: 33972779	SOX2 + KLF[illegible]
HS3ST1	SOX2-KLF5 coregulated genes from PMID: 33972779	SOX2 + KLF[illegible]
TMEM47	SOX2-KLF5 coregulated genes from PMID: 33972779	SOX2 + KLF[illegible]
RUNX1	SOX2-KLF5 coregulated genes from PMID: 33972779	SOX2 + KLF[illegible]
JAG1	SOX2-KLF5 coregulated genes from PMID: 33972779	SOX2 + KLF[illegible]
PLCB1	SOX2-KLF5 coregulated genes from PMID: 33972779	SOX2 + KLF[illegible]
PCDH17	SOX2-KLF5 coregulated genes from PMID: 33972779	SOX2 + KLF[illegible]
KCTD1	SOX2-KLF5 coregulated genes from PMID: 33972779	SOX2 + KLF[illegible]
DSG3	SOX2-KLF5 coregulated genes from PMID: 33972779	SOX2 + KLF[illegible]
BMP2	SOX2-KLF5 coregulated genes from PMID: 33972779	SOX2 + KLF[illegible]
PTGER2	SOX2-KLF5 coregulated genes from PMID: 33972779	SOX2 + KLF[illegible]
SLC5A3	SOX2-KLF5 coregulated genes from PMID: 33972779	SOX2 + KLF[illegible]
AKAP6	SOX2-KLF5 coregulated genes from PMID: 33972779	SOX2 + KLF[illegible]
SH3GL2	SOX2-KLF5 coregulated genes from PMID: 33972779	SOX2 + KLF[illegible]
TTC39C	SOX2-KLF5 coregulated genes from PMID: 33972779	SOX2 + KLF[illegible]
NRIP1	SOX2-KLF5 coregulated genes from PMID: 33972779	SOX2 + KLF[illegible]
OCIAD2	SOX2-KLF5 coregulated genes from PMID: 33972779	SOX2 + KLF[illegible]

KIAA1328	SOX2-KLF5 coregulated genes from PMID: 33972779	SOX2 + KLF5
ETS2	SOX2-KLF5 coregulated genes from PMID: 33972779	SOX2 + KLF5
SLC1A1	SOX2-KLF5 coregulated genes from PMID: 33972779	SOX2 + KLF5
LMO7	SOX2-KLF5 coregulated genes from PMID: 33972779	SOX2 + KLF5
BTG3	SOX2-KLF5 coregulated genes from PMID: 33972779	SOX2 + KLF5
DACH1	SOX2-KLF5 coregulated genes from PMID: 33972779	SOX2 + KLF5
MIPEP	SOX2-KLF5 coregulated genes from PMID: 33972779	SOX2 + KLF5
FERMT2	SOX2-KLF5 coregulated genes from PMID: 33972779	SOX2 + KLF5
SLIT2	SOX2-KLF5 coregulated genes from PMID: 33972779	SOX2 + KLF5
MAPRE2	SOX2-KLF5 coregulated genes from PMID: 33972779	SOX2 + KLF5
FUNDC1	SOX2-KLF5 coregulated genes from PMID: 33972779	SOX2 + KLF5
NFATC2	SOX2-KLF5 coregulated genes from PMID: 33972779	SOX2 + KLF5
TXNDC16	SOX2-KLF5 coregulated genes from PMID: 33972779	SOX2 + KLF5
ALG13	SOX2-KLF5 coregulated genes from PMID: 33972779	SOX2 + KLF5
RAB27B	SOX2-KLF5 coregulated genes from PMID: 33972779	SOX2 + KLF5
SPATA13	SOX2-KLF5 coregulated genes from PMID: 33972779	SOX2 + KLF5
NOVA1	SOX2-KLF5 coregulated genes from PMID: 33972779	SOX2 + KLF5
BACE2	SOX2-KLF5 coregulated genes from PMID: 33972779	SOX2 + KLF5
CYYR1	SOX2-KLF5 coregulated genes from PMID: 33972779	SOX2 + KLF5
FUT8	SOX2-KLF5 coregulated genes from PMID: 33972779	SOX2 + KLF5
TRMT2B	SOX2-KLF5 coregulated genes from PMID: 33972779	SOX2 + KLF5
SIK1B	SOX2-KLF5 coregulated genes from PMID: 33972779	SOX2 + KLF5
SIK1	SOX2-KLF5 coregulated genes from PMID: 33972779	SOX2 + KLF5
CNTLN	SOX2-KLF5 coregulated genes from PMID: 33972779	SOX2 + KLF5
LINGO2	SOX2-KLF5 coregulated genes from PMID: 33972779	SOX2 + KLF5
APBB2	SOX2-KLF5 coregulated genes from PMID: 33972779	SOX2 + KLF5
SERPINB2	SOX2-KLF5 coregulated genes from PMID: 33972779	SOX2 + KLF5
SHROOM2	SOX2-KLF5 coregulated genes from PMID: 33972779	SOX2 + KLF5
GNA14	SOX2-KLF5 coregulated genes from PMID: 33972779	SOX2 + KLF5
TULP3	SOX2-KLF5 coregulated genes from PMID: 33972779	SOX2 + KLF5
VOPP1	SOX2-KLF5 coregulated genes from PMID: 33972779	SOX2 + KLF5
DOCK11	SOX2-KLF5 coregulated genes from PMID: 33972779	SOX2 + KLF5
SETBP1	SOX2-KLF5 coregulated genes from PMID: 33972779	SOX2 + KLF5
GCNT3	SOX2-KLF5 coregulated genes from PMID: 33972779	SOX2 + KLF5
OSBPL3	SOX2-KLF5 coregulated genes from PMID: 33972779	SOX2 + KLF5
ANXA11	SOX2-KLF5 coregulated genes from PMID: 33972779	SOX2 + KLF5
GREB1L	SOX2-KLF5 coregulated genes from PMID: 33972779	SOX2 + KLF5
KLF3	SOX2-KLF5 coregulated genes from PMID: 33972779	SOX2 + KLF5
RSU1	SOX2-KLF5 coregulated genes from PMID: 33972779	SOX2 + KLF5
ATRNL1	SOX2-KLF5 coregulated genes from PMID: 33972779	SOX2 + KLF5
B4GALNT3	SOX2-KLF5 coregulated genes from PMID: 33972779	SOX2 + KLF5
MLLT3	SOX2-KLF5 coregulated genes from PMID: 33972779	SOX2 + KLF5
UGT8	SOX2-KLF5 coregulated genes from PMID: 33972779	SOX2 + KLF5
SHROOM3	SOX2-KLF5 coregulated genes from PMID: 33972779	SOX2 + KLF5
TMTC1	SOX2-KLF5 coregulated genes from PMID: 33972779	SOX2 + KLF5
DCLK2	SOX2-KLF5 coregulated genes from PMID: 33972779	SOX2 + KLF5
DUSP14	SOX2-KLF5 coregulated genes from PMID: 33972779	SOX2 + KLF5
SAMD4A	SOX2-KLF5 coregulated genes from PMID: 33972779	SOX2 + KLF5
NDUFA4	SOX2-KLF5 coregulated genes from PMID: 33972779	SOX2 + KLF5
TGFBR1	SOX2-KLF5 coregulated genes from PMID: 33972779	SOX2 + KLF5
APLN	SOX2-KLF5 coregulated genes from PMID: 33972779	SOX2 + KLF5
CALCB	SOX2-KLF5 coregulated genes from PMID: 33972779	SOX2 + KLF5
LMTK2	SOX2-KLF5 coregulated genes from PMID: 33972779	SOX2 + KLF5
FMN1	SOX2-KLF5 coregulated genes from PMID: 33972779	SOX2 + KLF5
TNS4	SOX2-KLF5 coregulated genes from PMID: 33972779	SOX2 + KLF5
TNFAIP2	SOX2-KLF5 coregulated genes from PMID: 33972779	SOX2 + KLF5
MOXD1	SOX2-KLF5 coregulated genes from PMID: 33972779	SOX2 + KLF5
MACROD2	SOX2-KLF5 coregulated genes from PMID: 33972779	SOX2 + KLF5
LNX1	SOX2-KLF5 coregulated genes from PMID: 33972779	SOX2 + KLF5

ETV1	SOX2-KLF5 coregulated genes from PMID: 33972779	SOX2 + KLF[illegible]
CCNG2	SOX2-KLF5 coregulated genes from PMID: 33972779	SOX2 + KLF[illegible]
ST8SIA6	SOX2-KLF5 coregulated genes from PMID: 33972779	SOX2 + KLF[illegible]
RNF150	SOX2-KLF5 coregulated genes from PMID: 33972779	SOX2 + KLF[illegible]
STARD13	SOX2-KLF5 coregulated genes from PMID: 33972779	SOX2 + KLF[illegible]
IPMK	SOX2-KLF5 coregulated genes from PMID: 33972779	SOX2 + KLF[illegible]
NCOA7	SOX2-KLF5 coregulated genes from PMID: 33972779	SOX2 + KLF[illegible]
ATP8B4	SOX2-KLF5 coregulated genes from PMID: 33972779	SOX2 + KLF[illegible]
PLIN2	SOX2-KLF5 coregulated genes from PMID: 33972779	SOX2 + KLF[illegible]
AHNAK	SOX2-KLF5 coregulated genes from PMID: 33972779	SOX2 + KLF[illegible]
RAB11FIP2	SOX2-KLF5 coregulated genes from PMID: 33972779	SOX2 + KLF[illegible]
STK38	SOX2-KLF5 coregulated genes from PMID: 33972779	SOX2 + KLF[illegible]
ARMC2	SOX2-KLF5 coregulated genes from PMID: 33972779	SOX2 + KLF[illegible]
AVPI1	SOX2-KLF5 coregulated genes from PMID: 33972779	SOX2 + KLF[illegible]
TNIP3	SOX2-KLF5 coregulated genes from PMID: 33972779	SOX2 + KLF[illegible]
MYO10	SOX2-KLF5 coregulated genes from PMID: 33972779	SOX2 + KLF[illegible]
EPDR1	SOX2-KLF5 coregulated genes from PMID: 33972779	SOX2 + KLF[illegible]
RASSF6	SOX2-KLF5 coregulated genes from PMID: 33972779	SOX2 + KLF[illegible]
BMP6	SOX2-KLF5 coregulated genes from PMID: 33972779	SOX2 + KLF[illegible]
RUNX2	SOX2-KLF5 coregulated genes from PMID: 33972779	SOX2 + KLF[illegible]
PAPPA	SOX2-KLF5 coregulated genes from PMID: 33972779	SOX2 + KLF[illegible]
FOXF2	SOX2-KLF5 coregulated genes from PMID: 33972779	SOX2 + KLF[illegible]
APCDD1	SOX2-KLF5 coregulated genes from PMID: 33972779	SOX2 + KLF[illegible]
IL15	SOX2-KLF5 coregulated genes from PMID: 33972779	SOX2 + KLF[illegible]
FAM171A1	SOX2-KLF5 coregulated genes from PMID: 33972779	SOX2 + KLF[illegible]
GRID2	SOX2-KLF5 coregulated genes from PMID: 33972779	SOX2 + KLF[illegible]
PCDH7	SOX2-KLF5 coregulated genes from PMID: 33972779	SOX2 + KLF[illegible]
FARP1	SOX2-KLF5 coregulated genes from PMID: 33972779	SOX2 + KLF[illegible]
TMPRSS11D	SOX2-KLF5 coregulated genes from PMID: 33972779	SOX2 + KLF[illegible]
XPNPEP2	SOX2-KLF5 coregulated genes from PMID: 33972779	SOX2 + KLF[illegible]
CTBP2	SOX2-KLF5 coregulated genes from PMID: 33972779	SOX2 + KLF[illegible]
SUSD1	SOX2-KLF5 coregulated genes from PMID: 33972779	SOX2 + KLF[illegible]
ITGB8	SOX2-KLF5 coregulated genes from PMID: 33972779	SOX2 + KLF[illegible]
MICAL3	SOX2-KLF5 coregulated genes from PMID: 33972779	SOX2 + KLF[illegible]
BCAT1	SOX2-KLF5 coregulated genes from PMID: 33972779	SOX2 + KLF[illegible]
MYOF	SOX2-KLF5 coregulated genes from PMID: 33972779	SOX2 + KLF[illegible]
SDCBP2	SOX2-KLF5 coregulated genes from PMID: 33972779	SOX2 + KLF[illegible]
MARCKS	SOX2-KLF5 coregulated genes from PMID: 33972779	SOX2 + KLF[illegible]
PC	SOX2-KLF5 coregulated genes from PMID: 33972779	SOX2 + KLF[illegible]
PKIB	SOX2-KLF5 coregulated genes from PMID: 33972779	SOX2 + KLF[illegible]
ATP8B1	SOX2-KLF5 coregulated genes from PMID: 33972779	SOX2 + KLF[illegible]
APBB1IP	SOX2-KLF5 coregulated genes from PMID: 33972779	SOX2 + KLF[illegible]
LAMA3	SOX2-KLF5 coregulated genes from PMID: 33972779	SOX2 + KLF[illegible]
TSPAN14	SOX2-KLF5 coregulated genes from PMID: 33972779	SOX2 + KLF[illegible]
BACH2	SOX2-KLF5 coregulated genes from PMID: 33972779	SOX2 + KLF[illegible]
MKX	SOX2-KLF5 coregulated genes from PMID: 33972779	SOX2 + KLF[illegible]
RRP12	SOX2-KLF5 coregulated genes from PMID: 33972779	SOX2 + KLF[illegible]
SULT1B1	SOX2-KLF5 coregulated genes from PMID: 33972779	SOX2 + KLF[illegible]
PIM1	SOX2-KLF5 coregulated genes from PMID: 33972779	SOX2 + KLF[illegible]
AQP11	SOX2-KLF5 coregulated genes from PMID: 33972779	SOX2 + KLF[illegible]
ZNF395	SOX2-KLF5 coregulated genes from PMID: 33972779	SOX2 + KLF[illegible]
DIRAS2	SOX2-KLF5 coregulated genes from PMID: 33972779	SOX2 + KLF[illegible]
RNF24	SOX2-KLF5 coregulated genes from PMID: 33972779	SOX2 + KLF[illegible]
EDN1	SOX2-KLF5 coregulated genes from PMID: 33972779	SOX2 + KLF[illegible]
SH3RF1	SOX2-KLF5 coregulated genes from PMID: 33972779	SOX2 + KLF[illegible]
FNBP1	SOX2-KLF5 coregulated genes from PMID: 33972779	SOX2 + KLF[illegible]
OPHN1	SOX2-KLF5 coregulated genes from PMID: 33972779	SOX2 + KLF[illegible]
AGR3	SOX2-KLF5 coregulated genes from PMID: 33972779	SOX2 + KLF[illegible]
HTRA1	SOX2-KLF5 coregulated genes from PMID: 33972779	SOX2 + KLF[illegible]

GFRA2	SOX2-KLF5 coregulated genes from PMID: 33972779	SOX2 + KLF
TNC	SOX2-KLF5 coregulated genes from PMID: 33972779	SOX2 + KLF
CTSZ	SOX2-KLF5 coregulated genes from PMID: 33972779	SOX2 + KLF
TSPAN5	SOX2-KLF5 coregulated genes from PMID: 33972779	SOX2 + KLF
SERPINB5	SOX2-KLF5 coregulated genes from PMID: 33972779	SOX2 + KLF
LPCAT1	SOX2-KLF5 coregulated genes from PMID: 33972779	SOX2 + KLF
DCLK1	SOX2-KLF5 coregulated genes from PMID: 33972779	SOX2 + KLF
PPP3CA	SOX2-KLF5 coregulated genes from PMID: 33972779	SOX2 + KLF
SLC7A11	SOX2-KLF5 coregulated genes from PMID: 33972779	SOX2 + KLF
TLE1	SOX2-KLF5 coregulated genes from PMID: 33972779	SOX2 + KLF
ANK3	SOX2-KLF5 coregulated genes from PMID: 33972779	SOX2 + KLF
PIP5K1B	SOX2-KLF5 coregulated genes from PMID: 33972779	SOX2 + KLF
GRIK1	SOX2-KLF5 coregulated genes from PMID: 33972779	SOX2 + KLF
TADA2A	SOX2-KLF5 coregulated genes from PMID: 33972779	SOX2 + KLF
PARM1	SOX2-KLF5 coregulated genes from PMID: 33972779	SOX2 + KLF
PRRG4	SOX2-KLF5 coregulated genes from PMID: 33972779	SOX2 + KLF
SOD3	SOX2-KLF5 coregulated genes from PMID: 33972779	SOX2 + KLF
FAM83B	SOX2-KLF5 coregulated genes from PMID: 33972779	SOX2 + KLF
FAM53B	SOX2-KLF5 coregulated genes from PMID: 33972779	SOX2 + KLF
RAB11FIP1	SOX2-KLF5 coregulated genes from PMID: 33972779	SOX2 + KLF
CYTH3	SOX2-KLF5 coregulated genes from PMID: 33972779	SOX2 + KLF
ENKUR	SOX2-KLF5 coregulated genes from PMID: 33972779	SOX2 + KLF
NRP1	SOX2-KLF5 coregulated genes from PMID: 33972779	SOX2 + KLF
OSBPL2	SOX2-KLF5 coregulated genes from PMID: 33972779	SOX2 + KLF
SEMA5A	SOX2-KLF5 coregulated genes from PMID: 33972779	SOX2 + KLF
RCBTB2	SOX2-KLF5 coregulated genes from PMID: 33972779	SOX2 + KLF
FOXQ1	SOX2-KLF5 coregulated genes from PMID: 33972779	SOX2 + KLF
RAP1GAP2	SOX2-KLF5 coregulated genes from PMID: 33972779	SOX2 + KLF
ARL4A	SOX2-KLF5 coregulated genes from PMID: 33972779	SOX2 + KLF
KLF4	SOX2-KLF5 coregulated genes from PMID: 33972779	SOX2 + KLF
CHST3	SOX2-KLF5 coregulated genes from PMID: 33972779	SOX2 + KLF
SLC16A7	SOX2-KLF5 coregulated genes from PMID: 33972779	SOX2 + KLF
GPR158	SOX2-KLF5 coregulated genes from PMID: 33972779	SOX2 + KLF
ARHGAP10	SOX2-KLF5 coregulated genes from PMID: 33972779	SOX2 + KLF
ABCC4	SOX2-KLF5 coregulated genes from PMID: 33972779	SOX2 + KLF
ZFAND5	SOX2-KLF5 coregulated genes from PMID: 33972779	SOX2 + KLF
RIN2	SOX2-KLF5 coregulated genes from PMID: 33972779	SOX2 + KLF
DSC3	SOX2-KLF5 coregulated genes from PMID: 33972779	SOX2 + KLF
SOX21	SOX2-KLF5 coregulated genes from PMID: 33972779	SOX2 + KLF
FRAT2	SOX2-KLF5 coregulated genes from PMID: 33972779	SOX2 + KLF
ANXA1	SOX2-KLF5 coregulated genes from PMID: 33972779	SOX2 + KLF
FCHSD2	SOX2-KLF5 coregulated genes from PMID: 33972779	SOX2 + KLF
TMPRSS11E	SOX2-KLF5 coregulated genes from PMID: 33972779	SOX2 + KLF
SLITRK6	SOX2-KLF5 coregulated genes from PMID: 33972779	SOX2 + KLF
PALLD	SOX2-KLF5 coregulated genes from PMID: 33972779	SOX2 + KLF
ZNF451	SOX2-KLF5 coregulated genes from PMID: 33972779	SOX2 + KLF
PDE1C	SOX2-KLF5 coregulated genes from PMID: 33972779	SOX2 + KLF
EFNB2	SOX2-KLF5 coregulated genes from PMID: 33972779	SOX2 + KLF
COL15A1	SOX2-KLF5 coregulated genes from PMID: 33972779	SOX2 + KLF
KLHL32	SOX2-KLF5 coregulated genes from PMID: 33972779	SOX2 + KLF
ASAH1	SOX2-KLF5 coregulated genes from PMID: 33972779	SOX2 + KLF
CHIC1	SOX2-KLF5 coregulated genes from PMID: 33972779	SOX2 + KLF
JUP	SOX2-KLF5 coregulated genes from PMID: 33972779	SOX2 + KLF
NDFIP2	SOX2-KLF5 coregulated genes from PMID: 33972779	SOX2 + KLF
POLM	SOX2-KLF5 coregulated genes from PMID: 33972779	SOX2 + KLF
TMEM211	SOX2-KLF5 coregulated genes from PMID: 33972779	SOX2 + KLF
SLC1A4	SOX2-KLF5 coregulated genes from PMID: 33972779	SOX2 + KLF
TSPAN8	SOX2-KLF5 coregulated genes from PMID: 33972779	SOX2 + KLF
TRIP10	SOX2-KLF5 coregulated genes from PMID: 33972779	SOX2 + KLF

PCDH18	SOX2-KLF5 coregulated genes from PMID: 33972779	SOX2 + KLF5
PPP1CB	SOX2-KLF5 coregulated genes from PMID: 33972779	SOX2 + KLF5
CA10	SOX2-KLF5 coregulated genes from PMID: 33972779	SOX2 + KLF5
SYNE2	SOX2-KLF5 coregulated genes from PMID: 33972779	SOX2 + KLF5
CECR2	SOX2-KLF5 coregulated genes from PMID: 33972779	SOX2 + KLF5
DOCK9	SOX2-KLF5 coregulated genes from PMID: 33972779	SOX2 + KLF5
SULF1	SOX2-KLF5 coregulated genes from PMID: 33972779	SOX2 + KLF5
WDR72	SOX2-KLF5 coregulated genes from PMID: 33972779	SOX2 + KLF5
RAP2A	SOX2-KLF5 coregulated genes from PMID: 33972779	SOX2 + KLF5
KIRREL3	SOX2-KLF5 coregulated genes from PMID: 33972779	SOX2 + KLF5
EXOC4	SOX2-KLF5 coregulated genes from PMID: 33972779	SOX2 + KLF5
LY75	SOX2-KLF5 coregulated genes from PMID: 33972779	SOX2 + KLF5
LAMA4	SOX2-KLF5 coregulated genes from PMID: 33972779	SOX2 + KLF5
IGSF11	SOX2-KLF5 coregulated genes from PMID: 33972779	SOX2 + KLF5
FRAS1	SOX2-KLF5 coregulated genes from PMID: 33972779	SOX2 + KLF5
DENND1A	SOX2-KLF5 coregulated genes from PMID: 33972779	SOX2 + KLF5
MAPK10	SOX2-KLF5 coregulated genes from PMID: 33972779	SOX2 + KLF5
TGM3	SOX2-KLF5 coregulated genes from PMID: 33972779	SOX2 + KLF5
TMPRSS4	SOX2-KLF5 coregulated genes from PMID: 33972779	SOX2 + KLF5
SNRPN	SOX2-KLF5 coregulated genes from PMID: 33972779	SOX2 + KLF5
DPYSL3	SOX2-KLF5 coregulated genes from PMID: 33972779	SOX2 + KLF5
C6orf136	SOX2-KLF5 coregulated genes from PMID: 33972779	SOX2 + KLF5
BNC2	SOX2-KLF5 coregulated genes from PMID: 33972779	SOX2 + KLF5
TANK	SOX2-KLF5 coregulated genes from PMID: 33972779	SOX2 + KLF5
TANC1	SOX2-KLF5 coregulated genes from PMID: 33972779	SOX2 + KLF5
NOL10	SOX2-KLF5 coregulated genes from PMID: 33972779	SOX2 + KLF5
AKAP12	SOX2-KLF5 coregulated genes from PMID: 33972779	SOX2 + KLF5
SEMA6D	SOX2-KLF5 coregulated genes from PMID: 33972779	SOX2 + KLF5
RIMS2	SOX2-KLF5 coregulated genes from PMID: 33972779	SOX2 + KLF5
GDNF	SOX2-KLF5 coregulated genes from PMID: 33972779	SOX2 + KLF5
FIGN	SOX2-KLF5 coregulated genes from PMID: 33972779	SOX2 + KLF5
WFDC1	SOX2-KLF5 coregulated genes from PMID: 33972779	SOX2 + KLF5
SIL1	SOX2-KLF5 coregulated genes from PMID: 33972779	SOX2 + KLF5
BARX2	SOX2-KLF5 coregulated genes from PMID: 33972779	SOX2 + KLF5
WNT7A	SOX2-KLF5 coregulated genes from PMID: 33972779	SOX2 + KLF5
MACC1	SOX2-KLF5 coregulated genes from PMID: 33972779	SOX2 + KLF5
CITED2	SOX2-KLF5 coregulated genes from PMID: 33972779	SOX2 + KLF5
FBXO16	SOX2-KLF5 coregulated genes from PMID: 33972779	SOX2 + KLF5
TGFBI	SOX2-KLF5 coregulated genes from PMID: 33972779	SOX2 + KLF5
ASB1	SOX2-KLF5 coregulated genes from PMID: 33972779	SOX2 + KLF5
ATP8A1	SOX2-KLF5 coregulated genes from PMID: 33972779	SOX2 + KLF5
CCDC85A	SOX2-KLF5 coregulated genes from PMID: 33972779	SOX2 + KLF5
OSGIN1	SOX2-KLF5 coregulated genes from PMID: 33972779	SOX2 + KLF5
WNK2	SOX2-KLF5 coregulated genes from PMID: 33972779	SOX2 + KLF5
THUMPD2	SOX2-KLF5 coregulated genes from PMID: 33972779	SOX2 + KLF5
SLC8A1	SOX2-KLF5 coregulated genes from PMID: 33972779	SOX2 + KLF5
N4BP1	SOX2-KLF5 coregulated genes from PMID: 33972779	SOX2 + KLF5
PRKCH	SOX2-KLF5 coregulated genes from PMID: 33972779	SOX2 + KLF5
PLS3	SOX2-KLF5 coregulated genes from PMID: 33972779	SOX2 + KLF5
PPIF	SOX2-KLF5 coregulated genes from PMID: 33972779	SOX2 + KLF5
AIG1	SOX2-KLF5 coregulated genes from PMID: 33972779	SOX2 + KLF5
VCL	SOX2-KLF5 coregulated genes from PMID: 33972779	SOX2 + KLF5
NRXN3	SOX2-KLF5 coregulated genes from PMID: 33972779	SOX2 + KLF5
GSS	SOX2-KLF5 coregulated genes from PMID: 33972779	SOX2 + KLF5
ARHGAP26	SOX2-KLF5 coregulated genes from PMID: 33972779	SOX2 + KLF5
RPS6KA3	SOX2-KLF5 coregulated genes from PMID: 33972779	SOX2 + KLF5
SLC16A9	SOX2-KLF5 coregulated genes from PMID: 33972779	SOX2 + KLF5
RGS9	SOX2-KLF5 coregulated genes from PMID: 33972779	SOX2 + KLF5
RERE	SOX2-KLF5 coregulated genes from PMID: 33972779	SOX2 + KLF5

FBLN2	SOX2-KLF5 coregulated genes from PMID: 33972779	SOX2 + KLF[illegible]
MEIS1	SOX2-KLF5 coregulated genes from PMID: 33972779	SOX2 + KLF[illegible]
LY6E	SOX2-KLF5 coregulated genes from PMID: 33972779	SOX2 + KLF[illegible]
COL8A1	SOX2-KLF5 coregulated genes from PMID: 33972779	SOX2 + KLF[illegible]
KCMF1	SOX2-KLF5 coregulated genes from PMID: 33972779	SOX2 + KLF[illegible]
PARP12	SOX2-KLF5 coregulated genes from PMID: 33972779	SOX2 + KLF[illegible]
SLCO5A1	SOX2-KLF5 coregulated genes from PMID: 33972779	SOX2 + KLF[illegible]
SMPD3	SOX2-KLF5 coregulated genes from PMID: 33972779	SOX2 + KLF[illegible]
SLC4A4	SOX2-KLF5 coregulated genes from PMID: 33972779	SOX2 + KLF[illegible]
FHL2	SOX2-KLF5 coregulated genes from PMID: 33972779	SOX2 + KLF[illegible]
SLC44A3	SOX2-KLF5 coregulated genes from PMID: 33972779	SOX2 + KLF[illegible]
SYN2	SOX2-KLF5 coregulated genes from PMID: 33972779	SOX2 + KLF[illegible]
GLI3	SOX2-KLF5 coregulated genes from PMID: 33972779	SOX2 + KLF[illegible]
TRIM24	SOX2-KLF5 coregulated genes from PMID: 33972779	SOX2 + KLF[illegible]
ENPP2	SOX2-KLF5 coregulated genes from PMID: 33972779	SOX2 + KLF[illegible]
RFX3	SOX2-KLF5 coregulated genes from PMID: 33972779	SOX2 + KLF[illegible]
SCARA5	SOX2-KLF5 coregulated genes from PMID: 33972779	SOX2 + KLF[illegible]
EMB	SOX2-KLF5 coregulated genes from PMID: 33972779	SOX2 + KLF[illegible]
MMP2	SOX2-KLF5 coregulated genes from PMID: 33972779	SOX2 + KLF[illegible]
PGM2L1	SOX2-KLF5 coregulated genes from PMID: 33972779	SOX2 + KLF[illegible]
MGAT4A	SOX2-KLF5 coregulated genes from PMID: 33972779	SOX2 + KLF[illegible]
FSD1L	SOX2-KLF5 coregulated genes from PMID: 33972779	SOX2 + KLF[illegible]
PLEKHH1	SOX2-KLF5 coregulated genes from PMID: 33972779	SOX2 + KLF[illegible]
FLRT2	SOX2-KLF5 coregulated genes from PMID: 33972779	SOX2 + KLF[illegible]
AMOT	SOX2-KLF5 coregulated genes from PMID: 33972779	SOX2 + KLF[illegible]
FAM81A	SOX2-KLF5 coregulated genes from PMID: 33972779	SOX2 + KLF[illegible]
DNMBP	SOX2-KLF5 coregulated genes from PMID: 33972779	SOX2 + KLF[illegible]
EPHB6	SOX2-KLF5 coregulated genes from PMID: 33972779	SOX2 + KLF[illegible]
STXBP1	SOX2-KLF5 coregulated genes from PMID: 33972779	SOX2 + KLF[illegible]
NEBL	SOX2-KLF5 coregulated genes from PMID: 33972779	SOX2 + KLF[illegible]
IGF2R	SOX2-KLF5 coregulated genes from PMID: 33972779	SOX2 + KLF[illegible]
ZFHX3	SOX2-KLF5 coregulated genes from PMID: 33972779	SOX2 + KLF[illegible]
TLE4	SOX2-KLF5 coregulated genes from PMID: 33972779	SOX2 + KLF[illegible]
GJB5	SOX2-KLF5 coregulated genes from PMID: 33972779	SOX2 + KLF[illegible]
KLF12	SOX2-KLF5 coregulated genes from PMID: 33972779	SOX2 + KLF[illegible]
RAI14	SOX2-KLF5 coregulated genes from PMID: 33972779	SOX2 + KLF[illegible]
PBRM1	SOX2-KLF5 coregulated genes from PMID: 33972779	SOX2 + KLF[illegible]
ILVBL	SOX2-KLF5 coregulated genes from PMID: 33972779	SOX2 + KLF[illegible]
PCTP	SOX2-KLF5 coregulated genes from PMID: 33972779	SOX2 + KLF[illegible]
SOX5	SOX2-KLF5 coregulated genes from PMID: 33972779	SOX2 + KLF[illegible]
NCOR2	SOX2-KLF5 coregulated genes from PMID: 33972779	SOX2 + KLF[illegible]
RAPGEF5	SOX2-KLF5 coregulated genes from PMID: 33972779	SOX2 + KLF[illegible]
PHACTR4	SOX2-KLF5 coregulated genes from PMID: 33972779	SOX2 + KLF[illegible]
ZNF365	SOX2-KLF5 coregulated genes from PMID: 33972779	SOX2 + KLF[illegible]
ARFGAP3	SOX2-KLF5 coregulated genes from PMID: 33972779	SOX2 + KLF[illegible]
PACS1	SOX2-KLF5 coregulated genes from PMID: 33972779	SOX2 + KLF[illegible]
COL4A6	SOX2-KLF5 coregulated genes from PMID: 33972779	SOX2 + KLF[illegible]
IL20RB	SOX2-KLF5 coregulated genes from PMID: 33972779	SOX2 + KLF[illegible]
MREG	SOX2-KLF5 coregulated genes from PMID: 33972779	SOX2 + KLF[illegible]
TMEM200A	SOX2-KLF5 coregulated genes from PMID: 33972779	SOX2 + KLF[illegible]
PKD2	SOX2-KLF5 coregulated genes from PMID: 33972779	SOX2 + KLF[illegible]
SOCS2	SOX2-KLF5 coregulated genes from PMID: 33972779	SOX2 + KLF[illegible]
MYH9	SOX2-KLF5 coregulated genes from PMID: 33972779	SOX2 + KLF[illegible]
ETS1	SOX2-KLF5 coregulated genes from PMID: 33972779	SOX2 + KLF[illegible]
CDK14	SOX2-KLF5 coregulated genes from PMID: 33972779	SOX2 + KLF[illegible]
NFKBIA	SOX2-KLF5 coregulated genes from PMID: 33972779	SOX2 + KLF[illegible]
GUCY1A2	SOX2-KLF5 coregulated genes from PMID: 33972779	SOX2 + KLF[illegible]
DTNBP1	SOX2-KLF5 coregulated genes from PMID: 33972779	SOX2 + KLF[illegible]
MAL2	SOX2-KLF5 coregulated genes from PMID: 33972779	SOX2 + KLF[illegible]

PDZRN3	SOX2-KLF5 coregulated genes from PMID: 33972779	SOX2 + KLF5
MRAS	SOX2-KLF5 coregulated genes from PMID: 33972779	SOX2 + KLF5
B3GAT1	SOX2-KLF5 coregulated genes from PMID: 33972779	SOX2 + KLF5
ADD2	SOX2-KLF5 coregulated genes from PMID: 33972779	SOX2 + KLF5
SGMS1	SOX2-KLF5 coregulated genes from PMID: 33972779	SOX2 + KLF5
IL1A	SOX2-KLF5 coregulated genes from PMID: 33972779	SOX2 + KLF5
RANBP17	SOX2-KLF5 coregulated genes from PMID: 33972779	SOX2 + KLF5
NUDT4	SOX2-KLF5 coregulated genes from PMID: 33972779	SOX2 + KLF5
TXNDC12	SOX2-KLF5 coregulated genes from PMID: 33972779	SOX2 + KLF5
PEBP1	SOX2-KLF5 coregulated genes from PMID: 33972779	SOX2 + KLF5
TMC1	SOX2-KLF5 coregulated genes from PMID: 33972779	SOX2 + KLF5
RBMS3	SOX2-KLF5 coregulated genes from PMID: 33972779	SOX2 + KLF5
LIPT2	SOX2-KLF5 coregulated genes from PMID: 33972779	SOX2 + KLF5
CDH6	SOX2-KLF5 coregulated genes from PMID: 33972779	SOX2 + KLF5
FAM3C	SOX2-KLF5 coregulated genes from PMID: 33972779	SOX2 + KLF5
GHR	SOX2-KLF5 coregulated genes from PMID: 33972779	SOX2 + KLF5
ALDH3A1	SOX2-KLF5 coregulated genes from PMID: 33972779	SOX2 + KLF5
EYA1	SOX2-KLF5 coregulated genes from PMID: 33972779	SOX2 + KLF5
LAMB1	SOX2-KLF5 coregulated genes from PMID: 33972779	SOX2 + KLF5
CDH11	SOX2-KLF5 coregulated genes from PMID: 33972779	SOX2 + KLF5
ESYT3	SOX2-KLF5 coregulated genes from PMID: 33972779	SOX2 + KLF5
BAIAP2L1	SOX2-KLF5 coregulated genes from PMID: 33972779	SOX2 + KLF5
CCDC88C	SOX2-KLF5 coregulated genes from PMID: 33972779	SOX2 + KLF5
HMGCLL1	SOX2-KLF5 coregulated genes from PMID: 33972779	SOX2 + KLF5
CMYA5	SOX2-KLF5 coregulated genes from PMID: 33972779	SOX2 + KLF5
TOX3	SOX2-KLF5 coregulated genes from PMID: 33972779	SOX2 + KLF5
HS6ST1	SOX2-KLF5 coregulated genes from PMID: 33972779	SOX2 + KLF5
MMP16	SOX2-KLF5 coregulated genes from PMID: 33972779	SOX2 + KLF5
DMD	SOX2-KLF5 coregulated genes from PMID: 33972779	SOX2 + KLF5
ARID5B	SOX2-KLF5 coregulated genes from PMID: 33972779	SOX2 + KLF5
SYT17	SOX2-KLF5 coregulated genes from PMID: 33972779	SOX2 + KLF5
PI15	SOX2-KLF5 coregulated genes from PMID: 33972779	SOX2 + KLF5
EEA1	SOX2-KLF5 coregulated genes from PMID: 33972779	SOX2 + KLF5
PRKD1	SOX2-KLF5 coregulated genes from PMID: 33972779	SOX2 + KLF5
MCC	SOX2-KLF5 coregulated genes from PMID: 33972779	SOX2 + KLF5
TRIM2	SOX2-KLF5 coregulated genes from PMID: 33972779	SOX2 + KLF5
B4GALNT4	SOX2-KLF5 coregulated genes from PMID: 33972779	SOX2 + KLF5
MPPED2	SOX2-KLF5 coregulated genes from PMID: 33972779	SOX2 + KLF5
GIPC2	SOX2-KLF5 coregulated genes from PMID: 33972779	SOX2 + KLF5
TEK	SOX2-KLF5 coregulated genes from PMID: 33972779	SOX2 + KLF5
ROBO2	SOX2-KLF5 coregulated genes from PMID: 33972779	SOX2 + KLF5
TJP2	SOX2-KLF5 coregulated genes from PMID: 33972779	SOX2 + KLF5
KCNN2	SOX2-KLF5 coregulated genes from PMID: 33972779	SOX2 + KLF5
NEGR1	SOX2-KLF5 coregulated genes from PMID: 33972779	SOX2 + KLF5
GPHN	SOX2-KLF5 coregulated genes from PMID: 33972779	SOX2 + KLF5
RNF19A	SOX2-KLF5 coregulated genes from PMID: 33972779	SOX2 + KLF5
FAM181B	SOX2-KLF5 coregulated genes from PMID: 33972779	SOX2 + KLF5
TM4SF1	SOX2-KLF5 coregulated genes from PMID: 33972779	SOX2 + KLF5
UGCG	SOX2-KLF5 coregulated genes from PMID: 33972779	SOX2 + KLF5
ATP10B	SOX2-KLF5 coregulated genes from PMID: 33972779	SOX2 + KLF5
FKBP5	SOX2-KLF5 coregulated genes from PMID: 33972779	SOX2 + KLF5
TGM7	SOX2-KLF5 coregulated genes from PMID: 33972779	SOX2 + KLF5
PLA1A	SOX2-KLF5 coregulated genes from PMID: 33972779	SOX2 + KLF5
NHLRC2	SOX2-KLF5 coregulated genes from PMID: 33972779	SOX2 + KLF5
NDUFS4	SOX2-KLF5 coregulated genes from PMID: 33972779	SOX2 + KLF5
AGPAT4	SOX2-KLF5 coregulated genes from PMID: 33972779	SOX2 + KLF5
TULP4	SOX2-KLF5 coregulated genes from PMID: 33972779	SOX2 + KLF5
EHF	SOX2-KLF5 coregulated genes from PMID: 33972779	SOX2 + KLF5
SLC9A2	SOX2-KLF5 coregulated genes from PMID: 33972779	SOX2 + KLF5

TNFSF15	SOX2-KLF5 coregulated genes from PMID: 33972779	SOX2 + KLF
GRM8	SOX2-KLF5 coregulated genes from PMID: 33972779	SOX2 + KLF
NUDT7	SOX2-KLF5 coregulated genes from PMID: 33972779	SOX2 + KLF
PARD3	SOX2-KLF5 coregulated genes from PMID: 33972779	SOX2 + KLF
SPRY1	SOX2-KLF5 coregulated genes from PMID: 33972779	SOX2 + KLF
EPHA3	SOX2-KLF5 coregulated genes from PMID: 33972779	SOX2 + KLF
PLB1	SOX2-KLF5 coregulated genes from PMID: 33972779	SOX2 + KLF
ANKH	SOX2-KLF5 coregulated genes from PMID: 33972779	SOX2 + KLF
DNAJC6	SOX2-KLF5 coregulated genes from PMID: 33972779	SOX2 + KLF
VEGFC	SOX2-KLF5 coregulated genes from PMID: 33972779	SOX2 + KLF
QKI	SOX2-KLF5 coregulated genes from PMID: 33972779	SOX2 + KLF
ADM	SOX2-KLF5 coregulated genes from PMID: 33972779	SOX2 + KLF
PODXL	SOX2-KLF5 coregulated genes from PMID: 33972779	SOX2 + KLF
PPP4R4	SOX2-KLF5 coregulated genes from PMID: 33972779	SOX2 + KLF
EPCAM	SOX2-KLF5 coregulated genes from PMID: 33972779	SOX2 + KLF
PTER	SOX2-KLF5 coregulated genes from PMID: 33972779	SOX2 + KLF
CEP68	SOX2-KLF5 coregulated genes from PMID: 33972779	SOX2 + KLF
CRADD	SOX2-KLF5 coregulated genes from PMID: 33972779	SOX2 + KLF
PTGR1	SOX2-KLF5 coregulated genes from PMID: 33972779	SOX2 + KLF
EPGN	SOX2-KLF5 coregulated genes from PMID: 33972779	SOX2 + KLF
BTG1	SOX2-KLF5 coregulated genes from PMID: 33972779	SOX2 + KLF
ARMCX1	SOX2-KLF5 coregulated genes from PMID: 33972779	SOX2 + KLF
KLHL8	SOX2-KLF5 coregulated genes from PMID: 33972779	SOX2 + KLF
ALDH5A1	SOX2-KLF5 coregulated genes from PMID: 33972779	SOX2 + KLF
CAMK4	SOX2-KLF5 coregulated genes from PMID: 33972779	SOX2 + KLF
ARG1	SOX2-KLF5 coregulated genes from PMID: 33972779	SOX2 + KLF
NEURL1B	SOX2-KLF5 coregulated genes from PMID: 33972779	SOX2 + KLF
FAM110C	SOX2-KLF5 coregulated genes from PMID: 33972779	SOX2 + KLF
SLC25A25	SOX2-KLF5 coregulated genes from PMID: 33972779	SOX2 + KLF
ANKRD6	SOX2-KLF5 coregulated genes from PMID: 33972779	SOX2 + KLF
MAP3K8	SOX2-KLF5 coregulated genes from PMID: 33972779	SOX2 + KLF
ALCAM	SOX2-KLF5 coregulated genes from PMID: 33972779	SOX2 + KLF
JPH1	SOX2-KLF5 coregulated genes from PMID: 33972779	SOX2 + KLF
DNAJC21	SOX2-KLF5 coregulated genes from PMID: 33972779	SOX2 + KLF
SESTD1	SOX2-KLF5 coregulated genes from PMID: 33972779	SOX2 + KLF
SRL	SOX2-KLF5 coregulated genes from PMID: 33972779	SOX2 + KLF
ARHGEF12	SOX2-KLF5 coregulated genes from PMID: 33972779	SOX2 + KLF
SORBS2	SOX2-KLF5 coregulated genes from PMID: 33972779	SOX2 + KLF
EFEMP1	SOX2-KLF5 coregulated genes from PMID: 33972779	SOX2 + KLF
MBNL3	SOX2-KLF5 coregulated genes from PMID: 33972779	SOX2 + KLF
KHDRBS3	SOX2-KLF5 coregulated genes from PMID: 33972779	SOX2 + KLF
MFAP3L	SOX2-KLF5 coregulated genes from PMID: 33972779	SOX2 + KLF
MEGF9	SOX2-KLF5 coregulated genes from PMID: 33972779	SOX2 + KLF
GLIS3	SOX2-KLF5 coregulated genes from PMID: 33972779	SOX2 + KLF
PAIP2B	SOX2-KLF5 coregulated genes from PMID: 33972779	SOX2 + KLF
CX3CL1	SOX2-KLF5 coregulated genes from PMID: 33972779	SOX2 + KLF
PFN2	SOX2-KLF5 coregulated genes from PMID: 33972779	SOX2 + KLF
MBOAT2	SOX2-KLF5 coregulated genes from PMID: 33972779	SOX2 + KLF
TBC1D30	SOX2-KLF5 coregulated genes from PMID: 33972779	SOX2 + KLF
NUAK2	SOX2-KLF5 coregulated genes from PMID: 33972779	SOX2 + KLF
IL6R	SOX2-KLF5 coregulated genes from PMID: 33972779	SOX2 + KLF
B3GNT2	SOX2-KLF5 coregulated genes from PMID: 33972779	SOX2 + KLF
PBX1	SOX2-KLF5 coregulated genes from PMID: 33972779	SOX2 + KLF
TBCA	SOX2-KLF5 coregulated genes from PMID: 33972779	SOX2 + KLF
SETD5	SOX2-KLF5 coregulated genes from PMID: 33972779	SOX2 + KLF
KLHL29	SOX2-KLF5 coregulated genes from PMID: 33972779	SOX2 + KLF
DGKG	SOX2-KLF5 coregulated genes from PMID: 33972779	SOX2 + KLF
LITAF	SOX2-KLF5 coregulated genes from PMID: 33972779	SOX2 + KLF
ST14	SOX2-KLF5 coregulated genes from PMID: 33972779	SOX2 + KLF

RBPJ	SOX2-KLF5 coregulated genes from PMID: 33972779	SOX2 + KLF
ADAM12	SOX2-KLF5 coregulated genes from PMID: 33972779	SOX2 + KLF
SLC4A3	SOX2-KLF5 coregulated genes from PMID: 33972779	SOX2 + KLF
CCDC141	SOX2-KLF5 coregulated genes from PMID: 33972779	SOX2 + KLF
IDH2	SOX2-KLF5 coregulated genes from PMID: 33972779	SOX2 + KLF
TNRC18	SOX2-KLF5 coregulated genes from PMID: 33972779	SOX2 + KLF
CDH13	SOX2-KLF5 coregulated genes from PMID: 33972779	SOX2 + KLF
MPZL1	SOX2-KLF5 coregulated genes from PMID: 33972779	SOX2 + KLF
TXNRD1	SOX2-KLF5 coregulated genes from PMID: 33972779	SOX2 + KLF
TANC2	SOX2-KLF5 coregulated genes from PMID: 33972779	SOX2 + KLF
RGS4	SOX2-KLF5 coregulated genes from PMID: 33972779	SOX2 + KLF
IL10	SOX2-KLF5 coregulated genes from PMID: 33972779	SOX2 + KLF
KRT19	SOX2-KLF5 coregulated genes from PMID: 33972779	SOX2 + KLF
ARHGAP18	SOX2-KLF5 coregulated genes from PMID: 33972779	SOX2 + KLF
MEGF11	SOX2-KLF5 coregulated genes from PMID: 33972779	SOX2 + KLF
KIRREL1	SOX2-KLF5 coregulated genes from PMID: 33972779	SOX2 + KLF
SLC13A2	SOX2-KLF5 coregulated genes from PMID: 33972779	SOX2 + KLF
ALOX12	SOX2-KLF5 coregulated genes from PMID: 33972779	SOX2 + KLF
SHISA6	SOX2-KLF5 coregulated genes from PMID: 33972779	SOX2 + KLF
SMOC2	SOX2-KLF5 coregulated genes from PMID: 33972779	SOX2 + KLF
NRXN1	SOX2-KLF5 coregulated genes from PMID: 33972779	SOX2 + KLF
RGL1	SOX2-KLF5 coregulated genes from PMID: 33972779	SOX2 + KLF
SOX6	SOX2-KLF5 coregulated genes from PMID: 33972779	SOX2 + KLF
CTNND2	SOX2-KLF5 coregulated genes from PMID: 33972779	SOX2 + KLF
BLNK	SOX2-KLF5 coregulated genes from PMID: 33972779	SOX2 + KLF
RARRES1	SOX2-KLF5 coregulated genes from PMID: 33972779	SOX2 + KLF
NMNAT2	SOX2-KLF5 coregulated genes from PMID: 33972779	SOX2 + KLF
PPL	SOX2-KLF5 coregulated genes from PMID: 33972779	SOX2 + KLF
CADPS2	SOX2-KLF5 coregulated genes from PMID: 33972779	SOX2 + KLF
EFNA5	SOX2-KLF5 coregulated genes from PMID: 33972779	SOX2 + KLF
UTRN	SOX2-KLF5 coregulated genes from PMID: 33972779	SOX2 + KLF
PKP1	SOX2-KLF5 coregulated genes from PMID: 33972779	SOX2 + KLF
KCNJ2	SOX2-KLF5 coregulated genes from PMID: 33972779	SOX2 + KLF
NUDT4B	SOX2-KLF5 coregulated genes from PMID: 33972779	SOX2 + KLF
ATP1B1	SOX2-KLF5 coregulated genes from PMID: 33972779	SOX2 + KLF
FAM43A	SOX2-KLF5 coregulated genes from PMID: 33972779	SOX2 + KLF
ADRB1	SOX2-KLF5 coregulated genes from PMID: 33972779	SOX2 + KLF
LYPD6B	SOX2-KLF5 coregulated genes from PMID: 33972779	SOX2 + KLF
TNK2	SOX2-KLF5 coregulated genes from PMID: 33972779	SOX2 + KLF
LY96	SOX2-KLF5 coregulated genes from PMID: 33972779	SOX2 + KLF
OTOP1	SOX2-KLF5 coregulated genes from PMID: 33972779	SOX2 + KLF
HDAC9	SOX2-KLF5 coregulated genes from PMID: 33972779	SOX2 + KLF
IQGAP1	SOX2-KLF5 coregulated genes from PMID: 33972779	SOX2 + KLF
PLD1	SOX2-KLF5 coregulated genes from PMID: 33972779	SOX2 + KLF
SMPDL3B	SOX2-KLF5 coregulated genes from PMID: 33972779	SOX2 + KLF
KCNK1	SOX2-KLF5 coregulated genes from PMID: 33972779	SOX2 + KLF
IRAK2	SOX2-KLF5 coregulated genes from PMID: 33972779	SOX2 + KLF
TMEM161B	SOX2-KLF5 coregulated genes from PMID: 33972779	SOX2 + KLF
RC3H1	SOX2-KLF5 coregulated genes from PMID: 33972779	SOX2 + KLF
HHAT	SOX2-KLF5 coregulated genes from PMID: 33972779	SOX2 + KLF
IRS1	SOX2-KLF5 coregulated genes from PMID: 33972779	SOX2 + KLF
CNIH4	SOX2-KLF5 coregulated genes from PMID: 33972779	SOX2 + KLF
ITGA6	SOX2-KLF5 coregulated genes from PMID: 33972779	SOX2 + KLF
RAB43	SOX2-KLF5 coregulated genes from PMID: 33972779	SOX2 + KLF
EPHA7	SOX2-KLF5 coregulated genes from PMID: 33972779	SOX2 + KLF
CFTR	SOX2-KLF5 coregulated genes from PMID: 33972779	SOX2 + KLF
ADAM9	SOX2-KLF5 coregulated genes from PMID: 33972779	SOX2 + KLF
SPRY4	SOX2-KLF5 coregulated genes from PMID: 33972779	SOX2 + KLF
ST3GAL3	SOX2-KLF5 coregulated genes from PMID: 33972779	SOX2 + KLF

BTG2	SOX2-KLF5 coregulated genes from PMID: 33972779	SOX2 + KLF
NFKBIZ	SOX2-KLF5 coregulated genes from PMID: 33972779	SOX2 + KLF
PGBD1	SOX2-KLF5 coregulated genes from PMID: 33972779	SOX2 + KLF
LPCAT4	SOX2-KLF5 coregulated genes from PMID: 33972779	SOX2 + KLF
RFFL	SOX2-KLF5 coregulated genes from PMID: 33972779	SOX2 + KLF
NOS1AP	SOX2-KLF5 coregulated genes from PMID: 33972779	SOX2 + KLF
CLSTN2	SOX2-KLF5 coregulated genes from PMID: 33972779	SOX2 + KLF
CLDN4	SOX2-KLF5 coregulated genes from PMID: 33972779	SOX2 + KLF
SLC25A24	SOX2-KLF5 coregulated genes from PMID: 33972779	SOX2 + KLF
GGH	SOX2-KLF5 coregulated genes from PMID: 33972779	SOX2 + KLF
ROCK2	SOX2-KLF5 coregulated genes from PMID: 33972779	SOX2 + KLF
ARHGAP20	SOX2-KLF5 coregulated genes from PMID: 33972779	SOX2 + KLF
DNM3	SOX2-KLF5 coregulated genes from PMID: 33972779	SOX2 + KLF
GRIP1	SOX2-KLF5 coregulated genes from PMID: 33972779	SOX2 + KLF
CHMP2B	SOX2-KLF5 coregulated genes from PMID: 33972779	SOX2 + KLF
CAPN2	SOX2-KLF5 coregulated genes from PMID: 33972779	SOX2 + KLF
ELF3	SOX2-KLF5 coregulated genes from PMID: 33972779	SOX2 + KLF
BMPR2	SOX2-KLF5 coregulated genes from PMID: 33972779	SOX2 + KLF
EIF4G3	SOX2-KLF5 coregulated genes from PMID: 33972779	SOX2 + KLF
DSP	SOX2-KLF5 coregulated genes from PMID: 33972779	SOX2 + KLF
ERC1	SOX2-KLF5 coregulated genes from PMID: 33972779	SOX2 + KLF
QSOX1	SOX2-KLF5 coregulated genes from PMID: 33972779	SOX2 + KLF
KRT20	SOX2-KLF5 coregulated genes from PMID: 33972779	SOX2 + KLF
ACOX1	SOX2-KLF5 coregulated genes from PMID: 33972779	SOX2 + KLF
RASGRP1	SOX2-KLF5 coregulated genes from PMID: 33972779	SOX2 + KLF
HMCN1	SOX2-KLF5 coregulated genes from PMID: 33972779	SOX2 + KLF
TNFAIP3	SOX2-KLF5 coregulated genes from PMID: 33972779	SOX2 + KLF
NGEF	SOX2-KLF5 coregulated genes from PMID: 33972779	SOX2 + KLF
ATP10A	SOX2-KLF5 coregulated genes from PMID: 33972779	SOX2 + KLF
LMNA	SOX2-KLF5 coregulated genes from PMID: 33972779	SOX2 + KLF
C3orf52	SOX2-KLF5 coregulated genes from PMID: 33972779	SOX2 + KLF
DST	SOX2-KLF5 coregulated genes from PMID: 33972779	SOX2 + KLF
SGMS2	SOX2-KLF5 coregulated genes from PMID: 33972779	SOX2 + KLF
FAM189B	SOX2-KLF5 coregulated genes from PMID: 33972779	SOX2 + KLF
EZR	SOX2-KLF5 coregulated genes from PMID: 33972779	SOX2 + KLF
CASQ2	SOX2-KLF5 coregulated genes from PMID: 33972779	SOX2 + KLF
CSGALNACT1	SOX2-KLF5 coregulated genes from PMID: 33972779	SOX2 + KLF
ZNF362	SOX2-KLF5 coregulated genes from PMID: 33972779	SOX2 + KLF
ACAD9	SOX2-KLF5 coregulated genes from PMID: 33972779	SOX2 + KLF
CDC42EP3	SOX2-KLF5 coregulated genes from PMID: 33972779	SOX2 + KLF
LIMA1	SOX2-KLF5 coregulated genes from PMID: 33972779	SOX2 + KLF
NR4A1	SOX2-KLF5 coregulated genes from PMID: 33972779	SOX2 + KLF
NRP2	SOX2-KLF5 coregulated genes from PMID: 33972779	SOX2 + KLF
ODC1	SOX2-KLF5 coregulated genes from PMID: 33972779	SOX2 + KLF
ACVR1	SOX2-KLF5 coregulated genes from PMID: 33972779	SOX2 + KLF
FAM126A	SOX2-KLF5 coregulated genes from PMID: 33972779	SOX2 + KLF
IFIH1	SOX2-KLF5 coregulated genes from PMID: 33972779	SOX2 + KLF
SYTL3	SOX2-KLF5 coregulated genes from PMID: 33972779	SOX2 + KLF
SMG7	SOX2-KLF5 coregulated genes from PMID: 33972779	SOX2 + KLF
SGK1	SOX2-KLF5 coregulated genes from PMID: 33972779	SOX2 + KLF
IL18RAP	SOX2-KLF5 coregulated genes from PMID: 33972779	SOX2 + KLF
LFNG	SOX2-KLF5 coregulated genes from PMID: 33972779	SOX2 + KLF
SLC4A7	SOX2-KLF5 coregulated genes from PMID: 33972779	SOX2 + KLF
C3orf70	SOX2-KLF5 coregulated genes from PMID: 33972779	SOX2 + KLF
LAMC2	SOX2-KLF5 coregulated genes from PMID: 33972779	SOX2 + KLF
EIF1	SOX2-KLF5 coregulated genes from PMID: 33972779	SOX2 + KLF
SFXN3	SOX2-KLF5 coregulated genes from PMID: 33972779	SOX2 + KLF
MBNL1	SOX2-KLF5 coregulated genes from PMID: 33972779	SOX2 + KLF
NHSL1	SOX2-KLF5 coregulated genes from PMID: 33972779	SOX2 + KLF

KIT	SOX2-KLF5 coregulated genes from PMID: 33972779	SOX2 + KLF[illegible]
GBP2	SOX2-KLF5 coregulated genes from PMID: 33972779	SOX2 + KLF[illegible]
PLD5	SOX2-KLF5 coregulated genes from PMID: 33972779	SOX2 + KLF[illegible]
STC2	SOX2-KLF5 coregulated genes from PMID: 33972779	SOX2 + KLF[illegible]
RGS5	SOX2-KLF5 coregulated genes from PMID: 33972779	SOX2 + KLF[illegible]
ASPA	SOX2-KLF5 coregulated genes from PMID: 33972779	SOX2 + KLF[illegible]
GBP5	SOX2-KLF5 coregulated genes from PMID: 33972779	SOX2 + KLF[illegible]
RPRM	SOX2-KLF5 coregulated genes from PMID: 33972779	SOX2 + KLF[illegible]
FOXP1	SOX2-KLF5 coregulated genes from PMID: 33972779	SOX2 + KLF[illegible]
AMIGO1	SOX2-KLF5 coregulated genes from PMID: 33972779	SOX2 + KLF[illegible]
PDE4DIP	SOX2-KLF5 coregulated genes from PMID: 33972779	SOX2 + KLF[illegible]
SNRNP48	SOX2-KLF5 coregulated genes from PMID: 33972779	SOX2 + KLF[illegible]
PXDN	SOX2-KLF5 coregulated genes from PMID: 33972779	SOX2 + KLF[illegible]
SULT2B1	SOX2-KLF5 coregulated genes from PMID: 33972779	SOX2 + KLF[illegible]
CRYZ	SOX2-KLF5 coregulated genes from PMID: 33972779	SOX2 + KLF[illegible]
THBS2	SOX2-KLF5 coregulated genes from PMID: 33972779	SOX2 + KLF[illegible]
STAC	SOX2-KLF5 coregulated genes from PMID: 33972779	SOX2 + KLF[illegible]
SLC6A6	SOX2-KLF5 coregulated genes from PMID: 33972779	SOX2 + KLF[illegible]
SPPL3	SOX2-KLF5 coregulated genes from PMID: 33972779	SOX2 + KLF[illegible]
LPGAT1	SOX2-KLF5 coregulated genes from PMID: 33972779	SOX2 + KLF[illegible]
CLDN1	SOX2-KLF5 coregulated genes from PMID: 33972779	SOX2 + KLF[illegible]
PDLIM4	SOX2-KLF5 coregulated genes from PMID: 33972779	SOX2 + KLF[illegible]
EPN2	SOX2-KLF5 coregulated genes from PMID: 33972779	SOX2 + KLF[illegible]
JUN	SOX2-KLF5 coregulated genes from PMID: 33972779	SOX2 + KLF[illegible]
TNIP1	SOX2-KLF5 coregulated genes from PMID: 33972779	SOX2 + KLF[illegible]
S1PR1	SOX2-KLF5 coregulated genes from PMID: 33972779	SOX2 + KLF[illegible]
CD9	SOX2-KLF5 coregulated genes from PMID: 33972779	SOX2 + KLF[illegible]
ANGPT1	SOX2-KLF5 coregulated genes from PMID: 33972779	SOX2 + KLF[illegible]
SLC6A2	SOX2-KLF5 coregulated genes from PMID: 33972779	SOX2 + KLF[illegible]
RBFOX1	SOX2-KLF5 coregulated genes from PMID: 33972779	SOX2 + KLF[illegible]
DGKI	SOX2-KLF5 coregulated genes from PMID: 33972779	SOX2 + KLF[illegible]
ATXN1	SOX2-KLF5 coregulated genes from PMID: 33972779	SOX2 + KLF[illegible]
SOX13	SOX2-KLF5 coregulated genes from PMID: 33972779	SOX2 + KLF[illegible]
VCAM1	SOX2-KLF5 coregulated genes from PMID: 33972779	SOX2 + KLF[illegible]
TFCP2L1	SOX2-KLF5 coregulated genes from PMID: 33972779	SOX2 + KLF[illegible]
LRRC8B	SOX2-KLF5 coregulated genes from PMID: 33972779	SOX2 + KLF[illegible]
MACF1	SOX2-KLF5 coregulated genes from PMID: 33972779	SOX2 + KLF[illegible]
TP63	SOX2-KLF5 coregulated genes from PMID: 33972779	SOX2 + KLF[illegible]
DUSP1	SOX2-KLF5 coregulated genes from PMID: 33972779	SOX2 + KLF[illegible]
SLC16A11	SOX2-KLF5 coregulated genes from PMID: 33972779	SOX2 + KLF[illegible]
PLS1	SOX2-KLF5 coregulated genes from PMID: 33972779	SOX2 + KLF[illegible]
ABCB11	SOX2-KLF5 coregulated genes from PMID: 33972779	SOX2 + KLF[illegible]
HS3ST3A1	SOX2-KLF5 coregulated genes from PMID: 33972779	SOX2 + KLF[illegible]
ANGPTL4	SOX2-KLF5 coregulated genes from PMID: 33972779	SOX2 + KLF[illegible]
HRH1	SOX2-KLF5 coregulated genes from PMID: 33972779	SOX2 + KLF[illegible]
GNB4	SOX2-KLF5 coregulated genes from PMID: 33972779	SOX2 + KLF[illegible]
CNN3	SOX2-KLF5 coregulated genes from PMID: 33972779	SOX2 + KLF[illegible]
CREG1	SOX2-KLF5 coregulated genes from PMID: 33972779	SOX2 + KLF[illegible]
RNF19B	SOX2-KLF5 coregulated genes from PMID: 33972779	SOX2 + KLF[illegible]
PDE4D	SOX2-KLF5 coregulated genes from PMID: 33972779	SOX2 + KLF[illegible]
MDFIC	SOX2-KLF5 coregulated genes from PMID: 33972779	SOX2 + KLF[illegible]
BCL2L15	SOX2-KLF5 coregulated genes from PMID: 33972779	SOX2 + KLF[illegible]
TRIO	SOX2-KLF5 coregulated genes from PMID: 33972779	SOX2 + KLF[illegible]
LPAR3	SOX2-KLF5 coregulated genes from PMID: 33972779	SOX2 + KLF[illegible]
GOLIM4	SOX2-KLF5 coregulated genes from PMID: 33972779	SOX2 + KLF[illegible]
MYH14	SOX2-KLF5 coregulated genes from PMID: 33972779	SOX2 + KLF[illegible]
VIM	SOX2-KLF5 coregulated genes from PMID: 33972779	SOX2 + KLF[illegible]
COL4A3	SOX2-KLF5 coregulated genes from PMID: 33972779	SOX2 + KLF[illegible]
PTPRJ	SOX2-KLF5 coregulated genes from PMID: 33972779	SOX2 + KLF[illegible]

NWD1	SOX2-KLF5 coregulated genes from PMID: 33972779	SOX2 + KLF5
MNDA	SOX2-KLF5 coregulated genes from PMID: 33972779	SOX2 + KLF5
SLC25A10	SOX2-KLF5 coregulated genes from PMID: 33972779	SOX2 + KLF5
PRMT6	SOX2-KLF5 coregulated genes from PMID: 33972779	SOX2 + KLF5
PTGS2	SOX2-KLF5 coregulated genes from PMID: 33972779	SOX2 + KLF5
CCL20	SOX2-KLF5 coregulated genes from PMID: 33972779	SOX2 + KLF5
RARB	SOX2-KLF5 coregulated genes from PMID: 33972779	SOX2 + KLF5
TWF2	SOX2-KLF5 coregulated genes from PMID: 33972779	SOX2 + KLF5
DHRS9	SOX2-KLF5 coregulated genes from PMID: 33972779	SOX2 + KLF5
SH3PXD2A	SOX2-KLF5 coregulated genes from PMID: 33972779	SOX2 + KLF5
PLEKHM3	SOX2-KLF5 coregulated genes from PMID: 33972779	SOX2 + KLF5
AFF1	SOX2-KLF5 coregulated genes from PMID: 33972779	SOX2 + KLF5
TRIM33	SOX2-KLF5 coregulated genes from PMID: 33972779	SOX2 + KLF5
LRIG1	SOX2-KLF5 coregulated genes from PMID: 33972779	SOX2 + KLF5
SIPA1L2	SOX2-KLF5 coregulated genes from PMID: 33972779	SOX2 + KLF5
ABR	SOX2-KLF5 coregulated genes from PMID: 33972779	SOX2 + KLF5
NUAK1	SOX2-KLF5 coregulated genes from PMID: 33972779	SOX2 + KLF5
RAP2B	SOX2-KLF5 coregulated genes from PMID: 33972779	SOX2 + KLF5
KRT8	SOX2-KLF5 coregulated genes from PMID: 33972779	SOX2 + KLF5
SEMA6A	SOX2-KLF5 coregulated genes from PMID: 33972779	SOX2 + KLF5
SBF2	SOX2-KLF5 coregulated genes from PMID: 33972779	SOX2 + KLF5
LRRC8C	SOX2-KLF5 coregulated genes from PMID: 33972779	SOX2 + KLF5
IQGAP2	SOX2-KLF5 coregulated genes from PMID: 33972779	SOX2 + KLF5
BMP2	SOX2 regulated genes from PMID: 20726797	SOX2
BMP4	SOX2 regulated genes from PMID: 20726797	SOX2
BMPR2	SOX2 regulated genes from PMID: 20726797	SOX2
PCSK6	SOX2 regulated genes from PMID: 20726797	SOX2
SMAD5	SOX2 regulated genes from PMID: 20726797	SOX2
SMAD6	SOX2 regulated genes from PMID: 20726797	SOX2
SMAD7	SOX2 regulated genes from PMID: 20726797	SOX2
SMURF1	SOX2 regulated genes from PMID: 20726797	SOX2
IGF1R	SOX2 regulated genes from PMID: 20726797	SOX2
GNA15	SOX2 regulated genes from PMID: 20726797	SOX2
TGFB2	SOX2 regulated genes from PMID: 20726797	SOX2
RGS3	SOX2 regulated genes from PMID: 20726797	SOX2
FGFR3	SOX2 regulated genes from PMID: 20726797	SOX2
IL6ST	SOX2 regulated genes from PMID: 20726797	SOX2
EREG	SOX2 regulated genes from PMID: 20726797	SOX2
SOCS5	SOX2 regulated genes from PMID: 20726797	SOX2
IRAK1	SOX2 regulated genes from PMID: 20726797	SOX2
PIK3R1	SOX2 regulated genes from PMID: 20726797	SOX2
EDN1	SOX2 regulated genes from PMID: 20726797	SOX2
OPN3	SOX2 regulated genes from PMID: 20726797	SOX2
PYCARD	SOX2 regulated genes from PMID: 20726797	SOX2
SIGIRR	SOX2 regulated genes from PMID: 20726797	SOX2
CD24	SOX2 regulated genes from PMID: 20726797	SOX2
AGRN	SOX2 regulated genes from PMID: 20726797	SOX2
RHOQ	SOX2 regulated genes from PMID: 20726797	SOX2
IRS1	SOX2 regulated genes from PMID: 20726797	SOX2
MAML3	SOX2 regulated genes from PMID: 20726797	SOX2
GNB2	SOX2 regulated genes from PMID: 20726797	SOX2
COL16A1	SOX2 regulated genes from PMID: 20726797	SOX2
MTSS1	SOX2 regulated genes from PMID: 20726797	SOX2
TRIB1	SOX2 regulated genes from PMID: 20726797	SOX2
PTK2	SOX2 regulated genes from PMID: 20726797	SOX2
IFITM1	SOX2 regulated genes from PMID: 20726797	SOX2
ACVR1	SOX2 regulated genes from PMID: 20726797	SOX2
CCRL2	SOX2 regulated genes from PMID: 20726797	SOX2
TACSTD2	SOX2 regulated genes from PMID: 20726797	SOX2

PTPRF	SOX2 regulated genes from PMID: 20726797	SOX2
SMAD3	SOX2 regulated genes from PMID: 20726797	SOX2
DGKQ	SOX2 regulated genes from PMID: 20726797	SOX2
SRC	SOX2 regulated genes from PMID: 20726797	SOX2
EGFR	SOX2 regulated genes from PMID: 20726797	SOX2
DGKH	SOX2 regulated genes from PMID: 20726797	SOX2
KLK6	SOX2 regulated genes from PMID: 20726797	SOX2
GAB1	SOX2 regulated genes from PMID: 20726797	SOX2
VIPR1	SOX2 regulated genes from PMID: 20726797	SOX2
PARD3	SOX2 regulated genes from PMID: 20726797	SOX2
CD14	SOX2 regulated genes from PMID: 20726797	SOX2
ROR1	SOX2 regulated genes from PMID: 20726797	SOX2
ADAM17	SOX2 regulated genes from PMID: 20726797	SOX2
ADAM10	SOX2 regulated genes from PMID: 20726797	SOX2
NUP62	SOX2 regulated genes from PMID: 20726797	SOX2
LEPR	SOX2 regulated genes from PMID: 20726797	SOX2
P2RY2	SOX2 regulated genes from PMID: 20726797	SOX2
AFAP1L2	SOX2 regulated genes from PMID: 20726797	SOX2
AREG	SOX2 regulated genes from PMID: 20726797	SOX2
HPGD	SOX2 regulated genes from PMID: 20726797	SOX2
STAT3	SOX2 regulated genes from PMID: 20726797	SOX2
CXCL1	SOX2 regulated genes from PMID: 20726797	SOX2
GPR37	SOX2 regulated genes from PMID: 20726797	SOX2
CD59	SOX2 regulated genes from PMID: 20726797	SOX2
LY6E	SOX2 regulated genes from PMID: 20726797	SOX2
CBLC	SOX2 regulated genes from PMID: 20726797	SOX2
PSENEN	SOX2 regulated genes from PMID: 20726797	SOX2
THBS1	SOX2 regulated genes from PMID: 20726797	SOX2
DEFB1	SOX2 regulated genes from PMID: 20726797	SOX2
ADRA2A	SOX2 regulated genes from PMID: 20726797	SOX2
RGS2	SOX2 regulated genes from PMID: 20726797	SOX2
ITPR3	SOX2 regulated genes from PMID: 20726797	SOX2
RBP4	SOX2 regulated genes from PMID: 20726797	SOX2
HSD17B2	SOX2 regulated genes from PMID: 20726797	SOX2
RXRA	SOX2 regulated genes from PMID: 20726797	SOX2
AQP3	SOX2 regulated genes from PMID: 20726797	SOX2
RARA	SOX2 regulated genes from PMID: 20726797	SOX2
TRIM16	SOX2 regulated genes from PMID: 20726797	SOX2
ELF1	SOX2 regulated genes in Wounding from PMID: 30772301	SOX2
FLRT3	SOX2 regulated genes in Wounding from PMID: 30772301	SOX2
SUV39H2	SOX2 regulated genes in Wounding from PMID: 30772301	SOX2
PSTPIP2	SOX2 regulated genes in Wounding from PMID: 30772301	SOX2
TTC39B	SOX2 regulated genes in Wounding from PMID: 30772301	SOX2
EXOC5	SOX2 regulated genes in Wounding from PMID: 30772301	SOX2
SLITRK4	SOX2 regulated genes in Wounding from PMID: 30772301	SOX2
TFAP2C	SOX2 regulated genes in Wounding from PMID: 30772301	SOX2
AHCY	SOX2 regulated genes in Wounding from PMID: 30772301	SOX2
OSBP2	SOX2 regulated genes in Wounding from PMID: 30772301	SOX2
IL13RA1	SOX2 regulated genes in Wounding from PMID: 30772301	SOX2
ADGRA3	SOX2 regulated genes in Wounding from PMID: 30772301	SOX2
PBDC1	SOX2 regulated genes in Wounding from PMID: 30772301	SOX2
SMOX	SOX2 regulated genes in Wounding from PMID: 30772301	SOX2
TCEAL5	SOX2 regulated genes in Wounding from PMID: 30772301	SOX2
FBXO7	SOX2 regulated genes in Wounding from PMID: 30772301	SOX2
SLC39A6	SOX2 regulated genes in Wounding from PMID: 30772301	SOX2
YES1	SOX2 regulated genes in Wounding from PMID: 30772301	SOX2
RGS6	SOX2 regulated genes in Wounding from PMID: 30772301	SOX2
TMEM50B	SOX2 regulated genes in Wounding from PMID: 30772301	SOX2
GAREM1	SOX2 regulated genes in Wounding from PMID: 30772301	SOX2

SLC37A1	SOX2 regulated genes in Wounding from PMID: 30772301	SOX2
FAM3B	SOX2 regulated genes in Wounding from PMID: 30772301	SOX2
CCT8	SOX2 regulated genes in Wounding from PMID: 30772301	SOX2
ASCC2	SOX2 regulated genes in Wounding from PMID: 30772301	SOX2
TPTEP2-CSNK1E	SOX2 regulated genes in Wounding from PMID: 30772301	SOX2
CSNK1E	SOX2 regulated genes in Wounding from PMID: 30772301	SOX2
KLF6	SOX2 regulated genes in Wounding from PMID: 30772301	SOX2
BTG3	SOX2 regulated genes in Wounding from PMID: 30772301	SOX2
LURAP1L	SOX2 regulated genes in Wounding from PMID: 30772301	SOX2
DSG3	SOX2 regulated genes in Wounding from PMID: 30772301	SOX2
MAFF	SOX2 regulated genes in Wounding from PMID: 30772301	SOX2
HSPA13	SOX2 regulated genes in Wounding from PMID: 30772301	SOX2
WDR1	SOX2 regulated genes in Wounding from PMID: 30772301	SOX2
PSPC1	SOX2 regulated genes in Wounding from PMID: 30772301	SOX2
RAE1	SOX2 regulated genes in Wounding from PMID: 30772301	SOX2
EIF2S2	SOX2 regulated genes in Wounding from PMID: 30772301	SOX2
GTPBP6	SOX2 regulated genes in Wounding from PMID: 30772301	SOX2
PRELID3B	SOX2 regulated genes in Wounding from PMID: 30772301	SOX2
UNC79	SOX2 regulated genes in Wounding from PMID: 30772301	SOX2
ZBTB7C	SOX2 regulated genes in Wounding from PMID: 30772301	SOX2
LTBP2	SOX2 regulated genes in Wounding from PMID: 30772301	SOX2
DEPDC5	SOX2 regulated genes in Wounding from PMID: 30772301	SOX2
TMEM30B	SOX2 regulated genes in Wounding from PMID: 30772301	SOX2
PCSK2	SOX2 regulated genes in Wounding from PMID: 30772301	SOX2
NUDT11	SOX2 regulated genes in Wounding from PMID: 30772301	SOX2
BRWD1	SOX2 regulated genes in Wounding from PMID: 30772301	SOX2
BTBD7	SOX2 regulated genes in Wounding from PMID: 30772301	SOX2
DSTN	SOX2 regulated genes in Wounding from PMID: 30772301	SOX2
SAMD10	SOX2 regulated genes in Wounding from PMID: 30772301	SOX2
XKRX	SOX2 regulated genes in Wounding from PMID: 30772301	SOX2
MTCL1	SOX2 regulated genes in Wounding from PMID: 30772301	SOX2
NAA30	SOX2 regulated genes in Wounding from PMID: 30772301	SOX2
SCEL	SOX2 regulated genes in Wounding from PMID: 30772301	SOX2
RRP1	SOX2 regulated genes in Wounding from PMID: 30772301	SOX2
CCDC85C	SOX2 regulated genes in Wounding from PMID: 30772301	SOX2
RNF152	SOX2 regulated genes in Wounding from PMID: 30772301	SOX2
CLDN14	SOX2 regulated genes in Wounding from PMID: 30772301	SOX2
PLEKHD1	SOX2 regulated genes in Wounding from PMID: 30772301	SOX2
OSM	SOX2 regulated genes in Wounding from PMID: 30772301	SOX2
RNF17	SOX2 regulated genes in Wounding from PMID: 30772301	SOX2
TMEM33	SOX2 regulated genes in Wounding from PMID: 30772301	SOX2
TRMT5	SOX2 regulated genes in Wounding from PMID: 30772301	SOX2
BMP2	SOX2 regulated genes in Wounding from PMID: 30772301	SOX2
SRSF5	SOX2 regulated genes in Wounding from PMID: 30772301	SOX2
LHFPL1	SOX2 regulated genes in Wounding from PMID: 30772301	SOX2
CDKN2B	SOX2 regulated genes in Wounding from PMID: 30772301	SOX2
TXNL1	SOX2 regulated genes in Wounding from PMID: 30772301	SOX2
DHX15	SOX2 regulated genes in Wounding from PMID: 30772301	SOX2
POF1B	SOX2 regulated genes in Wounding from PMID: 30772301	SOX2
NDP	SOX2 regulated genes in Wounding from PMID: 30772301	SOX2
MTAP	SOX2 regulated genes in Wounding from PMID: 30772301	SOX2
RIPK4	SOX2 regulated genes in Wounding from PMID: 30772301	SOX2
EFNB1	SOX2 regulated genes in Wounding from PMID: 30772301	SOX2
ACAA2	SOX2 regulated genes in Wounding from PMID: 30772301	SOX2
PDE9A	SOX2 regulated genes in Wounding from PMID: 30772301	SOX2
LIMK2	SOX2 regulated genes in Wounding from PMID: 30772301	SOX2
CTIF	SOX2 regulated genes in Wounding from PMID: 30772301	SOX2
SYNGR1	SOX2 regulated genes in Wounding from PMID: 30772301	SOX2
PMEPA1	SOX2 regulated genes in Wounding from PMID: 30772301	SOX2

HSPH1	SOX2 regulated genes in Wounding from PMID: 30772301	SOX2
USP16	SOX2 regulated genes in Wounding from PMID: 30772301	SOX2
C21orf91	SOX2 regulated genes in Wounding from PMID: 30772301	SOX2
RAB27B	SOX2 regulated genes in Wounding from PMID: 30772301	SOX2
CORIN	SOX2 regulated genes in Wounding from PMID: 30772301	SOX2
EIF4ENIF1	SOX2 regulated genes in Wounding from PMID: 30772301	SOX2
TGIF1	SOX2 regulated genes in Wounding from PMID: 30772301	SOX2
MYO5B	SOX2 regulated genes in Wounding from PMID: 30772301	SOX2
SLAIN1	SOX2 regulated genes in Wounding from PMID: 30772301	SOX2
MSX1	SOX2 regulated genes in Wounding from PMID: 30772301	SOX2
GTF2F2	SOX2 regulated genes in Wounding from PMID: 30772301	SOX2
JAG1	SOX2 regulated genes in Wounding from PMID: 30772301	SOX2
SLC25A17	SOX2 regulated genes in Wounding from PMID: 30772301	SOX2
CDKL5	SOX2 regulated genes in Wounding from PMID: 30772301	SOX2
BRWD3	SOX2 regulated genes in Wounding from PMID: 30772301	SOX2
SMAD2	SOX2 regulated genes in Wounding from PMID: 30772301	SOX2
TEC	SOX2 regulated genes in Wounding from PMID: 30772301	SOX2
HOPX	SOX2 regulated genes in Wounding from PMID: 30772301	SOX2
SDC4	SOX2 regulated genes in Wounding from PMID: 30772301	SOX2
ATRN	SOX2 regulated genes in Wounding from PMID: 30772301	SOX2
COL18A1	SOX2 regulated genes in Wounding from PMID: 30772301	SOX2
PLA2G3	SOX2 regulated genes in Wounding from PMID: 30772301	SOX2
ETS2	SOX2 regulated genes in Wounding from PMID: 30772301	SOX2
TBC1D25	SOX2 regulated genes in Wounding from PMID: 30772301	SOX2
DSG4	SOX2 regulated genes in Wounding from PMID: 30772301	SOX2
CCDC68	SOX2 regulated genes in Wounding from PMID: 30772301	SOX2
RUNX1	SOX2 regulated genes in Wounding from PMID: 30772301	SOX2
S100G	SOX2 regulated genes in Wounding from PMID: 30772301	SOX2
EYA2	SOX2 regulated genes in Wounding from PMID: 30772301	SOX2
CPLX1	SOX2 regulated genes in Wounding from PMID: 30772301	SOX2
JADE3	SOX2 regulated genes in Wounding from PMID: 30772301	SOX2
RNF4	SOX2 regulated genes in Wounding from PMID: 30772301	SOX2
MORC4	SOX2 regulated genes in Wounding from PMID: 30772301	SOX2
SYCP2	SOX2 regulated genes in Wounding from PMID: 30772301	SOX2
PPP2R5E	SOX2 regulated genes in Wounding from PMID: 30772301	SOX2
RBBP8NL	SOX2 regulated genes in Wounding from PMID: 30772301	SOX2
CSTB	SOX2 regulated genes in Wounding from PMID: 30772301	SOX2
TP53INP2	SOX2 regulated genes in Wounding from PMID: 30772301	SOX2
BMP7	SOX2 regulated genes in Wounding from PMID: 30772301	SOX2
CLDN8	SOX2 regulated genes in Wounding from PMID: 30772301	SOX2
RPRD1A	SOX2 regulated genes in Wounding from PMID: 30772301	SOX2
ATP9A	SOX2 regulated genes in Wounding from PMID: 30772301	SOX2
SYN3	SOX2 regulated genes in Wounding from PMID: 30772301	SOX2
PCGF3	SOX2 regulated genes in Wounding from PMID: 30772301	SOX2
SEC23B	SOX2 regulated genes in Wounding from PMID: 30772301	SOX2
PCBP3	SOX2 regulated genes in Wounding from PMID: 30772301	SOX2
PCNX4	SOX2 regulated genes in Wounding from PMID: 30772301	SOX2
PDRG1	SOX2 regulated genes in Wounding from PMID: 30772301	SOX2
RGP1	SOX2 regulated genes in Wounding from PMID: 30772301	SOX2
OSBPL3	SOX2 regulated genes in Wounding from PMID: 30772301	SOX2
PTPN3	SOX2 regulated genes in Wounding from PMID: 30772301	SOX2
DNASE1L2	SOX2 regulated genes in Wounding from PMID: 30772301	SOX2
TNFRSF13C	SOX2 regulated genes in Wounding from PMID: 30772301	SOX2
RNPS1	SOX2 regulated genes in Wounding from PMID: 30772301	SOX2
SPEF1	SOX2 regulated genes in Wounding from PMID: 30772301	SOX2
RASL10A	SOX2 regulated genes in Wounding from PMID: 30772301	SOX2
OSER1	SOX2 regulated genes in Wounding from PMID: 30772301	SOX2
TES	SOX2 regulated genes in Wounding from PMID: 30772301	SOX2
SPIDR	SOX2 regulated genes in Wounding from PMID: 30772301	SOX2

CAGE1	SOX2 regulated genes in Wounding from PMID: 30772301	SOX2
PNP	SOX2 regulated genes in Wounding from PMID: 30772301	SOX2
ARID3A	SOX2 regulated genes in Wounding from PMID: 30772301	SOX2
BNIP3L	SOX2 regulated genes in Wounding from PMID: 30772301	SOX2
CSPG4	SOX2 regulated genes in Wounding from PMID: 30772301	SOX2
PRRG4	SOX2 regulated genes in Wounding from PMID: 30772301	SOX2
PDAP1	SOX2 regulated genes in Wounding from PMID: 30772301	SOX2
CELSR1	SOX2 regulated genes in Wounding from PMID: 30772301	SOX2
PJA1	SOX2 regulated genes in Wounding from PMID: 30772301	SOX2
COL4A5	SOX2 regulated genes in Wounding from PMID: 30772301	SOX2
AIMP2	SOX2 regulated genes in Wounding from PMID: 30772301	SOX2
DUSP9	SOX2 regulated genes in Wounding from PMID: 30772301	SOX2
MICALL2	SOX2 regulated genes in Wounding from PMID: 30772301	SOX2
FRYL	SOX2 regulated genes in Wounding from PMID: 30772301	SOX2
CD2AP	SOX2 regulated genes in Wounding from PMID: 30772301	SOX2
ITGA5	SOX2 regulated genes in Wounding from PMID: 30772301	SOX2
C8orf48	SOX2 regulated genes in Wounding from PMID: 30772301	SOX2
GSDMA	SOX2 regulated genes in Wounding from PMID: 30772301	SOX2
BBOX1	SOX2 regulated genes in Wounding from PMID: 30772301	SOX2
ANKRD50	SOX2 regulated genes in Wounding from PMID: 30772301	SOX2
PARM1	SOX2 regulated genes in Wounding from PMID: 30772301	SOX2
SMTN	SOX2 regulated genes in Wounding from PMID: 30772301	SOX2
PARD6B	SOX2 regulated genes in Wounding from PMID: 30772301	SOX2
SLC39A2	SOX2 regulated genes in Wounding from PMID: 30772301	SOX2
TIAM1	SOX2 regulated genes in Wounding from PMID: 30772301	SOX2
TAX1BP3	SOX2 regulated genes in Wounding from PMID: 30772301	SOX2
RBM12	SOX2 regulated genes in Wounding from PMID: 30772301	SOX2
QSOX2	SOX2 regulated genes in Wounding from PMID: 30772301	SOX2
STAR	SOX2 regulated genes in Wounding from PMID: 30772301	SOX2
JHY	SOX2 regulated genes in Wounding from PMID: 30772301	SOX2
CSNK2A3	SOX2 regulated genes in Wounding from PMID: 30772301	SOX2
CSNK2A1	SOX2 regulated genes in Wounding from PMID: 30772301	SOX2
TADA2B	SOX2 regulated genes in Wounding from PMID: 30772301	SOX2
CTBP2	SOX2 regulated genes in Wounding from PMID: 30772301	SOX2
SLC38A2	SOX2 regulated genes in Wounding from PMID: 30772301	SOX2
CARMIL1	SOX2 regulated genes in Wounding from PMID: 30772301	SOX2
FAM83G	SOX2 regulated genes in Wounding from PMID: 30772301	SOX2
LMX1B	SOX2 regulated genes in Wounding from PMID: 30772301	SOX2
LAMA1	SOX2 regulated genes in Wounding from PMID: 30772301	SOX2
GLS2	SOX2 regulated genes in Wounding from PMID: 30772301	SOX2
NUP58	SOX2 regulated genes in Wounding from PMID: 30772301	SOX2
GCOM1	SOX2 regulated genes in Wounding from PMID: 30772301	SOX2
SERPINA3	SOX2 regulated genes in Wounding from PMID: 30772301	SOX2
AMOTL1	SOX2 regulated genes in Wounding from PMID: 30772301	SOX2
PIP5K1B	SOX2 regulated genes in Wounding from PMID: 30772301	SOX2
DSC2	SOX2 regulated genes in Wounding from PMID: 30772301	SOX2
PTPN13	SOX2 regulated genes in Wounding from PMID: 30772301	SOX2
THNSL1	SOX2 regulated genes in Wounding from PMID: 30772301	SOX2
ORMDL3	SOX2 regulated genes in Wounding from PMID: 30772301	SOX2
GJA1	SOX2 regulated genes in Wounding from PMID: 30772301	SOX2
CASK	SOX2 regulated genes in Wounding from PMID: 30772301	SOX2
NCBP1	SOX2 regulated genes in Wounding from PMID: 30772301	SOX2
HNRNPK	SOX2 regulated genes in Wounding from PMID: 30772301	SOX2
DIO3	SOX2 regulated genes in Wounding from PMID: 30772301	SOX2
PPIL1	SOX2 regulated genes in Wounding from PMID: 30772301	SOX2
TLE1	SOX2 regulated genes in Wounding from PMID: 30772301	SOX2
DLGAP4	SOX2 regulated genes in Wounding from PMID: 30772301	SOX2
CYB5R4	SOX2 regulated genes in Wounding from PMID: 30772301	SOX2
TBX1	SOX2 regulated genes in Wounding from PMID: 30772301	SOX2

RNGTT	SOX2 regulated genes in Wounding from PMID: 30772301	SOX2
MYO1D	SOX2 regulated genes in Wounding from PMID: 30772301	SOX2
FMN1	SOX2 regulated genes in Wounding from PMID: 30772301	SOX2
POLR2M	SOX2 regulated genes in Wounding from PMID: 30772301	SOX2
EIF2B2	SOX2 regulated genes in Wounding from PMID: 30772301	SOX2
ABHD13	SOX2 regulated genes in Wounding from PMID: 30772301	SOX2
ARHGEF17	SOX2 regulated genes in Wounding from PMID: 30772301	SOX2
CCT6A	SOX2 regulated genes in Wounding from PMID: 30772301	SOX2
SMARCA5	SOX2 regulated genes in Wounding from PMID: 30772301	SOX2
SPIRE1	SOX2 regulated genes in Wounding from PMID: 30772301	SOX2
DCLK2	SOX2 regulated genes in Wounding from PMID: 30772301	SOX2
PRAG1	SOX2 regulated genes in Wounding from PMID: 30772301	SOX2
APLN	SOX2 regulated genes in Wounding from PMID: 30772301	SOX2
GGT1	SOX2 regulated genes in Wounding from PMID: 30772301	SOX2
NEDD9	SOX2 regulated genes in Wounding from PMID: 30772301	SOX2
ERI1	SOX2 regulated genes in Wounding from PMID: 30772301	SOX2
PFN1	SOX2 regulated genes in Wounding from PMID: 30772301	SOX2
PGAP3	SOX2 regulated genes in Wounding from PMID: 30772301	SOX2
BCR	SOX2 regulated genes in Wounding from PMID: 30772301	SOX2
ATP12A	SOX2 regulated genes in Wounding from PMID: 30772301	SOX2
PROCR	SOX2 regulated genes in Wounding from PMID: 30772301	SOX2
SEMA7A	SOX2 regulated genes in Wounding from PMID: 30772301	SOX2
EFNB2	SOX2 regulated genes in Wounding from PMID: 30772301	SOX2
TMEM154	SOX2 regulated genes in Wounding from PMID: 30772301	SOX2
ANXA1	SOX2 regulated genes in Wounding from PMID: 30772301	SOX2
SRSF6	SOX2 regulated genes in Wounding from PMID: 30772301	SOX2
ARHGDIG	SOX2 regulated genes in Wounding from PMID: 30772301	SOX2
FOXQ1	SOX2 regulated genes in Wounding from PMID: 30772301	SOX2
MBOAT1	SOX2 regulated genes in Wounding from PMID: 30772301	SOX2
GSPT1	SOX2 regulated genes in Wounding from PMID: 30772301	SOX2
GPR180	SOX2 regulated genes in Wounding from PMID: 30772301	SOX2
TMEM98	SOX2 regulated genes in Wounding from PMID: 30772301	SOX2
LRRC75B	SOX2 regulated genes in Wounding from PMID: 30772301	SOX2
LPIN3	SOX2 regulated genes in Wounding from PMID: 30772301	SOX2
ATP6V1E1	SOX2 regulated genes in Wounding from PMID: 30772301	SOX2
TMPRSS2	SOX2 regulated genes in Wounding from PMID: 30772301	SOX2
ABRACL	SOX2 regulated genes in Wounding from PMID: 30772301	SOX2
ITPRIP	SOX2 regulated genes in Wounding from PMID: 30772301	SOX2
CPNE8	SOX2 regulated genes in Wounding from PMID: 30772301	SOX2
EBPL	SOX2 regulated genes in Wounding from PMID: 30772301	SOX2
MYO10	SOX2 regulated genes in Wounding from PMID: 30772301	SOX2
SPIN1	SOX2 regulated genes in Wounding from PMID: 30772301	SOX2
SCML4	SOX2 regulated genes in Wounding from PMID: 30772301	SOX2
PARD6G	SOX2 regulated genes in Wounding from PMID: 30772301	SOX2
CDC6	SOX2 regulated genes in Wounding from PMID: 30772301	SOX2
TBC1D2	SOX2 regulated genes in Wounding from PMID: 30772301	SOX2
CDKL2	SOX2 regulated genes in Wounding from PMID: 30772301	SOX2
HIRA	SOX2 regulated genes in Wounding from PMID: 30772301	SOX2
CNN2	SOX2 regulated genes in Wounding from PMID: 30772301	SOX2
GATAD1	SOX2 regulated genes in Wounding from PMID: 30772301	SOX2
PORCN	SOX2 regulated genes in Wounding from PMID: 30772301	SOX2
SENP6	SOX2 regulated genes in Wounding from PMID: 30772301	SOX2
DIS3	SOX2 regulated genes in Wounding from PMID: 30772301	SOX2
HBS1L	SOX2 regulated genes in Wounding from PMID: 30772301	SOX2
HSBP1L1	SOX2 regulated genes in Wounding from PMID: 30772301	SOX2
CXXC4	SOX2 regulated genes in Wounding from PMID: 30772301	SOX2
CREB3	SOX2 regulated genes in Wounding from PMID: 30772301	SOX2
FEM1B	SOX2 regulated genes in Wounding from PMID: 30772301	SOX2
STRAP	SOX2 regulated genes in Wounding from PMID: 30772301	SOX2

TADA2A	SOX2 regulated genes in Wounding from PMID: 30772301	SOX2
ITPR3	SOX2 regulated genes in Wounding from PMID: 30772301	SOX2
MYBL2	SOX2 regulated genes in Wounding from PMID: 30772301	SOX2
SYT7	SOX2 regulated genes in Wounding from PMID: 30772301	SOX2
PLXNA3	SOX2 regulated genes in Wounding from PMID: 30772301	SOX2
FAM20C	SOX2 regulated genes in Wounding from PMID: 30772301	SOX2
SEC14L2	SOX2 regulated genes in Wounding from PMID: 30772301	SOX2
RWDD4	SOX2 regulated genes in Wounding from PMID: 30772301	SOX2
BICD2	SOX2 regulated genes in Wounding from PMID: 30772301	SOX2
HSP90AA1	SOX2 regulated genes in Wounding from PMID: 30772301	SOX2
FARP1	SOX2 regulated genes in Wounding from PMID: 30772301	SOX2
KREMEN1	SOX2 regulated genes in Wounding from PMID: 30772301	SOX2
SMAP1	SOX2 regulated genes in Wounding from PMID: 30772301	SOX2
CAAP1	SOX2 regulated genes in Wounding from PMID: 30772301	SOX2
MMP9	SOX2 regulated genes in Wounding from PMID: 30772301	SOX2
RHOV	SOX2 regulated genes in Wounding from PMID: 30772301	SOX2
THBS1	SOX2 regulated genes in Wounding from PMID: 30772301	SOX2
FANCF	SOX2 regulated genes in Wounding from PMID: 30772301	SOX2
PCDH19	SOX2 regulated genes in Wounding from PMID: 30772301	SOX2
BDKRB1	SOX2 regulated genes in Wounding from PMID: 30772301	SOX2
COG6	SOX2 regulated genes in Wounding from PMID: 30772301	SOX2
PAWR	SOX2 regulated genes in Wounding from PMID: 30772301	SOX2
DUSP14	SOX2 regulated genes in Wounding from PMID: 30772301	SOX2
NCKAP5L	SOX2 regulated genes in Wounding from PMID: 30772301	SOX2
GP1BB	SOX2 regulated genes in Wounding from PMID: 30772301	SOX2
CCDC120	SOX2 regulated genes in Wounding from PMID: 30772301	SOX2
HSP90AB1	SOX2 regulated genes in Wounding from PMID: 30772301	SOX2
CCDC25	SOX2 regulated genes in Wounding from PMID: 30772301	SOX2
SEC63	SOX2 regulated genes in Wounding from PMID: 30772301	SOX2
CCDC116	SOX2 regulated genes in Wounding from PMID: 30772301	SOX2
ENTPD7	SOX2 regulated genes in Wounding from PMID: 30772301	SOX2
LNX1	SOX2 regulated genes in Wounding from PMID: 30772301	SOX2
SMAD7	SOX2 regulated genes in Wounding from PMID: 30772301	SOX2
ACE2	SOX2 regulated genes in Wounding from PMID: 30772301	SOX2
ARID2	SOX2 regulated genes in Wounding from PMID: 30772301	SOX2
ARHGAP24	SOX2 regulated genes in Wounding from PMID: 30772301	SOX2
DEPDC7	SOX2 regulated genes in Wounding from PMID: 30772301	SOX2
CLNS1A	SOX2 regulated genes in Wounding from PMID: 30772301	SOX2
GSTCD	SOX2 regulated genes in Wounding from PMID: 30772301	SOX2
DERA	SOX2 regulated genes in Wounding from PMID: 30772301	SOX2
PRICKLE1	SOX2 regulated genes in Wounding from PMID: 30772301	SOX2
CAMSAP1	SOX2 regulated genes in Wounding from PMID: 30772301	SOX2
RERG	SOX2 regulated genes in Wounding from PMID: 30772301	SOX2
GFOD1	SOX2 regulated genes in Wounding from PMID: 30772301	SOX2
TMPRSS11E	SOX2 regulated genes in Wounding from PMID: 30772301	SOX2
MORC2	SOX2 regulated genes in Wounding from PMID: 30772301	SOX2
NIPSNAP1	SOX2 regulated genes in Wounding from PMID: 30772301	SOX2
GMEB2	SOX2 regulated genes in Wounding from PMID: 30772301	SOX2
FOXE1	SOX2 regulated genes in Wounding from PMID: 30772301	SOX2
RUNX2	SOX2 regulated genes in Wounding from PMID: 30772301	SOX2
C10orf88	SOX2 regulated genes in Wounding from PMID: 30772301	SOX2
DPH6	SOX2 regulated genes in Wounding from PMID: 30772301	SOX2
TBC1D10A	SOX2 regulated genes in Wounding from PMID: 30772301	SOX2
RFLNB	SOX2 regulated genes in Wounding from PMID: 30772301	SOX2
MINPP1	SOX2 regulated genes in Wounding from PMID: 30772301	SOX2
CD109	SOX2 regulated genes in Wounding from PMID: 30772301	SOX2
MTRR	SOX2 regulated genes in Wounding from PMID: 30772301	SOX2
NXN	SOX2 regulated genes in Wounding from PMID: 30772301	SOX2
SMOC1	SOX2 regulated genes in Wounding from PMID: 30772301	SOX2

TCF7L2	SOX2 regulated genes in Wounding from PMID: 30772301	SOX2
CRELD2	SOX2 regulated genes in Wounding from PMID: 30772301	SOX2
ATP6V1B2	SOX2 regulated genes in Wounding from PMID: 30772301	SOX2
CISD2	SOX2 regulated genes in Wounding from PMID: 30772301	SOX2
TMPRSS11F	SOX2 regulated genes in Wounding from PMID: 30772301	SOX2
GCLC	SOX2 regulated genes in Wounding from PMID: 30772301	SOX2
SPATA18	SOX2 regulated genes in Wounding from PMID: 30772301	SOX2
GBA2	SOX2 regulated genes in Wounding from PMID: 30772301	SOX2
CPXM2	SOX2 regulated genes in Wounding from PMID: 30772301	SOX2
RNF182	SOX2 regulated genes in Wounding from PMID: 30772301	SOX2
CDK5R1	SOX2 regulated genes in Wounding from PMID: 30772301	SOX2
AFAP1L2	SOX2 regulated genes in Wounding from PMID: 30772301	SOX2
PDLIM1	SOX2 regulated genes in Wounding from PMID: 30772301	SOX2
HDAC2	SOX2 regulated genes in Wounding from PMID: 30772301	SOX2
KCNE1	SOX2 regulated genes in Wounding from PMID: 30772301	SOX2
RCN2	SOX2 regulated genes in Wounding from PMID: 30772301	SOX2
PRSS12	SOX2 regulated genes in Wounding from PMID: 30772301	SOX2
CLU	SOX2 regulated genes in Wounding from PMID: 30772301	SOX2
MICAL3	SOX2 regulated genes in Wounding from PMID: 30772301	SOX2
CYP39A1	SOX2 regulated genes in Wounding from PMID: 30772301	SOX2
SLC15A1	SOX2 regulated genes in Wounding from PMID: 30772301	SOX2
SLC25A37	SOX2 regulated genes in Wounding from PMID: 30772301	SOX2
SERPINB5	SOX2 regulated genes in Wounding from PMID: 30772301	SOX2
SMAD6	SOX2 regulated genes in Wounding from PMID: 30772301	SOX2
HDDC2	SOX2 regulated genes in Wounding from PMID: 30772301	SOX2
PAQR8	SOX2 regulated genes in Wounding from PMID: 30772301	SOX2
TCFL5	SOX2 regulated genes in Wounding from PMID: 30772301	SOX2
HACE1	SOX2 regulated genes in Wounding from PMID: 30772301	SOX2
LRAT	SOX2 regulated genes in Wounding from PMID: 30772301	SOX2
CCDC169	SOX2 regulated genes in Wounding from PMID: 30772301	SOX2
RASSF6	SOX2 regulated genes in Wounding from PMID: 30772301	SOX2
ENTPD8	SOX2 regulated genes in Wounding from PMID: 30772301	SOX2
STYK1	SOX2 regulated genes in Wounding from PMID: 30772301	SOX2
RANGAP1	SOX2 regulated genes in Wounding from PMID: 30772301	SOX2
DDX27	SOX2 regulated genes in Wounding from PMID: 30772301	SOX2
PLCXD1	SOX2 regulated genes in Wounding from PMID: 30772301	SOX2
SLC6A8	SOX2 regulated genes in Wounding from PMID: 30772301	SOX2
SHROOM3	SOX2 regulated genes in Wounding from PMID: 30772301	SOX2
RANBP1	SOX2 regulated genes in Wounding from PMID: 30772301	SOX2
RIOK1	SOX2 regulated genes in Wounding from PMID: 30772301	SOX2
FOXC1	SOX2 regulated genes in Wounding from PMID: 30772301	SOX2
AK7	SOX2 regulated genes in Wounding from PMID: 30772301	SOX2
CWH43	SOX2 regulated genes in Wounding from PMID: 30772301	SOX2
PROSER2	SOX2 regulated genes in Wounding from PMID: 30772301	SOX2
PALLD	SOX2 regulated genes in Wounding from PMID: 30772301	SOX2
FAM83C	SOX2 regulated genes in Wounding from PMID: 30772301	SOX2
TDRD3	SOX2 regulated genes in Wounding from PMID: 30772301	SOX2
MAPK11	SOX2 regulated genes in Wounding from PMID: 30772301	SOX2
LAMA3	SOX2 regulated genes in Wounding from PMID: 30772301	SOX2
AJUBA	SOX2 regulated genes in Wounding from PMID: 30772301	SOX2
SERPINB13	SOX2 regulated genes in Wounding from PMID: 30772301	SOX2
ABTB2	SOX2 regulated genes in Wounding from PMID: 30772301	SOX2
DBNDD2	SOX2 regulated genes in Wounding from PMID: 30772301	SOX2
TM9SF3	SOX2 regulated genes in Wounding from PMID: 30772301	SOX2
MORF4L2	SOX2 regulated genes in Wounding from PMID: 30772301	SOX2
MAPK1	SOX2 regulated genes in Wounding from PMID: 30772301	SOX2
DLST	SOX2 regulated genes in Wounding from PMID: 30772301	SOX2
SWI5	SOX2 regulated genes in Wounding from PMID: 30772301	SOX2
MAGOHB	SOX2 regulated genes in Wounding from PMID: 30772301	SOX2

RAD23B	SOX2 regulated genes in Wounding from PMID: 30772301	SOX2
GPRIN3	SOX2 regulated genes in Wounding from PMID: 30772301	SOX2
IRX4	SOX2 regulated genes in Wounding from PMID: 30772301	SOX2
BMP6	SOX2 regulated genes in Wounding from PMID: 30772301	SOX2
NAA35	SOX2 regulated genes in Wounding from PMID: 30772301	SOX2
NUS1	SOX2 regulated genes in Wounding from PMID: 30772301	SOX2
SUCLA2	SOX2 regulated genes in Wounding from PMID: 30772301	SOX2
DSG2	SOX2 regulated genes in Wounding from PMID: 30772301	SOX2
CPNE5	SOX2 regulated genes in Wounding from PMID: 30772301	SOX2
CRK	SOX2 regulated genes in Wounding from PMID: 30772301	SOX2
RCAN1	SOX2 regulated genes in Wounding from PMID: 30772301	SOX2
FLNA	SOX2 regulated genes in Wounding from PMID: 30772301	SOX2
FJX1	SOX2 regulated genes in Wounding from PMID: 30772301	SOX2
B4GALNT3	SOX2 regulated genes in Wounding from PMID: 30772301	SOX2
CA12	SOX2 regulated genes in Wounding from PMID: 30772301	SOX2
SNX12	SOX2 regulated genes in Wounding from PMID: 30772301	SOX2
KCTD9	SOX2 regulated genes in Wounding from PMID: 30772301	SOX2
MEOX1	SOX2 regulated genes in Wounding from PMID: 30772301	SOX2
FERMT1	SOX2 regulated genes in Wounding from PMID: 30772301	SOX2
EDN1	SOX2 regulated genes in Wounding from PMID: 30772301	SOX2
PLP2	SOX2 regulated genes in Wounding from PMID: 30772301	SOX2
FAM199X	SOX2 regulated genes in Wounding from PMID: 30772301	SOX2
L3MBTL1	SOX2 regulated genes in Wounding from PMID: 30772301	SOX2
SPRYD3	SOX2 regulated genes in Wounding from PMID: 30772301	SOX2
TFIP11	SOX2 regulated genes in Wounding from PMID: 30772301	SOX2
GOPC	SOX2 regulated genes in Wounding from PMID: 30772301	SOX2
TMEFF1	SOX2 regulated genes in Wounding from PMID: 30772301	SOX2
DSC3	SOX2 regulated genes in Wounding from PMID: 30772301	SOX2
NFIL3	SOX2 regulated genes in Wounding from PMID: 30772301	SOX2
NUDT15	SOX2 regulated genes in Wounding from PMID: 30772301	SOX2
ARHGAP1	SOX2 regulated genes in Wounding from PMID: 30772301	SOX2
ST8SIA6	SOX2 regulated genes in Wounding from PMID: 30772301	SOX2
SLC24A3	SOX2 regulated genes in Wounding from PMID: 30772301	SOX2
PAK3	SOX2 regulated genes in Wounding from PMID: 30772301	SOX2
PLAGL2	SOX2 regulated genes in Wounding from PMID: 30772301	SOX2
LGR4	SOX2 regulated genes in Wounding from PMID: 30772301	SOX2
FHOD3	SOX2 regulated genes in Wounding from PMID: 30772301	SOX2
SNU13	SOX2 regulated genes in Wounding from PMID: 30772301	SOX2
KDM1B	SOX2 regulated genes in Wounding from PMID: 30772301	SOX2
GOLGA7B	SOX2 regulated genes in Wounding from PMID: 30772301	SOX2
BTBD9	SOX2 regulated genes in Wounding from PMID: 30772301	SOX2
CHAC1	SOX2 regulated genes in Wounding from PMID: 30772301	SOX2
ID4	SOX2 regulated genes in Wounding from PMID: 30772301	SOX2
PPDPF	SOX2 regulated genes in Wounding from PMID: 30772301	SOX2
CSTF3	SOX2 regulated genes in Wounding from PMID: 30772301	SOX2
RAP1GAP2	SOX2 regulated genes in Wounding from PMID: 30772301	SOX2
LMAN1L	SOX2 regulated genes in Wounding from PMID: 30772301	SOX2
ANG	SOX2 regulated genes in Wounding from PMID: 30772301	SOX2
MED27	SOX2 regulated genes in Wounding from PMID: 30772301	SOX2
EIF3A	SOX2 regulated genes in Wounding from PMID: 30772301	SOX2
LMTK2	SOX2 regulated genes in Wounding from PMID: 30772301	SOX2
SERPINE1	SOX2 regulated genes in Wounding from PMID: 30772301	SOX2
ENDOD1	SOX2 regulated genes in Wounding from PMID: 30772301	SOX2
DLG5	SOX2 regulated genes in Wounding from PMID: 30772301	SOX2
ARIH1	SOX2 regulated genes in Wounding from PMID: 30772301	SOX2
SF3A1	SOX2 regulated genes in Wounding from PMID: 30772301	SOX2
HSPA4L	SOX2 regulated genes in Wounding from PMID: 30772301	SOX2
PPP1R3B	SOX2 regulated genes in Wounding from PMID: 30772301	SOX2
PPP2R1B	SOX2 regulated genes in Wounding from PMID: 30772301	SOX2

NEDD4L	SOX2 regulated genes in Wounding from PMID: 30772301	SOX2
IPPK	SOX2 regulated genes in Wounding from PMID: 30772301	SOX2
THOC1	SOX2 regulated genes in Wounding from PMID: 30772301	SOX2
OVOL2	SOX2 regulated genes in Wounding from PMID: 30772301	SOX2
LEPROTL1	SOX2 regulated genes in Wounding from PMID: 30772301	SOX2
TCEAL3	SOX2 regulated genes in Wounding from PMID: 30772301	SOX2
SLC30A9	SOX2 regulated genes in Wounding from PMID: 30772301	SOX2
CHIC2	SOX2 regulated genes in Wounding from PMID: 30772301	SOX2
DSG1	SOX2 regulated genes in Wounding from PMID: 30772301	SOX2
PRDM1	SOX2 regulated genes in Wounding from PMID: 30772301	SOX2
LIN7C	SOX2 regulated genes in Wounding from PMID: 30772301	SOX2
COL17A1	SOX2 regulated genes in Wounding from PMID: 30772301	SOX2
GCH1	SOX2 regulated genes in Wounding from PMID: 30772301	SOX2
LONRF1	SOX2 regulated genes in Wounding from PMID: 30772301	SOX2
EVC2	SOX2 regulated genes in Wounding from PMID: 30772301	SOX2
MUC15	SOX2 regulated genes in Wounding from PMID: 30772301	SOX2
MYO1E	SOX2 regulated genes in Wounding from PMID: 30772301	SOX2
BTC	SOX2 regulated genes in Wounding from PMID: 30772301	SOX2
BCORL1	SOX2 regulated genes in Wounding from PMID: 30772301	SOX2
PDCL	SOX2 regulated genes in Wounding from PMID: 30772301	SOX2
C15orf62	SOX2 regulated genes in Wounding from PMID: 30772301	SOX2
CRNKL1	SOX2 regulated genes in Wounding from PMID: 30772301	SOX2
RRP7A	SOX2 regulated genes in Wounding from PMID: 30772301	SOX2
PLEKHA7	SOX2 regulated genes in Wounding from PMID: 30772301	SOX2
COBL	SOX2 regulated genes in Wounding from PMID: 30772301	SOX2
BCOR	SOX2 regulated genes in Wounding from PMID: 30772301	SOX2
GPAT3	SOX2 regulated genes in Wounding from PMID: 30772301	SOX2
CTDP1	SOX2 regulated genes in Wounding from PMID: 30772301	SOX2
DHX32	SOX2 regulated genes in Wounding from PMID: 30772301	SOX2
SLK	SOX2 regulated genes in Wounding from PMID: 30772301	SOX2
CSTF2	SOX2 regulated genes in Wounding from PMID: 30772301	SOX2
LIG3	SOX2 regulated genes in Wounding from PMID: 30772301	SOX2
EEF1G	SOX2 regulated genes in Wounding from PMID: 30772301	SOX2
CFAP46	SOX2 regulated genes in Wounding from PMID: 30772301	SOX2
PPP1R14C	SOX2 regulated genes in Wounding from PMID: 30772301	SOX2
STK26	SOX2 regulated genes in Wounding from PMID: 30772301	SOX2
MEI1	SOX2 regulated genes in Wounding from PMID: 30772301	SOX2
TLL1	SOX2 regulated genes in Wounding from PMID: 30772301	SOX2
TMTC4	SOX2 regulated genes in Wounding from PMID: 30772301	SOX2
GTF2E2	SOX2 regulated genes in Wounding from PMID: 30772301	SOX2
RHOD	SOX2 regulated genes in Wounding from PMID: 30772301	SOX2
HEPHL1	SOX2 regulated genes in Wounding from PMID: 30772301	SOX2
ERVFRD-1	SOX2 regulated genes in Wounding from PMID: 30772301	SOX2
RAB11FIP1	SOX2 regulated genes in Wounding from PMID: 30772301	SOX2
MAPRE1	SOX2 regulated genes in Wounding from PMID: 30772301	SOX2
CDCA4	SOX2 regulated genes in Wounding from PMID: 30772301	SOX2
USP22	SOX2 regulated genes in Wounding from PMID: 30772301	SOX2
TOP1	SOX2 regulated genes in Wounding from PMID: 30772301	SOX2
TSPAN14	SOX2 regulated genes in Wounding from PMID: 30772301	SOX2
TTPAL	SOX2 regulated genes in Wounding from PMID: 30772301	SOX2
VPS4B	SOX2 regulated genes in Wounding from PMID: 30772301	SOX2
XPNPEP1	SOX2 regulated genes in Wounding from PMID: 30772301	SOX2
ZMYM5	SOX2 regulated genes in Wounding from PMID: 30772301	SOX2
WIPF2	SOX2 regulated genes in Wounding from PMID: 30772301	SOX2
UTP14A	SOX2 regulated genes in Wounding from PMID: 30772301	SOX2
ZNF407	SOX2 regulated genes in Wounding from PMID: 30772301	SOX2
TOLLIP	SOX2 regulated genes in Wounding from PMID: 30772301	SOX2
USP6	SOX2 regulated genes in Wounding from PMID: 30772301	SOX2
TOM1	SOX2 regulated genes in Wounding from PMID: 30772301	SOX2

TRPM6	SOX2 regulated genes in Wounding from PMID: 30772301	SOX2
ZFAND2A	SOX2 regulated genes in Wounding from PMID: 30772301	SOX2
UBE2A	SOX2 regulated genes in Wounding from PMID: 30772301	SOX2
TUBB6	SOX2 regulated genes in Wounding from PMID: 30772301	SOX2
TRIM29	SOX2 regulated genes in Wounding from PMID: 30772301	SOX2
ZGPAT	SOX2 regulated genes in Wounding from PMID: 30772301	SOX2
ZMYND19	SOX2 regulated genes in Wounding from PMID: 30772301	SOX2
YWHAH	SOX2 regulated genes in Wounding from PMID: 30772301	SOX2
TULP3	SOX2 regulated genes in Wounding from PMID: 30772301	SOX2
TRIL	SOX2 regulated genes in Wounding from PMID: 30772301	SOX2
TRAPPC8	SOX2 regulated genes in Wounding from PMID: 30772301	SOX2
ZNF532	SOX2 regulated genes in Wounding from PMID: 30772301	SOX2
YAP1	SOX2 regulated genes in Wounding from PMID: 30772301	SOX2
ULK3	SOX2 regulated genes in Wounding from PMID: 30772301	SOX2
TXNDC5	SOX2 regulated genes in Wounding from PMID: 30772301	SOX2
WBP4	SOX2 regulated genes in Wounding from PMID: 30772301	SOX2
ZDHHC13	SOX2 regulated genes in Wounding from PMID: 30772301	SOX2
TSPAN9	SOX2 regulated genes in Wounding from PMID: 30772301	SOX2
TSPAN6	SOX2 regulated genes in Wounding from PMID: 30772301	SOX2
UNC5B	SOX2 regulated genes in Wounding from PMID: 30772301	SOX2
UMODL1	SOX2 regulated genes in Wounding from PMID: 30772301	SOX2
TXNDC17	SOX2 regulated genes in Wounding from PMID: 30772301	SOX2
TOMM22	SOX2 regulated genes in Wounding from PMID: 30772301	SOX2
ZDHHC9	SOX2 regulated genes in Wounding from PMID: 30772301	SOX2
ZNRF2	SOX2 regulated genes in Wounding from PMID: 30772301	SOX2
AFAP1	SOX2 regulated genes in Wounding from PMID: 30772301	SOX2
CCDC112	SOX2 regulated genes in Wounding from PMID: 30772301	SOX2
SYNE2	SOX2 regulated genes in Wounding from PMID: 30772301	SOX2
FHDC1	SOX2 regulated genes in Wounding from PMID: 30772301	SOX2
CCNC	SOX2 regulated genes in Wounding from PMID: 30772301	SOX2
SOCS5	SOX2 regulated genes in Wounding from PMID: 30772301	SOX2
NAP1L4	SOX2 regulated genes in Wounding from PMID: 30772301	SOX2
ITGA3	SOX2 regulated genes in Wounding from PMID: 30772301	SOX2
CLIC3	SOX2 regulated genes in Wounding from PMID: 30772301	SOX2
SERPINA1	SOX2 regulated genes in Wounding from PMID: 30772301	SOX2
AEBP2	SOX2 regulated genes in Wounding from PMID: 30772301	SOX2
EFCAB1	SOX2 regulated genes in Wounding from PMID: 30772301	SOX2
MSANTD3	SOX2 regulated genes in Wounding from PMID: 30772301	SOX2
DNAJB13	SOX2 regulated genes in Wounding from PMID: 30772301	SOX2
NCS1	SOX2 regulated genes in Wounding from PMID: 30772301	SOX2
NOTCH1	SOX2 regulated genes in Wounding from PMID: 30772301	SOX2
NUP62CL	SOX2 regulated genes in Wounding from PMID: 30772301	SOX2
SMARCE1	SOX2 regulated genes in Wounding from PMID: 30772301	SOX2
GDPD5	SOX2 regulated genes in Wounding from PMID: 30772301	SOX2
CD151	SOX2 regulated genes in Wounding from PMID: 30772301	SOX2
MGAT4A	SOX2 regulated genes in Wounding from PMID: 30772301	SOX2
FAM135A	SOX2 regulated genes in Wounding from PMID: 30772301	SOX2
BEGAIN	SOX2 regulated genes in Wounding from PMID: 30772301	SOX2
CDK17	SOX2 regulated genes in Wounding from PMID: 30772301	SOX2
BRAF	SOX2 regulated genes in Wounding from PMID: 30772301	SOX2
ZNF597	SOX2 regulated genes in Wounding from PMID: 30772301	SOX2
UTP14C	SOX2 regulated genes in Wounding from PMID: 30772301	SOX2
KRTAP9-4	SOX2 regulated genes in Wounding from PMID: 30772301	SOX2
EFL1	SOX2 regulated genes in Wounding from PMID: 30772301	SOX2
RHBDL2	SOX2 regulated genes in Wounding from PMID: 30772301	SOX2
UBE2N	SOX2 regulated genes in Wounding from PMID: 30772301	SOX2
WAPL	SOX2 regulated genes in Wounding from PMID: 30772301	SOX2
CTNNB1	SOX2 regulated genes in Wounding from PMID: 30772301	SOX2
SHB	SOX2 regulated genes in Wounding from PMID: 30772301	SOX2

MYH9	SOX2 regulated genes in Wounding from PMID: 30772301	SOX2
SERINC2	SOX2 regulated genes in Wounding from PMID: 30772301	SOX2
ZNF664	SOX2 regulated genes in Wounding from PMID: 30772301	SOX2
OSBPL5	SOX2 regulated genes in Wounding from PMID: 30772301	SOX2
VPS35	SOX2 regulated genes in Wounding from PMID: 30772301	SOX2
SNX18	SOX2 regulated genes in Wounding from PMID: 30772301	SOX2
ATP11A	SOX2 regulated genes in Wounding from PMID: 30772301	SOX2
PRSS8	SOX2 regulated genes in Wounding from PMID: 30772301	SOX2
CPEB4	SOX2 regulated genes in Wounding from PMID: 30772301	SOX2
PLEK2	SOX2 regulated genes in Wounding from PMID: 30772301	SOX2
BICDL2	SOX2 regulated genes in Wounding from PMID: 30772301	SOX2
LEO1	SOX2 regulated genes in Wounding from PMID: 30772301	SOX2
NOCT	SOX2 regulated genes in Wounding from PMID: 30772301	SOX2
UBAP1	SOX2 regulated genes in Wounding from PMID: 30772301	SOX2
RAB23	SOX2 regulated genes in Wounding from PMID: 30772301	SOX2
GUCA1B	SOX2 regulated genes in Wounding from PMID: 30772301	SOX2
ZDHHC5	SOX2 regulated genes in Wounding from PMID: 30772301	SOX2
BTBD11	SOX2 regulated genes in Wounding from PMID: 30772301	SOX2
NCOR2	SOX2 regulated genes in Wounding from PMID: 30772301	SOX2
TMEM51	SOX2 regulated genes in Wounding from PMID: 30772301	SOX2
TGFA	SOX2 regulated genes in Wounding from PMID: 30772301	SOX2
LPAR6	SOX2 regulated genes in Wounding from PMID: 30772301	SOX2
MPHOSPH6	SOX2 regulated genes in Wounding from PMID: 30772301	SOX2
MICB	SOX2 regulated genes in Wounding from PMID: 30772301	SOX2
SLC39A4	SOX2 regulated genes in Wounding from PMID: 30772301	SOX2
TMEM151A	SOX2 regulated genes in Wounding from PMID: 30772301	SOX2
TPM4	SOX2 regulated genes in Wounding from PMID: 30772301	SOX2
DGKA	SOX2 regulated genes in Wounding from PMID: 30772301	SOX2
TGM7	SOX2 regulated genes in Wounding from PMID: 30772301	SOX2
DDX17	SOX2 regulated genes in Wounding from PMID: 30772301	SOX2
ZNF365	SOX2 regulated genes in Wounding from PMID: 30772301	SOX2
KAZN	SOX2 regulated genes in Wounding from PMID: 30772301	SOX2
CD2BP2	SOX2 regulated genes in Wounding from PMID: 30772301	SOX2
BMP3	SOX2 regulated genes in Wounding from PMID: 30772301	SOX2
GNL2	SOX2 regulated genes in Wounding from PMID: 30772301	SOX2
SFR1	SOX2 regulated genes in Wounding from PMID: 30772301	SOX2
AREG	SOX2 regulated genes in Wounding from PMID: 30772301	SOX2
DUSP5	SOX2 regulated genes in Wounding from PMID: 30772301	SOX2
CUX1	SOX2 regulated genes in Wounding from PMID: 30772301	SOX2
CPE	SOX2 regulated genes in Wounding from PMID: 30772301	SOX2
ESRP2	SOX2 regulated genes in Wounding from PMID: 30772301	SOX2
CA9	SOX2 regulated genes in Wounding from PMID: 30772301	SOX2
PRKCH	SOX2 regulated genes in Wounding from PMID: 30772301	SOX2
PAK1	SOX2 regulated genes in Wounding from PMID: 30772301	SOX2
CPA4	SOX2 regulated genes in Wounding from PMID: 30772301	SOX2
SNAI2	SOX2 regulated genes in Wounding from PMID: 30772301	SOX2
DNAJB6	SOX2 regulated genes in Wounding from PMID: 30772301	SOX2
BAG2	SOX2 regulated genes in Wounding from PMID: 30772301	SOX2
KRT72	SOX2 regulated genes in Wounding from PMID: 30772301	SOX2
DDX18	SOX2 regulated genes in Wounding from PMID: 30772301	SOX2
PUM3	SOX2 regulated genes in Wounding from PMID: 30772301	SOX2
REPS1	SOX2 regulated genes in Wounding from PMID: 30772301	SOX2
CDK4	SOX2 regulated genes in Wounding from PMID: 30772301	SOX2
FBXW8	SOX2 regulated genes in Wounding from PMID: 30772301	SOX2
HAS2	SOX2 regulated genes in Wounding from PMID: 30772301	SOX2
SYNJ2	SOX2 regulated genes in Wounding from PMID: 30772301	SOX2
FHL2	SOX2 regulated genes in Wounding from PMID: 30772301	SOX2
GALR3	SOX2 regulated genes in Wounding from PMID: 30772301	SOX2
PPP1R37	SOX2 regulated genes in Wounding from PMID: 30772301	SOX2

UBE2Z	SOX2 regulated genes in Wounding from PMID: 30772301	SOX2
RIOX2	SOX2 regulated genes in Wounding from PMID: 30772301	SOX2
MAPKAPK3	SOX2 regulated genes in Wounding from PMID: 30772301	SOX2
STRBP	SOX2 regulated genes in Wounding from PMID: 30772301	SOX2
TENM4	SOX2 regulated genes in Wounding from PMID: 30772301	SOX2
PARD3	SOX2 regulated genes in Wounding from PMID: 30772301	SOX2
ST3GAL2	SOX2 regulated genes in Wounding from PMID: 30772301	SOX2
TRIP6	SOX2 regulated genes in Wounding from PMID: 30772301	SOX2
KLC2	SOX2 regulated genes in Wounding from PMID: 30772301	SOX2
SRD5A2	SOX2 regulated genes in Wounding from PMID: 30772301	SOX2
STX1A	SOX2 regulated genes in Wounding from PMID: 30772301	SOX2
SLC5A5	SOX2 regulated genes in Wounding from PMID: 30772301	SOX2
SCX	SOX2 regulated genes in Wounding from PMID: 30772301	SOX2
TGM6	SOX2 regulated genes in Wounding from PMID: 30772301	SOX2
TMC7	SOX2 regulated genes in Wounding from PMID: 30772301	SOX2
SIPA1L3	SOX2 regulated genes in Wounding from PMID: 30772301	SOX2
OTUD6B	SOX2 regulated genes in Wounding from PMID: 30772301	SOX2
TMEM163	SOX2 regulated genes in Wounding from PMID: 30772301	SOX2
GPSM1	SOX2 regulated genes in Wounding from PMID: 30772301	SOX2
FSCN1	SOX2 regulated genes in Wounding from PMID: 30772301	SOX2
ATP6V1C2	SOX2 regulated genes in Wounding from PMID: 30772301	SOX2
NDFIP2	SOX2 regulated genes in Wounding from PMID: 30772301	SOX2
EEF1AKMT1	SOX2 regulated genes in Wounding from PMID: 30772301	SOX2
SKIL	SOX2 regulated genes in Wounding from PMID: 30772301	SOX2
USP20	SOX2 regulated genes in Wounding from PMID: 30772301	SOX2
SMDT1	SOX2 regulated genes in Wounding from PMID: 30772301	SOX2
IFITM10	SOX2 regulated genes in Wounding from PMID: 30772301	SOX2
RAB18	SOX2 regulated genes in Wounding from PMID: 30772301	SOX2
STARD4	SOX2 regulated genes in Wounding from PMID: 30772301	SOX2
ANKRD6	SOX2 regulated genes in Wounding from PMID: 30772301	SOX2
EGR4	SOX2 regulated genes in Wounding from PMID: 30772301	SOX2
FZD6	SOX2 regulated genes in Wounding from PMID: 30772301	SOX2
CRB3	SOX2 regulated genes in Wounding from PMID: 30772301	SOX2
CDKN2AIP	SOX2 regulated genes in Wounding from PMID: 30772301	SOX2
PHLDB2	SOX2 regulated genes in Wounding from PMID: 30772301	SOX2
TMTC3	SOX2 regulated genes in Wounding from PMID: 30772301	SOX2
EPPK1	SOX2 regulated genes in Wounding from PMID: 30772301	SOX2
SLC7A6	SOX2 regulated genes in Wounding from PMID: 30772301	SOX2
LAP3	SOX2 regulated genes in Wounding from PMID: 30772301	SOX2
ARHGEF16	SOX2 regulated genes in Wounding from PMID: 30772301	SOX2
SSRP1	SOX2 regulated genes in Wounding from PMID: 30772301	SOX2
MSX2	SOX2 regulated genes in Wounding from PMID: 30772301	SOX2
BTBD10	SOX2 regulated genes in Wounding from PMID: 30772301	SOX2
LRRFIP2	SOX2 regulated genes in Wounding from PMID: 30772301	SOX2
GGACT	SOX2 regulated genes in Wounding from PMID: 30772301	SOX2
SLC30A4	SOX2 regulated genes in Wounding from PMID: 30772301	SOX2
SMYD5	SOX2 regulated genes in Wounding from PMID: 30772301	SOX2
TUBB4B	SOX2 regulated genes in Wounding from PMID: 30772301	SOX2
STIM2	SOX2 regulated genes in Wounding from PMID: 30772301	SOX2
EIF4A1	SOX2 regulated genes in Wounding from PMID: 30772301	SOX2
ANPEP	SOX2 regulated genes in Wounding from PMID: 30772301	SOX2
SERINC5	SOX2 regulated genes in Wounding from PMID: 30772301	SOX2
SCRG1	SOX2 regulated genes in Wounding from PMID: 30772301	SOX2
SH3BGRL2	SOX2 regulated genes in Wounding from PMID: 30772301	SOX2
HYAL2	SOX2 regulated genes in Wounding from PMID: 30772301	SOX2
GDE1	SOX2 regulated genes in Wounding from PMID: 30772301	SOX2
CHD7	SOX2 regulated genes in Wounding from PMID: 30772301	SOX2
ARL1	SOX2 regulated genes in Wounding from PMID: 30772301	SOX2
NIM1K	SOX2 regulated genes in Wounding from PMID: 30772301	SOX2

EIF3CL	SOX2 regulated genes in Wounding from PMID: 30772301	SOX2
EIF3C	SOX2 regulated genes in Wounding from PMID: 30772301	SOX2
SURF6	SOX2 regulated genes in Wounding from PMID: 30772301	SOX2
ESRP1	SOX2 regulated genes in Wounding from PMID: 30772301	SOX2
NAA15	SOX2 regulated genes in Wounding from PMID: 30772301	SOX2
SMURF1	SOX2 regulated genes in Wounding from PMID: 30772301	SOX2
FTSJ3	SOX2 regulated genes in Wounding from PMID: 30772301	SOX2
RABL2B	SOX2 regulated genes in Wounding from PMID: 30772301	SOX2
LPAR2	SOX2 regulated genes in Wounding from PMID: 30772301	SOX2
ZCCHC7	SOX2 regulated genes in Wounding from PMID: 30772301	SOX2
KLHL25	SOX2 regulated genes in Wounding from PMID: 30772301	SOX2
GRIPAP1	SOX2 regulated genes in Wounding from PMID: 30772301	SOX2
NBN	SOX2 regulated genes in Wounding from PMID: 30772301	SOX2
LGALS3	SOX2 regulated genes in Wounding from PMID: 30772301	SOX2
TMF1	SOX2 regulated genes in Wounding from PMID: 30772301	SOX2
DZIP1L	SOX2 regulated genes in Wounding from PMID: 30772301	SOX2
TUBB	SOX2 regulated genes in Wounding from PMID: 30772301	SOX2
ZNF274	SOX2 regulated genes in Wounding from PMID: 30772301	SOX2
GDNF	SOX2 regulated genes in Wounding from PMID: 30772301	SOX2
MIA-RAB4B	SOX2 regulated genes in Wounding from PMID: 30772301	SOX2
MIA	SOX2 regulated genes in Wounding from PMID: 30772301	SOX2
VPS36	SOX2 regulated genes in Wounding from PMID: 30772301	SOX2
MATN3	SOX2 regulated genes in Wounding from PMID: 30772301	SOX2
DCBLD1	SOX2 regulated genes in Wounding from PMID: 30772301	SOX2
CLDN12	SOX2 regulated genes in Wounding from PMID: 30772301	SOX2
CST6	SOX2 regulated genes in Wounding from PMID: 30772301	SOX2
EIF2S3	SOX2 regulated genes in Wounding from PMID: 30772301	SOX2
MAGEL2	SOX2 regulated genes in Wounding from PMID: 30772301	SOX2
GALNT14	SOX2 regulated genes in Wounding from PMID: 30772301	SOX2
YKT6	SOX2 regulated genes in Wounding from PMID: 30772301	SOX2
WNK2	SOX2 regulated genes in Wounding from PMID: 30772301	SOX2
TUBB2A	SOX2 regulated genes in Wounding from PMID: 30772301	SOX2
EOGT	SOX2 regulated genes in Wounding from PMID: 30772301	SOX2
ERCC6	SOX2 regulated genes in Wounding from PMID: 30772301	SOX2
ZDHHC21	SOX2 regulated genes in Wounding from PMID: 30772301	SOX2
TUBA1B	SOX2 regulated genes in Wounding from PMID: 30772301	SOX2
GDF7	SOX2 regulated genes in Wounding from PMID: 30772301	SOX2
IL6	SOX2 regulated genes in Wounding from PMID: 30772301	SOX2
PADI4	SOX2 regulated genes in Wounding from PMID: 30772301	SOX2
THSD1	SOX2 regulated genes in Wounding from PMID: 30772301	SOX2
EIF2S1	SOX2 regulated genes in Wounding from PMID: 30772301	SOX2
RHPN2	SOX2 regulated genes in Wounding from PMID: 30772301	SOX2
ZC3H15	SOX2 regulated genes in Wounding from PMID: 30772301	SOX2
FAM156A	SOX2 regulated genes in Wounding from PMID: 30772301	SOX2
UHRF1BP1L	SOX2 regulated genes in Wounding from PMID: 30772301	SOX2
TAB3	SOX2 regulated genes in Wounding from PMID: 30772301	SOX2
TAF4	SOX2 regulated genes in Wounding from PMID: 30772301	SOX2
PPP1CA	SOX2 regulated genes in Wounding from PMID: 30772301	SOX2
CYP21A2	SOX2 regulated genes in Wounding from PMID: 30772301	SOX2
CDSN	SOX2 regulated genes in Wounding from PMID: 30772301	SOX2
TUBB2B	SOX2 regulated genes in Wounding from PMID: 30772301	SOX2
BHLHB9	SOX2 regulated genes in Wounding from PMID: 30772301	SOX2
RNF19A	SOX2 regulated genes in Wounding from PMID: 30772301	SOX2
SPATS2	SOX2 regulated genes in Wounding from PMID: 30772301	SOX2
ARHGEF26	SOX2 regulated genes in Wounding from PMID: 30772301	SOX2
NAB2	SOX2 regulated genes in Wounding from PMID: 30772301	SOX2
CABLES2	SOX2 regulated genes in Wounding from PMID: 30772301	SOX2
CREG2	SOX2 regulated genes in Wounding from PMID: 30772301	SOX2
MTHFD2L	SOX2 regulated genes in Wounding from PMID: 30772301	SOX2

RNF185	SOX2 regulated genes in Wounding from PMID: 30772301	SOX2
PKP3	SOX2 regulated genes in Wounding from PMID: 30772301	SOX2
GNL3L	SOX2 regulated genes in Wounding from PMID: 30772301	SOX2
ATF4	SOX2 regulated genes in Wounding from PMID: 30772301	SOX2
GJB6	SOX2 regulated genes in Wounding from PMID: 30772301	SOX2
CRIP2	SOX2 regulated genes in Wounding from PMID: 30772301	SOX2
MAEA	SOX2 regulated genes in Wounding from PMID: 30772301	SOX2
USP38	SOX2 regulated genes in Wounding from PMID: 30772301	SOX2
COL4A6	SOX2 regulated genes in Wounding from PMID: 30772301	SOX2
ILDR1	SOX2 regulated genes in Wounding from PMID: 30772301	SOX2
MET	SOX2 regulated genes in Wounding from PMID: 30772301	SOX2
MRPL48	SOX2 regulated genes in Wounding from PMID: 30772301	SOX2
ZNF507	SOX2 regulated genes in Wounding from PMID: 30772301	SOX2
OXSR1	SOX2 regulated genes in Wounding from PMID: 30772301	SOX2
PTBP3	SOX2 regulated genes in Wounding from PMID: 30772301	SOX2
GIPC1	SOX2 regulated genes in Wounding from PMID: 30772301	SOX2
ADD2	SOX2 regulated genes in Wounding from PMID: 30772301	SOX2
FBXW7	SOX2 regulated genes in Wounding from PMID: 30772301	SOX2
TRAPPC13	SOX2 regulated genes in Wounding from PMID: 30772301	SOX2
MARK3	SOX2 regulated genes in Wounding from PMID: 30772301	SOX2
HNRNPA2B1	SOX2 regulated genes in Wounding from PMID: 30772301	SOX2
ZBTB44	SOX2 regulated genes in Wounding from PMID: 30772301	SOX2
BAMBI	SOX2 regulated genes in Wounding from PMID: 30772301	SOX2
CEACAM21	SOX2 regulated genes in Wounding from PMID: 30772301	SOX2
WASF1	SOX2 regulated genes in Wounding from PMID: 30772301	SOX2
CTNS	SOX2 regulated genes in Wounding from PMID: 30772301	SOX2
PELO	SOX2 regulated genes in Wounding from PMID: 30772301	SOX2
UBE4B	SOX2 regulated genes in Wounding from PMID: 30772301	SOX2
PDZD7	SOX2 regulated genes in Wounding from PMID: 30772301	SOX2
CNKSR1	SOX2 regulated genes in Wounding from PMID: 30772301	SOX2
GALT	SOX2 regulated genes in Wounding from PMID: 30772301	SOX2
METAP2	SOX2 regulated genes in Wounding from PMID: 30772301	SOX2
PLSCR3	SOX2 regulated genes in Wounding from PMID: 30772301	SOX2
ABCE1	SOX2 regulated genes in Wounding from PMID: 30772301	SOX2
RAB6A	SOX2 regulated genes in Wounding from PMID: 30772301	SOX2
CMTM8	SOX2 regulated genes in Wounding from PMID: 30772301	SOX2
FCHSD1	SOX2 regulated genes in Wounding from PMID: 30772301	SOX2
CRIM1	SOX2 regulated genes in Wounding from PMID: 30772301	SOX2
JUP	SOX2 regulated genes in Wounding from PMID: 30772301	SOX2
GRHL2	SOX2 regulated genes in Wounding from PMID: 30772301	SOX2
HSPA1A	SOX2 regulated genes in Wounding from PMID: 30772301	SOX2
HSPA1B	SOX2 regulated genes in Wounding from PMID: 30772301	SOX2
NEBL	SOX2 regulated genes in Wounding from PMID: 30772301	SOX2
FBLIM1	SOX2 regulated genes in Wounding from PMID: 30772301	SOX2
SHISA2	SOX2 regulated genes in Wounding from PMID: 30772301	SOX2
PPFIA1	SOX2 regulated genes in Wounding from PMID: 30772301	SOX2
UCP2	SOX2 regulated genes in Wounding from PMID: 30772301	SOX2
COPS5	SOX2 regulated genes in Wounding from PMID: 30772301	SOX2
DAPK2	SOX2 regulated genes in Wounding from PMID: 30772301	SOX2
ESYT3	SOX2 regulated genes in Wounding from PMID: 30772301	SOX2
CDK12	SOX2 regulated genes in Wounding from PMID: 30772301	SOX2
BASP1	SOX2 regulated genes in Wounding from PMID: 30772301	SOX2
MAP3K5	SOX2 regulated genes in Wounding from PMID: 30772301	SOX2
AP3M1	SOX2 regulated genes in Wounding from PMID: 30772301	SOX2
CNPY4	SOX2 regulated genes in Wounding from PMID: 30772301	SOX2
PFKP	SOX2 regulated genes in Wounding from PMID: 30772301	SOX2
PXDC1	SOX2 regulated genes in Wounding from PMID: 30772301	SOX2
EHD3	SOX2 regulated genes in Wounding from PMID: 30772301	SOX2
EXOSC3	SOX2 regulated genes in Wounding from PMID: 30772301	SOX2

BCL2L2	SOX2 regulated genes in Wounding from PMID: 30772301	SOX2
PPP4C	SOX2 regulated genes in Wounding from PMID: 30772301	SOX2
SRF	SOX2 regulated genes in Wounding from PMID: 30772301	SOX2
BCAR3	SOX2 regulated genes in Wounding from PMID: 30772301	SOX2
ATP8A2	SOX2 regulated genes in Wounding from PMID: 30772301	SOX2
RCN1	SOX2 regulated genes in Wounding from PMID: 30772301	SOX2
ALCAM	SOX2 regulated genes in Wounding from PMID: 30772301	SOX2
HYAL4	SOX2 regulated genes in Wounding from PMID: 30772301	SOX2
RFLNA	SOX2 regulated genes in Wounding from PMID: 30772301	SOX2
TNIK	SOX2 regulated genes in Wounding from PMID: 30772301	SOX2
DMTF1	SOX2 regulated genes in Wounding from PMID: 30772301	SOX2
RIN1	SOX2 regulated genes in Wounding from PMID: 30772301	SOX2
CALML3	SOX2 regulated genes in Wounding from PMID: 30772301	SOX2
MREG	SOX2 regulated genes in Wounding from PMID: 30772301	SOX2
YWHAG	SOX2 regulated genes in Wounding from PMID: 30772301	SOX2
GPC2	SOX2 regulated genes in Wounding from PMID: 30772301	SOX2
UBE2D3	SOX2 regulated genes in Wounding from PMID: 30772301	SOX2
FZD1	SOX2 regulated genes in Wounding from PMID: 30772301	SOX2
BARX2	SOX2 regulated genes in Wounding from PMID: 30772301	SOX2
SLC22A23	SOX2 regulated genes in Wounding from PMID: 30772301	SOX2
TNKS1BP1	SOX2 regulated genes in Wounding from PMID: 30772301	SOX2
WNT10B	SOX2 regulated genes in Wounding from PMID: 30772301	SOX2
CCR4	SOX2 regulated genes in Wounding from PMID: 30772301	SOX2
DAAM1	SOX2 regulated genes in Wounding from PMID: 30772301	SOX2
HCFC2	SOX2 regulated genes in Wounding from PMID: 30772301	SOX2
MTERF4	SOX2 regulated genes in Wounding from PMID: 30772301	SOX2
ATP10B	SOX2 regulated genes in Wounding from PMID: 30772301	SOX2
TMPRSS13	SOX2 regulated genes in Wounding from PMID: 30772301	SOX2
TACSTD2	SOX2 regulated genes in Wounding from PMID: 30772301	SOX2
TRPV6	SOX2 regulated genes in Wounding from PMID: 30772301	SOX2
TMPRSS7	SOX2 regulated genes in Wounding from PMID: 30772301	SOX2
TTLL10	SOX2 regulated genes in Wounding from PMID: 30772301	SOX2
TRIB1	SOX2 regulated genes in Wounding from PMID: 30772301	SOX2
SCNN1G	SOX2 regulated genes in Wounding from PMID: 30772301	SOX2
CHMP3	SOX2 regulated genes in Wounding from PMID: 30772301	SOX2
RNF103-CHMP3	SOX2 regulated genes in Wounding from PMID: 30772301	SOX2
BPGM	SOX2 regulated genes in Wounding from PMID: 30772301	SOX2
PRRC2B	SOX2 regulated genes in Wounding from PMID: 30772301	SOX2
ALDH3A2	SOX2 regulated genes in Wounding from PMID: 30772301	SOX2
SLC25A13	SOX2 regulated genes in Wounding from PMID: 30772301	SOX2
BCL7A	SOX2 regulated genes in Wounding from PMID: 30772301	SOX2
SERTAD1	SOX2 regulated genes in Wounding from PMID: 30772301	SOX2
GPNMB	SOX2 regulated genes in Wounding from PMID: 30772301	SOX2
SAE1	SOX2 regulated genes in Wounding from PMID: 30772301	SOX2
DNAJC6	SOX2 regulated genes in Wounding from PMID: 30772301	SOX2
STOX1	SOX2 regulated genes in Wounding from PMID: 30772301	SOX2
DYNC1LI2	SOX2 regulated genes in Wounding from PMID: 30772301	SOX2
ZMYM2	SOX2 regulated genes in Wounding from PMID: 30772301	SOX2
RABGEF1	SOX2 regulated genes in Wounding from PMID: 30772301	SOX2
STBD1	SOX2 regulated genes in Wounding from PMID: 30772301	SOX2
INTS6	SOX2 regulated genes in Wounding from PMID: 30772301	SOX2
AP1AR	SOX2 regulated genes in Wounding from PMID: 30772301	SOX2
DDX51	SOX2 regulated genes in Wounding from PMID: 30772301	SOX2
PI4K2B	SOX2 regulated genes in Wounding from PMID: 30772301	SOX2
PUF60	SOX2 regulated genes in Wounding from PMID: 30772301	SOX2
SCT	SOX2 regulated genes in Wounding from PMID: 30772301	SOX2
ANAPC2	SOX2 regulated genes in Wounding from PMID: 30772301	SOX2
CYP4F12	SOX2 regulated genes in Wounding from PMID: 30772301	SOX2
CDCP1	SOX2 regulated genes in Wounding from PMID: 30772301	SOX2

TJP2	SOX2 regulated genes in Wounding from PMID: 30772301	SOX2
VCP	SOX2 regulated genes in Wounding from PMID: 30772301	SOX2
PDZRN3	SOX2 regulated genes in Wounding from PMID: 30772301	SOX2
SRC	SOX2 regulated genes in Wounding from PMID: 30772301	SOX2
CA6	SOX2 regulated genes in Wounding from PMID: 30772301	SOX2
LMNB1	SOX2 regulated genes in Wounding from PMID: 30772301	SOX2
SH3YL1	SOX2 regulated genes in Wounding from PMID: 30772301	SOX2
JMY	SOX2 regulated genes in Wounding from PMID: 30772301	SOX2
NUMB	SOX2 regulated genes in Wounding from PMID: 30772301	SOX2
TOB1	SOX2 regulated genes in Wounding from PMID: 30772301	SOX2
GIPC2	SOX2 regulated genes in Wounding from PMID: 30772301	SOX2
FOXK2	SOX2 regulated genes in Wounding from PMID: 30772301	SOX2
IMP4	SOX2 regulated genes in Wounding from PMID: 30772301	SOX2
KATNBL1	SOX2 regulated genes in Wounding from PMID: 30772301	SOX2
HSPA14	SOX2 regulated genes in Wounding from PMID: 30772301	SOX2
NFE2L3	SOX2 regulated genes in Wounding from PMID: 30772301	SOX2
YWHAZ	SOX2 regulated genes in Wounding from PMID: 30772301	SOX2
PTPRF	SOX2 regulated genes in Wounding from PMID: 30772301	SOX2
SLC38A5	SOX2 regulated genes in Wounding from PMID: 30772301	SOX2
ACP7	SOX2 regulated genes in Wounding from PMID: 30772301	SOX2
KCTD4	SOX2 regulated genes in Wounding from PMID: 30772301	SOX2
HEPH	SOX2 regulated genes in Wounding from PMID: 30772301	SOX2
ETF1	SOX2 regulated genes in Wounding from PMID: 30772301	SOX2
FLRT2	SOX2 regulated genes in Wounding from PMID: 30772301	SOX2
GCNT4	SOX2 regulated genes in Wounding from PMID: 30772301	SOX2
NRBP2	SOX2 regulated genes in Wounding from PMID: 30772301	SOX2
PNLDC1	SOX2 regulated genes in Wounding from PMID: 30772301	SOX2
WHRN	SOX2 regulated genes in Wounding from PMID: 30772301	SOX2
TMEM64	SOX2 regulated genes in Wounding from PMID: 30772301	SOX2
TRO	SOX2 regulated genes in Wounding from PMID: 30772301	SOX2
PPARD	SOX2 regulated genes in Wounding from PMID: 30772301	SOX2
SH3D19	SOX2 regulated genes in Wounding from PMID: 30772301	SOX2
SRSF12	SOX2 regulated genes in Wounding from PMID: 30772301	SOX2
AGAP3	SOX2 regulated genes in Wounding from PMID: 30772301	SOX2
C5orf46	SOX2 regulated genes in Wounding from PMID: 30772301	SOX2
SYT17	SOX2 regulated genes in Wounding from PMID: 30772301	SOX2
PLAUR	SOX2 regulated genes in Wounding from PMID: 30772301	SOX2
SNN	SOX2 regulated genes in Wounding from PMID: 30772301	SOX2
UBAP2	SOX2 regulated genes in Wounding from PMID: 30772301	SOX2
MFSD13A	SOX2 regulated genes in Wounding from PMID: 30772301	SOX2
EPB41L4A	SOX2 regulated genes in Wounding from PMID: 30772301	SOX2
PREP	SOX2 regulated genes in Wounding from PMID: 30772301	SOX2
TSSK1B	SOX2 regulated genes in Wounding from PMID: 30772301	SOX2
ATP6V0A4	SOX2 regulated genes in Wounding from PMID: 30772301	SOX2
LXN	SOX2 regulated genes in Wounding from PMID: 30772301	SOX2
DUSP6	SOX2 regulated genes in Wounding from PMID: 30772301	SOX2
SATB2	SOX2 regulated genes in Wounding from PMID: 30772301	SOX2
GORASP2	SOX2 regulated genes in Wounding from PMID: 30772301	SOX2
KDM5C	SOX2 regulated genes in Wounding from PMID: 30772301	SOX2
DGKZ	SOX2 regulated genes in Wounding from PMID: 30772301	SOX2
OTUB2	SOX2 regulated genes in Wounding from PMID: 30772301	SOX2
EEA1	SOX2 regulated genes in Wounding from PMID: 30772301	SOX2
VSIG10	SOX2 regulated genes in Wounding from PMID: 30772301	SOX2
ALDH1A3	SOX2 regulated genes in Wounding from PMID: 30772301	SOX2
USP32	SOX2 regulated genes in Wounding from PMID: 30772301	SOX2
ANKLE1	SOX2 regulated genes in Wounding from PMID: 30772301	SOX2
AKIRIN2	SOX2 regulated genes in Wounding from PMID: 30772301	SOX2
SAMD5	SOX2 regulated genes in Wounding from PMID: 30772301	SOX2
DYNLT1	SOX2 regulated genes in Wounding from PMID: 30772301	SOX2

PTK2	SOX2 regulated genes in Wounding from PMID: 30772301	SOX2
CROT	SOX2 regulated genes in Wounding from PMID: 30772301	SOX2
XBP1	SOX2 regulated genes in Wounding from PMID: 30772301	SOX2
PTK2B	SOX2 regulated genes in Wounding from PMID: 30772301	SOX2
TCF7L1	SOX2 regulated genes in Wounding from PMID: 30772301	SOX2
CBX1	SOX2 regulated genes in Wounding from PMID: 30772301	SOX2
GABRP	SOX2 regulated genes in Wounding from PMID: 30772301	SOX2
FZD2	SOX2 regulated genes in Wounding from PMID: 30772301	SOX2
BAIAP2L1	SOX2 regulated genes in Wounding from PMID: 30772301	SOX2
PINLYP	SOX2 regulated genes in Wounding from PMID: 30772301	SOX2
STIP1	SOX2 regulated genes in Wounding from PMID: 30772301	SOX2
CLDND1	SOX2 regulated genes in Wounding from PMID: 30772301	SOX2
C1QTNF12	SOX2 regulated genes in Wounding from PMID: 30772301	SOX2
DYNLL1	SOX2 regulated genes in Wounding from PMID: 30772301	SOX2
SLCO4C1	SOX2 regulated genes in Wounding from PMID: 30772301	SOX2
GJB2	SOX2 regulated genes in Wounding from PMID: 30772301	SOX2
CCL27	SOX2 regulated genes in Wounding from PMID: 30772301	SOX2
GABARAPL2	SOX2 regulated genes in Wounding from PMID: 30772301	SOX2
CDC42BPG	SOX2 regulated genes in Wounding from PMID: 30772301	SOX2
GLB1L2	SOX2 regulated genes in Wounding from PMID: 30772301	SOX2
SLC35A2	SOX2 regulated genes in Wounding from PMID: 30772301	SOX2
JPH4	SOX2 regulated genes in Wounding from PMID: 30772301	SOX2
STRN	SOX2 regulated genes in Wounding from PMID: 30772301	SOX2
RET	SOX2 regulated genes in Wounding from PMID: 30772301	SOX2
GPR37	SOX2 regulated genes in Wounding from PMID: 30772301	SOX2
BOK	SOX2 regulated genes in Wounding from PMID: 30772301	SOX2
CTSF	SOX2 regulated genes in Wounding from PMID: 30772301	SOX2
RTKN2	SOX2 regulated genes in Wounding from PMID: 30772301	SOX2
PEBP1	SOX2 regulated genes in Wounding from PMID: 30772301	SOX2
C3orf18	SOX2 regulated genes in Wounding from PMID: 30772301	SOX2
GAN	SOX2 regulated genes in Wounding from PMID: 30772301	SOX2
EIF4A3	SOX2 regulated genes in Wounding from PMID: 30772301	SOX2
SOWAHB	SOX2 regulated genes in Wounding from PMID: 30772301	SOX2
SUN2	SOX2 regulated genes in Wounding from PMID: 30772301	SOX2
GPC1	SOX2 regulated genes in Wounding from PMID: 30772301	SOX2
TDRD9	SOX2 regulated genes in Wounding from PMID: 30772301	SOX2
BCO1	SOX2 regulated genes in Wounding from PMID: 30772301	SOX2
EML4	SOX2 regulated genes in Wounding from PMID: 30772301	SOX2
THAP1	SOX2 regulated genes in Wounding from PMID: 30772301	SOX2
RGS9	SOX2 regulated genes in Wounding from PMID: 30772301	SOX2
TTLL7	SOX2 regulated genes in Wounding from PMID: 30772301	SOX2
IGF1R	SOX2 regulated genes in Wounding from PMID: 30772301	SOX2
EPGN	SOX2 regulated genes in Wounding from PMID: 30772301	SOX2
VASN	SOX2 regulated genes in Wounding from PMID: 30772301	SOX2
CRISP3	SOX2 regulated genes in Wounding from PMID: 30772301	SOX2
EIF1AX	SOX2 regulated genes in Wounding from PMID: 30772301	SOX2
HSPA2	SOX2 regulated genes in Wounding from PMID: 30772301	SOX2
BTG1	SOX2 regulated genes in Wounding from PMID: 30772301	SOX2
KIAA1191	SOX2 regulated genes in Wounding from PMID: 30772301	SOX2
TPM1	SOX2 regulated genes in Wounding from PMID: 30772301	SOX2
UNC45A	SOX2 regulated genes in Wounding from PMID: 30772301	SOX2
EIF4G2	SOX2 regulated genes in Wounding from PMID: 30772301	SOX2
PPP1R36	SOX2 regulated genes in Wounding from PMID: 30772301	SOX2
DUSP2	SOX2 regulated genes in Wounding from PMID: 30772301	SOX2
C11orf52	SOX2 regulated genes in Wounding from PMID: 30772301	SOX2
TMEM62	SOX2 regulated genes in Wounding from PMID: 30772301	SOX2
CXCL14	SOX2 regulated genes in Wounding from PMID: 30772301	SOX2
CAMTA1	SOX2 regulated genes in Wounding from PMID: 30772301	SOX2
SERTAD2	SOX2 regulated genes in Wounding from PMID: 30772301	SOX2

PLS3	SOX2 regulated genes in Wounding from PMID: 30772301	SOX2
SIAH2	SOX2 regulated genes in Wounding from PMID: 30772301	SOX2
NOL10	SOX2 regulated genes in Wounding from PMID: 30772301	SOX2
ZNF827	SOX2 regulated genes in Wounding from PMID: 30772301	SOX2
ITGB6	SOX2 regulated genes in Wounding from PMID: 30772301	SOX2
EIF1AD	SOX2 regulated genes in Wounding from PMID: 30772301	SOX2
PCYOX1	SOX2 regulated genes in Wounding from PMID: 30772301	SOX2
IGF2BP1	SOX2 regulated genes in Wounding from PMID: 30772301	SOX2
FADS3	SOX2 regulated genes in Wounding from PMID: 30772301	SOX2
SMARCC1	SOX2 regulated genes in Wounding from PMID: 30772301	SOX2
MT4	SOX2 regulated genes in Wounding from PMID: 30772301	SOX2
ATXN2	SOX2 regulated genes in Wounding from PMID: 30772301	SOX2
FAM47E	SOX2 regulated genes in Wounding from PMID: 30772301	SOX2
STK39	SOX2 regulated genes in Wounding from PMID: 30772301	SOX2
ANO9	SOX2 regulated genes in Wounding from PMID: 30772301	SOX2
POLH	SOX2 regulated genes in Wounding from PMID: 30772301	SOX2
TNFAIP1	SOX2 regulated genes in Wounding from PMID: 30772301	SOX2
FAM107B	SOX2 regulated genes in Wounding from PMID: 30772301	SOX2
DENR	SOX2 regulated genes in Wounding from PMID: 30772301	SOX2
PPP2R5B	SOX2 regulated genes in Wounding from PMID: 30772301	SOX2
CCNO	SOX2 regulated genes in Wounding from PMID: 30772301	SOX2
HSD17B14	SOX2 regulated genes in Wounding from PMID: 30772301	SOX2
LY6D	SOX2 regulated genes in Wounding from PMID: 30772301	SOX2
CSRNP1	SOX2 regulated genes in Wounding from PMID: 30772301	SOX2
DLX2	SOX2 regulated genes in Wounding from PMID: 30772301	SOX2
CA2	SOX2 regulated genes in Wounding from PMID: 30772301	SOX2
SLC35F6	SOX2 regulated genes in Wounding from PMID: 30772301	SOX2
CARM1	SOX2 regulated genes in Wounding from PMID: 30772301	SOX2
PSMD12	SOX2 regulated genes in Wounding from PMID: 30772301	SOX2
EDF1	SOX2 regulated genes in Wounding from PMID: 30772301	SOX2
PLCXD2	SOX2 regulated genes in Wounding from PMID: 30772301	SOX2
DDX19A	SOX2 regulated genes in Wounding from PMID: 30772301	SOX2
PURB	SOX2 regulated genes in Wounding from PMID: 30772301	SOX2
SEC23IP	SOX2 regulated genes in Wounding from PMID: 30772301	SOX2
HIF1A	SOX2 regulated genes in Wounding from PMID: 30772301	SOX2
UBA2	SOX2 regulated genes in Wounding from PMID: 30772301	SOX2
ALG9	SOX2 regulated genes in Wounding from PMID: 30772301	SOX2
FBP1	SOX2 regulated genes in Wounding from PMID: 30772301	SOX2
N4BP3	SOX2 regulated genes in Wounding from PMID: 30772301	SOX2
CSNK1G3	SOX2 regulated genes in Wounding from PMID: 30772301	SOX2
PLEKHG3	SOX2 regulated genes in Wounding from PMID: 30772301	SOX2
PPP2R1A	SOX2 regulated genes in Wounding from PMID: 30772301	SOX2
BOP1	SOX2 regulated genes in Wounding from PMID: 30772301	SOX2
ZNF185	SOX2 regulated genes in Wounding from PMID: 30772301	SOX2
ZBTB1	SOX2 regulated genes in Wounding from PMID: 30772301	SOX2
OPLAH	SOX2 regulated genes in Wounding from PMID: 30772301	SOX2
KRT80	SOX2 regulated genes in Wounding from PMID: 30772301	SOX2
TRPM4	SOX2 regulated genes in Wounding from PMID: 30772301	SOX2
MAP3K20	SOX2 regulated genes in Wounding from PMID: 30772301	SOX2
OSGIN1	SOX2 regulated genes in Wounding from PMID: 30772301	SOX2
GPC3	SOX2 regulated genes in Wounding from PMID: 30772301	SOX2
POLR2J3	SOX2 regulated genes in Wounding from PMID: 30772301	SOX2
UPK3BL1	SOX2 regulated genes in Wounding from PMID: 30772301	SOX2
UPK3BL2	SOX2 regulated genes in Wounding from PMID: 30772301	SOX2
PLCH2	SOX2 regulated genes in Wounding from PMID: 30772301	SOX2
CNIH2	SOX2 regulated genes in Wounding from PMID: 30772301	SOX2
CRYM	SOX2 regulated genes in Wounding from PMID: 30772301	SOX2
EPS8L1	SOX2 regulated genes in Wounding from PMID: 30772301	SOX2
LCN12	SOX2 regulated genes in Wounding from PMID: 30772301	SOX2

GTF2H2	SOX2 regulated genes in Wounding from PMID: 30772301	SOX2
SLC20A2	SOX2 regulated genes in Wounding from PMID: 30772301	SOX2
SCAPER	SOX2 regulated genes in Wounding from PMID: 30772301	SOX2
SLC38A3	SOX2 regulated genes in Wounding from PMID: 30772301	SOX2
SH2D4A	SOX2 regulated genes in Wounding from PMID: 30772301	SOX2
NAE1	SOX2 regulated genes in Wounding from PMID: 30772301	SOX2
MYCL	SOX2 regulated genes in Wounding from PMID: 30772301	SOX2
YWHAQ	SOX2 regulated genes in Wounding from PMID: 30772301	SOX2
NEK5	SOX2 regulated genes in Wounding from PMID: 30772301	SOX2
PTPRN2	SOX2 regulated genes in Wounding from PMID: 30772301	SOX2
TAGLN3	SOX2 regulated genes in Wounding from PMID: 30772301	SOX2
METAP1	SOX2 regulated genes in Wounding from PMID: 30772301	SOX2
PLEKHG1	SOX2 regulated genes in Wounding from PMID: 30772301	SOX2
POLD4	SOX2 regulated genes in Wounding from PMID: 30772301	SOX2
CLDN3	SOX2 regulated genes in Wounding from PMID: 30772301	SOX2
SRRT	SOX2 regulated genes in Wounding from PMID: 30772301	SOX2
UNC93A	SOX2 regulated genes in Wounding from PMID: 30772301	SOX2
EIF4E	SOX2 regulated genes in Wounding from PMID: 30772301	SOX2
PXYLP1	SOX2 regulated genes in Wounding from PMID: 30772301	SOX2
EPCAM	SOX2 regulated genes in Wounding from PMID: 30772301	SOX2
WRNIP1	SOX2 regulated genes in Wounding from PMID: 30772301	SOX2
LATS1	SOX2 regulated genes in Wounding from PMID: 30772301	SOX2
PAPSS2	SOX2 regulated genes in Wounding from PMID: 30772301	SOX2
ANKH	SOX2 regulated genes in Wounding from PMID: 30772301	SOX2
PPP1CB	SOX2 regulated genes in Wounding from PMID: 30772301	SOX2
C4orf36	SOX2 regulated genes in Wounding from PMID: 30772301	SOX2
CLASRP	SOX2 regulated genes in Wounding from PMID: 30772301	SOX2
DHX16	SOX2 regulated genes in Wounding from PMID: 30772301	SOX2
PSCA	SOX2 regulated genes in Wounding from PMID: 30772301	SOX2
NECAB1	SOX2 regulated genes in Wounding from PMID: 30772301	SOX2
CKAP4	SOX2 regulated genes in Wounding from PMID: 30772301	SOX2
BNC2	SOX2 regulated genes in Wounding from PMID: 30772301	SOX2
PDZD2	SOX2 regulated genes in Wounding from PMID: 30772301	SOX2
ENOX1	SOX2 regulated genes in Wounding from PMID: 30772301	SOX2
PRKCI	SOX2 regulated genes in Wounding from PMID: 30772301	SOX2
XRCC1	SOX2 regulated genes in Wounding from PMID: 30772301	SOX2
MPRIP	SOX2 regulated genes in Wounding from PMID: 30772301	SOX2
BMPR1B	SOX2 regulated genes in Wounding from PMID: 30772301	SOX2
CCK	SOX2 regulated genes in Wounding from PMID: 30772301	SOX2
LHX2	SOX2 regulated genes in Wounding from PMID: 30772301	SOX2
SP6	SOX2 regulated genes in Wounding from PMID: 30772301	SOX2
MFSD6	SOX2 regulated genes in Wounding from PMID: 30772301	SOX2
TOM1L1	SOX2 regulated genes in Wounding from PMID: 30772301	SOX2
FAM83H	SOX2 regulated genes in Wounding from PMID: 30772301	SOX2
AFF4	SOX2 regulated genes in Wounding from PMID: 30772301	SOX2
PIP4K2C	SOX2 regulated genes in Wounding from PMID: 30772301	SOX2
RNF144B	SOX2 regulated genes in Wounding from PMID: 30772301	SOX2
RBM15B	SOX2 regulated genes in Wounding from PMID: 30772301	SOX2
LGALS12	SOX2 regulated genes in Wounding from PMID: 30772301	SOX2
ATP6V1G1	SOX2 regulated genes in Wounding from PMID: 30772301	SOX2
SCARB2	SOX2 regulated genes in Wounding from PMID: 30772301	SOX2
INTS8	SOX2 regulated genes in Wounding from PMID: 30772301	SOX2
MTFR1	SOX2 regulated genes in Wounding from PMID: 30772301	SOX2
AHI1	SOX2 regulated genes in Wounding from PMID: 30772301	SOX2
PGAP2	SOX2 regulated genes in Wounding from PMID: 30772301	SOX2
PHRF1	SOX2 regulated genes in Wounding from PMID: 30772301	SOX2
MYC	SOX2 regulated genes in Wounding from PMID: 30772301	SOX2
PPRC1	SOX2 regulated genes in Wounding from PMID: 30772301	SOX2
SIGMAR1	SOX2 regulated genes in Wounding from PMID: 30772301	SOX2

CLIC4	SOX2 regulated genes in Wounding from PMID: 30772301	SOX2
ARNTL	SOX2 regulated genes in Wounding from PMID: 30772301	SOX2
CADM4	SOX2 regulated genes in Wounding from PMID: 30772301	SOX2
GARNL3	SOX2 regulated genes in Wounding from PMID: 30772301	SOX2
SPINK5	SOX2 regulated genes in Wounding from PMID: 30772301	SOX2
PPP1R10	SOX2 regulated genes in Wounding from PMID: 30772301	SOX2
FFAR3	SOX2 regulated genes in Wounding from PMID: 30772301	SOX2
SEC16A	SOX2 regulated genes in Wounding from PMID: 30772301	SOX2
BCAR1	SOX2 regulated genes in Wounding from PMID: 30772301	SOX2
SC5D	SOX2 regulated genes in Wounding from PMID: 30772301	SOX2
HOMER2	SOX2 regulated genes in Wounding from PMID: 30772301	SOX2
MTHFSD	SOX2 regulated genes in Wounding from PMID: 30772301	SOX2
RIPK2	SOX2 regulated genes in Wounding from PMID: 30772301	SOX2
RPL27	SOX2 regulated genes in Wounding from PMID: 30772301	SOX2
MACC1	SOX2 regulated genes in Wounding from PMID: 30772301	SOX2
PAK4	SOX2 regulated genes in Wounding from PMID: 30772301	SOX2
FMNL2	SOX2 regulated genes in Wounding from PMID: 30772301	SOX2
LTV1	SOX2 regulated genes in Wounding from PMID: 30772301	SOX2
FBXO42	SOX2 regulated genes in Wounding from PMID: 30772301	SOX2
PRKCA	SOX2 regulated genes in Wounding from PMID: 30772301	SOX2
CMAS	SOX2 regulated genes in Wounding from PMID: 30772301	SOX2
CTNNA1	SOX2 regulated genes in Wounding from PMID: 30772301	SOX2
KLHL32	SOX2 regulated genes in Wounding from PMID: 30772301	SOX2
SNTB1	SOX2 regulated genes in Wounding from PMID: 30772301	SOX2
ANKRD27	SOX2 regulated genes in Wounding from PMID: 30772301	SOX2
PHACTR4	SOX2 regulated genes in Wounding from PMID: 30772301	SOX2
SORCS2	SOX2 regulated genes in Wounding from PMID: 30772301	SOX2
SLC9A2	SOX2 regulated genes in Wounding from PMID: 30772301	SOX2
AIG1	SOX2 regulated genes in Wounding from PMID: 30772301	SOX2
CALD1	SOX2 regulated genes in Wounding from PMID: 30772301	SOX2
LRP8	SOX2 regulated genes in Wounding from PMID: 30772301	SOX2
PEX26	SOX2 regulated genes in Wounding from PMID: 30772301	SOX2
MICAL1	SOX2 regulated genes in Wounding from PMID: 30772301	SOX2
HSPB1	SOX2 regulated genes in Wounding from PMID: 30772301	SOX2
AQP9	SOX2 regulated genes in Wounding from PMID: 30772301	SOX2
ZNF428	SOX2 regulated genes in Wounding from PMID: 30772301	SOX2
DUSP8	SOX2 regulated genes in Wounding from PMID: 30772301	SOX2
UBLCP1	SOX2 regulated genes in Wounding from PMID: 30772301	SOX2
PERP	SOX2 regulated genes in Wounding from PMID: 30772301	SOX2
CCT2	SOX2 regulated genes in Wounding from PMID: 30772301	SOX2
ATAD2B	SOX2 regulated genes in Wounding from PMID: 30772301	SOX2
NCK2	SOX2 regulated genes in Wounding from PMID: 30772301	SOX2
PLB1	SOX2 regulated genes in Wounding from PMID: 30772301	SOX2
DACT2	SOX2 regulated genes in Wounding from PMID: 30772301	SOX2
PPP2CA	SOX2 regulated genes in Wounding from PMID: 30772301	SOX2
KRT73	SOX2 regulated genes in Wounding from PMID: 30772301	SOX2
RAB5A	SOX2 regulated genes in Wounding from PMID: 30772301	SOX2
CD82	SOX2 regulated genes in Wounding from PMID: 30772301	SOX2
ITPKC	SOX2 regulated genes in Wounding from PMID: 30772301	SOX2
NUP62	SOX2 regulated genes in Wounding from PMID: 30772301	SOX2
GJA3	SOX2 regulated genes in Wounding from PMID: 30772301	SOX2
FBXO30	SOX2 regulated genes in Wounding from PMID: 30772301	SOX2
RNF6	SOX2 regulated genes in Wounding from PMID: 30772301	SOX2
ARHGEF19	SOX2 regulated genes in Wounding from PMID: 30772301	SOX2
TSPAN8	SOX2 regulated genes in Wounding from PMID: 30772301	SOX2
DLX3	SOX2 regulated genes in Wounding from PMID: 30772301	SOX2
KRT75	SOX2 regulated genes in Wounding from PMID: 30772301	SOX2
MAP3K9	SOX2 regulated genes in Wounding from PMID: 30772301	SOX2
NECTIN1	SOX2 regulated genes in Wounding from PMID: 30772301	SOX2

SUDS3	SOX2 regulated genes in Wounding from PMID: 30772301	SOX2
MSI1	SOX2 regulated genes in Wounding from PMID: 30772301	SOX2
SARNP	SOX2 regulated genes in Wounding from PMID: 30772301	SOX2
ASH2L	SOX2 regulated genes in Wounding from PMID: 30772301	SOX2
EPB41L4B	SOX2 regulated genes in Wounding from PMID: 30772301	SOX2
ERCC1	SOX2 regulated genes in Wounding from PMID: 30772301	SOX2
IER3	SOX2 regulated genes in Wounding from PMID: 30772301	SOX2
TFAP2A	SOX2 regulated genes in Wounding from PMID: 30772301	SOX2
PRSS53	SOX2 regulated genes in Wounding from PMID: 30772301	SOX2
CDH6	SOX2 regulated genes in Wounding from PMID: 30772301	SOX2
CAV2	SOX2 regulated genes in Wounding from PMID: 30772301	SOX2
BCAS1	SOX2 regulated genes in Wounding from PMID: 30772301	SOX2
BDKRB2	SOX2 regulated genes in Wounding from PMID: 30772301	SOX2
MYCBP	SOX2 regulated genes in Wounding from PMID: 30772301	SOX2
STOX2	SOX2 regulated genes in Wounding from PMID: 30772301	SOX2
NUP107	SOX2 regulated genes in Wounding from PMID: 30772301	SOX2
RAI14	SOX2 regulated genes in Wounding from PMID: 30772301	SOX2
TMEM74	SOX2 regulated genes in Wounding from PMID: 30772301	SOX2
NOXA1	SOX2 regulated genes in Wounding from PMID: 30772301	SOX2
TMEM115	SOX2 regulated genes in Wounding from PMID: 30772301	SOX2
UBA3	SOX2 regulated genes in Wounding from PMID: 30772301	SOX2
INHBB	SOX2 regulated genes in Wounding from PMID: 30772301	SOX2
EPHA2	SOX2 regulated genes in Wounding from PMID: 30772301	SOX2
CPT1A	SOX2 regulated genes in Wounding from PMID: 30772301	SOX2
UBXN2A	SOX2 regulated genes in Wounding from PMID: 30772301	SOX2
MICALL1	SOX2 regulated genes in Wounding from PMID: 30772301	SOX2
CPM	SOX2 regulated genes in Wounding from PMID: 30772301	SOX2
STEAP1B	SOX2 regulated genes in Wounding from PMID: 30772301	SOX2
BAG3	SOX2 regulated genes in Wounding from PMID: 30772301	SOX2
DCAF12	SOX2 regulated genes in Wounding from PMID: 30772301	SOX2
CTNNBIP1	SOX2 regulated genes in Wounding from PMID: 30772301	SOX2
KDM8	SOX2 regulated genes in Wounding from PMID: 30772301	SOX2
MANF	SOX2 regulated genes in Wounding from PMID: 30772301	SOX2
PITPNB	SOX2 regulated genes in Wounding from PMID: 30772301	SOX2
APOL3	SOX2 regulated genes in Wounding from PMID: 30772301	SOX2
APOL4	SOX2 regulated genes in Wounding from PMID: 30772301	SOX2
APOL2	SOX2 regulated genes in Wounding from PMID: 30772301	SOX2
APOL1	SOX2 regulated genes in Wounding from PMID: 30772301	SOX2
RCOR2	SOX2 regulated genes in Wounding from PMID: 30772301	SOX2
ARPP19	SOX2 regulated genes in Wounding from PMID: 30772301	SOX2
ECHDC2	SOX2 regulated genes in Wounding from PMID: 30772301	SOX2
HAPLN4	SOX2 regulated genes in Wounding from PMID: 30772301	SOX2
PPIF	SOX2 regulated genes in Wounding from PMID: 30772301	SOX2
SRBD1	SOX2 regulated genes in Wounding from PMID: 30772301	SOX2
LRRC42	SOX2 regulated genes in Wounding from PMID: 30772301	SOX2
ESM1	SOX2 regulated genes in Wounding from PMID: 30772301	SOX2
PITRM1	SOX2 regulated genes in Wounding from PMID: 30772301	SOX2
METAP1D	SOX2 regulated genes in Wounding from PMID: 30772301	SOX2
MAP2K3	SOX2 regulated genes in Wounding from PMID: 30772301	SOX2
RFXANK	SOX2 regulated genes in Wounding from PMID: 30772301	SOX2
TP53	SOX2 regulated genes in Wounding from PMID: 30772301	SOX2
NR2C2AP	SOX2 regulated genes in Wounding from PMID: 30772301	SOX2
AGAP1	SOX2 regulated genes in Wounding from PMID: 30772301	SOX2
SNAI3	SOX2 regulated genes in Wounding from PMID: 30772301	SOX2
NCBP2L	SOX2 regulated genes in Wounding from PMID: 30772301	SOX2
SYCE1L	SOX2 regulated genes in Wounding from PMID: 30772301	SOX2
TENM2	SOX2 regulated genes in Wounding from PMID: 30772301	SOX2
DYNC1LI1	SOX2 regulated genes in Wounding from PMID: 30772301	SOX2
HOXC13	SOX2 regulated genes in Wounding from PMID: 30772301	SOX2

LEF1	SOX2 regulated genes in Wounding from PMID: 30772301	SOX2
STAM2	SOX2 regulated genes in Wounding from PMID: 30772301	SOX2
EPB41L5	SOX2 regulated genes in Wounding from PMID: 30772301	SOX2
METTL1	SOX2 regulated genes in Wounding from PMID: 30772301	SOX2
MYZAP	SOX2 regulated genes in Wounding from PMID: 30772301	SOX2
LMO4	SOX2 regulated genes in Wounding from PMID: 30772301	SOX2
MSRB3	SOX2 regulated genes in Wounding from PMID: 30772301	SOX2
DMBT1	SOX2 regulated genes in Wounding from PMID: 30772301	SOX2
UPK1B	SOX2 regulated genes in Wounding from PMID: 30772301	SOX2
MYRF	SOX2 regulated genes in Wounding from PMID: 30772301	SOX2
FASTKD5	SOX2 regulated genes in Wounding from PMID: 30772301	SOX2
PROM2	SOX2 regulated genes in Wounding from PMID: 30772301	SOX2
FRMPD1	SOX2 regulated genes in Wounding from PMID: 30772301	SOX2
KANK1	SOX2 regulated genes in Wounding from PMID: 30772301	SOX2
RHCG	SOX2 regulated genes in Wounding from PMID: 30772301	SOX2
GRPEL2	SOX2 regulated genes in Wounding from PMID: 30772301	SOX2
MXI1	SOX2 regulated genes in Wounding from PMID: 30772301	SOX2
SGTB	SOX2 regulated genes in Wounding from PMID: 30772301	SOX2
WNK4	SOX2 regulated genes in Wounding from PMID: 30772301	SOX2
MUS81	SOX2 regulated genes in Wounding from PMID: 30772301	SOX2
EIF3J	SOX2 regulated genes in Wounding from PMID: 30772301	SOX2
VCL	SOX2 regulated genes in Wounding from PMID: 30772301	SOX2
HOXB6	SOX2 regulated genes in Wounding from PMID: 30772301	SOX2
KMT5B	SOX2 regulated genes in Wounding from PMID: 30772301	SOX2
SRPRA	SOX2 regulated genes in Wounding from PMID: 30772301	SOX2
RBM18	SOX2 regulated genes in Wounding from PMID: 30772301	SOX2
HSPA5	SOX2 regulated genes in Wounding from PMID: 30772301	SOX2
USO1	SOX2 regulated genes in Wounding from PMID: 30772301	SOX2
PHTF2	SOX2 regulated genes in Wounding from PMID: 30772301	SOX2
SERPINA11	SOX2 regulated genes in Wounding from PMID: 30772301	SOX2
PDCL3	SOX2 regulated genes in Wounding from PMID: 30772301	SOX2
PCYOX1L	SOX2 regulated genes in Wounding from PMID: 30772301	SOX2
SLCO5A1	SOX2 regulated genes in Wounding from PMID: 30772301	SOX2
SLC27A1	SOX2 regulated genes in Wounding from PMID: 30772301	SOX2
AK4	SOX2 regulated genes in Wounding from PMID: 30772301	SOX2
IL1B	SOX2 regulated genes in Wounding from PMID: 30772301	SOX2
MPZL2	SOX2 regulated genes in Wounding from PMID: 30772301	SOX2
MAS1	SOX2 regulated genes in Wounding from PMID: 30772301	SOX2
TNFRSF11B	SOX2 regulated genes in Wounding from PMID: 30772301	SOX2
TECTA	SOX2 regulated genes in Wounding from PMID: 30772301	SOX2
CAPN1	SOX2 regulated genes in Wounding from PMID: 30772301	SOX2
CNKSR3	SOX2 regulated genes in Wounding from PMID: 30772301	SOX2
UBE2H	SOX2 regulated genes in Wounding from PMID: 30772301	SOX2
SIAH1	SOX2 regulated genes in Wounding from PMID: 30772301	SOX2
TRIM2	SOX2 regulated genes in Wounding from PMID: 30772301	SOX2
CD164L2	SOX2 regulated genes in Wounding from PMID: 30772301	SOX2
ATP1B3	SOX2 regulated genes in Wounding from PMID: 30772301	SOX2
SEC24C	SOX2 regulated genes in Wounding from PMID: 30772301	SOX2
CTTN	SOX2 regulated genes in Wounding from PMID: 30772301	SOX2
IFFO2	SOX2 regulated genes in Wounding from PMID: 30772301	SOX2
GDPD3	SOX2 regulated genes in Wounding from PMID: 30772301	SOX2
PRICKLE2	SOX2 regulated genes in Wounding from PMID: 30772301	SOX2
GCSH	SOX2 regulated genes in Wounding from PMID: 30772301	SOX2
COQ5	SOX2 regulated genes in Wounding from PMID: 30772301	SOX2
OAF	SOX2 regulated genes in Wounding from PMID: 30772301	SOX2
PWWP2B	SOX2 regulated genes in Wounding from PMID: 30772301	SOX2
HADHB	SOX2 regulated genes in Wounding from PMID: 30772301	SOX2
PLEKHH1	SOX2 regulated genes in Wounding from PMID: 30772301	SOX2
ELL2	SOX2 regulated genes in Wounding from PMID: 30772301	SOX2

PSORS1C2	SOX2 regulated genes in Wounding from PMID: 30772301	SOX2
ABHD17C	SOX2 regulated genes in Wounding from PMID: 30772301	SOX2
ELF5	SOX2 regulated genes in Wounding from PMID: 30772301	SOX2
TM4SF1	SOX2 regulated genes in Wounding from PMID: 30772301	SOX2
EIF3L	SOX2 regulated genes in Wounding from PMID: 30772301	SOX2
EIF3D	SOX2 regulated genes in Wounding from PMID: 30772301	SOX2
C10orf95	SOX2 regulated genes in Wounding from PMID: 30772301	SOX2
LYPD5	SOX2 regulated genes in Wounding from PMID: 30772301	SOX2
C11orf80	SOX2 regulated genes in Wounding from PMID: 30772301	SOX2
MAPK3	SOX2 regulated genes in Wounding from PMID: 30772301	SOX2
PTK7	SOX2 regulated genes in Wounding from PMID: 30772301	SOX2
INHBA	SOX2 regulated genes in Wounding from PMID: 30772301	SOX2
SNIP1	SOX2 regulated genes in Wounding from PMID: 30772301	SOX2
EIF5AL1	SOX2 regulated genes in Wounding from PMID: 30772301	SOX2
RAB21	SOX2 regulated genes in Wounding from PMID: 30772301	SOX2
ACTN1	SOX2 regulated genes in Wounding from PMID: 30772301	SOX2
NIPAL1	SOX2 regulated genes in Wounding from PMID: 30772301	SOX2
ICMT	SOX2 regulated genes in Wounding from PMID: 30772301	SOX2
SARDH	SOX2 regulated genes in Wounding from PMID: 30772301	SOX2
RDH10	SOX2 regulated genes in Wounding from PMID: 30772301	SOX2
KIF26A	SOX2 regulated genes in Wounding from PMID: 30772301	SOX2
RAB15	SOX2 regulated genes in Wounding from PMID: 30772301	SOX2
LINC02210-CRHR	SOX2 regulated genes in Wounding from PMID: 30772301	SOX2
TCP1	SOX2 regulated genes in Wounding from PMID: 30772301	SOX2
SOX9	SOX2 regulated genes in Wounding from PMID: 30772301	SOX2
FGFR3	SOX2 regulated genes in Wounding from PMID: 30772301	SOX2
ANXA8	SOX2 regulated genes in Wounding from PMID: 30772301	SOX2
ANXA8L1	SOX2 regulated genes in Wounding from PMID: 30772301	SOX2
PTPRK	SOX2 regulated genes in Wounding from PMID: 30772301	SOX2
SEC11A	SOX2 regulated genes in Wounding from PMID: 30772301	SOX2
PTGR1	SOX2 regulated genes in Wounding from PMID: 30772301	SOX2
CNTFR	SOX2 regulated genes in Wounding from PMID: 30772301	SOX2
ACTB	SOX2 regulated genes in Wounding from PMID: 30772301	SOX2
TMEM39A	SOX2 regulated genes in Wounding from PMID: 30772301	SOX2
SFRP1	SOX2 regulated genes in Wounding from PMID: 30772301	SOX2
EPHB6	SOX2 regulated genes in Wounding from PMID: 30772301	SOX2
PIGH	SOX2 regulated genes in Wounding from PMID: 30772301	SOX2
ZFAND6	SOX2 regulated genes in Wounding from PMID: 30772301	SOX2
NECTIN3	SOX2 regulated genes in Wounding from PMID: 30772301	SOX2
DOK4	SOX2 regulated genes in Wounding from PMID: 30772301	SOX2
NRARP	SOX2 regulated genes in Wounding from PMID: 30772301	SOX2
NTN4	SOX2 regulated genes in Wounding from PMID: 30772301	SOX2
TSFM	SOX2 regulated genes in Wounding from PMID: 30772301	SOX2
CXXC5	SOX2 regulated genes in Wounding from PMID: 30772301	SOX2
COPB1	SOX2 regulated genes in Wounding from PMID: 30772301	SOX2
SCNN1B	SOX2 regulated genes in Wounding from PMID: 30772301	SOX2
FAF2	SOX2 regulated genes in Wounding from PMID: 30772301	SOX2
ACTR1A	SOX2 regulated genes in Wounding from PMID: 30772301	SOX2
CLCF1	SOX2 regulated genes in Wounding from PMID: 30772301	SOX2
CPEB2	SOX2 regulated genes in Wounding from PMID: 30772301	SOX2
MAL	SOX2 regulated genes in Wounding from PMID: 30772301	SOX2
RALA	SOX2 regulated genes in Wounding from PMID: 30772301	SOX2
MOB1B	SOX2 regulated genes in Wounding from PMID: 30772301	SOX2
UBA1	SOX2 regulated genes in Wounding from PMID: 30772301	SOX2
EVA1A	SOX2 regulated genes in Wounding from PMID: 30772301	SOX2
VGLL3	SOX2 regulated genes in Wounding from PMID: 30772301	SOX2
LBH	SOX2 regulated genes in Wounding from PMID: 30772301	SOX2
C6orf132	SOX2 regulated genes in Wounding from PMID: 30772301	SOX2
CAND1	SOX2 regulated genes in Wounding from PMID: 30772301	SOX2

NUP35	SOX2 regulated genes in Wounding from PMID: 30772301	SOX2
SNRPA	SOX2 regulated genes in Wounding from PMID: 30772301	SOX2
FOSL1	SOX2 regulated genes in Wounding from PMID: 30772301	SOX2
PLPP5	SOX2 regulated genes in Wounding from PMID: 30772301	SOX2
DLX4	SOX2 regulated genes in Wounding from PMID: 30772301	SOX2
BRAP	SOX2 regulated genes in Wounding from PMID: 30772301	SOX2
HS6ST1	SOX2 regulated genes in Wounding from PMID: 30772301	SOX2
GPC4	SOX2 regulated genes in Wounding from PMID: 30772301	SOX2
DPYSL5	SOX2 regulated genes in Wounding from PMID: 30772301	SOX2
PPFIA3	SOX2 regulated genes in Wounding from PMID: 30772301	SOX2
MMP1	SOX2 regulated genes in Wounding from PMID: 30772301	SOX2
ATP6V1A	SOX2 regulated genes in Wounding from PMID: 30772301	SOX2
SMNDC1	SOX2 regulated genes in Wounding from PMID: 30772301	SOX2
TMEM121	SOX2 regulated genes in Wounding from PMID: 30772301	SOX2
GJB4	SOX2 regulated genes in Wounding from PMID: 30772301	SOX2
HNRNPAB	SOX2 regulated genes in Wounding from PMID: 30772301	SOX2
BSPRY	SOX2 regulated genes in Wounding from PMID: 30772301	SOX2
BRD1	SOX2 regulated genes in Wounding from PMID: 30772301	SOX2
SUFU	SOX2 regulated genes in Wounding from PMID: 30772301	SOX2
TLE2	SOX2 regulated genes in Wounding from PMID: 30772301	SOX2
ARHGAP39	SOX2 regulated genes in Wounding from PMID: 30772301	SOX2
EFNA2	SOX2 regulated genes in Wounding from PMID: 30772301	SOX2
ZNF821	SOX2 regulated genes in Wounding from PMID: 30772301	SOX2
FGF19	SOX2 regulated genes in Wounding from PMID: 30772301	SOX2
IL11RA	SOX2 regulated genes in Wounding from PMID: 30772301	SOX2
MYADM	SOX2 regulated genes in Wounding from PMID: 30772301	SOX2
UPF1	SOX2 regulated genes in Wounding from PMID: 30772301	SOX2
PLCD3	SOX2 regulated genes in Wounding from PMID: 30772301	SOX2
PRDX2	SOX2 regulated genes in Wounding from PMID: 30772301	SOX2
ATP2B4	SOX2 regulated genes in Wounding from PMID: 30772301	SOX2
MRTO4	SOX2 regulated genes in Wounding from PMID: 30772301	SOX2
UNC119B	SOX2 regulated genes in Wounding from PMID: 30772301	SOX2
PLA2G2F	SOX2 regulated genes in Wounding from PMID: 30772301	SOX2
ODC1	SOX2 regulated genes in Wounding from PMID: 30772301	SOX2
FLNB	SOX2 regulated genes in Wounding from PMID: 30772301	SOX2
SPTSSB	SOX2 regulated genes in Wounding from PMID: 30772301	SOX2
MAP6D1	SOX2 regulated genes in Wounding from PMID: 30772301	SOX2
YOD1	SOX2 regulated genes in Wounding from PMID: 30772301	SOX2
PDIA4	SOX2 regulated genes in Wounding from PMID: 30772301	SOX2
GNG4	SOX2 regulated genes in Wounding from PMID: 30772301	SOX2
MTA2	SOX2 regulated genes in Wounding from PMID: 30772301	SOX2
B3GNT3	SOX2 regulated genes in Wounding from PMID: 30772301	SOX2
KRT17	SOX2 regulated genes in Wounding from PMID: 30772301	SOX2
DDB1	SOX2 regulated genes in Wounding from PMID: 30772301	SOX2
CGN	SOX2 regulated genes in Wounding from PMID: 30772301	SOX2
CD44	SOX2 regulated genes in Wounding from PMID: 30772301	SOX2
CHTF8	SOX2 regulated genes in Wounding from PMID: 30772301	SOX2
VAMP8	SOX2 regulated genes in Wounding from PMID: 30772301	SOX2
POR	SOX2 regulated genes in Wounding from PMID: 30772301	SOX2
ARHGAP21	SOX2 regulated genes in Wounding from PMID: 30772301	SOX2
SOX13	SOX2 regulated genes in Wounding from PMID: 30772301	SOX2
COBLL1	SOX2 regulated genes in Wounding from PMID: 30772301	SOX2
ARL15	SOX2 regulated genes in Wounding from PMID: 30772301	SOX2
CCSER1	SOX2 regulated genes in Wounding from PMID: 30772301	SOX2
CHDH	SOX2 regulated genes in Wounding from PMID: 30772301	SOX2
PAFAH1B1	SOX2 regulated genes in Wounding from PMID: 30772301	SOX2
SNTB2	SOX2 regulated genes in Wounding from PMID: 30772301	SOX2
RNF40	SOX2 regulated genes in Wounding from PMID: 30772301	SOX2
SS18L2	SOX2 regulated genes in Wounding from PMID: 30772301	SOX2

KLK10	SOX2 regulated genes in Wounding from PMID: 30772301	SOX2
CYB561D1	SOX2 regulated genes in Wounding from PMID: 30772301	SOX2
MYO18A	SOX2 regulated genes in Wounding from PMID: 30772301	SOX2
CDH3	SOX2 regulated genes in Wounding from PMID: 30772301	SOX2
HNRNPM	SOX2 regulated genes in Wounding from PMID: 30772301	SOX2
ZNF608	SOX2 regulated genes in Wounding from PMID: 30772301	SOX2
ATG4B	SOX2 regulated genes in Wounding from PMID: 30772301	SOX2
CDK18	SOX2 regulated genes in Wounding from PMID: 30772301	SOX2
HDAC5	SOX2 regulated genes in Wounding from PMID: 30772301	SOX2
SNX7	SOX2 regulated genes in Wounding from PMID: 30772301	SOX2
METTL16	SOX2 regulated genes in Wounding from PMID: 30772301	SOX2
GBP6	SOX2 regulated genes in Wounding from PMID: 30772301	SOX2
KPNA4	SOX2 regulated genes in Wounding from PMID: 30772301	SOX2
CSNK1A1	SOX2 regulated genes in Wounding from PMID: 30772301	SOX2
BLZF1	SOX2 regulated genes in Wounding from PMID: 30772301	SOX2
PADI1	SOX2 regulated genes in Wounding from PMID: 30772301	SOX2
TLN2	SOX2 regulated genes in Wounding from PMID: 30772301	SOX2
KRTAP2-3	SOX2 regulated genes in Wounding from PMID: 30772301	SOX2
KRTAP4-11	SOX2 regulated genes in Wounding from PMID: 30772301	SOX2
KRTAP4-8	SOX2 regulated genes in Wounding from PMID: 30772301	SOX2
CAPN12	SOX2 regulated genes in Wounding from PMID: 30772301	SOX2
SPAG9	SOX2 regulated genes in Wounding from PMID: 30772301	SOX2
TJP1	SOX2 regulated genes in Wounding from PMID: 30772301	SOX2
C1orf52	SOX2 regulated genes in Wounding from PMID: 30772301	SOX2
APLP2	SOX2 regulated genes in Wounding from PMID: 30772301	SOX2
PTPN14	SOX2 regulated genes in Wounding from PMID: 30772301	SOX2
ACBD3	SOX2 regulated genes in Wounding from PMID: 30772301	SOX2
PDE4B	SOX2 regulated genes in Wounding from PMID: 30772301	SOX2
PSPN	SOX2 regulated genes in Wounding from PMID: 30772301	SOX2
HYOU1	SOX2 regulated genes in Wounding from PMID: 30772301	SOX2
CREB3L4	SOX2 regulated genes in Wounding from PMID: 30772301	SOX2
EDN2	SOX2 regulated genes in Wounding from PMID: 30772301	SOX2
CHFR	SOX2 regulated genes in Wounding from PMID: 30772301	SOX2
WDR12	SOX2 regulated genes in Wounding from PMID: 30772301	SOX2
SLC40A1	SOX2 regulated genes in Wounding from PMID: 30772301	SOX2
MICA	SOX2 regulated genes in Wounding from PMID: 30772301	SOX2
CDV3	SOX2 regulated genes in Wounding from PMID: 30772301	SOX2
FZD7	SOX2 regulated genes in Wounding from PMID: 30772301	SOX2
CALR	SOX2 regulated genes in Wounding from PMID: 30772301	SOX2
SPRR3	SOX2 regulated genes in Wounding from PMID: 30772301	SOX2
TRAPPC4	SOX2 regulated genes in Wounding from PMID: 30772301	SOX2
CLEC2D	SOX2 regulated genes in Wounding from PMID: 30772301	SOX2
DENND2C	SOX2 regulated genes in Wounding from PMID: 30772301	SOX2
FXYD4	SOX2 regulated genes in Wounding from PMID: 30772301	SOX2
MAST4	SOX2 regulated genes in Wounding from PMID: 30772301	SOX2
MEN1	SOX2 regulated genes in Wounding from PMID: 30772301	SOX2
PRDM4	SOX2 regulated genes in Wounding from PMID: 30772301	SOX2
BAHD1	SOX2 regulated genes in Wounding from PMID: 30772301	SOX2
UBQLN4	SOX2 regulated genes in Wounding from PMID: 30772301	SOX2
CELSR2	SOX2 regulated genes in Wounding from PMID: 30772301	SOX2
TSEN2	SOX2 regulated genes in Wounding from PMID: 30772301	SOX2
PRPF31	SOX2 regulated genes in Wounding from PMID: 30772301	SOX2
EPS8L2	SOX2 regulated genes in Wounding from PMID: 30772301	SOX2
IGSF9	SOX2 regulated genes in Wounding from PMID: 30772301	SOX2
SLC16A6	SOX2 regulated genes in Wounding from PMID: 30772301	SOX2
GTF2B	SOX2 regulated genes in Wounding from PMID: 30772301	SOX2
SH3TC2	SOX2 regulated genes in Wounding from PMID: 30772301	SOX2
EPHA1	SOX2 regulated genes in Wounding from PMID: 30772301	SOX2
MLF1	SOX2 regulated genes in Wounding from PMID: 30772301	SOX2

RUVBL2	SOX2 regulated genes in Wounding from PMID: 30772301	SOX2
MAP7	SOX2 regulated genes in Wounding from PMID: 30772301	SOX2
MYO19	SOX2 regulated genes in Wounding from PMID: 30772301	SOX2
SF1	SOX2 regulated genes in Wounding from PMID: 30772301	SOX2
CTTNBP2	SOX2 regulated genes in Wounding from PMID: 30772301	SOX2
KDF1	SOX2 regulated genes in Wounding from PMID: 30772301	SOX2
PPL	SOX2 regulated genes in Wounding from PMID: 30772301	SOX2
EFHD1	SOX2 regulated genes in Wounding from PMID: 30772301	SOX2
GSTM3	SOX2 regulated genes in Wounding from PMID: 30772301	SOX2
TIAM2	SOX2 regulated genes in Wounding from PMID: 30772301	SOX2
CMPK1	SOX2 regulated genes in Wounding from PMID: 30772301	SOX2
KRTAP1-5	SOX2 regulated genes in Wounding from PMID: 30772301	SOX2
KRTAP1-3	SOX2 regulated genes in Wounding from PMID: 30772301	SOX2
KRTAP1-1	SOX2 regulated genes in Wounding from PMID: 30772301	SOX2
DNAJA4	SOX2 regulated genes in Wounding from PMID: 30772301	SOX2
ERC2	SOX2 regulated genes in Wounding from PMID: 30772301	SOX2
PPFIBP2	SOX2 regulated genes in Wounding from PMID: 30772301	SOX2
INTS4	SOX2 regulated genes in Wounding from PMID: 30772301	SOX2
S100A6	SOX2 regulated genes in Wounding from PMID: 30772301	SOX2
MOB4	SOX2 regulated genes in Wounding from PMID: 30772301	SOX2
KLC1	SOX2 regulated genes in Wounding from PMID: 30772301	SOX2
LSR	SOX2 regulated genes in Wounding from PMID: 30772301	SOX2
CAPN8	SOX2 regulated genes in Wounding from PMID: 30772301	SOX2
NHSL1	SOX2 regulated genes in Wounding from PMID: 30772301	SOX2
HES2	SOX2 regulated genes in Wounding from PMID: 30772301	SOX2
S100A16	SOX2 regulated genes in Wounding from PMID: 30772301	SOX2
PUM1	SOX2 regulated genes in Wounding from PMID: 30772301	SOX2
PLXNA2	SOX2 regulated genes in Wounding from PMID: 30772301	SOX2
PKD1L3	SOX2 regulated genes in Wounding from PMID: 30772301	SOX2
UBE2G1	SOX2 regulated genes in Wounding from PMID: 30772301	SOX2
NDEL1	SOX2 regulated genes in Wounding from PMID: 30772301	SOX2
SGMS2	SOX2 regulated genes in Wounding from PMID: 30772301	SOX2
IQGAP1	SOX2 regulated genes in Wounding from PMID: 30772301	SOX2
NRDC	SOX2 regulated genes in Wounding from PMID: 30772301	SOX2
CSGALNACT1	SOX2 regulated genes in Wounding from PMID: 30772301	SOX2
LRRC8E	SOX2 regulated genes in Wounding from PMID: 30772301	SOX2
GMCL1	SOX2 regulated genes in Wounding from PMID: 30772301	SOX2
WNT11	SOX2 regulated genes in Wounding from PMID: 30772301	SOX2
GALNT3	SOX2 regulated genes in Wounding from PMID: 30772301	SOX2
PDLIM7	SOX2 regulated genes in Wounding from PMID: 30772301	SOX2
WDR26	SOX2 regulated genes in Wounding from PMID: 30772301	SOX2
OCLN	SOX2 regulated genes in Wounding from PMID: 30772301	SOX2
FARP2	SOX2 regulated genes in Wounding from PMID: 30772301	SOX2
RGS12	SOX2 regulated genes in Wounding from PMID: 30772301	SOX2
TM4SF4	SOX2 regulated genes in Wounding from PMID: 30772301	SOX2
CLIP1	SOX2 regulated genes in Wounding from PMID: 30772301	SOX2
ADGRL3	SOX2 regulated genes in Wounding from PMID: 30772301	SOX2
KRT34	SOX2 regulated genes in Wounding from PMID: 30772301	SOX2
NKAPL	SOX2 regulated genes in Wounding from PMID: 30772301	SOX2
BHLHE40	SOX2 regulated genes in Wounding from PMID: 30772301	SOX2
NIPAL3	SOX2 regulated genes in Wounding from PMID: 30772301	SOX2
KALRN	SOX2 regulated genes in Wounding from PMID: 30772301	SOX2
ETV2	SOX2 regulated genes in Wounding from PMID: 30772301	SOX2
B3GALNT2	SOX2 regulated genes in Wounding from PMID: 30772301	SOX2
GTF2I	SOX2 regulated genes in Wounding from PMID: 30772301	SOX2
HNRNPC	SOX2 regulated genes in Wounding from PMID: 30772301	SOX2
HNRNPCL1	SOX2 regulated genes in Wounding from PMID: 30772301	SOX2
HNRNPCL2	SOX2 regulated genes in Wounding from PMID: 30772301	SOX2
LRIG1	SOX2 regulated genes in Wounding from PMID: 30772301	SOX2

TRIM33	SOX2 regulated genes in Wounding from PMID: 30772301	SOX2
SPNS2	SOX2 regulated genes in Wounding from PMID: 30772301	SOX2
HAS3	SOX2 regulated genes in Wounding from PMID: 30772301	SOX2
INSIG2	SOX2 regulated genes in Wounding from PMID: 30772301	SOX2
BNIPL	SOX2 regulated genes in Wounding from PMID: 30772301	SOX2
CETN3	SOX2 regulated genes in Wounding from PMID: 30772301	SOX2
TRAK2	SOX2 regulated genes in Wounding from PMID: 30772301	SOX2
MAST1	SOX2 regulated genes in Wounding from PMID: 30772301	SOX2
NECTIN4	SOX2 regulated genes in Wounding from PMID: 30772301	SOX2
ACTN4	SOX2 regulated genes in Wounding from PMID: 30772301	SOX2
TNFAIP6	SOX2 regulated genes in Wounding from PMID: 30772301	SOX2
TMBIM1	SOX2 regulated genes in Wounding from PMID: 30772301	SOX2
CRNN	SOX2 regulated genes in Wounding from PMID: 30772301	SOX2
SMIM5	SOX2 regulated genes in Wounding from PMID: 30772301	SOX2
SIRT6	SOX2 regulated genes in Wounding from PMID: 30772301	SOX2
FAM167A	SOX2 regulated genes in Wounding from PMID: 30772301	SOX2
DNAJB2	SOX2 regulated genes in Wounding from PMID: 30772301	SOX2
GOLT1B	SOX2 regulated genes in Wounding from PMID: 30772301	SOX2
KLHL18	SOX2 regulated genes in Wounding from PMID: 30772301	SOX2
ZYX	SOX2 regulated genes in Wounding from PMID: 30772301	SOX2
ATXN1	SOX2 regulated genes in Wounding from PMID: 30772301	SOX2
ELAVL1	SOX2 regulated genes in Wounding from PMID: 30772301	SOX2
ACOT1	SOX2 regulated genes in Wounding from PMID: 30772301	SOX2
ACOT2	SOX2 regulated genes in Wounding from PMID: 30772301	SOX2
NCKAP5	SOX2 regulated genes in Wounding from PMID: 30772301	SOX2
DSP	SOX2 regulated genes in Wounding from PMID: 30772301	SOX2
PFDN1	SOX2 regulated genes in Wounding from PMID: 30772301	SOX2
TET3	SOX2 regulated genes in Wounding from PMID: 30772301	SOX2
KRT32	SOX2 regulated genes in Wounding from PMID: 30772301	SOX2
TMEM120B	SOX2 regulated genes in Wounding from PMID: 30772301	SOX2
CDK2AP1	SOX2 regulated genes in Wounding from PMID: 30772301	SOX2
PRR7	SOX2 regulated genes in Wounding from PMID: 30772301	SOX2
TMEM184A	SOX2 regulated genes in Wounding from PMID: 30772301	SOX2
SMUG1	SOX2 regulated genes in Wounding from PMID: 30772301	SOX2
RAP1GAP	SOX2 regulated genes in Wounding from PMID: 30772301	SOX2
FURIN	SOX2 regulated genes in Wounding from PMID: 30772301	SOX2
PTPN11	SOX2 regulated genes in Wounding from PMID: 30772301	SOX2
EIF2B3	SOX2 regulated genes in Wounding from PMID: 30772301	SOX2
AKAP9	SOX2 regulated genes in Wounding from PMID: 30772301	SOX2
SERTAD4	SOX2 regulated genes in Wounding from PMID: 30772301	SOX2
SNRPD2	SOX2 regulated genes in Wounding from PMID: 30772301	SOX2
SMIM7	SOX2 regulated genes in Wounding from PMID: 30772301	SOX2
MYL6	SOX2 regulated genes in Wounding from PMID: 30772301	SOX2
TLX1	SOX2 regulated genes in Wounding from PMID: 30772301	SOX2
TPPP	SOX2 regulated genes in Wounding from PMID: 30772301	SOX2
KRTCAP3	SOX2 regulated genes in Wounding from PMID: 30772301	SOX2
TBCD	SOX2 regulated genes in Wounding from PMID: 30772301	SOX2
PIGM	SOX2 regulated genes in Wounding from PMID: 30772301	SOX2
FZD3	SOX2 regulated genes in Wounding from PMID: 30772301	SOX2
RNF222	SOX2 regulated genes in Wounding from PMID: 30772301	SOX2
LINGO1	SOX2 regulated genes in Wounding from PMID: 30772301	SOX2
SPACA6	SOX2 regulated genes in Wounding from PMID: 30772301	SOX2
LMNB2	SOX2 regulated genes in Wounding from PMID: 30772301	SOX2
NAPEPLD	SOX2 regulated genes in Wounding from PMID: 30772301	SOX2
ABCF3	SOX2 regulated genes in Wounding from PMID: 30772301	SOX2
ALG8	SOX2 regulated genes in Wounding from PMID: 30772301	SOX2
TMED2	SOX2 regulated genes in Wounding from PMID: 30772301	SOX2
HES1	SOX2 regulated genes in Wounding from PMID: 30772301	SOX2
SFPQ	SOX2 regulated genes in Wounding from PMID: 30772301	SOX2

MARVELD3	SOX2 regulated genes in Wounding from PMID: 30772301	SOX2
PYROXD1	SOX2 regulated genes in Wounding from PMID: 30772301	SOX2
NUAK1	SOX2 regulated genes in Wounding from PMID: 30772301	SOX2
LTBP4	SOX2 regulated genes in Wounding from PMID: 30772301	SOX2
VANGL2	SOX2 regulated genes in Wounding from PMID: 30772301	SOX2
EFNA3	SOX2 regulated genes in Wounding from PMID: 30772301	SOX2
JMJD4	SOX2 regulated genes in Wounding from PMID: 30772301	SOX2
CALM1	SOX2 regulated genes in Wounding from PMID: 30772301	SOX2
PRKCZ	SOX2 regulated genes in Wounding from PMID: 30772301	SOX2
DDR1	SOX2 regulated genes in Wounding from PMID: 30772301	SOX2
SNRPA1	SOX2 regulated genes in Wounding from PMID: 30772301	SOX2
ARHGEF28	SOX2 regulated genes in Wounding from PMID: 30772301	SOX2
PHF23	SOX2 regulated genes in Wounding from PMID: 30772301	SOX2
SLC2A1	SOX2 regulated genes in Wounding from PMID: 30772301	SOX2
CNTNAP1	SOX2 regulated genes in Wounding from PMID: 30772301	SOX2
VDR	SOX2 regulated genes in Wounding from PMID: 30772301	SOX2
LMNA	SOX2 regulated genes in Wounding from PMID: 30772301	SOX2
GPRC5D	SOX2 regulated genes in Wounding from PMID: 30772301	SOX2
COPB2	SOX2 regulated genes in Wounding from PMID: 30772301	SOX2
EXOC7	SOX2 regulated genes in Wounding from PMID: 30772301	SOX2
GAS8	SOX2 regulated genes in Wounding from PMID: 30772301	SOX2
GLI2	SOX2 regulated genes in Wounding from PMID: 30772301	SOX2
AFDN	SOX2 regulated genes in Wounding from PMID: 30772301	SOX2
THEM4	SOX2 regulated genes in Wounding from PMID: 30772301	SOX2
ST14	SOX2 regulated genes in Wounding from PMID: 30772301	SOX2
RRM2	SOX2 regulated genes in Wounding from PMID: 30772301	SOX2
RABL2A	SOX2 regulated genes in Wounding from PMID: 30772301	SOX2
PPM1G	SOX2 regulated genes in Wounding from PMID: 30772301	SOX2
KIF21A	SOX2 regulated genes in Wounding from PMID: 30772301	SOX2
NPM2	SOX2 regulated genes in Wounding from PMID: 30772301	SOX2
EIF2S3B	SOX2 regulated genes in Wounding from PMID: 30772301	SOX2
KRT8	SOX2 regulated genes in Wounding from PMID: 30772301	SOX2
NKD2	SOX2 regulated genes in Wounding from PMID: 30772301	SOX2
NUDT21	SOX2 regulated genes in Wounding from PMID: 30772301	SOX2
SYT13	SOX2 regulated genes in Wounding from PMID: 30772301	SOX2
TNN	SOX2 regulated genes in Wounding from PMID: 30772301	SOX2
XXYLT1	SOX2 regulated genes in Wounding from PMID: 30772301	SOX2
LMTK3	SOX2 regulated genes in Wounding from PMID: 30772301	SOX2
THUMPD3	SOX2 regulated genes in Wounding from PMID: 30772301	SOX2
FOXJ1	SOX2 regulated genes in Wounding from PMID: 30772301	SOX2
ENAH	SOX2 regulated genes in Wounding from PMID: 30772301	SOX2
ARHGAP32	SOX2 regulated genes in Wounding from PMID: 30772301	SOX2
CAPN2	SOX2 regulated genes in Wounding from PMID: 30772301	SOX2
TMEM54	SOX2 regulated genes in Wounding from PMID: 30772301	SOX2
VPS45	SOX2 regulated genes in Wounding from PMID: 30772301	SOX2
FAM8A1	SOX2 regulated genes in Wounding from PMID: 30772301	SOX2
TMEM231	SOX2 regulated genes in Wounding from PMID: 30772301	SOX2
S100A14	SOX2 regulated genes in Wounding from PMID: 30772301	SOX2
ARHGEF5	SOX2 regulated genes in Wounding from PMID: 30772301	SOX2
PPP1R2	SOX2 regulated genes in Wounding from PMID: 30772301	SOX2
PPP1R2B	SOX2 regulated genes in Wounding from PMID: 30772301	SOX2
RAB3IP	SOX2 regulated genes in Wounding from PMID: 30772301	SOX2
TAX1BP1	SOX2 regulated genes in Wounding from PMID: 30772301	SOX2
PM20D1	SOX2 regulated genes in Wounding from PMID: 30772301	SOX2
EMP1	SOX2 regulated genes in Wounding from PMID: 30772301	SOX2
FZD10	SOX2 regulated genes in Wounding from PMID: 30772301	SOX2
UBE2O	SOX2 regulated genes in Wounding from PMID: 30772301	SOX2
FAM98A	SOX2 regulated genes in Wounding from PMID: 30772301	SOX2
CNN3	SOX2 regulated genes in Wounding from PMID: 30772301	SOX2

STK16	SOX2 regulated genes in Wounding from PMID: 30772301	SOX2
FBRS	SOX2 regulated genes in Wounding from PMID: 30772301	SOX2
TCERG1	SOX2 regulated genes in Wounding from PMID: 30772301	SOX2
FAM136A	SOX2 regulated genes in Wounding from PMID: 30772301	SOX2
ANP32E	SOX2 regulated genes in Wounding from PMID: 30772301	SOX2
NEMF	SOX2 regulated genes in Wounding from PMID: 30772301	SOX2
ZNF326	SOX2 regulated genes in Wounding from PMID: 30772301	SOX2
MTUS1	SOX2 regulated genes in Wounding from PMID: 30772301	SOX2
ADGRG1	SOX2 regulated genes in Wounding from PMID: 30772301	SOX2
NBEAL2	SOX2 regulated genes in Wounding from PMID: 30772301	SOX2
SIPA1L2	SOX2 regulated genes in Wounding from PMID: 30772301	SOX2
LLGL1	SOX2 regulated genes in Wounding from PMID: 30772301	SOX2
SPG7	SOX2 regulated genes in Wounding from PMID: 30772301	SOX2
MTX2	SOX2 regulated genes in Wounding from PMID: 30772301	SOX2
SHKBP1	SOX2 regulated genes in Wounding from PMID: 30772301	SOX2
FNBP1L	SOX2 regulated genes in Wounding from PMID: 30772301	SOX2
BAZ1A	SOX2 regulated genes in Wounding from PMID: 30772301	SOX2
KCTD3	SOX2 regulated genes in Wounding from PMID: 30772301	SOX2
SLC6A2	SOX2 regulated genes in Wounding from PMID: 30772301	SOX2
VGLL4	SOX2 regulated genes in Wounding from PMID: 30772301	SOX2
OST4	SOX2 regulated genes in Wounding from PMID: 30772301	SOX2
ARHGAP20	SOX2 regulated genes in Wounding from PMID: 30772301	SOX2
DNAJB1	SOX2 regulated genes in Wounding from PMID: 30772301	SOX2
EPHX4	SOX2 regulated genes in Wounding from PMID: 30772301	SOX2
SIKE1	SOX2 regulated genes in Wounding from PMID: 30772301	SOX2
CD9	SOX2 regulated genes in Wounding from PMID: 30772301	SOX2
DNAJC11	SOX2 regulated genes in Wounding from PMID: 30772301	SOX2
KRT40	SOX2 regulated genes in Wounding from PMID: 30772301	SOX2
CNOT9	SOX2 regulated genes in Wounding from PMID: 30772301	SOX2
MAP4	SOX2 regulated genes in Wounding from PMID: 30772301	SOX2
COG1	SOX2 regulated genes in Wounding from PMID: 30772301	SOX2
PTPN12	SOX2 regulated genes in Wounding from PMID: 30772301	SOX2
IL1F10	SOX2 regulated genes in Wounding from PMID: 30772301	SOX2
MAP7D1	SOX2 regulated genes in Wounding from PMID: 30772301	SOX2
SH3BP5L	SOX2 regulated genes in Wounding from PMID: 30772301	SOX2
RHBDD2	SOX2 regulated genes in Wounding from PMID: 30772301	SOX2
EPHB2	SOX2 regulated genes in Wounding from PMID: 30772301	SOX2
DAPL1	SOX2 regulated genes in Wounding from PMID: 30772301	SOX2
ERBB2	SOX2 regulated genes in Wounding from PMID: 30772301	SOX2
SAMD4B	SOX2 regulated genes in Wounding from PMID: 30772301	SOX2
LPCAT4	SOX2 regulated genes in Wounding from PMID: 30772301	SOX2
CDC14A	SOX2 regulated genes in Wounding from PMID: 30772301	SOX2
WNT9A	SOX2 regulated genes in Wounding from PMID: 30772301	SOX2
GTF2H3	SOX2 regulated genes in Wounding from PMID: 30772301	SOX2
HEATR3	SOX2 regulated genes in Wounding from PMID: 30772301	SOX2
PKP1	SOX2 regulated genes in Wounding from PMID: 30772301	SOX2
ANKRD13C	SOX2 regulated genes in Wounding from PMID: 30772301	SOX2
ZC3H11A	SOX2 regulated genes in Wounding from PMID: 30772301	SOX2
ZC3H11B	SOX2 regulated genes in Wounding from PMID: 30772301	SOX2
LMBRD2	SOX2 regulated genes in Wounding from PMID: 30772301	SOX2
NECAB2	SOX2 regulated genes in Wounding from PMID: 30772301	SOX2
SFN	SOX2 regulated genes in Wounding from PMID: 30772301	SOX2
GRHL1	SOX2 regulated genes in Wounding from PMID: 30772301	SOX2
MYBPC3	SOX2 regulated genes in Wounding from PMID: 30772301	SOX2
AMOTL2	SOX2 regulated genes in Wounding from PMID: 30772301	SOX2
PHLDA1	SOX2 regulated genes in Wounding from PMID: 30772301	SOX2
TRIM39	SOX2 regulated genes in Wounding from PMID: 30772301	SOX2
TUBA1C	SOX2 regulated genes in Wounding from PMID: 30772301	SOX2
EML2	SOX2 regulated genes in Wounding from PMID: 30772301	SOX2

HNRNPH1	SOX2 regulated genes in Wounding from PMID: 30772301	SOX2
TRIM28	SOX2 regulated genes in Wounding from PMID: 30772301	SOX2
KRTAP3-1	SOX2 regulated genes in Wounding from PMID: 30772301	SOX2
KRT23	SOX2 regulated genes in Wounding from PMID: 30772301	SOX2
ERP44	SOX2 regulated genes in Wounding from PMID: 30772301	SOX2
F2RL1	SOX2 regulated genes in Wounding from PMID: 30772301	SOX2
SPRY4	SOX2 regulated genes in Wounding from PMID: 30772301	SOX2
DUOXA1	SOX2 regulated genes in Wounding from PMID: 30772301	SOX2
RAD17	SOX2 regulated genes in Wounding from PMID: 30772301	SOX2
STK11	SOX2 regulated genes in Wounding from PMID: 30772301	SOX2
USP6NL	SOX2 regulated genes in Wounding from PMID: 30772301	SOX2
EPHB1	SOX2 regulated genes in Wounding from PMID: 30772301	SOX2
SNAP47	SOX2 regulated genes in Wounding from PMID: 30772301	SOX2
RBM14	SOX2 regulated genes in Wounding from PMID: 30772301	SOX2
MYT1L	SOX2 regulated genes in Wounding from PMID: 30772301	SOX2
RABIF	SOX2 regulated genes in Wounding from PMID: 30772301	SOX2
CDC42EP4	SOX2 regulated genes in Wounding from PMID: 30772301	SOX2
F2R	SOX2 regulated genes in Wounding from PMID: 30772301	SOX2
CAMSAP3	SOX2 regulated genes in Wounding from PMID: 30772301	SOX2
KPRP	SOX2 regulated genes in Wounding from PMID: 30772301	SOX2
THOC7	SOX2 regulated genes in Wounding from PMID: 30772301	SOX2
TRAPPC3	SOX2 regulated genes in Wounding from PMID: 30772301	SOX2
CDC25A	SOX2 regulated genes in Wounding from PMID: 30772301	SOX2
OLFM2	SOX2 regulated genes in Wounding from PMID: 30772301	SOX2
WSB2	SOX2 regulated genes in Wounding from PMID: 30772301	SOX2
GPATCH4	SOX2 regulated genes in Wounding from PMID: 30772301	SOX2
PLK2	SOX2 regulated genes in Wounding from PMID: 30772301	SOX2
HMGXB3	SOX2 regulated genes in Wounding from PMID: 30772301	SOX2
PCDHB16	SOX2 regulated genes in Wounding from PMID: 30772301	SOX2
EIF4G1	SOX2 regulated genes in Wounding from PMID: 30772301	SOX2
VPS37B	SOX2 regulated genes in Wounding from PMID: 30772301	SOX2
NPPC	SOX2 regulated genes in Wounding from PMID: 30772301	SOX2
TAGLN2	SOX2 regulated genes in Wounding from PMID: 30772301	SOX2
P3H2	SOX2 regulated genes in Wounding from PMID: 30772301	SOX2
NPEPPS	SOX2 regulated genes in Wounding from PMID: 30772301	SOX2
SEC22B	SOX2 regulated genes in Wounding from PMID: 30772301	SOX2
SH3PXD2A	SOX2 regulated genes in Wounding from PMID: 30772301	SOX2
KRT39	SOX2 regulated genes in Wounding from PMID: 30772301	SOX2
ALDH3B2	SOX2 regulated genes in Wounding from PMID: 30772301	SOX2
GRAMD2A	SOX2 regulated genes in Wounding from PMID: 30772301	SOX2
PCBP1	SOX2 regulated genes in Wounding from PMID: 30772301	SOX2
HS2ST1	SOX2 regulated genes in Wounding from PMID: 30772301	SOX2
KRAS	SOX2 regulated genes in Wounding from PMID: 30772301	SOX2
FAM83E	SOX2 regulated genes in Wounding from PMID: 30772301	SOX2
PTHLH	SOX2 regulated genes in Wounding from PMID: 30772301	SOX2
USP31	SOX2 regulated genes in Wounding from PMID: 30772301	SOX2
POLR3D	SOX2 regulated genes in Wounding from PMID: 30772301	SOX2
S100A1	SOX2 regulated genes in Wounding from PMID: 30772301	SOX2
FUT1	SOX2 regulated genes in Wounding from PMID: 30772301	SOX2
NT5E	SOX2 regulated genes in Wounding from PMID: 30772301	SOX2
SGK1	SOX2 regulated genes in Wounding from PMID: 30772301	SOX2
ZNF23	SOX2 regulated genes in Wounding from PMID: 30772301	SOX2
HMCN1	SOX2 regulated genes in Wounding from PMID: 30772301	SOX2
NECTIN2	SOX2 regulated genes in Wounding from PMID: 30772301	SOX2
EPN3	SOX2 regulated genes in Wounding from PMID: 30772301	SOX2
FOXO6	SOX2 regulated genes in Wounding from PMID: 30772301	SOX2
G3BP1	SOX2 regulated genes in Wounding from PMID: 30772301	SOX2
CAB39	SOX2 regulated genes in Wounding from PMID: 30772301	SOX2
PCBP4	SOX2 regulated genes in Wounding from PMID: 30772301	SOX2

THOC3	SOX2 regulated genes in Wounding from PMID: 30772301	SOX2
YAF2	SOX2 regulated genes in Wounding from PMID: 30772301	SOX2
GZMM	SOX2 regulated genes in Wounding from PMID: 30772301	SOX2
CYP2S1	SOX2 regulated genes in Wounding from PMID: 30772301	SOX2
SUCO	SOX2 regulated genes in Wounding from PMID: 30772301	SOX2
CAST	SOX2 regulated genes in Wounding from PMID: 30772301	SOX2
RNF225	SOX2 regulated genes in Wounding from PMID: 30772301	SOX2
KLHL12	SOX2 regulated genes in Wounding from PMID: 30772301	SOX2
STON2	SOX2 regulated genes in Wounding from PMID: 30772301	SOX2
SLC20A1	SOX2 regulated genes in Wounding from PMID: 30772301	SOX2
SCNN1A	SOX2 regulated genes in Wounding from PMID: 30772301	SOX2
NAPA	SOX2 regulated genes in Wounding from PMID: 30772301	SOX2
TMEM107	SOX2 regulated genes in Wounding from PMID: 30772301	SOX2
FGF22	SOX2 regulated genes in Wounding from PMID: 30772301	SOX2
DUSP11	SOX2 regulated genes in Wounding from PMID: 30772301	SOX2
NOP58	SOX2 regulated genes in Wounding from PMID: 30772301	SOX2
MFN1	SOX2 regulated genes in Wounding from PMID: 30772301	SOX2
UCK2	SOX2 regulated genes in Wounding from PMID: 30772301	SOX2
RNF10	SOX2 regulated genes in Wounding from PMID: 30772301	SOX2
CLIP4	SOX2 regulated genes in Wounding from PMID: 30772301	SOX2
ZSWIM4	SOX2 regulated genes in Wounding from PMID: 30772301	SOX2
RAB10	SOX2 regulated genes in Wounding from PMID: 30772301	SOX2
KDM6B	SOX2 regulated genes in Wounding from PMID: 30772301	SOX2
SPINK6	SOX2 regulated genes in Wounding from PMID: 30772301	SOX2
LEMD3	SOX2 regulated genes in Wounding from PMID: 30772301	SOX2
KDM1A	SOX2 regulated genes in Wounding from PMID: 30772301	SOX2
TTYH2	SOX2 regulated genes in Wounding from PMID: 30772301	SOX2
ANAPC13	SOX2 regulated genes in Wounding from PMID: 30772301	SOX2
EPHB3	SOX2 regulated genes in Wounding from PMID: 30772301	SOX2
FBXO45	SOX2 regulated genes in Wounding from PMID: 30772301	SOX2
RHBG	SOX2 regulated genes in Wounding from PMID: 30772301	SOX2
SEC13	SOX2 regulated genes in Wounding from PMID: 30772301	SOX2
MAPK6	SOX2 regulated genes in Wounding from PMID: 30772301	SOX2
ZNF750	SOX2 regulated genes in Wounding from PMID: 30772301	SOX2
CPS1	SOX2 regulated genes in Wounding from PMID: 30772301	SOX2
GOLGA7	SOX2 regulated genes in Wounding from PMID: 30772301	SOX2
CUL1	SOX2 regulated genes in Wounding from PMID: 30772301	SOX2
TRIM16	SOX2 regulated genes in Wounding from PMID: 30772301	SOX2
IRF6	SOX2 regulated genes in Wounding from PMID: 30772301	SOX2
LRRC20	SOX2 regulated genes in Wounding from PMID: 30772301	SOX2
RASSF1	SOX2 regulated genes in Wounding from PMID: 30772301	SOX2
INTS7	SOX2 regulated genes in Wounding from PMID: 30772301	SOX2
SPINT2	SOX2 regulated genes in Wounding from PMID: 30772301	SOX2
RYK	SOX2 regulated genes in Wounding from PMID: 30772301	SOX2
SPTBN2	SOX2 regulated genes in Wounding from PMID: 30772301	SOX2
FPGT	SOX2 regulated genes in Wounding from PMID: 30772301	SOX2
KRTAP5-8	SOX2 regulated genes in Wounding from PMID: 30772301	SOX2
NRAS	SOX2 regulated genes in Wounding from PMID: 30772301	SOX2
WNT5A	SOX2 regulated genes in Wounding from PMID: 30772301	SOX2
CDC42EP3	SOX2 regulated genes in Wounding from PMID: 30772301	SOX2
CUL3	SOX2 regulated genes in Wounding from PMID: 30772301	SOX2
CAMSAP2	SOX2 regulated genes in Wounding from PMID: 30772301	SOX2
ASCL4	SOX2 regulated genes in Wounding from PMID: 30772301	SOX2
NOTUM	SOX2 regulated genes in Wounding from PMID: 30772301	SOX2
BBS5	SOX2 regulated genes in Wounding from PMID: 30772301	SOX2
GPA33	SOX2 regulated genes in Wounding from PMID: 30772301	SOX2
GNB1	SOX2 regulated genes in Wounding from PMID: 30772301	SOX2
CYP4F11	SOX2 regulated genes in Wounding from PMID: 30772301	SOX2
CYP4A11	SOX2 regulated genes in Wounding from PMID: 30772301	SOX2

MARVELD2	SOX2 regulated genes in Wounding from PMID: 30772301	SOX2
SDC1	SOX2 regulated genes in Wounding from PMID: 30772301	SOX2
KRT33A	SOX2 regulated genes in Wounding from PMID: 30772301	SOX2
BMPR2	SOX2 regulated genes in Wounding from PMID: 30772301	SOX2
PATJ	SOX2 regulated genes in Wounding from PMID: 30772301	SOX2
ARF1	SOX2 regulated genes in Wounding from PMID: 30772301	SOX2
TCF3	SOX2 regulated genes in Wounding from PMID: 30772301	SOX2
CORO1C	SOX2 regulated genes in Wounding from PMID: 30772301	SOX2
EZR	SOX2 regulated genes in Wounding from PMID: 30772301	SOX2
APPBP2	SOX2 regulated genes in Wounding from PMID: 30772301	SOX2
RBM12B	SOX2 regulated genes in Wounding from PMID: 30772301	SOX2
RASA2	SOX2 regulated genes in Wounding from PMID: 30772301	SOX2
ATP1A1	SOX2 regulated genes in Wounding from PMID: 30772301	SOX2
DUOX1	SOX2 regulated genes in Wounding from PMID: 30772301	SOX2
LSM12	SOX2 regulated genes in Wounding from PMID: 30772301	SOX2
GPX2	SOX2 regulated genes in Wounding from PMID: 30772301	SOX2
GPR87	SOX2 regulated genes in Wounding from PMID: 30772301	SOX2
KRTAP3-3	SOX2 regulated genes in Wounding from PMID: 30772301	SOX2
KRTAP3-2	SOX2 regulated genes in Wounding from PMID: 30772301	SOX2
DUSP7	SOX2 regulated genes in Wounding from PMID: 30772301	SOX2
APLP1	SOX2 regulated genes in Wounding from PMID: 30772301	SOX2
GOLIM4	SOX2 regulated genes in Wounding from PMID: 30772301	SOX2
CTNND1	SOX2 regulated genes in Wounding from PMID: 30772301	SOX2
PLPPR2	SOX2 regulated genes in Wounding from PMID: 30772301	SOX2
FAF1	SOX2 regulated genes in Wounding from PMID: 30772301	SOX2
IFT20	SOX2 regulated genes in Wounding from PMID: 30772301	SOX2
LAMC2	SOX2 regulated genes in Wounding from PMID: 30772301	SOX2
ZNF7	SOX2 regulated genes in Wounding from PMID: 30772301	SOX2
FOXP4	SOX2 regulated genes in Wounding from PMID: 30772301	SOX2
RNF145	SOX2 regulated genes in Wounding from PMID: 30772301	SOX2
ITPKB	SOX2 regulated genes in Wounding from PMID: 30772301	SOX2
ACVR1B	SOX2 regulated genes in Wounding from PMID: 30772301	SOX2
IGSF3	SOX2 regulated genes in Wounding from PMID: 30772301	SOX2
PIK3R3	SOX2 regulated genes in Wounding from PMID: 30772301	SOX2
CBX2	SOX2 regulated genes in Wounding from PMID: 30772301	SOX2
USP7	SOX2 regulated genes in Wounding from PMID: 30772301	SOX2
KMT5A	SOX2 regulated genes in Wounding from PMID: 30772301	SOX2
MMADHC	SOX2 regulated genes in Wounding from PMID: 30772301	SOX2
RRP15	SOX2 regulated genes in Wounding from PMID: 30772301	SOX2
PRMT7	SOX2 regulated genes in Wounding from PMID: 30772301	SOX2
TSR1	SOX2 regulated genes in Wounding from PMID: 30772301	SOX2
HDAC1	SOX2 regulated genes in Wounding from PMID: 30772301	SOX2
OTOP2	SOX2 regulated genes in Wounding from PMID: 30772301	SOX2
EVPL	SOX2 regulated genes in Wounding from PMID: 30772301	SOX2
IST1	SOX2 regulated genes in Wounding from PMID: 30772301	SOX2
ATP2A2	SOX2 regulated genes in Wounding from PMID: 30772301	SOX2
CITED4	SOX2 regulated genes in Wounding from PMID: 30772301	SOX2
ERBB3	SOX2 regulated genes in Wounding from PMID: 30772301	SOX2
CCDC92	SOX2 regulated genes in Wounding from PMID: 30772301	SOX2
KIFC2	SOX2 regulated genes in Wounding from PMID: 30772301	SOX2
ACTG1	SOX2 regulated genes in Wounding from PMID: 30772301	SOX2
CFTR	SOX2 regulated genes in Wounding from PMID: 30772301	SOX2
TRIM59	SOX2 regulated genes in Wounding from PMID: 30772301	SOX2
AGBL5	SOX2 regulated genes in Wounding from PMID: 30772301	SOX2
B4GALT3	SOX2 regulated genes in Wounding from PMID: 30772301	SOX2
MAP4K4	SOX2 regulated genes in Wounding from PMID: 30772301	SOX2
TMEM65	SOX2 regulated genes in Wounding from PMID: 30772301	SOX2
RNF39	SOX2 regulated genes in Wounding from PMID: 30772301	SOX2
C6orf141	SOX2 regulated genes in Wounding from PMID: 30772301	SOX2

CTPS1	SOX2 regulated genes in Wounding from PMID: 30772301	SOX2
ATP2C2	SOX2 regulated genes in Wounding from PMID: 30772301	SOX2
ADGRL2	SOX2 regulated genes in Wounding from PMID: 30772301	SOX2
WDR75	SOX2 regulated genes in Wounding from PMID: 30772301	SOX2
LRP12	SOX2 regulated genes in Wounding from PMID: 30772301	SOX2
TMEM120A	SOX2 regulated genes in Wounding from PMID: 30772301	SOX2
FER	SOX2 regulated genes in Wounding from PMID: 30772301	SOX2
HSPA4	SOX2 regulated genes in Wounding from PMID: 30772301	SOX2
PTPN9	SOX2 regulated genes in Wounding from PMID: 30772301	SOX2
DVL3	SOX2 regulated genes in Wounding from PMID: 30772301	SOX2
FZD5	SOX2 regulated genes in Wounding from PMID: 30772301	SOX2
USP53	SOX2 regulated genes in Wounding from PMID: 30772301	SOX2
CHD1	SOX2 regulated genes in Wounding from PMID: 30772301	SOX2
KLF10	SOX2 regulated genes in Wounding from PMID: 30772301	SOX2
IGSF8	SOX2 regulated genes in Wounding from PMID: 30772301	SOX2
SYT8	SOX2 regulated genes in Wounding from PMID: 30772301	SOX2
PTBP1	SOX2 regulated genes in Wounding from PMID: 30772301	SOX2
WWC1	SOX2 regulated genes in Wounding from PMID: 30772301	SOX2
PITHD1	SOX2 regulated genes in Wounding from PMID: 30772301	SOX2
RBMXL1	SOX2 regulated genes in Wounding from PMID: 30772301	SOX2
RASGEF1B	SOX2 regulated genes in Wounding from PMID: 30772301	SOX2
RNF25	SOX2 regulated genes in Wounding from PMID: 30772301	SOX2
PADI3	SOX2 regulated genes in Wounding from PMID: 30772301	SOX2
CSRP1	SOX2 regulated genes in Wounding from PMID: 30772301	SOX2
AGRN	SOX2 regulated genes in Wounding from PMID: 30772301	SOX2
TMEM229B	SOX2 regulated genes in Wounding from PMID: 30772301	SOX2
CHD4	SOX2 regulated genes in Wounding from PMID: 30772301	SOX2
CYTH2	SOX2 regulated genes in Wounding from PMID: 30772301	SOX2
SLC30A3	SOX2 regulated genes in Wounding from PMID: 30772301	SOX2
EPHA4	SOX2 regulated genes in Wounding from PMID: 30772301	SOX2
CRIP3	SOX2 regulated genes in Wounding from PMID: 30772301	SOX2
ACTR1B	SOX2 regulated genes in Wounding from PMID: 30772301	SOX2
LY6G6D	SOX2 regulated genes in Wounding from PMID: 30772301	SOX2
NGF	SOX2 regulated genes in Wounding from PMID: 30772301	SOX2
ZRANB2	SOX2 regulated genes in Wounding from PMID: 30772301	SOX2
SSTR2	SOX2 regulated genes in Wounding from PMID: 30772301	SOX2
GALE	SOX2 regulated genes in Wounding from PMID: 30772301	SOX2
TMEM68	SOX2 regulated genes in Wounding from PMID: 30772301	SOX2
GATA3	SOX2 regulated genes in Wounding from PMID: 30772301	SOX2
SPTY2D1	SOX2 regulated genes in Wounding from PMID: 30772301	SOX2
ZNF605	SOX2 regulated genes in Wounding from PMID: 30772301	SOX2
PRPF40B	SOX2 regulated genes in Wounding from PMID: 30772301	SOX2
KLHL29	SOX2 regulated genes in Wounding from PMID: 30772301	SOX2
TUBA1A	SOX2 regulated genes in Wounding from PMID: 30772301	SOX2
TAF7	SOX2 regulated genes in Wounding from PMID: 30772301	SOX2
CEP83	SOX2 regulated genes in Wounding from PMID: 30772301	SOX2
LLGL2	SOX2 regulated genes in Wounding from PMID: 30772301	SOX2
KRT79	SOX2 regulated genes in Wounding from PMID: 30772301	SOX2
MAP6	SOX2 regulated genes in Wounding from PMID: 30772301	SOX2
PLK3	SOX2 regulated genes in Wounding from PMID: 30772301	SOX2
ACSL3	SOX2 regulated genes in Wounding from PMID: 30772301	SOX2
EGR2	SOX2 regulated genes in Wounding from PMID: 30772301	SOX2
PALMD	SOX2 regulated genes in Wounding from PMID: 30772301	SOX2
KIF21B	SOX2 regulated genes in Wounding from PMID: 30772301	SOX2
TRNP1	SOX2 regulated genes in Wounding from PMID: 30772301	SOX2
NUDCD1	SOX2 regulated genes in Wounding from PMID: 30772301	SOX2
DYNC1I2	SOX2 regulated genes in Wounding from PMID: 30772301	SOX2
ANGPTL4	SOX2 regulated genes in Wounding from PMID: 30772301	SOX2
POLB	SOX2 regulated genes in Wounding from PMID: 30772301	SOX2

TMEM139	SOX2 regulated genes in Wounding from PMID: 30772301	SOX2
ABCC1	SOX2 regulated genes in Wounding from PMID: 30772301	SOX2
KLHDC8A	SOX2 regulated genes in Wounding from PMID: 30772301	SOX2
HMGCS2	SOX2 regulated genes in Wounding from PMID: 30772301	SOX2
OTUD7B	SOX2 regulated genes in Wounding from PMID: 30772301	SOX2
LINS1	SOX2 regulated genes in Wounding from PMID: 30772301	SOX2
FLG2	SOX2 regulated genes in Wounding from PMID: 30772301	SOX2
CCDC106	SOX2 regulated genes in Wounding from PMID: 30772301	SOX2
ENC1	SOX2 regulated genes in Wounding from PMID: 30772301	SOX2
TPPP3	SOX2 regulated genes in Wounding from PMID: 30772301	SOX2
SYTL2	SOX2 regulated genes in Wounding from PMID: 30772301	SOX2
HRAS	SOX2 regulated genes in Wounding from PMID: 30772301	SOX2
CCT7	SOX2 regulated genes in Wounding from PMID: 30772301	SOX2
BAHCC1	SOX2 regulated genes in Wounding from PMID: 30772301	SOX2
TUFT1	SOX2 regulated genes in Wounding from PMID: 30772301	SOX2
SHOC2	SOX2 regulated genes in Wounding from PMID: 30772301	SOX2
ARL13B	SOX2 regulated genes in Wounding from PMID: 30772301	SOX2
SLC4A9	SOX2 regulated genes in Wounding from PMID: 30772301	SOX2
LYG2	SOX2 regulated genes in Wounding from PMID: 30772301	SOX2
IRGQ	SOX2 regulated genes in Wounding from PMID: 30772301	SOX2
FXYD3	SOX2 regulated genes in Wounding from PMID: 30772301	SOX2
MIDN	SOX2 regulated genes in Wounding from PMID: 30772301	SOX2
PTGS2	SOX2 regulated genes in Wounding from PMID: 30772301	SOX2
FSTL3	SOX2 regulated genes in Wounding from PMID: 30772301	SOX2
RNF223	SOX2 regulated genes in Wounding from PMID: 30772301	SOX2
GTF2H2C	SOX2 regulated genes in Wounding from PMID: 30772301	SOX2
BMPR1A	SOX2 regulated genes in Wounding from PMID: 30772301	SOX2
SETD5	SOX2 regulated genes in Wounding from PMID: 30772301	SOX2
S100A11	SOX2 regulated genes in Wounding from PMID: 30772301	SOX2
NT5C3A	SOX2 regulated genes in Wounding from PMID: 30772301	SOX2
RAB7B	SOX2 regulated genes in Wounding from PMID: 30772301	SOX2
HOOK2	SOX2 regulated genes in Wounding from PMID: 30772301	SOX2
TBC1D30	SOX2 regulated genes in Wounding from PMID: 30772301	SOX2
ZBTB17	SOX2 regulated genes in Wounding from PMID: 30772301	SOX2
ANGPT2	SOX2 regulated genes in Wounding from PMID: 30772301	SOX2
EBNA1BP2	SOX2 regulated genes in Wounding from PMID: 30772301	SOX2
CNOT2	SOX2 regulated genes in Wounding from PMID: 30772301	SOX2
PLXNB1	SOX2 regulated genes in Wounding from PMID: 30772301	SOX2
GALR2	SOX2 regulated genes in Wounding from PMID: 30772301	SOX2
EIF3B	SOX2 regulated genes in Wounding from PMID: 30772301	SOX2
MAGI1	SOX2 regulated genes in Wounding from PMID: 30772301	SOX2
ANXA9	SOX2 regulated genes in Wounding from PMID: 30772301	SOX2
SPRR4	SOX2 regulated genes in Wounding from PMID: 30772301	SOX2
ERH	SOX2 regulated genes in Wounding from PMID: 30772301	SOX2
HR	SOX2 regulated genes in Wounding from PMID: 30772301	SOX2
CCNYL1	SOX2 regulated genes in Wounding from PMID: 30772301	SOX2
FAM89A	SOX2 regulated genes in Wounding from PMID: 30772301	SOX2
KRTAP4-6	SOX2 regulated genes in Wounding from PMID: 30772301	SOX2
TCHH	SOX2 regulated genes in Wounding from PMID: 30772301	SOX2
FAM221A	SOX2 regulated genes in Wounding from PMID: 30772301	SOX2
ENPP1	SOX2 regulated genes in Wounding from PMID: 30772301	SOX2
KPNB1	SOX2 regulated genes in Wounding from PMID: 30772301	SOX2
MFF	SOX2 regulated genes in Wounding from PMID: 30772301	SOX2
CDC42SE1	SOX2 regulated genes in Wounding from PMID: 30772301	SOX2
ALYREF	SOX2 regulated genes in Wounding from PMID: 30772301	SOX2
RND3	SOX2 regulated genes in Wounding from PMID: 30772301	SOX2
TANC2	SOX2 regulated genes in Wounding from PMID: 30772301	SOX2
HDLBP	SOX2 regulated genes in Wounding from PMID: 30772301	SOX2
C17orf80	SOX2 regulated genes in Wounding from PMID: 30772301	SOX2

CAP1	SOX2 regulated genes in Wounding from PMID: 30772301	SOX2
ATP6V0E1	SOX2 regulated genes in Wounding from PMID: 30772301	SOX2
PGM1	SOX2 regulated genes in Wounding from PMID: 30772301	SOX2
MAPRE3	SOX2 regulated genes in Wounding from PMID: 30772301	SOX2
C3orf52	SOX2 regulated genes in Wounding from PMID: 30772301	SOX2
TXNRD3	SOX2 regulated genes in Wounding from PMID: 30772301	SOX2
WIZ	SOX2 regulated genes in Wounding from PMID: 30772301	SOX2
YWHAE	SOX2 regulated genes in Wounding from PMID: 30772301	SOX2
CUL2	SOX2 regulated genes in Wounding from PMID: 30772301	SOX2
SMYD2	SOX2 regulated genes in Wounding from PMID: 30772301	SOX2
TMEM181	SOX2 regulated genes in Wounding from PMID: 30772301	SOX2
PDGFA	SOX2 regulated genes in Wounding from PMID: 30772301	SOX2
CDH1	SOX2 regulated genes in Wounding from PMID: 30772301	SOX2
E2F3	SOX2 regulated genes in Wounding from PMID: 30772301	SOX2
SPECC1	SOX2 regulated genes in Wounding from PMID: 30772301	SOX2
GMPPB	SOX2 regulated genes in Wounding from PMID: 30772301	SOX2
TATDN2	SOX2 regulated genes in Wounding from PMID: 30772301	SOX2
SEC22A	SOX2 regulated genes in Wounding from PMID: 30772301	SOX2
PTGES3	SOX2 regulated genes in Wounding from PMID: 30772301	SOX2
CCT3	SOX2 regulated genes in Wounding from PMID: 30772301	SOX2
RAD23A	SOX2 regulated genes in Wounding from PMID: 30772301	SOX2
LYPD6B	SOX2 regulated genes in Wounding from PMID: 30772301	SOX2
DCLK3	SOX2 regulated genes in Wounding from PMID: 30772301	SOX2
GPRC5A	SOX2 regulated genes in Wounding from PMID: 30772301	SOX2
PIK3CB	SOX2 regulated genes in Wounding from PMID: 30772301	SOX2
BEND5	SOX2 regulated genes in Wounding from PMID: 30772301	SOX2
DPP9	SOX2 regulated genes in Wounding from PMID: 30772301	SOX2
SLC23A3	SOX2 regulated genes in Wounding from PMID: 30772301	SOX2
PAFAH1B3	SOX2 regulated genes in Wounding from PMID: 30772301	SOX2
TRIM5	SOX2 regulated genes in Wounding from PMID: 30772301	SOX2
PIP5K1A	SOX2 regulated genes in Wounding from PMID: 30772301	SOX2
RECQL	SOX2 regulated genes in Wounding from PMID: 30772301	SOX2
TNFRSF10B	SOX2 regulated genes in Wounding from PMID: 30772301	SOX2
TNFRSF10C	SOX2 regulated genes in Wounding from PMID: 30772301	SOX2
TNFRSF10D	SOX2 regulated genes in Wounding from PMID: 30772301	SOX2
YME1L1	SOX2 regulated genes in Wounding from PMID: 30772301	SOX2
HBEGF	SOX2 regulated genes in Wounding from PMID: 30772301	SOX2
RALGPS2	SOX2 regulated genes in Wounding from PMID: 30772301	SOX2
PDCD4	SOX2 regulated genes in Wounding from PMID: 30772301	SOX2
CNST	SOX2 regulated genes in Wounding from PMID: 30772301	SOX2
HNRNPU	SOX2 regulated genes in Wounding from PMID: 30772301	SOX2
ZNF746	SOX2 regulated genes in Wounding from PMID: 30772301	SOX2
TMEM69	SOX2 regulated genes in Wounding from PMID: 30772301	SOX2
WDR47	SOX2 regulated genes in Wounding from PMID: 30772301	SOX2
TUBA4A	SOX2 regulated genes in Wounding from PMID: 30772301	SOX2
SYNRG	SOX2 regulated genes in Wounding from PMID: 30772301	SOX2
HECTD1	SOX2 regulated genes in Wounding from PMID: 30772301	SOX2
FADS2	SOX2 regulated genes in Wounding from PMID: 30772301	SOX2
QRICH2	SOX2 regulated genes in Wounding from PMID: 30772301	SOX2
IWS1	SOX2 regulated genes in Wounding from PMID: 30772301	SOX2
SAXO2	SOX2 regulated genes in Wounding from PMID: 30772301	SOX2
IL11	SOX2 regulated genes in Wounding from PMID: 30772301	SOX2
DVL1	SOX2 regulated genes in Wounding from PMID: 30772301	SOX2
BHLHE41	SOX2 regulated genes in Wounding from PMID: 30772301	SOX2
ATP6V0A2	SOX2 regulated genes in Wounding from PMID: 30772301	SOX2
SPINT1	SOX2 regulated genes in Wounding from PMID: 30772301	SOX2
GCLM	SOX2 regulated genes in Wounding from PMID: 30772301	SOX2
CNTN5	SOX2 regulated genes in Wounding from PMID: 30772301	SOX2
BZW2	SOX2 regulated genes in Wounding from PMID: 30772301	SOX2

ST7L	SOX2 regulated genes in Wounding from PMID: 30772301	SOX2
ENOPH1	SOX2 regulated genes in Wounding from PMID: 30772301	SOX2
STX6	SOX2 regulated genes in Wounding from PMID: 30772301	SOX2
FAT1	SOX2 regulated genes in Wounding from PMID: 30772301	SOX2
SDR16C5	SOX2 regulated genes in Wounding from PMID: 30772301	SOX2
ZNF266	SOX2 regulated genes in Wounding from PMID: 30772301	SOX2
POLR2D	SOX2 regulated genes in Wounding from PMID: 30772301	SOX2
CCDC91	SOX2 regulated genes in Wounding from PMID: 30772301	SOX2
PDPN	SOX2 regulated genes in Wounding from PMID: 30772301	SOX2
VANGL1	SOX2 regulated genes in Wounding from PMID: 30772301	SOX2
KCNK12	SOX2 regulated genes in Wounding from PMID: 30772301	SOX2
ITGA6	SOX2 regulated genes in Wounding from PMID: 30772301	SOX2
GPR42	SOX2 regulated genes in Wounding from PMID: 30772301	SOX2
FILIP1L	SOX2 regulated genes in Wounding from PMID: 30772301	SOX2
NDUFAF7	SOX2 regulated genes in Wounding from PMID: 30772301	SOX2
MPP7	SOX2 regulated genes in Wounding from PMID: 30772301	SOX2
MYH14	SOX2 regulated genes in Wounding from PMID: 30772301	SOX2
GTF2F1	SOX2 regulated genes in Wounding from PMID: 30772301	SOX2
DHRS9	SOX2 regulated genes in Wounding from PMID: 30772301	SOX2
MARK1	SOX2 regulated genes in Wounding from PMID: 30772301	SOX2
C12orf56	SOX2 regulated genes in Wounding from PMID: 30772301	SOX2
BNC1	SOX2 regulated genes in Wounding from PMID: 30772301	SOX2
POGK	SOX2 regulated genes in Wounding from PMID: 30772301	SOX2
P4HB	SOX2 regulated genes in Wounding from PMID: 30772301	SOX2
B3GNT8	SOX2 regulated genes in Wounding from PMID: 30772301	SOX2
METTL23	SOX2 regulated genes in Wounding from PMID: 30772301	SOX2
TMED5	SOX2 regulated genes in Wounding from PMID: 30772301	SOX2
SLC4A1	SOX2 regulated genes in Wounding from PMID: 30772301	SOX2
PKP2	SOX2 regulated genes in Wounding from PMID: 30772301	SOX2
GPSM2	SOX2 regulated genes in Wounding from PMID: 30772301	SOX2
ITGB4	SOX2 regulated genes in Wounding from PMID: 30772301	SOX2
CLASP1	SOX2 regulated genes in Wounding from PMID: 30772301	SOX2
SIRT1	SOX2 regulated genes in Wounding from PMID: 30772301	SOX2
CCHCR1	SOX2 regulated genes in Wounding from PMID: 30772301	SOX2
TSG101	SOX2 regulated genes in Wounding from PMID: 30772301	SOX2
MACF1	SOX2 regulated genes in Wounding from PMID: 30772301	SOX2
TRAF4	SOX2 regulated genes in Wounding from PMID: 30772301	SOX2
TXNRD1	SOX2 regulated genes in Wounding from PMID: 30772301	SOX2
ARHGAP22	SOX2 regulated genes in Wounding from PMID: 30772301	SOX2
INTS14	SOX2 regulated genes in Wounding from PMID: 30772301	SOX2
CLK3	SOX2 regulated genes in Wounding from PMID: 30772301	SOX2
EGR1	SOX2 regulated genes in Wounding from PMID: 30772301	SOX2
ECD	SOX2 regulated genes in Wounding from PMID: 30772301	SOX2
MYH15	SOX2 regulated genes in Wounding from PMID: 30772301	SOX2
NSUN2	SOX2 regulated genes in Wounding from PMID: 30772301	SOX2
GEMIN4	SOX2 regulated genes in Wounding from PMID: 30772301	SOX2
LAD1	SOX2 regulated genes in Wounding from PMID: 30772301	SOX2
RBBP6	SOX2 regulated genes in Wounding from PMID: 30772301	SOX2
ATXN7L3	SOX2 regulated genes in Wounding from PMID: 30772301	SOX2
PPP2CB	SOX2 regulated genes in Wounding from PMID: 30772301	SOX2
SPPL3	SOX2 regulated genes in Wounding from PMID: 30772301	SOX2
SNAPC3	SOX2 regulated genes in Wounding from PMID: 30772301	SOX2
FBXO46	SOX2 regulated genes in Wounding from PMID: 30772301	SOX2
PDXDC1	SOX2 regulated genes in Wounding from PMID: 30772301	SOX2
HGS	SOX2 regulated genes in Wounding from PMID: 30772301	SOX2
STEAP1	SOX2 regulated genes in Wounding from PMID: 30772301	SOX2
KCTD5	SOX2 regulated genes in Wounding from PMID: 30772301	SOX2
PRSS22	SOX2 regulated genes in Wounding from PMID: 30772301	SOX2
PLXNA1	SOX2 regulated genes in Wounding from PMID: 30772301	SOX2

STRIP1	SOX2 regulated genes in Wounding from PMID: 30772301	SOX2
DLG1	SOX2 regulated genes in Wounding from PMID: 30772301	SOX2
TP63	SOX2 regulated genes in Wounding from PMID: 30772301	SOX2
USP43	SOX2 regulated genes in Wounding from PMID: 30772301	SOX2
MGST3	SOX2 regulated genes in Wounding from PMID: 30772301	SOX2
ANKRD13B	SOX2 regulated genes in Wounding from PMID: 30772301	SOX2
MPZL1	SOX2 regulated genes in Wounding from PMID: 30772301	SOX2
SMURF2	SOX2 regulated genes in Wounding from PMID: 30772301	SOX2
PRPF3	SOX2 regulated genes in Wounding from PMID: 30772301	SOX2
RBM45	SOX2 regulated genes in Wounding from PMID: 30772301	SOX2
SLC30A1	SOX2 regulated genes in Wounding from PMID: 30772301	SOX2
SHH	SOX2 regulated genes in Wounding from PMID: 30772301	SOX2
SLC7A8	SOX2 regulated genes in Wounding from PMID: 30772301	SOX2
PHACTR1	SOX2 regulated genes in Wounding from PMID: 30772301	SOX2
BPNT1	SOX2 regulated genes in Wounding from PMID: 30772301	SOX2
PCDHGB1	SOX2 regulated genes in Wounding from PMID: 30772301	SOX2
PSMD7	SOX2 regulated genes in Wounding from PMID: 30772301	SOX2
PELI1	SOX2 regulated genes in Wounding from PMID: 30772301	SOX2
ZNF800	SOX2 regulated genes in Wounding from PMID: 30772301	SOX2
FCAMR	SOX2 regulated genes in Wounding from PMID: 30772301	SOX2
DDX1	SOX2 regulated genes in Wounding from PMID: 30772301	SOX2
PPIP5K1	SOX2 regulated genes in Wounding from PMID: 30772301	SOX2
RHOU	SOX2 regulated genes in Wounding from PMID: 30772301	SOX2
UBA5	SOX2 regulated genes in Wounding from PMID: 30772301	SOX2
LRRFIP1	SOX2 regulated genes in Wounding from PMID: 30772301	SOX2
NIPAL4	SOX2 regulated genes in Wounding from PMID: 30772301	SOX2
PDK1	SOX2 regulated genes in Wounding from PMID: 30772301	SOX2
TMEM40	SOX2 regulated genes in Wounding from PMID: 30772301	SOX2
NME6	SOX2 regulated genes in Wounding from PMID: 30772301	SOX2
SLC27A6	SOX2 regulated genes in Wounding from PMID: 30772301	SOX2
PGAP1	SOX2 regulated genes in Wounding from PMID: 30772301	SOX2
KCTD11	SOX2 regulated genes in Wounding from PMID: 30772301	SOX2
POMGNT1	SOX2 regulated genes in Wounding from PMID: 30772301	SOX2
PRMT1	SOX2 regulated genes in Wounding from PMID: 30772301	SOX2
PDLIM4	SOX2 regulated genes in Wounding from PMID: 30772301	SOX2
CHMP4C	SOX2 regulated genes in Wounding from PMID: 30772301	SOX2
SLC44A2	SOX2 regulated genes in Wounding from PMID: 30772301	SOX2
LUZP1	SOX2 regulated genes in Wounding from PMID: 30772301	SOX2
TWF1	SOX2 regulated genes in Wounding from PMID: 30772301	SOX2
CTSE	SOX2 regulated genes in Wounding from PMID: 30772301	SOX2
CDC42BPA	SOX2 regulated genes in Wounding from PMID: 30772301	SOX2
SSR2	SOX2 regulated genes in Wounding from PMID: 30772301	SOX2
LIMA1	SOX2 regulated genes in Wounding from PMID: 30772301	SOX2
EFNA4	SOX2 regulated genes in Wounding from PMID: 30772301	SOX2
GUCY2C	SOX2 regulated genes in Wounding from PMID: 30772301	SOX2
ATP1B1	SOX2 regulated genes in Wounding from PMID: 30772301	SOX2
EFNA1	SOX2 regulated genes in Wounding from PMID: 30772301	SOX2
GTF2IRD1	SOX2 regulated genes in Wounding from PMID: 30772301	SOX2
PSME3	SOX2 regulated genes in Wounding from PMID: 30772301	SOX2
MKRN2OS	SOX2 regulated genes in Wounding from PMID: 30772301	SOX2
B3GNT5	SOX2 regulated genes in Wounding from PMID: 30772301	SOX2
CNTN2	SOX2 regulated genes in Wounding from PMID: 30772301	SOX2
KLC3	SOX2 regulated genes in Wounding from PMID: 30772301	SOX2
HIP1R	SOX2 regulated genes in Wounding from PMID: 30772301	SOX2
EFNA5	SOX2 regulated genes in Wounding from PMID: 30772301	SOX2
DAZAP1	SOX2 regulated genes in Wounding from PMID: 30772301	SOX2
PRKAR1A	SOX2 regulated genes in Wounding from PMID: 30772301	SOX2
NAV2	SOX2 regulated genes in Wounding from PMID: 30772301	SOX2
RANBP10	SOX2 regulated genes in Wounding from PMID: 30772301	SOX2

PFN2	SOX2 regulated genes in Wounding from PMID: 30772301	SOX2
SNX16	SOX2 regulated genes in Wounding from PMID: 30772301	SOX2
NME1	SOX2 regulated genes in Wounding from PMID: 30772301	SOX2
FYTTD1	SOX2 regulated genes in Wounding from PMID: 30772301	SOX2
BEAN1	SOX2 regulated genes in Wounding from PMID: 30772301	SOX2
SORT1	SOX2 regulated genes in Wounding from PMID: 30772301	SOX2
NCMAP	SOX2 regulated genes in Wounding from PMID: 30772301	SOX2
LTBP3	SOX2 regulated genes in Wounding from PMID: 30772301	SOX2
B3GNT2	SOX2 regulated genes in Wounding from PMID: 30772301	SOX2
CCDC124	SOX2 regulated genes in Wounding from PMID: 30772301	SOX2
CLDN4	SOX2 regulated genes in Wounding from PMID: 30772301	SOX2
SHF	SOX2 regulated genes in Wounding from PMID: 30772301	SOX2
RPL29	SOX2 regulated genes in Wounding from PMID: 30772301	SOX2
COQ10B	SOX2 regulated genes in Wounding from PMID: 30772301	SOX2
FAM187B	SOX2 regulated genes in Wounding from PMID: 30772301	SOX2
PSMD8	SOX2 regulated genes in Wounding from PMID: 30772301	SOX2
GATAD2A	SOX2 regulated genes in Wounding from PMID: 30772301	SOX2
TUBB3	SOX2 regulated genes in Wounding from PMID: 30772301	SOX2
BZW1	SOX2 regulated genes in Wounding from PMID: 30772301	SOX2
KDM5B	SOX2 regulated genes in Wounding from PMID: 30772301	SOX2
TMEM222	SOX2 regulated genes in Wounding from PMID: 30772301	SOX2
HAUS8	SOX2 regulated genes in Wounding from PMID: 30772301	SOX2
IL20	SOX2 regulated genes in Wounding from PMID: 30772301	SOX2
TIGD5	SOX2 regulated genes in Wounding from PMID: 30772301	SOX2
FBXO28	SOX2 regulated genes in Wounding from PMID: 30772301	SOX2
VSIG8	SOX2 regulated genes in Wounding from PMID: 30772301	SOX2
FBXO38	SOX2 regulated genes in Wounding from PMID: 30772301	SOX2
CSNK1D	SOX2 regulated genes in Wounding from PMID: 30772301	SOX2
FOXN1	SOX2 regulated genes in Wounding from PMID: 30772301	SOX2
WNT3A	SOX2 regulated genes in Wounding from PMID: 30772301	SOX2
EAF1	SOX2 regulated genes in Wounding from PMID: 30772301	SOX2
TOP2B	SOX2 regulated genes in Wounding from PMID: 30772301	SOX2
NGEF	SOX2 regulated genes in Wounding from PMID: 30772301	SOX2
LRRC59	SOX2 regulated genes in Wounding from PMID: 30772301	SOX2
TTC13	SOX2 regulated genes in Wounding from PMID: 30772301	SOX2
WDR77	SOX2 regulated genes in Wounding from PMID: 30772301	SOX2
S100A3	SOX2 regulated genes in Wounding from PMID: 30772301	SOX2
GNAI3	SOX2 regulated genes in Wounding from PMID: 30772301	SOX2
KRT27	SOX2 regulated genes in Wounding from PMID: 30772301	SOX2
DIS3L	SOX2 regulated genes in Wounding from PMID: 30772301	SOX2
GAB1	SOX2 regulated genes in Wounding from PMID: 30772301	SOX2
DDX5	SOX2 regulated genes in Wounding from PMID: 30772301	SOX2
UBTF	SOX2 regulated genes in Wounding from PMID: 30772301	SOX2
TXLNA	SOX2 regulated genes in Wounding from PMID: 30772301	SOX2
MEMO1	SOX2 regulated genes in Wounding from PMID: 30772301	SOX2
CRABP2	SOX2 regulated genes in Wounding from PMID: 30772301	SOX2
DEDD2	SOX2 regulated genes in Wounding from PMID: 30772301	SOX2
TPRG1	SOX2 regulated genes in Wounding from PMID: 30772301	SOX2
MYO6	SOX2 regulated genes in Wounding from PMID: 30772301	SOX2
HOOK1	SOX2 regulated genes in Wounding from PMID: 30772301	SOX2
SEMA6A	SOX2 regulated genes in Wounding from PMID: 30772301	SOX2
TEPP	SOX2 regulated genes in Wounding from PMID: 30772301	SOX2
SMAD3	SOX2 regulated genes in Wounding from PMID: 30772301	SOX2
REEP5	SOX2 regulated genes in Wounding from PMID: 30772301	SOX2
C1QL1	SOX2 regulated genes in Wounding from PMID: 30772301	SOX2
ECE1	SOX2 regulated genes in Wounding from PMID: 30772301	SOX2
HINFP	SOX2 regulated genes in Wounding from PMID: 30772301	SOX2
KRT18	SOX2 regulated genes in Wounding from PMID: 30772301	SOX2
ARF6	SOX2 regulated genes in Wounding from PMID: 30772301	SOX2

GSK3B	SOX2 regulated genes in Wounding from PMID: 30772301	SOX2
DUSP10	SOX2 regulated genes in Wounding from PMID: 30772301	SOX2
SEC61A1	SOX2 regulated genes in Wounding from PMID: 30772301	SOX2
FBXW11	SOX2 regulated genes in Wounding from PMID: 30772301	SOX2
SF3B4	SOX2 regulated genes in Wounding from PMID: 30772301	SOX2
ATP2C1	SOX2 regulated genes in Wounding from PMID: 30772301	SOX2
KRT36	SOX2 regulated genes in Wounding from PMID: 30772301	SOX2
HEYL	SOX2 regulated genes in Wounding from PMID: 30772301	SOX2
PHC2	SOX2 regulated genes in Wounding from PMID: 30772301	SOX2
UBE2I	SOX2 regulated genes in Wounding from PMID: 30772301	SOX2
KCNH1	SOX2 regulated genes in Wounding from PMID: 30772301	SOX2
PPP1R13L	SOX2 regulated genes in Wounding from PMID: 30772301	SOX2
PPM1D	SOX2 regulated genes in Wounding from PMID: 30772301	SOX2
ACP5	SOX2 regulated genes in Wounding from PMID: 30772301	SOX2
MRGPRX3	SOX2 regulated genes in Wounding from PMID: 30772301	SOX2
CHMP2B	SOX2 regulated genes in Wounding from PMID: 30772301	SOX2
PIWIL2	SOX2 regulated genes in Wounding from PMID: 30772301	SOX2
ATP11B	SOX2 regulated genes in Wounding from PMID: 30772301	SOX2
CFAP57	SOX2 regulated genes in Wounding from PMID: 30772301	SOX2
EIF5A	SOX2 regulated genes in Wounding from PMID: 30772301	SOX2
TFG	SOX2 regulated genes in Wounding from PMID: 30772301	SOX2
RAB34	SOX2 regulated genes in Wounding from PMID: 30772301	SOX2
ATOH7	SOX2 regulated genes in Wounding from PMID: 30772301	SOX2
KLK7	SOX2 regulated genes in Wounding from PMID: 30772301	SOX2
PLEKHG6	SOX2 regulated genes in Wounding from PMID: 30772301	SOX2
ARAP2	SOX2 regulated genes in Wounding from PMID: 30772301	SOX2
PIAS4	SOX2 regulated genes in Wounding from PMID: 30772301	SOX2
ABCB11	SOX2 regulated genes in Wounding from PMID: 30772301	SOX2
CLDN1	SOX2 regulated genes in Wounding from PMID: 30772301	SOX2
LAMB3	SOX2 regulated genes in Wounding from PMID: 30772301	SOX2
HAS1	SOX2 regulated genes in Wounding from PMID: 30772301	SOX2
VTCN1	SOX2 regulated genes in Wounding from PMID: 30772301	SOX2
UBE2F	SOX2 regulated genes in Wounding from PMID: 30772301	SOX2
MUC1	SOX2 regulated genes in Wounding from PMID: 30772301	SOX2
TSC22D2	SOX2 regulated genes in Wounding from PMID: 30772301	SOX2
ACTL6A	SOX2 regulated genes in Wounding from PMID: 30772301	SOX2
TNRC18	SOX2 regulated genes in Wounding from PMID: 30772301	SOX2
DCAKD	SOX2 regulated genes in Wounding from PMID: 30772301	SOX2
LPAR3	SOX2 regulated genes in Wounding from PMID: 30772301	SOX2
NIF3L1	SOX2 regulated genes in Wounding from PMID: 30772301	SOX2
TAF13	SOX2 regulated genes in Wounding from PMID: 30772301	SOX2
PCDH1	SOX2 regulated genes in Wounding from PMID: 30772301	SOX2
RTN4	SOX2 regulated genes in Wounding from PMID: 30772301	SOX2
ZPR1	SOX2 regulated genes in Wounding from PMID: 30772301	SOX2
E2F2	SOX2 regulated genes in Wounding from PMID: 30772301	SOX2
GRAMD1C	SOX2 regulated genes in Wounding from PMID: 30772301	SOX2
GCNT2	SOX2 regulated genes in Wounding from PMID: 30772301	SOX2
RGMA	SOX2 regulated genes in Wounding from PMID: 30772301	SOX2
MXD1	SOX2 regulated genes in Wounding from PMID: 30772301	SOX2
MEX3A	SOX2 regulated genes in Wounding from PMID: 30772301	SOX2
DYRK3	SOX2 regulated genes in Wounding from PMID: 30772301	SOX2
RNF11	SOX2 regulated genes in Wounding from PMID: 30772301	SOX2
USP2	SOX2 regulated genes in Wounding from PMID: 30772301	SOX2
FSCN2	SOX2 regulated genes in Wounding from PMID: 30772301	SOX2
PGBD1	SOX2 regulated genes in Wounding from PMID: 30772301	SOX2
IFNLR1	SOX2 regulated genes in Wounding from PMID: 30772301	SOX2
EMP2	SOX2 regulated genes in Wounding from PMID: 30772301	SOX2
ACOT7	SOX2 regulated genes in Wounding from PMID: 30772301	SOX2
NTF4	SOX2 regulated genes in Wounding from PMID: 30772301	SOX2

SLCO2A1	SOX2 regulated genes in Wounding from PMID: 30772301	SOX2
TSPAN1	SOX2 regulated genes in Wounding from PMID: 30772301	SOX2
HNRNPF	SOX2 regulated genes in Wounding from PMID: 30772301	SOX2
MYO1B	SOX2 regulated genes in Wounding from PMID: 30772301	SOX2
DHX29	SOX2 regulated genes in Wounding from PMID: 30772301	SOX2
HOMER3	SOX2 regulated genes in Wounding from PMID: 30772301	SOX2
RIMS1	SOX2 regulated genes in Wounding from PMID: 30772301	SOX2
F11R	SOX2 regulated genes in Wounding from PMID: 30772301	SOX2
PHYHIP	SOX2 regulated genes in Wounding from PMID: 30772301	SOX2
ERC1	SOX2 regulated genes in Wounding from PMID: 30772301	SOX2
SLC45A3	SOX2 regulated genes in Wounding from PMID: 30772301	SOX2
GPR12	SOX2 regulated genes in Keratinocyte Overexpression from PMID: 30772301	SOX2
ZNF512B	SOX2 regulated genes in Keratinocyte Overexpression from PMID: 30772301	SOX2
PWP2	SOX2 regulated genes in Keratinocyte Overexpression from PMID: 30772301	SOX2
RAB27B	SOX2 regulated genes in Keratinocyte Overexpression from PMID: 30772301	SOX2
MZT1	SOX2 regulated genes in Keratinocyte Overexpression from PMID: 30772301	SOX2
SYN3	SOX2 regulated genes in Keratinocyte Overexpression from PMID: 30772301	SOX2
USP12	SOX2 regulated genes in Keratinocyte Overexpression from PMID: 30772301	SOX2
ARL5B	SOX2 regulated genes in Keratinocyte Overexpression from PMID: 30772301	SOX2
PCGF3	SOX2 regulated genes in Keratinocyte Overexpression from PMID: 30772301	SOX2
HORMAD2	SOX2 regulated genes in Keratinocyte Overexpression from PMID: 30772301	SOX2
LIPG	SOX2 regulated genes in Keratinocyte Overexpression from PMID: 30772301	SOX2
YES1	SOX2 regulated genes in Keratinocyte Overexpression from PMID: 30772301	SOX2
UNC79	SOX2 regulated genes in Keratinocyte Overexpression from PMID: 30772301	SOX2
ZBTB21	SOX2 regulated genes in Keratinocyte Overexpression from PMID: 30772301	SOX2
GTPBP6	SOX2 regulated genes in Keratinocyte Overexpression from PMID: 30772301	SOX2
CPLX1	SOX2 regulated genes in Keratinocyte Overexpression from PMID: 30772301	SOX2
ATG2B	SOX2 regulated genes in Keratinocyte Overexpression from PMID: 30772301	SOX2
SMOX	SOX2 regulated genes in Keratinocyte Overexpression from PMID: 30772301	SOX2
CLDN17	SOX2 regulated genes in Keratinocyte Overexpression from PMID: 30772301	SOX2
DCAF4	SOX2 regulated genes in Keratinocyte Overexpression from PMID: 30772301	SOX2
MOCOS	SOX2 regulated genes in Keratinocyte Overexpression from PMID: 30772301	SOX2
PBDC1	SOX2 regulated genes in Keratinocyte Overexpression from PMID: 30772301	SOX2
PRELID3B	SOX2 regulated genes in Keratinocyte Overexpression from PMID: 30772301	SOX2
MEX3C	SOX2 regulated genes in Keratinocyte Overexpression from PMID: 30772301	SOX2
EVA1C	SOX2 regulated genes in Keratinocyte Overexpression from PMID: 30772301	SOX2
PSMA7	SOX2 regulated genes in Keratinocyte Overexpression from PMID: 30772301	SOX2
NUDT5	SOX2 regulated genes in Keratinocyte Overexpression from PMID: 30772301	SOX2
VBP1	SOX2 regulated genes in Keratinocyte Overexpression from PMID: 30772301	SOX2
SLC25A43	SOX2 regulated genes in Keratinocyte Overexpression from PMID: 30772301	SOX2
TTLL1	SOX2 regulated genes in Keratinocyte Overexpression from PMID: 30772301	SOX2
SIM2	SOX2 regulated genes in Keratinocyte Overexpression from PMID: 30772301	SOX2
RRP1B	SOX2 regulated genes in Keratinocyte Overexpression from PMID: 30772301	SOX2
CEP76	SOX2 regulated genes in Keratinocyte Overexpression from PMID: 30772301	SOX2
PCNX4	SOX2 regulated genes in Keratinocyte Overexpression from PMID: 30772301	SOX2
ZFP64	SOX2 regulated genes in Keratinocyte Overexpression from PMID: 30772301	SOX2
TAF4B	SOX2 regulated genes in Keratinocyte Overexpression from PMID: 30772301	SOX2
SKA1	SOX2 regulated genes in Keratinocyte Overexpression from PMID: 30772301	SOX2
PROSER1	SOX2 regulated genes in Keratinocyte Overexpression from PMID: 30772301	SOX2
TTC6	SOX2 regulated genes in Keratinocyte Overexpression from PMID: 30772301	SOX2
NFX1	SOX2 regulated genes in Keratinocyte Overexpression from PMID: 30772301	SOX2
POMP	SOX2 regulated genes in Keratinocyte Overexpression from PMID: 30772301	SOX2
SCAF4	SOX2 regulated genes in Keratinocyte Overexpression from PMID: 30772301	SOX2
LINGO2	SOX2 regulated genes in Keratinocyte Overexpression from PMID: 30772301	SOX2
PPP6R2	SOX2 regulated genes in Keratinocyte Overexpression from PMID: 30772301	SOX2
SEC23B	SOX2 regulated genes in Keratinocyte Overexpression from PMID: 30772301	SOX2
RALY	SOX2 regulated genes in Keratinocyte Overexpression from PMID: 30772301	SOX2
PDE9A	SOX2 regulated genes in Keratinocyte Overexpression from PMID: 30772301	SOX2
HAUS6	SOX2 regulated genes in Keratinocyte Overexpression from PMID: 30772301	SOX2

ANKRD9	SOX2 regulated genes in Keratinocyte Overexpression from PMID: 30772301	SOX2
RBX1	SOX2 regulated genes in Keratinocyte Overexpression from PMID: 30772301	SOX2
MNAT1	SOX2 regulated genes in Keratinocyte Overexpression from PMID: 30772301	SOX2
BEX3	SOX2 regulated genes in Keratinocyte Overexpression from PMID: 30772301	SOX2
PSPC1	SOX2 regulated genes in Keratinocyte Overexpression from PMID: 30772301	SOX2
BMP7	SOX2 regulated genes in Keratinocyte Overexpression from PMID: 30772301	SOX2
RPS21	SOX2 regulated genes in Keratinocyte Overexpression from PMID: 30772301	SOX2
SCEL	SOX2 regulated genes in Keratinocyte Overexpression from PMID: 30772301	SOX2
PCDH8	SOX2 regulated genes in Keratinocyte Overexpression from PMID: 30772301	SOX2
PSIP1	SOX2 regulated genes in Keratinocyte Overexpression from PMID: 30772301	SOX2
NUFIP1	SOX2 regulated genes in Keratinocyte Overexpression from PMID: 30772301	SOX2
XKRX	SOX2 regulated genes in Keratinocyte Overexpression from PMID: 30772301	SOX2
CSTB	SOX2 regulated genes in Keratinocyte Overexpression from PMID: 30772301	SOX2
GTF3A	SOX2 regulated genes in Keratinocyte Overexpression from PMID: 30772301	SOX2
CDKN2B	SOX2 regulated genes in Keratinocyte Overexpression from PMID: 30772301	SOX2
CDC123	SOX2 regulated genes in Keratinocyte Overexpression from PMID: 30772301	SOX2
PAN3	SOX2 regulated genes in Keratinocyte Overexpression from PMID: 30772301	SOX2
HOPX	SOX2 regulated genes in Keratinocyte Overexpression from PMID: 30772301	SOX2
TP53INP2	SOX2 regulated genes in Keratinocyte Overexpression from PMID: 30772301	SOX2
WDR1	SOX2 regulated genes in Keratinocyte Overexpression from PMID: 30772301	SOX2
GID8	SOX2 regulated genes in Keratinocyte Overexpression from PMID: 30772301	SOX2
POLR3F	SOX2 regulated genes in Keratinocyte Overexpression from PMID: 30772301	SOX2
MAGEE2	SOX2 regulated genes in Keratinocyte Overexpression from PMID: 30772301	SOX2
MOK	SOX2 regulated genes in Keratinocyte Overexpression from PMID: 30772301	SOX2
HMGN5	SOX2 regulated genes in Keratinocyte Overexpression from PMID: 30772301	SOX2
NUP50	SOX2 regulated genes in Keratinocyte Overexpression from PMID: 30772301	SOX2
MRPL39	SOX2 regulated genes in Keratinocyte Overexpression from PMID: 30772301	SOX2
RPRD1B	SOX2 regulated genes in Keratinocyte Overexpression from PMID: 30772301	SOX2
DHX15	SOX2 regulated genes in Keratinocyte Overexpression from PMID: 30772301	SOX2
UPF2	SOX2 regulated genes in Keratinocyte Overexpression from PMID: 30772301	SOX2
CDC42BPB	SOX2 regulated genes in Keratinocyte Overexpression from PMID: 30772301	SOX2
RPS4X	SOX2 regulated genes in Keratinocyte Overexpression from PMID: 30772301	SOX2
CITED1	SOX2 regulated genes in Keratinocyte Overexpression from PMID: 30772301	SOX2
RNF152	SOX2 regulated genes in Keratinocyte Overexpression from PMID: 30772301	SOX2
RFC3	SOX2 regulated genes in Keratinocyte Overexpression from PMID: 30772301	SOX2
RBM17	SOX2 regulated genes in Keratinocyte Overexpression from PMID: 30772301	SOX2
SPATA2	SOX2 regulated genes in Keratinocyte Overexpression from PMID: 30772301	SOX2
KIN	SOX2 regulated genes in Keratinocyte Overexpression from PMID: 30772301	SOX2
MIS18BP1	SOX2 regulated genes in Keratinocyte Overexpression from PMID: 30772301	SOX2
PRR14L	SOX2 regulated genes in Keratinocyte Overexpression from PMID: 30772301	SOX2
HAUS1	SOX2 regulated genes in Keratinocyte Overexpression from PMID: 30772301	SOX2
CDC42EP1	SOX2 regulated genes in Keratinocyte Overexpression from PMID: 30772301	SOX2
CRYL1	SOX2 regulated genes in Keratinocyte Overexpression from PMID: 30772301	SOX2
AMER1	SOX2 regulated genes in Keratinocyte Overexpression from PMID: 30772301	SOX2
YY1	SOX2 regulated genes in Keratinocyte Overexpression from PMID: 30772301	SOX2
NOL12	SOX2 regulated genes in Keratinocyte Overexpression from PMID: 30772301	SOX2
PSMG1	SOX2 regulated genes in Keratinocyte Overexpression from PMID: 30772301	SOX2
SLC19A1	SOX2 regulated genes in Keratinocyte Overexpression from PMID: 30772301	SOX2
SLC25A17	SOX2 regulated genes in Keratinocyte Overexpression from PMID: 30772301	SOX2
BTG3	SOX2 regulated genes in Keratinocyte Overexpression from PMID: 30772301	SOX2
YBEY	SOX2 regulated genes in Keratinocyte Overexpression from PMID: 30772301	SOX2
ISM1	SOX2 regulated genes in Keratinocyte Overexpression from PMID: 30772301	SOX2
SSTR1	SOX2 regulated genes in Keratinocyte Overexpression from PMID: 30772301	SOX2
CTNNBL1	SOX2 regulated genes in Keratinocyte Overexpression from PMID: 30772301	SOX2
DSG3	SOX2 regulated genes in Keratinocyte Overexpression from PMID: 30772301	SOX2
BTBD7	SOX2 regulated genes in Keratinocyte Overexpression from PMID: 30772301	SOX2
TUBAL3	SOX2 regulated genes in Keratinocyte Overexpression from PMID: 30772301	SOX2
ATP5F1A	SOX2 regulated genes in Keratinocyte Overexpression from PMID: 30772301	SOX2
BTBD3	SOX2 regulated genes in Keratinocyte Overexpression from PMID: 30772301	SOX2

MAFF	SOX2 regulated genes in Keratinocyte Overexpression from PMID: 30772301	SOX2
DOK5	SOX2 regulated genes in Keratinocyte Overexpression from PMID: 30772301	SOX2
U2AF1	SOX2 regulated genes in Keratinocyte Overexpression from PMID: 30772301	SOX2
NUDT11	SOX2 regulated genes in Keratinocyte Overexpression from PMID: 30772301	SOX2
DLG3	SOX2 regulated genes in Keratinocyte Overexpression from PMID: 30772301	SOX2
BRWD1	SOX2 regulated genes in Keratinocyte Overexpression from PMID: 30772301	SOX2
MORC4	SOX2 regulated genes in Keratinocyte Overexpression from PMID: 30772301	SOX2
SMU1	SOX2 regulated genes in Keratinocyte Overexpression from PMID: 30772301	SOX2
TSR2	SOX2 regulated genes in Keratinocyte Overexpression from PMID: 30772301	SOX2
FANCM	SOX2 regulated genes in Keratinocyte Overexpression from PMID: 30772301	SOX2
DMRTA1	SOX2 regulated genes in Keratinocyte Overexpression from PMID: 30772301	SOX2
RBBP8NL	SOX2 regulated genes in Keratinocyte Overexpression from PMID: 30772301	SOX2
TASP1	SOX2 regulated genes in Keratinocyte Overexpression from PMID: 30772301	SOX2
TECPR2	SOX2 regulated genes in Keratinocyte Overexpression from PMID: 30772301	SOX2
TFAP2C	SOX2 regulated genes in Keratinocyte Overexpression from PMID: 30772301	SOX2
PRDM15	SOX2 regulated genes in Keratinocyte Overexpression from PMID: 30772301	SOX2
RUNX1	SOX2 regulated genes in Keratinocyte Overexpression from PMID: 30772301	SOX2
ERCC6L	SOX2 regulated genes in Keratinocyte Overexpression from PMID: 30772301	SOX2
CHODL	SOX2 regulated genes in Keratinocyte Overexpression from PMID: 30772301	SOX2
FGD1	SOX2 regulated genes in Keratinocyte Overexpression from PMID: 30772301	SOX2
RBM3	SOX2 regulated genes in Keratinocyte Overexpression from PMID: 30772301	SOX2
GPR137C	SOX2 regulated genes in Keratinocyte Overexpression from PMID: 30772301	SOX2
SYTL5	SOX2 regulated genes in Keratinocyte Overexpression from PMID: 30772301	SOX2
HSPH1	SOX2 regulated genes in Keratinocyte Overexpression from PMID: 30772301	SOX2
CDKL5	SOX2 regulated genes in Keratinocyte Overexpression from PMID: 30772301	SOX2
GLRX5	SOX2 regulated genes in Keratinocyte Overexpression from PMID: 30772301	SOX2
SAMD10	SOX2 regulated genes in Keratinocyte Overexpression from PMID: 30772301	SOX2
KIF4A	SOX2 regulated genes in Keratinocyte Overexpression from PMID: 30772301	SOX2
LRR1	SOX2 regulated genes in Keratinocyte Overexpression from PMID: 30772301	SOX2
POLE2	SOX2 regulated genes in Keratinocyte Overexpression from PMID: 30772301	SOX2
EPG5	SOX2 regulated genes in Keratinocyte Overexpression from PMID: 30772301	SOX2
DCUN1D4	SOX2 regulated genes in Keratinocyte Overexpression from PMID: 30772301	SOX2
SEC61A2	SOX2 regulated genes in Keratinocyte Overexpression from PMID: 30772301	SOX2
PKNOX1	SOX2 regulated genes in Keratinocyte Overexpression from PMID: 30772301	SOX2
FRMD4A	SOX2 regulated genes in Keratinocyte Overexpression from PMID: 30772301	SOX2
CDH22	SOX2 regulated genes in Keratinocyte Overexpression from PMID: 30772301	SOX2
RBM11	SOX2 regulated genes in Keratinocyte Overexpression from PMID: 30772301	SOX2
SEH1L	SOX2 regulated genes in Keratinocyte Overexpression from PMID: 30772301	SOX2
FAM110A	SOX2 regulated genes in Keratinocyte Overexpression from PMID: 30772301	SOX2
C21orf58	SOX2 regulated genes in Keratinocyte Overexpression from PMID: 30772301	SOX2
HS3ST1	SOX2 regulated genes in Keratinocyte Overexpression from PMID: 30772301	SOX2
PFDN4	SOX2 regulated genes in Keratinocyte Overexpression from PMID: 30772301	SOX2
SEPHS1	SOX2 regulated genes in Keratinocyte Overexpression from PMID: 30772301	SOX2
TMX4	SOX2 regulated genes in Keratinocyte Overexpression from PMID: 30772301	SOX2
SPATA13	SOX2 regulated genes in Keratinocyte Overexpression from PMID: 30772301	SOX2
RCL1	SOX2 regulated genes in Keratinocyte Overexpression from PMID: 30772301	SOX2
BMP4	SOX2 regulated genes in Keratinocyte Overexpression from PMID: 30772301	SOX2
DEPDC5	SOX2 regulated genes in Keratinocyte Overexpression from PMID: 30772301	SOX2
PCNX1	SOX2 regulated genes in Keratinocyte Overexpression from PMID: 30772301	SOX2
ETS2	SOX2 regulated genes in Keratinocyte Overexpression from PMID: 30772301	SOX2
TPGS2	SOX2 regulated genes in Keratinocyte Overexpression from PMID: 30772301	SOX2
CDK5RAP1	SOX2 regulated genes in Keratinocyte Overexpression from PMID: 30772301	SOX2
LTN1	SOX2 regulated genes in Keratinocyte Overexpression from PMID: 30772301	SOX2
CFAP61	SOX2 regulated genes in Keratinocyte Overexpression from PMID: 30772301	SOX2
DONSON	SOX2 regulated genes in Keratinocyte Overexpression from PMID: 30772301	SOX2
DIAPH3	SOX2 regulated genes in Keratinocyte Overexpression from PMID: 30772301	SOX2
KCTD1	SOX2 regulated genes in Keratinocyte Overexpression from PMID: 30772301	SOX2
LSM14B	SOX2 regulated genes in Keratinocyte Overexpression from PMID: 30772301	SOX2
LHFPL6	SOX2 regulated genes in Keratinocyte Overexpression from PMID: 30772301	SOX2

PSMG2	SOX2 regulated genes in Keratinocyte Overexpression from PMID: 30772301	SOX2
ADRM1	SOX2 regulated genes in Keratinocyte Overexpression from PMID: 30772301	SOX2
MIPEP	SOX2 regulated genes in Keratinocyte Overexpression from PMID: 30772301	SOX2
GTSE1	SOX2 regulated genes in Keratinocyte Overexpression from PMID: 30772301	SOX2
SLC37A1	SOX2 regulated genes in Keratinocyte Overexpression from PMID: 30772301	SOX2
TGIF1	SOX2 regulated genes in Keratinocyte Overexpression from PMID: 30772301	SOX2
RNF17	SOX2 regulated genes in Keratinocyte Overexpression from PMID: 30772301	SOX2
CENPC	SOX2 regulated genes in Keratinocyte Overexpression from PMID: 30772301	SOX2
PSMC6	SOX2 regulated genes in Keratinocyte Overexpression from PMID: 30772301	SOX2
AHCY	SOX2 regulated genes in Keratinocyte Overexpression from PMID: 30772301	SOX2
MYO5B	SOX2 regulated genes in Keratinocyte Overexpression from PMID: 30772301	SOX2
NPC1	SOX2 regulated genes in Keratinocyte Overexpression from PMID: 30772301	SOX2
GRPEL1	SOX2 regulated genes in Keratinocyte Overexpression from PMID: 30772301	SOX2
PDXP	SOX2 regulated genes in Keratinocyte Overexpression from PMID: 30772301	SOX2
RNGTT	SOX2 regulated genes in Keratinocyte Overexpression from PMID: 30772301	SOX2
ESF1	SOX2 regulated genes in Keratinocyte Overexpression from PMID: 30772301	SOX2
QRFPR	SOX2 regulated genes in Keratinocyte Overexpression from PMID: 30772301	SOX2
THTPA	SOX2 regulated genes in Keratinocyte Overexpression from PMID: 30772301	SOX2
NKX1-2	SOX2 regulated genes in Keratinocyte Overexpression from PMID: 30772301	SOX2
C14orf93	SOX2 regulated genes in Keratinocyte Overexpression from PMID: 30772301	SOX2
MYO1D	SOX2 regulated genes in Keratinocyte Overexpression from PMID: 30772301	SOX2
LASP1	SOX2 regulated genes in Keratinocyte Overexpression from PMID: 30772301	SOX2
DLGAP4	SOX2 regulated genes in Keratinocyte Overexpression from PMID: 30772301	SOX2
BCOR	SOX2 regulated genes in Keratinocyte Overexpression from PMID: 30772301	SOX2
CYP17A1	SOX2 regulated genes in Keratinocyte Overexpression from PMID: 30772301	SOX2
FAM234B	SOX2 regulated genes in Keratinocyte Overexpression from PMID: 30772301	SOX2
GPAT3	SOX2 regulated genes in Keratinocyte Overexpression from PMID: 30772301	SOX2
TNKS2	SOX2 regulated genes in Keratinocyte Overexpression from PMID: 30772301	SOX2
EFCAB11	SOX2 regulated genes in Keratinocyte Overexpression from PMID: 30772301	SOX2
GPATCH8	SOX2 regulated genes in Keratinocyte Overexpression from PMID: 30772301	SOX2
SUPT16H	SOX2 regulated genes in Keratinocyte Overexpression from PMID: 30772301	SOX2
ZNF407	SOX2 regulated genes in Keratinocyte Overexpression from PMID: 30772301	SOX2
ATP5MF-PTCD1	SOX2 regulated genes in Keratinocyte Overexpression from PMID: 30772301	SOX2
TEX30	SOX2 regulated genes in Keratinocyte Overexpression from PMID: 30772301	SOX2
GRIA3	SOX2 regulated genes in Keratinocyte Overexpression from PMID: 30772301	SOX2
COPS4	SOX2 regulated genes in Keratinocyte Overexpression from PMID: 30772301	SOX2
TSSK4	SOX2 regulated genes in Keratinocyte Overexpression from PMID: 30772301	SOX2
INCENP	SOX2 regulated genes in Keratinocyte Overexpression from PMID: 30772301	SOX2
LZTS1	SOX2 regulated genes in Keratinocyte Overexpression from PMID: 30772301	SOX2
SETBP1	SOX2 regulated genes in Keratinocyte Overexpression from PMID: 30772301	SOX2
RPL36A	SOX2 regulated genes in Keratinocyte Overexpression from PMID: 30772301	SOX2
PLPP2	SOX2 regulated genes in Keratinocyte Overexpression from PMID: 30772301	SOX2
NF2	SOX2 regulated genes in Keratinocyte Overexpression from PMID: 30772301	SOX2
C18orf32	SOX2 regulated genes in Keratinocyte Overexpression from PMID: 30772301	SOX2
ABTB2	SOX2 regulated genes in Keratinocyte Overexpression from PMID: 30772301	SOX2
SERPINB13	SOX2 regulated genes in Keratinocyte Overexpression from PMID: 30772301	SOX2
MAP3K7	SOX2 regulated genes in Keratinocyte Overexpression from PMID: 30772301	SOX2
FRAT1	SOX2 regulated genes in Keratinocyte Overexpression from PMID: 30772301	SOX2
RNF167	SOX2 regulated genes in Keratinocyte Overexpression from PMID: 30772301	SOX2
TSPAN7	SOX2 regulated genes in Keratinocyte Overexpression from PMID: 30772301	SOX2
GUCD1	SOX2 regulated genes in Keratinocyte Overexpression from PMID: 30772301	SOX2
GSTCD	SOX2 regulated genes in Keratinocyte Overexpression from PMID: 30772301	SOX2
ZBTB43	SOX2 regulated genes in Keratinocyte Overexpression from PMID: 30772301	SOX2
PPP2R2A	SOX2 regulated genes in Keratinocyte Overexpression from PMID: 30772301	SOX2
NUDCD3	SOX2 regulated genes in Keratinocyte Overexpression from PMID: 30772301	SOX2
SERPINB11	SOX2 regulated genes in Keratinocyte Overexpression from PMID: 30772301	SOX2
SRXN1	SOX2 regulated genes in Keratinocyte Overexpression from PMID: 30772301	SOX2
LDLRAD3	SOX2 regulated genes in Keratinocyte Overexpression from PMID: 30772301	SOX2
NRBF2	SOX2 regulated genes in Keratinocyte Overexpression from PMID: 30772301	SOX2

MIEF1	SOX2 regulated genes in Keratinocyte Overexpression from PMID: 30772301	SOX2
LIG3	SOX2 regulated genes in Keratinocyte Overexpression from PMID: 30772301	SOX2
DLST	SOX2 regulated genes in Keratinocyte Overexpression from PMID: 30772301	SOX2
ASPHD2	SOX2 regulated genes in Keratinocyte Overexpression from PMID: 30772301	SOX2
SPATC1L	SOX2 regulated genes in Keratinocyte Overexpression from PMID: 30772301	SOX2
KIF1C	SOX2 regulated genes in Keratinocyte Overexpression from PMID: 30772301	SOX2
PPIL2	SOX2 regulated genes in Keratinocyte Overexpression from PMID: 30772301	SOX2
PPP1R26	SOX2 regulated genes in Keratinocyte Overexpression from PMID: 30772301	SOX2
MED4	SOX2 regulated genes in Keratinocyte Overexpression from PMID: 30772301	SOX2
ZFYVE19	SOX2 regulated genes in Keratinocyte Overexpression from PMID: 30772301	SOX2
FRMD6	SOX2 regulated genes in Keratinocyte Overexpression from PMID: 30772301	SOX2
PDZD8	SOX2 regulated genes in Keratinocyte Overexpression from PMID: 30772301	SOX2
BACH2	SOX2 regulated genes in Keratinocyte Overexpression from PMID: 30772301	SOX2
AQP11	SOX2 regulated genes in Keratinocyte Overexpression from PMID: 30772301	SOX2
AP1S1	SOX2 regulated genes in Keratinocyte Overexpression from PMID: 30772301	SOX2
ANO3	SOX2 regulated genes in Keratinocyte Overexpression from PMID: 30772301	SOX2
CENPP	SOX2 regulated genes in Keratinocyte Overexpression from PMID: 30772301	SOX2
POLR2M	SOX2 regulated genes in Keratinocyte Overexpression from PMID: 30772301	SOX2
CEP44	SOX2 regulated genes in Keratinocyte Overexpression from PMID: 30772301	SOX2
ZNRF2	SOX2 regulated genes in Keratinocyte Overexpression from PMID: 30772301	SOX2
C9orf40	SOX2 regulated genes in Keratinocyte Overexpression from PMID: 30772301	SOX2
UBR7	SOX2 regulated genes in Keratinocyte Overexpression from PMID: 30772301	SOX2
TPD52L1	SOX2 regulated genes in Keratinocyte Overexpression from PMID: 30772301	SOX2
RSL1D1	SOX2 regulated genes in Keratinocyte Overexpression from PMID: 30772301	SOX2
LSM5	SOX2 regulated genes in Keratinocyte Overexpression from PMID: 30772301	SOX2
IRAK1BP1	SOX2 regulated genes in Keratinocyte Overexpression from PMID: 30772301	SOX2
ABT1	SOX2 regulated genes in Keratinocyte Overexpression from PMID: 30772301	SOX2
TSTD2	SOX2 regulated genes in Keratinocyte Overexpression from PMID: 30772301	SOX2
CLDN10	SOX2 regulated genes in Keratinocyte Overexpression from PMID: 30772301	SOX2
TOR2A	SOX2 regulated genes in Keratinocyte Overexpression from PMID: 30772301	SOX2
MAPK1	SOX2 regulated genes in Keratinocyte Overexpression from PMID: 30772301	SOX2
VPS16	SOX2 regulated genes in Keratinocyte Overexpression from PMID: 30772301	SOX2
HAUS2	SOX2 regulated genes in Keratinocyte Overexpression from PMID: 30772301	SOX2
BUB3	SOX2 regulated genes in Keratinocyte Overexpression from PMID: 30772301	SOX2
PAK6	SOX2 regulated genes in Keratinocyte Overexpression from PMID: 30772301	SOX2
RCE1	SOX2 regulated genes in Keratinocyte Overexpression from PMID: 30772301	SOX2
YBX3	SOX2 regulated genes in Keratinocyte Overexpression from PMID: 30772301	SOX2
MORC2	SOX2 regulated genes in Keratinocyte Overexpression from PMID: 30772301	SOX2
PALM	SOX2 regulated genes in Keratinocyte Overexpression from PMID: 30772301	SOX2
RHOD	SOX2 regulated genes in Keratinocyte Overexpression from PMID: 30772301	SOX2
METTL14	SOX2 regulated genes in Keratinocyte Overexpression from PMID: 30772301	SOX2
NECAP1	SOX2 regulated genes in Keratinocyte Overexpression from PMID: 30772301	SOX2
AAR2	SOX2 regulated genes in Keratinocyte Overexpression from PMID: 30772301	SOX2
RBM26	SOX2 regulated genes in Keratinocyte Overexpression from PMID: 30772301	SOX2
UCHL3	SOX2 regulated genes in Keratinocyte Overexpression from PMID: 30772301	SOX2
BCL2L14	SOX2 regulated genes in Keratinocyte Overexpression from PMID: 30772301	SOX2
NONO	SOX2 regulated genes in Keratinocyte Overexpression from PMID: 30772301	SOX2
USP14	SOX2 regulated genes in Keratinocyte Overexpression from PMID: 30772301	SOX2
EDC3	SOX2 regulated genes in Keratinocyte Overexpression from PMID: 30772301	SOX2
ETV4	SOX2 regulated genes in Keratinocyte Overexpression from PMID: 30772301	SOX2
NKX3-1	SOX2 regulated genes in Keratinocyte Overexpression from PMID: 30772301	SOX2
BCL2L1	SOX2 regulated genes in Keratinocyte Overexpression from PMID: 30772301	SOX2
MAGOHB	SOX2 regulated genes in Keratinocyte Overexpression from PMID: 30772301	SOX2
UBE2K	SOX2 regulated genes in Keratinocyte Overexpression from PMID: 30772301	SOX2
HARBI1	SOX2 regulated genes in Keratinocyte Overexpression from PMID: 30772301	SOX2
CAPN15	SOX2 regulated genes in Keratinocyte Overexpression from PMID: 30772301	SOX2
RPL23	SOX2 regulated genes in Keratinocyte Overexpression from PMID: 30772301	SOX2
LRP6	SOX2 regulated genes in Keratinocyte Overexpression from PMID: 30772301	SOX2
DSC1	SOX2 regulated genes in Keratinocyte Overexpression from PMID: 30772301	SOX2

PAPSS1	SOX2 regulated genes in Keratinocyte Overexpression from PMID: 30772301	SOX2
RBBP7	SOX2 regulated genes in Keratinocyte Overexpression from PMID: 30772301	SOX2
C7orf26	SOX2 regulated genes in Keratinocyte Overexpression from PMID: 30772301	SOX2
ID1	SOX2 regulated genes in Keratinocyte Overexpression from PMID: 30772301	SOX2
DSG2	SOX2 regulated genes in Keratinocyte Overexpression from PMID: 30772301	SOX2
MCM3	SOX2 regulated genes in Keratinocyte Overexpression from PMID: 30772301	SOX2
SREBF2	SOX2 regulated genes in Keratinocyte Overexpression from PMID: 30772301	SOX2
DNTTIP1	SOX2 regulated genes in Keratinocyte Overexpression from PMID: 30772301	SOX2
ZGPAT	SOX2 regulated genes in Keratinocyte Overexpression from PMID: 30772301	SOX2
RBM39	SOX2 regulated genes in Keratinocyte Overexpression from PMID: 30772301	SOX2
NCOA5	SOX2 regulated genes in Keratinocyte Overexpression from PMID: 30772301	SOX2
POLR2F	SOX2 regulated genes in Keratinocyte Overexpression from PMID: 30772301	SOX2
ITFG2	SOX2 regulated genes in Keratinocyte Overexpression from PMID: 30772301	SOX2
DUSP4	SOX2 regulated genes in Keratinocyte Overexpression from PMID: 30772301	SOX2
MCM8	SOX2 regulated genes in Keratinocyte Overexpression from PMID: 30772301	SOX2
RAB11FIP1	SOX2 regulated genes in Keratinocyte Overexpression from PMID: 30772301	SOX2
NEDD9	SOX2 regulated genes in Keratinocyte Overexpression from PMID: 30772301	SOX2
MIOS	SOX2 regulated genes in Keratinocyte Overexpression from PMID: 30772301	SOX2
RUNX2	SOX2 regulated genes in Keratinocyte Overexpression from PMID: 30772301	SOX2
C10orf88	SOX2 regulated genes in Keratinocyte Overexpression from PMID: 30772301	SOX2
TRIM27	SOX2 regulated genes in Keratinocyte Overexpression from PMID: 30772301	SOX2
SOX21	SOX2 regulated genes in Keratinocyte Overexpression from PMID: 30772301	SOX2
GRIN3B	SOX2 regulated genes in Keratinocyte Overexpression from PMID: 30772301	SOX2
NIPSNAP1	SOX2 regulated genes in Keratinocyte Overexpression from PMID: 30772301	SOX2
MRPL1	SOX2 regulated genes in Keratinocyte Overexpression from PMID: 30772301	SOX2
KDM5A	SOX2 regulated genes in Keratinocyte Overexpression from PMID: 30772301	SOX2
PSMB5	SOX2 regulated genes in Keratinocyte Overexpression from PMID: 30772301	SOX2
RPA3	SOX2 regulated genes in Keratinocyte Overexpression from PMID: 30772301	SOX2
ASB6	SOX2 regulated genes in Keratinocyte Overexpression from PMID: 30772301	SOX2
CAAP1	SOX2 regulated genes in Keratinocyte Overexpression from PMID: 30772301	SOX2
TXNDC17	SOX2 regulated genes in Keratinocyte Overexpression from PMID: 30772301	SOX2
BRD3	SOX2 regulated genes in Keratinocyte Overexpression from PMID: 30772301	SOX2
IPPK	SOX2 regulated genes in Keratinocyte Overexpression from PMID: 30772301	SOX2
TYMS	SOX2 regulated genes in Keratinocyte Overexpression from PMID: 30772301	SOX2
NAPG	SOX2 regulated genes in Keratinocyte Overexpression from PMID: 30772301	SOX2
ESCO1	SOX2 regulated genes in Keratinocyte Overexpression from PMID: 30772301	SOX2
THOC1	SOX2 regulated genes in Keratinocyte Overexpression from PMID: 30772301	SOX2
FAM186B	SOX2 regulated genes in Keratinocyte Overexpression from PMID: 30772301	SOX2
STYK1	SOX2 regulated genes in Keratinocyte Overexpression from PMID: 30772301	SOX2
FAM98B	SOX2 regulated genes in Keratinocyte Overexpression from PMID: 30772301	SOX2
SPECC1L	SOX2 regulated genes in Keratinocyte Overexpression from PMID: 30772301	SOX2
DGCR2	SOX2 regulated genes in Keratinocyte Overexpression from PMID: 30772301	SOX2
SRGAP1	SOX2 regulated genes in Keratinocyte Overexpression from PMID: 30772301	SOX2
SLC38A2	SOX2 regulated genes in Keratinocyte Overexpression from PMID: 30772301	SOX2
PTAR1	SOX2 regulated genes in Keratinocyte Overexpression from PMID: 30772301	SOX2
MRPL41	SOX2 regulated genes in Keratinocyte Overexpression from PMID: 30772301	SOX2
GRID2	SOX2 regulated genes in Keratinocyte Overexpression from PMID: 30772301	SOX2
FLYWCH2	SOX2 regulated genes in Keratinocyte Overexpression from PMID: 30772301	SOX2
CENPQ	SOX2 regulated genes in Keratinocyte Overexpression from PMID: 30772301	SOX2
NUDT16L1	SOX2 regulated genes in Keratinocyte Overexpression from PMID: 30772301	SOX2
NUPR2	SOX2 regulated genes in Keratinocyte Overexpression from PMID: 30772301	SOX2
ING2	SOX2 regulated genes in Keratinocyte Overexpression from PMID: 30772301	SOX2
TADA2B	SOX2 regulated genes in Keratinocyte Overexpression from PMID: 30772301	SOX2
CPLX3	SOX2 regulated genes in Keratinocyte Overexpression from PMID: 30772301	SOX2
EIF6	SOX2 regulated genes in Keratinocyte Overexpression from PMID: 30772301	SOX2
CSE1L	SOX2 regulated genes in Keratinocyte Overexpression from PMID: 30772301	SOX2
PRNP	SOX2 regulated genes in Keratinocyte Overexpression from PMID: 30772301	SOX2
UROS	SOX2 regulated genes in Keratinocyte Overexpression from PMID: 30772301	SOX2
CCDC120	SOX2 regulated genes in Keratinocyte Overexpression from PMID: 30772301	SOX2

FKBP4	SOX2 regulated genes in Keratinocyte Overexpression from PMID: 30772301	SOX2
ARHGAP23	SOX2 regulated genes in Keratinocyte Overexpression from PMID: 30772301	SOX2
CXCL6	SOX2 regulated genes in Keratinocyte Overexpression from PMID: 30772301	SOX2
TRABD	SOX2 regulated genes in Keratinocyte Overexpression from PMID: 30772301	SOX2
RIOK1	SOX2 regulated genes in Keratinocyte Overexpression from PMID: 30772301	SOX2
HCFC1	SOX2 regulated genes in Keratinocyte Overexpression from PMID: 30772301	SOX2
SLC52A1	SOX2 regulated genes in Keratinocyte Overexpression from PMID: 30772301	SOX2
CRTAC1	SOX2 regulated genes in Keratinocyte Overexpression from PMID: 30772301	SOX2
APEX2	SOX2 regulated genes in Keratinocyte Overexpression from PMID: 30772301	SOX2
RASEF	SOX2 regulated genes in Keratinocyte Overexpression from PMID: 30772301	SOX2
EGR3	SOX2 regulated genes in Keratinocyte Overexpression from PMID: 30772301	SOX2
TRIM29	SOX2 regulated genes in Keratinocyte Overexpression from PMID: 30772301	SOX2
DKK1	SOX2 regulated genes in Keratinocyte Overexpression from PMID: 30772301	SOX2
SAMD8	SOX2 regulated genes in Keratinocyte Overexpression from PMID: 30772301	SOX2
NUP58	SOX2 regulated genes in Keratinocyte Overexpression from PMID: 30772301	SOX2
SIVA1	SOX2 regulated genes in Keratinocyte Overexpression from PMID: 30772301	SOX2
LRFN4	SOX2 regulated genes in Keratinocyte Overexpression from PMID: 30772301	SOX2
UMODL1	SOX2 regulated genes in Keratinocyte Overexpression from PMID: 30772301	SOX2
POLR2E	SOX2 regulated genes in Keratinocyte Overexpression from PMID: 30772301	SOX2
ACOT8	SOX2 regulated genes in Keratinocyte Overexpression from PMID: 30772301	SOX2
NEDD8	SOX2 regulated genes in Keratinocyte Overexpression from PMID: 30772301	SOX2
REM2	SOX2 regulated genes in Keratinocyte Overexpression from PMID: 30772301	SOX2
ZNF503	SOX2 regulated genes in Keratinocyte Overexpression from PMID: 30772301	SOX2
PRICKLE3	SOX2 regulated genes in Keratinocyte Overexpression from PMID: 30772301	SOX2
RHOT2	SOX2 regulated genes in Keratinocyte Overexpression from PMID: 30772301	SOX2
C15orf65	SOX2 regulated genes in Keratinocyte Overexpression from PMID: 30772301	SOX2
TFAM	SOX2 regulated genes in Keratinocyte Overexpression from PMID: 30772301	SOX2
SLC26A8	SOX2 regulated genes in Keratinocyte Overexpression from PMID: 30772301	SOX2
PACSIN1	SOX2 regulated genes in Keratinocyte Overexpression from PMID: 30772301	SOX2
FANCF	SOX2 regulated genes in Keratinocyte Overexpression from PMID: 30772301	SOX2
SH3RF1	SOX2 regulated genes in Keratinocyte Overexpression from PMID: 30772301	SOX2
OSBP	SOX2 regulated genes in Keratinocyte Overexpression from PMID: 30772301	SOX2
NUP88	SOX2 regulated genes in Keratinocyte Overexpression from PMID: 30772301	SOX2
PCID2	SOX2 regulated genes in Keratinocyte Overexpression from PMID: 30772301	SOX2
SLCO4A1	SOX2 regulated genes in Keratinocyte Overexpression from PMID: 30772301	SOX2
LMX1B	SOX2 regulated genes in Keratinocyte Overexpression from PMID: 30772301	SOX2
SMC2	SOX2 regulated genes in Keratinocyte Overexpression from PMID: 30772301	SOX2
FRMD8	SOX2 regulated genes in Keratinocyte Overexpression from PMID: 30772301	SOX2
CWH43	SOX2 regulated genes in Keratinocyte Overexpression from PMID: 30772301	SOX2
FNDC11	SOX2 regulated genes in Keratinocyte Overexpression from PMID: 30772301	SOX2
ABCB7	SOX2 regulated genes in Keratinocyte Overexpression from PMID: 30772301	SOX2
LPIN2	SOX2 regulated genes in Keratinocyte Overexpression from PMID: 30772301	SOX2
TMEM251	SOX2 regulated genes in Keratinocyte Overexpression from PMID: 30772301	SOX2
MIF	SOX2 regulated genes in Keratinocyte Overexpression from PMID: 30772301	SOX2
MRPS2	SOX2 regulated genes in Keratinocyte Overexpression from PMID: 30772301	SOX2
TERT	SOX2 regulated genes in Keratinocyte Overexpression from PMID: 30772301	SOX2
THNSL1	SOX2 regulated genes in Keratinocyte Overexpression from PMID: 30772301	SOX2
AK7	SOX2 regulated genes in Keratinocyte Overexpression from PMID: 30772301	SOX2
RBMX	SOX2 regulated genes in Keratinocyte Overexpression from PMID: 30772301	SOX2
SLC30A9	SOX2 regulated genes in Keratinocyte Overexpression from PMID: 30772301	SOX2
CHIC2	SOX2 regulated genes in Keratinocyte Overexpression from PMID: 30772301	SOX2
SLC44A1	SOX2 regulated genes in Keratinocyte Overexpression from PMID: 30772301	SOX2
NGDN	SOX2 regulated genes in Keratinocyte Overexpression from PMID: 30772301	SOX2
NEU3	SOX2 regulated genes in Keratinocyte Overexpression from PMID: 30772301	SOX2
DSG1	SOX2 regulated genes in Keratinocyte Overexpression from PMID: 30772301	SOX2
TAF1D	SOX2 regulated genes in Keratinocyte Overexpression from PMID: 30772301	SOX2
TMEM144	SOX2 regulated genes in Keratinocyte Overexpression from PMID: 30772301	SOX2
NSFL1C	SOX2 regulated genes in Keratinocyte Overexpression from PMID: 30772301	SOX2
TTC5	SOX2 regulated genes in Keratinocyte Overexpression from PMID: 30772301	SOX2

MRPL36	SOX2 regulated genes in Keratinocyte Overexpression from PMID: 30772301	SOX2
NCLN	SOX2 regulated genes in Keratinocyte Overexpression from PMID: 30772301	SOX2
RBM20	SOX2 regulated genes in Keratinocyte Overexpression from PMID: 30772301	SOX2
FOXC1	SOX2 regulated genes in Keratinocyte Overexpression from PMID: 30772301	SOX2
URM1	SOX2 regulated genes in Keratinocyte Overexpression from PMID: 30772301	SOX2
TMEM255A	SOX2 regulated genes in Keratinocyte Overexpression from PMID: 30772301	SOX2
PRPF19	SOX2 regulated genes in Keratinocyte Overexpression from PMID: 30772301	SOX2
CCDC116	SOX2 regulated genes in Keratinocyte Overexpression from PMID: 30772301	SOX2
RFXAP	SOX2 regulated genes in Keratinocyte Overexpression from PMID: 30772301	SOX2
TDP1	SOX2 regulated genes in Keratinocyte Overexpression from PMID: 30772301	SOX2
LEPROTL1	SOX2 regulated genes in Keratinocyte Overexpression from PMID: 30772301	SOX2
RAB11FIP2	SOX2 regulated genes in Keratinocyte Overexpression from PMID: 30772301	SOX2
PDCL	SOX2 regulated genes in Keratinocyte Overexpression from PMID: 30772301	SOX2
TDRD3	SOX2 regulated genes in Keratinocyte Overexpression from PMID: 30772301	SOX2
RND2	SOX2 regulated genes in Keratinocyte Overexpression from PMID: 30772301	SOX2
TRIM68	SOX2 regulated genes in Keratinocyte Overexpression from PMID: 30772301	SOX2
BMPER	SOX2 regulated genes in Keratinocyte Overexpression from PMID: 30772301	SOX2
WFIKKN1	SOX2 regulated genes in Keratinocyte Overexpression from PMID: 30772301	SOX2
MAPK11	SOX2 regulated genes in Keratinocyte Overexpression from PMID: 30772301	SOX2
ZNF219	SOX2 regulated genes in Keratinocyte Overexpression from PMID: 30772301	SOX2
ARHGAP24	SOX2 regulated genes in Keratinocyte Overexpression from PMID: 30772301	SOX2
MUC15	SOX2 regulated genes in Keratinocyte Overexpression from PMID: 30772301	SOX2
BCORL1	SOX2 regulated genes in Keratinocyte Overexpression from PMID: 30772301	SOX2
NMRK1	SOX2 regulated genes in Keratinocyte Overexpression from PMID: 30772301	SOX2
EVC2	SOX2 regulated genes in Keratinocyte Overexpression from PMID: 30772301	SOX2
ARPC1A	SOX2 regulated genes in Keratinocyte Overexpression from PMID: 30772301	SOX2
SHMT2	SOX2 regulated genes in Keratinocyte Overexpression from PMID: 30772301	SOX2
CDC5L	SOX2 regulated genes in Keratinocyte Overexpression from PMID: 30772301	SOX2
TLE6	SOX2 regulated genes in Keratinocyte Overexpression from PMID: 30772301	SOX2
TEX9	SOX2 regulated genes in Keratinocyte Overexpression from PMID: 30772301	SOX2
PRPSAP2	SOX2 regulated genes in Keratinocyte Overexpression from PMID: 30772301	SOX2
ARGLU1	SOX2 regulated genes in Keratinocyte Overexpression from PMID: 30772301	SOX2
FASTKD3	SOX2 regulated genes in Keratinocyte Overexpression from PMID: 30772301	SOX2
SLTM	SOX2 regulated genes in Keratinocyte Overexpression from PMID: 30772301	SOX2
PIM1	SOX2 regulated genes in Keratinocyte Overexpression from PMID: 30772301	SOX2
PLAA	SOX2 regulated genes in Keratinocyte Overexpression from PMID: 30772301	SOX2
UBQLN1	SOX2 regulated genes in Keratinocyte Overexpression from PMID: 30772301	SOX2
ORMDL3	SOX2 regulated genes in Keratinocyte Overexpression from PMID: 30772301	SOX2
TBRG4	SOX2 regulated genes in Keratinocyte Overexpression from PMID: 30772301	SOX2
PORCN	SOX2 regulated genes in Keratinocyte Overexpression from PMID: 30772301	SOX2
SNRPD1	SOX2 regulated genes in Keratinocyte Overexpression from PMID: 30772301	SOX2
PSMF1	SOX2 regulated genes in Keratinocyte Overexpression from PMID: 30772301	SOX2
MRPL50	SOX2 regulated genes in Keratinocyte Overexpression from PMID: 30772301	SOX2
SLC10A4	SOX2 regulated genes in Keratinocyte Overexpression from PMID: 30772301	SOX2
AIMP2	SOX2 regulated genes in Keratinocyte Overexpression from PMID: 30772301	SOX2
TRMT11	SOX2 regulated genes in Keratinocyte Overexpression from PMID: 30772301	SOX2
GATAD1	SOX2 regulated genes in Keratinocyte Overexpression from PMID: 30772301	SOX2
AARS2	SOX2 regulated genes in Keratinocyte Overexpression from PMID: 30772301	SOX2
RDM1	SOX2 regulated genes in Keratinocyte Overexpression from PMID: 30772301	SOX2
CBFA2T2	SOX2 regulated genes in Keratinocyte Overexpression from PMID: 30772301	SOX2
UBE2A	SOX2 regulated genes in Keratinocyte Overexpression from PMID: 30772301	SOX2
C9orf85	SOX2 regulated genes in Keratinocyte Overexpression from PMID: 30772301	SOX2
COL4A5	SOX2 regulated genes in Keratinocyte Overexpression from PMID: 30772301	SOX2
GSDMA	SOX2 regulated genes in Keratinocyte Overexpression from PMID: 30772301	SOX2
PRIM2	SOX2 regulated genes in Keratinocyte Overexpression from PMID: 30772301	SOX2
CBX6	SOX2 regulated genes in Keratinocyte Overexpression from PMID: 30772301	SOX2
RPGR	SOX2 regulated genes in Keratinocyte Overexpression from PMID: 30772301	SOX2
P2RX5	SOX2 regulated genes in Keratinocyte Overexpression from PMID: 30772301	SOX2
NUDT15	SOX2 regulated genes in Keratinocyte Overexpression from PMID: 30772301	SOX2

PLK4	SOX2 regulated genes in Keratinocyte Overexpression from PMID: 30772301	SOX2
AXIN1	SOX2 regulated genes in Keratinocyte Overexpression from PMID: 30772301	SOX2
ARHGAP1	SOX2 regulated genes in Keratinocyte Overexpression from PMID: 30772301	SOX2
RPS24	SOX2 regulated genes in Keratinocyte Overexpression from PMID: 30772301	SOX2
BLMH	SOX2 regulated genes in Keratinocyte Overexpression from PMID: 30772301	SOX2
TNC	SOX2 regulated genes in Keratinocyte Overexpression from PMID: 30772301	SOX2
TUG1	SOX2 regulated genes in Keratinocyte Overexpression from PMID: 30772301	SOX2
NATD1	SOX2 regulated genes in Keratinocyte Overexpression from PMID: 30772301	SOX2
RNF20	SOX2 regulated genes in Keratinocyte Overexpression from PMID: 30772301	SOX2
HMG20A	SOX2 regulated genes in Keratinocyte Overexpression from PMID: 30772301	SOX2
AVPI1	SOX2 regulated genes in Keratinocyte Overexpression from PMID: 30772301	SOX2
MRPL49	SOX2 regulated genes in Keratinocyte Overexpression from PMID: 30772301	SOX2
TSPAN9	SOX2 regulated genes in Keratinocyte Overexpression from PMID: 30772301	SOX2
MISP	SOX2 regulated genes in Keratinocyte Overexpression from PMID: 30772301	SOX2
UPF3A	SOX2 regulated genes in Keratinocyte Overexpression from PMID: 30772301	SOX2
TMTC1	SOX2 regulated genes in Keratinocyte Overexpression from PMID: 30772301	SOX2
ANLN	SOX2 regulated genes in Keratinocyte Overexpression from PMID: 30772301	SOX2
PINX1	SOX2 regulated genes in Keratinocyte Overexpression from PMID: 30772301	SOX2
EXPH5	SOX2 regulated genes in Keratinocyte Overexpression from PMID: 30772301	SOX2
POLR3H	SOX2 regulated genes in Keratinocyte Overexpression from PMID: 30772301	SOX2
ZSWIM1	SOX2 regulated genes in Keratinocyte Overexpression from PMID: 30772301	SOX2
SHPK	SOX2 regulated genes in Keratinocyte Overexpression from PMID: 30772301	SOX2
RRP12	SOX2 regulated genes in Keratinocyte Overexpression from PMID: 30772301	SOX2
SNRPD3	SOX2 regulated genes in Keratinocyte Overexpression from PMID: 30772301	SOX2
LIME1	SOX2 regulated genes in Keratinocyte Overexpression from PMID: 30772301	SOX2
WIPI2	SOX2 regulated genes in Keratinocyte Overexpression from PMID: 30772301	SOX2
EFS	SOX2 regulated genes in Keratinocyte Overexpression from PMID: 30772301	SOX2
DHRS1	SOX2 regulated genes in Keratinocyte Overexpression from PMID: 30772301	SOX2
EFHC1	SOX2 regulated genes in Keratinocyte Overexpression from PMID: 30772301	SOX2
TAB1	SOX2 regulated genes in Keratinocyte Overexpression from PMID: 30772301	SOX2
VAPA	SOX2 regulated genes in Keratinocyte Overexpression from PMID: 30772301	SOX2
HSBP1L1	SOX2 regulated genes in Keratinocyte Overexpression from PMID: 30772301	SOX2
TMC5	SOX2 regulated genes in Keratinocyte Overexpression from PMID: 30772301	SOX2
CD2AP	SOX2 regulated genes in Keratinocyte Overexpression from PMID: 30772301	SOX2
ROMO1	SOX2 regulated genes in Keratinocyte Overexpression from PMID: 30772301	SOX2
SLC15A1	SOX2 regulated genes in Keratinocyte Overexpression from PMID: 30772301	SOX2
XKR6	SOX2 regulated genes in Keratinocyte Overexpression from PMID: 30772301	SOX2
PAPPA	SOX2 regulated genes in Keratinocyte Overexpression from PMID: 30772301	SOX2
KIF27	SOX2 regulated genes in Keratinocyte Overexpression from PMID: 30772301	SOX2
YAP1	SOX2 regulated genes in Keratinocyte Overexpression from PMID: 30772301	SOX2
R3HDM4	SOX2 regulated genes in Keratinocyte Overexpression from PMID: 30772301	SOX2
ZFAND2A	SOX2 regulated genes in Keratinocyte Overexpression from PMID: 30772301	SOX2
SERGEF	SOX2 regulated genes in Keratinocyte Overexpression from PMID: 30772301	SOX2
WDHD1	SOX2 regulated genes in Keratinocyte Overexpression from PMID: 30772301	SOX2
PALD1	SOX2 regulated genes in Keratinocyte Overexpression from PMID: 30772301	SOX2
RNF138	SOX2 regulated genes in Keratinocyte Overexpression from PMID: 30772301	SOX2
FRYL	SOX2 regulated genes in Keratinocyte Overexpression from PMID: 30772301	SOX2
IPO8	SOX2 regulated genes in Keratinocyte Overexpression from PMID: 30772301	SOX2
SENP6	SOX2 regulated genes in Keratinocyte Overexpression from PMID: 30772301	SOX2
UTP14A	SOX2 regulated genes in Keratinocyte Overexpression from PMID: 30772301	SOX2
PRPS1	SOX2 regulated genes in Keratinocyte Overexpression from PMID: 30772301	SOX2
RPH3AL	SOX2 regulated genes in Keratinocyte Overexpression from PMID: 30772301	SOX2
CCAR1	SOX2 regulated genes in Keratinocyte Overexpression from PMID: 30772301	SOX2
INTS13	SOX2 regulated genes in Keratinocyte Overexpression from PMID: 30772301	SOX2
MTERF1	SOX2 regulated genes in Keratinocyte Overexpression from PMID: 30772301	SOX2
HDDC2	SOX2 regulated genes in Keratinocyte Overexpression from PMID: 30772301	SOX2
TCP11	SOX2 regulated genes in Keratinocyte Overexpression from PMID: 30772301	SOX2
ADNP	SOX2 regulated genes in Keratinocyte Overexpression from PMID: 30772301	SOX2
SLC39A2	SOX2 regulated genes in Keratinocyte Overexpression from PMID: 30772301	SOX2

IPO4	SOX2 regulated genes in Keratinocyte Overexpression from PMID: 30772301	SOX2
FAM204A	SOX2 regulated genes in Keratinocyte Overexpression from PMID: 30772301	SOX2
SDAD1	SOX2 regulated genes in Keratinocyte Overexpression from PMID: 30772301	SOX2
STK24	SOX2 regulated genes in Keratinocyte Overexpression from PMID: 30772301	SOX2
SLC34A2	SOX2 regulated genes in Keratinocyte Overexpression from PMID: 30772301	SOX2
RAP2C	SOX2 regulated genes in Keratinocyte Overexpression from PMID: 30772301	SOX2
C7orf50	SOX2 regulated genes in Keratinocyte Overexpression from PMID: 30772301	SOX2
BARX1	SOX2 regulated genes in Keratinocyte Overexpression from PMID: 30772301	SOX2
ZDHHC16	SOX2 regulated genes in Keratinocyte Overexpression from PMID: 30772301	SOX2
PHF2	SOX2 regulated genes in Keratinocyte Overexpression from PMID: 30772301	SOX2
ARFGAP2	SOX2 regulated genes in Keratinocyte Overexpression from PMID: 30772301	SOX2
RPS27L	SOX2 regulated genes in Keratinocyte Overexpression from PMID: 30772301	SOX2
PSMB7	SOX2 regulated genes in Keratinocyte Overexpression from PMID: 30772301	SOX2
FAU	SOX2 regulated genes in Keratinocyte Overexpression from PMID: 30772301	SOX2
PRDM8	SOX2 regulated genes in Keratinocyte Overexpression from PMID: 30772301	SOX2
ANKRD50	SOX2 regulated genes in Keratinocyte Overexpression from PMID: 30772301	SOX2
ID4	SOX2 regulated genes in Keratinocyte Overexpression from PMID: 30772301	SOX2
SERPINB5	SOX2 regulated genes in Keratinocyte Overexpression from PMID: 30772301	SOX2
PARM1	SOX2 regulated genes in Keratinocyte Overexpression from PMID: 30772301	SOX2
RIBC1	SOX2 regulated genes in Keratinocyte Overexpression from PMID: 30772301	SOX2
MRPS31	SOX2 regulated genes in Keratinocyte Overexpression from PMID: 30772301	SOX2
IGF2BP3	SOX2 regulated genes in Keratinocyte Overexpression from PMID: 30772301	SOX2
U2AF1L5	SOX2 regulated genes in Keratinocyte Overexpression from PMID: 30772301	SOX2
TFAP2B	SOX2 regulated genes in Keratinocyte Overexpression from PMID: 30772301	SOX2
E4F1	SOX2 regulated genes in Keratinocyte Overexpression from PMID: 30772301	SOX2
OPHN1	SOX2 regulated genes in Keratinocyte Overexpression from PMID: 30772301	SOX2
SOX12	SOX2 regulated genes in Keratinocyte Overexpression from PMID: 30772301	SOX2
CEP78	SOX2 regulated genes in Keratinocyte Overexpression from PMID: 30772301	SOX2
HOMEZ	SOX2 regulated genes in Keratinocyte Overexpression from PMID: 30772301	SOX2
TRIL	SOX2 regulated genes in Keratinocyte Overexpression from PMID: 30772301	SOX2
PEBP4	SOX2 regulated genes in Keratinocyte Overexpression from PMID: 30772301	SOX2
ARIH1	SOX2 regulated genes in Keratinocyte Overexpression from PMID: 30772301	SOX2
NKAP	SOX2 regulated genes in Keratinocyte Overexpression from PMID: 30772301	SOX2
ASCC3	SOX2 regulated genes in Keratinocyte Overexpression from PMID: 30772301	SOX2
GPKOW	SOX2 regulated genes in Keratinocyte Overexpression from PMID: 30772301	SOX2
BORCS5	SOX2 regulated genes in Keratinocyte Overexpression from PMID: 30772301	SOX2
SEC14L2	SOX2 regulated genes in Keratinocyte Overexpression from PMID: 30772301	SOX2
CCDC169	SOX2 regulated genes in Keratinocyte Overexpression from PMID: 30772301	SOX2
RWDD4	SOX2 regulated genes in Keratinocyte Overexpression from PMID: 30772301	SOX2
AFG3L2	SOX2 regulated genes in Keratinocyte Overexpression from PMID: 30772301	SOX2
UXT	SOX2 regulated genes in Keratinocyte Overexpression from PMID: 30772301	SOX2
ZNHIT2	SOX2 regulated genes in Keratinocyte Overexpression from PMID: 30772301	SOX2
HSPA4L	SOX2 regulated genes in Keratinocyte Overexpression from PMID: 30772301	SOX2
GON7	SOX2 regulated genes in Keratinocyte Overexpression from PMID: 30772301	SOX2
PCNA	SOX2 regulated genes in Keratinocyte Overexpression from PMID: 30772301	SOX2
ENDOU	SOX2 regulated genes in Keratinocyte Overexpression from PMID: 30772301	SOX2
AGR2	SOX2 regulated genes in Keratinocyte Overexpression from PMID: 30772301	SOX2
EIF2AK4	SOX2 regulated genes in Keratinocyte Overexpression from PMID: 30772301	SOX2
RTCB	SOX2 regulated genes in Keratinocyte Overexpression from PMID: 30772301	SOX2
RPP40	SOX2 regulated genes in Keratinocyte Overexpression from PMID: 30772301	SOX2
KIAA1217	SOX2 regulated genes in Keratinocyte Overexpression from PMID: 30772301	SOX2
CDCA7L	SOX2 regulated genes in Keratinocyte Overexpression from PMID: 30772301	SOX2
SCD	SOX2 regulated genes in Keratinocyte Overexpression from PMID: 30772301	SOX2
CDC42EP2	SOX2 regulated genes in Keratinocyte Overexpression from PMID: 30772301	SOX2
FOXO1	SOX2 regulated genes in Keratinocyte Overexpression from PMID: 30772301	SOX2
ONECUT3	SOX2 regulated genes in Keratinocyte Overexpression from PMID: 30772301	SOX2
TCFL5	SOX2 regulated genes in Keratinocyte Overexpression from PMID: 30772301	SOX2
RPL35	SOX2 regulated genes in Keratinocyte Overexpression from PMID: 30772301	SOX2
HACE1	SOX2 regulated genes in Keratinocyte Overexpression from PMID: 30772301	SOX2

QSOX2	SOX2 regulated genes in Keratinocyte Overexpression from PMID: 30772301	SOX2
HNRNPD	SOX2 regulated genes in Keratinocyte Overexpression from PMID: 30772301	SOX2
PLXNA3	SOX2 regulated genes in Keratinocyte Overexpression from PMID: 30772301	SOX2
UQCR10	SOX2 regulated genes in Keratinocyte Overexpression from PMID: 30772301	SOX2
AASDHPPT	SOX2 regulated genes in Keratinocyte Overexpression from PMID: 30772301	SOX2
TXNL4A	SOX2 regulated genes in Keratinocyte Overexpression from PMID: 30772301	SOX2
CEP295	SOX2 regulated genes in Keratinocyte Overexpression from PMID: 30772301	SOX2
ZNF280C	SOX2 regulated genes in Keratinocyte Overexpression from PMID: 30772301	SOX2
TAX1BP3	SOX2 regulated genes in Keratinocyte Overexpression from PMID: 30772301	SOX2
ELP4	SOX2 regulated genes in Keratinocyte Overexpression from PMID: 30772301	SOX2
RPUSD2	SOX2 regulated genes in Keratinocyte Overexpression from PMID: 30772301	SOX2
EIF3A	SOX2 regulated genes in Keratinocyte Overexpression from PMID: 30772301	SOX2
TSPAN14	SOX2 regulated genes in Keratinocyte Overexpression from PMID: 30772301	SOX2
HMGXB4	SOX2 regulated genes in Keratinocyte Overexpression from PMID: 30772301	SOX2
YTHDF1	SOX2 regulated genes in Keratinocyte Overexpression from PMID: 30772301	SOX2
SPEF1	SOX2 regulated genes in Keratinocyte Overexpression from PMID: 30772301	SOX2
JARID2	SOX2 regulated genes in Keratinocyte Overexpression from PMID: 30772301	SOX2
TXN	SOX2 regulated genes in Keratinocyte Overexpression from PMID: 30772301	SOX2
OAT	SOX2 regulated genes in Keratinocyte Overexpression from PMID: 30772301	SOX2
PRMT9	SOX2 regulated genes in Keratinocyte Overexpression from PMID: 30772301	SOX2
ATP12A	SOX2 regulated genes in Keratinocyte Overexpression from PMID: 30772301	SOX2
OSER1	SOX2 regulated genes in Keratinocyte Overexpression from PMID: 30772301	SOX2
ZNF335	SOX2 regulated genes in Keratinocyte Overexpression from PMID: 30772301	SOX2
ZSWIM3	SOX2 regulated genes in Keratinocyte Overexpression from PMID: 30772301	SOX2
SFXN2	SOX2 regulated genes in Keratinocyte Overexpression from PMID: 30772301	SOX2
ZFAND5	SOX2 regulated genes in Keratinocyte Overexpression from PMID: 30772301	SOX2
ETNPPL	SOX2 regulated genes in Keratinocyte Overexpression from PMID: 30772301	SOX2
RGP1	SOX2 regulated genes in Keratinocyte Overexpression from PMID: 30772301	SOX2
TRUB2	SOX2 regulated genes in Keratinocyte Overexpression from PMID: 30772301	SOX2
RCAN1	SOX2 regulated genes in Keratinocyte Overexpression from PMID: 30772301	SOX2
C11orf58	SOX2 regulated genes in Keratinocyte Overexpression from PMID: 30772301	SOX2
MAK16	SOX2 regulated genes in Keratinocyte Overexpression from PMID: 30772301	SOX2
TNFRSF13C	SOX2 regulated genes in Keratinocyte Overexpression from PMID: 30772301	SOX2
ZNF330	SOX2 regulated genes in Keratinocyte Overexpression from PMID: 30772301	SOX2
FPGS	SOX2 regulated genes in Keratinocyte Overexpression from PMID: 30772301	SOX2
DHX33	SOX2 regulated genes in Keratinocyte Overexpression from PMID: 30772301	SOX2
SAC3D1	SOX2 regulated genes in Keratinocyte Overexpression from PMID: 30772301	SOX2
RAB40C	SOX2 regulated genes in Keratinocyte Overexpression from PMID: 30772301	SOX2
RFLNB	SOX2 regulated genes in Keratinocyte Overexpression from PMID: 30772301	SOX2
MACROD2	SOX2 regulated genes in Keratinocyte Overexpression from PMID: 30772301	SOX2
VNN1	SOX2 regulated genes in Keratinocyte Overexpression from PMID: 30772301	SOX2
HIPK3	SOX2 regulated genes in Keratinocyte Overexpression from PMID: 30772301	SOX2
ARHGEF39	SOX2 regulated genes in Keratinocyte Overexpression from PMID: 30772301	SOX2
PGAP3	SOX2 regulated genes in Keratinocyte Overexpression from PMID: 30772301	SOX2
CCKBR	SOX2 regulated genes in Keratinocyte Overexpression from PMID: 30772301	SOX2
BMS1	SOX2 regulated genes in Keratinocyte Overexpression from PMID: 30772301	SOX2
MED31	SOX2 regulated genes in Keratinocyte Overexpression from PMID: 30772301	SOX2
DCDC2	SOX2 regulated genes in Keratinocyte Overexpression from PMID: 30772301	SOX2
XRCC6	SOX2 regulated genes in Keratinocyte Overexpression from PMID: 30772301	SOX2
EGFL6	SOX2 regulated genes in Keratinocyte Overexpression from PMID: 30772301	SOX2
KCTD9	SOX2 regulated genes in Keratinocyte Overexpression from PMID: 30772301	SOX2
SNX12	SOX2 regulated genes in Keratinocyte Overexpression from PMID: 30772301	SOX2
NT5C3B	SOX2 regulated genes in Keratinocyte Overexpression from PMID: 30772301	SOX2
BCCIP	SOX2 regulated genes in Keratinocyte Overexpression from PMID: 30772301	SOX2
STUB1	SOX2 regulated genes in Keratinocyte Overexpression from PMID: 30772301	SOX2
INIP	SOX2 regulated genes in Keratinocyte Overexpression from PMID: 30772301	SOX2
DDX3X	SOX2 regulated genes in Keratinocyte Overexpression from PMID: 30772301	SOX2
ANKRD10	SOX2 regulated genes in Keratinocyte Overexpression from PMID: 30772301	SOX2
EHMT1	SOX2 regulated genes in Keratinocyte Overexpression from PMID: 30772301	SOX2

DICER1	SOX2 regulated genes in Keratinocyte Overexpression from PMID: 30772301	SOX2
MBOAT1	SOX2 regulated genes in Keratinocyte Overexpression from PMID: 30772301	SOX2
NR6A1	SOX2 regulated genes in Keratinocyte Overexpression from PMID: 30772301	SOX2
MDN1	SOX2 regulated genes in Keratinocyte Overexpression from PMID: 30772301	SOX2
CREB5	SOX2 regulated genes in Keratinocyte Overexpression from PMID: 30772301	SOX2
S1PR3	SOX2 regulated genes in Keratinocyte Overexpression from PMID: 30772301	SOX2
ASCC1	SOX2 regulated genes in Keratinocyte Overexpression from PMID: 30772301	SOX2
SOGA3	SOX2 regulated genes in Keratinocyte Overexpression from PMID: 30772301	SOX2
ARHGAP42	SOX2 regulated genes in Keratinocyte Overexpression from PMID: 30772301	SOX2
KIF6	SOX2 regulated genes in Keratinocyte Overexpression from PMID: 30772301	SOX2
GNB1L	SOX2 regulated genes in Keratinocyte Overexpression from PMID: 30772301	SOX2
ALDH8A1	SOX2 regulated genes in Keratinocyte Overexpression from PMID: 30772301	SOX2
CDKAL1	SOX2 regulated genes in Keratinocyte Overexpression from PMID: 30772301	SOX2
SLC35F2	SOX2 regulated genes in Keratinocyte Overexpression from PMID: 30772301	SOX2
MID2	SOX2 regulated genes in Keratinocyte Overexpression from PMID: 30772301	SOX2
EBF4	SOX2 regulated genes in Keratinocyte Overexpression from PMID: 30772301	SOX2
UFD1	SOX2 regulated genes in Keratinocyte Overexpression from PMID: 30772301	SOX2
NUDT3	SOX2 regulated genes in Keratinocyte Overexpression from PMID: 30772301	SOX2
TRMT2A	SOX2 regulated genes in Keratinocyte Overexpression from PMID: 30772301	SOX2
ZC3H14	SOX2 regulated genes in Keratinocyte Overexpression from PMID: 30772301	SOX2
ARFGEF2	SOX2 regulated genes in Keratinocyte Overexpression from PMID: 30772301	SOX2
COX4I2	SOX2 regulated genes in Keratinocyte Overexpression from PMID: 30772301	SOX2
SALL3	SOX2 regulated genes in Keratinocyte Overexpression from PMID: 30772301	SOX2
EFNB2	SOX2 regulated genes in Keratinocyte Overexpression from PMID: 30772301	SOX2
PMS2	SOX2 regulated genes in Keratinocyte Overexpression from PMID: 30772301	SOX2
SCLT1	SOX2 regulated genes in Keratinocyte Overexpression from PMID: 30772301	SOX2
TTF1	SOX2 regulated genes in Keratinocyte Overexpression from PMID: 30772301	SOX2
ITPRIP	SOX2 regulated genes in Keratinocyte Overexpression from PMID: 30772301	SOX2
DSN1	SOX2 regulated genes in Keratinocyte Overexpression from PMID: 30772301	SOX2
CPNE8	SOX2 regulated genes in Keratinocyte Overexpression from PMID: 30772301	SOX2
RPAP3	SOX2 regulated genes in Keratinocyte Overexpression from PMID: 30772301	SOX2
CISD2	SOX2 regulated genes in Keratinocyte Overexpression from PMID: 30772301	SOX2
INTS1	SOX2 regulated genes in Keratinocyte Overexpression from PMID: 30772301	SOX2
METTL4	SOX2 regulated genes in Keratinocyte Overexpression from PMID: 30772301	SOX2
TMPRSS11F	SOX2 regulated genes in Keratinocyte Overexpression from PMID: 30772301	SOX2
BNIP3L	SOX2 regulated genes in Keratinocyte Overexpression from PMID: 30772301	SOX2
ADGRF1	SOX2 regulated genes in Keratinocyte Overexpression from PMID: 30772301	SOX2
SPRYD3	SOX2 regulated genes in Keratinocyte Overexpression from PMID: 30772301	SOX2
MRPL16	SOX2 regulated genes in Keratinocyte Overexpression from PMID: 30772301	SOX2
FANCC	SOX2 regulated genes in Keratinocyte Overexpression from PMID: 30772301	SOX2
PLP2	SOX2 regulated genes in Keratinocyte Overexpression from PMID: 30772301	SOX2
WDR24	SOX2 regulated genes in Keratinocyte Overexpression from PMID: 30772301	SOX2
TCF7L2	SOX2 regulated genes in Keratinocyte Overexpression from PMID: 30772301	SOX2
LRP10	SOX2 regulated genes in Keratinocyte Overexpression from PMID: 30772301	SOX2
CRELD2	SOX2 regulated genes in Keratinocyte Overexpression from PMID: 30772301	SOX2
ABRACL	SOX2 regulated genes in Keratinocyte Overexpression from PMID: 30772301	SOX2
NOL8	SOX2 regulated genes in Keratinocyte Overexpression from PMID: 30772301	SOX2
MTMR3	SOX2 regulated genes in Keratinocyte Overexpression from PMID: 30772301	SOX2
HHIP	SOX2 regulated genes in Keratinocyte Overexpression from PMID: 30772301	SOX2
HAP1	SOX2 regulated genes in Keratinocyte Overexpression from PMID: 30772301	SOX2
CHKA	SOX2 regulated genes in Keratinocyte Overexpression from PMID: 30772301	SOX2
CYP46A1	SOX2 regulated genes in Keratinocyte Overexpression from PMID: 30772301	SOX2
MBD2	SOX2 regulated genes in Keratinocyte Overexpression from PMID: 30772301	SOX2
TMEM98	SOX2 regulated genes in Keratinocyte Overexpression from PMID: 30772301	SOX2
FERMT1	SOX2 regulated genes in Keratinocyte Overexpression from PMID: 30772301	SOX2
SMCHD1	SOX2 regulated genes in Keratinocyte Overexpression from PMID: 30772301	SOX2
PLXNB2	SOX2 regulated genes in Keratinocyte Overexpression from PMID: 30772301	SOX2
MAD2L1	SOX2 regulated genes in Keratinocyte Overexpression from PMID: 30772301	SOX2
VTA1	SOX2 regulated genes in Keratinocyte Overexpression from PMID: 30772301	SOX2

CARNMT1	SOX2 regulated genes in Keratinocyte Overexpression from PMID: 30772301	SOX2
CELSR1	SOX2 regulated genes in Keratinocyte Overexpression from PMID: 30772301	SOX2
C5orf49	SOX2 regulated genes in Keratinocyte Overexpression from PMID: 30772301	SOX2
CDKL2	SOX2 regulated genes in Keratinocyte Overexpression from PMID: 30772301	SOX2
CNOT7	SOX2 regulated genes in Keratinocyte Overexpression from PMID: 30772301	SOX2
DSC3	SOX2 regulated genes in Keratinocyte Overexpression from PMID: 30772301	SOX2
BUD31	SOX2 regulated genes in Keratinocyte Overexpression from PMID: 30772301	SOX2
HDAC2	SOX2 regulated genes in Keratinocyte Overexpression from PMID: 30772301	SOX2
C20orf27	SOX2 regulated genes in Keratinocyte Overexpression from PMID: 30772301	SOX2
PITPNM3	SOX2 regulated genes in Keratinocyte Overexpression from PMID: 30772301	SOX2
SUZ12	SOX2 regulated genes in Keratinocyte Overexpression from PMID: 30772301	SOX2
TUBA8	SOX2 regulated genes in Keratinocyte Overexpression from PMID: 30772301	SOX2
ZCCHC3	SOX2 regulated genes in Keratinocyte Overexpression from PMID: 30772301	SOX2
DMRT1	SOX2 regulated genes in Keratinocyte Overexpression from PMID: 30772301	SOX2
RPS19BP1	SOX2 regulated genes in Keratinocyte Overexpression from PMID: 30772301	SOX2
OSBPL2	SOX2 regulated genes in Keratinocyte Overexpression from PMID: 30772301	SOX2
FABP7	SOX2 regulated genes in Keratinocyte Overexpression from PMID: 30772301	SOX2
PGP	SOX2 regulated genes in Keratinocyte Overexpression from PMID: 30772301	SOX2
KRT6B	SOX2 regulated genes in Keratinocyte Overexpression from PMID: 30772301	SOX2
KRT6C	SOX2 regulated genes in Keratinocyte Overexpression from PMID: 30772301	SOX2
CPXM2	SOX2 regulated genes in Keratinocyte Overexpression from PMID: 30772301	SOX2
SCML4	SOX2 regulated genes in Keratinocyte Overexpression from PMID: 30772301	SOX2
PARD6G	SOX2 regulated genes in Keratinocyte Overexpression from PMID: 30772301	SOX2
ZNF341	SOX2 regulated genes in Keratinocyte Overexpression from PMID: 30772301	SOX2
AFAP1L2	SOX2 regulated genes in Keratinocyte Overexpression from PMID: 30772301	SOX2
PIGW	SOX2 regulated genes in Keratinocyte Overexpression from PMID: 30772301	SOX2
ASNS	SOX2 regulated genes in Keratinocyte Overexpression from PMID: 30772301	SOX2
DNAAF5	SOX2 regulated genes in Keratinocyte Overexpression from PMID: 30772301	SOX2
GRB7	SOX2 regulated genes in Keratinocyte Overexpression from PMID: 30772301	SOX2
FKBP1A	SOX2 regulated genes in Keratinocyte Overexpression from PMID: 30772301	SOX2
FKBP1C	SOX2 regulated genes in Keratinocyte Overexpression from PMID: 30772301	SOX2
PRRG4	SOX2 regulated genes in Keratinocyte Overexpression from PMID: 30772301	SOX2
GBA2	SOX2 regulated genes in Keratinocyte Overexpression from PMID: 30772301	SOX2
CUL4B	SOX2 regulated genes in Keratinocyte Overexpression from PMID: 30772301	SOX2
TFIP11	SOX2 regulated genes in Keratinocyte Overexpression from PMID: 30772301	SOX2
PLEKHS1	SOX2 regulated genes in Keratinocyte Overexpression from PMID: 30772301	SOX2
GOPC	SOX2 regulated genes in Keratinocyte Overexpression from PMID: 30772301	SOX2
KDM2A	SOX2 regulated genes in Keratinocyte Overexpression from PMID: 30772301	SOX2
SPIN1	SOX2 regulated genes in Keratinocyte Overexpression from PMID: 30772301	SOX2
PON2	SOX2 regulated genes in Keratinocyte Overexpression from PMID: 30772301	SOX2
MRPS17	SOX2 regulated genes in Keratinocyte Overexpression from PMID: 30772301	SOX2
KIAA0753	SOX2 regulated genes in Keratinocyte Overexpression from PMID: 30772301	SOX2
TSSC4	SOX2 regulated genes in Keratinocyte Overexpression from PMID: 30772301	SOX2
USP22	SOX2 regulated genes in Keratinocyte Overexpression from PMID: 30772301	SOX2
UBL4A	SOX2 regulated genes in Keratinocyte Overexpression from PMID: 30772301	SOX2
CSPG4	SOX2 regulated genes in Keratinocyte Overexpression from PMID: 30772301	SOX2
FANCI	SOX2 regulated genes in Keratinocyte Overexpression from PMID: 30772301	SOX2
PERP	SOX2 regulated genes in Keratinocyte Overexpression from PMID: 30772301	SOX2
TP53RK	SOX2 regulated genes in Keratinocyte Overexpression from PMID: 30772301	SOX2
COX8A	SOX2 regulated genes in Keratinocyte Overexpression from PMID: 30772301	SOX2
TIMMDC1	SOX2 regulated genes in Keratinocyte Overexpression from PMID: 30772301	SOX2
BMP2K	SOX2 regulated genes in Keratinocyte Overexpression from PMID: 30772301	SOX2
ACTR5	SOX2 regulated genes in Keratinocyte Overexpression from PMID: 30772301	SOX2
WDR59	SOX2 regulated genes in Keratinocyte Overexpression from PMID: 30772301	SOX2
POLR2K	SOX2 regulated genes in Keratinocyte Overexpression from PMID: 30772301	SOX2
WRAP53	SOX2 regulated genes in Keratinocyte Overexpression from PMID: 30772301	SOX2
APOM	SOX2 regulated genes in Keratinocyte Overexpression from PMID: 30772301	SOX2
NELFB	SOX2 regulated genes in Keratinocyte Overexpression from PMID: 30772301	SOX2
KIF18A	SOX2 regulated genes in Keratinocyte Overexpression from PMID: 30772301	SOX2

ZNF274	SOX2 regulated genes in Keratinocyte Overexpression from PMID: 30772301	SOX2
PNN	SOX2 regulated genes in Keratinocyte Overexpression from PMID: 30772301	SOX2
GABRQ	SOX2 regulated genes in Keratinocyte Overexpression from PMID: 30772301	SOX2
UBE2E1	SOX2 regulated genes in Keratinocyte Overexpression from PMID: 30772301	SOX2
MIA-RAB4B	SOX2 regulated genes in Keratinocyte Overexpression from PMID: 30772301	SOX2
MIA	SOX2 regulated genes in Keratinocyte Overexpression from PMID: 30772301	SOX2
RNF214	SOX2 regulated genes in Keratinocyte Overexpression from PMID: 30772301	SOX2
SERPIND1	SOX2 regulated genes in Keratinocyte Overexpression from PMID: 30772301	SOX2
ZNF426	SOX2 regulated genes in Keratinocyte Overexpression from PMID: 30772301	SOX2
DXO	SOX2 regulated genes in Keratinocyte Overexpression from PMID: 30772301	SOX2
CD82	SOX2 regulated genes in Keratinocyte Overexpression from PMID: 30772301	SOX2
ITPKC	SOX2 regulated genes in Keratinocyte Overexpression from PMID: 30772301	SOX2
NXPE4	SOX2 regulated genes in Keratinocyte Overexpression from PMID: 30772301	SOX2
TRAPPC11	SOX2 regulated genes in Keratinocyte Overexpression from PMID: 30772301	SOX2
CST6	SOX2 regulated genes in Keratinocyte Overexpression from PMID: 30772301	SOX2
SYMPK	SOX2 regulated genes in Keratinocyte Overexpression from PMID: 30772301	SOX2
RWDD1	SOX2 regulated genes in Keratinocyte Overexpression from PMID: 30772301	SOX2
CHCHD6	SOX2 regulated genes in Keratinocyte Overexpression from PMID: 30772301	SOX2
YRDC	SOX2 regulated genes in Keratinocyte Overexpression from PMID: 30772301	SOX2
FBXO30	SOX2 regulated genes in Keratinocyte Overexpression from PMID: 30772301	SOX2
POLR3B	SOX2 regulated genes in Keratinocyte Overexpression from PMID: 30772301	SOX2
CLPX	SOX2 regulated genes in Keratinocyte Overexpression from PMID: 30772301	SOX2
TPRA1	SOX2 regulated genes in Keratinocyte Overexpression from PMID: 30772301	SOX2
GALNT14	SOX2 regulated genes in Keratinocyte Overexpression from PMID: 30772301	SOX2
MYO5C	SOX2 regulated genes in Keratinocyte Overexpression from PMID: 30772301	SOX2
ZNRF1	SOX2 regulated genes in Keratinocyte Overexpression from PMID: 30772301	SOX2
NUP214	SOX2 regulated genes in Keratinocyte Overexpression from PMID: 30772301	SOX2
WNK2	SOX2 regulated genes in Keratinocyte Overexpression from PMID: 30772301	SOX2
CBWD3	SOX2 regulated genes in Keratinocyte Overexpression from PMID: 30772301	SOX2
CBWD5	SOX2 regulated genes in Keratinocyte Overexpression from PMID: 30772301	SOX2
CBWD6	SOX2 regulated genes in Keratinocyte Overexpression from PMID: 30772301	SOX2
USP1	SOX2 regulated genes in Keratinocyte Overexpression from PMID: 30772301	SOX2
SLC27A5	SOX2 regulated genes in Keratinocyte Overexpression from PMID: 30772301	SOX2
RNF6	SOX2 regulated genes in Keratinocyte Overexpression from PMID: 30772301	SOX2
TMEM254	SOX2 regulated genes in Keratinocyte Overexpression from PMID: 30772301	SOX2
RPP30	SOX2 regulated genes in Keratinocyte Overexpression from PMID: 30772301	SOX2
RNASEH2C	SOX2 regulated genes in Keratinocyte Overexpression from PMID: 30772301	SOX2
COMMD2	SOX2 regulated genes in Keratinocyte Overexpression from PMID: 30772301	SOX2
AEN	SOX2 regulated genes in Keratinocyte Overexpression from PMID: 30772301	SOX2
NRG2	SOX2 regulated genes in Keratinocyte Overexpression from PMID: 30772301	SOX2
CENPU	SOX2 regulated genes in Keratinocyte Overexpression from PMID: 30772301	SOX2
KCNMB4	SOX2 regulated genes in Keratinocyte Overexpression from PMID: 30772301	SOX2
MAP3K9	SOX2 regulated genes in Keratinocyte Overexpression from PMID: 30772301	SOX2
EPC1	SOX2 regulated genes in Keratinocyte Overexpression from PMID: 30772301	SOX2
GRTP1	SOX2 regulated genes in Keratinocyte Overexpression from PMID: 30772301	SOX2
NETO2	SOX2 regulated genes in Keratinocyte Overexpression from PMID: 30772301	SOX2
SEMA4D	SOX2 regulated genes in Keratinocyte Overexpression from PMID: 30772301	SOX2
TMC1	SOX2 regulated genes in Keratinocyte Overexpression from PMID: 30772301	SOX2
SARNP	SOX2 regulated genes in Keratinocyte Overexpression from PMID: 30772301	SOX2
RHPN1	SOX2 regulated genes in Keratinocyte Overexpression from PMID: 30772301	SOX2
ALG11	SOX2 regulated genes in Keratinocyte Overexpression from PMID: 30772301	SOX2
CTHRC1	SOX2 regulated genes in Keratinocyte Overexpression from PMID: 30772301	SOX2
EPB41L4B	SOX2 regulated genes in Keratinocyte Overexpression from PMID: 30772301	SOX2
MAMDC4	SOX2 regulated genes in Keratinocyte Overexpression from PMID: 30772301	SOX2
TLK1	SOX2 regulated genes in Keratinocyte Overexpression from PMID: 30772301	SOX2
IER3	SOX2 regulated genes in Keratinocyte Overexpression from PMID: 30772301	SOX2
ENHO	SOX2 regulated genes in Keratinocyte Overexpression from PMID: 30772301	SOX2
TAB3	SOX2 regulated genes in Keratinocyte Overexpression from PMID: 30772301	SOX2
MED22	SOX2 regulated genes in Keratinocyte Overexpression from PMID: 30772301	SOX2

DBR1	SOX2 regulated genes in Keratinocyte Overexpression from PMID: 30772301	SOX2
MPV17L	SOX2 regulated genes in Keratinocyte Overexpression from PMID: 30772301	SOX2
IFT74	SOX2 regulated genes in Keratinocyte Overexpression from PMID: 30772301	SOX2
CYP1B1	SOX2 regulated genes in Keratinocyte Overexpression from PMID: 30772301	SOX2
TG	SOX2 regulated genes in Keratinocyte Overexpression from PMID: 30772301	SOX2
CDSN	SOX2 regulated genes in Keratinocyte Overexpression from PMID: 30772301	SOX2
PAIP2B	SOX2 regulated genes in Keratinocyte Overexpression from PMID: 30772301	SOX2
DTYMK	SOX2 regulated genes in Keratinocyte Overexpression from PMID: 30772301	SOX2
SPATA5L1	SOX2 regulated genes in Keratinocyte Overexpression from PMID: 30772301	SOX2
COPG2	SOX2 regulated genes in Keratinocyte Overexpression from PMID: 30772301	SOX2
ZBTB40	SOX2 regulated genes in Keratinocyte Overexpression from PMID: 30772301	SOX2
CTU2	SOX2 regulated genes in Keratinocyte Overexpression from PMID: 30772301	SOX2
NEIL1	SOX2 regulated genes in Keratinocyte Overexpression from PMID: 30772301	SOX2
MYCBP	SOX2 regulated genes in Keratinocyte Overexpression from PMID: 30772301	SOX2
BDKRB2	SOX2 regulated genes in Keratinocyte Overexpression from PMID: 30772301	SOX2
BHLHB9	SOX2 regulated genes in Keratinocyte Overexpression from PMID: 30772301	SOX2
CHCHD2	SOX2 regulated genes in Keratinocyte Overexpression from PMID: 30772301	SOX2
POLR2L	SOX2 regulated genes in Keratinocyte Overexpression from PMID: 30772301	SOX2
SNRNP200	SOX2 regulated genes in Keratinocyte Overexpression from PMID: 30772301	SOX2
CALCOCO1	SOX2 regulated genes in Keratinocyte Overexpression from PMID: 30772301	SOX2
NUP107	SOX2 regulated genes in Keratinocyte Overexpression from PMID: 30772301	SOX2
NDE1	SOX2 regulated genes in Keratinocyte Overexpression from PMID: 30772301	SOX2
SKIV2L	SOX2 regulated genes in Keratinocyte Overexpression from PMID: 30772301	SOX2
PRSS36	SOX2 regulated genes in Keratinocyte Overexpression from PMID: 30772301	SOX2
RNF19A	SOX2 regulated genes in Keratinocyte Overexpression from PMID: 30772301	SOX2
SOD2	SOX2 regulated genes in Keratinocyte Overexpression from PMID: 30772301	SOX2
FXN	SOX2 regulated genes in Keratinocyte Overexpression from PMID: 30772301	SOX2
NOXA1	SOX2 regulated genes in Keratinocyte Overexpression from PMID: 30772301	SOX2
EXOSC6	SOX2 regulated genes in Keratinocyte Overexpression from PMID: 30772301	SOX2
TRPV3	SOX2 regulated genes in Keratinocyte Overexpression from PMID: 30772301	SOX2
TMEM115	SOX2 regulated genes in Keratinocyte Overexpression from PMID: 30772301	SOX2
FBXO43	SOX2 regulated genes in Keratinocyte Overexpression from PMID: 30772301	SOX2
PDIA2	SOX2 regulated genes in Keratinocyte Overexpression from PMID: 30772301	SOX2
HAUS7	SOX2 regulated genes in Keratinocyte Overexpression from PMID: 30772301	SOX2
IL17D	SOX2 regulated genes in Keratinocyte Overexpression from PMID: 30772301	SOX2
RAD52	SOX2 regulated genes in Keratinocyte Overexpression from PMID: 30772301	SOX2
INHBB	SOX2 regulated genes in Keratinocyte Overexpression from PMID: 30772301	SOX2
FAM184B	SOX2 regulated genes in Keratinocyte Overexpression from PMID: 30772301	SOX2
TAPT1	SOX2 regulated genes in Keratinocyte Overexpression from PMID: 30772301	SOX2
UBXN2A	SOX2 regulated genes in Keratinocyte Overexpression from PMID: 30772301	SOX2
SEMA4G	SOX2 regulated genes in Keratinocyte Overexpression from PMID: 30772301	SOX2
CPT1A	SOX2 regulated genes in Keratinocyte Overexpression from PMID: 30772301	SOX2
BRMS1	SOX2 regulated genes in Keratinocyte Overexpression from PMID: 30772301	SOX2
ARHGEF26	SOX2 regulated genes in Keratinocyte Overexpression from PMID: 30772301	SOX2
PTCD1	SOX2 regulated genes in Keratinocyte Overexpression from PMID: 30772301	SOX2
CDKN1A	SOX2 regulated genes in Keratinocyte Overexpression from PMID: 30772301	SOX2
CABLES2	SOX2 regulated genes in Keratinocyte Overexpression from PMID: 30772301	SOX2
RBM22	SOX2 regulated genes in Keratinocyte Overexpression from PMID: 30772301	SOX2
MTHFD2L	SOX2 regulated genes in Keratinocyte Overexpression from PMID: 30772301	SOX2
DTX3	SOX2 regulated genes in Keratinocyte Overexpression from PMID: 30772301	SOX2
SMARCAD1	SOX2 regulated genes in Keratinocyte Overexpression from PMID: 30772301	SOX2
DRD4	SOX2 regulated genes in Keratinocyte Overexpression from PMID: 30772301	SOX2
KLK1	SOX2 regulated genes in Keratinocyte Overexpression from PMID: 30772301	SOX2
LAMB1	SOX2 regulated genes in Keratinocyte Overexpression from PMID: 30772301	SOX2
RNF185	SOX2 regulated genes in Keratinocyte Overexpression from PMID: 30772301	SOX2
POLR1D	SOX2 regulated genes in Keratinocyte Overexpression from PMID: 30772301	SOX2
GLE1	SOX2 regulated genes in Keratinocyte Overexpression from PMID: 30772301	SOX2
CBY1	SOX2 regulated genes in Keratinocyte Overexpression from PMID: 30772301	SOX2
SPOPL	SOX2 regulated genes in Keratinocyte Overexpression from PMID: 30772301	SOX2

MASTL	SOX2 regulated genes in Keratinocyte Overexpression from PMID: 30772301	SOX2
POLR2B	SOX2 regulated genes in Keratinocyte Overexpression from PMID: 30772301	SOX2
RBM27	SOX2 regulated genes in Keratinocyte Overexpression from PMID: 30772301	SOX2
KATNA1	SOX2 regulated genes in Keratinocyte Overexpression from PMID: 30772301	SOX2
PALB2	SOX2 regulated genes in Keratinocyte Overexpression from PMID: 30772301	SOX2
MCRS1	SOX2 regulated genes in Keratinocyte Overexpression from PMID: 30772301	SOX2
GPR3	SOX2 regulated genes in Keratinocyte Overexpression from PMID: 30772301	SOX2
MANF	SOX2 regulated genes in Keratinocyte Overexpression from PMID: 30772301	SOX2
QRSL1	SOX2 regulated genes in Keratinocyte Overexpression from PMID: 30772301	SOX2
SH3RF2	SOX2 regulated genes in Keratinocyte Overexpression from PMID: 30772301	SOX2
RCOR2	SOX2 regulated genes in Keratinocyte Overexpression from PMID: 30772301	SOX2
HIPK4	SOX2 regulated genes in Keratinocyte Overexpression from PMID: 30772301	SOX2
NUP205	SOX2 regulated genes in Keratinocyte Overexpression from PMID: 30772301	SOX2
NT5DC2	SOX2 regulated genes in Keratinocyte Overexpression from PMID: 30772301	SOX2
ARPP19	SOX2 regulated genes in Keratinocyte Overexpression from PMID: 30772301	SOX2
RNF7	SOX2 regulated genes in Keratinocyte Overexpression from PMID: 30772301	SOX2
MAGOH	SOX2 regulated genes in Keratinocyte Overexpression from PMID: 30772301	SOX2
ECHDC2	SOX2 regulated genes in Keratinocyte Overexpression from PMID: 30772301	SOX2
MET	SOX2 regulated genes in Keratinocyte Overexpression from PMID: 30772301	SOX2
MRPL52	SOX2 regulated genes in Keratinocyte Overexpression from PMID: 30772301	SOX2
MRPL48	SOX2 regulated genes in Keratinocyte Overexpression from PMID: 30772301	SOX2
YPEL3	SOX2 regulated genes in Keratinocyte Overexpression from PMID: 30772301	SOX2
PPIF	SOX2 regulated genes in Keratinocyte Overexpression from PMID: 30772301	SOX2
SRBD1	SOX2 regulated genes in Keratinocyte Overexpression from PMID: 30772301	SOX2
RANBP3	SOX2 regulated genes in Keratinocyte Overexpression from PMID: 30772301	SOX2
ZNF507	SOX2 regulated genes in Keratinocyte Overexpression from PMID: 30772301	SOX2
NHLRC2	SOX2 regulated genes in Keratinocyte Overexpression from PMID: 30772301	SOX2
OXSR1	SOX2 regulated genes in Keratinocyte Overexpression from PMID: 30772301	SOX2
MRRF	SOX2 regulated genes in Keratinocyte Overexpression from PMID: 30772301	SOX2
ESM1	SOX2 regulated genes in Keratinocyte Overexpression from PMID: 30772301	SOX2
PIGO	SOX2 regulated genes in Keratinocyte Overexpression from PMID: 30772301	SOX2
UTP3	SOX2 regulated genes in Keratinocyte Overexpression from PMID: 30772301	SOX2
CBX4	SOX2 regulated genes in Keratinocyte Overexpression from PMID: 30772301	SOX2
C6orf226	SOX2 regulated genes in Keratinocyte Overexpression from PMID: 30772301	SOX2
PKMYT1	SOX2 regulated genes in Keratinocyte Overexpression from PMID: 30772301	SOX2
ERICH5	SOX2 regulated genes in Keratinocyte Overexpression from PMID: 30772301	SOX2
FBXW7	SOX2 regulated genes in Keratinocyte Overexpression from PMID: 30772301	SOX2
CENPI	SOX2 regulated genes in Keratinocyte Overexpression from PMID: 30772301	SOX2
CDKL3	SOX2 regulated genes in Keratinocyte Overexpression from PMID: 30772301	SOX2
CCT4	SOX2 regulated genes in Keratinocyte Overexpression from PMID: 30772301	SOX2
TRAPPC13	SOX2 regulated genes in Keratinocyte Overexpression from PMID: 30772301	SOX2
INSIG1	SOX2 regulated genes in Keratinocyte Overexpression from PMID: 30772301	SOX2
PRC1	SOX2 regulated genes in Keratinocyte Overexpression from PMID: 30772301	SOX2
HIPK2	SOX2 regulated genes in Keratinocyte Overexpression from PMID: 30772301	SOX2
FSBP	SOX2 regulated genes in Keratinocyte Overexpression from PMID: 30772301	SOX2
PSMB1	SOX2 regulated genes in Keratinocyte Overexpression from PMID: 30772301	SOX2
GCAT	SOX2 regulated genes in Keratinocyte Overexpression from PMID: 30772301	SOX2
ZBTB44	SOX2 regulated genes in Keratinocyte Overexpression from PMID: 30772301	SOX2
ORC6	SOX2 regulated genes in Keratinocyte Overexpression from PMID: 30772301	SOX2
DERL1	SOX2 regulated genes in Keratinocyte Overexpression from PMID: 30772301	SOX2
NUTF2	SOX2 regulated genes in Keratinocyte Overexpression from PMID: 30772301	SOX2
UBE4B	SOX2 regulated genes in Keratinocyte Overexpression from PMID: 30772301	SOX2
YLPM1	SOX2 regulated genes in Keratinocyte Overexpression from PMID: 30772301	SOX2
GALT	SOX2 regulated genes in Keratinocyte Overexpression from PMID: 30772301	SOX2
NR2C2AP	SOX2 regulated genes in Keratinocyte Overexpression from PMID: 30772301	SOX2
RFWD3	SOX2 regulated genes in Keratinocyte Overexpression from PMID: 30772301	SOX2
GINS2	SOX2 regulated genes in Keratinocyte Overexpression from PMID: 30772301	SOX2
ETAA1	SOX2 regulated genes in Keratinocyte Overexpression from PMID: 30772301	SOX2
PDCD2L	SOX2 regulated genes in Keratinocyte Overexpression from PMID: 30772301	SOX2

NCBP2L	SOX2 regulated genes in Keratinocyte Overexpression from PMID: 30772301	SOX2
CHP1	SOX2 regulated genes in Keratinocyte Overexpression from PMID: 30772301	SOX2
BRPF3	SOX2 regulated genes in Keratinocyte Overexpression from PMID: 30772301	SOX2
KRTAP9-4	SOX2 regulated genes in Keratinocyte Overexpression from PMID: 30772301	SOX2
XPO4	SOX2 regulated genes in Keratinocyte Overexpression from PMID: 30772301	SOX2
DDA1	SOX2 regulated genes in Keratinocyte Overexpression from PMID: 30772301	SOX2
DOCK3	SOX2 regulated genes in Keratinocyte Overexpression from PMID: 30772301	SOX2
ENO4	SOX2 regulated genes in Keratinocyte Overexpression from PMID: 30772301	SOX2
TUSC2	SOX2 regulated genes in Keratinocyte Overexpression from PMID: 30772301	SOX2
SPEN	SOX2 regulated genes in Keratinocyte Overexpression from PMID: 30772301	SOX2
DHFR	SOX2 regulated genes in Keratinocyte Overexpression from PMID: 30772301	SOX2
MED6	SOX2 regulated genes in Keratinocyte Overexpression from PMID: 30772301	SOX2
KANK4	SOX2 regulated genes in Keratinocyte Overexpression from PMID: 30772301	SOX2
FUS	SOX2 regulated genes in Keratinocyte Overexpression from PMID: 30772301	SOX2
SGF29	SOX2 regulated genes in Keratinocyte Overexpression from PMID: 30772301	SOX2
EPB41L5	SOX2 regulated genes in Keratinocyte Overexpression from PMID: 30772301	SOX2
STAM2	SOX2 regulated genes in Keratinocyte Overexpression from PMID: 30772301	SOX2
FEM1C	SOX2 regulated genes in Keratinocyte Overexpression from PMID: 30772301	SOX2
CKAP2	SOX2 regulated genes in Keratinocyte Overexpression from PMID: 30772301	SOX2
SLC25A25	SOX2 regulated genes in Keratinocyte Overexpression from PMID: 30772301	SOX2
OXNAD1	SOX2 regulated genes in Keratinocyte Overexpression from PMID: 30772301	SOX2
PPP6R1	SOX2 regulated genes in Keratinocyte Overexpression from PMID: 30772301	SOX2
TMEM86B	SOX2 regulated genes in Keratinocyte Overexpression from PMID: 30772301	SOX2
AGMAT	SOX2 regulated genes in Keratinocyte Overexpression from PMID: 30772301	SOX2
DYNLRB2	SOX2 regulated genes in Keratinocyte Overexpression from PMID: 30772301	SOX2
HYLS1	SOX2 regulated genes in Keratinocyte Overexpression from PMID: 30772301	SOX2
ZNF398	SOX2 regulated genes in Keratinocyte Overexpression from PMID: 30772301	SOX2
CMSS1	SOX2 regulated genes in Keratinocyte Overexpression from PMID: 30772301	SOX2
CMTM8	SOX2 regulated genes in Keratinocyte Overexpression from PMID: 30772301	SOX2
PDCD7	SOX2 regulated genes in Keratinocyte Overexpression from PMID: 30772301	SOX2
MAP3K10	SOX2 regulated genes in Keratinocyte Overexpression from PMID: 30772301	SOX2
CCDC65	SOX2 regulated genes in Keratinocyte Overexpression from PMID: 30772301	SOX2
RDX	SOX2 regulated genes in Keratinocyte Overexpression from PMID: 30772301	SOX2
NOA1	SOX2 regulated genes in Keratinocyte Overexpression from PMID: 30772301	SOX2
RPA1	SOX2 regulated genes in Keratinocyte Overexpression from PMID: 30772301	SOX2
DOC2A	SOX2 regulated genes in Keratinocyte Overexpression from PMID: 30772301	SOX2
LARGE2	SOX2 regulated genes in Keratinocyte Overexpression from PMID: 30772301	SOX2
GABRE	SOX2 regulated genes in Keratinocyte Overexpression from PMID: 30772301	SOX2
MED13	SOX2 regulated genes in Keratinocyte Overexpression from PMID: 30772301	SOX2
IRF2BP1	SOX2 regulated genes in Keratinocyte Overexpression from PMID: 30772301	SOX2
RBM19	SOX2 regulated genes in Keratinocyte Overexpression from PMID: 30772301	SOX2
AMTN	SOX2 regulated genes in Keratinocyte Overexpression from PMID: 30772301	SOX2
PROM2	SOX2 regulated genes in Keratinocyte Overexpression from PMID: 30772301	SOX2
JUP	SOX2 regulated genes in Keratinocyte Overexpression from PMID: 30772301	SOX2
CTR9	SOX2 regulated genes in Keratinocyte Overexpression from PMID: 30772301	SOX2
FRMPD1	SOX2 regulated genes in Keratinocyte Overexpression from PMID: 30772301	SOX2
SLC5A8	SOX2 regulated genes in Keratinocyte Overexpression from PMID: 30772301	SOX2
HSPA1A	SOX2 regulated genes in Keratinocyte Overexpression from PMID: 30772301	SOX2
HSPA1B	SOX2 regulated genes in Keratinocyte Overexpression from PMID: 30772301	SOX2
PNOC	SOX2 regulated genes in Keratinocyte Overexpression from PMID: 30772301	SOX2
GRHL2	SOX2 regulated genes in Keratinocyte Overexpression from PMID: 30772301	SOX2
KANK1	SOX2 regulated genes in Keratinocyte Overexpression from PMID: 30772301	SOX2
ARFGEF1	SOX2 regulated genes in Keratinocyte Overexpression from PMID: 30772301	SOX2
NOL7	SOX2 regulated genes in Keratinocyte Overexpression from PMID: 30772301	SOX2
NAF1	SOX2 regulated genes in Keratinocyte Overexpression from PMID: 30772301	SOX2
RHCG	SOX2 regulated genes in Keratinocyte Overexpression from PMID: 30772301	SOX2
ATP6V1C1	SOX2 regulated genes in Keratinocyte Overexpression from PMID: 30772301	SOX2
ZNF212	SOX2 regulated genes in Keratinocyte Overexpression from PMID: 30772301	SOX2
MAD1L1	SOX2 regulated genes in Keratinocyte Overexpression from PMID: 30772301	SOX2

GRPEL2	SOX2 regulated genes in Keratinocyte Overexpression from PMID: 30772301	SOX2
UBE3C	SOX2 regulated genes in Keratinocyte Overexpression from PMID: 30772301	SOX2
TMEM147	SOX2 regulated genes in Keratinocyte Overexpression from PMID: 30772301	SOX2
C16orf87	SOX2 regulated genes in Keratinocyte Overexpression from PMID: 30772301	SOX2
DYNLT1	SOX2 regulated genes in Keratinocyte Overexpression from PMID: 30772301	SOX2
NRF1	SOX2 regulated genes in Keratinocyte Overexpression from PMID: 30772301	SOX2
CRHBP	SOX2 regulated genes in Keratinocyte Overexpression from PMID: 30772301	SOX2
SGTB	SOX2 regulated genes in Keratinocyte Overexpression from PMID: 30772301	SOX2
IDI2	SOX2 regulated genes in Keratinocyte Overexpression from PMID: 30772301	SOX2
TIMM17B	SOX2 regulated genes in Keratinocyte Overexpression from PMID: 30772301	SOX2
MATN4	SOX2 regulated genes in Keratinocyte Overexpression from PMID: 30772301	SOX2
COL12A1	SOX2 regulated genes in Keratinocyte Overexpression from PMID: 30772301	SOX2
FBXO32	SOX2 regulated genes in Keratinocyte Overexpression from PMID: 30772301	SOX2
COX6A1	SOX2 regulated genes in Keratinocyte Overexpression from PMID: 30772301	SOX2
EIF3J	SOX2 regulated genes in Keratinocyte Overexpression from PMID: 30772301	SOX2
SOWAHA	SOX2 regulated genes in Keratinocyte Overexpression from PMID: 30772301	SOX2
VCL	SOX2 regulated genes in Keratinocyte Overexpression from PMID: 30772301	SOX2
PHF20L1	SOX2 regulated genes in Keratinocyte Overexpression from PMID: 30772301	SOX2
RAPGEF4	SOX2 regulated genes in Keratinocyte Overexpression from PMID: 30772301	SOX2
METTL17	SOX2 regulated genes in Keratinocyte Overexpression from PMID: 30772301	SOX2
SCRIB	SOX2 regulated genes in Keratinocyte Overexpression from PMID: 30772301	SOX2
SHISA2	SOX2 regulated genes in Keratinocyte Overexpression from PMID: 30772301	SOX2
PBX2	SOX2 regulated genes in Keratinocyte Overexpression from PMID: 30772301	SOX2
SYTL1	SOX2 regulated genes in Keratinocyte Overexpression from PMID: 30772301	SOX2
RNF103	SOX2 regulated genes in Keratinocyte Overexpression from PMID: 30772301	SOX2
KMT5B	SOX2 regulated genes in Keratinocyte Overexpression from PMID: 30772301	SOX2
SMG9	SOX2 regulated genes in Keratinocyte Overexpression from PMID: 30772301	SOX2
ALX1	SOX2 regulated genes in Keratinocyte Overexpression from PMID: 30772301	SOX2
IFT57	SOX2 regulated genes in Keratinocyte Overexpression from PMID: 30772301	SOX2
LRRC74A	SOX2 regulated genes in Keratinocyte Overexpression from PMID: 30772301	SOX2
CPT2	SOX2 regulated genes in Keratinocyte Overexpression from PMID: 30772301	SOX2
NUDT14	SOX2 regulated genes in Keratinocyte Overexpression from PMID: 30772301	SOX2
COPS5	SOX2 regulated genes in Keratinocyte Overexpression from PMID: 30772301	SOX2
LANCL1	SOX2 regulated genes in Keratinocyte Overexpression from PMID: 30772301	SOX2
TTC9C	SOX2 regulated genes in Keratinocyte Overexpression from PMID: 30772301	SOX2
COX18	SOX2 regulated genes in Keratinocyte Overexpression from PMID: 30772301	SOX2
RSPH9	SOX2 regulated genes in Keratinocyte Overexpression from PMID: 30772301	SOX2
SARS2	SOX2 regulated genes in Keratinocyte Overexpression from PMID: 30772301	SOX2
FIS1	SOX2 regulated genes in Keratinocyte Overexpression from PMID: 30772301	SOX2
POLE3	SOX2 regulated genes in Keratinocyte Overexpression from PMID: 30772301	SOX2
SSNA1	SOX2 regulated genes in Keratinocyte Overexpression from PMID: 30772301	SOX2
GPAA1	SOX2 regulated genes in Keratinocyte Overexpression from PMID: 30772301	SOX2
MRE11	SOX2 regulated genes in Keratinocyte Overexpression from PMID: 30772301	SOX2
UBE3A	SOX2 regulated genes in Keratinocyte Overexpression from PMID: 30772301	SOX2
SPOP	SOX2 regulated genes in Keratinocyte Overexpression from PMID: 30772301	SOX2
POC5	SOX2 regulated genes in Keratinocyte Overexpression from PMID: 30772301	SOX2
HSPA5	SOX2 regulated genes in Keratinocyte Overexpression from PMID: 30772301	SOX2
PDCL3	SOX2 regulated genes in Keratinocyte Overexpression from PMID: 30772301	SOX2
PSMA2	SOX2 regulated genes in Keratinocyte Overexpression from PMID: 30772301	SOX2
ALDH5A1	SOX2 regulated genes in Keratinocyte Overexpression from PMID: 30772301	SOX2
MRPS23	SOX2 regulated genes in Keratinocyte Overexpression from PMID: 30772301	SOX2
TFB1M	SOX2 regulated genes in Keratinocyte Overexpression from PMID: 30772301	SOX2
PANK3	SOX2 regulated genes in Keratinocyte Overexpression from PMID: 30772301	SOX2
CDK12	SOX2 regulated genes in Keratinocyte Overexpression from PMID: 30772301	SOX2
CCDC60	SOX2 regulated genes in Keratinocyte Overexpression from PMID: 30772301	SOX2
DAPK2	SOX2 regulated genes in Keratinocyte Overexpression from PMID: 30772301	SOX2
LAS1L	SOX2 regulated genes in Keratinocyte Overexpression from PMID: 30772301	SOX2
DCTD	SOX2 regulated genes in Keratinocyte Overexpression from PMID: 30772301	SOX2
PCYOX1L	SOX2 regulated genes in Keratinocyte Overexpression from PMID: 30772301	SOX2

NUDCD2	SOX2 regulated genes in Keratinocyte Overexpression from PMID: 30772301	SOX2
MCM6	SOX2 regulated genes in Keratinocyte Overexpression from PMID: 30772301	SOX2
CPA5	SOX2 regulated genes in Keratinocyte Overexpression from PMID: 30772301	SOX2
HES5	SOX2 regulated genes in Keratinocyte Overexpression from PMID: 30772301	SOX2
APOL3	SOX2 regulated genes in Keratinocyte Overexpression from PMID: 30772301	SOX2
APOL4	SOX2 regulated genes in Keratinocyte Overexpression from PMID: 30772301	SOX2
APOL2	SOX2 regulated genes in Keratinocyte Overexpression from PMID: 30772301	SOX2
APOL1	SOX2 regulated genes in Keratinocyte Overexpression from PMID: 30772301	SOX2
MPZL2	SOX2 regulated genes in Keratinocyte Overexpression from PMID: 30772301	SOX2
SPRED2	SOX2 regulated genes in Keratinocyte Overexpression from PMID: 30772301	SOX2
RPEL1	SOX2 regulated genes in Keratinocyte Overexpression from PMID: 30772301	SOX2
RPE	SOX2 regulated genes in Keratinocyte Overexpression from PMID: 30772301	SOX2
CFL1	SOX2 regulated genes in Keratinocyte Overexpression from PMID: 30772301	SOX2
DUSP19	SOX2 regulated genes in Keratinocyte Overexpression from PMID: 30772301	SOX2
CCDC15	SOX2 regulated genes in Keratinocyte Overexpression from PMID: 30772301	SOX2
MAPK8IP1	SOX2 regulated genes in Keratinocyte Overexpression from PMID: 30772301	SOX2
CAPN1	SOX2 regulated genes in Keratinocyte Overexpression from PMID: 30772301	SOX2
TECTA	SOX2 regulated genes in Keratinocyte Overexpression from PMID: 30772301	SOX2
ELP3	SOX2 regulated genes in Keratinocyte Overexpression from PMID: 30772301	SOX2
PPAT	SOX2 regulated genes in Keratinocyte Overexpression from PMID: 30772301	SOX2
RFX3	SOX2 regulated genes in Keratinocyte Overexpression from PMID: 30772301	SOX2
FAM227A	SOX2 regulated genes in Keratinocyte Overexpression from PMID: 30772301	SOX2
MRPL18	SOX2 regulated genes in Keratinocyte Overexpression from PMID: 30772301	SOX2
TRIM2	SOX2 regulated genes in Keratinocyte Overexpression from PMID: 30772301	SOX2
ASAP2	SOX2 regulated genes in Keratinocyte Overexpression from PMID: 30772301	SOX2
SIAH1	SOX2 regulated genes in Keratinocyte Overexpression from PMID: 30772301	SOX2
ATP1B3	SOX2 regulated genes in Keratinocyte Overexpression from PMID: 30772301	SOX2
CYB5D1	SOX2 regulated genes in Keratinocyte Overexpression from PMID: 30772301	SOX2
SRF	SOX2 regulated genes in Keratinocyte Overexpression from PMID: 30772301	SOX2
CPSF1	SOX2 regulated genes in Keratinocyte Overexpression from PMID: 30772301	SOX2
DTWD2	SOX2 regulated genes in Keratinocyte Overexpression from PMID: 30772301	SOX2
SEC24C	SOX2 regulated genes in Keratinocyte Overexpression from PMID: 30772301	SOX2
GNE	SOX2 regulated genes in Keratinocyte Overexpression from PMID: 30772301	SOX2
PTCH1	SOX2 regulated genes in Keratinocyte Overexpression from PMID: 30772301	SOX2
ZNF282	SOX2 regulated genes in Keratinocyte Overexpression from PMID: 30772301	SOX2
MTOR	SOX2 regulated genes in Keratinocyte Overexpression from PMID: 30772301	SOX2
SUPT5H	SOX2 regulated genes in Keratinocyte Overexpression from PMID: 30772301	SOX2
ATP8A2	SOX2 regulated genes in Keratinocyte Overexpression from PMID: 30772301	SOX2
GDPD3	SOX2 regulated genes in Keratinocyte Overexpression from PMID: 30772301	SOX2
IFFO2	SOX2 regulated genes in Keratinocyte Overexpression from PMID: 30772301	SOX2
RCN1	SOX2 regulated genes in Keratinocyte Overexpression from PMID: 30772301	SOX2
MBTD1	SOX2 regulated genes in Keratinocyte Overexpression from PMID: 30772301	SOX2
GCSH	SOX2 regulated genes in Keratinocyte Overexpression from PMID: 30772301	SOX2
SLBP	SOX2 regulated genes in Keratinocyte Overexpression from PMID: 30772301	SOX2
DOCK9	SOX2 regulated genes in Keratinocyte Overexpression from PMID: 30772301	SOX2
COQ5	SOX2 regulated genes in Keratinocyte Overexpression from PMID: 30772301	SOX2
POLR2G	SOX2 regulated genes in Keratinocyte Overexpression from PMID: 30772301	SOX2
WDR20	SOX2 regulated genes in Keratinocyte Overexpression from PMID: 30772301	SOX2
RBM28	SOX2 regulated genes in Keratinocyte Overexpression from PMID: 30772301	SOX2
MRPS30	SOX2 regulated genes in Keratinocyte Overexpression from PMID: 30772301	SOX2
PWWP2B	SOX2 regulated genes in Keratinocyte Overexpression from PMID: 30772301	SOX2
AP4S1	SOX2 regulated genes in Keratinocyte Overexpression from PMID: 30772301	SOX2
ALCAM	SOX2 regulated genes in Keratinocyte Overexpression from PMID: 30772301	SOX2
JMJD7-PLA2G4B	SOX2 regulated genes in Keratinocyte Overexpression from PMID: 30772301	SOX2
HYAL4	SOX2 regulated genes in Keratinocyte Overexpression from PMID: 30772301	SOX2
NUBP1	SOX2 regulated genes in Keratinocyte Overexpression from PMID: 30772301	SOX2
HADHB	SOX2 regulated genes in Keratinocyte Overexpression from PMID: 30772301	SOX2
SPINK9	SOX2 regulated genes in Keratinocyte Overexpression from PMID: 30772301	SOX2
CCP110	SOX2 regulated genes in Keratinocyte Overexpression from PMID: 30772301	SOX2

FAAP100	SOX2 regulated genes in Keratinocyte Overexpression from PMID: 30772301	SOX2
JAKMIP2	SOX2 regulated genes in Keratinocyte Overexpression from PMID: 30772301	SOX2
DMTF1	SOX2 regulated genes in Keratinocyte Overexpression from PMID: 30772301	SOX2
NDOR1	SOX2 regulated genes in Keratinocyte Overexpression from PMID: 30772301	SOX2
UBE3D	SOX2 regulated genes in Keratinocyte Overexpression from PMID: 30772301	SOX2
ANAPC1	SOX2 regulated genes in Keratinocyte Overexpression from PMID: 30772301	SOX2
CALML3	SOX2 regulated genes in Keratinocyte Overexpression from PMID: 30772301	SOX2
HSPA8	SOX2 regulated genes in Keratinocyte Overexpression from PMID: 30772301	SOX2
SF3B5	SOX2 regulated genes in Keratinocyte Overexpression from PMID: 30772301	SOX2
FOSL2	SOX2 regulated genes in Keratinocyte Overexpression from PMID: 30772301	SOX2
MREG	SOX2 regulated genes in Keratinocyte Overexpression from PMID: 30772301	SOX2
DNPH1	SOX2 regulated genes in Keratinocyte Overexpression from PMID: 30772301	SOX2
GPC2	SOX2 regulated genes in Keratinocyte Overexpression from PMID: 30772301	SOX2
MTMR2	SOX2 regulated genes in Keratinocyte Overexpression from PMID: 30772301	SOX2
TOP2A	SOX2 regulated genes in Keratinocyte Overexpression from PMID: 30772301	SOX2
WDR70	SOX2 regulated genes in Keratinocyte Overexpression from PMID: 30772301	SOX2
PEX6	SOX2 regulated genes in Keratinocyte Overexpression from PMID: 30772301	SOX2
ADAT2	SOX2 regulated genes in Keratinocyte Overexpression from PMID: 30772301	SOX2
LYPD5	SOX2 regulated genes in Keratinocyte Overexpression from PMID: 30772301	SOX2
PTPN1	SOX2 regulated genes in Keratinocyte Overexpression from PMID: 30772301	SOX2
C10orf95	SOX2 regulated genes in Keratinocyte Overexpression from PMID: 30772301	SOX2
TNKS1BP1	SOX2 regulated genes in Keratinocyte Overexpression from PMID: 30772301	SOX2
SLC22A23	SOX2 regulated genes in Keratinocyte Overexpression from PMID: 30772301	SOX2
CACFD1	SOX2 regulated genes in Keratinocyte Overexpression from PMID: 30772301	SOX2
SUGP1	SOX2 regulated genes in Keratinocyte Overexpression from PMID: 30772301	SOX2
ZNF786	SOX2 regulated genes in Keratinocyte Overexpression from PMID: 30772301	SOX2
EHD1	SOX2 regulated genes in Keratinocyte Overexpression from PMID: 30772301	SOX2
ARG1	SOX2 regulated genes in Keratinocyte Overexpression from PMID: 30772301	SOX2
WNT10B	SOX2 regulated genes in Keratinocyte Overexpression from PMID: 30772301	SOX2
LIG1	SOX2 regulated genes in Keratinocyte Overexpression from PMID: 30772301	SOX2
HILPDA	SOX2 regulated genes in Keratinocyte Overexpression from PMID: 30772301	SOX2
RABGAP1	SOX2 regulated genes in Keratinocyte Overexpression from PMID: 30772301	SOX2
EDAR	SOX2 regulated genes in Keratinocyte Overexpression from PMID: 30772301	SOX2
HADHA	SOX2 regulated genes in Keratinocyte Overexpression from PMID: 30772301	SOX2
ADM	SOX2 regulated genes in Keratinocyte Overexpression from PMID: 30772301	SOX2
TMCO3	SOX2 regulated genes in Keratinocyte Overexpression from PMID: 30772301	SOX2
HNF4G	SOX2 regulated genes in Keratinocyte Overexpression from PMID: 30772301	SOX2
SNIP1	SOX2 regulated genes in Keratinocyte Overexpression from PMID: 30772301	SOX2
KBTBD6	SOX2 regulated genes in Keratinocyte Overexpression from PMID: 30772301	SOX2
KBTBD7	SOX2 regulated genes in Keratinocyte Overexpression from PMID: 30772301	SOX2
PAX1	SOX2 regulated genes in Keratinocyte Overexpression from PMID: 30772301	SOX2
TOMM6	SOX2 regulated genes in Keratinocyte Overexpression from PMID: 30772301	SOX2
ATP10B	SOX2 regulated genes in Keratinocyte Overexpression from PMID: 30772301	SOX2
KAT5	SOX2 regulated genes in Keratinocyte Overexpression from PMID: 30772301	SOX2
MTERF4	SOX2 regulated genes in Keratinocyte Overexpression from PMID: 30772301	SOX2
MAP3K15	SOX2 regulated genes in Keratinocyte Overexpression from PMID: 30772301	SOX2
NIPAL1	SOX2 regulated genes in Keratinocyte Overexpression from PMID: 30772301	SOX2
PUS10	SOX2 regulated genes in Keratinocyte Overexpression from PMID: 30772301	SOX2
ANAPC5	SOX2 regulated genes in Keratinocyte Overexpression from PMID: 30772301	SOX2
RDH10	SOX2 regulated genes in Keratinocyte Overexpression from PMID: 30772301	SOX2
NOP14	SOX2 regulated genes in Keratinocyte Overexpression from PMID: 30772301	SOX2
SPATA20	SOX2 regulated genes in Keratinocyte Overexpression from PMID: 30772301	SOX2
TACSTD2	SOX2 regulated genes in Keratinocyte Overexpression from PMID: 30772301	SOX2
MED29	SOX2 regulated genes in Keratinocyte Overexpression from PMID: 30772301	SOX2
DMAP1	SOX2 regulated genes in Keratinocyte Overexpression from PMID: 30772301	SOX2
JOSD2	SOX2 regulated genes in Keratinocyte Overexpression from PMID: 30772301	SOX2
TMPRSS13	SOX2 regulated genes in Keratinocyte Overexpression from PMID: 30772301	SOX2
KIF26A	SOX2 regulated genes in Keratinocyte Overexpression from PMID: 30772301	SOX2
TRPV6	SOX2 regulated genes in Keratinocyte Overexpression from PMID: 30772301	SOX2

CDKN3	SOX2 regulated genes in Keratinocyte Overexpression from PMID: 30772301	SOX2
TTLL10	SOX2 regulated genes in Keratinocyte Overexpression from PMID: 30772301	SOX2
RTN4IP1	SOX2 regulated genes in Keratinocyte Overexpression from PMID: 30772301	SOX2
TMEM35A	SOX2 regulated genes in Keratinocyte Overexpression from PMID: 30772301	SOX2
TRIB1	SOX2 regulated genes in Keratinocyte Overexpression from PMID: 30772301	SOX2
IZUMO1	SOX2 regulated genes in Keratinocyte Overexpression from PMID: 30772301	SOX2
TMEM134	SOX2 regulated genes in Keratinocyte Overexpression from PMID: 30772301	SOX2
ABCB8	SOX2 regulated genes in Keratinocyte Overexpression from PMID: 30772301	SOX2
YEATS4	SOX2 regulated genes in Keratinocyte Overexpression from PMID: 30772301	SOX2
RAD54B	SOX2 regulated genes in Keratinocyte Overexpression from PMID: 30772301	SOX2
SCUBE1	SOX2 regulated genes in Keratinocyte Overexpression from PMID: 30772301	SOX2
OGDHL	SOX2 regulated genes in Keratinocyte Overexpression from PMID: 30772301	SOX2
SCNN1G	SOX2 regulated genes in Keratinocyte Overexpression from PMID: 30772301	SOX2
CEBPB	SOX2 regulated genes in Keratinocyte Overexpression from PMID: 30772301	SOX2
RAD51C	SOX2 regulated genes in Keratinocyte Overexpression from PMID: 30772301	SOX2
SERPINA1	SOX2 regulated genes in Keratinocyte Overexpression from PMID: 30772301	SOX2
NIPAL2	SOX2 regulated genes in Keratinocyte Overexpression from PMID: 30772301	SOX2
PTPRK	SOX2 regulated genes in Keratinocyte Overexpression from PMID: 30772301	SOX2
B3GNT4	SOX2 regulated genes in Keratinocyte Overexpression from PMID: 30772301	SOX2
ALDH3A2	SOX2 regulated genes in Keratinocyte Overexpression from PMID: 30772301	SOX2
KITLG	SOX2 regulated genes in Keratinocyte Overexpression from PMID: 30772301	SOX2
PRRC2B	SOX2 regulated genes in Keratinocyte Overexpression from PMID: 30772301	SOX2
HAT1	SOX2 regulated genes in Keratinocyte Overexpression from PMID: 30772301	SOX2
NDST1	SOX2 regulated genes in Keratinocyte Overexpression from PMID: 30772301	SOX2
SLC25A13	SOX2 regulated genes in Keratinocyte Overexpression from PMID: 30772301	SOX2
FAM102A	SOX2 regulated genes in Keratinocyte Overexpression from PMID: 30772301	SOX2
CCND2	SOX2 regulated genes in Keratinocyte Overexpression from PMID: 30772301	SOX2
PAFAH1B2	SOX2 regulated genes in Keratinocyte Overexpression from PMID: 30772301	SOX2
UBE2E3	SOX2 regulated genes in Keratinocyte Overexpression from PMID: 30772301	SOX2
ZNF169	SOX2 regulated genes in Keratinocyte Overexpression from PMID: 30772301	SOX2
SLC4A2	SOX2 regulated genes in Keratinocyte Overexpression from PMID: 30772301	SOX2
SLC22A1	SOX2 regulated genes in Keratinocyte Overexpression from PMID: 30772301	SOX2
SFRP1	SOX2 regulated genes in Keratinocyte Overexpression from PMID: 30772301	SOX2
E2F5	SOX2 regulated genes in Keratinocyte Overexpression from PMID: 30772301	SOX2
CHRM5	SOX2 regulated genes in Keratinocyte Overexpression from PMID: 30772301	SOX2
GTF3C1	SOX2 regulated genes in Keratinocyte Overexpression from PMID: 30772301	SOX2
NXT1	SOX2 regulated genes in Keratinocyte Overexpression from PMID: 30772301	SOX2
TULP1	SOX2 regulated genes in Keratinocyte Overexpression from PMID: 30772301	SOX2
EPHB6	SOX2 regulated genes in Keratinocyte Overexpression from PMID: 30772301	SOX2
ATOH8	SOX2 regulated genes in Keratinocyte Overexpression from PMID: 30772301	SOX2
HAUS5	SOX2 regulated genes in Keratinocyte Overexpression from PMID: 30772301	SOX2
RABGEF1	SOX2 regulated genes in Keratinocyte Overexpression from PMID: 30772301	SOX2
ZMYM2	SOX2 regulated genes in Keratinocyte Overexpression from PMID: 30772301	SOX2
STBD1	SOX2 regulated genes in Keratinocyte Overexpression from PMID: 30772301	SOX2
ZDHHC6	SOX2 regulated genes in Keratinocyte Overexpression from PMID: 30772301	SOX2
MAD2L1BP	SOX2 regulated genes in Keratinocyte Overexpression from PMID: 30772301	SOX2
PSMB2	SOX2 regulated genes in Keratinocyte Overexpression from PMID: 30772301	SOX2
DDX51	SOX2 regulated genes in Keratinocyte Overexpression from PMID: 30772301	SOX2
MED7	SOX2 regulated genes in Keratinocyte Overexpression from PMID: 30772301	SOX2
NECTIN3	SOX2 regulated genes in Keratinocyte Overexpression from PMID: 30772301	SOX2
KRT6A	SOX2 regulated genes in Keratinocyte Overexpression from PMID: 30772301	SOX2
CNOT6	SOX2 regulated genes in Keratinocyte Overexpression from PMID: 30772301	SOX2
ZBTB39	SOX2 regulated genes in Keratinocyte Overexpression from PMID: 30772301	SOX2
STAG2	SOX2 regulated genes in Keratinocyte Overexpression from PMID: 30772301	SOX2
ZSCAN25	SOX2 regulated genes in Keratinocyte Overexpression from PMID: 30772301	SOX2
RHOG	SOX2 regulated genes in Keratinocyte Overexpression from PMID: 30772301	SOX2
CYP4F12	SOX2 regulated genes in Keratinocyte Overexpression from PMID: 30772301	SOX2
KAT2B	SOX2 regulated genes in Keratinocyte Overexpression from PMID: 30772301	SOX2
ANAPC2	SOX2 regulated genes in Keratinocyte Overexpression from PMID: 30772301	SOX2

FRAS1	SOX2 regulated genes in Keratinocyte Overexpression from PMID: 30772301	SOX2
SLX1B	SOX2 regulated genes in Keratinocyte Overexpression from PMID: 30772301	SOX2
SLX1A	SOX2 regulated genes in Keratinocyte Overexpression from PMID: 30772301	SOX2
CKAP2L	SOX2 regulated genes in Keratinocyte Overexpression from PMID: 30772301	SOX2
CDCP1	SOX2 regulated genes in Keratinocyte Overexpression from PMID: 30772301	SOX2
NRARP	SOX2 regulated genes in Keratinocyte Overexpression from PMID: 30772301	SOX2
PNO1	SOX2 regulated genes in Keratinocyte Overexpression from PMID: 30772301	SOX2
VCP	SOX2 regulated genes in Keratinocyte Overexpression from PMID: 30772301	SOX2
HECW2	SOX2 regulated genes in Keratinocyte Overexpression from PMID: 30772301	SOX2
ABHD17B	SOX2 regulated genes in Keratinocyte Overexpression from PMID: 30772301	SOX2
DNTTIP2	SOX2 regulated genes in Keratinocyte Overexpression from PMID: 30772301	SOX2
LIPM	SOX2 regulated genes in Keratinocyte Overexpression from PMID: 30772301	SOX2
HOXB8	SOX2 regulated genes in Keratinocyte Overexpression from PMID: 30772301	SOX2
PARN	SOX2 regulated genes in Keratinocyte Overexpression from PMID: 30772301	SOX2
SCUBE3	SOX2 regulated genes in Keratinocyte Overexpression from PMID: 30772301	SOX2
RALGAPA1	SOX2 regulated genes in Keratinocyte Overexpression from PMID: 30772301	SOX2
TIMM10	SOX2 regulated genes in Keratinocyte Overexpression from PMID: 30772301	SOX2
ANAPC10	SOX2 regulated genes in Keratinocyte Overexpression from PMID: 30772301	SOX2
CXXC5	SOX2 regulated genes in Keratinocyte Overexpression from PMID: 30772301	SOX2
CTCF	SOX2 regulated genes in Keratinocyte Overexpression from PMID: 30772301	SOX2
EEF1A1	SOX2 regulated genes in Keratinocyte Overexpression from PMID: 30772301	SOX2
UACA	SOX2 regulated genes in Keratinocyte Overexpression from PMID: 30772301	SOX2
FAF2	SOX2 regulated genes in Keratinocyte Overexpression from PMID: 30772301	SOX2
SUB1	SOX2 regulated genes in Keratinocyte Overexpression from PMID: 30772301	SOX2
SCNN1B	SOX2 regulated genes in Keratinocyte Overexpression from PMID: 30772301	SOX2
WAC	SOX2 regulated genes in Keratinocyte Overexpression from PMID: 30772301	SOX2
SNUPN	SOX2 regulated genes in Keratinocyte Overexpression from PMID: 30772301	SOX2
USP34	SOX2 regulated genes in Keratinocyte Overexpression from PMID: 30772301	SOX2
SKIDA1	SOX2 regulated genes in Keratinocyte Overexpression from PMID: 30772301	SOX2
BICD1	SOX2 regulated genes in Keratinocyte Overexpression from PMID: 30772301	SOX2
TOB1	SOX2 regulated genes in Keratinocyte Overexpression from PMID: 30772301	SOX2
TANGO6	SOX2 regulated genes in Keratinocyte Overexpression from PMID: 30772301	SOX2
BAALC	SOX2 regulated genes in Keratinocyte Overexpression from PMID: 30772301	SOX2
FOXK2	SOX2 regulated genes in Keratinocyte Overexpression from PMID: 30772301	SOX2
FOXA3	SOX2 regulated genes in Keratinocyte Overexpression from PMID: 30772301	SOX2
MKRN1	SOX2 regulated genes in Keratinocyte Overexpression from PMID: 30772301	SOX2
GRM7	SOX2 regulated genes in Keratinocyte Overexpression from PMID: 30772301	SOX2
PHPT1	SOX2 regulated genes in Keratinocyte Overexpression from PMID: 30772301	SOX2
CPEB2	SOX2 regulated genes in Keratinocyte Overexpression from PMID: 30772301	SOX2
YWHAZ	SOX2 regulated genes in Keratinocyte Overexpression from PMID: 30772301	SOX2
RPP25L	SOX2 regulated genes in Keratinocyte Overexpression from PMID: 30772301	SOX2
TMEM11	SOX2 regulated genes in Keratinocyte Overexpression from PMID: 30772301	SOX2
MRPL11	SOX2 regulated genes in Keratinocyte Overexpression from PMID: 30772301	SOX2
ZNF276	SOX2 regulated genes in Keratinocyte Overexpression from PMID: 30772301	SOX2
RALA	SOX2 regulated genes in Keratinocyte Overexpression from PMID: 30772301	SOX2
NUP188	SOX2 regulated genes in Keratinocyte Overexpression from PMID: 30772301	SOX2
PTPRF	SOX2 regulated genes in Keratinocyte Overexpression from PMID: 30772301	SOX2
MEST	SOX2 regulated genes in Keratinocyte Overexpression from PMID: 30772301	SOX2
POLD4	SOX2 regulated genes in Keratinocyte Overexpression from PMID: 30772301	SOX2
SLITRK5	SOX2 regulated genes in Keratinocyte Overexpression from PMID: 30772301	SOX2
N4BP2L2	SOX2 regulated genes in Keratinocyte Overexpression from PMID: 30772301	SOX2
ACP7	SOX2 regulated genes in Keratinocyte Overexpression from PMID: 30772301	SOX2
HBP1	SOX2 regulated genes in Keratinocyte Overexpression from PMID: 30772301	SOX2
LRRTM2	SOX2 regulated genes in Keratinocyte Overexpression from PMID: 30772301	SOX2
RRN3	SOX2 regulated genes in Keratinocyte Overexpression from PMID: 30772301	SOX2
HEPH	SOX2 regulated genes in Keratinocyte Overexpression from PMID: 30772301	SOX2
MOB1B	SOX2 regulated genes in Keratinocyte Overexpression from PMID: 30772301	SOX2
BRCC3	SOX2 regulated genes in Keratinocyte Overexpression from PMID: 30772301	SOX2
OPA3	SOX2 regulated genes in Keratinocyte Overexpression from PMID: 30772301	SOX2

UBA1	SOX2 regulated genes in Keratinocyte Overexpression from PMID: 30772301	SOX2
FBXO10	SOX2 regulated genes in Keratinocyte Overexpression from PMID: 30772301	SOX2
MPZL3	SOX2 regulated genes in Keratinocyte Overexpression from PMID: 30772301	SOX2
ORMDL2	SOX2 regulated genes in Keratinocyte Overexpression from PMID: 30772301	SOX2
PRR3	SOX2 regulated genes in Keratinocyte Overexpression from PMID: 30772301	SOX2
TSPAN3	SOX2 regulated genes in Keratinocyte Overexpression from PMID: 30772301	SOX2
CBLN3	SOX2 regulated genes in Keratinocyte Overexpression from PMID: 30772301	SOX2
LETM1	SOX2 regulated genes in Keratinocyte Overexpression from PMID: 30772301	SOX2
RC3H2	SOX2 regulated genes in Keratinocyte Overexpression from PMID: 30772301	SOX2
CX3CL1	SOX2 regulated genes in Keratinocyte Overexpression from PMID: 30772301	SOX2
FLRT2	SOX2 regulated genes in Keratinocyte Overexpression from PMID: 30772301	SOX2
GEN1	SOX2 regulated genes in Keratinocyte Overexpression from PMID: 30772301	SOX2
BCL11A	SOX2 regulated genes in Keratinocyte Overexpression from PMID: 30772301	SOX2
TMEM178A	SOX2 regulated genes in Keratinocyte Overexpression from PMID: 30772301	SOX2
EXOSC7	SOX2 regulated genes in Keratinocyte Overexpression from PMID: 30772301	SOX2
LBH	SOX2 regulated genes in Keratinocyte Overexpression from PMID: 30772301	SOX2
COPS7A	SOX2 regulated genes in Keratinocyte Overexpression from PMID: 30772301	SOX2
RBMX2	SOX2 regulated genes in Keratinocyte Overexpression from PMID: 30772301	SOX2
NRBP2	SOX2 regulated genes in Keratinocyte Overexpression from PMID: 30772301	SOX2
KLRG2	SOX2 regulated genes in Keratinocyte Overexpression from PMID: 30772301	SOX2
DPH3	SOX2 regulated genes in Keratinocyte Overexpression from PMID: 30772301	SOX2
VPS37D	SOX2 regulated genes in Keratinocyte Overexpression from PMID: 30772301	SOX2
PLG	SOX2 regulated genes in Keratinocyte Overexpression from PMID: 30772301	SOX2
C1QTNF4	SOX2 regulated genes in Keratinocyte Overexpression from PMID: 30772301	SOX2
CSRP2	SOX2 regulated genes in Keratinocyte Overexpression from PMID: 30772301	SOX2
EFCAB7	SOX2 regulated genes in Keratinocyte Overexpression from PMID: 30772301	SOX2
TEAD2	SOX2 regulated genes in Keratinocyte Overexpression from PMID: 30772301	SOX2
HERC4	SOX2 regulated genes in Keratinocyte Overexpression from PMID: 30772301	SOX2
GLRX3	SOX2 regulated genes in Keratinocyte Overexpression from PMID: 30772301	SOX2
SLC16A8	SOX2 regulated genes in Keratinocyte Overexpression from PMID: 30772301	SOX2
NFIB	SOX2 regulated genes in Keratinocyte Overexpression from PMID: 30772301	SOX2
NUP35	SOX2 regulated genes in Keratinocyte Overexpression from PMID: 30772301	SOX2
PPARD	SOX2 regulated genes in Keratinocyte Overexpression from PMID: 30772301	SOX2
GLA	SOX2 regulated genes in Keratinocyte Overexpression from PMID: 30772301	SOX2
SRSF12	SOX2 regulated genes in Keratinocyte Overexpression from PMID: 30772301	SOX2
COPS6	SOX2 regulated genes in Keratinocyte Overexpression from PMID: 30772301	SOX2
SH3D19	SOX2 regulated genes in Keratinocyte Overexpression from PMID: 30772301	SOX2
SYCP3	SOX2 regulated genes in Keratinocyte Overexpression from PMID: 30772301	SOX2
GZF1	SOX2 regulated genes in Keratinocyte Overexpression from PMID: 30772301	SOX2
AGAP3	SOX2 regulated genes in Keratinocyte Overexpression from PMID: 30772301	SOX2
JAG2	SOX2 regulated genes in Keratinocyte Overexpression from PMID: 30772301	SOX2
TLE4	SOX2 regulated genes in Keratinocyte Overexpression from PMID: 30772301	SOX2
SNX33	SOX2 regulated genes in Keratinocyte Overexpression from PMID: 30772301	SOX2
ALKBH4	SOX2 regulated genes in Keratinocyte Overexpression from PMID: 30772301	SOX2
GPC4	SOX2 regulated genes in Keratinocyte Overexpression from PMID: 30772301	SOX2
BRAP	SOX2 regulated genes in Keratinocyte Overexpression from PMID: 30772301	SOX2
HS6ST1	SOX2 regulated genes in Keratinocyte Overexpression from PMID: 30772301	SOX2
MPHOSPH10	SOX2 regulated genes in Keratinocyte Overexpression from PMID: 30772301	SOX2
KIAA0586	SOX2 regulated genes in Keratinocyte Overexpression from PMID: 30772301	SOX2
HSF1	SOX2 regulated genes in Keratinocyte Overexpression from PMID: 30772301	SOX2
DPYSL5	SOX2 regulated genes in Keratinocyte Overexpression from PMID: 30772301	SOX2
ADO	SOX2 regulated genes in Keratinocyte Overexpression from PMID: 30772301	SOX2
PDIK1L	SOX2 regulated genes in Keratinocyte Overexpression from PMID: 30772301	SOX2
PPFIA3	SOX2 regulated genes in Keratinocyte Overexpression from PMID: 30772301	SOX2
TSSK1B	SOX2 regulated genes in Keratinocyte Overexpression from PMID: 30772301	SOX2
DUSP6	SOX2 regulated genes in Keratinocyte Overexpression from PMID: 30772301	SOX2
PPIL4	SOX2 regulated genes in Keratinocyte Overexpression from PMID: 30772301	SOX2
SMNDC1	SOX2 regulated genes in Keratinocyte Overexpression from PMID: 30772301	SOX2
SOSTDC1	SOX2 regulated genes in Keratinocyte Overexpression from PMID: 30772301	SOX2

NUP98	SOX2 regulated genes in Keratinocyte Overexpression from PMID: 30772301	SOX2
TMEM121	SOX2 regulated genes in Keratinocyte Overexpression from PMID: 30772301	SOX2
GJB4	SOX2 regulated genes in Keratinocyte Overexpression from PMID: 30772301	SOX2
RPL37	SOX2 regulated genes in Keratinocyte Overexpression from PMID: 30772301	SOX2
MAP9	SOX2 regulated genes in Keratinocyte Overexpression from PMID: 30772301	SOX2
MTTP	SOX2 regulated genes in Keratinocyte Overexpression from PMID: 30772301	SOX2
IQCK	SOX2 regulated genes in Keratinocyte Overexpression from PMID: 30772301	SOX2
PPP2R5D	SOX2 regulated genes in Keratinocyte Overexpression from PMID: 30772301	SOX2
NUP37	SOX2 regulated genes in Keratinocyte Overexpression from PMID: 30772301	SOX2
MLLT10	SOX2 regulated genes in Keratinocyte Overexpression from PMID: 30772301	SOX2
FNTB	SOX2 regulated genes in Keratinocyte Overexpression from PMID: 30772301	SOX2
PPP1R15A	SOX2 regulated genes in Keratinocyte Overexpression from PMID: 30772301	SOX2
PNPLA1	SOX2 regulated genes in Keratinocyte Overexpression from PMID: 30772301	SOX2
RPL22L1	SOX2 regulated genes in Keratinocyte Overexpression from PMID: 30772301	SOX2
NRN1L	SOX2 regulated genes in Keratinocyte Overexpression from PMID: 30772301	SOX2
EEFSEC	SOX2 regulated genes in Keratinocyte Overexpression from PMID: 30772301	SOX2
PPID	SOX2 regulated genes in Keratinocyte Overexpression from PMID: 30772301	SOX2
ARMC10	SOX2 regulated genes in Keratinocyte Overexpression from PMID: 30772301	SOX2
ZBTB45	SOX2 regulated genes in Keratinocyte Overexpression from PMID: 30772301	SOX2
NXPE2	SOX2 regulated genes in Keratinocyte Overexpression from PMID: 30772301	SOX2
PSMD14	SOX2 regulated genes in Keratinocyte Overexpression from PMID: 30772301	SOX2
KIF20B	SOX2 regulated genes in Keratinocyte Overexpression from PMID: 30772301	SOX2
TMEM161A	SOX2 regulated genes in Keratinocyte Overexpression from PMID: 30772301	SOX2
CCDC112	SOX2 regulated genes in Keratinocyte Overexpression from PMID: 30772301	SOX2
PRRG3	SOX2 regulated genes in Keratinocyte Overexpression from PMID: 30772301	SOX2
PUS3	SOX2 regulated genes in Keratinocyte Overexpression from PMID: 30772301	SOX2
SYNE2	SOX2 regulated genes in Keratinocyte Overexpression from PMID: 30772301	SOX2
ZBTB12	SOX2 regulated genes in Keratinocyte Overexpression from PMID: 30772301	SOX2
REXO4	SOX2 regulated genes in Keratinocyte Overexpression from PMID: 30772301	SOX2
WDR31	SOX2 regulated genes in Keratinocyte Overexpression from PMID: 30772301	SOX2
AP1S3	SOX2 regulated genes in Keratinocyte Overexpression from PMID: 30772301	SOX2
GTF3C5	SOX2 regulated genes in Keratinocyte Overexpression from PMID: 30772301	SOX2
PTK2	SOX2 regulated genes in Keratinocyte Overexpression from PMID: 30772301	SOX2
LRFN1	SOX2 regulated genes in Keratinocyte Overexpression from PMID: 30772301	SOX2
CCNC	SOX2 regulated genes in Keratinocyte Overexpression from PMID: 30772301	SOX2
PLA2G12A	SOX2 regulated genes in Keratinocyte Overexpression from PMID: 30772301	SOX2
ARID1B	SOX2 regulated genes in Keratinocyte Overexpression from PMID: 30772301	SOX2
SOCS5	SOX2 regulated genes in Keratinocyte Overexpression from PMID: 30772301	SOX2
FANCA	SOX2 regulated genes in Keratinocyte Overexpression from PMID: 30772301	SOX2
NAXD	SOX2 regulated genes in Keratinocyte Overexpression from PMID: 30772301	SOX2
CLIC3	SOX2 regulated genes in Keratinocyte Overexpression from PMID: 30772301	SOX2
ABHD10	SOX2 regulated genes in Keratinocyte Overexpression from PMID: 30772301	SOX2
INSM2	SOX2 regulated genes in Keratinocyte Overexpression from PMID: 30772301	SOX2
MYCBPAP	SOX2 regulated genes in Keratinocyte Overexpression from PMID: 30772301	SOX2
TEAD3	SOX2 regulated genes in Keratinocyte Overexpression from PMID: 30772301	SOX2
PTK2B	SOX2 regulated genes in Keratinocyte Overexpression from PMID: 30772301	SOX2
TCF7L1	SOX2 regulated genes in Keratinocyte Overexpression from PMID: 30772301	SOX2
CBX1	SOX2 regulated genes in Keratinocyte Overexpression from PMID: 30772301	SOX2
MDGA1	SOX2 regulated genes in Keratinocyte Overexpression from PMID: 30772301	SOX2
CLDND1	SOX2 regulated genes in Keratinocyte Overexpression from PMID: 30772301	SOX2
CENPH	SOX2 regulated genes in Keratinocyte Overexpression from PMID: 30772301	SOX2
PLEKHF2	SOX2 regulated genes in Keratinocyte Overexpression from PMID: 30772301	SOX2
DNAJB13	SOX2 regulated genes in Keratinocyte Overexpression from PMID: 30772301	SOX2
LUC7L3	SOX2 regulated genes in Keratinocyte Overexpression from PMID: 30772301	SOX2
ARHGEF1	SOX2 regulated genes in Keratinocyte Overexpression from PMID: 30772301	SOX2
PLOD2	SOX2 regulated genes in Keratinocyte Overexpression from PMID: 30772301	SOX2
NPRL2	SOX2 regulated genes in Keratinocyte Overexpression from PMID: 30772301	SOX2
METTL2B	SOX2 regulated genes in Keratinocyte Overexpression from PMID: 30772301	SOX2
TUBE1	SOX2 regulated genes in Keratinocyte Overexpression from PMID: 30772301	SOX2

C1QTNF12	SOX2 regulated genes in Keratinocyte Overexpression from PMID: 30772301	SOX2
BORCS8	SOX2 regulated genes in Keratinocyte Overexpression from PMID: 30772301	SOX2
SLCO4C1	SOX2 regulated genes in Keratinocyte Overexpression from PMID: 30772301	SOX2
LIG4	SOX2 regulated genes in Keratinocyte Overexpression from PMID: 30772301	SOX2
MEPCE	SOX2 regulated genes in Keratinocyte Overexpression from PMID: 30772301	SOX2
CYFIP2	SOX2 regulated genes in Keratinocyte Overexpression from PMID: 30772301	SOX2
CD151	SOX2 regulated genes in Keratinocyte Overexpression from PMID: 30772301	SOX2
LYRM4	SOX2 regulated genes in Keratinocyte Overexpression from PMID: 30772301	SOX2
TP53I11	SOX2 regulated genes in Keratinocyte Overexpression from PMID: 30772301	SOX2
CTBP1	SOX2 regulated genes in Keratinocyte Overexpression from PMID: 30772301	SOX2
ARL6	SOX2 regulated genes in Keratinocyte Overexpression from PMID: 30772301	SOX2
LRWD1	SOX2 regulated genes in Keratinocyte Overexpression from PMID: 30772301	SOX2
HDHD3	SOX2 regulated genes in Keratinocyte Overexpression from PMID: 30772301	SOX2
ZC3HAV1L	SOX2 regulated genes in Keratinocyte Overexpression from PMID: 30772301	SOX2
STAM	SOX2 regulated genes in Keratinocyte Overexpression from PMID: 30772301	SOX2
URI1	SOX2 regulated genes in Keratinocyte Overexpression from PMID: 30772301	SOX2
MRPL2	SOX2 regulated genes in Keratinocyte Overexpression from PMID: 30772301	SOX2
GABARAPL2	SOX2 regulated genes in Keratinocyte Overexpression from PMID: 30772301	SOX2
RARG	SOX2 regulated genes in Keratinocyte Overexpression from PMID: 30772301	SOX2
NXF1	SOX2 regulated genes in Keratinocyte Overexpression from PMID: 30772301	SOX2
VWA7	SOX2 regulated genes in Keratinocyte Overexpression from PMID: 30772301	SOX2
BBS12	SOX2 regulated genes in Keratinocyte Overexpression from PMID: 30772301	SOX2
SESN2	SOX2 regulated genes in Keratinocyte Overexpression from PMID: 30772301	SOX2
PYURF	SOX2 regulated genes in Keratinocyte Overexpression from PMID: 30772301	SOX2
FAM166A	SOX2 regulated genes in Keratinocyte Overexpression from PMID: 30772301	SOX2
GLB1L2	SOX2 regulated genes in Keratinocyte Overexpression from PMID: 30772301	SOX2
PSMC2	SOX2 regulated genes in Keratinocyte Overexpression from PMID: 30772301	SOX2
NSMCE4A	SOX2 regulated genes in Keratinocyte Overexpression from PMID: 30772301	SOX2
CLUAP1	SOX2 regulated genes in Keratinocyte Overexpression from PMID: 30772301	SOX2
JMJD7	SOX2 regulated genes in Keratinocyte Overexpression from PMID: 30772301	SOX2
CDC42BPG	SOX2 regulated genes in Keratinocyte Overexpression from PMID: 30772301	SOX2
MEA1	SOX2 regulated genes in Keratinocyte Overexpression from PMID: 30772301	SOX2
CDK17	SOX2 regulated genes in Keratinocyte Overexpression from PMID: 30772301	SOX2
ATAD5	SOX2 regulated genes in Keratinocyte Overexpression from PMID: 30772301	SOX2
DDX19B	SOX2 regulated genes in Keratinocyte Overexpression from PMID: 30772301	SOX2
CCDC189	SOX2 regulated genes in Keratinocyte Overexpression from PMID: 30772301	SOX2
ADAMTS13	SOX2 regulated genes in Keratinocyte Overexpression from PMID: 30772301	SOX2
EMC4	SOX2 regulated genes in Keratinocyte Overexpression from PMID: 30772301	SOX2
ZNF597	SOX2 regulated genes in Keratinocyte Overexpression from PMID: 30772301	SOX2
CEP131	SOX2 regulated genes in Keratinocyte Overexpression from PMID: 30772301	SOX2
KLHL2	SOX2 regulated genes in Keratinocyte Overexpression from PMID: 30772301	SOX2
FRA10AC1	SOX2 regulated genes in Keratinocyte Overexpression from PMID: 30772301	SOX2
RPS6KA3	SOX2 regulated genes in Keratinocyte Overexpression from PMID: 30772301	SOX2
PSMA3	SOX2 regulated genes in Keratinocyte Overexpression from PMID: 30772301	SOX2
CDC26	SOX2 regulated genes in Keratinocyte Overexpression from PMID: 30772301	SOX2
SPSB3	SOX2 regulated genes in Keratinocyte Overexpression from PMID: 30772301	SOX2
GPATCH1	SOX2 regulated genes in Keratinocyte Overexpression from PMID: 30772301	SOX2
TPO	SOX2 regulated genes in Keratinocyte Overexpression from PMID: 30772301	SOX2
RUFY3	SOX2 regulated genes in Keratinocyte Overexpression from PMID: 30772301	SOX2
PRPF4	SOX2 regulated genes in Keratinocyte Overexpression from PMID: 30772301	SOX2
STRN	SOX2 regulated genes in Keratinocyte Overexpression from PMID: 30772301	SOX2
LMNTD2	SOX2 regulated genes in Keratinocyte Overexpression from PMID: 30772301	SOX2
NCAPG2	SOX2 regulated genes in Keratinocyte Overexpression from PMID: 30772301	SOX2
CEP170B	SOX2 regulated genes in Keratinocyte Overexpression from PMID: 30772301	SOX2
XPO5	SOX2 regulated genes in Keratinocyte Overexpression from PMID: 30772301	SOX2
BOK	SOX2 regulated genes in Keratinocyte Overexpression from PMID: 30772301	SOX2
UTP20	SOX2 regulated genes in Keratinocyte Overexpression from PMID: 30772301	SOX2
CRYBB3	SOX2 regulated genes in Keratinocyte Overexpression from PMID: 30772301	SOX2
ARF5	SOX2 regulated genes in Keratinocyte Overexpression from PMID: 30772301	SOX2

CTSF	SOX2 regulated genes in Keratinocyte Overexpression from PMID: 30772301	SOX2
AVEN	SOX2 regulated genes in Keratinocyte Overexpression from PMID: 30772301	SOX2
SFTPD	SOX2 regulated genes in Keratinocyte Overexpression from PMID: 30772301	SOX2
GNMT	SOX2 regulated genes in Keratinocyte Overexpression from PMID: 30772301	SOX2
ZMYND8	SOX2 regulated genes in Keratinocyte Overexpression from PMID: 30772301	SOX2
TRIM32	SOX2 regulated genes in Keratinocyte Overexpression from PMID: 30772301	SOX2
CENPK	SOX2 regulated genes in Keratinocyte Overexpression from PMID: 30772301	SOX2
ZSCAN22	SOX2 regulated genes in Keratinocyte Overexpression from PMID: 30772301	SOX2
C3orf18	SOX2 regulated genes in Keratinocyte Overexpression from PMID: 30772301	SOX2
AGO2	SOX2 regulated genes in Keratinocyte Overexpression from PMID: 30772301	SOX2
NPHP4	SOX2 regulated genes in Keratinocyte Overexpression from PMID: 30772301	SOX2
IRX6	SOX2 regulated genes in Keratinocyte Overexpression from PMID: 30772301	SOX2
MRPS10	SOX2 regulated genes in Keratinocyte Overexpression from PMID: 30772301	SOX2
OIP5	SOX2 regulated genes in Keratinocyte Overexpression from PMID: 30772301	SOX2
CEP55	SOX2 regulated genes in Keratinocyte Overexpression from PMID: 30772301	SOX2
GAPVD1	SOX2 regulated genes in Keratinocyte Overexpression from PMID: 30772301	SOX2
GPC1	SOX2 regulated genes in Keratinocyte Overexpression from PMID: 30772301	SOX2
SSC4D	SOX2 regulated genes in Keratinocyte Overexpression from PMID: 30772301	SOX2
UBE2N	SOX2 regulated genes in Keratinocyte Overexpression from PMID: 30772301	SOX2
UBN2	SOX2 regulated genes in Keratinocyte Overexpression from PMID: 30772301	SOX2
CDC40	SOX2 regulated genes in Keratinocyte Overexpression from PMID: 30772301	SOX2
YPEL5	SOX2 regulated genes in Keratinocyte Overexpression from PMID: 30772301	SOX2
NEK4	SOX2 regulated genes in Keratinocyte Overexpression from PMID: 30772301	SOX2
TIMM9	SOX2 regulated genes in Keratinocyte Overexpression from PMID: 30772301	SOX2
TRIM9	SOX2 regulated genes in Keratinocyte Overexpression from PMID: 30772301	SOX2
DDX31	SOX2 regulated genes in Keratinocyte Overexpression from PMID: 30772301	SOX2
MYH9	SOX2 regulated genes in Keratinocyte Overexpression from PMID: 30772301	SOX2
TTPA	SOX2 regulated genes in Keratinocyte Overexpression from PMID: 30772301	SOX2
PARG	SOX2 regulated genes in Keratinocyte Overexpression from PMID: 30772301	SOX2
OTOA	SOX2 regulated genes in Keratinocyte Overexpression from PMID: 30772301	SOX2
RGS9	SOX2 regulated genes in Keratinocyte Overexpression from PMID: 30772301	SOX2
MYRFL	SOX2 regulated genes in Keratinocyte Overexpression from PMID: 30772301	SOX2
SALL2	SOX2 regulated genes in Keratinocyte Overexpression from PMID: 30772301	SOX2
TTLL7	SOX2 regulated genes in Keratinocyte Overexpression from PMID: 30772301	SOX2
POU5F2	SOX2 regulated genes in Keratinocyte Overexpression from PMID: 30772301	SOX2
NDUFAF4	SOX2 regulated genes in Keratinocyte Overexpression from PMID: 30772301	SOX2
IGF1R	SOX2 regulated genes in Keratinocyte Overexpression from PMID: 30772301	SOX2
VASN	SOX2 regulated genes in Keratinocyte Overexpression from PMID: 30772301	SOX2
CASKIN1	SOX2 regulated genes in Keratinocyte Overexpression from PMID: 30772301	SOX2
HERPUD2	SOX2 regulated genes in Keratinocyte Overexpression from PMID: 30772301	SOX2
OXCT2	SOX2 regulated genes in Keratinocyte Overexpression from PMID: 30772301	SOX2
SLC4A10	SOX2 regulated genes in Keratinocyte Overexpression from PMID: 30772301	SOX2
APPL2	SOX2 regulated genes in Keratinocyte Overexpression from PMID: 30772301	SOX2
OMD	SOX2 regulated genes in Keratinocyte Overexpression from PMID: 30772301	SOX2
WNK3	SOX2 regulated genes in Keratinocyte Overexpression from PMID: 30772301	SOX2
SNX18	SOX2 regulated genes in Keratinocyte Overexpression from PMID: 30772301	SOX2
DPM2	SOX2 regulated genes in Keratinocyte Overexpression from PMID: 30772301	SOX2
KIAA1191	SOX2 regulated genes in Keratinocyte Overexpression from PMID: 30772301	SOX2
RFC2	SOX2 regulated genes in Keratinocyte Overexpression from PMID: 30772301	SOX2
BTG1	SOX2 regulated genes in Keratinocyte Overexpression from PMID: 30772301	SOX2
ATP11A	SOX2 regulated genes in Keratinocyte Overexpression from PMID: 30772301	SOX2
MPLKIP	SOX2 regulated genes in Keratinocyte Overexpression from PMID: 30772301	SOX2
SPNS1	SOX2 regulated genes in Keratinocyte Overexpression from PMID: 30772301	SOX2
SAP18	SOX2 regulated genes in Keratinocyte Overexpression from PMID: 30772301	SOX2
JRK	SOX2 regulated genes in Keratinocyte Overexpression from PMID: 30772301	SOX2
CAPN13	SOX2 regulated genes in Keratinocyte Overexpression from PMID: 30772301	SOX2
EIF4G2	SOX2 regulated genes in Keratinocyte Overexpression from PMID: 30772301	SOX2
RNFT1	SOX2 regulated genes in Keratinocyte Overexpression from PMID: 30772301	SOX2
COQ7	SOX2 regulated genes in Keratinocyte Overexpression from PMID: 30772301	SOX2

DNAAF2	SOX2 regulated genes in Keratinocyte Overexpression from PMID: 30772301	SOX2
BRIX1	SOX2 regulated genes in Keratinocyte Overexpression from PMID: 30772301	SOX2
PAF1	SOX2 regulated genes in Keratinocyte Overexpression from PMID: 30772301	SOX2
BFSP2	SOX2 regulated genes in Keratinocyte Overexpression from PMID: 30772301	SOX2
PPP1R36	SOX2 regulated genes in Keratinocyte Overexpression from PMID: 30772301	SOX2
ARMC1	SOX2 regulated genes in Keratinocyte Overexpression from PMID: 30772301	SOX2
C11orf52	SOX2 regulated genes in Keratinocyte Overexpression from PMID: 30772301	SOX2
CEMIP	SOX2 regulated genes in Keratinocyte Overexpression from PMID: 30772301	SOX2
CPEB4	SOX2 regulated genes in Keratinocyte Overexpression from PMID: 30772301	SOX2
ZNF593	SOX2 regulated genes in Keratinocyte Overexpression from PMID: 30772301	SOX2
PRSS8	SOX2 regulated genes in Keratinocyte Overexpression from PMID: 30772301	SOX2
HDAC11	SOX2 regulated genes in Keratinocyte Overexpression from PMID: 30772301	SOX2
PHF14	SOX2 regulated genes in Keratinocyte Overexpression from PMID: 30772301	SOX2
CETN2	SOX2 regulated genes in Keratinocyte Overexpression from PMID: 30772301	SOX2
TBRG1	SOX2 regulated genes in Keratinocyte Overexpression from PMID: 30772301	SOX2
ERCC3	SOX2 regulated genes in Keratinocyte Overexpression from PMID: 30772301	SOX2
CDK5	SOX2 regulated genes in Keratinocyte Overexpression from PMID: 30772301	SOX2
EYA3	SOX2 regulated genes in Keratinocyte Overexpression from PMID: 30772301	SOX2
CAMTA1	SOX2 regulated genes in Keratinocyte Overexpression from PMID: 30772301	SOX2
POP7	SOX2 regulated genes in Keratinocyte Overexpression from PMID: 30772301	SOX2
HNRNPLL	SOX2 regulated genes in Keratinocyte Overexpression from PMID: 30772301	SOX2
ZFP92	SOX2 regulated genes in Keratinocyte Overexpression from PMID: 30772301	SOX2
TMEM63B	SOX2 regulated genes in Keratinocyte Overexpression from PMID: 30772301	SOX2
ZNF473	SOX2 regulated genes in Keratinocyte Overexpression from PMID: 30772301	SOX2
TNFAIP8	SOX2 regulated genes in Keratinocyte Overexpression from PMID: 30772301	SOX2
NLN	SOX2 regulated genes in Keratinocyte Overexpression from PMID: 30772301	SOX2
CNGA1	SOX2 regulated genes in Keratinocyte Overexpression from PMID: 30772301	SOX2
LDB1	SOX2 regulated genes in Keratinocyte Overexpression from PMID: 30772301	SOX2
UBAP1	SOX2 regulated genes in Keratinocyte Overexpression from PMID: 30772301	SOX2
TFAP4	SOX2 regulated genes in Keratinocyte Overexpression from PMID: 30772301	SOX2
YTHDC1	SOX2 regulated genes in Keratinocyte Overexpression from PMID: 30772301	SOX2
SIAH2	SOX2 regulated genes in Keratinocyte Overexpression from PMID: 30772301	SOX2
PLS3	SOX2 regulated genes in Keratinocyte Overexpression from PMID: 30772301	SOX2
LZTR1	SOX2 regulated genes in Keratinocyte Overexpression from PMID: 30772301	SOX2
ZNF827	SOX2 regulated genes in Keratinocyte Overexpression from PMID: 30772301	SOX2
NAP1L1	SOX2 regulated genes in Keratinocyte Overexpression from PMID: 30772301	SOX2
CRISPLD1	SOX2 regulated genes in Keratinocyte Overexpression from PMID: 30772301	SOX2
EIF3F	SOX2 regulated genes in Keratinocyte Overexpression from PMID: 30772301	SOX2
KIF23	SOX2 regulated genes in Keratinocyte Overexpression from PMID: 30772301	SOX2
RRP36	SOX2 regulated genes in Keratinocyte Overexpression from PMID: 30772301	SOX2
TRIM31	SOX2 regulated genes in Keratinocyte Overexpression from PMID: 30772301	SOX2
TAF6L	SOX2 regulated genes in Keratinocyte Overexpression from PMID: 30772301	SOX2
ITGB6	SOX2 regulated genes in Keratinocyte Overexpression from PMID: 30772301	SOX2
ARMCX5	SOX2 regulated genes in Keratinocyte Overexpression from PMID: 30772301	SOX2
BTBD11	SOX2 regulated genes in Keratinocyte Overexpression from PMID: 30772301	SOX2
EIF1AD	SOX2 regulated genes in Keratinocyte Overexpression from PMID: 30772301	SOX2
ACRBP	SOX2 regulated genes in Keratinocyte Overexpression from PMID: 30772301	SOX2
METTL3	SOX2 regulated genes in Keratinocyte Overexpression from PMID: 30772301	SOX2
IGF2BP1	SOX2 regulated genes in Keratinocyte Overexpression from PMID: 30772301	SOX2
GPR176	SOX2 regulated genes in Keratinocyte Overexpression from PMID: 30772301	SOX2
PDZD4	SOX2 regulated genes in Keratinocyte Overexpression from PMID: 30772301	SOX2
TRMO	SOX2 regulated genes in Keratinocyte Overexpression from PMID: 30772301	SOX2
KDM4B	SOX2 regulated genes in Keratinocyte Overexpression from PMID: 30772301	SOX2
FAIM2	SOX2 regulated genes in Keratinocyte Overexpression from PMID: 30772301	SOX2
DMGDH	SOX2 regulated genes in Keratinocyte Overexpression from PMID: 30772301	SOX2
ZNF101	SOX2 regulated genes in Keratinocyte Overexpression from PMID: 30772301	SOX2
MT4	SOX2 regulated genes in Keratinocyte Overexpression from PMID: 30772301	SOX2
ATXN2	SOX2 regulated genes in Keratinocyte Overexpression from PMID: 30772301	SOX2
XRCC2	SOX2 regulated genes in Keratinocyte Overexpression from PMID: 30772301	SOX2

CNOT11	SOX2 regulated genes in Keratinocyte Overexpression from PMID: 30772301	SOX2
RAVER2	SOX2 regulated genes in Keratinocyte Overexpression from PMID: 30772301	SOX2
UBE2V2	SOX2 regulated genes in Keratinocyte Overexpression from PMID: 30772301	SOX2
MPHOSPH6	SOX2 regulated genes in Keratinocyte Overexpression from PMID: 30772301	SOX2
THRAP3	SOX2 regulated genes in Keratinocyte Overexpression from PMID: 30772301	SOX2
FAM47E	SOX2 regulated genes in Keratinocyte Overexpression from PMID: 30772301	SOX2
STK39	SOX2 regulated genes in Keratinocyte Overexpression from PMID: 30772301	SOX2
TPM4	SOX2 regulated genes in Keratinocyte Overexpression from PMID: 30772301	SOX2
TDRD12	SOX2 regulated genes in Keratinocyte Overexpression from PMID: 30772301	SOX2
VAMP3	SOX2 regulated genes in Keratinocyte Overexpression from PMID: 30772301	SOX2
ALDH18A1	SOX2 regulated genes in Keratinocyte Overexpression from PMID: 30772301	SOX2
ZDHHC24	SOX2 regulated genes in Keratinocyte Overexpression from PMID: 30772301	SOX2
CDCA7	SOX2 regulated genes in Keratinocyte Overexpression from PMID: 30772301	SOX2
PIGT	SOX2 regulated genes in Keratinocyte Overexpression from PMID: 30772301	SOX2
EMD	SOX2 regulated genes in Keratinocyte Overexpression from PMID: 30772301	SOX2
WDR18	SOX2 regulated genes in Keratinocyte Overexpression from PMID: 30772301	SOX2
SLC52A2	SOX2 regulated genes in Keratinocyte Overexpression from PMID: 30772301	SOX2
DENR	SOX2 regulated genes in Keratinocyte Overexpression from PMID: 30772301	SOX2
GLT1D1	SOX2 regulated genes in Keratinocyte Overexpression from PMID: 30772301	SOX2
LCAT	SOX2 regulated genes in Keratinocyte Overexpression from PMID: 30772301	SOX2
CRYBG3	SOX2 regulated genes in Keratinocyte Overexpression from PMID: 30772301	SOX2
TMEM102	SOX2 regulated genes in Keratinocyte Overexpression from PMID: 30772301	SOX2
C2orf49	SOX2 regulated genes in Keratinocyte Overexpression from PMID: 30772301	SOX2
CASC3	SOX2 regulated genes in Keratinocyte Overexpression from PMID: 30772301	SOX2
SLC35B1	SOX2 regulated genes in Keratinocyte Overexpression from PMID: 30772301	SOX2
MAPK13	SOX2 regulated genes in Keratinocyte Overexpression from PMID: 30772301	SOX2
JOSD1	SOX2 regulated genes in Keratinocyte Overexpression from PMID: 30772301	SOX2
ASIC1	SOX2 regulated genes in Keratinocyte Overexpression from PMID: 30772301	SOX2
PCSK1	SOX2 regulated genes in Keratinocyte Overexpression from PMID: 30772301	SOX2
CEACAM21	SOX2 regulated genes in Keratinocyte Overexpression from PMID: 30772301	SOX2
UTP6	SOX2 regulated genes in Keratinocyte Overexpression from PMID: 30772301	SOX2
TOMM70	SOX2 regulated genes in Keratinocyte Overexpression from PMID: 30772301	SOX2
LCOR	SOX2 regulated genes in Keratinocyte Overexpression from PMID: 30772301	SOX2
KIF24	SOX2 regulated genes in Keratinocyte Overexpression from PMID: 30772301	SOX2
CCDC61	SOX2 regulated genes in Keratinocyte Overexpression from PMID: 30772301	SOX2
RRM1	SOX2 regulated genes in Keratinocyte Overexpression from PMID: 30772301	SOX2
TRPV5	SOX2 regulated genes in Keratinocyte Overexpression from PMID: 30772301	SOX2
NEMP1	SOX2 regulated genes in Keratinocyte Overexpression from PMID: 30772301	SOX2
SBF1	SOX2 regulated genes in Keratinocyte Overexpression from PMID: 30772301	SOX2
CAPN11	SOX2 regulated genes in Keratinocyte Overexpression from PMID: 30772301	SOX2
WNT6	SOX2 regulated genes in Keratinocyte Overexpression from PMID: 30772301	SOX2
SPOCK3	SOX2 regulated genes in Keratinocyte Overexpression from PMID: 30772301	SOX2
CTRL	SOX2 regulated genes in Keratinocyte Overexpression from PMID: 30772301	SOX2
SMO	SOX2 regulated genes in Keratinocyte Overexpression from PMID: 30772301	SOX2
KNOP1	SOX2 regulated genes in Keratinocyte Overexpression from PMID: 30772301	SOX2
DNAJC21	SOX2 regulated genes in Keratinocyte Overexpression from PMID: 30772301	SOX2
CARM1	SOX2 regulated genes in Keratinocyte Overexpression from PMID: 30772301	SOX2
NUSAP1	SOX2 regulated genes in Keratinocyte Overexpression from PMID: 30772301	SOX2
PSMD12	SOX2 regulated genes in Keratinocyte Overexpression from PMID: 30772301	SOX2
NFKBIB	SOX2 regulated genes in Keratinocyte Overexpression from PMID: 30772301	SOX2
PPP4R4	SOX2 regulated genes in Keratinocyte Overexpression from PMID: 30772301	SOX2
ESRP2	SOX2 regulated genes in Keratinocyte Overexpression from PMID: 30772301	SOX2
PEX7	SOX2 regulated genes in Keratinocyte Overexpression from PMID: 30772301	SOX2
IYD	SOX2 regulated genes in Keratinocyte Overexpression from PMID: 30772301	SOX2
BCDIN3D	SOX2 regulated genes in Keratinocyte Overexpression from PMID: 30772301	SOX2
NCKAP1	SOX2 regulated genes in Keratinocyte Overexpression from PMID: 30772301	SOX2
ILVBL	SOX2 regulated genes in Keratinocyte Overexpression from PMID: 30772301	SOX2
NEK6	SOX2 regulated genes in Keratinocyte Overexpression from PMID: 30772301	SOX2
PRKCH	SOX2 regulated genes in Keratinocyte Overexpression from PMID: 30772301	SOX2

POLD2	SOX2 regulated genes in Keratinocyte Overexpression from PMID: 30772301	SOX2
ZNF740	SOX2 regulated genes in Keratinocyte Overexpression from PMID: 30772301	SOX2
NIPBL	SOX2 regulated genes in Keratinocyte Overexpression from PMID: 30772301	SOX2
CPA4	SOX2 regulated genes in Keratinocyte Overexpression from PMID: 30772301	SOX2
SNAI2	SOX2 regulated genes in Keratinocyte Overexpression from PMID: 30772301	SOX2
SMYD4	SOX2 regulated genes in Keratinocyte Overexpression from PMID: 30772301	SOX2
CDPF1	SOX2 regulated genes in Keratinocyte Overexpression from PMID: 30772301	SOX2
DNAJB6	SOX2 regulated genes in Keratinocyte Overexpression from PMID: 30772301	SOX2
DCAF10	SOX2 regulated genes in Keratinocyte Overexpression from PMID: 30772301	SOX2
CLSPN	SOX2 regulated genes in Keratinocyte Overexpression from PMID: 30772301	SOX2
CFDP1	SOX2 regulated genes in Keratinocyte Overexpression from PMID: 30772301	SOX2
EYA1	SOX2 regulated genes in Keratinocyte Overexpression from PMID: 30772301	SOX2
UBN1	SOX2 regulated genes in Keratinocyte Overexpression from PMID: 30772301	SOX2
ATXN7L3B	SOX2 regulated genes in Keratinocyte Overexpression from PMID: 30772301	SOX2
GALNT6	SOX2 regulated genes in Keratinocyte Overexpression from PMID: 30772301	SOX2
TTC23	SOX2 regulated genes in Keratinocyte Overexpression from PMID: 30772301	SOX2
TCP11L2	SOX2 regulated genes in Keratinocyte Overexpression from PMID: 30772301	SOX2
CHCHD4	SOX2 regulated genes in Keratinocyte Overexpression from PMID: 30772301	SOX2
CEP89	SOX2 regulated genes in Keratinocyte Overexpression from PMID: 30772301	SOX2
FBXW8	SOX2 regulated genes in Keratinocyte Overexpression from PMID: 30772301	SOX2
DMRTA2	SOX2 regulated genes in Keratinocyte Overexpression from PMID: 30772301	SOX2
KAT7	SOX2 regulated genes in Keratinocyte Overexpression from PMID: 30772301	SOX2
NCAPH2	SOX2 regulated genes in Keratinocyte Overexpression from PMID: 30772301	SOX2
PPP2R1A	SOX2 regulated genes in Keratinocyte Overexpression from PMID: 30772301	SOX2
SAFB2	SOX2 regulated genes in Keratinocyte Overexpression from PMID: 30772301	SOX2
IGF2R	SOX2 regulated genes in Keratinocyte Overexpression from PMID: 30772301	SOX2
AOC2	SOX2 regulated genes in Keratinocyte Overexpression from PMID: 30772301	SOX2
EXOSC9	SOX2 regulated genes in Keratinocyte Overexpression from PMID: 30772301	SOX2
IGSF11	SOX2 regulated genes in Keratinocyte Overexpression from PMID: 30772301	SOX2
ALDH4A1	SOX2 regulated genes in Keratinocyte Overexpression from PMID: 30772301	SOX2
SEMA3F	SOX2 regulated genes in Keratinocyte Overexpression from PMID: 30772301	SOX2
ZBTB1	SOX2 regulated genes in Keratinocyte Overexpression from PMID: 30772301	SOX2
MRPL54	SOX2 regulated genes in Keratinocyte Overexpression from PMID: 30772301	SOX2
RPL22	SOX2 regulated genes in Keratinocyte Overexpression from PMID: 30772301	SOX2
KRT80	SOX2 regulated genes in Keratinocyte Overexpression from PMID: 30772301	SOX2
SOX5	SOX2 regulated genes in Keratinocyte Overexpression from PMID: 30772301	SOX2
GTF3C4	SOX2 regulated genes in Keratinocyte Overexpression from PMID: 30772301	SOX2
TRPM4	SOX2 regulated genes in Keratinocyte Overexpression from PMID: 30772301	SOX2
PAXIP1	SOX2 regulated genes in Keratinocyte Overexpression from PMID: 30772301	SOX2
AARSD1	SOX2 regulated genes in Keratinocyte Overexpression from PMID: 30772301	SOX2
ZRANB3	SOX2 regulated genes in Keratinocyte Overexpression from PMID: 30772301	SOX2
GPR173	SOX2 regulated genes in Keratinocyte Overexpression from PMID: 30772301	SOX2
HSPA1L	SOX2 regulated genes in Keratinocyte Overexpression from PMID: 30772301	SOX2
TMEM170A	SOX2 regulated genes in Keratinocyte Overexpression from PMID: 30772301	SOX2
NOC3L	SOX2 regulated genes in Keratinocyte Overexpression from PMID: 30772301	SOX2
MTRF1L	SOX2 regulated genes in Keratinocyte Overexpression from PMID: 30772301	SOX2
ATF1	SOX2 regulated genes in Keratinocyte Overexpression from PMID: 30772301	SOX2
MED18	SOX2 regulated genes in Keratinocyte Overexpression from PMID: 30772301	SOX2
GPC3	SOX2 regulated genes in Keratinocyte Overexpression from PMID: 30772301	SOX2
SYBU	SOX2 regulated genes in Keratinocyte Overexpression from PMID: 30772301	SOX2
BBS1	SOX2 regulated genes in Keratinocyte Overexpression from PMID: 30772301	SOX2
SUPT4H1	SOX2 regulated genes in Keratinocyte Overexpression from PMID: 30772301	SOX2
TSSK6	SOX2 regulated genes in Keratinocyte Overexpression from PMID: 30772301	SOX2
UTP14C	SOX2 regulated genes in Keratinocyte Overexpression from PMID: 30772301	SOX2
PLCH2	SOX2 regulated genes in Keratinocyte Overexpression from PMID: 30772301	SOX2
UBR5	SOX2 regulated genes in Keratinocyte Overexpression from PMID: 30772301	SOX2
EDC4	SOX2 regulated genes in Keratinocyte Overexpression from PMID: 30772301	SOX2
LSM8	SOX2 regulated genes in Keratinocyte Overexpression from PMID: 30772301	SOX2
LCN12	SOX2 regulated genes in Keratinocyte Overexpression from PMID: 30772301	SOX2

MBNL3	SOX2 regulated genes in Keratinocyte Overexpression from PMID: 30772301	SOX2
NAE1	SOX2 regulated genes in Keratinocyte Overexpression from PMID: 30772301	SOX2
SOX10	SOX2 regulated genes in Keratinocyte Overexpression from PMID: 30772301	SOX2
STRN4	SOX2 regulated genes in Keratinocyte Overexpression from PMID: 30772301	SOX2
SLC38A3	SOX2 regulated genes in Keratinocyte Overexpression from PMID: 30772301	SOX2
RIOX2	SOX2 regulated genes in Keratinocyte Overexpression from PMID: 30772301	SOX2
HELQ	SOX2 regulated genes in Keratinocyte Overexpression from PMID: 30772301	SOX2
CD200R1	SOX2 regulated genes in Keratinocyte Overexpression from PMID: 30772301	SOX2
EXOSC8	SOX2 regulated genes in Keratinocyte Overexpression from PMID: 30772301	SOX2
FANCE	SOX2 regulated genes in Keratinocyte Overexpression from PMID: 30772301	SOX2
NEK5	SOX2 regulated genes in Keratinocyte Overexpression from PMID: 30772301	SOX2
YWHAQ	SOX2 regulated genes in Keratinocyte Overexpression from PMID: 30772301	SOX2
TENM4	SOX2 regulated genes in Keratinocyte Overexpression from PMID: 30772301	SOX2
AMMECR1	SOX2 regulated genes in Keratinocyte Overexpression from PMID: 30772301	SOX2
STRBP	SOX2 regulated genes in Keratinocyte Overexpression from PMID: 30772301	SOX2
METAP1	SOX2 regulated genes in Keratinocyte Overexpression from PMID: 30772301	SOX2
DNAJC2	SOX2 regulated genes in Keratinocyte Overexpression from PMID: 30772301	SOX2
HAUS3	SOX2 regulated genes in Keratinocyte Overexpression from PMID: 30772301	SOX2
PAQR5	SOX2 regulated genes in Keratinocyte Overexpression from PMID: 30772301	SOX2
CECR2	SOX2 regulated genes in Keratinocyte Overexpression from PMID: 30772301	SOX2
SPDL1	SOX2 regulated genes in Keratinocyte Overexpression from PMID: 30772301	SOX2
ABLIM2	SOX2 regulated genes in Keratinocyte Overexpression from PMID: 30772301	SOX2
PIN1	SOX2 regulated genes in Keratinocyte Overexpression from PMID: 30772301	SOX2
PHF6	SOX2 regulated genes in Keratinocyte Overexpression from PMID: 30772301	SOX2
CTDSPL	SOX2 regulated genes in Keratinocyte Overexpression from PMID: 30772301	SOX2
SERHL2	SOX2 regulated genes in Keratinocyte Overexpression from PMID: 30772301	SOX2
NOL11	SOX2 regulated genes in Keratinocyte Overexpression from PMID: 30772301	SOX2
PIGU	SOX2 regulated genes in Keratinocyte Overexpression from PMID: 30772301	SOX2
STX1A	SOX2 regulated genes in Keratinocyte Overexpression from PMID: 30772301	SOX2
SDK2	SOX2 regulated genes in Keratinocyte Overexpression from PMID: 30772301	SOX2
SRD5A2	SOX2 regulated genes in Keratinocyte Overexpression from PMID: 30772301	SOX2
BOLA2B	SOX2 regulated genes in Keratinocyte Overexpression from PMID: 30772301	SOX2
COPS3	SOX2 regulated genes in Keratinocyte Overexpression from PMID: 30772301	SOX2
C9orf152	SOX2 regulated genes in Keratinocyte Overexpression from PMID: 30772301	SOX2
UNC93A	SOX2 regulated genes in Keratinocyte Overexpression from PMID: 30772301	SOX2
MROH7	SOX2 regulated genes in Keratinocyte Overexpression from PMID: 30772301	SOX2
SCX	SOX2 regulated genes in Keratinocyte Overexpression from PMID: 30772301	SOX2
ZC3H4	SOX2 regulated genes in Keratinocyte Overexpression from PMID: 30772301	SOX2
RPL37A	SOX2 regulated genes in Keratinocyte Overexpression from PMID: 30772301	SOX2
ADCK2	SOX2 regulated genes in Keratinocyte Overexpression from PMID: 30772301	SOX2
SIPA1L3	SOX2 regulated genes in Keratinocyte Overexpression from PMID: 30772301	SOX2
TMC7	SOX2 regulated genes in Keratinocyte Overexpression from PMID: 30772301	SOX2
TMEM171	SOX2 regulated genes in Keratinocyte Overexpression from PMID: 30772301	SOX2
OCM	SOX2 regulated genes in Keratinocyte Overexpression from PMID: 30772301	SOX2
DYRK2	SOX2 regulated genes in Keratinocyte Overexpression from PMID: 30772301	SOX2
PLAG1	SOX2 regulated genes in Keratinocyte Overexpression from PMID: 30772301	SOX2
DYNLL1	SOX2 regulated genes in Keratinocyte Overexpression from PMID: 30772301	SOX2
PTGES2	SOX2 regulated genes in Keratinocyte Overexpression from PMID: 30772301	SOX2
PLA2G4C	SOX2 regulated genes in Keratinocyte Overexpression from PMID: 30772301	SOX2
PXYLP1	SOX2 regulated genes in Keratinocyte Overexpression from PMID: 30772301	SOX2
RIMBP3C	SOX2 regulated genes in Keratinocyte Overexpression from PMID: 30772301	SOX2
NDFIP2	SOX2 regulated genes in Keratinocyte Overexpression from PMID: 30772301	SOX2
RSPO3	SOX2 regulated genes in Keratinocyte Overexpression from PMID: 30772301	SOX2
TMEM185A	SOX2 regulated genes in Keratinocyte Overexpression from PMID: 30772301	SOX2
LSM3	SOX2 regulated genes in Keratinocyte Overexpression from PMID: 30772301	SOX2
ATP6V1H	SOX2 regulated genes in Keratinocyte Overexpression from PMID: 30772301	SOX2
EPCAM	SOX2 regulated genes in Keratinocyte Overexpression from PMID: 30772301	SOX2
EEF1AKMT1	SOX2 regulated genes in Keratinocyte Overexpression from PMID: 30772301	SOX2
LATS1	SOX2 regulated genes in Keratinocyte Overexpression from PMID: 30772301	SOX2

PPP4R3A	SOX2 regulated genes in Keratinocyte Overexpression from PMID: 30772301	SOX2
APBA3	SOX2 regulated genes in Keratinocyte Overexpression from PMID: 30772301	SOX2
SERTAD3	SOX2 regulated genes in Keratinocyte Overexpression from PMID: 30772301	SOX2
PPP1CB	SOX2 regulated genes in Keratinocyte Overexpression from PMID: 30772301	SOX2
MCM4	SOX2 regulated genes in Keratinocyte Overexpression from PMID: 30772301	SOX2
ODF2L	SOX2 regulated genes in Keratinocyte Overexpression from PMID: 30772301	SOX2
CASP8AP2	SOX2 regulated genes in Keratinocyte Overexpression from PMID: 30772301	SOX2
SKIL	SOX2 regulated genes in Keratinocyte Overexpression from PMID: 30772301	SOX2
FUBP3	SOX2 regulated genes in Keratinocyte Overexpression from PMID: 30772301	SOX2
MTBP	SOX2 regulated genes in Keratinocyte Overexpression from PMID: 30772301	SOX2
PSCA	SOX2 regulated genes in Keratinocyte Overexpression from PMID: 30772301	SOX2
VPS33B	SOX2 regulated genes in Keratinocyte Overexpression from PMID: 30772301	SOX2
RAB3B	SOX2 regulated genes in Keratinocyte Overexpression from PMID: 30772301	SOX2
CLDN34	SOX2 regulated genes in Keratinocyte Overexpression from PMID: 30772301	SOX2
LRRC45	SOX2 regulated genes in Keratinocyte Overexpression from PMID: 30772301	SOX2
HMMR	SOX2 regulated genes in Keratinocyte Overexpression from PMID: 30772301	SOX2
RAC3	SOX2 regulated genes in Keratinocyte Overexpression from PMID: 30772301	SOX2
NECAB1	SOX2 regulated genes in Keratinocyte Overexpression from PMID: 30772301	SOX2
ZNF248	SOX2 regulated genes in Keratinocyte Overexpression from PMID: 30772301	SOX2
PSMA1	SOX2 regulated genes in Keratinocyte Overexpression from PMID: 30772301	SOX2
CKAP4	SOX2 regulated genes in Keratinocyte Overexpression from PMID: 30772301	SOX2
LGALSL	SOX2 regulated genes in Keratinocyte Overexpression from PMID: 30772301	SOX2
ARMC5	SOX2 regulated genes in Keratinocyte Overexpression from PMID: 30772301	SOX2
PSMD11	SOX2 regulated genes in Keratinocyte Overexpression from PMID: 30772301	SOX2
FBXL6	SOX2 regulated genes in Keratinocyte Overexpression from PMID: 30772301	SOX2
CYC1	SOX2 regulated genes in Keratinocyte Overexpression from PMID: 30772301	SOX2
RPL31	SOX2 regulated genes in Keratinocyte Overexpression from PMID: 30772301	SOX2
SMDT1	SOX2 regulated genes in Keratinocyte Overexpression from PMID: 30772301	SOX2
TALDO1	SOX2 regulated genes in Keratinocyte Overexpression from PMID: 30772301	SOX2
ZFP37	SOX2 regulated genes in Keratinocyte Overexpression from PMID: 30772301	SOX2
SNF8	SOX2 regulated genes in Keratinocyte Overexpression from PMID: 30772301	SOX2
CFAP54	SOX2 regulated genes in Keratinocyte Overexpression from PMID: 30772301	SOX2
ACAT1	SOX2 regulated genes in Keratinocyte Overexpression from PMID: 30772301	SOX2
STARD4	SOX2 regulated genes in Keratinocyte Overexpression from PMID: 30772301	SOX2
SAAL1	SOX2 regulated genes in Keratinocyte Overexpression from PMID: 30772301	SOX2
PRPF18	SOX2 regulated genes in Keratinocyte Overexpression from PMID: 30772301	SOX2
BMPR1B	SOX2 regulated genes in Keratinocyte Overexpression from PMID: 30772301	SOX2
ANKRD6	SOX2 regulated genes in Keratinocyte Overexpression from PMID: 30772301	SOX2
LHX2	SOX2 regulated genes in Keratinocyte Overexpression from PMID: 30772301	SOX2
NELFA	SOX2 regulated genes in Keratinocyte Overexpression from PMID: 30772301	SOX2
TMEM129	SOX2 regulated genes in Keratinocyte Overexpression from PMID: 30772301	SOX2
C4orf46	SOX2 regulated genes in Keratinocyte Overexpression from PMID: 30772301	SOX2
MCC	SOX2 regulated genes in Keratinocyte Overexpression from PMID: 30772301	SOX2
TBCC	SOX2 regulated genes in Keratinocyte Overexpression from PMID: 30772301	SOX2
RAB37	SOX2 regulated genes in Keratinocyte Overexpression from PMID: 30772301	SOX2
EGR4	SOX2 regulated genes in Keratinocyte Overexpression from PMID: 30772301	SOX2
CHSY3	SOX2 regulated genes in Keratinocyte Overexpression from PMID: 30772301	SOX2
ZNF2	SOX2 regulated genes in Keratinocyte Overexpression from PMID: 30772301	SOX2
FZD6	SOX2 regulated genes in Keratinocyte Overexpression from PMID: 30772301	SOX2
NELFE	SOX2 regulated genes in Keratinocyte Overexpression from PMID: 30772301	SOX2
TRMT10A	SOX2 regulated genes in Keratinocyte Overexpression from PMID: 30772301	SOX2
DSCC1	SOX2 regulated genes in Keratinocyte Overexpression from PMID: 30772301	SOX2
DNAH17	SOX2 regulated genes in Keratinocyte Overexpression from PMID: 30772301	SOX2
CRB3	SOX2 regulated genes in Keratinocyte Overexpression from PMID: 30772301	SOX2
CREBBP	SOX2 regulated genes in Keratinocyte Overexpression from PMID: 30772301	SOX2
PMPCA	SOX2 regulated genes in Keratinocyte Overexpression from PMID: 30772301	SOX2
C11orf94	SOX2 regulated genes in Keratinocyte Overexpression from PMID: 30772301	SOX2
BARD1	SOX2 regulated genes in Keratinocyte Overexpression from PMID: 30772301	SOX2
DNAJA2	SOX2 regulated genes in Keratinocyte Overexpression from PMID: 30772301	SOX2

TPBG	SOX2 regulated genes in Keratinocyte Overexpression from PMID: 30772301	SOX2
RANBP17	SOX2 regulated genes in Keratinocyte Overexpression from PMID: 30772301	SOX2
KIAA1958	SOX2 regulated genes in Keratinocyte Overexpression from PMID: 30772301	SOX2
MSH3	SOX2 regulated genes in Keratinocyte Overexpression from PMID: 30772301	SOX2
PDCD5	SOX2 regulated genes in Keratinocyte Overexpression from PMID: 30772301	SOX2
ATN1	SOX2 regulated genes in Keratinocyte Overexpression from PMID: 30772301	SOX2
PIP4K2C	SOX2 regulated genes in Keratinocyte Overexpression from PMID: 30772301	SOX2
DEF6	SOX2 regulated genes in Keratinocyte Overexpression from PMID: 30772301	SOX2
TMTC3	SOX2 regulated genes in Keratinocyte Overexpression from PMID: 30772301	SOX2
PHLDB2	SOX2 regulated genes in Keratinocyte Overexpression from PMID: 30772301	SOX2
LGALS12	SOX2 regulated genes in Keratinocyte Overexpression from PMID: 30772301	SOX2
ZC3HC1	SOX2 regulated genes in Keratinocyte Overexpression from PMID: 30772301	SOX2
DCAF13	SOX2 regulated genes in Keratinocyte Overexpression from PMID: 30772301	SOX2
CHIC1	SOX2 regulated genes in Keratinocyte Overexpression from PMID: 30772301	SOX2
TP53BP1	SOX2 regulated genes in Keratinocyte Overexpression from PMID: 30772301	SOX2
SBK1	SOX2 regulated genes in Keratinocyte Overexpression from PMID: 30772301	SOX2
GPLD1	SOX2 regulated genes in Keratinocyte Overexpression from PMID: 30772301	SOX2
AHI1	SOX2 regulated genes in Keratinocyte Overexpression from PMID: 30772301	SOX2
NHS	SOX2 regulated genes in Keratinocyte Overexpression from PMID: 30772301	SOX2
IFT80	SOX2 regulated genes in Keratinocyte Overexpression from PMID: 30772301	SOX2
PAM16	SOX2 regulated genes in Keratinocyte Overexpression from PMID: 30772301	SOX2
ZCCHC10	SOX2 regulated genes in Keratinocyte Overexpression from PMID: 30772301	SOX2
SLF1	SOX2 regulated genes in Keratinocyte Overexpression from PMID: 30772301	SOX2
MCMBP	SOX2 regulated genes in Keratinocyte Overexpression from PMID: 30772301	SOX2
OSGIN2	SOX2 regulated genes in Keratinocyte Overexpression from PMID: 30772301	SOX2
XYLB	SOX2 regulated genes in Keratinocyte Overexpression from PMID: 30772301	SOX2
BTBD10	SOX2 regulated genes in Keratinocyte Overexpression from PMID: 30772301	SOX2
CLIC4	SOX2 regulated genes in Keratinocyte Overexpression from PMID: 30772301	SOX2
GEMIN6	SOX2 regulated genes in Keratinocyte Overexpression from PMID: 30772301	SOX2
MRPL46	SOX2 regulated genes in Keratinocyte Overexpression from PMID: 30772301	SOX2
ATP7A	SOX2 regulated genes in Keratinocyte Overexpression from PMID: 30772301	SOX2
PPM1B	SOX2 regulated genes in Keratinocyte Overexpression from PMID: 30772301	SOX2
GADD45A	SOX2 regulated genes in Keratinocyte Overexpression from PMID: 30772301	SOX2
WTAP	SOX2 regulated genes in Keratinocyte Overexpression from PMID: 30772301	SOX2
GRIK4	SOX2 regulated genes in Keratinocyte Overexpression from PMID: 30772301	SOX2
MED26	SOX2 regulated genes in Keratinocyte Overexpression from PMID: 30772301	SOX2
PREX2	SOX2 regulated genes in Keratinocyte Overexpression from PMID: 30772301	SOX2
GGACT	SOX2 regulated genes in Keratinocyte Overexpression from PMID: 30772301	SOX2
ARNTL	SOX2 regulated genes in Keratinocyte Overexpression from PMID: 30772301	SOX2
SLC30A4	SOX2 regulated genes in Keratinocyte Overexpression from PMID: 30772301	SOX2
SUGT1	SOX2 regulated genes in Keratinocyte Overexpression from PMID: 30772301	SOX2
PPWD1	SOX2 regulated genes in Keratinocyte Overexpression from PMID: 30772301	SOX2
RASSF7	SOX2 regulated genes in Keratinocyte Overexpression from PMID: 30772301	SOX2
TUBB4B	SOX2 regulated genes in Keratinocyte Overexpression from PMID: 30772301	SOX2
MND1	SOX2 regulated genes in Keratinocyte Overexpression from PMID: 30772301	SOX2
ZFYVE28	SOX2 regulated genes in Keratinocyte Overexpression from PMID: 30772301	SOX2
TTC26	SOX2 regulated genes in Keratinocyte Overexpression from PMID: 30772301	SOX2
GARNL3	SOX2 regulated genes in Keratinocyte Overexpression from PMID: 30772301	SOX2
PRPF6	SOX2 regulated genes in Keratinocyte Overexpression from PMID: 30772301	SOX2
KDM4A	SOX2 regulated genes in Keratinocyte Overexpression from PMID: 30772301	SOX2
SPATA5	SOX2 regulated genes in Keratinocyte Overexpression from PMID: 30772301	SOX2
SYT1	SOX2 regulated genes in Keratinocyte Overexpression from PMID: 30772301	SOX2
DUS4L	SOX2 regulated genes in Keratinocyte Overexpression from PMID: 30772301	SOX2
MRPS9	SOX2 regulated genes in Keratinocyte Overexpression from PMID: 30772301	SOX2
ERMARD	SOX2 regulated genes in Keratinocyte Overexpression from PMID: 30772301	SOX2
NSMAF	SOX2 regulated genes in Keratinocyte Overexpression from PMID: 30772301	SOX2
CACNG4	SOX2 regulated genes in Keratinocyte Overexpression from PMID: 30772301	SOX2
CEP126	SOX2 regulated genes in Keratinocyte Overexpression from PMID: 30772301	SOX2
ZFHX2	SOX2 regulated genes in Keratinocyte Overexpression from PMID: 30772301	SOX2

TMEM132A	SOX2 regulated genes in Keratinocyte Overexpression from PMID: 30772301	SOX2
AOC1	SOX2 regulated genes in Keratinocyte Overexpression from PMID: 30772301	SOX2
NDUFAF8	SOX2 regulated genes in Keratinocyte Overexpression from PMID: 30772301	SOX2
HYAL2	SOX2 regulated genes in Keratinocyte Overexpression from PMID: 30772301	SOX2
MSH5	SOX2 regulated genes in Keratinocyte Overexpression from PMID: 30772301	SOX2
FFAR3	SOX2 regulated genes in Keratinocyte Overexpression from PMID: 30772301	SOX2
C16orf74	SOX2 regulated genes in Keratinocyte Overexpression from PMID: 30772301	SOX2
METTL26	SOX2 regulated genes in Keratinocyte Overexpression from PMID: 30772301	SOX2
SC5D	SOX2 regulated genes in Keratinocyte Overexpression from PMID: 30772301	SOX2
MIP	SOX2 regulated genes in Keratinocyte Overexpression from PMID: 30772301	SOX2
SEC16A	SOX2 regulated genes in Keratinocyte Overexpression from PMID: 30772301	SOX2
ZBTB8B	SOX2 regulated genes in Keratinocyte Overexpression from PMID: 30772301	SOX2
DNAJC17	SOX2 regulated genes in Keratinocyte Overexpression from PMID: 30772301	SOX2
MACC1	SOX2 regulated genes in Keratinocyte Overexpression from PMID: 30772301	SOX2
FBXO34	SOX2 regulated genes in Keratinocyte Overexpression from PMID: 30772301	SOX2
ARID4A	SOX2 regulated genes in Keratinocyte Overexpression from PMID: 30772301	SOX2
FBXO42	SOX2 regulated genes in Keratinocyte Overexpression from PMID: 30772301	SOX2
ESRP1	SOX2 regulated genes in Keratinocyte Overexpression from PMID: 30772301	SOX2
FMNL2	SOX2 regulated genes in Keratinocyte Overexpression from PMID: 30772301	SOX2
RPL30	SOX2 regulated genes in Keratinocyte Overexpression from PMID: 30772301	SOX2
ZNF213	SOX2 regulated genes in Keratinocyte Overexpression from PMID: 30772301	SOX2
SUPV3L1	SOX2 regulated genes in Keratinocyte Overexpression from PMID: 30772301	SOX2
ZNF334	SOX2 regulated genes in Keratinocyte Overexpression from PMID: 30772301	SOX2
LPAR2	SOX2 regulated genes in Keratinocyte Overexpression from PMID: 30772301	SOX2
ZCCHC7	SOX2 regulated genes in Keratinocyte Overexpression from PMID: 30772301	SOX2
SYP	SOX2 regulated genes in Keratinocyte Overexpression from PMID: 30772301	SOX2
CTNNA1	SOX2 regulated genes in Keratinocyte Overexpression from PMID: 30772301	SOX2
ANKRD27	SOX2 regulated genes in Keratinocyte Overexpression from PMID: 30772301	SOX2
LCMT1	SOX2 regulated genes in Keratinocyte Overexpression from PMID: 30772301	SOX2
SNTB1	SOX2 regulated genes in Keratinocyte Overexpression from PMID: 30772301	SOX2
KLHL32	SOX2 regulated genes in Keratinocyte Overexpression from PMID: 30772301	SOX2
STX5	SOX2 regulated genes in Keratinocyte Overexpression from PMID: 30772301	SOX2
IGHMBP2	SOX2 regulated genes in Keratinocyte Overexpression from PMID: 30772301	SOX2
PLD6	SOX2 regulated genes in Keratinocyte Overexpression from PMID: 30772301	SOX2
GNA11	SOX2 regulated genes in Keratinocyte Overexpression from PMID: 30772301	SOX2
TTL	SOX2 regulated genes in Keratinocyte Overexpression from PMID: 30772301	SOX2
SLC25A46	SOX2 regulated genes in Keratinocyte Overexpression from PMID: 30772301	SOX2
GLI1	SOX2 regulated genes in Keratinocyte Overexpression from PMID: 30772301	SOX2
RBM10	SOX2 regulated genes in Keratinocyte Overexpression from PMID: 30772301	SOX2
THAP11	SOX2 regulated genes in Keratinocyte Overexpression from PMID: 30772301	SOX2
DCTN3	SOX2 regulated genes in Keratinocyte Overexpression from PMID: 30772301	SOX2
CALD1	SOX2 regulated genes in Keratinocyte Overexpression from PMID: 30772301	SOX2
FAM193B	SOX2 regulated genes in Keratinocyte Overexpression from PMID: 30772301	SOX2
AIG1	SOX2 regulated genes in Keratinocyte Overexpression from PMID: 30772301	SOX2
SLC9A2	SOX2 regulated genes in Keratinocyte Overexpression from PMID: 30772301	SOX2
TMEM268	SOX2 regulated genes in Keratinocyte Overexpression from PMID: 30772301	SOX2
HAGH	SOX2 regulated genes in Keratinocyte Overexpression from PMID: 30772301	SOX2
NUP210	SOX2 regulated genes in Keratinocyte Overexpression from PMID: 30772301	SOX2
LRP8	SOX2 regulated genes in Keratinocyte Overexpression from PMID: 30772301	SOX2
RBM42	SOX2 regulated genes in Keratinocyte Overexpression from PMID: 30772301	SOX2
PTGER4	SOX2 regulated genes in Keratinocyte Overexpression from PMID: 30772301	SOX2
MSH2	SOX2 regulated genes in Keratinocyte Overexpression from PMID: 30772301	SOX2
YIF1A	SOX2 regulated genes in Keratinocyte Overexpression from PMID: 30772301	SOX2
TUFM	SOX2 regulated genes in Keratinocyte Overexpression from PMID: 30772301	SOX2
MICAL1	SOX2 regulated genes in Keratinocyte Overexpression from PMID: 30772301	SOX2
TCF19	SOX2 regulated genes in Keratinocyte Overexpression from PMID: 30772301	SOX2
LZTS2	SOX2 regulated genes in Keratinocyte Overexpression from PMID: 30772301	SOX2
HSPB1	SOX2 regulated genes in Keratinocyte Overexpression from PMID: 30772301	SOX2
CPSF2	SOX2 regulated genes in Keratinocyte Overexpression from PMID: 30772301	SOX2

SHCBP1	SOX2 regulated genes in Keratinocyte Overexpression from PMID: 30772301	SOX2
DNLZ	SOX2 regulated genes in Keratinocyte Overexpression from PMID: 30772301	SOX2
NSD2	SOX2 regulated genes in Keratinocyte Overexpression from PMID: 30772301	SOX2
ZFP1	SOX2 regulated genes in Keratinocyte Overexpression from PMID: 30772301	SOX2
GTPBP10	SOX2 regulated genes in Keratinocyte Overexpression from PMID: 30772301	SOX2
DEPDC1B	SOX2 regulated genes in Keratinocyte Overexpression from PMID: 30772301	SOX2
SERPINB9	SOX2 regulated genes in Keratinocyte Overexpression from PMID: 30772301	SOX2
KIAA1549	SOX2 regulated genes in Keratinocyte Overexpression from PMID: 30772301	SOX2
MBLAC1	SOX2 regulated genes in Keratinocyte Overexpression from PMID: 30772301	SOX2
B3GALT6	SOX2 regulated genes in Keratinocyte Overexpression from PMID: 30772301	SOX2
IFRD1	SOX2 regulated genes in Keratinocyte Overexpression from PMID: 30772301	SOX2
KIT	SOX2 regulated genes in Keratinocyte Overexpression from PMID: 30772301	SOX2
DIS3L	SOX2 regulated genes in Keratinocyte Overexpression from PMID: 30772301	SOX2
S100PBP	SOX2 regulated genes in Keratinocyte Overexpression from PMID: 30772301	SOX2
TDG	SOX2 regulated genes in Keratinocyte Overexpression from PMID: 30772301	SOX2
POU2F1	SOX2 regulated genes in Keratinocyte Overexpression from PMID: 30772301	SOX2
ABLIM3	SOX2 regulated genes in Keratinocyte Overexpression from PMID: 30772301	SOX2
KCNK7	SOX2 regulated genes in Keratinocyte Overexpression from PMID: 30772301	SOX2
KCNC3	SOX2 regulated genes in Keratinocyte Overexpression from PMID: 30772301	SOX2
TXLNA	SOX2 regulated genes in Keratinocyte Overexpression from PMID: 30772301	SOX2
DDX5	SOX2 regulated genes in Keratinocyte Overexpression from PMID: 30772301	SOX2
RITA1	SOX2 regulated genes in Keratinocyte Overexpression from PMID: 30772301	SOX2
RUNDC3A	SOX2 regulated genes in Keratinocyte Overexpression from PMID: 30772301	SOX2
NLRX1	SOX2 regulated genes in Keratinocyte Overexpression from PMID: 30772301	SOX2
DDX59	SOX2 regulated genes in Keratinocyte Overexpression from PMID: 30772301	SOX2
ARHGAP29	SOX2 regulated genes in Keratinocyte Overexpression from PMID: 30772301	SOX2
FBXO25	SOX2 regulated genes in Keratinocyte Overexpression from PMID: 30772301	SOX2
SMPD4	SOX2 regulated genes in Keratinocyte Overexpression from PMID: 30772301	SOX2
ODF2	SOX2 regulated genes in Keratinocyte Overexpression from PMID: 30772301	SOX2
TXNL4B	SOX2 regulated genes in Keratinocyte Overexpression from PMID: 30772301	SOX2
TIGD5	SOX2 regulated genes in Keratinocyte Overexpression from PMID: 30772301	SOX2
PLA2G4B	SOX2 regulated genes in Keratinocyte Overexpression from PMID: 30772301	SOX2
HAUS8	SOX2 regulated genes in Keratinocyte Overexpression from PMID: 30772301	SOX2
C6orf163	SOX2 regulated genes in Keratinocyte Overexpression from PMID: 30772301	SOX2
FBXO38	SOX2 regulated genes in Keratinocyte Overexpression from PMID: 30772301	SOX2
UBE3B	SOX2 regulated genes in Keratinocyte Overexpression from PMID: 30772301	SOX2
CACNA1H	SOX2 regulated genes in Keratinocyte Overexpression from PMID: 30772301	SOX2
RFX1	SOX2 regulated genes in Keratinocyte Overexpression from PMID: 30772301	SOX2
TTC13	SOX2 regulated genes in Keratinocyte Overexpression from PMID: 30772301	SOX2
NGEF	SOX2 regulated genes in Keratinocyte Overexpression from PMID: 30772301	SOX2
LRRC59	SOX2 regulated genes in Keratinocyte Overexpression from PMID: 30772301	SOX2
PDCD10	SOX2 regulated genes in Keratinocyte Overexpression from PMID: 30772301	SOX2
TOP2B	SOX2 regulated genes in Keratinocyte Overexpression from PMID: 30772301	SOX2
OVCA2	SOX2 regulated genes in Keratinocyte Overexpression from PMID: 30772301	SOX2
COX7A2	SOX2 regulated genes in Keratinocyte Overexpression from PMID: 30772301	SOX2
SPSB2	SOX2 regulated genes in Keratinocyte Overexpression from PMID: 30772301	SOX2
SNAPC2	SOX2 regulated genes in Keratinocyte Overexpression from PMID: 30772301	SOX2
LETMD1	SOX2 regulated genes in Keratinocyte Overexpression from PMID: 30772301	SOX2
TRMT13	SOX2 regulated genes in Keratinocyte Overexpression from PMID: 30772301	SOX2
TRNAU1AP	SOX2 regulated genes in Keratinocyte Overexpression from PMID: 30772301	SOX2
HEXIM2	SOX2 regulated genes in Keratinocyte Overexpression from PMID: 30772301	SOX2
C3orf62	SOX2 regulated genes in Keratinocyte Overexpression from PMID: 30772301	SOX2
KIFC1	SOX2 regulated genes in Keratinocyte Overexpression from PMID: 30772301	SOX2
PFDN6	SOX2 regulated genes in Keratinocyte Overexpression from PMID: 30772301	SOX2
PPM1D	SOX2 regulated genes in Keratinocyte Overexpression from PMID: 30772301	SOX2
CD320	SOX2 regulated genes in Keratinocyte Overexpression from PMID: 30772301	SOX2
NUDC	SOX2 regulated genes in Keratinocyte Overexpression from PMID: 30772301	SOX2
TRADD	SOX2 regulated genes in Keratinocyte Overexpression from PMID: 30772301	SOX2
SSB	SOX2 regulated genes in Keratinocyte Overexpression from PMID: 30772301	SOX2

MUL1	SOX2 regulated genes in Keratinocyte Overexpression from PMID: 30772301	SOX2
C16orf91	SOX2 regulated genes in Keratinocyte Overexpression from PMID: 30772301	SOX2
CHMP2B	SOX2 regulated genes in Keratinocyte Overexpression from PMID: 30772301	SOX2
FAM169A	SOX2 regulated genes in Keratinocyte Overexpression from PMID: 30772301	SOX2
TFPT	SOX2 regulated genes in Keratinocyte Overexpression from PMID: 30772301	SOX2
ZFAND2B	SOX2 regulated genes in Keratinocyte Overexpression from PMID: 30772301	SOX2
PHLDA3	SOX2 regulated genes in Keratinocyte Overexpression from PMID: 30772301	SOX2
ARAP2	SOX2 regulated genes in Keratinocyte Overexpression from PMID: 30772301	SOX2
ATP13A2	SOX2 regulated genes in Keratinocyte Overexpression from PMID: 30772301	SOX2
RAD9A	SOX2 regulated genes in Keratinocyte Overexpression from PMID: 30772301	SOX2
PLEKHG6	SOX2 regulated genes in Keratinocyte Overexpression from PMID: 30772301	SOX2
AMT	SOX2 regulated genes in Keratinocyte Overexpression from PMID: 30772301	SOX2
ERI2	SOX2 regulated genes in Keratinocyte Overexpression from PMID: 30772301	SOX2
CPNE4	SOX2 regulated genes in Keratinocyte Overexpression from PMID: 30772301	SOX2
KLK7	SOX2 regulated genes in Keratinocyte Overexpression from PMID: 30772301	SOX2
PYM1	SOX2 regulated genes in Keratinocyte Overexpression from PMID: 30772301	SOX2
ATG9B	SOX2 regulated genes in Keratinocyte Overexpression from PMID: 30772301	SOX2
ECE1	SOX2 regulated genes in Keratinocyte Overexpression from PMID: 30772301	SOX2
ZNF768	SOX2 regulated genes in Keratinocyte Overexpression from PMID: 30772301	SOX2
NPLOC4	SOX2 regulated genes in Keratinocyte Overexpression from PMID: 30772301	SOX2
RAD54L	SOX2 regulated genes in Keratinocyte Overexpression from PMID: 30772301	SOX2
SFSWAP	SOX2 regulated genes in Keratinocyte Overexpression from PMID: 30772301	SOX2
ARF6	SOX2 regulated genes in Keratinocyte Overexpression from PMID: 30772301	SOX2
GSK3B	SOX2 regulated genes in Keratinocyte Overexpression from PMID: 30772301	SOX2
SMYD3	SOX2 regulated genes in Keratinocyte Overexpression from PMID: 30772301	SOX2
VPS53	SOX2 regulated genes in Keratinocyte Overexpression from PMID: 30772301	SOX2
FBXO41	SOX2 regulated genes in Keratinocyte Overexpression from PMID: 30772301	SOX2
VPS26B	SOX2 regulated genes in Keratinocyte Overexpression from PMID: 30772301	SOX2
CNOT3	SOX2 regulated genes in Keratinocyte Overexpression from PMID: 30772301	SOX2
SEC61A1	SOX2 regulated genes in Keratinocyte Overexpression from PMID: 30772301	SOX2
FBXW11	SOX2 regulated genes in Keratinocyte Overexpression from PMID: 30772301	SOX2
DUSP10	SOX2 regulated genes in Keratinocyte Overexpression from PMID: 30772301	SOX2
PPP1R13L	SOX2 regulated genes in Keratinocyte Overexpression from PMID: 30772301	SOX2
ZFYVE27	SOX2 regulated genes in Keratinocyte Overexpression from PMID: 30772301	SOX2
BDP1	SOX2 regulated genes in Keratinocyte Overexpression from PMID: 30772301	SOX2
PDE6G	SOX2 regulated genes in Keratinocyte Overexpression from PMID: 30772301	SOX2
KLF16	SOX2 regulated genes in Keratinocyte Overexpression from PMID: 30772301	SOX2
GPR19	SOX2 regulated genes in Keratinocyte Overexpression from PMID: 30772301	SOX2
RBM33	SOX2 regulated genes in Keratinocyte Overexpression from PMID: 30772301	SOX2
WDR55	SOX2 regulated genes in Keratinocyte Overexpression from PMID: 30772301	SOX2
ZBTB7A	SOX2 regulated genes in Keratinocyte Overexpression from PMID: 30772301	SOX2
RBBP4	SOX2 regulated genes in Keratinocyte Overexpression from PMID: 30772301	SOX2
SNRPE	SOX2 regulated genes in Keratinocyte Overexpression from PMID: 30772301	SOX2
MRPL24	SOX2 regulated genes in Keratinocyte Overexpression from PMID: 30772301	SOX2
PPHLN1	SOX2 regulated genes in Keratinocyte Overexpression from PMID: 30772301	SOX2
PCDH1	SOX2 regulated genes in Keratinocyte Overexpression from PMID: 30772301	SOX2
RUNDC1	SOX2 regulated genes in Keratinocyte Overexpression from PMID: 30772301	SOX2
YBX2	SOX2 regulated genes in Keratinocyte Overexpression from PMID: 30772301	SOX2
VPS9D1	SOX2 regulated genes in Keratinocyte Overexpression from PMID: 30772301	SOX2
RTCA	SOX2 regulated genes in Keratinocyte Overexpression from PMID: 30772301	SOX2
HOXA5	SOX2 regulated genes in Keratinocyte Overexpression from PMID: 30772301	SOX2
RGL2	SOX2 regulated genes in Keratinocyte Overexpression from PMID: 30772301	SOX2
VIPAS39	SOX2 regulated genes in Keratinocyte Overexpression from PMID: 30772301	SOX2
SWT1	SOX2 regulated genes in Keratinocyte Overexpression from PMID: 30772301	SOX2
ATP5F1B	SOX2 regulated genes in Keratinocyte Overexpression from PMID: 30772301	SOX2
DNMT1	SOX2 regulated genes in Keratinocyte Overexpression from PMID: 30772301	SOX2
SENP5	SOX2 regulated genes in Keratinocyte Overexpression from PMID: 30772301	SOX2
CLDN1	SOX2 regulated genes in Keratinocyte Overexpression from PMID: 30772301	SOX2
ABCB11	SOX2 regulated genes in Keratinocyte Overexpression from PMID: 30772301	SOX2

SCRN2	SOX2 regulated genes in Keratinocyte Overexpression from PMID: 30772301	SOX2
DLEC1	SOX2 regulated genes in Keratinocyte Overexpression from PMID: 30772301	SOX2
TEPSIN	SOX2 regulated genes in Keratinocyte Overexpression from PMID: 30772301	SOX2
UBE2F	SOX2 regulated genes in Keratinocyte Overexpression from PMID: 30772301	SOX2
ACVR2A	SOX2 regulated genes in Keratinocyte Overexpression from PMID: 30772301	SOX2
BSDC1	SOX2 regulated genes in Keratinocyte Overexpression from PMID: 30772301	SOX2
KIF3C	SOX2 regulated genes in Keratinocyte Overexpression from PMID: 30772301	SOX2
ZNF280D	SOX2 regulated genes in Keratinocyte Overexpression from PMID: 30772301	SOX2
VPS25	SOX2 regulated genes in Keratinocyte Overexpression from PMID: 30772301	SOX2
BLM	SOX2 regulated genes in Keratinocyte Overexpression from PMID: 30772301	SOX2
NMD3	SOX2 regulated genes in Keratinocyte Overexpression from PMID: 30772301	SOX2
NTF4	SOX2 regulated genes in Keratinocyte Overexpression from PMID: 30772301	SOX2
ZNF653	SOX2 regulated genes in Keratinocyte Overexpression from PMID: 30772301	SOX2
PRMT6	SOX2 regulated genes in Keratinocyte Overexpression from PMID: 30772301	SOX2
ATOX1	SOX2 regulated genes in Keratinocyte Overexpression from PMID: 30772301	SOX2
ZNF691	SOX2 regulated genes in Keratinocyte Overexpression from PMID: 30772301	SOX2
JUN	SOX2 regulated genes in Keratinocyte Overexpression from PMID: 30772301	SOX2
MTMR4	SOX2 regulated genes in Keratinocyte Overexpression from PMID: 30772301	SOX2
MRPL4	SOX2 regulated genes in Keratinocyte Overexpression from PMID: 30772301	SOX2
USP5	SOX2 regulated genes in Keratinocyte Overexpression from PMID: 30772301	SOX2
MCOLN3	SOX2 regulated genes in Keratinocyte Overexpression from PMID: 30772301	SOX2
HNRNPR	SOX2 regulated genes in Keratinocyte Overexpression from PMID: 30772301	SOX2
DAPK3	SOX2 regulated genes in Keratinocyte Overexpression from PMID: 30772301	SOX2
CTXN1	SOX2 regulated genes in Keratinocyte Overexpression from PMID: 30772301	SOX2
CACNB3	SOX2 regulated genes in Keratinocyte Overexpression from PMID: 30772301	SOX2
SLC45A3	SOX2 regulated genes in Keratinocyte Overexpression from PMID: 30772301	SOX2
TRIM45	SOX2 regulated genes in Keratinocyte Overexpression from PMID: 30772301	SOX2
PHYHIP	SOX2 regulated genes in Keratinocyte Overexpression from PMID: 30772301	SOX2
TAF6	SOX2 regulated genes in Keratinocyte Overexpression from PMID: 30772301	SOX2
ZNF329	SOX2 regulated genes in Keratinocyte Overexpression from PMID: 30772301	SOX2
ERF	SOX2 regulated genes in Keratinocyte Overexpression from PMID: 30772301	SOX2
NUP42	SOX2 regulated genes in Keratinocyte Overexpression from PMID: 30772301	SOX2
PIGF	SOX2 regulated genes in Keratinocyte Overexpression from PMID: 30772301	SOX2
RIMS1	SOX2 regulated genes in Keratinocyte Overexpression from PMID: 30772301	SOX2
FAM20A	SOX2 regulated genes in Keratinocyte Overexpression from PMID: 30772301	SOX2
EIF5B	SOX2 regulated genes in Keratinocyte Overexpression from PMID: 30772301	SOX2
MXD1	SOX2 regulated genes in Keratinocyte Overexpression from PMID: 30772301	SOX2
RGMA	SOX2 regulated genes in Keratinocyte Overexpression from PMID: 30772301	SOX2
RYBP	SOX2 regulated genes in Keratinocyte Overexpression from PMID: 30772301	SOX2
NME7	SOX2 regulated genes in Keratinocyte Overexpression from PMID: 30772301	SOX2
NUP43	SOX2 regulated genes in Keratinocyte Overexpression from PMID: 30772301	SOX2
MLX	SOX2 regulated genes in Keratinocyte Overexpression from PMID: 30772301	SOX2
CCS	SOX2 regulated genes in Keratinocyte Overexpression from PMID: 30772301	SOX2
PSMB3	SOX2 regulated genes in Keratinocyte Overexpression from PMID: 30772301	SOX2
CBARP	SOX2 regulated genes in Keratinocyte Overexpression from PMID: 30772301	SOX2
USP2	SOX2 regulated genes in Keratinocyte Overexpression from PMID: 30772301	SOX2
ITGA10	SOX2 regulated genes in Keratinocyte Overexpression from PMID: 30772301	SOX2
VPS72	SOX2 regulated genes in Keratinocyte Overexpression from PMID: 30772301	SOX2
PGBD1	SOX2 regulated genes in Keratinocyte Overexpression from PMID: 30772301	SOX2
SDE2	SOX2 regulated genes in Keratinocyte Overexpression from PMID: 30772301	SOX2
TSR3	SOX2 regulated genes in Keratinocyte Overexpression from PMID: 30772301	SOX2
KHDRBS1	SOX2 regulated genes in Keratinocyte Overexpression from PMID: 30772301	SOX2
COX10	SOX2 regulated genes in Keratinocyte Overexpression from PMID: 30772301	SOX2
EMP2	SOX2 regulated genes in Keratinocyte Overexpression from PMID: 30772301	SOX2
SIRT7	SOX2 regulated genes in Keratinocyte Overexpression from PMID: 30772301	SOX2
IFNLR1	SOX2 regulated genes in Keratinocyte Overexpression from PMID: 30772301	SOX2
LARP4B	SOX2 regulated genes in Keratinocyte Overexpression from PMID: 30772301	SOX2
ANKRD11	SOX2 regulated genes in Keratinocyte Overexpression from PMID: 30772301	SOX2
FOXP1	SOX2 regulated genes in Keratinocyte Overexpression from PMID: 30772301	SOX2

WNT5B	SOX2 regulated genes in Keratinocyte Overexpression from PMID: 30772301	SOX2
FAM214A	SOX2 regulated genes in Keratinocyte Overexpression from PMID: 30772301	SOX2
STK19	SOX2 regulated genes in Keratinocyte Overexpression from PMID: 30772301	SOX2
EGR1	SOX2 regulated genes in Keratinocyte Overexpression from PMID: 30772301	SOX2
ZC3H8	SOX2 regulated genes in Keratinocyte Overexpression from PMID: 30772301	SOX2
GEMIN4	SOX2 regulated genes in Keratinocyte Overexpression from PMID: 30772301	SOX2
FABP12	SOX2 regulated genes in Keratinocyte Overexpression from PMID: 30772301	SOX2
MYH15	SOX2 regulated genes in Keratinocyte Overexpression from PMID: 30772301	SOX2
SERPINI1	SOX2 regulated genes in Keratinocyte Overexpression from PMID: 30772301	SOX2
DCTN1	SOX2 regulated genes in Keratinocyte Overexpression from PMID: 30772301	SOX2
LLPH	SOX2 regulated genes in Keratinocyte Overexpression from PMID: 30772301	SOX2
SNAPC3	SOX2 regulated genes in Keratinocyte Overexpression from PMID: 30772301	SOX2
POU4F3	SOX2 regulated genes in Keratinocyte Overexpression from PMID: 30772301	SOX2
GRSF1	SOX2 regulated genes in Keratinocyte Overexpression from PMID: 30772301	SOX2
PRKAB1	SOX2 regulated genes in Keratinocyte Overexpression from PMID: 30772301	SOX2
ACTR8	SOX2 regulated genes in Keratinocyte Overexpression from PMID: 30772301	SOX2
CAMK2N1	SOX2 regulated genes in Keratinocyte Overexpression from PMID: 30772301	SOX2
SRSF4	SOX2 regulated genes in Keratinocyte Overexpression from PMID: 30772301	SOX2
ZNF512	SOX2 regulated genes in Keratinocyte Overexpression from PMID: 30772301	SOX2
GDPD1	SOX2 regulated genes in Keratinocyte Overexpression from PMID: 30772301	SOX2
SIRT1	SOX2 regulated genes in Keratinocyte Overexpression from PMID: 30772301	SOX2
NUP133	SOX2 regulated genes in Keratinocyte Overexpression from PMID: 30772301	SOX2
MKNK2	SOX2 regulated genes in Keratinocyte Overexpression from PMID: 30772301	SOX2
WDR53	SOX2 regulated genes in Keratinocyte Overexpression from PMID: 30772301	SOX2
MAPKBP1	SOX2 regulated genes in Keratinocyte Overexpression from PMID: 30772301	SOX2
SIRT4	SOX2 regulated genes in Keratinocyte Overexpression from PMID: 30772301	SOX2
PHB	SOX2 regulated genes in Keratinocyte Overexpression from PMID: 30772301	SOX2
MACF1	SOX2 regulated genes in Keratinocyte Overexpression from PMID: 30772301	SOX2
TSG101	SOX2 regulated genes in Keratinocyte Overexpression from PMID: 30772301	SOX2
RNF208	SOX2 regulated genes in Keratinocyte Overexpression from PMID: 30772301	SOX2
ARHGAP22	SOX2 regulated genes in Keratinocyte Overexpression from PMID: 30772301	SOX2
GCDH	SOX2 regulated genes in Keratinocyte Overexpression from PMID: 30772301	SOX2
SDF2	SOX2 regulated genes in Keratinocyte Overexpression from PMID: 30772301	SOX2
TKFC	SOX2 regulated genes in Keratinocyte Overexpression from PMID: 30772301	SOX2
MZT2A	SOX2 regulated genes in Keratinocyte Overexpression from PMID: 30772301	SOX2
MZT2B	SOX2 regulated genes in Keratinocyte Overexpression from PMID: 30772301	SOX2
EPN1	SOX2 regulated genes in Keratinocyte Overexpression from PMID: 30772301	SOX2
DHODH	SOX2 regulated genes in Keratinocyte Overexpression from PMID: 30772301	SOX2
TBCA	SOX2 regulated genes in Keratinocyte Overexpression from PMID: 30772301	SOX2
MFSD2B	SOX2 regulated genes in Keratinocyte Overexpression from PMID: 30772301	SOX2
MTX1	SOX2 regulated genes in Keratinocyte Overexpression from PMID: 30772301	SOX2
DCUN1D5	SOX2 regulated genes in Keratinocyte Overexpression from PMID: 30772301	SOX2
NINJ2	SOX2 regulated genes in Keratinocyte Overexpression from PMID: 30772301	SOX2
RHOU	SOX2 regulated genes in Keratinocyte Overexpression from PMID: 30772301	SOX2
PPIP5K1	SOX2 regulated genes in Keratinocyte Overexpression from PMID: 30772301	SOX2
RPS15	SOX2 regulated genes in Keratinocyte Overexpression from PMID: 30772301	SOX2
FBXL18	SOX2 regulated genes in Keratinocyte Overexpression from PMID: 30772301	SOX2
FCAMR	SOX2 regulated genes in Keratinocyte Overexpression from PMID: 30772301	SOX2
ALKBH7	SOX2 regulated genes in Keratinocyte Overexpression from PMID: 30772301	SOX2
C1orf109	SOX2 regulated genes in Keratinocyte Overexpression from PMID: 30772301	SOX2
ZNF800	SOX2 regulated genes in Keratinocyte Overexpression from PMID: 30772301	SOX2
USH2A	SOX2 regulated genes in Keratinocyte Overexpression from PMID: 30772301	SOX2
AXDND1	SOX2 regulated genes in Keratinocyte Overexpression from PMID: 30772301	SOX2
TIMM22	SOX2 regulated genes in Keratinocyte Overexpression from PMID: 30772301	SOX2
C1orf35	SOX2 regulated genes in Keratinocyte Overexpression from PMID: 30772301	SOX2
PRKCQ	SOX2 regulated genes in Keratinocyte Overexpression from PMID: 30772301	SOX2
MIGA1	SOX2 regulated genes in Keratinocyte Overexpression from PMID: 30772301	SOX2
PDXDC1	SOX2 regulated genes in Keratinocyte Overexpression from PMID: 30772301	SOX2
PTOV1	SOX2 regulated genes in Keratinocyte Overexpression from PMID: 30772301	SOX2

NFATC2IP	SOX2 regulated genes in Keratinocyte Overexpression from PMID: 30772301	SOX2
STRIP1	SOX2 regulated genes in Keratinocyte Overexpression from PMID: 30772301	SOX2
MEX3D	SOX2 regulated genes in Keratinocyte Overexpression from PMID: 30772301	SOX2
ASB7	SOX2 regulated genes in Keratinocyte Overexpression from PMID: 30772301	SOX2
PRSS22	SOX2 regulated genes in Keratinocyte Overexpression from PMID: 30772301	SOX2
HGS	SOX2 regulated genes in Keratinocyte Overexpression from PMID: 30772301	SOX2
NKIRAS2	SOX2 regulated genes in Keratinocyte Overexpression from PMID: 30772301	SOX2
MGST3	SOX2 regulated genes in Keratinocyte Overexpression from PMID: 30772301	SOX2
CAPN10	SOX2 regulated genes in Keratinocyte Overexpression from PMID: 30772301	SOX2
EME1	SOX2 regulated genes in Keratinocyte Overexpression from PMID: 30772301	SOX2
DLG1	SOX2 regulated genes in Keratinocyte Overexpression from PMID: 30772301	SOX2
SHH	SOX2 regulated genes in Keratinocyte Overexpression from PMID: 30772301	SOX2
PRPF3	SOX2 regulated genes in Keratinocyte Overexpression from PMID: 30772301	SOX2
DUS3L	SOX2 regulated genes in Keratinocyte Overexpression from PMID: 30772301	SOX2
FAT2	SOX2 regulated genes in Keratinocyte Overexpression from PMID: 30772301	SOX2
PDHB	SOX2 regulated genes in Keratinocyte Overexpression from PMID: 30772301	SOX2
TOMM40	SOX2 regulated genes in Keratinocyte Overexpression from PMID: 30772301	SOX2
ORC1	SOX2 regulated genes in Keratinocyte Overexpression from PMID: 30772301	SOX2
SLC44A2	SOX2 regulated genes in Keratinocyte Overexpression from PMID: 30772301	SOX2
MRPS5	SOX2 regulated genes in Keratinocyte Overexpression from PMID: 30772301	SOX2
TFAP2E	SOX2 regulated genes in Keratinocyte Overexpression from PMID: 30772301	SOX2
KIF2A	SOX2 regulated genes in Keratinocyte Overexpression from PMID: 30772301	SOX2
TADA3	SOX2 regulated genes in Keratinocyte Overexpression from PMID: 30772301	SOX2
RRP8	SOX2 regulated genes in Keratinocyte Overexpression from PMID: 30772301	SOX2
PFDN2	SOX2 regulated genes in Keratinocyte Overexpression from PMID: 30772301	SOX2
ZNF513	SOX2 regulated genes in Keratinocyte Overexpression from PMID: 30772301	SOX2
B3GNTL1	SOX2 regulated genes in Keratinocyte Overexpression from PMID: 30772301	SOX2
S100A2	SOX2 regulated genes in Keratinocyte Overexpression from PMID: 30772301	SOX2
LRIG3	SOX2 regulated genes in Keratinocyte Overexpression from PMID: 30772301	SOX2
LUZP1	SOX2 regulated genes in Keratinocyte Overexpression from PMID: 30772301	SOX2
C8orf58	SOX2 regulated genes in Keratinocyte Overexpression from PMID: 30772301	SOX2
SSR2	SOX2 regulated genes in Keratinocyte Overexpression from PMID: 30772301	SOX2
CDC42BPA	SOX2 regulated genes in Keratinocyte Overexpression from PMID: 30772301	SOX2
USP40	SOX2 regulated genes in Keratinocyte Overexpression from PMID: 30772301	SOX2
SLC5A10	SOX2 regulated genes in Keratinocyte Overexpression from PMID: 30772301	SOX2
SMG6	SOX2 regulated genes in Keratinocyte Overexpression from PMID: 30772301	SOX2
EFCAB5	SOX2 regulated genes in Keratinocyte Overexpression from PMID: 30772301	SOX2
ATP1B1	SOX2 regulated genes in Keratinocyte Overexpression from PMID: 30772301	SOX2
DENND5B	SOX2 regulated genes in Keratinocyte Overexpression from PMID: 30772301	SOX2
CNOT6L	SOX2 regulated genes in Keratinocyte Overexpression from PMID: 30772301	SOX2
LIMA1	SOX2 regulated genes in Keratinocyte Overexpression from PMID: 30772301	SOX2
EFNA4	SOX2 regulated genes in Keratinocyte Overexpression from PMID: 30772301	SOX2
RAF1	SOX2 regulated genes in Keratinocyte Overexpression from PMID: 30772301	SOX2
CRIPT	SOX2 regulated genes in Keratinocyte Overexpression from PMID: 30772301	SOX2
TBCEL	SOX2 regulated genes in Keratinocyte Overexpression from PMID: 30772301	SOX2
NIPAL4	SOX2 regulated genes in Keratinocyte Overexpression from PMID: 30772301	SOX2
LRRC14	SOX2 regulated genes in Keratinocyte Overexpression from PMID: 30772301	SOX2
TMEM40	SOX2 regulated genes in Keratinocyte Overexpression from PMID: 30772301	SOX2
GRM3	SOX2 regulated genes in Keratinocyte Overexpression from PMID: 30772301	SOX2
DDX46	SOX2 regulated genes in Keratinocyte Overexpression from PMID: 30772301	SOX2
SLC16A10	SOX2 regulated genes in Keratinocyte Overexpression from PMID: 30772301	SOX2
AURKC	SOX2 regulated genes in Keratinocyte Overexpression from PMID: 30772301	SOX2
PASK	SOX2 regulated genes in Keratinocyte Overexpression from PMID: 30772301	SOX2
LARP4	SOX2 regulated genes in Keratinocyte Overexpression from PMID: 30772301	SOX2
PARD3B	SOX2 regulated genes in Keratinocyte Overexpression from PMID: 30772301	SOX2
HJURP	SOX2 regulated genes in Keratinocyte Overexpression from PMID: 30772301	SOX2
TBCB	SOX2 regulated genes in Keratinocyte Overexpression from PMID: 30772301	SOX2
PGAP1	SOX2 regulated genes in Keratinocyte Overexpression from PMID: 30772301	SOX2
PSMA5	SOX2 regulated genes in Keratinocyte Overexpression from PMID: 30772301	SOX2

SLC27A6	SOX2 regulated genes in Keratinocyte Overexpression from PMID: 30772301	SOX2
ETV3	SOX2 regulated genes in Keratinocyte Overexpression from PMID: 30772301	SOX2
RBM8A	SOX2 regulated genes in Keratinocyte Overexpression from PMID: 30772301	SOX2
MEGF11	SOX2 regulated genes in Keratinocyte Overexpression from PMID: 30772301	SOX2
IQGAP3	SOX2 regulated genes in Keratinocyte Overexpression from PMID: 30772301	SOX2
TSEN34	SOX2 regulated genes in Keratinocyte Overexpression from PMID: 30772301	SOX2
PHF13	SOX2 regulated genes in Keratinocyte Overexpression from PMID: 30772301	SOX2
TARS2	SOX2 regulated genes in Keratinocyte Overexpression from PMID: 30772301	SOX2
FYTTD1	SOX2 regulated genes in Keratinocyte Overexpression from PMID: 30772301	SOX2
ZKSCAN4	SOX2 regulated genes in Keratinocyte Overexpression from PMID: 30772301	SOX2
SNX16	SOX2 regulated genes in Keratinocyte Overexpression from PMID: 30772301	SOX2
PFN2	SOX2 regulated genes in Keratinocyte Overexpression from PMID: 30772301	SOX2
RNF32	SOX2 regulated genes in Keratinocyte Overexpression from PMID: 30772301	SOX2
DISP2	SOX2 regulated genes in Keratinocyte Overexpression from PMID: 30772301	SOX2
RIF1	SOX2 regulated genes in Keratinocyte Overexpression from PMID: 30772301	SOX2
SH2B1	SOX2 regulated genes in Keratinocyte Overexpression from PMID: 30772301	SOX2
TLK2	SOX2 regulated genes in Keratinocyte Overexpression from PMID: 30772301	SOX2
PSMB6	SOX2 regulated genes in Keratinocyte Overexpression from PMID: 30772301	SOX2
BEAN1	SOX2 regulated genes in Keratinocyte Overexpression from PMID: 30772301	SOX2
TRMT112	SOX2 regulated genes in Keratinocyte Overexpression from PMID: 30772301	SOX2
TARBP1	SOX2 regulated genes in Keratinocyte Overexpression from PMID: 30772301	SOX2
COQ10B	SOX2 regulated genes in Keratinocyte Overexpression from PMID: 30772301	SOX2
SHF	SOX2 regulated genes in Keratinocyte Overexpression from PMID: 30772301	SOX2
USF1	SOX2 regulated genes in Keratinocyte Overexpression from PMID: 30772301	SOX2
RPGRIP1L	SOX2 regulated genes in Keratinocyte Overexpression from PMID: 30772301	SOX2
AXIN2	SOX2 regulated genes in Keratinocyte Overexpression from PMID: 30772301	SOX2
LIN37	SOX2 regulated genes in Keratinocyte Overexpression from PMID: 30772301	SOX2
INAFM2	SOX2 regulated genes in Keratinocyte Overexpression from PMID: 30772301	SOX2
PSMB4	SOX2 regulated genes in Keratinocyte Overexpression from PMID: 30772301	SOX2
ABCG4	SOX2 regulated genes in Keratinocyte Overexpression from PMID: 30772301	SOX2
NUCKS1	SOX2 regulated genes in Keratinocyte Overexpression from PMID: 30772301	SOX2
MKRN2OS	SOX2 regulated genes in Keratinocyte Overexpression from PMID: 30772301	SOX2
HIP1R	SOX2 regulated genes in Keratinocyte Overexpression from PMID: 30772301	SOX2
METTL8	SOX2 regulated genes in Keratinocyte Overexpression from PMID: 30772301	SOX2
IQCC	SOX2 regulated genes in Keratinocyte Overexpression from PMID: 30772301	SOX2
CLDN7	SOX2 regulated genes in Keratinocyte Overexpression from PMID: 30772301	SOX2
TCEA1	SOX2 regulated genes in Keratinocyte Overexpression from PMID: 30772301	SOX2
KLC3	SOX2 regulated genes in Keratinocyte Overexpression from PMID: 30772301	SOX2
DPH5	SOX2 regulated genes in Keratinocyte Overexpression from PMID: 30772301	SOX2
PCBP2	SOX2 regulated genes in Keratinocyte Overexpression from PMID: 30772301	SOX2
NOL6	SOX2 regulated genes in Keratinocyte Overexpression from PMID: 30772301	SOX2
OOEP	SOX2 regulated genes in Keratinocyte Overexpression from PMID: 30772301	SOX2
NMT1	SOX2 regulated genes in Keratinocyte Overexpression from PMID: 30772301	SOX2
HR	SOX2 regulated genes in Keratinocyte Overexpression from PMID: 30772301	SOX2
DROSHA	SOX2 regulated genes in Keratinocyte Overexpression from PMID: 30772301	SOX2
PELP1	SOX2 regulated genes in Keratinocyte Overexpression from PMID: 30772301	SOX2
SPRR4	SOX2 regulated genes in Keratinocyte Overexpression from PMID: 30772301	SOX2
SNAPC4	SOX2 regulated genes in Keratinocyte Overexpression from PMID: 30772301	SOX2
TAF1C	SOX2 regulated genes in Keratinocyte Overexpression from PMID: 30772301	SOX2
CGNL1	SOX2 regulated genes in Keratinocyte Overexpression from PMID: 30772301	SOX2
WDR83OS	SOX2 regulated genes in Keratinocyte Overexpression from PMID: 30772301	SOX2
DUSP16	SOX2 regulated genes in Keratinocyte Overexpression from PMID: 30772301	SOX2
THOP1	SOX2 regulated genes in Keratinocyte Overexpression from PMID: 30772301	SOX2
SUSD4	SOX2 regulated genes in Keratinocyte Overexpression from PMID: 30772301	SOX2
CDC42SE1	SOX2 regulated genes in Keratinocyte Overexpression from PMID: 30772301	SOX2
MFF	SOX2 regulated genes in Keratinocyte Overexpression from PMID: 30772301	SOX2
IER5	SOX2 regulated genes in Keratinocyte Overexpression from PMID: 30772301	SOX2
RAD21	SOX2 regulated genes in Keratinocyte Overexpression from PMID: 30772301	SOX2
SETD5	SOX2 regulated genes in Keratinocyte Overexpression from PMID: 30772301	SOX2

MBTPS1	SOX2 regulated genes in Keratinocyte Overexpression from PMID: 30772301	SOX2
REXO1	SOX2 regulated genes in Keratinocyte Overexpression from PMID: 30772301	SOX2
SRPRB	SOX2 regulated genes in Keratinocyte Overexpression from PMID: 30772301	SOX2
AKTIP	SOX2 regulated genes in Keratinocyte Overexpression from PMID: 30772301	SOX2
RNF223	SOX2 regulated genes in Keratinocyte Overexpression from PMID: 30772301	SOX2
TIMM13	SOX2 regulated genes in Keratinocyte Overexpression from PMID: 30772301	SOX2
RABL3	SOX2 regulated genes in Keratinocyte Overexpression from PMID: 30772301	SOX2
XRRA1	SOX2 regulated genes in Keratinocyte Overexpression from PMID: 30772301	SOX2
PIGL	SOX2 regulated genes in Keratinocyte Overexpression from PMID: 30772301	SOX2
MARCKSL1	SOX2 regulated genes in Keratinocyte Overexpression from PMID: 30772301	SOX2
PUS7L	SOX2 regulated genes in Keratinocyte Overexpression from PMID: 30772301	SOX2
CCNE2	SOX2 regulated genes in Keratinocyte Overexpression from PMID: 30772301	SOX2
HOOK2	SOX2 regulated genes in Keratinocyte Overexpression from PMID: 30772301	SOX2
HNRNPH3	SOX2 regulated genes in Keratinocyte Overexpression from PMID: 30772301	SOX2
LONP1	SOX2 regulated genes in Keratinocyte Overexpression from PMID: 30772301	SOX2
MRPL53	SOX2 regulated genes in Keratinocyte Overexpression from PMID: 30772301	SOX2
ANXA9	SOX2 regulated genes in Keratinocyte Overexpression from PMID: 30772301	SOX2
GALR2	SOX2 regulated genes in Keratinocyte Overexpression from PMID: 30772301	SOX2
PRPF40A	SOX2 regulated genes in Keratinocyte Overexpression from PMID: 30772301	SOX2
TFCP2	SOX2 regulated genes in Keratinocyte Overexpression from PMID: 30772301	SOX2
RING1	SOX2 regulated genes in Keratinocyte Overexpression from PMID: 30772301	SOX2
GBF1	SOX2 regulated genes in Keratinocyte Overexpression from PMID: 30772301	SOX2
CCDC51	SOX2 regulated genes in Keratinocyte Overexpression from PMID: 30772301	SOX2
PDGFA	SOX2 regulated genes in Keratinocyte Overexpression from PMID: 30772301	SOX2
CEP72	SOX2 regulated genes in Keratinocyte Overexpression from PMID: 30772301	SOX2
HCN3	SOX2 regulated genes in Keratinocyte Overexpression from PMID: 30772301	SOX2
TMEM181	SOX2 regulated genes in Keratinocyte Overexpression from PMID: 30772301	SOX2
VSIG10L	SOX2 regulated genes in Keratinocyte Overexpression from PMID: 30772301	SOX2
CUL2	SOX2 regulated genes in Keratinocyte Overexpression from PMID: 30772301	SOX2
TSN	SOX2 regulated genes in Keratinocyte Overexpression from PMID: 30772301	SOX2
NKD1	SOX2 regulated genes in Keratinocyte Overexpression from PMID: 30772301	SOX2
SLC39A14	SOX2 regulated genes in Keratinocyte Overexpression from PMID: 30772301	SOX2
GRM5	SOX2 regulated genes in Keratinocyte Overexpression from PMID: 30772301	SOX2
NIT1	SOX2 regulated genes in Keratinocyte Overexpression from PMID: 30772301	SOX2
DND1	SOX2 regulated genes in Keratinocyte Overexpression from PMID: 30772301	SOX2
LMNTD1	SOX2 regulated genes in Keratinocyte Overexpression from PMID: 30772301	SOX2
CDH1	SOX2 regulated genes in Keratinocyte Overexpression from PMID: 30772301	SOX2
FRS2	SOX2 regulated genes in Keratinocyte Overexpression from PMID: 30772301	SOX2
AP4B1	SOX2 regulated genes in Keratinocyte Overexpression from PMID: 30772301	SOX2
SGK3	SOX2 regulated genes in Keratinocyte Overexpression from PMID: 30772301	SOX2
RAD23A	SOX2 regulated genes in Keratinocyte Overexpression from PMID: 30772301	SOX2
POM121	SOX2 regulated genes in Keratinocyte Overexpression from PMID: 30772301	SOX2
POM121C	SOX2 regulated genes in Keratinocyte Overexpression from PMID: 30772301	SOX2
BRICD5	SOX2 regulated genes in Keratinocyte Overexpression from PMID: 30772301	SOX2
TANC2	SOX2 regulated genes in Keratinocyte Overexpression from PMID: 30772301	SOX2
SLC41A1	SOX2 regulated genes in Keratinocyte Overexpression from PMID: 30772301	SOX2
CACNA2D2	SOX2 regulated genes in Keratinocyte Overexpression from PMID: 30772301	SOX2
RNF157	SOX2 regulated genes in Keratinocyte Overexpression from PMID: 30772301	SOX2
DAP3	SOX2 regulated genes in Keratinocyte Overexpression from PMID: 30772301	SOX2
MAP3K13	SOX2 regulated genes in Keratinocyte Overexpression from PMID: 30772301	SOX2
CNNM4	SOX2 regulated genes in Keratinocyte Overexpression from PMID: 30772301	SOX2
CWC27	SOX2 regulated genes in Keratinocyte Overexpression from PMID: 30772301	SOX2
MRPL55	SOX2 regulated genes in Keratinocyte Overexpression from PMID: 30772301	SOX2
RAI1	SOX2 regulated genes in Keratinocyte Overexpression from PMID: 30772301	SOX2
CCSAP	SOX2 regulated genes in Keratinocyte Overexpression from PMID: 30772301	SOX2
EZH2	SOX2 regulated genes in Keratinocyte Overexpression from PMID: 30772301	SOX2
ARHGAP27	SOX2 regulated genes in Keratinocyte Overexpression from PMID: 30772301	SOX2
SRFBP1	SOX2 regulated genes in Keratinocyte Overexpression from PMID: 30772301	SOX2
TMEM41B	SOX2 regulated genes in Keratinocyte Overexpression from PMID: 30772301	SOX2

QRICH2	SOX2 regulated genes in Keratinocyte Overexpression from PMID: 30772301	SOX2
TNNI1	SOX2 regulated genes in Keratinocyte Overexpression from PMID: 30772301	SOX2
TUBA4A	SOX2 regulated genes in Keratinocyte Overexpression from PMID: 30772301	SOX2
SYNRG	SOX2 regulated genes in Keratinocyte Overexpression from PMID: 30772301	SOX2
RARB	SOX2 regulated genes in Keratinocyte Overexpression from PMID: 30772301	SOX2
HSF4	SOX2 regulated genes in Keratinocyte Overexpression from PMID: 30772301	SOX2
SRSF1	SOX2 regulated genes in Keratinocyte Overexpression from PMID: 30772301	SOX2
DMPK	SOX2 regulated genes in Keratinocyte Overexpression from PMID: 30772301	SOX2
EIF4EBP3	SOX2 regulated genes in Keratinocyte Overexpression from PMID: 30772301	SOX2
C1orf131	SOX2 regulated genes in Keratinocyte Overexpression from PMID: 30772301	SOX2
IL11	SOX2 regulated genes in Keratinocyte Overexpression from PMID: 30772301	SOX2
SAXO2	SOX2 regulated genes in Keratinocyte Overexpression from PMID: 30772301	SOX2
PNKP	SOX2 regulated genes in Keratinocyte Overexpression from PMID: 30772301	SOX2
C1orf74	SOX2 regulated genes in Keratinocyte Overexpression from PMID: 30772301	SOX2
DVL1	SOX2 regulated genes in Keratinocyte Overexpression from PMID: 30772301	SOX2
SLC19A3	SOX2 regulated genes in Keratinocyte Overexpression from PMID: 30772301	SOX2
PMS1	SOX2 regulated genes in Keratinocyte Overexpression from PMID: 30772301	SOX2
ECI2	SOX2 regulated genes in Keratinocyte Overexpression from PMID: 30772301	SOX2
CDC42EP5	SOX2 regulated genes in Keratinocyte Overexpression from PMID: 30772301	SOX2
DPP9	SOX2 regulated genes in Keratinocyte Overexpression from PMID: 30772301	SOX2
MYL6B	SOX2 regulated genes in Keratinocyte Overexpression from PMID: 30772301	SOX2
DTL	SOX2 regulated genes in Keratinocyte Overexpression from PMID: 30772301	SOX2
PCGF2	SOX2 regulated genes in Keratinocyte Overexpression from PMID: 30772301	SOX2
TAF10	SOX2 regulated genes in Keratinocyte Overexpression from PMID: 30772301	SOX2
PCF11	SOX2 regulated genes in Keratinocyte Overexpression from PMID: 30772301	SOX2
SLC23A3	SOX2 regulated genes in Keratinocyte Overexpression from PMID: 30772301	SOX2
PROSER3	SOX2 regulated genes in Keratinocyte Overexpression from PMID: 30772301	SOX2
SETD6	SOX2 regulated genes in Keratinocyte Overexpression from PMID: 30772301	SOX2
PARL	SOX2 regulated genes in Keratinocyte Overexpression from PMID: 30772301	SOX2
IFRD2	SOX2 regulated genes in Keratinocyte Overexpression from PMID: 30772301	SOX2
RPL23A	SOX2 regulated genes in Keratinocyte Overexpression from PMID: 30772301	SOX2
LANCL2	SOX2 regulated genes in Keratinocyte Overexpression from PMID: 30772301	SOX2
HBEGF	SOX2 regulated genes in Keratinocyte Overexpression from PMID: 30772301	SOX2
YME1L1	SOX2 regulated genes in Keratinocyte Overexpression from PMID: 30772301	SOX2
THUMPD1	SOX2 regulated genes in Keratinocyte Overexpression from PMID: 30772301	SOX2
MRPL58	SOX2 regulated genes in Keratinocyte Overexpression from PMID: 30772301	SOX2
ANKRD39	SOX2 regulated genes in Keratinocyte Overexpression from PMID: 30772301	SOX2
AUNIP	SOX2 regulated genes in Keratinocyte Overexpression from PMID: 30772301	SOX2
CNST	SOX2 regulated genes in Keratinocyte Overexpression from PMID: 30772301	SOX2
ECSIT	SOX2 regulated genes in Keratinocyte Overexpression from PMID: 30772301	SOX2
RPS27	SOX2 regulated genes in Keratinocyte Overexpression from PMID: 30772301	SOX2
GRIN2A	SOX2 regulated genes in Keratinocyte Overexpression from PMID: 30772301	SOX2
HDGF	SOX2 regulated genes in Keratinocyte Overexpression from PMID: 30772301	SOX2
F12	SOX2 regulated genes in Keratinocyte Overexpression from PMID: 30772301	SOX2
SIK3	SOX2 regulated genes in Keratinocyte Overexpression from PMID: 30772301	SOX2
GLOD4	SOX2 regulated genes in Keratinocyte Overexpression from PMID: 30772301	SOX2
NDUFAF7	SOX2 regulated genes in Keratinocyte Overexpression from PMID: 30772301	SOX2
MDH2	SOX2 regulated genes in Keratinocyte Overexpression from PMID: 30772301	SOX2
SBNO1	SOX2 regulated genes in Keratinocyte Overexpression from PMID: 30772301	SOX2
GPR42	SOX2 regulated genes in Keratinocyte Overexpression from PMID: 30772301	SOX2
SIN3B	SOX2 regulated genes in Keratinocyte Overexpression from PMID: 30772301	SOX2
ESPNL	SOX2 regulated genes in Keratinocyte Overexpression from PMID: 30772301	SOX2
DHRS9	SOX2 regulated genes in Keratinocyte Overexpression from PMID: 30772301	SOX2
CCDC78	SOX2 regulated genes in Keratinocyte Overexpression from PMID: 30772301	SOX2
GTF2F1	SOX2 regulated genes in Keratinocyte Overexpression from PMID: 30772301	SOX2
DVL2	SOX2 regulated genes in Keratinocyte Overexpression from PMID: 30772301	SOX2
RECQL5	SOX2 regulated genes in Keratinocyte Overexpression from PMID: 30772301	SOX2
TATDN1	SOX2 regulated genes in Keratinocyte Overexpression from PMID: 30772301	SOX2
ALOX12	SOX2 regulated genes in Keratinocyte Overexpression from PMID: 30772301	SOX2

MRPS27	SOX2 regulated genes in Keratinocyte Overexpression from PMID: 30772301	SOX2
LSG1	SOX2 regulated genes in Keratinocyte Overexpression from PMID: 30772301	SOX2
METTL23	SOX2 regulated genes in Keratinocyte Overexpression from PMID: 30772301	SOX2
POGK	SOX2 regulated genes in Keratinocyte Overexpression from PMID: 30772301	SOX2
MRPS22	SOX2 regulated genes in Keratinocyte Overexpression from PMID: 30772301	SOX2
HP1BP3	SOX2 regulated genes in Keratinocyte Overexpression from PMID: 30772301	SOX2
CAPN5	SOX2 regulated genes in Keratinocyte Overexpression from PMID: 30772301	SOX2
SRCAP	SOX2 regulated genes in Keratinocyte Overexpression from PMID: 30772301	SOX2
GTF3C2	SOX2 regulated genes in Keratinocyte Overexpression from PMID: 30772301	SOX2
TMA7	SOX2 regulated genes in Keratinocyte Overexpression from PMID: 30772301	SOX2
PPP1R16A	SOX2 regulated genes in Keratinocyte Overexpression from PMID: 30772301	SOX2
HOXB5	SOX2 regulated genes in Keratinocyte Overexpression from PMID: 30772301	SOX2
ENOPH1	SOX2 regulated genes in Keratinocyte Overexpression from PMID: 30772301	SOX2
ZNF146	SOX2 regulated genes in Keratinocyte Overexpression from PMID: 30772301	SOX2
POLR2D	SOX2 regulated genes in Keratinocyte Overexpression from PMID: 30772301	SOX2
DCAF15	SOX2 regulated genes in Keratinocyte Overexpression from PMID: 30772301	SOX2
FAT1	SOX2 regulated genes in Keratinocyte Overexpression from PMID: 30772301	SOX2
SDR16C5	SOX2 regulated genes in Keratinocyte Overexpression from PMID: 30772301	SOX2
TRNT1	SOX2 regulated genes in Keratinocyte Overexpression from PMID: 30772301	SOX2
BCL2L12	SOX2 regulated genes in Keratinocyte Overexpression from PMID: 30772301	SOX2
LMBR1L	SOX2 regulated genes in Keratinocyte Overexpression from PMID: 30772301	SOX2
BLOC1S4	SOX2 regulated genes in Keratinocyte Overexpression from PMID: 30772301	SOX2
VANGL1	SOX2 regulated genes in Keratinocyte Overexpression from PMID: 30772301	SOX2
SAP30BP	SOX2 regulated genes in Keratinocyte Overexpression from PMID: 30772301	SOX2
CCDC158	SOX2 regulated genes in Keratinocyte Overexpression from PMID: 30772301	SOX2
CCDC91	SOX2 regulated genes in Keratinocyte Overexpression from PMID: 30772301	SOX2
SPATA24	SOX2 regulated genes in Keratinocyte Overexpression from PMID: 30772301	SOX2
C8orf76	SOX2 regulated genes in Keratinocyte Overexpression from PMID: 30772301	SOX2
ZHX1-C8orf76	SOX2 regulated genes in Keratinocyte Overexpression from PMID: 30772301	SOX2
MAZ	SOX2 regulated genes in Keratinocyte Overexpression from PMID: 30772301	SOX2
JUND	SOX2 regulated genes in Keratinocyte Overexpression from PMID: 30772301	SOX2
POLR3A	SOX2 regulated genes in Keratinocyte Overexpression from PMID: 30772301	SOX2
FOXH1	SOX2 regulated genes in Keratinocyte Overexpression from PMID: 30772301	SOX2
KIFC2	SOX2 regulated genes in Keratinocyte Overexpression from PMID: 30772301	SOX2
RGS16	SOX2 regulated genes in Keratinocyte Overexpression from PMID: 30772301	SOX2
SPIRE2	SOX2 regulated genes in Keratinocyte Overexpression from PMID: 30772301	SOX2
CFAP45	SOX2 regulated genes in Keratinocyte Overexpression from PMID: 30772301	SOX2
RNASEK-C17orf4[illegible]	SOX2 regulated genes in Keratinocyte Overexpression from PMID: 30772301	SOX2
C17orf49	SOX2 regulated genes in Keratinocyte Overexpression from PMID: 30772301	SOX2
BTF3L4	SOX2 regulated genes in Keratinocyte Overexpression from PMID: 30772301	SOX2
ATG101	SOX2 regulated genes in Keratinocyte Overexpression from PMID: 30772301	SOX2
SOS1	SOX2 regulated genes in Keratinocyte Overexpression from PMID: 30772301	SOX2
TMEM101	SOX2 regulated genes in Keratinocyte Overexpression from PMID: 30772301	SOX2
BAP1	SOX2 regulated genes in Keratinocyte Overexpression from PMID: 30772301	SOX2
PIK3R2	SOX2 regulated genes in Keratinocyte Overexpression from PMID: 30772301	SOX2
ZMYM1	SOX2 regulated genes in Keratinocyte Overexpression from PMID: 30772301	SOX2
B4GALT3	SOX2 regulated genes in Keratinocyte Overexpression from PMID: 30772301	SOX2
KRR1	SOX2 regulated genes in Keratinocyte Overexpression from PMID: 30772301	SOX2
KLHDC3	SOX2 regulated genes in Keratinocyte Overexpression from PMID: 30772301	SOX2
ARHGAP35	SOX2 regulated genes in Keratinocyte Overexpression from PMID: 30772301	SOX2
CXCR5	SOX2 regulated genes in Keratinocyte Overexpression from PMID: 30772301	SOX2
NMRAL1	SOX2 regulated genes in Keratinocyte Overexpression from PMID: 30772301	SOX2
SUPT7L	SOX2 regulated genes in Keratinocyte Overexpression from PMID: 30772301	SOX2
TMEM65	SOX2 regulated genes in Keratinocyte Overexpression from PMID: 30772301	SOX2
MCM2	SOX2 regulated genes in Keratinocyte Overexpression from PMID: 30772301	SOX2
HSPE1	SOX2 regulated genes in Keratinocyte Overexpression from PMID: 30772301	SOX2
MFSD2A	SOX2 regulated genes in Keratinocyte Overexpression from PMID: 30772301	SOX2
HMBS	SOX2 regulated genes in Keratinocyte Overexpression from PMID: 30772301	SOX2
SRPK2	SOX2 regulated genes in Keratinocyte Overexpression from PMID: 30772301	SOX2

SPC25	SOX2 regulated genes in Keratinocyte Overexpression from PMID: 30772301	SOX2
MAP4K4	SOX2 regulated genes in Keratinocyte Overexpression from PMID: 30772301	SOX2
UBA52	SOX2 regulated genes in Keratinocyte Overexpression from PMID: 30772301	SOX2
TTYH1	SOX2 regulated genes in Keratinocyte Overexpression from PMID: 30772301	SOX2
MMADHC	SOX2 regulated genes in Keratinocyte Overexpression from PMID: 30772301	SOX2
ZNF598	SOX2 regulated genes in Keratinocyte Overexpression from PMID: 30772301	SOX2
PHLDB3	SOX2 regulated genes in Keratinocyte Overexpression from PMID: 30772301	SOX2
FBRSL1	SOX2 regulated genes in Keratinocyte Overexpression from PMID: 30772301	SOX2
USP7	SOX2 regulated genes in Keratinocyte Overexpression from PMID: 30772301	SOX2
OGG1	SOX2 regulated genes in Keratinocyte Overexpression from PMID: 30772301	SOX2
TEX19	SOX2 regulated genes in Keratinocyte Overexpression from PMID: 30772301	SOX2
SMARCD2	SOX2 regulated genes in Keratinocyte Overexpression from PMID: 30772301	SOX2
CHD1L	SOX2 regulated genes in Keratinocyte Overexpression from PMID: 30772301	SOX2
MORN2	SOX2 regulated genes in Keratinocyte Overexpression from PMID: 30772301	SOX2
PWWP2A	SOX2 regulated genes in Keratinocyte Overexpression from PMID: 30772301	SOX2
RPS13	SOX2 regulated genes in Keratinocyte Overexpression from PMID: 30772301	SOX2
OPRD1	SOX2 regulated genes in Keratinocyte Overexpression from PMID: 30772301	SOX2
ZBTB9	SOX2 regulated genes in Keratinocyte Overexpression from PMID: 30772301	SOX2
DDX54	SOX2 regulated genes in Keratinocyte Overexpression from PMID: 30772301	SOX2
UNG	SOX2 regulated genes in Keratinocyte Overexpression from PMID: 30772301	SOX2
EIF2D	SOX2 regulated genes in Keratinocyte Overexpression from PMID: 30772301	SOX2
CHMP1A	SOX2 regulated genes in Keratinocyte Overexpression from PMID: 30772301	SOX2
ICAM4	SOX2 regulated genes in Keratinocyte Overexpression from PMID: 30772301	SOX2
ATRIP	SOX2 regulated genes in Keratinocyte Overexpression from PMID: 30772301	SOX2
ATP2A2	SOX2 regulated genes in Keratinocyte Overexpression from PMID: 30772301	SOX2
ERBB3	SOX2 regulated genes in Keratinocyte Overexpression from PMID: 30772301	SOX2
HARS2	SOX2 regulated genes in Keratinocyte Overexpression from PMID: 30772301	SOX2
TP53BP2	SOX2 regulated genes in Keratinocyte Overexpression from PMID: 30772301	SOX2
PIGV	SOX2 regulated genes in Keratinocyte Overexpression from PMID: 30772301	SOX2
IGSF8	SOX2 regulated genes in Keratinocyte Overexpression from PMID: 30772301	SOX2
FIGNL1	SOX2 regulated genes in Keratinocyte Overexpression from PMID: 30772301	SOX2
CENPL	SOX2 regulated genes in Keratinocyte Overexpression from PMID: 30772301	SOX2
MON1A	SOX2 regulated genes in Keratinocyte Overexpression from PMID: 30772301	SOX2
LRRC56	SOX2 regulated genes in Keratinocyte Overexpression from PMID: 30772301	SOX2
AGRN	SOX2 regulated genes in Keratinocyte Overexpression from PMID: 30772301	SOX2
UBE2T	SOX2 regulated genes in Keratinocyte Overexpression from PMID: 30772301	SOX2
C16orf96	SOX2 regulated genes in Keratinocyte Overexpression from PMID: 30772301	SOX2
SDS	SOX2 regulated genes in Keratinocyte Overexpression from PMID: 30772301	SOX2
CRIP3	SOX2 regulated genes in Keratinocyte Overexpression from PMID: 30772301	SOX2
PRR36	SOX2 regulated genes in Keratinocyte Overexpression from PMID: 30772301	SOX2
LIN9	SOX2 regulated genes in Keratinocyte Overexpression from PMID: 30772301	SOX2
CYTH2	SOX2 regulated genes in Keratinocyte Overexpression from PMID: 30772301	SOX2
NPHP1	SOX2 regulated genes in Keratinocyte Overexpression from PMID: 30772301	SOX2
ACOT4	SOX2 regulated genes in Keratinocyte Overexpression from PMID: 30772301	SOX2
ADGRL2	SOX2 regulated genes in Keratinocyte Overexpression from PMID: 30772301	SOX2
C6orf141	SOX2 regulated genes in Keratinocyte Overexpression from PMID: 30772301	SOX2
HSPA4	SOX2 regulated genes in Keratinocyte Overexpression from PMID: 30772301	SOX2
XAB2	SOX2 regulated genes in Keratinocyte Overexpression from PMID: 30772301	SOX2
TRPM8	SOX2 regulated genes in Keratinocyte Overexpression from PMID: 30772301	SOX2
NOP16	SOX2 regulated genes in Keratinocyte Overexpression from PMID: 30772301	SOX2
PMF1-BGLAP	SOX2 regulated genes in Keratinocyte Overexpression from PMID: 30772301	SOX2
PMF1	SOX2 regulated genes in Keratinocyte Overexpression from PMID: 30772301	SOX2
ZBTB11	SOX2 regulated genes in Keratinocyte Overexpression from PMID: 30772301	SOX2
TMEM120A	SOX2 regulated genes in Keratinocyte Overexpression from PMID: 30772301	SOX2
CNPPD1	SOX2 regulated genes in Keratinocyte Overexpression from PMID: 30772301	SOX2
NEURL4	SOX2 regulated genes in Keratinocyte Overexpression from PMID: 30772301	SOX2
POLR3K	SOX2 regulated genes in Keratinocyte Overexpression from PMID: 30772301	SOX2
DVL3	SOX2 regulated genes in Keratinocyte Overexpression from PMID: 30772301	SOX2
RAVER1	SOX2 regulated genes in Keratinocyte Overexpression from PMID: 30772301	SOX2

CHEK1	SOX2 regulated genes in Keratinocyte Overexpression from PMID: 30772301	SOX2
TMEM238	SOX2 regulated genes in Keratinocyte Overexpression from PMID: 30772301	SOX2
UCHL5	SOX2 regulated genes in Keratinocyte Overexpression from PMID: 30772301	SOX2
CHD1	SOX2 regulated genes in Keratinocyte Overexpression from PMID: 30772301	SOX2
SLC25A3	SOX2 regulated genes in Keratinocyte Overexpression from PMID: 30772301	SOX2
ZNF32	SOX2 regulated genes in Keratinocyte Overexpression from PMID: 30772301	SOX2
SENP8	SOX2 regulated genes in Keratinocyte Overexpression from PMID: 30772301	SOX2
METTL5	SOX2 regulated genes in Keratinocyte Overexpression from PMID: 30772301	SOX2
ZNF566	SOX2 regulated genes in Keratinocyte Overexpression from PMID: 30772301	SOX2
HAPLN3	SOX2 regulated genes in Keratinocyte Overexpression from PMID: 30772301	SOX2
SMC3	SOX2 regulated genes in Keratinocyte Overexpression from PMID: 30772301	SOX2
GADD45B	SOX2 regulated genes in Keratinocyte Overexpression from PMID: 30772301	SOX2
MAP6	SOX2 regulated genes in Keratinocyte Overexpression from PMID: 30772301	SOX2
ANKRD52	SOX2 regulated genes in Keratinocyte Overexpression from PMID: 30772301	SOX2
PALMD	SOX2 regulated genes in Keratinocyte Overexpression from PMID: 30772301	SOX2
EGR2	SOX2 regulated genes in Keratinocyte Overexpression from PMID: 30772301	SOX2
PRR11	SOX2 regulated genes in Keratinocyte Overexpression from PMID: 30772301	SOX2
CWC25	SOX2 regulated genes in Keratinocyte Overexpression from PMID: 30772301	SOX2
HDAC3	SOX2 regulated genes in Keratinocyte Overexpression from PMID: 30772301	SOX2
DHX37	SOX2 regulated genes in Keratinocyte Overexpression from PMID: 30772301	SOX2
NUDCD1	SOX2 regulated genes in Keratinocyte Overexpression from PMID: 30772301	SOX2
LIPH	SOX2 regulated genes in Keratinocyte Overexpression from PMID: 30772301	SOX2
KIF21B	SOX2 regulated genes in Keratinocyte Overexpression from PMID: 30772301	SOX2
DHPS	SOX2 regulated genes in Keratinocyte Overexpression from PMID: 30772301	SOX2
TRNP1	SOX2 regulated genes in Keratinocyte Overexpression from PMID: 30772301	SOX2
C2CD2L	SOX2 regulated genes in Keratinocyte Overexpression from PMID: 30772301	SOX2
AMBRA1	SOX2 regulated genes in Keratinocyte Overexpression from PMID: 30772301	SOX2
LCLAT1	SOX2 regulated genes in Keratinocyte Overexpression from PMID: 30772301	SOX2
MRPL28	SOX2 regulated genes in Keratinocyte Overexpression from PMID: 30772301	SOX2
ANGPTL4	SOX2 regulated genes in Keratinocyte Overexpression from PMID: 30772301	SOX2
PLEKHA1	SOX2 regulated genes in Keratinocyte Overexpression from PMID: 30772301	SOX2
UBXN1	SOX2 regulated genes in Keratinocyte Overexpression from PMID: 30772301	SOX2
SDHB	SOX2 regulated genes in Keratinocyte Overexpression from PMID: 30772301	SOX2
GALE	SOX2 regulated genes in Keratinocyte Overexpression from PMID: 30772301	SOX2
ORAI2	SOX2 regulated genes in Keratinocyte Overexpression from PMID: 30772301	SOX2
AMMECR1L	SOX2 regulated genes in Keratinocyte Overexpression from PMID: 30772301	SOX2
FIGLA	SOX2 regulated genes in Keratinocyte Overexpression from PMID: 30772301	SOX2
BTBD19	SOX2 regulated genes in Keratinocyte Overexpression from PMID: 30772301	SOX2
ZRANB2	SOX2 regulated genes in Keratinocyte Overexpression from PMID: 30772301	SOX2
PTCHD4	SOX2 regulated genes in Keratinocyte Overexpression from PMID: 30772301	SOX2
PRPF40B	SOX2 regulated genes in Keratinocyte Overexpression from PMID: 30772301	SOX2
RTF1	SOX2 regulated genes in Keratinocyte Overexpression from PMID: 30772301	SOX2
GPAT4	SOX2 regulated genes in Keratinocyte Overexpression from PMID: 30772301	SOX2
SMC4	SOX2 regulated genes in Keratinocyte Overexpression from PMID: 30772301	SOX2
SPTY2D1	SOX2 regulated genes in Keratinocyte Overexpression from PMID: 30772301	SOX2
GATA3	SOX2 regulated genes in Keratinocyte Overexpression from PMID: 30772301	SOX2
ZNF296	SOX2 regulated genes in Keratinocyte Overexpression from PMID: 30772301	SOX2
KIF9	SOX2 regulated genes in Keratinocyte Overexpression from PMID: 30772301	SOX2
AZIN2	SOX2 regulated genes in Keratinocyte Overexpression from PMID: 30772301	SOX2
INTS5	SOX2 regulated genes in Keratinocyte Overexpression from PMID: 30772301	SOX2
SULT2B1	SOX2 regulated genes in Keratinocyte Overexpression from PMID: 30772301	SOX2
ALOX15B	SOX2 regulated genes in Keratinocyte Overexpression from PMID: 30772301	SOX2
TIMM17A	SOX2 regulated genes in Keratinocyte Overexpression from PMID: 30772301	SOX2
CCNL1	SOX2 regulated genes in Keratinocyte Overexpression from PMID: 30772301	SOX2
KLHL29	SOX2 regulated genes in Keratinocyte Overexpression from PMID: 30772301	SOX2
RAD54L2	SOX2 regulated genes in Keratinocyte Overexpression from PMID: 30772301	SOX2
LLGL2	SOX2 regulated genes in Keratinocyte Overexpression from PMID: 30772301	SOX2
CYBRD1	SOX2 regulated genes in Keratinocyte Overexpression from PMID: 30772301	SOX2
DPY30	SOX2 regulated genes in Keratinocyte Overexpression from PMID: 30772301	SOX2

C2orf69	SOX2 regulated genes in Keratinocyte Overexpression from PMID: 30772301	SOX2
CEP83	SOX2 regulated genes in Keratinocyte Overexpression from PMID: 30772301	SOX2
NCAPD3	SOX2 regulated genes in Keratinocyte Overexpression from PMID: 30772301	SOX2
RNF220	SOX2 regulated genes in Keratinocyte Overexpression from PMID: 30772301	SOX2
USP42	SOX2 regulated genes in Keratinocyte Overexpression from PMID: 30772301	SOX2
RFC4	SOX2 regulated genes in Keratinocyte Overexpression from PMID: 30772301	SOX2
KCTD15	SOX2 regulated genes in Keratinocyte Overexpression from PMID: 30772301	SOX2
ZNF239	SOX2 regulated genes in Keratinocyte Overexpression from PMID: 30772301	SOX2
RSRC2	SOX2 regulated genes in Keratinocyte Overexpression from PMID: 30772301	SOX2
RTKN	SOX2 regulated genes in Keratinocyte Overexpression from PMID: 30772301	SOX2
ZNF780A	SOX2 regulated genes in Keratinocyte Overexpression from PMID: 30772301	SOX2
NVL	SOX2 regulated genes in Keratinocyte Overexpression from PMID: 30772301	SOX2
TUFT1	SOX2 regulated genes in Keratinocyte Overexpression from PMID: 30772301	SOX2
SRP19	SOX2 regulated genes in Keratinocyte Overexpression from PMID: 30772301	SOX2
YARS2	SOX2 regulated genes in Keratinocyte Overexpression from PMID: 30772301	SOX2
TDRD5	SOX2 regulated genes in Keratinocyte Overexpression from PMID: 30772301	SOX2
RPS25	SOX2 regulated genes in Keratinocyte Overexpression from PMID: 30772301	SOX2
ECT2	SOX2 regulated genes in Keratinocyte Overexpression from PMID: 30772301	SOX2
MARK4	SOX2 regulated genes in Keratinocyte Overexpression from PMID: 30772301	SOX2
GRM2	SOX2 regulated genes in Keratinocyte Overexpression from PMID: 30772301	SOX2
ZNF180	SOX2 regulated genes in Keratinocyte Overexpression from PMID: 30772301	SOX2
ACSM3	SOX2 regulated genes in Keratinocyte Overexpression from PMID: 30772301	SOX2
PURA	SOX2 regulated genes in Keratinocyte Overexpression from PMID: 30772301	SOX2
SCAF1	SOX2 regulated genes in Keratinocyte Overexpression from PMID: 30772301	SOX2
ZBTB49	SOX2 regulated genes in Keratinocyte Overexpression from PMID: 30772301	SOX2
FBXL14	SOX2 regulated genes in Keratinocyte Overexpression from PMID: 30772301	SOX2
DDX41	SOX2 regulated genes in Keratinocyte Overexpression from PMID: 30772301	SOX2
SLC30A6	SOX2 regulated genes in Keratinocyte Overexpression from PMID: 30772301	SOX2
MRPL37	SOX2 regulated genes in Keratinocyte Overexpression from PMID: 30772301	SOX2
OTUD7B	SOX2 regulated genes in Keratinocyte Overexpression from PMID: 30772301	SOX2
UBE2J2	SOX2 regulated genes in Keratinocyte Overexpression from PMID: 30772301	SOX2
HMGCS2	SOX2 regulated genes in Keratinocyte Overexpression from PMID: 30772301	SOX2
SLC23A1	SOX2 regulated genes in Keratinocyte Overexpression from PMID: 30772301	SOX2
STIL	SOX2 regulated genes in Keratinocyte Overexpression from PMID: 30772301	SOX2
EIF3E	SOX2 regulated genes in Keratinocyte Overexpression from PMID: 30772301	SOX2
COPS7B	SOX2 regulated genes in Keratinocyte Overexpression from PMID: 30772301	SOX2
DPM3	SOX2 regulated genes in Keratinocyte Overexpression from PMID: 30772301	SOX2
SLC6A15	SOX2 regulated genes in Keratinocyte Overexpression from PMID: 30772301	SOX2
ENC1	SOX2 regulated genes in Keratinocyte Overexpression from PMID: 30772301	SOX2
TIMM44	SOX2 regulated genes in Keratinocyte Overexpression from PMID: 30772301	SOX2
CCDC106	SOX2 regulated genes in Keratinocyte Overexpression from PMID: 30772301	SOX2
ZNF131	SOX2 regulated genes in Keratinocyte Overexpression from PMID: 30772301	SOX2
PTP4A1	SOX2 regulated genes in Keratinocyte Overexpression from PMID: 30772301	SOX2
UBIAD1	SOX2 regulated genes in Keratinocyte Overexpression from PMID: 30772301	SOX2
SLC7A14	SOX2 regulated genes in Keratinocyte Overexpression from PMID: 30772301	SOX2
MRPL12	SOX2 regulated genes in Keratinocyte Overexpression from PMID: 30772301	SOX2
NUDT1	SOX2 regulated genes in Keratinocyte Overexpression from PMID: 30772301	SOX2
TMEM138	SOX2 regulated genes in Keratinocyte Overexpression from PMID: 30772301	SOX2
PBLD	SOX2 regulated genes in Keratinocyte Overexpression from PMID: 30772301	SOX2
PRR14	SOX2 regulated genes in Keratinocyte Overexpression from PMID: 30772301	SOX2
GGPS1	SOX2 regulated genes in Keratinocyte Overexpression from PMID: 30772301	SOX2
SLC45A1	SOX2 regulated genes in Keratinocyte Overexpression from PMID: 30772301	SOX2
PTHLH	SOX2 regulated genes in Keratinocyte Overexpression from PMID: 30772301	SOX2
FAM83E	SOX2 regulated genes in Keratinocyte Overexpression from PMID: 30772301	SOX2
KCNJ13	SOX2 regulated genes in Keratinocyte Overexpression from PMID: 30772301	SOX2
COA7	SOX2 regulated genes in Keratinocyte Overexpression from PMID: 30772301	SOX2
TOMM7	SOX2 regulated genes in Keratinocyte Overexpression from PMID: 30772301	SOX2
KRAS	SOX2 regulated genes in Keratinocyte Overexpression from PMID: 30772301	SOX2
HS2ST1	SOX2 regulated genes in Keratinocyte Overexpression from PMID: 30772301	SOX2

NEK8	SOX2 regulated genes in Keratinocyte Overexpression from PMID: 30772301	SOX2
ZNF23	SOX2 regulated genes in Keratinocyte Overexpression from PMID: 30772301	SOX2
EPOP	SOX2 regulated genes in Keratinocyte Overexpression from PMID: 30772301	SOX2
NT5E	SOX2 regulated genes in Keratinocyte Overexpression from PMID: 30772301	SOX2
S100A1	SOX2 regulated genes in Keratinocyte Overexpression from PMID: 30772301	SOX2
FUT1	SOX2 regulated genes in Keratinocyte Overexpression from PMID: 30772301	SOX2
POLR3D	SOX2 regulated genes in Keratinocyte Overexpression from PMID: 30772301	SOX2
EGFR	SOX2 regulated genes in Keratinocyte Overexpression from PMID: 30772301	SOX2
ZNF446	SOX2 regulated genes in Keratinocyte Overexpression from PMID: 30772301	SOX2
PIH1D2	SOX2 regulated genes in Keratinocyte Overexpression from PMID: 30772301	SOX2
CDK5RAP2	SOX2 regulated genes in Keratinocyte Overexpression from PMID: 30772301	SOX2
VPS37C	SOX2 regulated genes in Keratinocyte Overexpression from PMID: 30772301	SOX2
GALK1	SOX2 regulated genes in Keratinocyte Overexpression from PMID: 30772301	SOX2
G3BP1	SOX2 regulated genes in Keratinocyte Overexpression from PMID: 30772301	SOX2
BBS2	SOX2 regulated genes in Keratinocyte Overexpression from PMID: 30772301	SOX2
OVGP1	SOX2 regulated genes in Keratinocyte Overexpression from PMID: 30772301	SOX2
FBXO33	SOX2 regulated genes in Keratinocyte Overexpression from PMID: 30772301	SOX2
EPN3	SOX2 regulated genes in Keratinocyte Overexpression from PMID: 30772301	SOX2
FOXO6	SOX2 regulated genes in Keratinocyte Overexpression from PMID: 30772301	SOX2
ZDHHC1	SOX2 regulated genes in Keratinocyte Overexpression from PMID: 30772301	SOX2
PHOSPHO2	SOX2 regulated genes in Keratinocyte Overexpression from PMID: 30772301	SOX2
LRPPRC	SOX2 regulated genes in Keratinocyte Overexpression from PMID: 30772301	SOX2
RNF2	SOX2 regulated genes in Keratinocyte Overexpression from PMID: 30772301	SOX2
ALDH3B2	SOX2 regulated genes in Keratinocyte Overexpression from PMID: 30772301	SOX2
BACE1	SOX2 regulated genes in Keratinocyte Overexpression from PMID: 30772301	SOX2
ABCB6	SOX2 regulated genes in Keratinocyte Overexpression from PMID: 30772301	SOX2
IMP3	SOX2 regulated genes in Keratinocyte Overexpression from PMID: 30772301	SOX2
PSMD2	SOX2 regulated genes in Keratinocyte Overexpression from PMID: 30772301	SOX2
KCNJ16	SOX2 regulated genes in Keratinocyte Overexpression from PMID: 30772301	SOX2
LYG1	SOX2 regulated genes in Keratinocyte Overexpression from PMID: 30772301	SOX2
ZNF251	SOX2 regulated genes in Keratinocyte Overexpression from PMID: 30772301	SOX2
BANP	SOX2 regulated genes in Keratinocyte Overexpression from PMID: 30772301	SOX2
TAMM41	SOX2 regulated genes in Keratinocyte Overexpression from PMID: 30772301	SOX2
MIER2	SOX2 regulated genes in Keratinocyte Overexpression from PMID: 30772301	SOX2
MAB21L3	SOX2 regulated genes in Keratinocyte Overexpression from PMID: 30772301	SOX2
DAXX	SOX2 regulated genes in Keratinocyte Overexpression from PMID: 30772301	SOX2
GIPR	SOX2 regulated genes in Keratinocyte Overexpression from PMID: 30772301	SOX2
HOXD4	SOX2 regulated genes in Keratinocyte Overexpression from PMID: 30772301	SOX2
DDX10	SOX2 regulated genes in Keratinocyte Overexpression from PMID: 30772301	SOX2
NSD1	SOX2 regulated genes in Keratinocyte Overexpression from PMID: 30772301	SOX2
ABHD14A-ACY1	SOX2 regulated genes in Keratinocyte Overexpression from PMID: 30772301	SOX2
ACY1	SOX2 regulated genes in Keratinocyte Overexpression from PMID: 30772301	SOX2
RAD9B	SOX2 regulated genes in Keratinocyte Overexpression from PMID: 30772301	SOX2
CCDC71L	SOX2 regulated genes in Keratinocyte Overexpression from PMID: 30772301	SOX2
MFN1	SOX2 regulated genes in Keratinocyte Overexpression from PMID: 30772301	SOX2
SLC16A11	SOX2 regulated genes in Keratinocyte Overexpression from PMID: 30772301	SOX2
CENPE	SOX2 regulated genes in Keratinocyte Overexpression from PMID: 30772301	SOX2
PSMD6	SOX2 regulated genes in Keratinocyte Overexpression from PMID: 30772301	SOX2
RHBDF2	SOX2 regulated genes in Keratinocyte Overexpression from PMID: 30772301	SOX2
HMG20B	SOX2 regulated genes in Keratinocyte Overexpression from PMID: 30772301	SOX2
ZSWIM4	SOX2 regulated genes in Keratinocyte Overexpression from PMID: 30772301	SOX2
IFT122	SOX2 regulated genes in Keratinocyte Overexpression from PMID: 30772301	SOX2
NOS1AP	SOX2 regulated genes in Keratinocyte Overexpression from PMID: 30772301	SOX2
ANAPC13	SOX2 regulated genes in Keratinocyte Overexpression from PMID: 30772301	SOX2
CDC23	SOX2 regulated genes in Keratinocyte Overexpression from PMID: 30772301	SOX2
CLCN2	SOX2 regulated genes in Keratinocyte Overexpression from PMID: 30772301	SOX2
ZNF445	SOX2 regulated genes in Keratinocyte Overexpression from PMID: 30772301	SOX2
CDC73	SOX2 regulated genes in Keratinocyte Overexpression from PMID: 30772301	SOX2
ASAP3	SOX2 regulated genes in Keratinocyte Overexpression from PMID: 30772301	SOX2

RFNG	SOX2 regulated genes in Keratinocyte Overexpression from PMID: 30772301	SOX2
LEMD3	SOX2 regulated genes in Keratinocyte Overexpression from PMID: 30772301	SOX2
MAPK6	SOX2 regulated genes in Keratinocyte Overexpression from PMID: 30772301	SOX2
ZNF628	SOX2 regulated genes in Keratinocyte Overexpression from PMID: 30772301	SOX2
IMMT	SOX2 regulated genes in Keratinocyte Overexpression from PMID: 30772301	SOX2
AKT1S1	SOX2 regulated genes in Keratinocyte Overexpression from PMID: 30772301	SOX2
RPAP1	SOX2 regulated genes in Keratinocyte Overexpression from PMID: 30772301	SOX2
TAF12	SOX2 regulated genes in Keratinocyte Overexpression from PMID: 30772301	SOX2
MFSD3	SOX2 regulated genes in Keratinocyte Overexpression from PMID: 30772301	SOX2
KIAA0895L	SOX2 regulated genes in Keratinocyte Overexpression from PMID: 30772301	SOX2
ERCC8	SOX2 regulated genes in Keratinocyte Overexpression from PMID: 30772301	SOX2
TRIM62	SOX2 regulated genes in Keratinocyte Overexpression from PMID: 30772301	SOX2
YAF2	SOX2 regulated genes in Keratinocyte Overexpression from PMID: 30772301	SOX2
GTF2H5	SOX2 regulated genes in Keratinocyte Overexpression from PMID: 30772301	SOX2
NDUFA7	SOX2 regulated genes in Keratinocyte Overexpression from PMID: 30772301	SOX2
MSI2	SOX2 regulated genes in Keratinocyte Overexpression from PMID: 30772301	SOX2
ABHD17A	SOX2 regulated genes in Keratinocyte Overexpression from PMID: 30772301	SOX2
PLEKHJ1	SOX2 regulated genes in Keratinocyte Overexpression from PMID: 30772301	SOX2
TAF15	SOX2 regulated genes in Keratinocyte Overexpression from PMID: 30772301	SOX2
NMNAT3	SOX2 regulated genes in Keratinocyte Overexpression from PMID: 30772301	SOX2
ZNF575	SOX2 regulated genes in Keratinocyte Overexpression from PMID: 30772301	SOX2
UBXN8	SOX2 regulated genes in Keratinocyte Overexpression from PMID: 30772301	SOX2
GZMM	SOX2 regulated genes in Keratinocyte Overexpression from PMID: 30772301	SOX2
ASPM	SOX2 regulated genes in Keratinocyte Overexpression from PMID: 30772301	SOX2
ZNF700	SOX2 regulated genes in Keratinocyte Overexpression from PMID: 30772301	SOX2
ZNF878	SOX2 regulated genes in Keratinocyte Overexpression from PMID: 30772301	SOX2
WDR35	SOX2 regulated genes in Keratinocyte Overexpression from PMID: 30772301	SOX2
NME5	SOX2 regulated genes in Keratinocyte Overexpression from PMID: 30772301	SOX2
STXBP2	SOX2 regulated genes in Keratinocyte Overexpression from PMID: 30772301	SOX2
SZT2	SOX2 regulated genes in Keratinocyte Overexpression from PMID: 30772301	SOX2
MRFAP1L1	SOX2 regulated genes in Keratinocyte Overexpression from PMID: 30772301	SOX2
MRFAP1	SOX2 regulated genes in Keratinocyte Overexpression from PMID: 30772301	SOX2
CACTIN	SOX2 regulated genes in Keratinocyte Overexpression from PMID: 30772301	SOX2
HPCA	SOX2 regulated genes in Keratinocyte Overexpression from PMID: 30772301	SOX2
PGLS	SOX2 regulated genes in Keratinocyte Overexpression from PMID: 30772301	SOX2
NAPA	SOX2 regulated genes in Keratinocyte Overexpression from PMID: 30772301	SOX2
PRIM1	SOX2 regulated genes in Keratinocyte Overexpression from PMID: 30772301	SOX2
SLC20A1	SOX2 regulated genes in Keratinocyte Overexpression from PMID: 30772301	SOX2
BBS5	SOX2 regulated genes in Keratinocyte Overexpression from PMID: 30772301	SOX2
GPA33	SOX2 regulated genes in Keratinocyte Overexpression from PMID: 30772301	SOX2
NOTUM	SOX2 regulated genes in Keratinocyte Overexpression from PMID: 30772301	SOX2
RPS9	SOX2 regulated genes in Keratinocyte Overexpression from PMID: 30772301	SOX2
PCYT2	SOX2 regulated genes in Keratinocyte Overexpression from PMID: 30772301	SOX2
ASCL4	SOX2 regulated genes in Keratinocyte Overexpression from PMID: 30772301	SOX2
DNAH9	SOX2 regulated genes in Keratinocyte Overexpression from PMID: 30772301	SOX2
CIB1	SOX2 regulated genes in Keratinocyte Overexpression from PMID: 30772301	SOX2
CAMSAP2	SOX2 regulated genes in Keratinocyte Overexpression from PMID: 30772301	SOX2
ATG4D	SOX2 regulated genes in Keratinocyte Overexpression from PMID: 30772301	SOX2
ABCB10	SOX2 regulated genes in Keratinocyte Overexpression from PMID: 30772301	SOX2
ABTB1	SOX2 regulated genes in Keratinocyte Overexpression from PMID: 30772301	SOX2
SLC12A8	SOX2 regulated genes in Keratinocyte Overexpression from PMID: 30772301	SOX2
MAT2A	SOX2 regulated genes in Keratinocyte Overexpression from PMID: 30772301	SOX2
CYP4F11	SOX2 regulated genes in Keratinocyte Overexpression from PMID: 30772301	SOX2
CYP4A11	SOX2 regulated genes in Keratinocyte Overexpression from PMID: 30772301	SOX2
GNB1	SOX2 regulated genes in Keratinocyte Overexpression from PMID: 30772301	SOX2
IER2	SOX2 regulated genes in Keratinocyte Overexpression from PMID: 30772301	SOX2
RBAK	SOX2 regulated genes in Keratinocyte Overexpression from PMID: 30772301	SOX2
PODXL2	SOX2 regulated genes in Keratinocyte Overexpression from PMID: 30772301	SOX2
MARVELD2	SOX2 regulated genes in Keratinocyte Overexpression from PMID: 30772301	SOX2

POLD1	SOX2 regulated genes in Keratinocyte Overexpression from PMID: 30772301	SOX2
RPL28	SOX2 regulated genes in Keratinocyte Overexpression from PMID: 30772301	SOX2
BMPR2	SOX2 regulated genes in Keratinocyte Overexpression from PMID: 30772301	SOX2
LARP7	SOX2 regulated genes in Keratinocyte Overexpression from PMID: 30772301	SOX2
VIL1	SOX2 regulated genes in Keratinocyte Overexpression from PMID: 30772301	SOX2
TIMM29	SOX2 regulated genes in Keratinocyte Overexpression from PMID: 30772301	SOX2
PARPBP	SOX2 regulated genes in Keratinocyte Overexpression from PMID: 30772301	SOX2
TMC6	SOX2 regulated genes in Keratinocyte Overexpression from PMID: 30772301	SOX2
FAM222B	SOX2 regulated genes in Keratinocyte Overexpression from PMID: 30772301	SOX2
CUL1	SOX2 regulated genes in Keratinocyte Overexpression from PMID: 30772301	SOX2
GOLGA7	SOX2 regulated genes in Keratinocyte Overexpression from PMID: 30772301	SOX2
SRSF11	SOX2 regulated genes in Keratinocyte Overexpression from PMID: 30772301	SOX2
INO80B	SOX2 regulated genes in Keratinocyte Overexpression from PMID: 30772301	SOX2
ZNF574	SOX2 regulated genes in Keratinocyte Overexpression from PMID: 30772301	SOX2
C19orf25	SOX2 regulated genes in Keratinocyte Overexpression from PMID: 30772301	SOX2
RASSF1	SOX2 regulated genes in Keratinocyte Overexpression from PMID: 30772301	SOX2
CIT	SOX2 regulated genes in Keratinocyte Overexpression from PMID: 30772301	SOX2
CDC42EP3	SOX2 regulated genes in Keratinocyte Overexpression from PMID: 30772301	SOX2
DPYSL4	SOX2 regulated genes in Keratinocyte Overexpression from PMID: 30772301	SOX2
CCDC73	SOX2 regulated genes in Keratinocyte Overexpression from PMID: 30772301	SOX2
PRRT3	SOX2 regulated genes in Keratinocyte Overexpression from PMID: 30772301	SOX2
SPTBN2	SOX2 regulated genes in Keratinocyte Overexpression from PMID: 30772301	SOX2
RYK	SOX2 regulated genes in Keratinocyte Overexpression from PMID: 30772301	SOX2
SPINT2	SOX2 regulated genes in Keratinocyte Overexpression from PMID: 30772301	SOX2
IFT20	SOX2 regulated genes in Keratinocyte Overexpression from PMID: 30772301	SOX2
PTRHD1	SOX2 regulated genes in Keratinocyte Overexpression from PMID: 30772301	SOX2
CCDC43	SOX2 regulated genes in Keratinocyte Overexpression from PMID: 30772301	SOX2
DCTN2	SOX2 regulated genes in Keratinocyte Overexpression from PMID: 30772301	SOX2
LRRC46	SOX2 regulated genes in Keratinocyte Overexpression from PMID: 30772301	SOX2
TAF1A	SOX2 regulated genes in Keratinocyte Overexpression from PMID: 30772301	SOX2
AAMP	SOX2 regulated genes in Keratinocyte Overexpression from PMID: 30772301	SOX2
ACVR1B	SOX2 regulated genes in Keratinocyte Overexpression from PMID: 30772301	SOX2
CCDC74B	SOX2 regulated genes in Keratinocyte Overexpression from PMID: 30772301	SOX2
CCDC74A	SOX2 regulated genes in Keratinocyte Overexpression from PMID: 30772301	SOX2
SFTPC	SOX2 regulated genes in Keratinocyte Overexpression from PMID: 30772301	SOX2
AHSA1	SOX2 regulated genes in Keratinocyte Overexpression from PMID: 30772301	SOX2
RNF145	SOX2 regulated genes in Keratinocyte Overexpression from PMID: 30772301	SOX2
CXCL12	SOX2 regulated genes in Keratinocyte Overexpression from PMID: 30772301	SOX2
SNCAIP	SOX2 regulated genes in Keratinocyte Overexpression from PMID: 30772301	SOX2
TADA1	SOX2 regulated genes in Keratinocyte Overexpression from PMID: 30772301	SOX2
IGSF3	SOX2 regulated genes in Keratinocyte Overexpression from PMID: 30772301	SOX2
CENPO	SOX2 regulated genes in Keratinocyte Overexpression from PMID: 30772301	SOX2
RBM12B	SOX2 regulated genes in Keratinocyte Overexpression from PMID: 30772301	SOX2
APPBP2	SOX2 regulated genes in Keratinocyte Overexpression from PMID: 30772301	SOX2
TMEM186	SOX2 regulated genes in Keratinocyte Overexpression from PMID: 30772301	SOX2
EZR	SOX2 regulated genes in Keratinocyte Overexpression from PMID: 30772301	SOX2
TTC21B	SOX2 regulated genes in Keratinocyte Overexpression from PMID: 30772301	SOX2
FANCD2	SOX2 regulated genes in Keratinocyte Overexpression from PMID: 30772301	SOX2
ACOT1	SOX2 regulated genes in Keratinocyte Overexpression from PMID: 30772301	SOX2
ACOT2	SOX2 regulated genes in Keratinocyte Overexpression from PMID: 30772301	SOX2
SSX2IP	SOX2 regulated genes in Keratinocyte Overexpression from PMID: 30772301	SOX2
TCOF1	SOX2 regulated genes in Keratinocyte Overexpression from PMID: 30772301	SOX2
ZNF205	SOX2 regulated genes in Keratinocyte Overexpression from PMID: 30772301	SOX2
MAFK	SOX2 regulated genes in Keratinocyte Overexpression from PMID: 30772301	SOX2
LIN7B	SOX2 regulated genes in Keratinocyte Overexpression from PMID: 30772301	SOX2
DUSP7	SOX2 regulated genes in Keratinocyte Overexpression from PMID: 30772301	SOX2
NOTCH3	SOX2 regulated genes in Keratinocyte Overexpression from PMID: 30772301	SOX2
CFHR2	SOX2 regulated genes in Keratinocyte Overexpression from PMID: 30772301	SOX2
CFAP36	SOX2 regulated genes in Keratinocyte Overexpression from PMID: 30772301	SOX2

ZNF845	SOX2 regulated genes in Keratinocyte Overexpression from PMID: 30772301	SOX2
CTNND1	SOX2 regulated genes in Keratinocyte Overexpression from PMID: 30772301	SOX2
ZNF777	SOX2 regulated genes in Keratinocyte Overexpression from PMID: 30772301	SOX2
APLP1	SOX2 regulated genes in Keratinocyte Overexpression from PMID: 30772301	SOX2
TMEM231	SOX2 regulated genes in Keratinocyte Overexpression from PMID: 30772301	SOX2
CRTC2	SOX2 regulated genes in Keratinocyte Overexpression from PMID: 30772301	SOX2
FAM8A1	SOX2 regulated genes in Keratinocyte Overexpression from PMID: 30772301	SOX2
TMEM54	SOX2 regulated genes in Keratinocyte Overexpression from PMID: 30772301	SOX2
HNRNPUL1	SOX2 regulated genes in Keratinocyte Overexpression from PMID: 30772301	SOX2
CYB5R2	SOX2 regulated genes in Keratinocyte Overexpression from PMID: 30772301	SOX2
BCL9	SOX2 regulated genes in Keratinocyte Overexpression from PMID: 30772301	SOX2
FAM189A1	SOX2 regulated genes in Keratinocyte Overexpression from PMID: 30772301	SOX2
OSCAR	SOX2 regulated genes in Keratinocyte Overexpression from PMID: 30772301	SOX2
DYNC1I1	SOX2 regulated genes in Keratinocyte Overexpression from PMID: 30772301	SOX2
ZNF286A	SOX2 regulated genes in Keratinocyte Overexpression from PMID: 30772301	SOX2
RHOB	SOX2 regulated genes in Keratinocyte Overexpression from PMID: 30772301	SOX2
MRPL43	SOX2 regulated genes in Keratinocyte Overexpression from PMID: 30772301	SOX2
KMT2D	SOX2 regulated genes in Keratinocyte Overexpression from PMID: 30772301	SOX2
SRRM1	SOX2 regulated genes in Keratinocyte Overexpression from PMID: 30772301	SOX2
SRSF10	SOX2 regulated genes in Keratinocyte Overexpression from PMID: 30772301	SOX2
GPBP1L1	SOX2 regulated genes in Keratinocyte Overexpression from PMID: 30772301	SOX2
ADGRB1	SOX2 regulated genes in Keratinocyte Overexpression from PMID: 30772301	SOX2
PYCR3	SOX2 regulated genes in Keratinocyte Overexpression from PMID: 30772301	SOX2
FAM98A	SOX2 regulated genes in Keratinocyte Overexpression from PMID: 30772301	SOX2
STARD10	SOX2 regulated genes in Keratinocyte Overexpression from PMID: 30772301	SOX2
CSNK1G2	SOX2 regulated genes in Keratinocyte Overexpression from PMID: 30772301	SOX2
TRIP12	SOX2 regulated genes in Keratinocyte Overexpression from PMID: 30772301	SOX2
WWTR1	SOX2 regulated genes in Keratinocyte Overexpression from PMID: 30772301	SOX2
C2CD3	SOX2 regulated genes in Keratinocyte Overexpression from PMID: 30772301	SOX2
SLC25A39	SOX2 regulated genes in Keratinocyte Overexpression from PMID: 30772301	SOX2
NACA	SOX2 regulated genes in Keratinocyte Overexpression from PMID: 30772301	SOX2
MAP2K2	SOX2 regulated genes in Keratinocyte Overexpression from PMID: 30772301	SOX2
WDR6	SOX2 regulated genes in Keratinocyte Overexpression from PMID: 30772301	SOX2
TMEM216	SOX2 regulated genes in Keratinocyte Overexpression from PMID: 30772301	SOX2
XPO1	SOX2 regulated genes in Keratinocyte Overexpression from PMID: 30772301	SOX2
FAM53C	SOX2 regulated genes in Keratinocyte Overexpression from PMID: 30772301	SOX2
SHANK2	SOX2 regulated genes in Keratinocyte Overexpression from PMID: 30772301	SOX2
CEBPZ	SOX2 regulated genes in Keratinocyte Overexpression from PMID: 30772301	SOX2
CNOT1	SOX2 regulated genes in Keratinocyte Overexpression from PMID: 30772301	SOX2
CTF1	SOX2 regulated genes in Keratinocyte Overexpression from PMID: 30772301	SOX2
RPL19	SOX2 regulated genes in Keratinocyte Overexpression from PMID: 30772301	SOX2
C19orf33	SOX2 regulated genes in Keratinocyte Overexpression from PMID: 30772301	SOX2
ELP6	SOX2 regulated genes in Keratinocyte Overexpression from PMID: 30772301	SOX2
NAA38	SOX2 regulated genes in Keratinocyte Overexpression from PMID: 30772301	SOX2
LRRN2	SOX2 regulated genes in Keratinocyte Overexpression from PMID: 30772301	SOX2
SCCPDH	SOX2 regulated genes in Keratinocyte Overexpression from PMID: 30772301	SOX2
ARHGAP33	SOX2 regulated genes in Keratinocyte Overexpression from PMID: 30772301	SOX2
TMEM161B	SOX2 regulated genes in Keratinocyte Overexpression from PMID: 30772301	SOX2
RELL2	SOX2 regulated genes in Keratinocyte Overexpression from PMID: 30772301	SOX2
KCNJ14	SOX2 regulated genes in Keratinocyte Overexpression from PMID: 30772301	SOX2
SPINK8	SOX2 regulated genes in Keratinocyte Overexpression from PMID: 30772301	SOX2
LSM14A	SOX2 regulated genes in Keratinocyte Overexpression from PMID: 30772301	SOX2
METRNL	SOX2 regulated genes in Keratinocyte Overexpression from PMID: 30772301	SOX2
HSD11B2	SOX2 regulated genes in Keratinocyte Overexpression from PMID: 30772301	SOX2
DCAF7	SOX2 regulated genes in Keratinocyte Overexpression from PMID: 30772301	SOX2
ANKRD22	SOX2 regulated genes in Keratinocyte Overexpression from PMID: 30772301	SOX2
ZCRB1	SOX2 regulated genes in Keratinocyte Overexpression from PMID: 30772301	SOX2
ACAN	SOX2 regulated genes in Keratinocyte Overexpression from PMID: 30772301	SOX2
GADD45GIP1	SOX2 regulated genes in Keratinocyte Overexpression from PMID: 30772301	SOX2

NKPD1	SOX2 regulated genes in Keratinocyte Overexpression from PMID: 30772301	SOX2
HOXD8	SOX2 regulated genes in Keratinocyte Overexpression from PMID: 30772301	SOX2
ARHGAP20	SOX2 regulated genes in Keratinocyte Overexpression from PMID: 30772301	SOX2
WDR89	SOX2 regulated genes in Keratinocyte Overexpression from PMID: 30772301	SOX2
SENP2	SOX2 regulated genes in Keratinocyte Overexpression from PMID: 30772301	SOX2
ELMOD3	SOX2 regulated genes in Keratinocyte Overexpression from PMID: 30772301	SOX2
TCERG1	SOX2 regulated genes in Keratinocyte Overexpression from PMID: 30772301	SOX2
CCDC174	SOX2 regulated genes in Keratinocyte Overexpression from PMID: 30772301	SOX2
PRCC	SOX2 regulated genes in Keratinocyte Overexpression from PMID: 30772301	SOX2
DBI	SOX2 regulated genes in Keratinocyte Overexpression from PMID: 30772301	SOX2
NEMF	SOX2 regulated genes in Keratinocyte Overexpression from PMID: 30772301	SOX2
ZNF326	SOX2 regulated genes in Keratinocyte Overexpression from PMID: 30772301	SOX2
ITGB3BP	SOX2 regulated genes in Keratinocyte Overexpression from PMID: 30772301	SOX2
JAGN1	SOX2 regulated genes in Keratinocyte Overexpression from PMID: 30772301	SOX2
SIPA1L2	SOX2 regulated genes in Keratinocyte Overexpression from PMID: 30772301	SOX2
XPO7	SOX2 regulated genes in Keratinocyte Overexpression from PMID: 30772301	SOX2
ELOF1	SOX2 regulated genes in Keratinocyte Overexpression from PMID: 30772301	SOX2
SPG7	SOX2 regulated genes in Keratinocyte Overexpression from PMID: 30772301	SOX2
DIMT1	SOX2 regulated genes in Keratinocyte Overexpression from PMID: 30772301	SOX2
RPS23	SOX2 regulated genes in Keratinocyte Overexpression from PMID: 30772301	SOX2
ARL6IP6	SOX2 regulated genes in Keratinocyte Overexpression from PMID: 30772301	SOX2
WNT4	SOX2 regulated genes in Keratinocyte Overexpression from PMID: 30772301	SOX2
LMBRD2	SOX2 regulated genes in Keratinocyte Overexpression from PMID: 30772301	SOX2
ZNF358	SOX2 regulated genes in Keratinocyte Overexpression from PMID: 30772301	SOX2
ANKRD13C	SOX2 regulated genes in Keratinocyte Overexpression from PMID: 30772301	SOX2
GNL1	SOX2 regulated genes in Keratinocyte Overexpression from PMID: 30772301	SOX2
PTCD3	SOX2 regulated genes in Keratinocyte Overexpression from PMID: 30772301	SOX2
PKP1	SOX2 regulated genes in Keratinocyte Overexpression from PMID: 30772301	SOX2
ZNF579	SOX2 regulated genes in Keratinocyte Overexpression from PMID: 30772301	SOX2
MME	SOX2 regulated genes in Keratinocyte Overexpression from PMID: 30772301	SOX2
PUM2	SOX2 regulated genes in Keratinocyte Overexpression from PMID: 30772301	SOX2
DDX20	SOX2 regulated genes in Keratinocyte Overexpression from PMID: 30772301	SOX2
MLPH	SOX2 regulated genes in Keratinocyte Overexpression from PMID: 30772301	SOX2
KRT20	SOX2 regulated genes in Keratinocyte Overexpression from PMID: 30772301	SOX2
MSTO1	SOX2 regulated genes in Keratinocyte Overexpression from PMID: 30772301	SOX2
DHX30	SOX2 regulated genes in Keratinocyte Overexpression from PMID: 30772301	SOX2
NECAB2	SOX2 regulated genes in Keratinocyte Overexpression from PMID: 30772301	SOX2
RNF126	SOX2 regulated genes in Keratinocyte Overexpression from PMID: 30772301	SOX2
NFYC	SOX2 regulated genes in Keratinocyte Overexpression from PMID: 30772301	SOX2
ALKBH3	SOX2 regulated genes in Keratinocyte Overexpression from PMID: 30772301	SOX2
GUCY2D	SOX2 regulated genes in Keratinocyte Overexpression from PMID: 30772301	SOX2
MARK2	SOX2 regulated genes in Keratinocyte Overexpression from PMID: 30772301	SOX2
SLC25A10	SOX2 regulated genes in Keratinocyte Overexpression from PMID: 30772301	SOX2
RNASET2	SOX2 regulated genes in Keratinocyte Overexpression from PMID: 30772301	SOX2
CEP97	SOX2 regulated genes in Keratinocyte Overexpression from PMID: 30772301	SOX2
RPL24	SOX2 regulated genes in Keratinocyte Overexpression from PMID: 30772301	SOX2
ORC4	SOX2 regulated genes in Keratinocyte Overexpression from PMID: 30772301	SOX2
SNRNP48	SOX2 regulated genes in Keratinocyte Overexpression from PMID: 30772301	SOX2
CYSTM1	SOX2 regulated genes in Keratinocyte Overexpression from PMID: 30772301	SOX2
ZNF623	SOX2 regulated genes in Keratinocyte Overexpression from PMID: 30772301	SOX2
ZNF781	SOX2 regulated genes in Keratinocyte Overexpression from PMID: 30772301	SOX2
ARFIP2	SOX2 regulated genes in Keratinocyte Overexpression from PMID: 30772301	SOX2
HNRNPH1	SOX2 regulated genes in Keratinocyte Overexpression from PMID: 30772301	SOX2
WBP1	SOX2 regulated genes in Keratinocyte Overexpression from PMID: 30772301	SOX2
EML2	SOX2 regulated genes in Keratinocyte Overexpression from PMID: 30772301	SOX2
BRD8	SOX2 regulated genes in Keratinocyte Overexpression from PMID: 30772301	SOX2
TUBA1C	SOX2 regulated genes in Keratinocyte Overexpression from PMID: 30772301	SOX2
CMTM4	SOX2 regulated genes in Keratinocyte Overexpression from PMID: 30772301	SOX2
CD9	SOX2 regulated genes in Keratinocyte Overexpression from PMID: 30772301	SOX2

ZNF672	SOX2 regulated genes in Keratinocyte Overexpression from PMID: 30772301	SOX2
CHTOP	SOX2 regulated genes in Keratinocyte Overexpression from PMID: 30772301	SOX2
TMEM218	SOX2 regulated genes in Keratinocyte Overexpression from PMID: 30772301	SOX2
PEF1	SOX2 regulated genes in Keratinocyte Overexpression from PMID: 30772301	SOX2
COG1	SOX2 regulated genes in Keratinocyte Overexpression from PMID: 30772301	SOX2
IL1F10	SOX2 regulated genes in Keratinocyte Overexpression from PMID: 30772301	SOX2
SAMD4B	SOX2 regulated genes in Keratinocyte Overexpression from PMID: 30772301	SOX2
ERBB2	SOX2 regulated genes in Keratinocyte Overexpression from PMID: 30772301	SOX2
NOM1	SOX2 regulated genes in Keratinocyte Overexpression from PMID: 30772301	SOX2
VAV3	SOX2 regulated genes in Keratinocyte Overexpression from PMID: 30772301	SOX2
DAPL1	SOX2 regulated genes in Keratinocyte Overexpression from PMID: 30772301	SOX2
RHBDD2	SOX2 regulated genes in Keratinocyte Overexpression from PMID: 30772301	SOX2
SH3BP5L	SOX2 regulated genes in Keratinocyte Overexpression from PMID: 30772301	SOX2
TPGS1	SOX2 regulated genes in Keratinocyte Overexpression from PMID: 30772301	SOX2
KRTAP2-3	SOX2 regulated genes in Keratinocyte Overexpression from PMID: 30772301	SOX2
KRTAP4-11	SOX2 regulated genes in Keratinocyte Overexpression from PMID: 30772301	SOX2
KRTAP4-8	SOX2 regulated genes in Keratinocyte Overexpression from PMID: 30772301	SOX2
NACC1	SOX2 regulated genes in Keratinocyte Overexpression from PMID: 30772301	SOX2
RNF26	SOX2 regulated genes in Keratinocyte Overexpression from PMID: 30772301	SOX2
WNT9A	SOX2 regulated genes in Keratinocyte Overexpression from PMID: 30772301	SOX2
HCAR1	SOX2 regulated genes in Keratinocyte Overexpression from PMID: 30772301	SOX2
DHFR2	SOX2 regulated genes in Keratinocyte Overexpression from PMID: 30772301	SOX2
TBP	SOX2 regulated genes in Keratinocyte Overexpression from PMID: 30772301	SOX2
CAMSAP3	SOX2 regulated genes in Keratinocyte Overexpression from PMID: 30772301	SOX2
MDM1	SOX2 regulated genes in Keratinocyte Overexpression from PMID: 30772301	SOX2
CDC42EP4	SOX2 regulated genes in Keratinocyte Overexpression from PMID: 30772301	SOX2
MOB2	SOX2 regulated genes in Keratinocyte Overexpression from PMID: 30772301	SOX2
C5orf22	SOX2 regulated genes in Keratinocyte Overexpression from PMID: 30772301	SOX2
JMJD6	SOX2 regulated genes in Keratinocyte Overexpression from PMID: 30772301	SOX2
ELL	SOX2 regulated genes in Keratinocyte Overexpression from PMID: 30772301	SOX2
RCCD1	SOX2 regulated genes in Keratinocyte Overexpression from PMID: 30772301	SOX2
THOC7	SOX2 regulated genes in Keratinocyte Overexpression from PMID: 30772301	SOX2
UGT1A6	SOX2 regulated genes in Keratinocyte Overexpression from PMID: 30772301	SOX2
MED16	SOX2 regulated genes in Keratinocyte Overexpression from PMID: 30772301	SOX2
VEZT	SOX2 regulated genes in Keratinocyte Overexpression from PMID: 30772301	SOX2
SNRNP25	SOX2 regulated genes in Keratinocyte Overexpression from PMID: 30772301	SOX2
RNF19B	SOX2 regulated genes in Keratinocyte Overexpression from PMID: 30772301	SOX2
DDX49	SOX2 regulated genes in Keratinocyte Overexpression from PMID: 30772301	SOX2
BAZ2B	SOX2 regulated genes in Keratinocyte Overexpression from PMID: 30772301	SOX2
MUTYH	SOX2 regulated genes in Keratinocyte Overexpression from PMID: 30772301	SOX2
DDX55	SOX2 regulated genes in Keratinocyte Overexpression from PMID: 30772301	SOX2
EIF4G1	SOX2 regulated genes in Keratinocyte Overexpression from PMID: 30772301	SOX2
VPS37B	SOX2 regulated genes in Keratinocyte Overexpression from PMID: 30772301	SOX2
ATP13A3	SOX2 regulated genes in Keratinocyte Overexpression from PMID: 30772301	SOX2
RPL26	SOX2 regulated genes in Keratinocyte Overexpression from PMID: 30772301	SOX2
PLK2	SOX2 regulated genes in Keratinocyte Overexpression from PMID: 30772301	SOX2
SPRY4	SOX2 regulated genes in Keratinocyte Overexpression from PMID: 30772301	SOX2
SPATA2L	SOX2 regulated genes in Keratinocyte Overexpression from PMID: 30772301	SOX2
EXOC3L4	SOX2 regulated genes in Keratinocyte Overexpression from PMID: 30772301	SOX2
F2RL1	SOX2 regulated genes in Keratinocyte Overexpression from PMID: 30772301	SOX2
KRT23	SOX2 regulated genes in Keratinocyte Overexpression from PMID: 30772301	SOX2
ULK1	SOX2 regulated genes in Keratinocyte Overexpression from PMID: 30772301	SOX2
C19orf67	SOX2 regulated genes in Keratinocyte Overexpression from PMID: 30772301	SOX2
CCNJ	SOX2 regulated genes in Keratinocyte Overexpression from PMID: 30772301	SOX2
CNTROB	SOX2 regulated genes in Keratinocyte Overexpression from PMID: 30772301	SOX2
UXS1	SOX2 regulated genes in Keratinocyte Overexpression from PMID: 30772301	SOX2
MVK	SOX2 regulated genes in Keratinocyte Overexpression from PMID: 30772301	SOX2
RASAL2	SOX2 regulated genes in Keratinocyte Overexpression from PMID: 30772301	SOX2
STK11	SOX2 regulated genes in Keratinocyte Overexpression from PMID: 30772301	SOX2

RAD17	SOX2 regulated genes in Keratinocyte Overexpression from PMID: 30772301	SOX2
NDUFC2	SOX2 regulated genes in Keratinocyte Overexpression from PMID: 30772301	SOX2
DUOXA1	SOX2 regulated genes in Keratinocyte Overexpression from PMID: 30772301	SOX2
PPP2R2B	SOX2 regulated genes in Keratinocyte Overexpression from PMID: 30772301	SOX2
TNFAIP8L1	SOX2 regulated genes in Keratinocyte Overexpression from PMID: 30772301	SOX2
HDAC4	SOX2 regulated genes in Keratinocyte Overexpression from PMID: 30772301	SOX2
DPP3	SOX2 regulated genes in Keratinocyte Overexpression from PMID: 30772301	SOX2
DHRS13	SOX2 regulated genes in Keratinocyte Overexpression from PMID: 30772301	SOX2
DYNC2LI1	SOX2 regulated genes in Keratinocyte Overexpression from PMID: 30772301	SOX2
ZBTB2	SOX2 regulated genes in Keratinocyte Overexpression from PMID: 30772301	SOX2
EIF1	SOX2 regulated genes in Keratinocyte Overexpression from PMID: 30772301	SOX2
RPF1	SOX2 regulated genes in Keratinocyte Overexpression from PMID: 30772301	SOX2
IPO7	SOX2 regulated genes in Keratinocyte Overexpression from PMID: 30772301	SOX2
RASSF9	SOX2 regulated genes in Keratinocyte Overexpression from PMID: 30772301	SOX2
ZFAND1	SOX2 regulated genes in Keratinocyte Overexpression from PMID: 30772301	SOX2
RABIF	SOX2 regulated genes in Keratinocyte Overexpression from PMID: 30772301	SOX2
MYT1L	SOX2 regulated genes in Keratinocyte Overexpression from PMID: 30772301	SOX2
USP37	SOX2 regulated genes in Keratinocyte Overexpression from PMID: 30772301	SOX2
GPN1	SOX2 regulated genes in Keratinocyte Overexpression from PMID: 30772301	SOX2
MED19	SOX2 regulated genes in Keratinocyte Overexpression from PMID: 30772301	SOX2
ADGRL3	SOX2 regulated genes in Keratinocyte Overexpression from PMID: 30772301	SOX2
STK11IP	SOX2 regulated genes in Keratinocyte Overexpression from PMID: 30772301	SOX2
PNMA1	SOX2 regulated genes in Keratinocyte Overexpression from PMID: 30772301	SOX2
CC2D1B	SOX2 regulated genes in Keratinocyte Overexpression from PMID: 30772301	SOX2
FABP4	SOX2 regulated genes in Keratinocyte Overexpression from PMID: 30772301	SOX2
ABHD14A	SOX2 regulated genes in Keratinocyte Overexpression from PMID: 30772301	SOX2
NR2F6	SOX2 regulated genes in Keratinocyte Overexpression from PMID: 30772301	SOX2
ARMT1	SOX2 regulated genes in Keratinocyte Overexpression from PMID: 30772301	SOX2
WDR33	SOX2 regulated genes in Keratinocyte Overexpression from PMID: 30772301	SOX2
HNRNPA3	SOX2 regulated genes in Keratinocyte Overexpression from PMID: 30772301	SOX2
LRIG1	SOX2 regulated genes in Keratinocyte Overexpression from PMID: 30772301	SOX2
ETV2	SOX2 regulated genes in Keratinocyte Overexpression from PMID: 30772301	SOX2
BNIPL	SOX2 regulated genes in Keratinocyte Overexpression from PMID: 30772301	SOX2
INSIG2	SOX2 regulated genes in Keratinocyte Overexpression from PMID: 30772301	SOX2
CETN3	SOX2 regulated genes in Keratinocyte Overexpression from PMID: 30772301	SOX2
FAM98C	SOX2 regulated genes in Keratinocyte Overexpression from PMID: 30772301	SOX2
HAS3	SOX2 regulated genes in Keratinocyte Overexpression from PMID: 30772301	SOX2
PAN2	SOX2 regulated genes in Keratinocyte Overexpression from PMID: 30772301	SOX2
SPNS2	SOX2 regulated genes in Keratinocyte Overexpression from PMID: 30772301	SOX2
TRIM33	SOX2 regulated genes in Keratinocyte Overexpression from PMID: 30772301	SOX2
SGMS2	SOX2 regulated genes in Keratinocyte Overexpression from PMID: 30772301	SOX2
ZNF771	SOX2 regulated genes in Keratinocyte Overexpression from PMID: 30772301	SOX2
CLP1	SOX2 regulated genes in Keratinocyte Overexpression from PMID: 30772301	SOX2
DCBLD2	SOX2 regulated genes in Keratinocyte Overexpression from PMID: 30772301	SOX2
RRP9	SOX2 regulated genes in Keratinocyte Overexpression from PMID: 30772301	SOX2
OTUD3	SOX2 regulated genes in Keratinocyte Overexpression from PMID: 30772301	SOX2
LRRC8E	SOX2 regulated genes in Keratinocyte Overexpression from PMID: 30772301	SOX2
TONSL	SOX2 regulated genes in Keratinocyte Overexpression from PMID: 30772301	SOX2
NRDC	SOX2 regulated genes in Keratinocyte Overexpression from PMID: 30772301	SOX2
DCP1A	SOX2 regulated genes in Keratinocyte Overexpression from PMID: 30772301	SOX2
ELMO3	SOX2 regulated genes in Keratinocyte Overexpression from PMID: 30772301	SOX2
ZNF12	SOX2 regulated genes in Keratinocyte Overexpression from PMID: 30772301	SOX2
FAM126B	SOX2 regulated genes in Keratinocyte Overexpression from PMID: 30772301	SOX2
EDARADD	SOX2 regulated genes in Keratinocyte Overexpression from PMID: 30772301	SOX2
NABP2	SOX2 regulated genes in Keratinocyte Overexpression from PMID: 30772301	SOX2
TKT	SOX2 regulated genes in Keratinocyte Overexpression from PMID: 30772301	SOX2
MCUR1	SOX2 regulated genes in Keratinocyte Overexpression from PMID: 30772301	SOX2
GALNT3	SOX2 regulated genes in Keratinocyte Overexpression from PMID: 30772301	SOX2
WIF1	SOX2 regulated genes in Keratinocyte Overexpression from PMID: 30772301	SOX2

SGTA	SOX2 regulated genes in Keratinocyte Overexpression from PMID: 30772301	SOX2
DGKE	SOX2 regulated genes in Keratinocyte Overexpression from PMID: 30772301	SOX2
OCLN	SOX2 regulated genes in Keratinocyte Overexpression from PMID: 30772301	SOX2
PDLIM7	SOX2 regulated genes in Keratinocyte Overexpression from PMID: 30772301	SOX2
INHA	SOX2 regulated genes in Keratinocyte Overexpression from PMID: 30772301	SOX2
BOLA2	SOX2 regulated genes in Keratinocyte Overexpression from PMID: 30772301	SOX2
USH1G	SOX2 regulated genes in Keratinocyte Overexpression from PMID: 30772301	SOX2
KLHL18	SOX2 regulated genes in Keratinocyte Overexpression from PMID: 30772301	SOX2
PFDN1	SOX2 regulated genes in Keratinocyte Overexpression from PMID: 30772301	SOX2
DSP	SOX2 regulated genes in Keratinocyte Overexpression from PMID: 30772301	SOX2
TRAF3IP1	SOX2 regulated genes in Keratinocyte Overexpression from PMID: 30772301	SOX2
MRPS12	SOX2 regulated genes in Keratinocyte Overexpression from PMID: 30772301	SOX2
KNL1	SOX2 regulated genes in Keratinocyte Overexpression from PMID: 30772301	SOX2
CACYBP	SOX2 regulated genes in Keratinocyte Overexpression from PMID: 30772301	SOX2
ARIH2	SOX2 regulated genes in Keratinocyte Overexpression from PMID: 30772301	SOX2
POLQ	SOX2 regulated genes in Keratinocyte Overexpression from PMID: 30772301	SOX2
TEN1	SOX2 regulated genes in Keratinocyte Overexpression from PMID: 30772301	SOX2
GTF2E1	SOX2 regulated genes in Keratinocyte Overexpression from PMID: 30772301	SOX2
RAP1GAP	SOX2 regulated genes in Keratinocyte Overexpression from PMID: 30772301	SOX2
TMCO6	SOX2 regulated genes in Keratinocyte Overexpression from PMID: 30772301	SOX2
SPTBN4	SOX2 regulated genes in Keratinocyte Overexpression from PMID: 30772301	SOX2
TMEM237	SOX2 regulated genes in Keratinocyte Overexpression from PMID: 30772301	SOX2
ASF1B	SOX2 regulated genes in Keratinocyte Overexpression from PMID: 30772301	SOX2
SLC8A2	SOX2 regulated genes in Keratinocyte Overexpression from PMID: 30772301	SOX2
SNX11	SOX2 regulated genes in Keratinocyte Overexpression from PMID: 30772301	SOX2
CTU1	SOX2 regulated genes in Keratinocyte Overexpression from PMID: 30772301	SOX2
AKAP8	SOX2 regulated genes in Keratinocyte Overexpression from PMID: 30772301	SOX2
CCNL2	SOX2 regulated genes in Keratinocyte Overexpression from PMID: 30772301	SOX2
SPC24	SOX2 regulated genes in Keratinocyte Overexpression from PMID: 30772301	SOX2
SMIM5	SOX2 regulated genes in Keratinocyte Overexpression from PMID: 30772301	SOX2
GPATCH3	SOX2 regulated genes in Keratinocyte Overexpression from PMID: 30772301	SOX2
RBM4	SOX2 regulated genes in Keratinocyte Overexpression from PMID: 30772301	SOX2
NUF2	SOX2 regulated genes in Keratinocyte Overexpression from PMID: 30772301	SOX2
MRPS14	SOX2 regulated genes in Keratinocyte Overexpression from PMID: 30772301	SOX2
XPOT	SOX2 regulated genes in Keratinocyte Overexpression from PMID: 30772301	SOX2
LZTFL1	SOX2 regulated genes in Keratinocyte Overexpression from PMID: 30772301	SOX2
TMEM221	SOX2 regulated genes in Keratinocyte Overexpression from PMID: 30772301	SOX2
KLK13	SOX2 regulated genes in Keratinocyte Overexpression from PMID: 30772301	SOX2
C7orf25	SOX2 regulated genes in Keratinocyte Overexpression from PMID: 30772301	SOX2
GLMN	SOX2 regulated genes in Keratinocyte Overexpression from PMID: 30772301	SOX2
WDR83	SOX2 regulated genes in Keratinocyte Overexpression from PMID: 30772301	SOX2
LINGO1	SOX2 regulated genes in Keratinocyte Overexpression from PMID: 30772301	SOX2
CNFN	SOX2 regulated genes in Keratinocyte Overexpression from PMID: 30772301	SOX2
CLN8	SOX2 regulated genes in Keratinocyte Overexpression from PMID: 30772301	SOX2
RTBDN	SOX2 regulated genes in Keratinocyte Overexpression from PMID: 30772301	SOX2
ALG8	SOX2 regulated genes in Keratinocyte Overexpression from PMID: 30772301	SOX2
ABCF3	SOX2 regulated genes in Keratinocyte Overexpression from PMID: 30772301	SOX2
DDX23	SOX2 regulated genes in Keratinocyte Overexpression from PMID: 30772301	SOX2
SLC26A9	SOX2 regulated genes in Keratinocyte Overexpression from PMID: 30772301	SOX2
TARBP2	SOX2 regulated genes in Keratinocyte Overexpression from PMID: 30772301	SOX2
FUT4	SOX2 regulated genes in Keratinocyte Overexpression from PMID: 30772301	SOX2
COPZ1	SOX2 regulated genes in Keratinocyte Overexpression from PMID: 30772301	SOX2
ETHE1	SOX2 regulated genes in Keratinocyte Overexpression from PMID: 30772301	SOX2
CNBP	SOX2 regulated genes in Keratinocyte Overexpression from PMID: 30772301	SOX2
TMEM9	SOX2 regulated genes in Keratinocyte Overexpression from PMID: 30772301	SOX2
ZNF233	SOX2 regulated genes in Keratinocyte Overexpression from PMID: 30772301	SOX2
RXRB	SOX2 regulated genes in Keratinocyte Overexpression from PMID: 30772301	SOX2
AKAP9	SOX2 regulated genes in Keratinocyte Overexpression from PMID: 30772301	SOX2
SERTAD4	SOX2 regulated genes in Keratinocyte Overexpression from PMID: 30772301	SOX2

UBALD2	SOX2 regulated genes in Keratinocyte Overexpression from PMID: 30772301	SOX2
DNASE2	SOX2 regulated genes in Keratinocyte Overexpression from PMID: 30772301	SOX2
OMG	SOX2 regulated genes in Keratinocyte Overexpression from PMID: 30772301	SOX2
MAN1A2	SOX2 regulated genes in Keratinocyte Overexpression from PMID: 30772301	SOX2
TMEM80	SOX2 regulated genes in Keratinocyte Overexpression from PMID: 30772301	SOX2
OLA1	SOX2 regulated genes in Keratinocyte Overexpression from PMID: 30772301	SOX2
TRAPPC6A	SOX2 regulated genes in Keratinocyte Overexpression from PMID: 30772301	SOX2
SMIM7	SOX2 regulated genes in Keratinocyte Overexpression from PMID: 30772301	SOX2
TMEM234	SOX2 regulated genes in Keratinocyte Overexpression from PMID: 30772301	SOX2
PCNX3	SOX2 regulated genes in Keratinocyte Overexpression from PMID: 30772301	SOX2
STK36	SOX2 regulated genes in Keratinocyte Overexpression from PMID: 30772301	SOX2
NUMA1	SOX2 regulated genes in Keratinocyte Overexpression from PMID: 30772301	SOX2
FAM72C	SOX2 regulated genes in Keratinocyte Overexpression from PMID: 30772301	SOX2
FAM72A	SOX2 regulated genes in Keratinocyte Overexpression from PMID: 30772301	SOX2
FAM72D	SOX2 regulated genes in Keratinocyte Overexpression from PMID: 30772301	SOX2
FAM72B	SOX2 regulated genes in Keratinocyte Overexpression from PMID: 30772301	SOX2
MCPH1	SOX2 regulated genes in Keratinocyte Overexpression from PMID: 30772301	SOX2
RBM15	SOX2 regulated genes in Keratinocyte Overexpression from PMID: 30772301	SOX2
MYL6	SOX2 regulated genes in Keratinocyte Overexpression from PMID: 30772301	SOX2
GNB5	SOX2 regulated genes in Keratinocyte Overexpression from PMID: 30772301	SOX2
SLC4A1AP	SOX2 regulated genes in Keratinocyte Overexpression from PMID: 30772301	SOX2
KRTCAP3	SOX2 regulated genes in Keratinocyte Overexpression from PMID: 30772301	SOX2
RSRC1	SOX2 regulated genes in Keratinocyte Overexpression from PMID: 30772301	SOX2
SP9	SOX2 regulated genes in Keratinocyte Overexpression from PMID: 30772301	SOX2
ASTE1	SOX2 regulated genes in Keratinocyte Overexpression from PMID: 30772301	SOX2
TTC1	SOX2 regulated genes in Keratinocyte Overexpression from PMID: 30772301	SOX2
FLYWCH1	SOX2 regulated genes in Keratinocyte Overexpression from PMID: 30772301	SOX2
MRPS35	SOX2 regulated genes in Keratinocyte Overexpression from PMID: 30772301	SOX2
CLEC2D	SOX2 regulated genes in Keratinocyte Overexpression from PMID: 30772301	SOX2
GLI2	SOX2 regulated genes in Keratinocyte Overexpression from PMID: 30772301	SOX2
GAS8	SOX2 regulated genes in Keratinocyte Overexpression from PMID: 30772301	SOX2
DPP4	SOX2 regulated genes in Keratinocyte Overexpression from PMID: 30772301	SOX2
ARMC9	SOX2 regulated genes in Keratinocyte Overexpression from PMID: 30772301	SOX2
FKBP8	SOX2 regulated genes in Keratinocyte Overexpression from PMID: 30772301	SOX2
AGER	SOX2 regulated genes in Keratinocyte Overexpression from PMID: 30772301	SOX2
USP36	SOX2 regulated genes in Keratinocyte Overexpression from PMID: 30772301	SOX2
TMEM128	SOX2 regulated genes in Keratinocyte Overexpression from PMID: 30772301	SOX2
KIF21A	SOX2 regulated genes in Keratinocyte Overexpression from PMID: 30772301	SOX2
BRI3BP	SOX2 regulated genes in Keratinocyte Overexpression from PMID: 30772301	SOX2
ADAT3	SOX2 regulated genes in Keratinocyte Overexpression from PMID: 30772301	SOX2
USP54	SOX2 regulated genes in Keratinocyte Overexpression from PMID: 30772301	SOX2
TIPIN	SOX2 regulated genes in Keratinocyte Overexpression from PMID: 30772301	SOX2
RPTN	SOX2 regulated genes in Keratinocyte Overexpression from PMID: 30772301	SOX2
RAB3GAP2	SOX2 regulated genes in Keratinocyte Overexpression from PMID: 30772301	SOX2
ULK4	SOX2 regulated genes in Keratinocyte Overexpression from PMID: 30772301	SOX2
RAB24	SOX2 regulated genes in Keratinocyte Overexpression from PMID: 30772301	SOX2
PFKFB2	SOX2 regulated genes in Keratinocyte Overexpression from PMID: 30772301	SOX2
MARVELD3	SOX2 regulated genes in Keratinocyte Overexpression from PMID: 30772301	SOX2
BAG6	SOX2 regulated genes in Keratinocyte Overexpression from PMID: 30772301	SOX2
DCTPP1	SOX2 regulated genes in Keratinocyte Overexpression from PMID: 30772301	SOX2
UBXN7	SOX2 regulated genes in Keratinocyte Overexpression from PMID: 30772301	SOX2
THADA	SOX2 regulated genes in Keratinocyte Overexpression from PMID: 30772301	SOX2
LTBP4	SOX2 regulated genes in Keratinocyte Overexpression from PMID: 30772301	SOX2
ARHGEF28	SOX2 regulated genes in Keratinocyte Overexpression from PMID: 30772301	SOX2
HNRNPA1	SOX2 regulated genes in Keratinocyte Overexpression from PMID: 30772301	SOX2
DDR1	SOX2 regulated genes in Keratinocyte Overexpression from PMID: 30772301	SOX2
TYW3	SOX2 regulated genes in Keratinocyte Overexpression from PMID: 30772301	SOX2
TOPBP1	SOX2 regulated genes in Keratinocyte Overexpression from PMID: 30772301	SOX2
PRKCZ	SOX2 regulated genes in Keratinocyte Overexpression from PMID: 30772301	SOX2

DEAF1	SOX2 regulated genes in Keratinocyte Overexpression from PMID: 30772301	SOX2
BRD4	SOX2 regulated genes in Keratinocyte Overexpression from PMID: 30772301	SOX2
ATP5F1D	SOX2 regulated genes in Keratinocyte Overexpression from PMID: 30772301	SOX2
MMACHC	SOX2 regulated genes in Keratinocyte Overexpression from PMID: 30772301	SOX2
CCNJL	SOX2 regulated genes in Keratinocyte Overexpression from PMID: 30772301	SOX2
PHF23	SOX2 regulated genes in Keratinocyte Overexpression from PMID: 30772301	SOX2
KIFAP3	SOX2 regulated genes in Keratinocyte Overexpression from PMID: 30772301	SOX2
POLE	SOX2 regulated genes in Keratinocyte Overexpression from PMID: 30772301	SOX2
DDB1	SOX2 regulated genes in Keratinocyte Overexpression from PMID: 30772301	SOX2
CNIH4	SOX2 regulated genes in Keratinocyte Overexpression from PMID: 30772301	SOX2
GNG4	SOX2 regulated genes in Keratinocyte Overexpression from PMID: 30772301	SOX2
GORASP1	SOX2 regulated genes in Keratinocyte Overexpression from PMID: 30772301	SOX2
ZNF316	SOX2 regulated genes in Keratinocyte Overexpression from PMID: 30772301	SOX2
YOD1	SOX2 regulated genes in Keratinocyte Overexpression from PMID: 30772301	SOX2
TTLL4	SOX2 regulated genes in Keratinocyte Overexpression from PMID: 30772301	SOX2
JUNB	SOX2 regulated genes in Keratinocyte Overexpression from PMID: 30772301	SOX2
KIF1B	SOX2 regulated genes in Keratinocyte Overexpression from PMID: 30772301	SOX2
PRPF4B	SOX2 regulated genes in Keratinocyte Overexpression from PMID: 30772301	SOX2
GRXCR2	SOX2 regulated genes in Keratinocyte Overexpression from PMID: 30772301	SOX2
NDC1	SOX2 regulated genes in Keratinocyte Overexpression from PMID: 30772301	SOX2
RBBP5	SOX2 regulated genes in Keratinocyte Overexpression from PMID: 30772301	SOX2
VAMP8	SOX2 regulated genes in Keratinocyte Overexpression from PMID: 30772301	SOX2
KNTC1	SOX2 regulated genes in Keratinocyte Overexpression from PMID: 30772301	SOX2
SOX13	SOX2 regulated genes in Keratinocyte Overexpression from PMID: 30772301	SOX2
TMEM79	SOX2 regulated genes in Keratinocyte Overexpression from PMID: 30772301	SOX2
INTS2	SOX2 regulated genes in Keratinocyte Overexpression from PMID: 30772301	SOX2
RBM34	SOX2 regulated genes in Keratinocyte Overexpression from PMID: 30772301	SOX2
PSMG3	SOX2 regulated genes in Keratinocyte Overexpression from PMID: 30772301	SOX2
NMNAT2	SOX2 regulated genes in Keratinocyte Overexpression from PMID: 30772301	SOX2
FAM171A2	SOX2 regulated genes in Keratinocyte Overexpression from PMID: 30772301	SOX2
TEX38	SOX2 regulated genes in Keratinocyte Overexpression from PMID: 30772301	SOX2
TTC36	SOX2 regulated genes in Keratinocyte Overexpression from PMID: 30772301	SOX2
CELF1	SOX2 regulated genes in Keratinocyte Overexpression from PMID: 30772301	SOX2
MRPL9	SOX2 regulated genes in Keratinocyte Overexpression from PMID: 30772301	SOX2
CERS3	SOX2 regulated genes in Keratinocyte Overexpression from PMID: 30772301	SOX2
CIDEB	SOX2 regulated genes in Keratinocyte Overexpression from PMID: 30772301	SOX2
METTL2A	SOX2 regulated genes in Keratinocyte Overexpression from PMID: 30772301	SOX2
IL11RA	SOX2 regulated genes in Keratinocyte Overexpression from PMID: 30772301	SOX2
BCL7C	SOX2 regulated genes in Keratinocyte Overexpression from PMID: 30772301	SOX2
AZIN1	SOX2 regulated genes in Keratinocyte Overexpression from PMID: 30772301	SOX2
DNAJC9	SOX2 regulated genes in Keratinocyte Overexpression from PMID: 30772301	SOX2
IMPA1	SOX2 regulated genes in Keratinocyte Overexpression from PMID: 30772301	SOX2
PRRC2C	SOX2 regulated genes in Keratinocyte Overexpression from PMID: 30772301	SOX2
TXNDC9	SOX2 regulated genes in Keratinocyte Overexpression from PMID: 30772301	SOX2
ODC1	SOX2 regulated genes in Keratinocyte Overexpression from PMID: 30772301	SOX2
ZNF142	SOX2 regulated genes in Keratinocyte Overexpression from PMID: 30772301	SOX2
FOXRED1	SOX2 regulated genes in Keratinocyte Overexpression from PMID: 30772301	SOX2
YEATS2	SOX2 regulated genes in Keratinocyte Overexpression from PMID: 30772301	SOX2
DEF8	SOX2 regulated genes in Keratinocyte Overexpression from PMID: 30772301	SOX2
TRMT10C	SOX2 regulated genes in Keratinocyte Overexpression from PMID: 30772301	SOX2
EFCAB12	SOX2 regulated genes in Keratinocyte Overexpression from PMID: 30772301	SOX2
PSRC1	SOX2 regulated genes in Keratinocyte Overexpression from PMID: 30772301	SOX2
IRF3	SOX2 regulated genes in Keratinocyte Overexpression from PMID: 30772301	SOX2
CSNK1A1	SOX2 regulated genes in Keratinocyte Overexpression from PMID: 30772301	SOX2
KANSL1	SOX2 regulated genes in Keratinocyte Overexpression from PMID: 30772301	SOX2
BDH1	SOX2 regulated genes in Keratinocyte Overexpression from PMID: 30772301	SOX2
TIA1	SOX2 regulated genes in Keratinocyte Overexpression from PMID: 30772301	SOX2
C16orf72	SOX2 regulated genes in Keratinocyte Overexpression from PMID: 30772301	SOX2
DARS2	SOX2 regulated genes in Keratinocyte Overexpression from PMID: 30772301	SOX2

PANX1	SOX2 regulated genes in Keratinocyte Overexpression from PMID: 30772301	SOX2
IPO11	SOX2 regulated genes in Keratinocyte Overexpression from PMID: 30772301	SOX2
PLEKHH2	SOX2 regulated genes in Keratinocyte Overexpression from PMID: 30772301	SOX2
SMG8	SOX2 regulated genes in Keratinocyte Overexpression from PMID: 30772301	SOX2
HMCES	SOX2 regulated genes in Keratinocyte Overexpression from PMID: 30772301	SOX2
C1orf56	SOX2 regulated genes in Keratinocyte Overexpression from PMID: 30772301	SOX2
CYB561D1	SOX2 regulated genes in Keratinocyte Overexpression from PMID: 30772301	SOX2
CBX5	SOX2 regulated genes in Keratinocyte Overexpression from PMID: 30772301	SOX2
KLK10	SOX2 regulated genes in Keratinocyte Overexpression from PMID: 30772301	SOX2
SS18L2	SOX2 regulated genes in Keratinocyte Overexpression from PMID: 30772301	SOX2
RNF40	SOX2 regulated genes in Keratinocyte Overexpression from PMID: 30772301	SOX2
CLPP	SOX2 regulated genes in Keratinocyte Overexpression from PMID: 30772301	SOX2
ZNF608	SOX2 regulated genes in Keratinocyte Overexpression from PMID: 30772301	SOX2
MYO18A	SOX2 regulated genes in Keratinocyte Overexpression from PMID: 30772301	SOX2
HOXD1	SOX2 regulated genes in Keratinocyte Overexpression from PMID: 30772301	SOX2
SKA2	SOX2 regulated genes in Keratinocyte Overexpression from PMID: 30772301	SOX2
ATF7IP2	SOX2 regulated genes in Keratinocyte Overexpression from PMID: 30772301	SOX2
AMH	SOX2 regulated genes in Keratinocyte Overexpression from PMID: 30772301	SOX2
MVB12A	SOX2 regulated genes in Keratinocyte Overexpression from PMID: 30772301	SOX2
METTL16	SOX2 regulated genes in Keratinocyte Overexpression from PMID: 30772301	SOX2
BUB1	SOX2 regulated genes in Keratinocyte Overexpression from PMID: 30772301	SOX2
HDAC5	SOX2 regulated genes in Keratinocyte Overexpression from PMID: 30772301	SOX2
DENND2C	SOX2 regulated genes in Keratinocyte Overexpression from PMID: 30772301	SOX2
ATR	SOX2 regulated genes in Keratinocyte Overexpression from PMID: 30772301	SOX2
TRAPPC4	SOX2 regulated genes in Keratinocyte Overexpression from PMID: 30772301	SOX2
DDX6	SOX2 regulated genes in Keratinocyte Overexpression from PMID: 30772301	SOX2
PSD3	SOX2 regulated genes in Keratinocyte Overexpression from PMID: 30772301	SOX2
PLBD1	SOX2 regulated genes in Keratinocyte Overexpression from PMID: 30772301	SOX2
CCDC97	SOX2 regulated genes in Keratinocyte Overexpression from PMID: 30772301	SOX2
KIAA0825	SOX2 regulated genes in Keratinocyte Overexpression from PMID: 30772301	SOX2
TSEN2	SOX2 regulated genes in Keratinocyte Overexpression from PMID: 30772301	SOX2
KLF1	SOX2 regulated genes in Keratinocyte Overexpression from PMID: 30772301	SOX2
COL11A2	SOX2 regulated genes in Keratinocyte Overexpression from PMID: 30772301	SOX2
TDRD1	SOX2 regulated genes in Keratinocyte Overexpression from PMID: 30772301	SOX2
IGSF9	SOX2 regulated genes in Keratinocyte Overexpression from PMID: 30772301	SOX2
EPS8L2	SOX2 regulated genes in Keratinocyte Overexpression from PMID: 30772301	SOX2
TERF1	SOX2 regulated genes in Keratinocyte Overexpression from PMID: 30772301	SOX2
MXD3	SOX2 regulated genes in Keratinocyte Overexpression from PMID: 30772301	SOX2
GABPB1	SOX2 regulated genes in Keratinocyte Overexpression from PMID: 30772301	SOX2
WWP2	SOX2 regulated genes in Keratinocyte Overexpression from PMID: 30772301	SOX2
CCAR2	SOX2 regulated genes in Keratinocyte Overexpression from PMID: 30772301	SOX2
SH3TC2	SOX2 regulated genes in Keratinocyte Overexpression from PMID: 30772301	SOX2
PRR22	SOX2 regulated genes in Keratinocyte Overexpression from PMID: 30772301	SOX2
C1orf52	SOX2 regulated genes in Keratinocyte Overexpression from PMID: 30772301	SOX2
SH3GL1	SOX2 regulated genes in Keratinocyte Overexpression from PMID: 30772301	SOX2
DALRD3	SOX2 regulated genes in Keratinocyte Overexpression from PMID: 30772301	SOX2
NDUFAF3	SOX2 regulated genes in Keratinocyte Overexpression from PMID: 30772301	SOX2
NOSIP	SOX2 regulated genes in Keratinocyte Overexpression from PMID: 30772301	SOX2
CREB3L4	SOX2 regulated genes in Keratinocyte Overexpression from PMID: 30772301	SOX2
TRPV1	SOX2 regulated genes in Keratinocyte Overexpression from PMID: 30772301	SOX2
HYOU1	SOX2 regulated genes in Keratinocyte Overexpression from PMID: 30772301	SOX2
PSPN	SOX2 regulated genes in Keratinocyte Overexpression from PMID: 30772301	SOX2
SLC35E2B	SOX2 regulated genes in Keratinocyte Overexpression from PMID: 30772301	SOX2
TMEM198	SOX2 regulated genes in Keratinocyte Overexpression from PMID: 30772301	SOX2
RPL35A	SOX2 regulated genes in Keratinocyte Overexpression from PMID: 30772301	SOX2
LSM6	SOX2 regulated genes in Keratinocyte Overexpression from PMID: 30772301	SOX2
TYW5	SOX2 regulated genes in Keratinocyte Overexpression from PMID: 30772301	SOX2
NFRKB	SOX2 regulated genes in Keratinocyte Overexpression from PMID: 30772301	SOX2
ZC3H10	SOX2 regulated genes in Keratinocyte Overexpression from PMID: 30772301	SOX2

RCC1L	SOX2 regulated genes in Keratinocyte Overexpression from PMID: 30772301	SOX2
AHCTF1	SOX2 regulated genes in Keratinocyte Overexpression from PMID: 30772301	SOX2
ENGASE	SOX2 regulated genes in Keratinocyte Overexpression from PMID: 30772301	SOX2
PSMD4	SOX2 regulated genes in Keratinocyte Overexpression from PMID: 30772301	SOX2
SMG7	SOX2 regulated genes in Keratinocyte Overexpression from PMID: 30772301	SOX2
CDK10	SOX2 regulated genes in Keratinocyte Overexpression from PMID: 30772301	SOX2
KLHL21	SOX2 regulated genes in Keratinocyte Overexpression from PMID: 30772301	SOX2
EVI5L	SOX2 regulated genes in Keratinocyte Overexpression from PMID: 30772301	SOX2
INTS11	SOX2 regulated genes in Keratinocyte Overexpression from PMID: 30772301	SOX2
SUPT6H	SOX2 regulated genes in Keratinocyte Overexpression from PMID: 30772301	SOX2
FZD7	SOX2 regulated genes in Keratinocyte Overexpression from PMID: 30772301	SOX2
SPAST	SOX2 regulated genes in Keratinocyte Overexpression from PMID: 30772301	SOX2
APOLD1	SOX2 regulated genes in Keratinocyte Overexpression from PMID: 30772301	SOX2
RAPGEFL1	SOX2 regulated genes in Keratinocyte Overexpression from PMID: 30772301	SOX2
RNFT2	SOX2 regulated genes in Keratinocyte Overexpression from PMID: 30772301	SOX2
ADGRL1	SOX2 regulated genes in Keratinocyte Overexpression from PMID: 30772301	SOX2
MOB4	SOX2 regulated genes in Keratinocyte Overexpression from PMID: 30772301	SOX2
S100A6	SOX2 regulated genes in Keratinocyte Overexpression from PMID: 30772301	SOX2
SCYL3	SOX2 regulated genes in Keratinocyte Overexpression from PMID: 30772301	SOX2
RAPGEF2	SOX2 regulated genes in Keratinocyte Overexpression from PMID: 30772301	SOX2
STAMBP	SOX2 regulated genes in Keratinocyte Overexpression from PMID: 30772301	SOX2
LSR	SOX2 regulated genes in Keratinocyte Overexpression from PMID: 30772301	SOX2
FBLN7	SOX2 regulated genes in Keratinocyte Overexpression from PMID: 30772301	SOX2
ATIC	SOX2 regulated genes in Keratinocyte Overexpression from PMID: 30772301	SOX2
CARF	SOX2 regulated genes in Keratinocyte Overexpression from PMID: 30772301	SOX2
NHSL1	SOX2 regulated genes in Keratinocyte Overexpression from PMID: 30772301	SOX2
CSRNP2	SOX2 regulated genes in Keratinocyte Overexpression from PMID: 30772301	SOX2
CCND1	SOX2 regulated genes in Keratinocyte Overexpression from PMID: 30772301	SOX2
UBE2G1	SOX2 regulated genes in Keratinocyte Overexpression from PMID: 30772301	SOX2
CDKN2D	SOX2 regulated genes in Keratinocyte Overexpression from PMID: 30772301	SOX2
WDR46	SOX2 regulated genes in Keratinocyte Overexpression from PMID: 30772301	SOX2
SETD1B	SOX2 regulated genes in Keratinocyte Overexpression from PMID: 30772301	SOX2
PUM1	SOX2 regulated genes in Keratinocyte Overexpression from PMID: 30772301	SOX2
NDUFV1	SOX2 regulated genes in Keratinocyte Overexpression from PMID: 30772301	SOX2
RABGGTB	SOX2 regulated genes in Keratinocyte Overexpression from PMID: 30772301	SOX2
SLC35A1	SOX2 regulated genes in Keratinocyte Overexpression from PMID: 30772301	SOX2
CSPP1	SOX2 regulated genes in Keratinocyte Overexpression from PMID: 30772301	SOX2
COMMD1	SOX2 regulated genes in Keratinocyte Overexpression from PMID: 30772301	SOX2
LYPD3	SOX2 regulated genes in Keratinocyte Overexpression from PMID: 30772301	SOX2
ST3GAL5	SOX2 regulated genes in Keratinocyte Overexpression from PMID: 30772301	SOX2
VPS54	SOX2 regulated genes in Keratinocyte Overexpression from PMID: 30772301	SOX2
CTTNBP2	SOX2 regulated genes in Keratinocyte Overexpression from PMID: 30772301	SOX2
MAP2K7	SOX2 regulated genes in Keratinocyte Overexpression from PMID: 30772301	SOX2
MYO19	SOX2 regulated genes in Keratinocyte Overexpression from PMID: 30772301	SOX2
FMO4	SOX2 regulated genes in Keratinocyte Overexpression from PMID: 30772301	SOX2
VEPH1	SOX2 regulated genes in Keratinocyte Overexpression from PMID: 30772301	SOX2
PPL	SOX2 regulated genes in Keratinocyte Overexpression from PMID: 30772301	SOX2
EFHD1	SOX2 regulated genes in Keratinocyte Overexpression from PMID: 30772301	SOX2
DYNC2H1	SOX2 regulated genes in Keratinocyte Overexpression from PMID: 30772301	SOX2
SNRNP70	SOX2 regulated genes in Keratinocyte Overexpression from PMID: 30772301	SOX2
KDF1	SOX2 regulated genes in Keratinocyte Overexpression from PMID: 30772301	SOX2
GLIS1	SOX2 regulated genes in Keratinocyte Overexpression from PMID: 30772301	SOX2
ERC2	SOX2 regulated genes in Keratinocyte Overexpression from PMID: 30772301	SOX2
LRRC40	SOX2 regulated genes in Keratinocyte Overexpression from PMID: 30772301	SOX2
TFDP2	SOX2 regulated genes in Keratinocyte Overexpression from PMID: 30772301	SOX2
NCDN	SOX2 regulated genes in Keratinocyte Overexpression from PMID: 30772301	SOX2
SLC35A4	SOX2 regulated genes in Keratinocyte Overexpression from PMID: 30772301	SOX2
MRPL35	SOX2 regulated genes in Keratinocyte Overexpression from PMID: 30772301	SOX2
HR	KLF5 regulated genes from PMID 34217701	KLF5

ITGB4	KLF5 regulated genes from PMID 34217701	KLF5
GLI2	KLF5 regulated genes from PMID 34217701	KLF5
HERPUD2	KLF5 regulated genes from PMID 34217701	KLF5
PTPRU	KLF5 regulated genes from PMID 34217701	KLF5
DPYSL2	KLF5 regulated genes from PMID 34217701	KLF5
XYLT1	KLF5 regulated genes from PMID 34217701	KLF5
TRAK1	KLF5 regulated genes from PMID 34217701	KLF5
DTX4	KLF5 regulated genes from PMID 34217701	KLF5
SEC62	KLF5 regulated genes from PMID 34217701	KLF5
NEDD9	KLF5 regulated genes from PMID 34217701	KLF5
PCDH1	KLF5 regulated genes from PMID 34217701	KLF5
ZNF385A	KLF5 regulated genes from PMID 34217701	KLF5
CD55	KLF5 regulated genes from PMID 34217701	KLF5
RUNX1	KLF5 regulated genes from PMID 34217701	KLF5
TMPRSS5	KLF5 regulated genes from PMID 34217701	KLF5
LAMA3	KLF5 regulated genes from PMID 34217701	KLF5
MICALL1	KLF5 regulated genes from PMID 34217701	KLF5
PHACTR3	KLF5 regulated genes from PMID 34217701	KLF5
EMP1	KLF5 regulated genes from PMID 34217701	KLF5
MYH14	KLF5 regulated genes from PMID 34217701	KLF5
PI3	KLF5 regulated genes from PMID 34217701	KLF5
STEAP4	KLF5 regulated genes from PMID 34217701	KLF5
EPHB3	KLF5 regulated genes from PMID 34217701	KLF5
FAM129A	KLF5 regulated genes from PMID 34217701	KLF5
TRIM2	KLF5 regulated genes from PMID 34217701	KLF5
PKP1	KLF5 regulated genes from PMID 34217701	KLF5
AHCYL2	KLF5 regulated genes from PMID 34217701	KLF5
`MARCH3	KLF5 regulated genes from PMID 34217701	KLF5
TRPV4	KLF5 regulated genes from PMID 34217701	KLF5
RAPGEFL1	KLF5 regulated genes from PMID 34217701	KLF5
WNT7A	KLF5 regulated genes from PMID 34217701	KLF5
DZIP1	KLF5 regulated genes from PMID 34217701	KLF5
SEMA3B	KLF5 regulated genes from PMID 34217701	KLF5
PLAC9	KLF5 regulated genes from PMID 34217701	KLF5
ROS1	KLF5 regulated genes from PMID 34217701	KLF5
CACNG4	KLF5 regulated genes from PMID 34217701	KLF5
ALPP	KLF5 regulated genes from PMID 34217701	KLF5
AIM1L	KLF5 regulated genes from PMID 34217701	KLF5
AHNAK2	KLF5 regulated genes from PMID 34217701	KLF5
GPRC5A	KLF5 regulated genes from PMID 34217701	KLF5
ALS2CL	KLF5 regulated genes from PMID 34217701	KLF5
ZNF860	KLF5 regulated genes from PMID 34217701	KLF5
ARHGEF37	KLF5 regulated genes from PMID 34217701	KLF5
IGFBP2	KLF5 regulated genes from PMID 34217701	KLF5
PMAIP1	KLF5 regulated genes from PMID 34217701	KLF5
MYEOV	KLF5 regulated genes from PMID 34217701	KLF5
MUC1	KLF5 regulated genes from PMID 34217701	KLF5
MISP	KLF5 regulated genes from PMID 34217701	KLF5
TSPAN2	KLF5 regulated genes from PMID 34217701	KLF5
SERTAD4-AS1	KLF5 regulated genes from PMID 34217701	KLF5
ADD3	KLF5 regulated genes from PMID 34217701	KLF5
CMKLR1	KLF5 regulated genes from PMID 34217701	KLF5
PIK3C2B	KLF5 regulated genes from PMID 34217701	KLF5
VILL	KLF5 regulated genes from PMID 34217701	KLF5
ID1	KLF5 regulated genes from PMID 34217701	KLF5
C10orf54	KLF5 regulated genes from PMID 34217701	KLF5
PHLDA3	KLF5 regulated genes from PMID 34217701	KLF5
SRMS	KLF5 regulated genes from PMID 34217701	KLF5
KRT6A	KLF5 regulated genes from PMID 34217701	KLF5

MECOM	KLF5 regulated genes from PMID 34217701	KLF5
TLR5	KLF5 regulated genes from PMID 34217701	KLF5
FAM83A	KLF5 regulated genes from PMID 34217701	KLF5
ANO1	KLF5 regulated genes from PMID 34217701	KLF5

Supplemental Table 6. Module 7 genes known to be regulated by SOX2 and/or KLF5.

gene	source	regulator
ANO1	KLF5 regulated genes from PMID 34217701	KLF5
APOL1	SOX2 regulated genes in Wounding from PMID: 30772301	SOX2
APOL1	Enrichr CHEA_2022: SOX2 20726797 ChIP-Seq SW620 Human	SOX2
APOL1	SOX2 regulated genes in Keratinocyte Overexpression from PMID: 30772301	SOX2
ATP8B1	SOX2-KLF5 coregulated genes from PMID: 33972779	SOX2 + KLF5
BCL2L15	SOX2-KLF5 coregulated genes from PMID: 33972779	SOX2 + KLF5
CA2	Enrichr CHEA_2022: SOX2 20726797 ChIP-Seq SW620 Human	SOX2
CA2	SOX2 regulated genes in Wounding from PMID: 30772301	SOX2
CAMK1D	Enrichr TF Perturbations followed by expression: KLF5 20726797 ChIP-Seq SW620 Human	KLF5
CDH3	SOX2 regulated genes in Wounding from PMID: 30772301	SOX2
CLDN1	SOX2-KLF5 coregulated genes from PMID: 33972779	SOX2 + KLF5
CLDN1	SOX2 regulated genes in Keratinocyte Overexpression from PMID: 30772301	SOX2
CLDN1	SOX2 regulated genes in Wounding from PMID: 30772301	SOX2
CLDN1	Enrichr CHEA_2022: SOX2 20726797 ChIP-Seq SW620 Human	SOX2
CYP2S1	SOX2 regulated genes in Wounding from PMID: 30772301	SOX2
DPYD	Enrichr CHEA_2022: SOX2 20726797 ChIP-Seq SW620 Human	SOX2
DSG3	SOX2-KLF5 coregulated genes from PMID: 33972779	SOX2 + KLF5
DSG3	Enrichr TF Perturbations followed by expression: KLF5 20726797 ChIP-Seq SW620 Human	KLF5
DSG3	SOX2 regulated genes in Wounding from PMID: 30772301	SOX2
DSG3	SOX2 regulated genes in Keratinocyte Overexpression from PMID: 30772301	SOX2
DSP	SOX2 regulated genes in Keratinocyte Overexpression from PMID: 30772301	SOX2
DSP	SOX2-KLF5 coregulated genes from PMID: 33972779	SOX2 + KLF5
DSP	Enrichr CHEA_2022: SOX2 20726797 ChIP-Seq SW620 Human	SOX2
DSP	SOX2 regulated genes in Wounding from PMID: 30772301	SOX2
DUOX1	SOX2 regulated genes in Wounding from PMID: 30772301	SOX2
EPPK1	Enrichr CHEA_2022: SOX2 20726797 ChIP-Seq SW620 Human	SOX2
EPPK1	SOX2 regulated genes in Wounding from PMID: 30772301	SOX2
GALNT5	Enrichr TF Perturbations followed by expression: KLF5 20726797 ChIP-Seq SW620 Human	KLF5
GALNT5	Enrichr CHEA_2022: SOX2 20726797 ChIP-Seq SW620 Human	SOX2
HAS3	SOX2 regulated genes in Wounding from PMID: 30772301	SOX2
HAS3	Enrichr CHEA_2022: SOX2 20726797 ChIP-Seq SW620 Human	SOX2
HAS3	SOX2 regulated genes in Keratinocyte Overexpression from PMID: 30772301	SOX2
HPGD	Enrichr CHEA_2022: SOX2 20726797 ChIP-Seq SW620 Human	SOX2
HPGD	SOX2 regulated genes from PMID: 20726797	SOX2
KCNJ2	SOX2-KLF5 coregulated genes from PMID: 33972779	SOX2 + KLF5
MPP7	SOX2 regulated genes in Wounding from PMID: 30772301	SOX2
MX1	Enrichr CHEA_2022: SOX2 20726797 ChIP-Seq SW620 Human	SOX2
MYH9	SOX2-KLF5 coregulated genes from PMID: 33972779	SOX2 + KLF5
MYH9	SOX2 regulated genes in Keratinocyte Overexpression from PMID: 30772301	SOX2
MYH9	SOX2 regulated genes in Wounding from PMID: 30772301	SOX2
NEFL	Enrichr TF Perturbations followed by expression: KLF5 20726797 ChIP-Seq SW620 Human	KLF5
PDZK1IP1	Enrichr CHEA_2022: SOX2 20726797 ChIP-Seq SW620 Human	SOX2
PHLDB2	SOX2 regulated genes in Wounding from PMID: 30772301	SOX2
PHLDB2	Enrichr CHEA_2022: SOX2 20726797 ChIP-Seq SW620 Human	SOX2
PHLDB2	SOX2 regulated genes in Keratinocyte Overexpression from PMID: 30772301	SOX2
SERPINB3	Enrichr CHEA_2022: SOX2 20726797 ChIP-Seq SW620 Human	SOX2
SFRP1	SOX2 regulated genes in Keratinocyte Overexpression from PMID: 30772301	SOX2
SFRP1	SOX2 regulated genes in Wounding from PMID: 30772301	SOX2
SGK1	SOX2 regulated genes in Wounding from PMID: 30772301	SOX2
SGK1	SOX2-KLF5 coregulated genes from PMID: 33972779	SOX2 + KLF5
SH3RF2	Enrichr TF Perturbations followed by expression: KLF5 20726797 ChIP-Seq SW620 Human	KLF5
SH3RF2	SOX2 regulated genes in Keratinocyte Overexpression from PMID: 30772301	SOX2
TFPI	Enrichr CHEA_2022: SOX2 20726797 ChIP-Seq SW620 Human	SOX2
TNFAIP6	SOX2 regulated genes in Wounding from PMID: 30772301	SOX2

Supplemental Table 7. Gene expression profile of EoE DEGs known to be regulated by SOX2 and/or KLF5.

gene	Log2FC	Pval	FDR_Pval	pct.EoE_Biopsy	pct.Healthy_Control	Top_Changed_Compartment	Regulators
TNFAIP6	14.03276603	0.000195546	0.010489143	26.30969971	0.008333034	Suprabasal	SOX2
NRXN1	8.481843776	0.000599264	0.010733065	5.067165747	0.005928385	Suprabasal	SOX2 + KLF5
DPP4	7.164647328	0.004291437	0.022284502	7.110944705	0.042029564	Suprabasal	SOX2
TRPM6	6.351720177	0.000545165	0.007274975	12.68360569	0.06651167	Superficial	SOX2
LURAP1L	6.213447206	0.000715353	0.007615337	18.34363791	0.132134256	Superficial	SOX2
ANO1	5.903669769	0.000196466	0.007176147	57.68957389	1.889071736	Superficial	KLF5
GLDC	5.812522131	9.15E-05	0.010489143	17.66430308	0.256528795	Suprabasal	KLF5
MYRFL	5.249646268	0.000375835	0.007176147	6.520629319	0.195081078	Superficial	SOX2
NEFL	5.137160698	0.000504805	0.007178523	23.20878673	1.038348856	Superficial	KLF5
RPTN	4.987397171	9.26E-05	0.007176147	9.402714391	0.681967049	Superficial	SOX2
NFATC2	4.981012295	0.000989444	0.008042817	15.46639165	0.386585977	Superficial	SOX2 + KLF5
LBH	4.82051517	0.000506425	0.010489143	15.18280651	0.367077206	Suprabasal	SOX2
ADAM28	4.740686733	0.003303641	0.024854712	5.530658274	0.161625895	Basal	KLF5
KCNJ2	4.535506071	0.000196466	0.010489143	42.41879274	3.237283542	Suprabasal	SOX2 + KLF5
GABRP	4.346532147	5.55E-05	0.012455402	27.82034888	2.002158343	Basal	SOX2, KLF5
SFRP1	4.336773754	0.000153669	0.010489143	44.6669391	2.593848432	Suprabasal	SOX2
EDAR	4.248568858	0.000401828	0.007176147	19.44942997	1.08545527	Superficial	SOX2
EPPK1	4.143182566	0.006166656	0.027303057	34.09283879	3.257315631	Suprabasal	SOX2
IFFO2	4.134503394	0.000250281	0.007176147	59.91842482	9.317539544	Superficial	SOX2
CD200R1	4.133533272	0.000506425	0.007178523	27.369393	0.983226601	Superficial	SOX2
APOL1	4.043383815	0.001232925	0.008679903	40.60842821	3.499022944	Superficial	SOX2
BMPR1B	3.971683288	0.00591569	0.032981404	5.098860854	0.282051825	Basal	SOX2
IFI35	3.965447773	0.001232925	0.008679903	48.98584135	4.252996719	Superficial	SOX2
UPK1B	3.886272489	0.000119764	0.007176147	30.00643469	3.383373143	Superficial	KLF5, SOX2
CFB	3.852144876	0.000401828	0.007176147	33.99028752	3.320047752	Superficial	SOX2
LOXL4	3.819802832	0.000992312	0.014320968	17.36548658	1.18068853	Basal	SOX2
RUNX2	3.769129479	0.000504805	0.007178523	13.80876203	0.842100666	Superficial	SOX2, SOX2 + KLF5
CA2	3.736257496	7.20E-05	0.010489143	56.63468793	13.10997844	Suprabasal	SOX2
SLC28A3	3.717054301	0.000635975	0.007274975	22.8202256	2.11699801	Superficial	SOX2
LYPD6B	3.712117954	0.004211401	0.016013117	23.48505282	1.455960635	Superficial	SOX2 + KLF5, SOX2
SGK1	3.680085691	0.000153669	0.007176147	61.49330151	14.372983	Superficial	SOX2 + KLF5, SOX2
SERPINB3	3.601168438	7.20E-05	0.012455402	61.04787159	17.17234047	Basal	SOX2
ST8SIA6	3.586099976	0.000634007	0.007274975	9.78899944	0.441575518	Superficial	SOX2 + KLF5, SOX2
CCDC68	3.570963328	0.000506425	0.007178523	25.08739932	1.949503374	Superficial	SOX2
CMYA5	3.552817397	0.001526458	0.009387421	11.51192042	0.649201685	Superficial	SOX2 + KLF5
PHLDB2	3.526366051	0.000795824	0.010838271	19.35094986	1.167949601	Suprabasal	SOX2
CYP2S1	3.520993063	0.000992312	0.008042817	38.06773576	4.106293048	Superficial	SOX2
HAS3	3.515910568	0.000635975	0.007274975	31.49524154	3.578664882	Superficial	SOX2
PCSK6	3.498236414	5.55E-05	0.012455402	10.72980733	1.09770805	Basal	SOX2
NFE2L3	3.298054351	0.000250281	0.007176147	27.78456634	3.028185524	Superficial	SOX2
BCL2L15	3.169348667	0.000401828	0.010489143	52.77173551	9.09037378	Suprabasal	SOX2 + KLF5
UPK3BL2	3.151048298	0.0033823	0.014108599	11.77819237	0.999182365	Superficial	SOX2
CADM4	3.111929462	0.000401828	0.007176147	34.66563546	5.040451809	Superficial	SOX2
PAPSS2	3.082313982	0.002315114	0.020052792	14.29165028	1.259098065	Basal	SOX2
MUC4	3.064276121	0.001883197	0.018415354	9.945660526	1.283194417	Basal	KLF5
NAV1	3.01803895	0.000795824	0.007615337	24.20119131	3.215183485	Superficial	SOX2
TNS4	2.993108132	0.001232925	0.008679903	32.53389208	3.314587495	Superficial	SOX2, SOX2 + KLF5
APOL4	2.985249452	0.001526458	0.009387421	13.88024072	1.439759571	Superficial	SOX2
FA2H	2.981191994	7.20E-05	0.007176147	27.14904728	2.928368931	Superficial	KLF5
GALNT4	2.956378661	0.000963884	0.008042817	31.11337918	3.725274822	Superficial	SOX2, KLF5
GCNT4	2.929654443	0.000196466	0.007176147	10.75329782	1.615330968	Superficial	SOX2
KLK1	2.807174652	0.005105016	0.018023407	15.83369405	1.804143357	Superficial	KLF5, SOX2
RASGRP1	2.782742217	0.008904627	0.040392504	17.65106615	2.44746748	Basal	SOX2 + KLF5
PLA2G3	2.78085798	0.000989444	0.008042817	18.96022052	2.368144974	Superficial	SOX2
SERPINB13	2.770886827	0.000119764	0.012455402	64.24636718	20.44419918	Basal	SOX2
FOXE1	2.755248075	0.000795824	0.007615337	34.51116941	5.187653321	Superficial	SOX2
HRH1	2.7242374	0.001883197	0.014679007	24.54736841	3.982327992	Suprabasal	SOX2 + KLF5
APOL2	2.72256637	0.001232925	0.008679903	27.58021793	3.445414582	Superficial	SOX2
IFI27	2.685018167	0.002836074	0.0176927	78.44556802	38.98017695	Suprabasal	SOX2
KITLG	2.677505538	7.20E-05	0.010489143	40.63911213	7.568671567	Suprabasal	SOX2
LY75	2.675558265	5.55E-05	0.007176147	16.56232468	1.694338305	Superficial	SOX2, SOX2 + KLF5
SULF1	2.665452586	0.008904627	0.034252903	2.866133536	0.322283713	Suprabasal	SOX2 + KLF5
CAMK1D	2.657917619	0.000506425	0.007178523	49.15873668	9.200779378	Superficial	KLF5
SHF	2.642479077	0.001883197	0.010234018	10.17290233	1.181406638	Superficial	SOX2
APOL3	2.616122791	0.000317696	0.007176147	14.03510257	2.124512865	Superficial	SOX2
PLSCR3	2.590344397	9.30E-05	0.007176147	51.28244557	9.30799657	Superficial	SOX2
DPYD	2.582534489	0.000250281	0.007176147	56.95813881	13.81974657	Superficial	SOX2
C1orf74	2.569639155	0.000635975	0.007274975	18.30073531	2.487355771	Superficial	SOX2
HS3ST3A1	2.549605795	0.003462059	0.014108599	16.86965958	2.495265241	Superficial	SOX2 + KLF5
ADAT3	2.540027253	0.000196466	0.007176147	10.05232351	1.179084409	Superficial	SOX2
RARB	2.530013416	0.000992312	0.008042817	22.96760446	3.024920953	Superficial	SOX2 + KLF5, SOX2
TSPAN1	2.529823549	0.008904627	0.025990329	11.37029984	1.026495801	Superficial	SOX2
GALNT5	2.527879018	0.000153669	0.007176147	62.61494461	14.19470699	Superficial	SOX2, KLF5
HPGD	2.517803476	9.30E-05	0.012455402	63.4370818	26.66092629	Basal	SOX2
NEK6	2.497806445	0.000196466	0.010489143	9.695906506	1.247626474	Suprabasal	SOX2
USP2	2.470920442	7.20E-05	0.010489143	20.35654798	3.795647067	Suprabasal	SOX2
CDH3	2.454629294	0.001883197	0.014679007	33.06720283	5.979505217	Suprabasal	SOX2

NRP2	2.4318478	0.000635975	0.010733065	10.71042608	1.788625	Suprabasal	SOX2, SOX2 + KLF5
CLDN1	2.418800238	0.000506425	0.007178523	59.84833475	16.1506835	Superficial	SOX2, SOX2 + KLF5
EHD2	2.365985362	0.000992312	0.008042817	22.15175338	3.977983815	Superficial	SOX2
EPHA4	2.363866254	0.002315114	0.011381143	23.97424291	3.646350234	Superficial	SOX2, KLF5
ATP11C	2.357061891	0.006166656	0.020290909	9.252597911	1.160883516	Superficial	SOX2
TMTC3	2.351630508	0.000635975	0.007274975	45.10321587	9.259139716	Superficial	SOX2
VDR	2.343310449	0.000506425	0.007178523	46.65513349	9.496180257	Superficial	SOX2
ILDR1	2.3418189	0.000635975	0.007274975	10.28288777	1.988042993	Superficial	SOX2
FZD10	2.335435136	0.000196466	0.007176147	38.87791107	6.491347125	Superficial	SOX2
GJB4	2.297127992	0.001883197	0.010234018	14.24072294	2.556640803	Superficial	SOX2
SOX21	2.296941678	0.000506425	0.007178523	41.19240615	8.728487216	Superficial	SOX2 + KLF5, SOX2
SERINC5	2.292352434	0.000992312	0.008042817	57.34016553	13.89975778	Superficial	SOX2
APOL6	2.290382729	0.000401828	0.007176147	47.34837516	9.465108027	Superficial	SOX2
SH3RF2	2.288890426	0.000401828	0.007176147	57.94436358	14.02325258	Superficial	KLF5, SOX2
LGALSL	2.268271415	0.001526458	0.009387421	55.59902515	18.58782962	Superficial	SOX2
AADAC	2.267827947	0.005105016	0.018023407	10.87524615	1.59110865	Superficial	SOX2
SGPP2	2.250143174	0.000119764	0.007176147	52.07167939	12.31623848	Superficial	SOX2
PDZK1IP1	2.242093901	0.001526458	0.016792744	64.0916668	22.57691274	Basal	SOX2
GGH	2.23883892	0.000317696	0.007176147	60.15013769	13.53596753	Superficial	SOX2, SOX2 + KLF5
PKP2	2.222886641	0.000795824	0.007615337	26.63168494	2.617248693	Superficial	SOX2
UTRN	2.217242242	0.000196466	0.007176147	50.01075484	10.51356248	Superficial	SOX2, SOX2 + KLF5
NUP210	2.213606148	0.000795824	0.010838271	9.800731136	2.142050625	Suprabasal	SOX2
GCNT2	2.205203227	0.000317696	0.007176147	24.98548601	4.185120601	Superficial	SOX2
TSPAN3	2.193440752	0.000250281	0.007176147	72.63979106	28.82128249	Superficial	SOX2
MYOF	2.181332839	0.000795824	0.007615337	51.76574142	12.51822189	Superficial	SOX2 + KLF5
DAPK2	2.170784779	0.001232925	0.012246384	11.93187041	2.113989461	Suprabasal	SOX2
CEP72	2.167186654	0.000196466	0.010489143	5.579343935	1.139148869	Suprabasal	SOX2
GALR2	2.166969424	0.001232925	0.008679903	13.57405301	2.436665487	Superficial	SOX2
AMOTL1	2.165279249	0.000153669	0.007176147	53.80914283	12.90952452	Superficial	SOX2
FAM83E	2.154161807	0.001526458	0.009387421	6.837893593	1.341925006	Superficial	KLF5, SOX2
HBEGF	2.139981014	0.000401828	0.007176147	49.07344726	14.25840594	Superficial	SOX2
GIPC2	2.130796901	0.000119764	0.007176147	11.29330817	1.737078975	Superficial	SOX2, SOX2 + KLF5
PIK3CB	2.116387008	0.000317696	0.007176147	30.49497273	5.37065142	Superficial	SOX2
FNTB	2.113774756	0.000196466	0.007176147	38.35216549	9.331467725	Superficial	SOX2
CALML4	2.098383243	0.006116011	0.020290909	16.40768279	3.452763393	Superficial	SOX2
PPP2R5B	2.096347506	0.000196466	0.007176147	8.853189121	1.585170099	Superficial	SOX2
RAI1	2.083551412	0.000401828	0.007176147	19.62176647	3.030777351	Superficial	SOX2
SOX2	2.081913146	0.000401828	0.007176147	64.78270526	23.45059511	Superficial	SOX2, KLF5
JDP2	2.080656788	0.000119764	0.007176147	38.95069837	7.653122801	Superficial	SOX2
KLHL5	2.07040443	0.001883197	0.014679007	18.03936572	3.182826682	Suprabasal	SOX2
PADI3	2.067623508	0.006166656	0.032981404	5.285130858	0.790356966	Basal	SOX2
HS3ST1	2.063319572	0.001232925	0.015721789	23.50845249	5.330860422	Basal	SOX2, SOX2 + KLF5
ARHGAP23	2.062876631	0.000992312	0.008042817	43.87809247	10.02607057	Superficial	SOX2
HECW2	2.058221439	9.30E-05	0.007176147	15.71587344	3.116907862	Superficial	SOX2
PRICKLE2	2.055478112	0.000795824	0.010838271	25.65292316	5.691109794	Suprabasal	SOX2
C9orf152	2.042243894	0.000317696	0.007176147	13.25621132	2.381023333	Superficial	SOX2
F2RL1	2.04202804	0.000795824	0.007615337	15.22355279	3.090463749	Superficial	SOX2
STK17B	2.02769262	0.001526458	0.009387421	41.63652012	7.333922578	Superficial	SOX2
C9orf40	2.018582283	0.000401828	0.007176147	18.20397196	3.43412846	Superficial	SOX2
MYH9	2.016181739	0.000401828	0.007176147	61.10773592	18.88556925	Superficial	SOX2 + KLF5, SOX2
PLAUR	2.002795365	0.002836074	0.012662188	15.93572912	3.786355668	Superficial	SOX2
CD44	1.998034871	0.000506425	0.007178523	61.11347514	19.65892181	Superficial	SOX2
PPARG	1.995811597	0.000401828	0.007176147	27.13150116	5.448962677	Superficial	SOX2, KLF5
PDLIM4	1.989624172	0.000317696	0.007176147	69.2066852	25.82174452	Superficial	SOX2, SOX2 + KLF5
C16orf74	1.983715146	0.005105016	0.018023407	23.21339782	4.600880205	Superficial	SOX2
NUAK2	1.98293209	0.001232925	0.008679903	43.77931763	10.90822545	Superficial	SOX2 + KLF5
KLK7	1.977463828	0.001883197	0.010234018	66.73816902	34.01620165	Superficial	SOX2
LPCAT4	1.972938051	0.000795824	0.007615337	62.90111134	20.64436002	Superficial	SOX2 + KLF5, SOX2
RIPK2	1.966802897	0.000795824	0.007615337	24.08933789	4.963363477	Superficial	SOX2
VANGL2	1.960523499	0.005105016	0.018023407	9.102787321	1.461988544	Superficial	SOX2
KCTD12	1.956658379	0.000401828	0.010489143	15.00195718	2.947502003	Suprabasal	SOX2
PLXNA1	1.956245434	0.002315114	0.011381143	13.16078784	2.350686874	Superficial	SOX2
UBE2Z	1.951186499	0.000506425	0.007178523	50.65988193	13.56189302	Superficial	SOX2
LRRC8B	1.948757418	0.001526458	0.009387421	14.99469118	2.794121962	Superficial	SOX2 + KLF5
DUSP10	1.946963734	0.000992312	0.01142308	16.06853386	3.224143338	Suprabasal	SOX2
MVP	1.941839212	0.000401828	0.007176147	51.13459459	14.41912597	Superficial	SOX2
TLE1	1.923101892	0.000119764	0.007176147	27.10808303	5.539944391	Superficial	SOX2, SOX2 + KLF5
CLMN	1.900395402	0.000401828	0.010489143	10.25851781	1.920383559	Suprabasal	SOX2
BTBD11	1.881944659	0.000401828	0.007176147	32.29979612	7.660902713	Superficial	SOX2
UCK2	1.879818849	0.000506425	0.007178523	25.81261694	5.843574533	Superficial	SOX2
PRICKLE3	1.874661139	0.001232925	0.008679903	19.11973397	4.835371474	Superficial	SOX2
SGMS2	1.870786282	0.000401828	0.007176147	35.5703333	7.638278866	Superficial	SOX2 + KLF5, SOX2
FMN1	1.866940741	0.000992312	0.008042817	13.93481253	2.490917074	Superficial	SOX2 + KLF5, SOX2
MBOAT1	1.856378922	0.004211401	0.016013117	20.12833534	4.645925894	Superficial	SOX2
PLCXD2	1.855577703	0.001883197	0.018415354	10.25091971	1.496509635	Basal	SOX2
SLFN5	1.848849547	0.001232925	0.008679903	27.75619287	6.018649947	Superficial	SOX2
TRAK1	1.846069152	0.000401828	0.007176147	35.07851392	7.922600535	Superficial	SOX2, KLF5
PLAG1	1.834621629	0.002315114	0.020052792	7.541644707	1.172226914	Basal	SOX2
PCGF2	1.829922147	0.000401828	0.007176147	18.9966338	3.843979558	Superficial	SOX2
CFH	1.828796647	0.002315114	0.011381143	15.10224119	2.619784914	Superficial	SOX2

BPNT1	1.821261596	0.000119764	0.007176147	23.50791508	5.202532602	Superficial	SOX2
SERPINB5	1.819859014	0.000506425	0.007178523	67.98078257	28.24706043	Superficial	SOX2, SOX2 + KLF5
VPS9D1	1.8139499	0.004211401	0.016013117	32.5671341	9.788833771	Superficial	SOX2
PARP14	1.810369034	0.005105016	0.018023407	38.26737244	7.373411595	Superficial	SOX2
PPFIA3	1.808834433	0.000506425	0.007178523	14.42758209	3.603490342	Superficial	SOX2
HAPLN3	1.805854094	0.017811616	0.043066688	9.09934586	1.590747789	Superficial	SOX2
SLC26A2	1.800102188	0.001526458	0.009387421	45.30830537	11.39121798	Superficial	SOX2
RHBDF2	1.7980639	0.000317696	0.007176147	22.85167975	5.481160876	Superficial	SOX2
EPB41L1	1.793989519	0.001232925	0.008679903	42.29613711	10.90537302	Superficial	SOX2
LZTS2	1.792350541	0.000795824	0.007615337	21.57360734	4.784089227	Superficial	SOX2
DUOX1	1.791874816	0.000250281	0.007176147	78.09045313	40.85380185	Superficial	SOX2
RGS2	1.791283961	0.012681065	0.033478436	25.73956524	5.400301716	Superficial	SOX2
CDH1	1.790786868	0.000196466	0.007176147	66.43421356	23.36472372	Superficial	SOX2
FLNB	1.789509468	0.000795824	0.007615337	41.12314191	11.05496167	Superficial	SOX2
ACSL5	1.783440086	0.006166656	0.027303057	11.74714312	2.411107733	Suprabasal	SOX2
EFNA3	1.780245834	0.000317696	0.007176147	27.72683451	6.791475493	Superficial	SOX2
MX1	1.775059796	0.000992312	0.008042817	52.23414703	14.14516091	Superficial	SOX2
HSPA4L	1.774195024	0.000992312	0.008042817	44.30957301	11.98002916	Superficial	SOX2
NMI	1.773761102	0.002315114	0.011381143	47.10510036	12.99264976	Superficial	SOX2
TMED8	1.76610339	0.000795824	0.007615337	9.847590848	1.882202548	Superficial	SOX2
ACTN1	1.757688537	0.000992312	0.008042817	49.01098628	14.15301096	Superficial	SOX2
ARHGAP26	1.751173085	7.20E-05	0.007176147	19.94818841	4.677088953	Superficial	SOX2 + KLF5
RAB40B	1.750815018	0.002315114	0.011381143	29.68013769	6.615881291	Superficial	SOX2
LITAF	1.746481223	0.000317696	0.010489143	65.22896326	21.81166448	Suprabasal	SOX2, SOX2 + KLF5
BIK	1.744369223	0.001526458	0.016792744	17.82374359	4.38678935	Basal	SOX2
LMTK3	1.740611516	0.000401828	0.007176147	31.40700303	7.955807442	Superficial	SOX2
PCP4L1	1.738642273	0.002315114	0.020052792	8.98214398	2.87134725	Basal	KLF5
ZNF385A	1.737405876	0.001526458	0.009387421	52.81129606	15.73736124	Superficial	KLF5
TRIM34	1.734596734	0.002257606	0.011381143	12.00649374	2.23866282	Superficial	SOX2
DUSP7	1.73342227	0.005105016	0.018023407	26.21992767	6.095908265	Superficial	SOX2
IGF2R	1.719942816	0.000992312	0.008042817	48.48639949	13.65241974	Superficial	SOX2 + KLF5, SOX2
UBR1	1.719522138	0.000196466	0.007176147	14.06178488	2.530448228	Superficial	SOX2
SMAD7	1.715565935	0.000317696	0.010489143	23.33039354	6.328498368	Suprabasal	SOX2
CFAP45	1.713651152	0.008904627	0.034252903	3.768521822	1.162924672	Suprabasal	SOX2
PSMB9	1.713579321	0.001883197	0.014679007	46.76968644	13.20609663	Suprabasal	SOX2
RAD50	1.709565758	0.000250281	0.007176147	41.71108638	10.58867033	Superficial	SOX2
ARFGEF1	1.709561526	5.55E-05	0.007176147	59.31506671	18.23150087	Superficial	SOX2
PCCA	1.708003858	0.000250281	0.007176147	25.73152384	5.309542158	Superficial	SOX2
TNIK	1.706346895	0.017811616	0.043066688	5.392242583	0.752506829	Superficial	SOX2
ITGAV	1.702941913	0.000317696	0.007176147	28.00302136	5.643803668	Superficial	SOX2
NAB2	1.702282164	0.000401828	0.007176147	9.914356536	2.392261266	Superficial	SOX2
PITRM1	1.702119527	0.002315114	0.011381143	39.3035774	9.553439545	Superficial	SOX2
GALNT7	1.696244002	0.000317696	0.007176147	53.0088514	14.34898878	Superficial	KLF5
FAM3C	1.6929743	0.000992312	0.008042817	62.57202139	21.71212462	Superficial	SOX2, SOX2 + KLF5
CARNMT1	1.69008525	0.000635975	0.007274975	27.23772045	6.517738001	Superficial	SOX2
PLXNB1	1.689316796	0.000635975	0.007274975	28.19070091	8.442735153	Superficial	SOX2
AQP3	1.688886054	0.000635975	0.007274975	88.79367883	56.05032049	Superficial	SOX2
MAOA	1.687062072	0.000250281	0.007176147	53.15988498	16.42076969	Superficial	SOX2
PLEKHA7	1.686755148	0.000635975	0.007274975	50.68256995	15.01051921	Superficial	SOX2
BHLHE40	1.683975933	0.000250281	0.012455402	66.70715515	29.07714623	Basal	SOX2
TMEM268	1.683413946	0.000795824	0.007615337	6.305050499	1.013843833	Superficial	SOX2
RGS12	1.679974365	0.000196466	0.007176147	52.44813675	16.05946172	Superficial	SOX2
FAM3B	1.679634197	0.000401828	0.007176147	76.76679222	41.20933182	Superficial	SOX2, KLF5
FAM83C	1.679177088	0.000992312	0.008042817	34.67295623	9.58995339	Superficial	SOX2
USP54	1.677511187	0.001883197	0.010234018	48.22455818	13.1742308	Superficial	SOX2
NRARP	1.676776951	0.001526458	0.009387421	59.48866715	22.43678013	Superficial	SOX2
VANGL1	1.674308055	0.000992312	0.008042817	26.754429	5.911651905	Superficial	SOX2
SERTAD4-AS1	1.665063871	0.017811616	0.043066688	7.56408644	1.836828275	Superficial	KLF5
CD9	1.664880927	0.000317696	0.007176147	95.7959489	87.17960747	Superficial	SOX2 + KLF5, SOX2
SIPA1L1	1.662110852	9.30E-05	0.007176147	45.53935844	12.70121283	Superficial	SOX2
FZD1	1.660406189	0.000401828	0.007176147	15.36367518	3.652475248	Superficial	SOX2
DSG3	1.659685097	0.000401828	0.007176147	76.2731689	37.72281451	Superficial	KLF5, SOX2 + KLF5, SOX2
FAM83G	1.655884408	0.001883197	0.010234018	35.56417675	10.87087784	Superficial	SOX2
ADCY7	1.655709863	0.002836074	0.012662188	26.2365789	5.391710904	Superficial	SOX2
MDGA1	1.649359333	0.005105016	0.024262694	6.424994564	1.914752811	Suprabasal	SOX2
CEACAM6	1.648410896	0.001232925	0.015721789	14.47583323	4.150146377	Basal	SOX2
ATP2C1	1.646785429	0.000401828	0.007176147	34.44258344	8.519262101	Superficial	SOX2
TCF7L2	1.645495297	0.000635975	0.007274975	24.10556753	5.483411759	Superficial	SOX2
DEF6	1.643050311	0.000401828	0.007176147	22.74032432	5.806535233	Superficial	SOX2
ERI1	1.638743032	0.000795824	0.007615337	19.59835376	5.244473791	Superficial	SOX2
E2F8	1.632929528	0.002836074	0.012662188	19.05196528	4.16204622	Superficial	SOX2
PRDM1	1.628753891	0.005105016	0.029792864	9.74035703	2.23802724	Basal	SOX2
WNT5A	1.626161756	0.000401828	0.007176147	40.61458636	11.46950904	Superficial	SOX2
ACSL1	1.625384316	0.006166656	0.020290909	45.16007735	13.44840278	Superficial	SOX2
IFIT2	1.625274549	0.021001058	0.04834985	7.148106854	1.150788534	Superficial	SOX2
AEBP2	1.622302638	0.000795824	0.007615337	39.97302843	9.842466958	Superficial	SOX2
STX6	1.612517544	0.000795824	0.007615337	41.35112964	11.00713868	Superficial	SOX2
TEF	1.60997414	0.007423146	0.030449933	15.90490035	4.406701305	Suprabasal	SOX2
ZNF326	1.607653042	0.000119764	0.007176147	58.61505907	18.15590786	Superficial	SOX2
CASK	1.607623668	0.000317696	0.007176147	25.64028894	6.432160107	Superficial	SOX2

STAT6	1.605855949	0.000992312	0.008042817	35.45387829	11.31713013	Superficial	SOX2
TLR5	1.603201904	0.001232925	0.008679903	17.15841106	4.438147352	Superficial	KLF5
NECTIN1	1.600704419	0.000992312	0.008042817	62.49026247	22.13619408	Superficial	SOX2
LDLRAD3	1.598177175	0.000795824	0.007615337	13.15101248	2.23742001	Superficial	SOX2
SIRT6	1.593566613	0.003462059	0.014108599	11.50078903	3.946469396	Superficial	SOX2
ADGRG1	1.588484691	0.002315114	0.011381143	40.98278712	11.67161753	Superficial	SOX2
MACF1	1.585172836	0.001883197	0.010234018	45.42867422	13.42301982	Superficial	SOX2 + KLF5, SOX2
KALRN	1.568401192	0.001232925	0.008679903	39.8906692	11.36810286	Superficial	SOX2
EFNA1	1.567624298	0.000250281	0.007176147	35.81170057	9.972419373	Superficial	SOX2
SIAH2	1.561358934	0.000506425	0.007178523	54.29178907	18.17855221	Superficial	SOX2
MPP7	1.555620236	0.000795824	0.007615337	74.5401543	35.82324735	Superficial	SOX2
LRIG3	1.554041648	0.001526458	0.009387421	16.87745803	4.27268611	Superficial	SOX2
CBLC	1.550539974	0.000795824	0.007615337	54.08040173	16.73185142	Superficial	SOX2
RAPGEFL1	1.546741477	0.000635975	0.007274975	59.0466891	21.22229912	Superficial	KLF5, SOX2
NBEAL2	1.543643779	0.001526458	0.009387421	66.32498761	27.02085762	Superficial	SOX2
TMPRSS11D	1.540082196	0.008904627	0.040392504	18.5208653	5.78738771	Basal	SOX2 + KLF5
CYB561D1	1.539648021	0.003462059	0.014108599	15.63686224	3.624462484	Superficial	SOX2
DYRK2	1.537828927	0.000317696	0.007176147	30.61055724	7.976776919	Superficial	SOX2
EGR2	1.532135907	0.010644787	0.029507311	8.930218254	2.317618094	Superficial	SOX2
UNC5B	1.532021274	0.004211401	0.016013117	21.54159413	7.012623892	Superficial	SOX2
EGR1	1.531257162	0.000401828	0.007176147	77.3750348	37.25352246	Superficial	SOX2
ID4	1.529712713	0.005105016	0.018023407	61.2478735	25.59836537	Superficial	SOX2
NRIP1	1.527774608	0.000992312	0.008042817	31.56908623	8.02702205	Superficial	SOX2, SOX2 + KLF5
SETBP1	1.526392337	0.000795824	0.007615337	12.37924636	2.847820355	Superficial	SOX2 + KLF5, SOX2
CTTNBP2	1.523952442	0.001526458	0.009387421	38.23349606	9.953505753	Superficial	SOX2
ZNF750	1.521825816	0.000635975	0.007274975	71.74142611	34.19483123	Superficial	SOX2
ROBO1	1.521395177	0.002315114	0.011381143	20.09483419	4.223508011	Superficial	SOX2
SERPINB2	1.520402251	0.001232925	0.015721789	56.47425191	25.15403273	Basal	SOX2 + KLF5
DLG5	1.520094924	0.000795824	0.010838271	31.97247699	10.87101714	Suprabasal	SOX2
USP32	1.513329317	0.000992312	0.008042817	27.57071909	6.844546538	Superficial	SOX2
MBTPS1	1.5075199	0.000992312	0.008042817	40.77920392	12.36148303	Superficial	SOX2
SPTBN2	1.507423391	0.000635975	0.007274975	45.72760689	15.53689929	Superficial	SOX2
MYH14	1.506036602	0.000635975	0.007274975	65.02307798	27.62556019	Superficial	SOX2, SOX2 + KLF5, KLF5
TTC39B	1.504724353	0.000153669	0.007176147	31.35300257	8.277748331	Superficial	SOX2
PPFIBP2	1.502913882	0.000506425	0.007178523	38.08840061	10.11643122	Superficial	SOX2
PARP9	1.497471188	0.002836074	0.012662188	45.0973443	13.68164257	Superficial	SOX2
MCC	1.495164205	0.001232925	0.008679903	33.17844453	9.480764163	Superficial	SOX2, SOX2 + KLF5
SLC16A7	1.492659835	0.000317696	0.007176147	36.68869929	10.26325143	Superficial	SOX2 + KLF5
KIAA1522	1.491037323	0.000795824	0.007615337	57.95200293	21.15180697	Superficial	SOX2
CTF1	1.489903466	0.015054848	0.038009884	6.497786284	2.532560526	Superficial	SOX2
ZFYVE28	1.489529705	0.004211401	0.016013117	11.28814489	2.205883735	Superficial	SOX2
MARK2	1.485849189	0.000795824	0.007615337	64.26837362	27.57208954	Superficial	SOX2
ZNF512B	1.482940443	0.000153669	0.007176147	14.48764198	3.998364131	Superficial	SOX2
EEA1	1.479639763	0.000196466	0.007176147	68.01721549	26.67149732	Superficial	SOX2 + KLF5, SOX2
ANG	1.470912351	0.017811616	0.043066688	10.58303921	2.925742081	Superficial	SOX2
PSTPIP2	1.470147182	0.000153669	0.007176147	20.22059631	3.435694389	Superficial	SOX2
GLS	1.46994638	0.000119764	0.007176147	24.94966084	6.436039892	Superficial	SOX2
ACADSB	1.46729936	0.000635975	0.007274975	33.22753083	7.725227649	Superficial	SOX2
TAPBP	1.462687605	0.002315114	0.011381143	60.46527377	23.25742706	Superficial	SOX2
TMC6	1.459446866	0.003462059	0.014108599	33.19438017	10.41223051	Superficial	SOX2
ATF3	1.4580875	0.003462059	0.014108599	64.8069383	31.24154827	Superficial	SOX2
GPAT4	1.454502053	0.001232925	0.008679903	16.79760858	4.655621659	Superficial	SOX2
P2RY2	1.454448709	0.001883197	0.010234018	26.87639549	8.337861884	Superficial	SOX2
NR2F2	1.453940982	0.000250281	0.007176147	7.515066166	1.627785245	Superficial	SOX2
KLF4	1.451647249	0.000317696	0.007176147	74.16657085	38.12955866	Superficial	SOX2 + KLF5
PPARGC1B	1.451020759	0.010644787	0.029507311	17.5730362	4.146815669	Superficial	KLF5
MARK1	1.450576993	0.005105016	0.018023407	11.31410423	2.61341336	Superficial	SOX2
ACVR2A	1.449181971	0.000795824	0.007615337	30.56926601	7.030736758	Superficial	SOX2
EFS	1.443933494	0.001526458	0.009387421	29.99052364	9.591608224	Superficial	SOX2
FOSL1	1.44377816	0.012681065	0.049425568	19.99797835	6.879080718	Basal	SOX2
SOX9	1.443054728	0.003462059	0.024854712	24.37891667	6.330952656	Basal	SOX2
RAB11FIP1	1.442200578	0.000317696	0.012455402	44.59727837	19.1411243	Basal	SOX2, SOX2 + KLF5
EXT1	1.436512243	0.002836074	0.012662188	13.54263902	3.548045055	Superficial	SOX2
GBP3	1.435078234	0.002836074	0.012662188	39.4589241	11.77543464	Superficial	SOX2
AGPAT5	1.429902007	0.001883197	0.010234018	27.61419053	6.879942189	Superficial	SOX2
TJP2	1.428786705	0.000250281	0.012455402	37.63135572	12.37153651	Basal	SOX2, SOX2 + KLF5
MYO1B	1.424682138	0.000795824	0.0136425	35.06249235	12.49455715	Basal	SOX2
WRN	1.422447463	0.000317696	0.007176147	12.03213883	2.82000383	Superficial	SOX2
MTSS1	1.414208577	7.20E-05	0.007176147	56.05422812	18.62672861	Superficial	SOX2
RARA	1.414049556	0.007423146	0.022959455	10.0578091	1.361551578	Superficial	SOX2
CELSR2	1.412827235	0.001883197	0.010234018	32.83846129	8.471091898	Superficial	SOX2
TMEM62	1.41211041	0.000401828	0.007176147	15.85865511	4.494080284	Superficial	SOX2
ATP1A1	1.410743248	0.002836074	0.012662188	54.54542011	19.58899334	Superficial	SOX2
KDM6B	1.410464515	0.000635975	0.007274975	26.18538702	7.047815	Superficial	SOX2
AHR	1.409902398	0.000196466	0.010489143	37.6195811	12.92727326	Suprabasal	SOX2
FAM83B	1.409210634	0.005105016	0.018023407	24.44070299	5.677640141	Superficial	SOX2, SOX2 + KLF5
IL15RA	1.405856308	0.000992312	0.01142308	8.983045649	2.947652454	Suprabasal	SOX2
TRNP1	1.39874506	0.001526458	0.016792744	26.25894725	8.679371806	Basal	SOX2
IGSF9	1.397302481	0.015054848	0.038009884	10.74871348	2.239511216	Superficial	SOX2
B3GALNT2	1.394665908	0.000317696	0.007176147	23.68419665	6.990842078	Superficial	SOX2

ANGPTL4	1.390767663	9.30E-05	0.012455402	16.31586531	4.670732675	Basal	SOX2 + KLF5, SOX2
GFOD1	1.385270232	0.001883197	0.010234018	23.04950039	5.817350747	Superficial	SOX2
TPPP	1.384548287	0.004211401	0.021879278	16.77886912	4.832901743	Suprabasal	SOX2
DUOXA1	1.37848433	0.000401828	0.012455402	48.21228319	19.71710377	Basal	SOX2
EDC4	1.373595403	0.000980874	0.008042817	19.45990268	5.084122399	Superficial	SOX2
PRCP	1.372815391	0.000506425	0.007178523	51.28852932	17.1700345	Superficial	SOX2
KDM4A	1.368537175	0.000506425	0.007178523	21.96859569	4.670725141	Superficial	SOX2
AHCTF1	1.364426399	0.000992312	0.008042817	20.42186335	4.885164396	Superficial	SOX2
CCDC51	1.364414193	0.006166656	0.020290909	30.08544728	9.187453014	Superficial	SOX2
GBF1	1.363385147	0.000992312	0.008042817	37.52272583	12.18132941	Superficial	SOX2
CIB1	1.359374292	0.000635975	0.007274975	71.89402045	37.61930816	Superficial	SOX2
ZBTB40	1.357638761	0.001526458	0.009387421	9.441108813	1.982079465	Superficial	SOX2
PDK3	1.356669527	0.001232925	0.008679903	15.30966875	4.188927431	Superficial	SOX2
UBE2L6	1.353073987	0.001526458	0.009387421	34.95959257	8.921171972	Superficial	SOX2
ANXA8L1	1.352486799	0.000506425	0.007178523	31.13710693	8.787587644	Superficial	SOX2
ETS2	1.348438538	0.000506425	0.010489143	70.5467627	37.00163852	Suprabasal	SOX2, SOX2 + KLF5
ZNF316	1.346975294	0.000506425	0.007178523	14.80715013	4.977715972	Superficial	SOX2
PPFIA1	1.345698612	5.55E-05	0.007176147	34.56770634	9.912383496	Superficial	SOX2
NUP205	1.342293498	0.000401828	0.007176147	14.31714078	4.145322997	Superficial	SOX2
TMEM129	1.341297541	0.003462059	0.014108599	15.54081075	4.998039805	Superficial	SOX2
KRT6A	1.338300874	0.007423146	0.036548425	70.83206676	49.5782978	Basal	SOX2, KLF5
UTP20	1.338154756	0.003462059	0.014108599	13.15682885	2.679443984	Superficial	SOX2
CERS3	1.336695002	0.000992312	0.008042817	66.75354368	27.89605679	Superficial	KLF5, SOX2
PHF2	1.333360461	0.000506425	0.007178523	18.51205223	4.924088628	Superficial	SOX2
SOWAHB	1.330616268	0.000506425	0.010489143	19.25278672	6.539010872	Suprabasal	SOX2
ARHGEF37	1.330558945	0.000119764	0.007176147	26.30959446	7.028673068	Superficial	KLF5
UBE2O	1.32923008	0.012681065	0.033478436	10.25200313	3.090639077	Superficial	SOX2
SEMA4B	1.328875176	0.005105016	0.018023407	40.34899141	14.00945595	Superficial	SOX2
MUC15	1.328370967	0.000506425	0.007178523	59.25483221	20.34713306	Superficial	SOX2
TSHZ1	1.32797418	0.000795824	0.007615337	17.38245968	4.734116887	Superficial	SOX2
SLC30A4	1.324891857	0.000992312	0.008042817	15.57983981	4.129145692	Superficial	SOX2
PLAC8	1.321845779	0.008904627	0.040392504	23.19185551	9.186900909	Basal	SOX2
PROS1	1.320692718	0.001232925	0.008679903	16.76337057	4.722148576	Superficial	SOX2
QRSL1	1.319743783	0.000196466	0.007176147	15.55650242	4.687149578	Superficial	SOX2
OCIAD2	1.31691942	0.000795824	0.007615337	50.38690675	15.63470735	Superficial	SOX2 + KLF5
MUC1	1.316339356	0.008904627	0.040392504	16.0412245	4.959969675	Basal	KLF5, SOX2
PAK6	1.316197272	0.005105016	0.018023407	17.81476293	5.584040472	Superficial	SOX2
RNASE4	1.314834402	0.005094082	0.018023407	12.16976102	3.108359674	Superficial	SOX2
ARHGAP18	1.313208605	0.001526458	0.009387421	17.67087429	4.49131787	Superficial	SOX2, SOX2 + KLF5
TOP2B	1.312446793	0.000795824	0.007615337	39.86008267	11.05700902	Superficial	SOX2
MARVELD2	1.312375904	0.000317696	0.007176147	28.05964149	9.039323031	Superficial	SOX2
ERC1	1.308852525	0.000992312	0.008042817	39.6157528	12.99023703	Superficial	SOX2 + KLF5, SOX2
TMEM132A	1.306731913	0.000250281	0.007176147	36.14513057	9.791551434	Superficial	SOX2
TMEM125	1.305306728	0.000635975	0.007274975	31.74072791	10.27266171	Superficial	SOX2
ATP2A2	1.30393959	0.002836074	0.012662188	67.33126411	29.68867078	Superficial	SOX2
GSTO1	1.30389326	0.002315114	0.011381143	58.6348769	23.03531844	Superficial	KLF5
SOX13	1.301876207	0.004211401	0.016013117	13.72289931	3.118639101	Superficial	SOX2 + KLF5, SOX2
RCC1L	1.30181624	0.000795824	0.007615337	28.93228558	8.302303983	Superficial	SOX2
PI4K2B	1.301679821	0.004211401	0.016013117	19.88158248	4.693846417	Superficial	SOX2
NBN	1.293606262	0.000317696	0.007176147	40.2244907	12.58433084	Superficial	SOX2
EPHA1	1.292228293	0.000795824	0.007615337	47.17389356	15.8337107	Superficial	SOX2
CBX6	1.29167696	0.002315114	0.011381143	31.83148599	10.1731285	Superficial	SOX2
TMPRSS4	1.291648313	0.000317696	0.012455402	52.64854085	22.60403832	Basal	SOX2, KLF5, SOX2 + KLF5
PDE4D	1.288243	0.003462059	0.014108599	11.39595236	2.129328554	Superficial	SOX2, SOX2 + KLF5
DBNDD2	1.286934243	0.001219203	0.008679903	49.89250831	17.33137271	Superficial	SOX2
IRX2	1.284964767	5.55E-05	0.007176147	13.28747174	3.955746035	Superficial	SOX2
PTPRF	1.283332755	0.001883197	0.010234018	54.86782032	19.86110899	Superficial	SOX2
RNGTT	1.281199467	0.000250281	0.007176147	27.16286848	7.841618321	Superficial	SOX2
PLA2G4B	1.280849172	0.017698983	0.043066688	29.65748348	11.18833209	Superficial	SOX2
CDC42EP1	1.280809566	0.000506425	0.012898798	49.93940725	20.79124099	Basal	SOX2
ARL5B	1.2806538	0.002315114	0.011381143	38.32525881	10.54936115	Superficial	SOX2
FAM135A	1.278999435	0.000992312	0.008042817	63.70652239	28.27070091	Superficial	SOX2
RAPGEF5	1.278312014	0.000153669	0.010489143	38.66857075	15.89877526	Suprabasal	SOX2, SOX2 + KLF5
PAK4	1.278029191	0.000153669	0.007176147	36.06503889	11.77443905	Superficial	SOX2
SPRYD3	1.277772516	0.000992312	0.008042817	24.20572055	6.994672203	Superficial	SOX2
FGFR3	1.277577146	0.004211401	0.016013117	43.50353723	15.34914819	Superficial	SOX2, KLF5
LSR	1.271662219	0.001526458	0.009387421	59.20009736	22.67903464	Superficial	SOX2
PARG	1.267231595	0.000153669	0.007176147	18.63111661	5.046069019	Superficial	SOX2
GSDMC	1.265904005	0.001526458	0.009387421	51.45721865	18.99913397	Superficial	KLF5
RANGAP1	1.262746573	0.001883197	0.010234018	39.92014309	12.52755039	Superficial	SOX2
CTSD	1.261128396	0.000401828	0.007176147	87.81296053	60.78044895	Superficial	SOX2
KDM1A	1.259944271	0.001883197	0.010234018	24.42107785	6.395816716	Superficial	SOX2
SHROOM2	1.258271405	0.000635975	0.007274975	16.76833305	5.01205908	Superficial	SOX2 + KLF5
PTPN1	1.25765891	0.000506425	0.007178523	39.88181934	12.6414766	Superficial	SOX2
MAP7D1	1.256968222	0.000795824	0.007615337	56.70640296	22.14215924	Superficial	SOX2
FSCN1	1.256104721	0.001883197	0.010234018	24.7998747	6.980756334	Superficial	SOX2
MID1	1.254097172	0.006166656	0.020290909	15.17176327	4.455197887	Superficial	SOX2
LIN7C	1.25095532	0.005105016	0.018023407	43.72459786	13.99481155	Superficial	SOX2
AP1S3	1.248619366	0.002836074	0.012662188	11.99405619	3.409620745	Superficial	SOX2
SP110	1.247917051	0.002315114	0.011381143	19.79106955	6.139409573	Superficial	SOX2, KLF5

DTX3L	1.24559442	0.000795824	0.010838271	38.57229673	15.76772911	Suprabasal	SOX2
DAB2IP	1.245422489	0.001232925	0.012246384	23.98713205	8.994272101	Suprabasal	SOX2
METRNL	1.242786049	0.000317696	0.012455402	32.07582811	13.26662833	Basal	SOX2
FBLIM1	1.23859892	0.000992312	0.008042817	14.55913436	3.715759949	Superficial	SOX2
CDK5R1	1.23645138	0.000635975	0.007274975	13.61044702	4.337751879	Superficial	SOX2
BDP1	1.231393276	0.000250281	0.007176147	39.56516605	11.52677018	Superficial	SOX2
SMARCA5	1.229865827	0.000153669	0.007176147	44.63132421	14.69604831	Superficial	SOX2
ARHGEF1	1.222985993	0.002315114	0.011381143	18.69026155	5.468147191	Superficial	SOX2
ANO9	1.222957452	0.006166656	0.020290909	11.72755418	4.158654321	Superficial	KLF5, SOX2
TMEM30B	1.222077703	0.001232925	0.008679903	42.12799768	12.74691984	Superficial	SOX2
EVL	1.221452066	0.003462059	0.014108599	18.78014165	5.231162739	Superficial	SOX2
BTC	1.219656775	0.001883197	0.010234018	23.18820738	7.421255007	Superficial	SOX2
HYOU1	1.21822226	0.003462059	0.014108599	28.78499902	11.00331255	Superficial	SOX2
MREG	1.217754559	0.000401828	0.012455402	27.45165405	10.06667687	Basal	SOX2 + KLF5, SOX2
TFPI	1.217749911	0.008904627	0.040392504	20.46071555	6.988812663	Basal	SOX2
TFCP2	1.214854752	0.001883197	0.010234018	14.82542175	4.280428251	Superficial	SOX2
NOTCH3	1.213496016	0.004211401	0.016013117	41.33700568	15.82539899	Superficial	SOX2
MBOAT2	1.21344202	0.000795824	0.0136425	18.88332959	7.348331732	Basal	SOX2 + KLF5
RGMA	1.21235656	0.000795824	0.007615337	26.3261414	8.690737267	Superficial	SOX2
ROCK2	1.20861768	0.001232925	0.008679903	31.89008375	9.583988437	Superficial	SOX2 + KLF5
IL27RA	1.208220342	0.000795824	0.010838271	9.158256842	3.247951193	Suprabasal	SOX2
STK11IP	1.207708762	0.002836074	0.012662188	9.584264857	3.029777569	Superficial	SOX2
PPIP5K1	1.206931468	0.000317696	0.007176147	8.678543046	2.470109768	Superficial	SOX2
MXRA7	1.20590904	0.006166656	0.020290909	30.70137793	7.885702193	Superficial	SOX2
LIMA1	1.205072658	0.001232925	0.008679903	57.36614584	22.15407701	Superficial	SOX2, SOX2 + KLF5
P4HA2	1.203825583	0.002315114	0.011381143	29.25974961	9.077935764	Superficial	SOX2
SLC25A24	1.202476433	0.002836074	0.012662188	31.87004604	9.835333136	Superficial	SOX2 + KLF5
E2F2	1.202226402	0.000401828	0.012455402	25.69220688	10.11018419	Basal	SOX2
NCS1	1.199970243	0.010644787	0.029507311	18.84944464	5.393233849	Superficial	SOX2
ABCC5	1.199556541	0.000506425	0.010489143	47.79352163	22.35662642	Suprabasal	SOX2
VSNL1	1.198717733	0.002836074	0.012662188	41.90794306	13.72784784	Superficial	SOX2
MUC20	1.197371809	0.006166656	0.032981404	17.33627206	6.009769404	Basal	SOX2, KLF5
NIPAL4	1.196951548	0.015054848	0.038009884	5.624375738	1.930404675	Superficial	SOX2
CYP4F12	1.196520524	0.010644787	0.029507311	28.25141081	8.950303583	Superficial	SOX2
FHDC1	1.196319661	0.006166656	0.020290909	23.99616288	7.021853973	Superficial	SOX2
SCNN1A	1.194828355	0.000992312	0.014320968	27.93955498	11.20588616	Basal	SOX2, KLF5
ATP8A1	1.192565378	0.000635975	0.010733065	6.400921197	2.31669173	Suprabasal	SOX2 + KLF5
MYO10	1.19168863	0.000635975	0.007274975	35.09234401	9.749988388	Superficial	SOX2, SOX2 + KLF5
AFF1	1.191217771	0.001526458	0.009387421	36.90037795	11.39818151	Superficial	SOX2, SOX2 + KLF5
MYCBP2	1.190959105	0.001232925	0.008679903	58.42803821	21.11847344	Superficial	SOX2
GCNT3	1.190903852	0.002315114	0.011381143	68.37598759	34.32034018	Superficial	SOX2, SOX2 + KLF5
ANXA4	1.188849498	0.000635975	0.007274975	55.84232817	19.17807815	Superficial	SOX2
MLLT3	1.18449493	0.002836074	0.012662188	15.62951055	4.585014268	Superficial	SOX2, SOX2 + KLF5
PTGS1	1.18264683	0.010644787	0.029507311	20.59203145	5.959216272	Superficial	SOX2
DLG3	1.181702408	0.000506425	0.007178523	53.66370214	20.84782301	Superficial	SOX2
CXCL16	1.179795921	0.001883197	0.010234018	46.79771293	17.26782239	Superficial	SOX2
ATP11B	1.178194803	0.000635975	0.007274975	65.46139314	27.9797841	Superficial	SOX2
CACFD1	1.177820059	0.002315114	0.011381143	14.49874791	4.530803054	Superficial	SOX2
MEIS1	1.17701975	0.000992312	0.008042817	34.91496587	11.38435718	Superficial	SOX2 + KLF5
PBX2	1.176236683	0.000153669	0.007176147	30.22501723	8.896086592	Superficial	SOX2
NSUN2	1.17513159	0.004211401	0.016013117	18.99430485	5.369107729	Superficial	SOX2
FUBP3	1.172413423	9.30E-05	0.007176147	27.44845694	8.374105466	Superficial	SOX2
ATP8B1	1.17101992	0.000401828	0.012455402	39.21041059	15.67056765	Basal	SOX2 + KLF5
PARP12	1.170055421	0.000635975	0.012925379	15.58978688	5.126299574	Basal	SOX2, SOX2 + KLF5
GBA2	1.169999924	0.005105016	0.018023407	14.65856801	4.196633117	Superficial	SOX2
EPHA2	1.169962509	0.004211401	0.027185186	30.15484264	11.81886947	Basal	SOX2
ZNF700	1.167879082	0.000196466	0.007176147	12.40076197	3.881582813	Superficial	SOX2
VPS54	1.167549799	0.004211401	0.016013117	17.16867299	4.649827082	Superficial	SOX2
LMBRD2	1.162448387	0.007423146	0.022959455	15.96772823	4.108028138	Superficial	SOX2
LRRC59	1.161487916	0.000635975	0.007274975	42.52666787	14.53896465	Superficial	SOX2
FASTKD3	1.16065057	0.017811616	0.043066688	10.31174552	3.498409326	Superficial	SOX2
TATDN2	1.159747117	0.001883197	0.010234018	15.92499475	5.709440714	Superficial	SOX2
ZNF827	1.158149884	0.010644787	0.029507311	6.442233256	1.5745672	Superficial	SOX2
ENDOD1	1.157892731	0.001526458	0.009387421	35.09992903	11.74305729	Superficial	SOX2
SLC30A7	1.157881703	0.006166656	0.020290909	13.96798379	3.84775842	Superficial	SOX2
XBP1	1.155508829	0.003462059	0.014108599	57.3569173	22.63957265	Superficial	SOX2
STK26	1.15468951	0.000992312	0.008042817	31.7416801	8.213308058	Superficial	SOX2
BARX2	1.154142342	0.000317696	0.007176147	60.46456916	23.96402767	Superficial	SOX2 + KLF5, SOX2
APOBEC3C	1.150402917	0.002315114	0.011381143	31.44693812	10.14252514	Superficial	SOX2
TRIB1	1.147939362	0.001526458	0.016792744	28.23414165	9.735724133	Basal	SOX2
TP63	1.146853343	0.006166656	0.020290909	37.28071544	11.45048963	Superficial	SOX2 + KLF5, SOX2
SEMA3F	1.146811766	0.001883197	0.010234018	49.11393328	19.38989025	Superficial	SOX2
B4GALNT3	1.142848614	0.012681065	0.033478436	9.131762208	2.77867419	Superficial	SOX2 + KLF5, SOX2
ZCCHC2	1.141906951	0.000992312	0.008042817	16.46931387	4.382144031	Superficial	SOX2
RPS6KA3	1.139760282	0.001232925	0.008679903	37.84514834	11.68629436	Superficial	SOX2 + KLF5, SOX2
CD24	1.136471416	0.005105016	0.029792864	54.26310106	31.8283184	Basal	SOX2
FAM98A	1.136144885	0.000250281	0.007176147	32.20407289	10.52410277	Superficial	SOX2
PWWP2A	1.135767663	0.000119764	0.007176147	23.25985825	7.532852069	Superficial	SOX2
ROBO2	1.132994815	0.007423146	0.022959455	10.96655989	2.722355349	Superficial	SOX2 + KLF5
LEPROTL1	1.130920154	0.002836074	0.012662188	37.83090472	12.64397208	Superficial	SOX2

USP14	1.129592738	0.001526458	0.009387421	41.12020623	13.37419131	Superficial	SOX2
LONRF1	1.128697791	0.003462059	0.014108599	16.98832356	3.932101419	Superficial	SOX2
PTPN11	1.127491694	0.000250281	0.007176147	45.38995352	15.60369219	Superficial	SOX2
SLC44A1	1.124630481	0.010644787	0.029507311	30.70360064	9.008386975	Superficial	SOX2
PAPSS1	1.123121593	0.002315114	0.011381143	42.22052091	14.95779843	Superficial	SOX2
AMMECR1	1.123103887	0.000506425	0.007178523	40.51025441	13.19490157	Superficial	SOX2
ATG4D	1.120578518	0.001883197	0.010234018	42.99015922	16.54741472	Superficial	SOX2
FOXQ1	1.120308157	0.000795824	0.010838271	49.85377761	21.73787519	Suprabasal	SOX2 + KLF5, SOX2
DROSHA	1.120211159	0.002315114	0.011381143	12.39509232	3.912492171	Superficial	SOX2
DGCR2	1.119753322	0.000992312	0.008042817	36.05458181	11.88605041	Superficial	SOX2
NAT1	1.11949899	0.005105016	0.018023407	9.560135729	2.733991516	Superficial	SOX2
SNX7	1.118765388	0.000506425	0.007178523	24.582421	8.291960086	Superficial	SOX2
BCL2L2	1.118547819	0.002315114	0.011381143	33.03297089	10.55302431	Superficial	SOX2
OXNAD1	1.115216096	0.001232925	0.008679903	12.00572046	3.740562131	Superficial	SOX2
KANK1	1.115157148	0.003462059	0.014108599	45.85522332	16.13807382	Superficial	SOX2
FBXL18	1.11490085	0.008904627	0.025990329	18.8479122	5.558665317	Superficial	SOX2
TRIM4	1.114744712	0.000119764	0.007176147	21.61232033	6.44415365	Superficial	SOX2
DCTD	1.114599725	0.000992312	0.008042817	41.37982677	14.56407296	Superficial	SOX2
TMEM87A	1.112172671	9.30E-05	0.007176147	62.99294426	28.05497565	Superficial	SOX2
ACVR1	1.11208563	0.010644787	0.029507311	7.565015766	1.571333265	Superficial	SOX2, SOX2 + KLF5
PRPF8	1.110632241	0.001232925	0.008679903	43.91749476	15.59100667	Superficial	SOX2
SLC5A3	1.108546111	0.017811616	0.043066688	15.68467168	4.283838368	Superficial	SOX2 + KLF5
KNOP1	1.107463007	0.000635975	0.007274975	26.61624999	8.346682897	Superficial	SOX2
RASEF	1.106436035	0.002836074	0.012662188	33.89849056	10.29433174	Superficial	SOX2
ITPRIP	1.103478092	0.000119764	0.012455402	20.07023032	8.016875374	Basal	SOX2
AGAP1	1.103049375	0.004211401	0.016013117	10.92596213	3.220511929	Superficial	SOX2
LYPD3	1.10226506	0.001526458	0.009387421	86.62604201	62.41967361	Superficial	SOX2
CEBPB	1.100953174	0.001883197	0.010234018	59.35662314	25.73980246	Superficial	SOX2
EPHB6	1.098421093	0.002315114	0.011381143	23.20066096	6.510947152	Superficial	SOX2, SOX2 + KLF5
SMAD5	1.096440138	0.006166656	0.020290909	35.81411772	11.0476338	Superficial	SOX2
GBP6	1.096333706	0.000506425	0.007178523	71.41018052	33.62966856	Superficial	SOX2
ASCC3	1.096048273	0.000795824	0.007615337	35.83077768	11.24188818	Superficial	SOX2
FAT2	1.095564156	0.007423146	0.022959455	32.04126368	11.20764163	Superficial	SOX2
ECT2	1.095234017	0.004211401	0.021879278	15.76508345	5.983007448	Suprabasal	SOX2
ZBTB7C	1.095229116	0.006166656	0.020290909	37.09729954	12.38960308	Superficial	SOX2
ELK3	1.093367327	0.001883197	0.018415354	28.56296451	10.92497033	Basal	SOX2
CRYZ	1.092201992	0.001883197	0.010234018	21.73380901	5.586580444	Superficial	SOX2 + KLF5
PITPNC1	1.091116348	0.004211401	0.021879278	21.70312809	8.978402459	Suprabasal	SOX2
MYO1E	1.090802208	0.004211401	0.027185186	27.99278453	10.70128805	Basal	SOX2
LACTB2	1.090260768	0.001883197	0.010234018	20.41603957	5.557427606	Superficial	SOX2
ANXA8	1.088520498	0.006166656	0.020290909	37.31820819	12.19482701	Superficial	SOX2
PDIA3	1.087627963	0.000992312	0.008042817	71.74290452	35.69881232	Superficial	SOX2
EPAS1	1.087157497	0.007423146	0.030449933	48.81513341	24.82490815	Suprabasal	SOX2
EPHB4	1.086430382	0.002836074	0.012662188	8.61356527	3.03940643	Superficial	SOX2
FAM83A	1.084655702	0.000992312	0.014320968	24.79974942	11.00318279	Basal	SOX2, KLF5
PAQR5	1.084374053	0.000401828	0.012455402	11.68677813	4.523001064	Basal	SOX2
ACTR1B	1.083564297	0.002836074	0.012662188	31.0276409	10.4311608	Superficial	SOX2
ID1	1.083067962	0.008904627	0.025990329	67.99885112	36.38589517	Superficial	SOX2, KLF5
ITPR2	1.082561726	0.002315114	0.011381143	36.76971811	11.19914389	Superficial	SOX2, KLF5
TGFBR1	1.082552543	0.017811616	0.043066688	21.28035822	5.768770277	Superficial	SOX2 + KLF5
ELOVL5	1.081800374	0.004211401	0.021879278	35.05474557	15.29151468	Suprabasal	SOX2
HNRNPLL	1.081470571	0.000119764	0.007176147	19.51931021	5.953028379	Superficial	SOX2
CKMT1A	1.080797256	0.003462059	0.014108599	45.82714419	16.93959481	Superficial	KLF5
RTKN2	1.07996988	0.000401828	0.012455402	26.91170963	12.18065285	Basal	SOX2
IRF6	1.076699665	0.000635975	0.007274975	66.79817342	31.74169161	Superficial	SOX2
GJB6	1.074050502	0.010644787	0.029507311	72.67287901	34.07712441	Superficial	SOX2
ZNF276	1.073832933	0.004211401	0.016013117	21.6021014	8.143746	Superficial	SOX2
GJB3	1.073481421	0.001883197	0.010234018	52.3986983	20.43895374	Superficial	SOX2
ZDHHC24	1.073405826	0.005105016	0.018023407	31.33471846	10.87359273	Superficial	SOX2
TXLNA	1.072929136	0.007423146	0.022959455	26.7723363	9.668176579	Superficial	SOX2
GNPDA2	1.072787336	0.003462059	0.014108599	8.553457117	2.209436035	Superficial	SOX2
ATP7A	1.07034877	0.001526458	0.009387421	18.37821598	5.769978836	Superficial	SOX2
CDKN2B	1.070294963	0.012681065	0.049425568	18.07850545	8.341417478	Basal	SOX2
PCNX1	1.068627785	0.000795824	0.007615337	13.87619405	3.845484788	Superficial	SOX2
CCDC60	1.068090748	0.004173922	0.016013117	5.07941827	1.732237896	Superficial	SOX2
EML4	1.066487042	0.001232925	0.008679903	29.48556773	8.712000848	Superficial	SOX2
ZNF219	1.066374966	0.002836074	0.012662188	38.58342754	13.5543592	Superficial	SOX2
RIF1	1.06443131	0.004211401	0.016013117	24.59154314	6.647944401	Superficial	SOX2
CDC42EP4	1.064259537	0.000506425	0.010489143	28.86197028	11.84880536	Suprabasal	SOX2
HR	1.060194125	0.000635975	0.007274975	22.82513006	8.75455057	Superficial	SOX2, KLF5
WWP1	1.05999757	7.20E-05	0.007176147	45.17265309	16.34281943	Superficial	SOX2
BOLA2	1.059889855	0.012659085	0.033478436	27.52597963	8.841058943	Superficial	SOX2
HES2	1.059617182	0.000196466	0.012455402	47.53905383	23.28976828	Basal	SOX2
CDC42BPG	1.058014931	0.006166656	0.020290909	29.73744229	10.84726719	Superficial	SOX2
SQLE	1.057507387	0.010644787	0.029507311	53.21453026	22.40711233	Superficial	SOX2
PIGO	1.055646727	0.004211401	0.016013117	11.60956119	3.701016275	Superficial	SOX2
TADA3	1.055607235	0.000317696	0.007176147	40.42552055	15.3811476	Superficial	SOX2
PIK3C2B	1.054550473	0.001526458	0.009387421	23.97874975	8.155468379	Superficial	KLF5
ZNFX1	1.05419277	0.000992312	0.014320968	16.99829037	5.815432506	Basal	SOX2
SMG6	1.052156641	0.000506425	0.007178523	28.41227063	10.15996499	Superficial	SOX2

RAB11FIP2	1.051539922	0.004211401	0.016013117	12.72551013	2.863554896 Superficial	SOX2 + KLF5, SOX2
GALNT12	1.049379383	0.001232925	0.008679903	34.35833398	11.63233406 Superficial	SOX2
PDIK1L	1.048431055	0.006166656	0.020290909	11.5882273	3.088217267 Superficial	SOX2
FOXC1	1.047638776	0.017811616	0.043066688	15.52853274	7.61471716 Superficial	SOX2
COG6	1.04676455	0.001526458	0.009387421	18.12524566	6.012127181 Superficial	SOX2
CLDN4	1.046614095	0.003462059	0.024854712	53.00129205	29.07079485 Basal	SOX2, SOX2 + KLF5
TBC1D5	1.045495453	0.000506425	0.007178523	21.15279137	6.383257151 Superficial	SOX2
GBP2	1.043537437	0.006166656	0.020290909	43.075913	16.48742557 Superficial	SOX2, SOX2 + KLF5
CHCHD4	1.041517737	0.005105016	0.018023407	13.20831071	4.101917452 Superficial	SOX2
PIAS3	1.039484217	0.005105016	0.018023407	21.87521411	7.021571866 Superficial	SOX2
PDZRN3	1.038845423	0.005105016	0.018023407	9.730521189	3.580156724 Superficial	SOX2 + KLF5, SOX2
EPB41L5	1.038189591	0.001883197	0.010234018	32.11329554	10.05839763 Superficial	SOX2
TNKS	1.036998801	0.001883197	0.010234018	19.29376441	5.687144619 Superficial	SOX2
GJB5	1.036529192	0.012681065	0.033478436	50.72292565	17.65083088 Superficial	SOX2 + KLF5
MANF	1.032784742	0.003462059	0.014108599	68.1159678	32.72827979 Superficial	SOX2
HMGXB3	1.031603042	0.005105016	0.018023407	13.16907487	4.247519548 Superficial	SOX2
DLG1	1.030612623	0.000250281	0.007176147	59.02506663	26.05889543 Superficial	SOX2
ATAD2B	1.029425719	0.001526458	0.009387421	21.42384634	5.846551603 Superficial	SOX2
USP12	1.028326657	0.000506425	0.010489143	24.13745095	9.410233246 Suprabasal	SOX2, SOX2 + KLF5
USP42	1.027599754	0.001526458	0.009387421	11.22238109	3.728432416 Superficial	SOX2
APEX2	1.026096018	0.000992312	0.008042817	25.71451459	9.11992571 Superficial	SOX2
RAP2C	1.025837921	0.000401828	0.007176147	31.69093352	10.06563417 Superficial	SOX2
CGN	1.025734272	0.001526458	0.009387421	44.55844951	16.51730629 Superficial	SOX2
HCFC2	1.025545192	0.001526458	0.009387421	17.90303463	5.748783799 Superficial	SOX2
CLIP1	1.025361477	0.000250281	0.012455402	51.95265842	26.04386187 Basal	SOX2
SRFBP1	1.025231274	0.006166656	0.020290909	20.35835208	6.194081963 Superficial	SOX2
SFN	1.024113903	0.003462059	0.014108599	86.40801124	59.03093976 Superficial	SOX2
CHMP7	1.023040049	0.004211401	0.016013117	14.31554793	5.15490207 Superficial	SOX2
SEMA4C	1.020675853	0.000401828	0.012455402	13.60453169	4.878061834 Basal	SOX2
IMMT	1.020403361	0.007423146	0.022959455	41.15591597	13.86769913 Superficial	SOX2
MED27	1.016751381	0.001883197	0.010234018	20.7375588	6.37266373 Superficial	SOX2
STK39	1.016669337	0.001883197	0.018415354	21.59722621	8.154177809 Basal	SOX2
VCL	1.016433297	0.000250281	0.012455402	50.18358999	23.40486234 Basal	SOX2 + KLF5, SOX2
GLG1	1.014764777	0.000506425	0.007178523	38.66809769	13.84952322 Superficial	SOX2
DGKH	1.014239019	0.012681065	0.033478436	16.90556525	3.927563317 Superficial	SOX2
SPIRE2	1.012985199	0.010644787	0.038255467	4.88784887	2.320639171 Suprabasal	KLF5, SOX2
PDIA4	1.011720547	0.006166656	0.020290909	44.57312376	18.01306715 Superficial	SOX2
IPO8	1.011278306	0.002315114	0.011381143	19.10865866	6.340994881 Superficial	SOX2
WRNIP1	1.01058191	0.000401828	0.007176147	31.36699098	10.96769113 Superficial	SOX2
MRPL35	1.009994966	0.001526458	0.009387421	44.0994244	15.84466649 Superficial	SOX2
CYP2J2	1.009979358	0.002836074	0.012662188	39.69669461	14.93332307 Superficial	SOX2
SSBP2	1.008614228	0.003462059	0.014108599	34.56102056	10.69519446 Superficial	SOX2
EMP2	1.008286992	0.000401828	0.007176147	85.15076119	65.60610945 Superficial	SOX2
ELOVL1	1.007657584	0.002836074	0.012662188	67.73851337	30.2888522 Superficial	SOX2
WIZ	1.003348574	0.004211401	0.016013117	14.06167781	4.932187334 Superficial	SOX2
FLYWCH1	1.003246571	0.015054848	0.048140298	13.30169272	6.134460931 Suprabasal	SOX2
PANK3	1.002008835	0.005105016	0.018023407	42.2483754	14.84793779 Superficial	SOX2
ERBB2	1.000797318	0.003462059	0.014108599	64.90384468	29.36807898 Superficial	SOX2
GJB2	1.000707221	0.015054848	0.038009884	77.54819408	42.9288687 Superficial	SOX2
SREBF2	1.000004846	0.001526458	0.009387421	46.98351695	20.86267964 Superficial	SOX2
NAA35	0.999499739	0.000250281	0.007176147	24.88781225	7.308081533 Superficial	SOX2
OAS3	0.999411652	0.017811616	0.043066688	33.91694122	9.730762134 Superficial	SOX2
SLC4A2	0.998539121	0.000506425	0.007178523	17.02633306	5.480113383 Superficial	SOX2
RNF220	0.998062117	0.001526458	0.009387421	21.53668386	7.445293659 Superficial	SOX2
CYP3A5	0.997896563	0.008904627	0.025990329	33.40356	12.07692171 Superficial	SOX2
PREP	0.997620789	0.000506425	0.007178523	18.8332859	5.754453574 Superficial	SOX2
ANKRD50	0.997212216	0.006166656	0.020290909	10.84753741	3.542220569 Superficial	SOX2
SMARCAD1	0.996268912	0.007423146	0.022959455	14.35571326	4.114308749 Superficial	SOX2
CLASP2	0.995469306	0.021001058	0.04834985	17.84008933	5.495820276 Superficial	SOX2
CREB3	0.993048401	0.021001058	0.04834985	16.08875673	6.82847754 Superficial	SOX2
GSN	0.992984158	0.000250281	0.012455402	80.3245791	55.83838799 Basal	SOX2, KLF5
PLCH2	0.990452369	0.000317696	0.010489143	19.86664017	9.082930267 Suprabasal	SOX2
KDM1B	0.988968052	0.001883197	0.010234018	17.13891304	5.515185538 Superficial	SOX2
MAPKAPK3	0.985942266	0.000250281	0.007176147	61.16829182	27.56571044 Superficial	SOX2
N4BP3	0.982741775	0.000317696	0.012455402	13.99363767	5.703652307 Basal	SOX2
RAB3GAP2	0.981611807	0.000795824	0.007615337	30.71255762	10.52537581 Superficial	SOX2
CDC40	0.979458465	0.001526458	0.009387421	42.10328835	14.46730754 Superficial	SOX2
APOBEC3B	0.978993045	0.006166656	0.032981404	10.8000218	4.018199162 Basal	SOX2
ETV4	0.978127481	0.021001058	0.04834985	18.32034763	5.168240674 Superficial	SOX2
RBM27	0.976527696	0.000317696	0.007176147	41.41526646	14.85977794 Superficial	SOX2
VPS13A	0.975658933	0.001883197	0.010234018	29.64566246	9.650092596 Superficial	SOX2
TRIM29	0.975559011	0.003462059	0.014108599	83.4744379	60.80594581 Superficial	SOX2
CLNS1A	0.973230763	0.001526458	0.009387421	52.95854222	20.20355588 Superficial	SOX2
CLDN7	0.973223705	0.000119764	0.012455402	72.00569277	42.20990403 Basal	SOX2
NFIL3	0.973007118	0.000153669	0.010489143	31.80256845	14.76095525 Suprabasal	SOX2
OVOL1	0.972186252	0.004211401	0.027185186	36.62987761	17.67239326 Basal	SOX2
ARL6IP6	0.971918062	0.001883197	0.010234018	27.33317617	8.29542678 Superficial	SOX2
VLDLR	0.971565055	0.005105016	0.018023407	34.62065303	10.68412266 Superficial	SOX2
EFNB2	0.971028932	0.002315114	0.011381143	34.08986984	10.68114608 Superficial	SOX2 + KLF5, SOX2
C5orf22	0.964610162	0.002315114	0.011381143	14.12842584	4.040622089 Superficial	SOX2

SLC35F6	0.963323494	0.004211401	0.016013117	36.32587527	13.4031697	Superficial	SOX2
SBF1	0.962933302	0.000401828	0.012455402	18.97321274	7.722577967	Basal	SOX2
HEATR3	0.962903361	0.010644787	0.029507311	10.24391514	3.623009779	Superficial	SOX2
GRHL2	0.962221635	0.002836074	0.012662188	26.28896804	9.49884602	Superficial	SOX2
CCDC88C	0.960891309	0.008904627	0.025990329	14.82332483	4.120973742	Superficial	SOX2 + KLF5
OCLN	0.95992656	0.000250281	0.012455402	16.5872587	6.29292647	Basal	SOX2
GK	0.959674774	0.012681065	0.033478436	22.60421961	7.571280152	Superficial	SOX2
CASP1	0.957510724	0.000795824	0.007615337	26.91963947	8.607716575	Superficial	KLF5
PATJ	0.954789173	0.000119764	0.007176147	62.19275094	28.5356374	Superficial	SOX2
RBM12B	0.951788942	0.007423146	0.022959455	9.07536362	2.496305965	Superficial	SOX2
MBNL3	0.951778334	0.005105016	0.018023407	20.86518059	7.084354369	Superficial	SOX2 + KLF5, SOX2
GPHN	0.950352459	0.005105016	0.018023407	10.12383445	2.84515677	Superficial	SOX2 + KLF5
CLASP1	0.948346748	0.003462059	0.019729589	23.72656834	10.93478622	Suprabasal	SOX2
TJP1	0.947613883	0.000196466	0.012455402	59.43942815	31.52598771	Basal	SOX2
NTF4	0.947273158	0.012681065	0.042921737	5.706436766	2.210792774	Suprabasal	SOX2
ITPKC	0.947170762	0.002836074	0.022562695	44.35874301	23.17878921	Basal	SOX2
SNRNP200	0.945149806	0.000506425	0.007178523	38.43103539	14.67962995	Superficial	SOX2
LIMK2	0.943596112	0.002836074	0.012662188	38.02056013	14.07641227	Superficial	SOX2
IFI44	0.942373886	0.012681065	0.042921737	16.09657028	5.918626253	Suprabasal	SOX2
IFNGR1	0.941175231	0.004211401	0.016013117	33.31017628	11.24701455	Superficial	SOX2
TXNDC5	0.940628861	0.000795824	0.0136425	35.12375823	13.57082836	Basal	SOX2
PKP1	0.940526247	0.005105016	0.018023407	73.57168146	41.33040784	Superficial	SOX2 + KLF5, SOX2, KLF5
TM7SF2	0.940277008	0.003462059	0.014108599	74.58981573	44.97928738	Superficial	SOX2
ATXN1	0.939973269	0.008904627	0.025990329	30.66077976	9.153560369	Superficial	SOX2 + KLF5, SOX2
HYAL2	0.939193889	0.008904627	0.025990329	25.45374355	9.165485301	Superficial	SOX2
PIR	0.938528782	0.010644787	0.029507311	42.51794111	15.60827055	Superficial	SOX2
SRCAP	0.937080417	0.004211401	0.021879278	23.12213982	10.22666155	Suprabasal	SOX2
KDM5A	0.936624009	0.000401828	0.007176147	52.87431596	22.545332	Superficial	SOX2
HIPK2	0.936277725	0.000795824	0.007615337	44.33080382	16.53588677	Superficial	SOX2
XPR1	0.935980881	0.008904627	0.034252903	13.83697012	5.461275977	Suprabasal	SOX2
C11orf80	0.934813283	0.001883197	0.010234018	19.61554888	7.757036142	Superficial	SOX2
TLCD1	0.934508989	0.008904627	0.025990329	26.94830138	9.425029458	Superficial	SOX2
IL15	0.931288218	0.002836074	0.0176927	10.39551987	3.705311357	Suprabasal	SOX2, SOX2 + KLF5
IGF1R	0.930404525	0.003462059	0.019729589	35.5263818	16.47930112	Suprabasal	SOX2
VPS16	0.930332913	0.003462059	0.014108599	23.27652963	9.006523619	Superficial	SOX2
PARN	0.930213959	0.015054848	0.038009884	13.26146873	4.826815714	Superficial	SOX2
IFIH1	0.930212135	0.007423146	0.030449933	37.56081844	17.67361083	Suprabasal	SOX2 + KLF5
MLKL	0.929694873	0.007423146	0.022959455	13.79556589	5.302842768	Superficial	SOX2
BCL6	0.929155584	0.004211401	0.021879278	25.26670431	10.89606288	Suprabasal	SOX2
SULF2	0.926816247	0.004211401	0.021879278	56.00061141	30.06269751	Suprabasal	SOX2
SPRY2	0.92469581	0.003462059	0.019729589	9.968966492	3.716915077	Suprabasal	SOX2
RPUSD3	0.921826912	0.004211401	0.016013117	36.06544029	14.38430226	Superficial	SOX2
TNKS1BP1	0.921810736	0.000401828	0.007176147	43.2456963	17.19199187	Superficial	SOX2
METTL16	0.921425744	0.015054848	0.038009884	10.71836209	3.663641463	Superficial	SOX2
THUMPD1	0.921027525	0.000795824	0.007615337	35.43257965	11.99410572	Superficial	SOX2
MORC4	0.919114825	0.021001058	0.04834985	19.58222702	6.690165479	Superficial	SOX2
SMPD3	0.91883153	0.012681065	0.033478436	9.692248683	3.781776836	Superficial	SOX2, KLF5, SOX2 + KLF5
CTSS	0.918754346	0.003462059	0.019729589	58.74895434	32.65659344	Suprabasal	SOX2
DDX23	0.918262459	0.000635975	0.007274975	33.28708232	12.49172321	Superficial	SOX2
DNAJC11	0.918087484	0.005105016	0.018023407	17.65883872	6.07592926	Superficial	SOX2
CHD1	0.916971655	0.002315114	0.011381143	29.52238981	10.04115794	Superficial	SOX2
NUP133	0.914397334	0.015054848	0.038009884	13.23558916	4.066194821	Superficial	SOX2
ZNF532	0.913554972	0.008904627	0.034252903	14.08271725	5.827776916	Suprabasal	SOX2
ANK3	0.913274645	0.012681065	0.033478436	32.92849736	10.82374859	Superficial	SOX2 + KLF5
PEX26	0.911924325	0.021001058	0.04834985	17.34632142	5.907570971	Superficial	SOX2
RTCB	0.910029323	0.000506425	0.007178523	64.11821594	31.35594231	Superficial	SOX2
AFG3L2	0.909662689	0.001526458	0.009387421	45.0308695	18.25711922	Superficial	SOX2
UBE3C	0.908984058	0.002315114	0.011381143	28.28995982	9.861957868	Superficial	SOX2
SCEL	0.908585979	0.004211401	0.027185186	35.21521921	18.03409494	Basal	SOX2
CCAR1	0.908014534	0.008904627	0.025990329	41.8177982	14.38612398	Superficial	SOX2
TNFSF10	0.906889478	0.001526458	0.016792744	90.0032318	67.96705726	Basal	SOX2, KLF5
SLC45A4	0.906108969	0.008904627	0.040392504	8.764812014	2.929838771	Basal	SOX2
MAGED2	0.905063097	0.000635975	0.007274975	36.3342794	13.70758675	Superficial	SOX2
BCR	0.904694009	0.000795824	0.0136425	21.22696209	8.213040475	Basal	SOX2
ITPR3	0.90283063	0.001526458	0.016792744	27.31887442	11.01249269	Basal	SOX2
ARHGEF5	0.902031503	0.004211401	0.021879278	49.08157174	27.73966165	Suprabasal	SOX2
TBC1D10A	0.901080387	0.004211401	0.016013117	31.31177269	11.81774667	Superficial	SOX2
ANKIB1	0.900545456	0.000153669	0.007176147	37.04964394	13.30203144	Superficial	SOX2
SLC4A7	0.899999926	0.017811616	0.043066688	6.29061434	1.861275296	Superficial	SOX2 + KLF5
FXYD3	0.899656014	0.002315114	0.011381143	87.03589411	66.84367355	Superficial	SOX2
CDKAL1	0.89933994	0.000506425	0.007178523	11.61467749	3.791144178	Superficial	SOX2
FURIN	0.899232364	0.000153669	0.012455402	17.94979085	6.915633525	Basal	SOX2
CAMSAP1	0.899201892	0.000119764	0.012455402	33.88678212	15.33563619	Basal	SOX2
PHC2	0.896625623	0.001883197	0.010234018	17.54207804	6.22869448	Superficial	SOX2
LRRC42	0.895968102	0.001232925	0.008679903	32.72943189	11.61962184	Superficial	SOX2
SLC39A6	0.895816619	0.006166656	0.020290909	16.16572683	4.572418678	Superficial	SOX2
ST14	0.89536654	7.20E-05	0.012455402	50.40009109	25.34790976	Basal	SOX2, SOX2 + KLF5
PLEKHG3	0.895015137	0.015054848	0.038009884	13.95511449	4.823953346	Superficial	SOX2
TIAM1	0.894450063	0.000992312	0.008042817	54.68463967	24.04975501	Superficial	SOX2
KIAA1217	0.893786401	0.000992312	0.008042817	53.56579201	21.83646765	Superficial	SOX2

SPECC1L	0.893320652	0.001232925	0.015721789	24.77042245	9.815567676	Basal	SOX2
EPN2	0.893135704	0.000795824	0.007615337	63.41211342	30.86327485	Superficial	SOX2 + KLF5
BMPR2	0.892138295	0.001883197	0.010234018	38.46729266	14.761013	Superficial	SOX2, SOX2 + KLF5
CDCP1	0.890652929	0.000992312	0.008042817	66.76017312	32.69936178	Superficial	SOX2
TNRC18	0.889205782	0.017811616	0.043066688	24.24592989	8.527045348	Superficial	SOX2 + KLF5, SOX2
TOMM70	0.887267944	0.003462059	0.014108599	30.88206662	10.68652037	Superficial	SOX2
KCNK7	0.886984141	0.012681065	0.033478436	31.28106222	10.82219727	Superficial	SOX2
EPHB3	0.885669184	0.001526458	0.016792744	17.94114574	7.389341179	Basal	SOX2, KLF5
BMPR1A	0.882293181	0.003462059	0.014108599	12.01608827	3.634607464	Superficial	SOX2
TRAF3IP1	0.882212095	0.005105016	0.018023407	14.53927799	4.312759643	Superficial	SOX2
NOL10	0.882088665	0.015054848	0.038009884	17.02148532	6.188813952	Superficial	SOX2 + KLF5, SOX2
RNF19B	0.881491342	0.000506425	0.012898798	25.32034955	10.32639443	Basal	SOX2 + KLF5, SOX2
UGCG	0.880956022	0.000317696	0.012455402	41.6884798	18.93866914	Basal	SOX2, SOX2 + KLF5
ABLIM2	0.87843226	0.001232925	0.012246384	21.47830619	10.05916517	Suprabasal	SOX2
CEP170B	0.878140221	0.000401828	0.012455402	19.88590991	7.313422792	Basal	SOX2
MORC2	0.87731019	0.001526458	0.009387421	11.36270155	3.478454545	Superficial	SOX2
FTSJ3	0.876905147	0.000635975	0.007274975	19.33425643	6.629415989	Superficial	SOX2
NOTCH1	0.876227202	0.003462059	0.024854712	28.32956634	13.11437681	Basal	SOX2
ZDHHC16	0.875936954	0.008904627	0.025990329	18.0535891	6.514104604	Superficial	SOX2
IGSF3	0.875568181	0.004211401	0.016013117	20.64854284	7.060117599	Superficial	SOX2
METTL14	0.875376042	0.002836074	0.012662188	20.15456999	6.39679254	Superficial	SOX2
HDGF	0.875145778	0.000506425	0.007178523	63.33322983	30.12978082	Superficial	SOX2
BTG1	0.874641303	7.20E-05	0.007176147	76.33575407	46.98814185	Superficial	SOX2, SOX2 + KLF5
CHRAC1	0.874329147	0.001526458	0.009387421	32.58122904	11.03633825	Superficial	SOX2
PPP1R26	0.874196498	0.000795824	0.010838271	9.010442668	3.864186864	Suprabasal	SOX2
CAMSAP3	0.873243075	0.001232925	0.015721789	12.6460338	5.306066422	Basal	SOX2
TP53RK	0.873165537	0.001883197	0.010234018	34.69402433	13.34150254	Superficial	SOX2
LETM1	0.872109216	0.000635975	0.007274975	48.00505357	21.60553929	Superficial	SOX2
RBM19	0.870734356	0.008904627	0.025990329	9.455645202	3.177116214	Superficial	SOX2
NOP14	0.870040844	0.012681065	0.033478436	17.67069829	5.687473923	Superficial	SOX2
SLC25A46	0.869726759	0.010644787	0.029507311	27.18592165	9.768766573	Superficial	SOX2
NLRX1	0.869680205	0.006166656	0.020290909	44.2626525	19.65292128	Superficial	SOX2
ADCK2	0.869583124	0.007423146	0.022959455	21.60813141	8.531418818	Superficial	SOX2
RAB3D	0.86952271	0.015054848	0.038009884	36.43417123	14.40115223	Superficial	SOX2
HNRNPAB	0.868905205	0.002836074	0.012662188	55.3602438	22.22158232	Superficial	SOX2
CSNK1G3	0.868108155	0.000635975	0.007274975	25.97849208	8.715818884	Superficial	SOX2
PLXNA2	0.867985641	0.006166656	0.020290909	29.09724384	8.662493629	Superficial	SOX2
POLR3A	0.867854544	0.017811616	0.043066688	9.674285364	2.890829878	Superficial	SOX2
PLOD2	0.867405121	0.003462059	0.014108599	24.23100976	7.329784954	Superficial	SOX2
CLOCK	0.867335537	0.015054848	0.038009884	35.97663274	12.96369281	Superficial	SOX2
VRK2	0.866624796	0.002836074	0.012662188	27.63407787	9.880195058	Superficial	SOX2
MTA2	0.866164613	0.017811616	0.043066688	24.48027662	10.40104993	Superficial	SOX2
IRF1	0.864606905	7.20E-05	0.012455402	66.36951881	38.12897296	Basal	SOX2
SPSB2	0.863671948	0.021001058	0.04834985	12.26063796	4.552374875	Superficial	SOX2
UNC45A	0.863347383	0.008904627	0.025990329	30.21492431	11.95655965	Superficial	SOX2
FAM110C	0.863318044	0.000401828	0.010489143	49.1183436	27.19016149	Suprabasal	SOX2, SOX2 + KLF5
RNF214	0.860639186	0.000992312	0.008042817	20.37905803	7.210504233	Superficial	SOX2
NLN	0.860495589	0.002836074	0.012662188	10.91799313	3.618501469	Superficial	SOX2
NSMAF	0.858277222	0.012681065	0.033478436	11.88905994	4.845257228	Superficial	SOX2
TPST1	0.858189681	0.000992312	0.01142308	22.00115385	9.487918828	Suprabasal	SOX2
MCMBP	0.856648759	0.000401828	0.007176147	28.42016649	10.86479165	Superficial	SOX2
OSBP	0.855633877	0.006166656	0.020290909	40.09049547	16.42969957	Superficial	SOX2
FAS	0.855309759	0.000196466	0.010489143	28.48938444	12.96714597	Suprabasal	SOX2
LUZP1	0.855211261	0.000196466	0.007176147	37.10983106	13.77168569	Superficial	SOX2
SLC10A3	0.855190282	0.008904627	0.025990329	24.99035341	9.877241285	Superficial	SOX2
PDCD4	0.852743281	0.005105016	0.018023407	76.60735903	42.69342999	Superficial	SOX2
PHF14	0.852521794	0.002836074	0.012662188	29.36397933	9.593443001	Superficial	SOX2
HDAC1	0.851318097	0.000992312	0.008042817	61.95501145	30.23757095	Superficial	SOX2
FCHSD2	0.850963554	0.002836074	0.0176927	8.91420517	4.048490828	Suprabasal	SOX2 + KLF5
ERCC3	0.849248528	0.008904627	0.025990329	12.5092704	4.311489353	Superficial	SOX2
DMRTA2	0.848884765	0.002315114	0.016074975	6.741522978	3.19563976	Suprabasal	SOX2
ALCAM	0.848190649	0.007423146	0.022959455	11.32639601	3.871626927	Superficial	SOX2 + KLF5, SOX2
SKIV2L	0.848108426	0.000506425	0.007178523	13.76146259	4.464769501	Superficial	SOX2
DSC2	0.846921441	0.017811616	0.043066688	72.26288159	40.41740992	Superficial	SOX2
DTNBP1	0.846794959	0.021001058	0.04834985	14.28105129	4.932464707	Superficial	SOX2 + KLF5
BORCS5	0.846268858	0.002836074	0.012662188	20.25345521	7.504594465	Superficial	SOX2
POLE	0.846155403	0.002836074	0.0176927	7.470452375	3.782665953	Suprabasal	SOX2
MAP3K8	0.845745236	0.004211401	0.027185186	25.65143559	12.18378813	Basal	SOX2, SOX2 + KLF5
RUFY3	0.844831691	0.000635975	0.007274975	44.04485076	16.50861145	Superficial	SOX2
ITGA3	0.844554837	0.007423146	0.030449933	13.76692359	6.266478229	Suprabasal	SOX2
ARFGEF2	0.844127629	0.002315114	0.011381143	41.32971032	15.23832937	Superficial	SOX2
SPNS1	0.842942886	0.003462059	0.014108599	21.89951967	7.858241511	Superficial	SOX2
UBR5	0.842481048	0.006166656	0.020290909	41.02552479	15.33512244	Superficial	SOX2
ALG9	0.839853938	0.007423146	0.022959455	10.62872224	4.26325388	Superficial	SOX2
MTHFD1L	0.839822411	0.007423146	0.036548425	14.53056637	6.246920981	Basal	SOX2
CHD4	0.839801259	0.000635975	0.007274975	57.16082184	26.07656657	Superficial	SOX2
OAF	0.839505259	0.003462059	0.019729589	13.15030459	5.79206902	Suprabasal	SOX2
MAP4	0.839469094	0.000506425	0.007178523	56.55506459	24.597045	Superficial	SOX2
GLE1	0.835935781	0.008904627	0.025990329	16.91032054	5.336535038	Superficial	SOX2
NECTIN4	0.835610283	0.000635975	0.012925379	30.87073029	15.59987036	Basal	SOX2

IWS1	0.83526127	0.001526458	0.009387421	33.72332735	12.23744966	Superficial	SOX2
RBM12	0.834785213	0.001232925	0.008679903	23.96776883	8.159807832	Superficial	SOX2
CCDC71L	0.834591944	0.006166656	0.020290909	18.69169873	6.190759114	Superficial	SOX2
MACC1	0.834417685	0.001883197	0.018415354	24.81749513	10.86425508	Basal	SOX2 + KLF5, SOX2
HES1	0.834394053	0.015054848	0.038009884	72.77617354	40.31242283	Superficial	SOX2
DNMBP	0.834004899	0.017811616	0.043066688	12.19837134	4.683669686	Superficial	SOX2 + KLF5
EFL1	0.833887149	0.002836074	0.012662188	12.54418382	4.708387837	Superficial	SOX2
YLPM1	0.833877819	0.004211401	0.016013117	21.20338158	7.420526934	Superficial	SOX2
PHC3	0.833132444	0.021001058	0.04834985	15.7324662	4.172159185	Superficial	SOX2
ARHGAP1	0.832801399	0.001526458	0.009387421	49.16523241	21.6705731	Superficial	SOX2
MTMR4	0.832773168	0.012681065	0.033478436	12.61282616	4.384877816	Superficial	SOX2
USP34	0.831927479	0.000250281	0.007176147	48.76179144	19.59301972	Superficial	SOX2
LTN1	0.831844928	0.003462059	0.014108599	32.27681339	10.69037507	Superficial	SOX2
SAMD8	0.83084174	0.007423146	0.022959455	30.77720131	12.43241064	Superficial	SOX2
LTBP4	0.830027435	0.000795824	0.010838271	42.65305007	22.45104814	Suprabasal	SOX2
EFNB1	0.828770052	0.007423146	0.022959455	12.44857866	4.371969674	Superficial	SOX2
TAF15	0.827829113	0.002836074	0.012662188	46.64494557	17.66507047	Superficial	SOX2
ZNF205	0.827619182	0.006166656	0.020290909	9.321767583	3.501849539	Superficial	SOX2
DSC3	0.82685136	0.021001058	0.04834985	60.40379599	27.05844558	Superficial	SOX2 + KLF5, SOX2
TDG	0.826493209	0.005105016	0.018023407	45.1714113	18.47106601	Superficial	SOX2
PPA2	0.825614967	0.000795824	0.007615337	45.85643088	18.36050404	Superficial	SOX2
EPB41L2	0.825187272	0.012681065	0.042921737	13.2310882	5.383598174	Suprabasal	SOX2
RHOV	0.824745801	0.000506425	0.012898798	55.32474773	31.36023103	Basal	SOX2
THOC3	0.823897476	0.010644787	0.029507311	59.47882876	28.67750392	Superficial	SOX2
TWF2	0.823549219	0.000992312	0.008042817	58.15254953	27.21826086	Superficial	SOX2 + KLF5
ATRN	0.823194799	0.021001058	0.04834985	17.37967553	6.135836441	Superficial	SOX2
PPL	0.822801611	0.000317696	0.012455402	57.78159745	33.11426547	Basal	SOX2, SOX2 + KLF5
FBXO38	0.822800065	0.000992312	0.008042817	19.93874301	6.63278819	Superficial	SOX2
MRPS35	0.82271128	0.012681065	0.033478436	41.47677578	15.25102828	Superficial	SOX2
ACAD9	0.822605788	0.000401828	0.007176147	36.21095613	14.39931058	Superficial	SOX2 + KLF5
SNX12	0.822585602	0.006166656	0.020290909	25.06938895	11.27638862	Superficial	SOX2
TFAP2A	0.820450997	0.002315114	0.011381143	42.82071234	17.82890683	Superficial	SOX2
ATN1	0.819507951	0.000992312	0.008042817	32.18520156	12.76600372	Superficial	SOX2
CPT2	0.818968522	0.015054848	0.038009884	23.64381963	8.697933431	Superficial	SOX2
SPTLC2	0.817816947	0.008904627	0.025990329	58.58796534	29.63327216	Superficial	SOX2
HACE1	0.816745618	0.008904627	0.034252903	13.23797985	6.170834204	Suprabasal	SOX2
PIM1	0.816682613	0.000506425	0.012898798	35.4644167	17.60863066	Basal	SOX2 + KLF5, SOX2
DHX16	0.816455155	0.000992312	0.008042817	20.49134581	7.234232442	Superficial	SOX2
DNAAF2	0.815990058	0.008904627	0.025990329	12.88862781	5.011421676	Superficial	SOX2
SDC1	0.813719129	0.002315114	0.011381143	87.75694466	66.95145226	Superficial	SOX2
ELL3	0.813317704	0.015054848	0.048140298	7.192728655	3.329046936	Suprabasal	SOX2
CCNE1	0.812722988	0.001883197	0.018415354	11.8773326	5.413782807	Basal	SOX2
PLXNB2	0.812108846	0.001232925	0.015721789	46.65187659	22.95899987	Basal	SOX2
WDR47	0.811734977	5.55E-05	0.012455402	35.21788058	15.80573817	Basal	SOX2
MID1IP1	0.810782697	0.002836074	0.012662188	33.06994348	13.45685532	Superficial	SOX2
MFN1	0.810406977	0.000795824	0.007615337	37.4800731	14.78617492	Superficial	SOX2
EFNA4	0.809859537	0.003462059	0.014108599	21.55207442	7.257920127	Superficial	SOX2
ARMCX3	0.808889184	0.001883197	0.010234018	22.49931016	7.20222225	Superficial	SOX2
DDX46	0.808617792	0.001883197	0.010234018	55.8352134	23.18074829	Superficial	SOX2
NXF1	0.808612993	0.021001058	0.04834985	18.32360737	7.616265295	Superficial	SOX2
NDUFV1	0.808605636	0.008904627	0.025990329	58.69079218	25.16361058	Superficial	SOX2
KIF1C	0.808132244	0.000401828	0.012455402	35.31714962	16.56280017	Basal	SOX2
CDKN2D	0.807747808	0.017811616	0.043066688	31.18476328	12.50599597	Superficial	SOX2
TMED5	0.807701462	0.001883197	0.010234018	40.71109999	16.55487528	Superficial	SOX2
ANKRD22	0.804461386	0.001526458	0.009387421	53.90767745	23.26610733	Superficial	SOX2
MCPH1	0.800090408	0.012681065	0.033478436	17.43680128	6.513088304	Superficial	SOX2
FNDC3A	0.800001038	0.004211401	0.016013117	20.19240284	7.23045575	Superficial	SOX2
SIRT1	0.799938477	0.007423146	0.022959455	14.80977543	4.692575729	Superficial	SOX2
LRPPRC	0.798944589	0.021001058	0.04834985	31.40078505	9.816116635	Superficial	SOX2
ATP5F1A	0.798738249	0.006166656	0.020290909	71.23746796	38.86356897	Superficial	SOX2
CPSF2	0.798423626	0.000992312	0.008042817	34.48820073	13.36730562	Superficial	SOX2
UBALD2	0.798313434	0.000250281	0.012455402	58.45055428	32.53669687	Basal	SOX2
SFPQ	0.797818023	0.001232925	0.008679903	61.97537887	29.57785022	Superficial	SOX2
SLC52A2	0.797173775	0.010644787	0.029507311	28.79074487	10.90435535	Superficial	SOX2
NFRKB	0.797033244	0.004211401	0.016013117	12.0529207	4.844985206	Superficial	SOX2
ABHD13	0.796556468	0.000992312	0.008042817	22.41406526	8.264951864	Superficial	SOX2
ARHGAP21	0.795336566	0.004211401	0.021879278	36.03350368	18.17611891	Suprabasal	SOX2
EVPL	0.793544001	0.000795824	0.0136425	44.99753962	23.63771042	Basal	SOX2
MSH3	0.793458301	0.003462059	0.014108599	22.98398105	7.918764414	Superficial	SOX2
ARL1	0.79214833	0.001232925	0.008679903	71.80733536	42.39486522	Superficial	SOX2
KCTD1	0.790819901	0.000795824	0.007615337	50.90920202	21.19738831	Superficial	SOX2, SOX2 + KLF5
FAM199X	0.790489698	0.000992312	0.008042817	33.68678037	12.41133265	Superficial	SOX2
IPPK	0.790086212	0.021001058	0.04834985	32.36939208	11.4416446	Superficial	SOX2
GRIPAP1	0.790033602	0.000992312	0.008042817	32.41525033	11.48465582	Superficial	SOX2
IGSF8	0.789619705	0.002315114	0.011381143	14.91047128	5.385316998	Superficial	SOX2
SMARCE1	0.788128386	0.000401828	0.007176147	53.98102131	23.8797572	Superficial	SOX2
PAK1	0.786995191	0.000635975	0.007274975	29.64145241	11.08264459	Superficial	SOX2
SLC22A18	0.786650694	0.000992312	0.008042817	27.90101417	9.786049759	Superficial	SOX2
SCRIB	0.785180723	0.000317696	0.012455402	24.07048471	10.71812052	Basal	SOX2
PPP2R1B	0.785024498	0.002836074	0.012662188	28.21036033	10.75997223	Superficial	SOX2

KLK10	0.784915262	0.001883197	0.018415354	49.63143813	30.40373894	Basal	SOX2
ESRP2	0.784736975	0.003462059	0.014108599	49.49809927	23.21995429	Superficial	SOX2
DSP	0.78419116	0.000506425	0.012898798	95.57120634	83.16967388	Basal	SOX2, SOX2 + KLF5
SUPT6H	0.783663486	0.001883197	0.010234018	39.09967722	17.04241278	Superficial	SOX2
NSD1	0.782623544	0.000250281	0.007176147	44.34785098	18.99005533	Superficial	SOX2
RARG	0.781083073	0.000401828	0.007176147	76.26745981	42.47873787	Superficial	SOX2
RASSF6	0.780974466	0.021001058	0.04834985	19.09196449	5.374914478	Superficial	SOX2, SOX2 + KLF5
TRIM5	0.780767742	0.021001058	0.04834985	14.72009644	4.341097256	Superficial	SOX2
ARHGAP35	0.779858557	0.000795824	0.007615337	30.72874296	11.10752697	Superficial	SOX2
TBRG4	0.777140895	0.017811616	0.043066688	23.73424314	8.528389369	Superficial	SOX2
TRABD	0.776314911	0.006166656	0.020290909	44.64577932	19.65405638	Superficial	SOX2
ARMCX6	0.776127211	0.001883197	0.010234018	31.53888816	12.22350712	Superficial	SOX2
NCOA5	0.775988711	0.008904627	0.025990329	9.113392929	3.455771311	Superficial	SOX2
FRMD4B	0.772823978	0.001526458	0.009387421	72.27208121	40.58876961	Superficial	SOX2
LAP3	0.771547699	0.001883197	0.010234018	40.8294852	15.63526895	Superficial	SOX2
CDC42BPB	0.770069085	0.000153669	0.012455402	39.33363482	18.95615945	Basal	SOX2
SLC7A8	0.768503851	0.003462059	0.014108599	23.18110177	9.569188016	Superficial	SOX2
TMEM154	0.766978768	0.010644787	0.029507311	73.6246979	44.77279121	Superficial	SOX2
TXN	0.766068419	0.017811616	0.043066688	94.92987078	90.59413398	Superficial	SOX2
CAAP1	0.765982227	0.001232925	0.008679903	27.8715948	10.35073339	Superficial	SOX2
ZNF407	0.765275288	0.005105016	0.018023407	9.565013872	2.98368203	Superficial	SOX2
TAB1	0.765243129	0.008904627	0.025990329	7.66350226	2.519377628	Superficial	SOX2
VILL	0.765232309	0.010644787	0.038255467	12.66776283	5.055959665	Suprabasal	KLF5
TPM4	0.765005693	0.002836074	0.022562695	83.37863428	60.677827	Basal	SOX2
UTP6	0.764059543	0.001883197	0.010234018	24.32650647	9.796163309	Superficial	SOX2
ZNF142	0.763854846	0.000795824	0.010838271	7.199945873	3.709384109	Suprabasal	SOX2
USP7	0.762927097	0.000506425	0.007178523	57.37381352	26.69855543	Superficial	SOX2
TKFC	0.762788105	0.021001058	0.04834985	15.27432299	5.605121704	Superficial	SOX2
TTC17	0.761819095	0.008904627	0.025990329	24.36359453	8.844420798	Superficial	SOX2
PXK	0.758536609	0.005105016	0.018023407	14.76121213	4.158985349	Superficial	SOX2
NMB	0.757007026	0.003462059	0.024854712	19.49023009	8.735687673	Basal	SOX2
PIGK	0.75685835	0.010644787	0.029507311	10.61173633	3.827762891	Superficial	SOX2
PRPF40A	0.755048603	0.000795824	0.007615337	58.42226805	27.22883402	Superficial	SOX2
RIPK4	0.753786917	0.000506425	0.010489143	56.92429997	34.29619525	Suprabasal	SOX2
MARCKSL1	0.753039523	0.005105016	0.029792864	19.57397271	7.984022926	Basal	SOX2
COX10	0.75216747	0.017811616	0.043066688	15.53587995	5.187538716	Superficial	SOX2
XRN2	0.751931606	0.008904627	0.025990329	38.10142195	14.27251888	Superficial	SOX2
POR	0.751814117	0.000992312	0.008042817	66.781586	35.01139862	Superficial	KLF5, SOX2
MECOM	0.750699729	0.002315114	0.016074975	56.52334109	32.74640448	Suprabasal	KLF5
PLP2	0.749774242	0.010644787	0.029507311	77.34020689	49.58391191	Superficial	SOX2
RAB11FIP4	0.74958562	0.002836074	0.022562695	11.93939559	4.465728883	Basal	SOX2
CRADD	0.748667913	0.007423146	0.022959455	14.26033086	4.924503899	Superficial	SOX2 + KLF5
ATXN7L3B	0.748484548	0.000153669	0.007176147	44.03819642	18.55042579	Superficial	SOX2
GRIP1	0.747835615	0.003462059	0.019729589	7.134486236	3.06073658	Suprabasal	SOX2 + KLF5
NIPAL1	0.747293766	0.001883197	0.010234018	58.7054824	29.04320719	Superficial	SOX2
RASA1	0.747100481	0.003462059	0.014108599	25.67635873	8.934911271	Superficial	SOX2
MFSD6	0.746746359	0.012681065	0.033478436	53.60128769	24.41723396	Superficial	SOX2
INTS2	0.746668773	0.010644787	0.029507311	8.778939305	2.442925882	Superficial	SOX2
CCDC34	0.746367735	0.001526458	0.013290882	40.02681251	19.40977035	Suprabasal	SOX2
UBR7	0.74631484	0.012681065	0.033478436	16.05246902	5.706209921	Superficial	SOX2
SNTB2	0.745802206	0.001883197	0.010234018	27.06126241	9.939822852	Superficial	SOX2
MAN1A2	0.744280933	0.001883197	0.010234018	50.53089819	20.95246014	Superficial	SOX2
NDUFA8	0.743813982	0.004211401	0.016013117	56.18648971	26.12503468	Superficial	SOX2
EIF3B	0.74245088	0.005105016	0.018023407	43.13425729	17.85244483	Superficial	SOX2
POLR3B	0.74062025	0.000506425	0.007178523	21.82467954	8.464220903	Superficial	SOX2
WNK4	0.739616678	0.008904627	0.025990329	16.96339688	6.600647357	Superficial	SOX2
MRPS30	0.739498814	0.010644787	0.029507311	26.85606255	9.562664138	Superficial	SOX2
RBBP8NL	0.738379695	0.008904627	0.025990329	18.58587441	7.808829489	Superficial	SOX2
ARFGAP3	0.737414745	0.004211401	0.016013117	34.43334593	13.93679235	Superficial	SOX2 + KLF5
PRRC2B	0.737288087	0.004211401	0.016013117	37.54429978	15.16190471	Superficial	SOX2
CLIC4	0.737162028	0.007423146	0.036548425	18.68317984	8.50586959	Basal	SOX2
AFDN	0.736654672	5.55E-05	0.012455402	48.38939108	25.14959746	Basal	SOX2
PHF13	0.73612879	0.000635975	0.012925379	18.94767185	8.609546677	Basal	SOX2
ZYX	0.735779973	0.015054848	0.038009884	36.28202793	16.07075639	Superficial	SOX2
TMEM98	0.735476257	0.008904627	0.034252903	30.33653877	14.52821983	Suprabasal	SOX2
BICDL2	0.735059923	0.002315114	0.020052792	22.07254004	10.57748777	Basal	SOX2
PRC1	0.734220942	0.002836074	0.022562695	44.06968575	25.03313541	Basal	SOX2
ZNF131	0.733472982	0.002315114	0.011381143	25.08109627	8.963884002	Superficial	SOX2
PIK3R1	0.733066436	0.007423146	0.022959455	19.32332041	6.742500994	Superficial	SOX2
BST2	0.732636317	0.012681065	0.049425568	41.46014877	20.02884409	Basal	SOX2
MGST3	0.732351335	0.010644787	0.029507311	65.51241176	32.75084606	Superficial	SOX2
HTATIP2	0.732198546	0.005105016	0.018023407	54.11311985	24.77233011	Superficial	SOX2
ZPR1	0.730854063	0.017811616	0.043066688	28.17455576	10.73635346	Superficial	SOX2
URI1	0.730252363	0.000795824	0.007615337	48.99660551	20.34154253	Superficial	SOX2
ADD3	0.729947864	0.002315114	0.011381143	66.28396552	34.34038086	Superficial	KLF5
EZR	0.72742657	0.002315114	0.020052792	82.97696886	59.68243158	Basal	SOX2 + KLF5, SOX2
TMEM184A	0.726810155	0.006166656	0.032981404	16.76278201	7.803146974	Basal	SOX2
GRB7	0.726375743	0.000317696	0.012455402	39.18241856	19.55517725	Basal	SOX2
MRPL4	0.726193947	0.008904627	0.025990329	43.49259063	17.67791629	Superficial	SOX2
AKAP13	0.72614015	0.000635975	0.007274975	69.84387281	38.37719104	Superficial	SOX2

ICMT	0.726052153	0.000992312	0.014320968	39.13219758	20.3780701	Basal	SOX2
G3BP1	0.72578975	0.017811616	0.043066688	45.8882534	18.82497802	Superficial	SOX2
INPP5F	0.725043383	0.007423146	0.022959455	13.93767328	4.990127193	Superficial	SOX2
RNF138	0.724834032	0.017811616	0.043066688	16.12579921	5.149135029	Superficial	SOX2
ETV3	0.724257442	0.006166656	0.027303057	36.51993618	18.36824247	Suprabasal	SOX2
PBDC1	0.724096304	0.007423146	0.022959455	47.58340403	19.4017906	Superficial	SOX2
TBC1D4	0.722873499	0.017811616	0.043066688	9.618053519	3.152159836	Superficial	SOX2
WWC2	0.722704636	0.000635975	0.012925379	14.52289953	5.932481265	Basal	SOX2
CD82	0.721546364	0.012681065	0.033478436	57.52904438	27.31531055	Superficial	SOX2
PFKP	0.720566334	0.002836074	0.022562695	42.15295301	21.9314053	Basal	SOX2
GTF3C5	0.72007063	0.001526458	0.009387421	28.57071781	12.03709938	Superficial	SOX2
SUGT1	0.719687512	0.001232925	0.008679903	60.55400256	28.61191437	Superficial	SOX2
EIF4G1	0.718208604	0.000635975	0.012925379	63.74988687	36.77598904	Basal	SOX2
RHBDD2	0.717960761	0.000992312	0.01142308	43.30584464	25.55995337	Suprabasal	SOX2
RAB3IP	0.717948673	0.001232925	0.012246384	18.14985456	8.781444715	Suprabasal	SOX2
GOPC	0.717192669	0.000506425	0.007178523	40.99698046	17.8396565	Superficial	SOX2
CA13	0.716964043	0.012681065	0.033478436	26.84068309	9.406202184	Superficial	SOX2
FOXP4	0.716400668	0.000635975	0.012925379	10.1266356	4.367339639	Basal	SOX2
DIAPH1	0.715897124	0.000992312	0.014320968	49.98329195	26.85333151	Basal	SOX2
MPHOSPH10	0.715736333	0.006166656	0.020290909	41.49656213	16.38485875	Superficial	SOX2
PPIC	0.715013805	0.015054848	0.038009884	68.41924217	34.96201968	Superficial	SOX2
KHDRBS1	0.714588018	0.000401828	0.007176147	62.73550413	29.47083304	Superficial	SOX2
UTP3	0.714515084	0.006166656	0.020290909	25.70999785	9.52559203	Superficial	SOX2
SPEN	0.713127731	0.002836074	0.012662188	48.98677362	21.73061439	Superficial	SOX2
KREMEN1	0.713062714	0.000401828	0.012455402	51.12077132	27.52790114	Basal	SOX2
TMEM147	0.712669208	0.007423146	0.022959455	72.89427386	49.57190643	Superficial	SOX2
TOM1L1	0.712554624	0.004211401	0.016013117	23.61587939	8.747457616	Superficial	SOX2
TECPR2	0.712470979	9.30E-05	0.010489143	13.29861592	6.763670291	Suprabasal	SOX2
NR6A1	0.711946773	0.002315114	0.016074975	14.65826514	7.063050314	Suprabasal	SOX2
KHDRBS3	0.710487783	0.000635975	0.012925379	11.98724433	5.379939887	Basal	SOX2 + KLF5
GTF3C2	0.70899392	0.002836074	0.012662188	27.90357716	11.75661752	Superficial	SOX2
SETD7	0.708207417	0.000635975	0.007274975	27.99014587	13.201659	Superficial	SOX2
PARD6G	0.708078019	0.004211401	0.016013117	44.41779238	18.59663141	Superficial	SOX2
LASP1	0.70727919	0.001526458	0.016792744	36.89944035	16.6859833	Basal	SOX2
MIPEP	0.707166754	0.021001058	0.04834985	10.893853	3.702076167	Superficial	SOX2 + KLF5, SOX2
LEMD3	0.706801908	0.006166656	0.020290909	18.31855904	5.567001364	Superficial	SOX2
IER3	0.705746954	0.005105016	0.029792864	91.0662064	71.33517237	Basal	SOX2
TM9SF3	0.705468786	0.000317696	0.007176147	75.11455963	44.74854753	Superficial	SOX2
TPST2	0.705368445	0.008904627	0.025990329	29.58094075	12.14561785	Superficial	SOX2
MAST4	0.70518553	0.015054848	0.038009884	60.88267442	33.03507244	Superficial	SOX2
PPP1R3D	0.705122846	0.001883197	0.010234018	23.83506292	9.135189693	Superficial	SOX2
SENP6	0.703318284	0.001526458	0.009387421	39.02975419	15.42974016	Superficial	SOX2
FAM83H	0.702860491	0.002315114	0.020052792	35.23918804	17.10164149	Basal	SOX2
HIP1R	0.702694987	0.001526458	0.016792744	11.04972321	4.742867003	Basal	SOX2
USP40	0.702333154	0.006166656	0.027303057	29.51910976	16.18770834	Suprabasal	SOX2
PPARA	0.702123681	0.000153669	0.010489143	30.47842317	16.4040569	Suprabasal	SOX2
NECTIN2	0.701897768	0.000250281	0.012455402	33.17085744	15.91725556	Basal	SOX2
XPO7	0.701674727	0.012681065	0.033478436	17.37725907	6.268661514	Superficial	SOX2
MUS81	0.701670445	0.008904627	0.025990329	10.32278069	4.818304548	Superficial	SOX2
YRDC	0.69921272	0.003462059	0.014108599	21.51501832	8.558992813	Superficial	SOX2
BRD1	0.699059598	0.002315114	0.016074975	42.85777072	23.81696503	Suprabasal	SOX2
SEMA4D	0.699049431	0.006166656	0.020290909	29.50955164	12.78839383	Superficial	SOX2
TRUB2	0.698692417	0.017811616	0.043066688	28.49174933	11.26687257	Superficial	SOX2
SEC63	0.698476952	0.012681065	0.033478436	36.80728048	14.45232384	Superficial	SOX2
ATP5F1D	0.698358337	0.007423146	0.022959455	82.5012794	63.14211755	Superficial	SOX2
AJUBA	0.697980755	0.015054848	0.048140298	24.81183555	12.03369669	Suprabasal	SOX2
RWDD4	0.696875045	0.000401828	0.007176147	51.77094435	24.11983066	Superficial	SOX2
NACC1	0.696463116	0.000506425	0.012898798	20.19965077	9.054914034	Basal	SOX2
FLNA	0.695574276	0.001883197	0.018415354	68.91808084	43.431091	Basal	SOX2
SHROOM3	0.695335913	0.007423146	0.036548425	9.574191765	3.812745015	Basal	SOX2 + KLF5, SOX2
SLTM	0.695313462	0.000506425	0.007178523	63.31126801	31.76210791	Superficial	SOX2
SCAPER	0.694072236	0.007423146	0.022959455	27.74667976	10.29388181	Superficial	SOX2
MON1B	0.693303248	0.006166656	0.020290909	27.61617344	11.3071686	Superficial	SOX2
NPC1	0.692962549	0.004211401	0.027185186	15.36013339	6.703777517	Basal	SOX2
TAX1BP3	0.692447302	0.005105016	0.018023407	78.13399476	51.10089037	Superficial	SOX2
SGMS1	0.691297817	0.000635975	0.010733065	29.86271925	15.19705323	Suprabasal	SOX2 + KLF5
SDC4	0.690969578	0.001883197	0.018415354	63.26780819	36.20162181	Basal	SOX2
RING1	0.690902998	0.002836074	0.012662188	22.65387288	9.21827708	Superficial	SOX2
SUSD4	0.688905232	0.006166656	0.020290909	48.73984961	21.34803969	Superficial	KLF5, SOX2
POLR3D	0.687357327	0.003462059	0.014108599	20.8707821	7.43613414	Superficial	SOX2
PSMB4	0.685299892	0.001526458	0.009387421	58.61299947	27.96010638	Superficial	SOX2
HSPB1	0.684800626	0.012681065	0.033478436	98.45290194	93.43259814	Superficial	SOX2
EGFR	0.684370469	0.010644787	0.029507311	41.9502083	15.63246924	Superficial	SOX2
GTF3C1	0.683517925	0.004211401	0.016013117	60.9092851	33.78517881	Superficial	SOX2
ADGRL2	0.683192397	0.008904627	0.040392504	10.7417921	4.139408272	Basal	SOX2
HDAC4	0.682742832	0.015054848	0.038009884	13.30421243	4.220842783	Superficial	SOX2
CEP55	0.682070144	0.001232925	0.015721789	33.14551567	19.35039483	Basal	SOX2
ASH2L	0.681737225	0.001883197	0.010234018	23.09281134	8.409669845	Superficial	SOX2
USP37	0.681305269	0.000992312	0.01142308	15.85613317	8.095477402	Suprabasal	SOX2
COBL	0.681239795	0.015054848	0.038009884	31.42195234	12.03903453	Superficial	SOX2

CDC42SE2	0.679917135	0.017811616	0.043066688	13.52002582	5.114852508	Superficial	SOX2
LSM14B	0.678316094	0.012681065	0.033478436	21.66978744	8.709179062	Superficial	SOX2
KLHL21	0.677983908	0.000401828	0.012455402	38.21633848	18.43710045	Basal	SOX2
CASP7	0.677572835	0.004211401	0.021879278	32.86386227	16.70000183	Suprabasal	SOX2
CCSAP	0.675473422	0.000250281	0.012455402	15.54470514	7.578541078	Basal	SOX2
BRI3BP	0.675241931	0.017811616	0.043066688	27.70199565	10.31740369	Superficial	SOX2
ELP3	0.674993585	0.007423146	0.022959455	12.44484451	4.645805987	Superficial	SOX2
XPNPEP1	0.673875063	0.012681065	0.033478436	22.6153257	8.807703763	Superficial	SOX2
TSPAN31	0.673329211	0.007423146	0.022959455	32.32370625	14.57475959	Superficial	SOX2
GATAD1	0.672889383	9.30E-05	0.007176147	33.34992132	13.42729982	Superficial	SOX2
SBNO1	0.67149385	0.001232925	0.008679903	39.14640306	15.55429681	Superficial	SOX2
TTC23	0.670929937	0.004211401	0.016013117	11.357851	5.074134562	Superficial	SOX2
TBRG1	0.670360458	9.30E-05	0.007176147	35.02731125	14.25893381	Superficial	SOX2
MYO1D	0.670223138	0.001883197	0.010234018	48.98233231	23.24023778	Superficial	SOX2
AGAP3	0.669761063	0.001232925	0.008679903	41.32602591	18.75107029	Superficial	SOX2
DAXX	0.668789997	0.003462059	0.014108599	30.21577035	12.06403782	Superficial	SOX2
YKT6	0.668035629	0.001526458	0.016792744	26.64004822	12.79324033	Basal	SOX2
CDK17	0.668015169	0.003462059	0.019729589	16.41360549	8.270330179	Suprabasal	SOX2
MAL2	0.666971971	0.000992312	0.014320968	67.6656544	46.069602	Basal	SOX2, SOX2 + KLF5
LGALS8	0.666036557	0.005105016	0.018023407	31.07031235	15.28461671	Superficial	SOX2
SLC44A2	0.665545253	0.007423146	0.022959455	61.18924802	32.86938981	Superficial	SOX2
ZBTB17	0.665396487	0.000795824	0.007615337	15.53672061	6.058259512	Superficial	SOX2
RABAC1	0.665008765	0.006166656	0.020290909	73.60564023	45.0711054	Superficial	SOX2
TNFRSF10B	0.663928537	0.012681065	0.042921737	8.142564324	4.107895104	Suprabasal	SOX2
PHF10	0.663225937	0.001232925	0.012246384	57.30647758	35.85848239	Suprabasal	SOX2
SEC62	0.662496138	0.007423146	0.022959455	75.34348824	48.12215296	Superficial	KLF5
CDC25A	0.662365282	0.003462059	0.024854712	11.09681846	5.301737689	Basal	SOX2
JOSD2	0.661599184	0.001883197	0.010234018	65.70995211	35.94558676	Superficial	SOX2
TAF6	0.661561026	0.001526458	0.009387421	16.25357545	6.858573155	Superficial	SOX2
PSMC2	0.661474033	0.008904627	0.025990329	48.13192169	20.69490945	Superficial	SOX2
INTS10	0.661149836	0.021001058	0.04834985	25.09640797	9.976829546	Superficial	SOX2
NUP50	0.660882124	0.010644787	0.038255467	53.95810736	32.51693783	Suprabasal	SOX2
B3GNT2	0.659308899	0.015054848	0.038009884	48.76608833	22.08346533	Superficial	SOX2 + KLF5, SOX2
TEAD4	0.65922919	0.000795824	0.0136425	17.22245281	8.132829946	Basal	SOX2
MIDN	0.658466962	0.010644787	0.029507311	76.35570508	51.43854435	Superficial	SOX2
IL13RA1	0.658415331	0.000506425	0.012898798	29.98096386	15.03877761	Basal	SOX2
PRR14L	0.658376186	0.000506425	0.007178523	42.93223762	17.25211076	Superficial	SOX2
AARS2	0.658110216	0.010644787	0.029507311	10.38037111	3.661738738	Superficial	SOX2
LRP6	0.656261512	0.001883197	0.014679007	10.47197382	5.225409838	Suprabasal	SOX2
SLC2A1	0.655554397	0.007423146	0.022959455	54.5759334	27.05487701	Superficial	SOX2
RBMXL1	0.655488128	0.021001058	0.04834985	21.07186616	8.056498063	Superficial	SOX2
ARHGEF3	0.655422067	0.005105016	0.018023407	22.4596444	8.620999995	Superficial	SOX2
TPP2	0.654015025	0.004211401	0.016013117	29.24394806	12.0423832	Superficial	SOX2
AKAP11	0.653966626	0.017811616	0.043066688	21.36105886	7.919796639	Superficial	SOX2
MYO6	0.653027843	0.001526458	0.016792744	43.91930761	23.10721262	Basal	SOX2
PHF6	0.652696533	0.005105016	0.018023407	25.04223587	10.11666217	Superficial	SOX2
MAFK	0.650414578	0.010644787	0.044687249	14.57536945	6.225943242	Basal	SOX2
LLGL2	0.649878814	0.000401828	0.012455402	35.39044093	19.03596924	Basal	SOX2
ZNF146	0.649786015	0.021001058	0.04834985	16.61304701	5.79814609	Superficial	SOX2
SLC9A3R1	0.649284456	0.000317696	0.012455402	70.56397011	47.30720979	Basal	SOX2
COPB1	0.649171708	0.000635975	0.007274975	59.26939583	29.49107963	Superficial	SOX2
CXXC5	0.649012341	0.000635975	0.012925379	21.53093653	10.79801587	Basal	SOX2
NFIA	0.64874555	0.002836074	0.012662188	53.7574233	26.49796931	Superficial	SOX2
SNAI2	0.648425085	0.015054848	0.048140298	12.43176037	6.318331755	Suprabasal	SOX2
PIP4K2C	0.648192568	0.000401828	0.012455402	18.10247521	8.037186698	Basal	SOX2
ILVBL	0.648089774	0.021001058	0.04834985	48.24008432	20.18812239	Superficial	SOX2 + KLF5, SOX2
PRKD2	0.647914132	0.000992312	0.014320968	23.08315078	10.53299073	Basal	SOX2
GRHL1	0.647828984	0.002315114	0.020052792	17.11618921	8.766363269	Basal	SOX2
CTR9	0.647808145	0.001883197	0.010234018	46.14068978	21.288081	Superficial	SOX2
MRPS5	0.647420793	0.000795824	0.007615337	49.35732999	23.18354163	Superficial	SOX2
GCH1	0.647126938	0.002836074	0.022562695	14.58129743	6.129531195	Basal	SOX2
DNAJC2	0.646987291	0.021001058	0.04834985	25.67749226	9.788446426	Superficial	SOX2
CRTC2	0.646003988	0.004211401	0.027185186	14.0923151	5.928055588	Basal	SOX2
BCL7C	0.64482131	0.007423146	0.022959455	43.10442673	18.3022768	Superficial	SOX2
KLF16	0.644492518	0.010644787	0.044687249	26.12696008	12.3925702	Basal	SOX2
RWDD1	0.644476403	0.000153669	0.007176147	73.7233654	44.41614732	Superficial	SOX2
REXO1	0.644393123	0.000506425	0.010489143	21.29491628	11.63250148	Suprabasal	SOX2
ALKBH6	0.643264176	0.015054848	0.038009884	8.987903727	4.148990879	Superficial	SOX2
TP53BP2	0.642931517	0.004211401	0.027185186	27.76129219	12.84269071	Basal	SOX2
ETV6	0.642798297	0.001883197	0.014679007	37.04574122	20.39227825	Suprabasal	SOX2
RPAIN	0.640544324	0.002836074	0.012662188	38.56017222	15.87384888	Superficial	SOX2
NPLOC4	0.638617174	0.000635975	0.012925379	29.33666899	14.58457346	Basal	SOX2
CLN6	0.636965297	0.010644787	0.038255467	17.08599633	8.317263013	Suprabasal	SOX2
GDAP1	0.635852454	0.007423146	0.030449933	10.90194566	5.623306424	Suprabasal	SOX2
SEC23IP	0.634659651	0.007423146	0.022959455	22.1392895	8.593233906	Superficial	SOX2
CDR2	0.634532008	0.001883197	0.018415354	10.22859731	4.830933407	Basal	SOX2
B3GNT5	0.634212695	0.010644787	0.029507311	51.9158176	27.30802555	Superficial	SOX2
SPPL2A	0.633852245	0.004211401	0.016013117	43.33985558	18.32567339	Superficial	SOX2
GSK3B	0.633816567	0.000506425	0.007178523	40.39832657	17.8837765	Superficial	SOX2
PUS7L	0.633488025	0.002836074	0.012662188	29.01936478	12.46560073	Superficial	SOX2

LINS1	0.631907647	0.004211401	0.016013117	16.64845294	5.985100765	Superficial	SOX2
APPL2	0.631652725	0.008904627	0.034252903	42.9805544	24.81626319	Suprabasal	SOX2
TACSTD2	0.631602355	0.000795824	0.0136425	98.55844464	89.75262691	Basal	SOX2, KLF5
RALB	0.631027731	0.001526458	0.009387421	66.92308937	38.04879059	Superficial	SOX2
NUS1	0.630748992	0.001526458	0.009387421	26.18152527	10.93146073	Superficial	SOX2
PLEKHG6	0.630732547	0.010644787	0.044687249	10.86442646	4.748218178	Basal	SOX2
CTDP1	0.630691088	0.001883197	0.018415354	12.37768396	5.680913332	Basal	SOX2
HCFC1	0.62946212	0.010644787	0.038255467	25.31814262	13.62847611	Suprabasal	SOX2
TPBG	0.628615415	0.008904627	0.025990329	12.37015338	4.658716986	Superficial	SOX2
PSMD12	0.628161006	0.001883197	0.010234018	60.76021059	32.83275084	Superficial	SOX2
VPS53	0.627854593	0.005105016	0.018023407	31.16917285	11.9110685	Superficial	SOX2
PHRF1	0.627348754	0.000635975	0.010733065	28.08723807	15.10110419	Suprabasal	SOX2
WDFY2	0.627045805	0.001232925	0.008679903	37.26846613	15.89920156	Superficial	SOX2
DDX54	0.626076966	0.015054848	0.038009884	27.37361913	9.95378725	Superficial	SOX2
GNA15	0.625857909	0.001526458	0.016792744	34.12656535	17.38627815	Basal	SOX2
PACS1	0.624993882	0.000635975	0.012925379	32.06671086	15.46331705	Basal	SOX2 + KLF5
KIFAP3	0.624614681	0.010644787	0.029507311	24.60834527	9.675495534	Superficial	SOX2
TMBIM1	0.624550933	0.001883197	0.018415354	47.7802457	26.01769816	Basal	SOX2
RPAP3	0.62447027	0.021001058	0.04834985	22.6548561	8.653170341	Superficial	SOX2
PKIA	0.624035874	0.003462059	0.019729589	17.9108499	9.009216803	Suprabasal	SOX2
TTLL4	0.622221452	0.010644787	0.038255467	6.490223942	3.11242573	Suprabasal	SOX2
TMEM51	0.621004095	0.000506425	0.012898798	13.93565344	6.630753718	Basal	SOX2
CAPN15	0.620456443	0.008904627	0.040392504	15.5871956	6.756952252	Basal	SOX2
TTC39C	0.619828384	0.017811616	0.043066688	15.32945915	7.118245549	Superficial	SOX2 + KLF5
LMNB1	0.619073998	0.001526458	0.016792744	46.9676545	27.82506765	Basal	SOX2
NDST1	0.618998721	0.001232925	0.015721789	16.85826686	7.854229583	Basal	SOX2
HS6ST1	0.618991894	0.002315114	0.016074975	26.59241943	14.78961537	Suprabasal	SOX2 + KLF5, SOX2
NSD2	0.618825078	0.002836074	0.022562695	34.05755097	18.57604866	Basal	SOX2
NDUFA4	0.617621009	0.007423146	0.022959455	87.20515658	75.61760295	Superficial	SOX2 + KLF5
TTL	0.616625565	0.015054848	0.038009884	9.39597726	3.866933503	Superficial	SOX2
SCAF1	0.616556744	0.000401828	0.012455402	26.69961579	12.46298288	Basal	SOX2
LMNA	0.616183225	0.002315114	0.011381143	80.38720529	57.80369745	Superficial	SOX2, SOX2 + KLF5
PARL	0.615975765	0.006166656	0.020290909	34.42614282	14.25106949	Superficial	SOX2
CBX4	0.614355706	0.006166656	0.032981404	24.91951176	12.4972595	Basal	SOX2
BRWD3	0.613658138	0.008904627	0.025990329	34.96651289	13.44089262	Superficial	SOX2
RIN2	0.613619312	0.003462059	0.019729589	48.89795167	27.70980442	Suprabasal	SOX2 + KLF5
AXIN1	0.613476955	9.30E-05	0.010489143	14.62137054	7.716630072	Suprabasal	SOX2
JUN	0.612848095	0.010644787	0.029507311	77.53502385	50.74961947	Superficial	SOX2, SOX2 + KLF5
MTOR	0.612490458	0.000119764	0.012455402	22.77006362	11.23841573	Basal	SOX2
PTK7	0.612100471	0.007423146	0.030449933	17.046313	8.949674957	Suprabasal	SOX2
HNRNPU	0.611217861	0.005105016	0.018023407	71.0978732	39.92723052	Superficial	SOX2
ZBTB7A	0.611118396	0.000635975	0.012925379	72.61060878	45.60543903	Basal	SOX2
LMNB2	0.609316573	0.001526458	0.016792744	41.27761467	23.83188838	Basal	SOX2
STAM	0.608060929	0.015054848	0.038009884	33.32040064	13.39469361	Superficial	SOX2
PSPC1	0.607965342	0.006166656	0.020290909	26.2929197	9.608926425	Superficial	SOX2
UBA2	0.607402761	0.004211401	0.016013117	38.70500567	16.10997115	Superficial	SOX2
REEP5	0.606826435	0.001232925	0.008679903	66.34652048	37.75642278	Superficial	SOX2
ST3GAL1	0.606535423	0.017811616	0.043066688	24.29650378	9.987123775	Superficial	SOX2
USP47	0.60533587	0.002836074	0.012662188	45.99539819	18.77180015	Superficial	SOX2
DICER1	0.605163275	0.007423146	0.022959455	54.5955466	26.41130541	Superficial	SOX2
STIL	0.605032204	0.012681065	0.049425568	17.41909562	9.114647452	Basal	SOX2
MEX3C	0.604743821	0.002836074	0.0176927	19.5438782	10.34685587	Suprabasal	SOX2
EBNA1BP2	0.604051292	0.008904627	0.025990329	40.02189028	17.49491332	Superficial	SOX2
PKMYT1	0.603866657	0.005105016	0.029792864	26.85050168	15.06546933	Basal	SOX2
DNTTIP2	0.60369074	0.007423146	0.022959455	38.13670698	15.82482952	Superficial	SOX2
RABEP1	0.603633062	0.002315114	0.011381143	24.08573739	9.127485293	Superficial	SOX2
PSMB2	0.603597338	0.007423146	0.022959455	59.36204562	29.71514402	Superficial	SOX2
ATP11A	0.602712245	0.007423146	0.036548425	16.71750869	7.367259194	Basal	SOX2
HSD17B1	0.602155405	0.004211401	0.016013117	24.56695092	11.3566743	Superficial	SOX2
DDX1	0.601574847	0.008904627	0.025990329	44.5230924	18.97647767	Superficial	SOX2
CCDC43	0.601502898	0.012681065	0.033478436	28.05317601	11.88733009	Superficial	SOX2
F11R	0.601020271	0.001526458	0.016792744	63.0512991	36.17533407	Basal	SOX2
RRM2	0.600475257	0.010644787	0.044687249	39.28345524	25.80666442	Basal	SOX2
ZNF367	0.600262693	0.000317696	0.012455402	15.13660037	7.800981888	Basal	SOX2
IMP3	0.60004906	0.006166656	0.020290909	64.98953505	33.25115252	Superficial	SOX2
FUT2	0.599158969	0.010644787	0.044687249	11.62944402	5.532194569	Basal	SOX2
DAAM1	0.59915439	0.000992312	0.008042817	76.38738117	49.14984258	Superficial	SOX2
SYPL1	0.598514626	0.008904627	0.025990329	76.69146901	50.8731247	Superficial	SOX2
PDIA6	0.598308542	0.021001058	0.04834985	67.6873498	34.83698894	Superficial	SOX2
FRMD8	0.598257819	0.003462059	0.024854712	21.81927065	10.05410138	Basal	SOX2
ZNF286A	0.598000696	0.002315114	0.016074975	10.4340277	5.156451451	Suprabasal	SOX2
DDX5	0.596734835	0.002315114	0.011381143	80.40466764	53.89941309	Superficial	SOX2
BRD3	0.596508411	0.001883197	0.014679007	26.70160161	14.51964849	Suprabasal	SOX2
BACE2	0.594567261	0.005105016	0.029792864	44.63772598	24.73032218	Basal	SOX2 + KLF5
TNIP1	0.594212161	0.000635975	0.012925379	39.40134427	20.60852556	Basal	SOX2 + KLF5
PALB2	0.593794395	0.002836074	0.0176927	16.12236284	8.209849083	Suprabasal	SOX2
KLHL2	0.592070357	0.000795824	0.0136425	12.37145745	5.614726525	Basal	SOX2
KMT5A	0.59128364	0.006166656	0.020290909	65.0270615	35.7780276	Superficial	SOX2
FBXO45	0.588969119	0.017811616	0.043066688	19.16361532	8.241544775	Superficial	SOX2
S100PBP	0.588772465	0.003462059	0.019729589	15.51095779	7.592054591	Suprabasal	SOX2

DDB1	0.588606432	0.021001058	0.04834985	36.33819273	15.45625643	Superficial	SOX2
CCDC25	0.587148492	0.021001058	0.04834985	47.68870804	21.05364584	Superficial	SOX2
DGKZ	0.587067796	0.002836074	0.0176927	30.55586994	17.20076353	Suprabasal	SOX2
KLF6	0.586976239	0.001526458	0.009387421	83.35824169	60.69685533	Superficial	SOX2
CEBPD	0.585393294	0.004211401	0.021879278	85.52303744	65.28861982	Suprabasal	SOX2
ANKRD9	0.584905296	0.005105016	0.029792864	35.83613769	18.98583165	Basal	SOX2
SPAST	0.584604471	0.017811616	0.043066688	24.12051452	10.02080618	Superficial	SOX2
NT5C3B	0.584186592	0.021001058	0.04834985	18.38476757	7.533026674	Superficial	SOX2
CDC123	0.583905789	0.010644787	0.029507311	42.59845803	18.30831463	Superficial	SOX2
ADAM10	0.583902874	0.012681065	0.049425568	46.92135914	25.26441665	Basal	SOX2
TBCB	0.583533491	0.005105016	0.018023407	57.99479646	30.40976353	Superficial	SOX2
TTC9	0.583380961	0.012681065	0.049425568	20.57073371	10.38099781	Basal	SOX2
HMCES	0.583210817	0.002836074	0.012662188	35.52203737	16.2197152	Superficial	SOX2
PEX11B	0.583019182	0.015054848	0.038009884	23.01143191	9.454768488	Superficial	SOX2
RABGAP1	0.582347637	0.012681065	0.033478436	52.92135628	25.18508645	Superficial	SOX2
PAIP2B	0.580739387	0.021001058	0.04834985	44.41576247	20.3766825	Superficial	SOX2 + KLF5, SOX2
CHD2	0.580357251	0.000795824	0.010838271	54.26481291	33.87147191	Suprabasal	SOX2
MYO1C	0.579266015	0.004211401	0.027185186	50.3517943	28.31410955	Basal	SOX2
ACOT7	0.579261072	0.017811616	0.043066688	21.06320546	8.444328094	Superficial	SOX2
ORC1	0.578777928	0.006166656	0.032981404	13.08983666	6.574679554	Basal	SOX2
GNL3L	0.577846754	0.012681065	0.042921737	27.7323956	15.59629515	Suprabasal	SOX2
MICALL2	0.577590978	0.000795824	0.0136425	24.54449984	12.34438818	Basal	SOX2
CDC5L	0.577367904	0.010644787	0.029507311	46.85050398	20.28831503	Superficial	SOX2
ZNF503	0.575857212	0.010644787	0.029507311	27.20047124	12.22366228	Superficial	SOX2
MYO5B	0.575610396	0.001883197	0.018415354	12.41517352	5.794956349	Basal	SOX2
SHPK	0.574491073	0.008904627	0.025990329	9.471988201	3.753573693	Superficial	SOX2
BZW2	0.57447235	0.017811616	0.043066688	40.84455152	16.52980784	Superficial	SOX2
SPPL3	0.573634654	0.003462059	0.014108599	47.28374064	22.03450031	Superficial	SOX2 + KLF5, SOX2
CUL3	0.573016092	0.000401828	0.012455402	59.14956542	35.1104698	Basal	SOX2
RTN4R	0.573006335	0.010644787	0.044687249	10.07206455	4.963485412	Basal	SOX2
KATNA1	0.572676717	0.000250281	0.007176147	25.18839815	11.23271671	Superficial	SOX2
CHFR	0.572376262	0.005105016	0.029792864	15.92621312	7.523139889	Basal	SOX2
ABCB10	0.571942293	0.004211401	0.027185186	15.32551643	7.592237018	Basal	SOX2
TSTD2	0.571866552	0.017811616	0.043066688	10.47451837	4.636528888	Superficial	SOX2
STK11	0.570912857	0.004211401	0.027185186	39.54364401	21.58271748	Basal	SOX2
CNOT6	0.570895163	0.010644787	0.029507311	27.29005571	10.21974886	Superficial	SOX2
SMARCC1	0.570863674	0.010644787	0.029507311	41.37989477	19.06307239	Superficial	SOX2
STRBP	0.570780625	0.002315114	0.011381143	59.67198685	30.93992246	Superficial	SOX2
ATXN7L3	0.569461391	0.000635975	0.012925379	22.89580349	10.95539312	Basal	SOX2
MAP3K1	0.569441306	0.015054848	0.038009884	45.13891115	19.58848491	Superficial	SOX2
KLF10	0.568633025	0.008904627	0.034252903	36.85873202	21.70693085	Suprabasal	SOX2
MYLIP	0.568414442	0.001232925	0.012246384	41.7823653	24.48742287	Suprabasal	SOX2
PDXK	0.567977931	0.000992312	0.014320968	57.7535651	33.79410889	Basal	SOX2
ZMYM1	0.56738358	0.000506425	0.010489143	25.27509558	13.49142163	Suprabasal	SOX2
UBLCP1	0.566918222	0.004211401	0.016013117	39.65019314	17.19327528	Superficial	SOX2
MYBBP1A	0.56688332	0.003462059	0.024854712	22.06185652	10.56027538	Basal	SOX2
FBRSL1	0.566139832	0.003462059	0.024854712	23.29403516	10.99696911	Basal	SOX2
OPA1	0.565910951	0.015054848	0.038009884	40.1444524	16.26258589	Superficial	SOX2
MCM4	0.565800435	0.003462059	0.024854712	57.40336068	35.47954179	Basal	SOX2
PLAGL2	0.565398167	0.008904627	0.040392504	19.64283433	9.125336889	Basal	SOX2
TNFAIP8L1	0.564537093	0.007423146	0.036548425	18.10017498	10.5457503	Basal	SOX2
LARS2	0.563555094	0.006166656	0.032981404	12.02626978	5.845680963	Basal	SOX2
ALYREF	0.563301502	0.021001058	0.04834985	41.48176973	18.83980691	Superficial	SOX2
LRP10	0.563132611	0.000153669	0.012455402	60.99423501	37.67474411	Basal	SOX2
SLC12A2	0.562474231	0.003462059	0.019729589	10.77812199	5.408597712	Suprabasal	SOX2
DDR1	0.562373943	0.000795824	0.010838271	68.0993919	45.28934753	Suprabasal	SOX2
CNOT3	0.561689481	0.000992312	0.01142308	28.71801011	17.27564704	Suprabasal	SOX2
NUP188	0.561464204	0.002836074	0.022562695	30.2577921	15.1332891	Basal	SOX2
RGP1	0.560435385	0.003462059	0.019729589	13.42346619	7.532436965	Suprabasal	SOX2
ZNF768	0.559358288	0.001883197	0.014679007	12.46714038	6.490597492	Suprabasal	SOX2
INTS1	0.55918513	0.006166656	0.032981404	30.31020082	16.19867459	Basal	SOX2
BAP1	0.558420415	0.015054848	0.048140298	30.97902085	18.53894319	Suprabasal	SOX2
USP25	0.557295031	0.000196466	0.012455402	29.0726618	14.66598057	Basal	SOX2
GUF1	0.5567566	0.007423146	0.022959455	23.83669527	8.827857569	Superficial	SOX2
FAF2	0.556593152	0.001526458	0.009387421	36.29272897	14.54763219	Superficial	SOX2
EGFL6	0.556147345	0.006166656	0.027303057	33.72800327	18.89160666	Suprabasal	SOX2
ATP13A2	0.555814137	0.000401828	0.012455402	17.53077521	9.000378518	Basal	SOX2
RAB27B	0.555355604	0.002315114	0.011381143	59.79240219	31.73912783	Superficial	SOX2, SOX2 + KLF5
XPO5	0.554728734	0.015054848	0.038009884	12.41895402	4.453709417	Superficial	SOX2
TARBP1	0.553824775	0.003462059	0.019729589	19.92559264	11.6501114	Suprabasal	SOX2
RRP12	0.553439503	0.001232925	0.015721789	12.13356295	5.922857206	Basal	SOX2 + KLF5, SOX2
MCM2	0.553145931	0.008904627	0.040392504	26.87734726	14.5281405	Basal	SOX2
TES	0.551859738	0.006166656	0.020290909	40.8340672	18.31834669	Superficial	SOX2
SLC25A10	0.551805139	0.010644787	0.044687249	38.67628151	21.72036806	Basal	SOX2 + KLF5, SOX2
TMEM33	0.550352753	0.003462059	0.014108599	43.15784579	21.71450811	Superficial	SOX2
ECD	0.549038951	0.017811616	0.043066688	19.31874557	9.430919656	Superficial	SOX2
TBCD	0.548662953	0.003462059	0.024854712	27.13218841	14.47561619	Basal	SOX2
JUP	0.54864085	0.000795824	0.0136425	86.39745245	66.15826609	Basal	SOX2, SOX2 + KLF5
FAM102A	0.547362144	0.000635975	0.012925379	31.91566041	16.70012751	Basal	SOX2
TRIP12	0.545203053	0.007423146	0.036548425	49.7497347	27.85387132	Basal	SOX2

ATP6V0A2	0.544362438	0.015054848	0.048140298	13.39822462	7.244591033	Suprabasal	SOX2
SYTL1	0.544315471	0.021001058	0.04834985	73.97048156	45.83813186	Superficial	SOX2
HNRNPM	0.543720788	0.012681065	0.033478436	63.11040245	32.54516521	Superficial	SOX2
ELF3	0.54136574	0.012681065	0.049425568	56.54352937	38.3781648	Basal	SOX2, KLF5, SOX2 + KLF5
ERBB3	0.541330501	0.000795824	0.0136425	42.43033253	24.05521405	Basal	SOX2
KLC2	0.54054988	0.004211401	0.021879278	11.36024429	6.507917261	Suprabasal	SOX2
CBFB	0.539190443	0.001526458	0.016792744	30.44720682	14.98475264	Basal	SOX2
MICALL1	0.53903016	0.001526458	0.016792744	26.62948582	13.60714347	Basal	SOX2, KLF5
ATP13A3	0.538620588	0.006166656	0.032981404	33.65402979	16.85817377	Basal	SOX2
BMP2K	0.5381026	0.010644787	0.029507311	30.86873589	12.87664539	Superficial	SOX2
IRAK1	0.538095381	0.002315114	0.020052792	38.96317903	21.4747565	Basal	SOX2
TM7SF3	0.537123275	0.003462059	0.014108599	39.04521885	18.4880171	Superficial	SOX2
PSMB3	0.535779065	0.002315114	0.011381143	73.69962452	44.93950696	Superficial	SOX2
CRYBG2	0.535424694	0.003462059	0.024854712	13.47297953	6.898201952	Basal	KLF5
SOD2	0.535167327	0.008904627	0.040392504	74.53929203	49.21507383	Basal	SOX2
SNN	0.534874669	0.000401828	0.012455402	12.75407637	6.481786994	Basal	SOX2
CWC27	0.534707173	0.004211401	0.016013117	26.12936858	11.5878165	Superficial	SOX2
ARSJ	0.533709462	0.012681065	0.042921737	7.698190606	3.763901651	Suprabasal	SOX2
NUDT3	0.533337388	0.012681065	0.033478436	25.04808303	9.578946621	Superficial	SOX2
ANKRD52	0.53277344	0.004211401	0.021879278	10.11138008	5.40810124	Suprabasal	SOX2
S100A11	0.532661764	0.004211401	0.027185186	99.86119542	98.82193269	Basal	SOX2
CNOT11	0.5325964	0.002315114	0.011381143	38.1402724	17.44697014	Superficial	SOX2
CUL2	0.532346494	0.012681065	0.033478436	31.02835804	13.27069577	Superficial	SOX2
EIF2S1	0.532152229	0.006166656	0.020290909	47.45304071	21.71808627	Superficial	SOX2
ERCC6	0.531874264	0.002836074	0.0176927	24.24188941	14.03843851	Suprabasal	SOX2
NKAP	0.531109713	0.002315114	0.011381143	36.56583353	15.56036051	Superficial	SOX2
ITPKB	0.530289527	0.004211401	0.021879278	8.021108444	4.822059891	Suprabasal	SOX2
TGOLN2	0.529440537	0.006166656	0.020290909	45.81425303	23.33559263	Superficial	SOX2
TRIP10	0.527179997	0.000795824	0.0136425	26.74185502	13.52408073	Basal	SOX2 + KLF5
WAPL	0.527163417	0.017811616	0.043066688	45.72441968	20.94921423	Superficial	SOX2
ANLN	0.526520833	0.008904627	0.040392504	36.530004	22.85027331	Basal	SOX2
ANXA1	0.526354665	0.010644787	0.044687249	99.04728296	97.15687469	Basal	SOX2 + KLF5, SOX2
CTNNA1	0.525896828	0.002315114	0.020052792	77.18314236	53.75795767	Basal	SOX2
NXN	0.52540313	0.012681065	0.042921737	43.88886404	24.88549412	Suprabasal	SOX2
KLHL8	0.523147525	0.010644787	0.038255467	15.65540226	8.980403041	Suprabasal	SOX2 + KLF5
JAG1	0.522042648	0.002836074	0.022562695	60.931408	37.7428955	Basal	SOX2, SOX2 + KLF5
CHD7	0.521984316	0.007423146	0.022959455	33.38086425	14.66102246	Superficial	SOX2
RAD23A	0.521616714	0.010644787	0.029507311	49.6591592	21.30608421	Superficial	SOX2
ACBD3	0.521467682	0.001526458	0.013290882	80.56801461	59.80486031	Suprabasal	SOX2
NUMA1	0.521181726	0.005105016	0.029792864	53.1244713	30.20781378	Basal	SOX2
ATRIP	0.520900406	0.002315114	0.020052792	14.17842467	7.484290553	Basal	SOX2
NFATC2IP	0.52049701	0.012681065	0.033478436	26.51865935	10.89065796	Superficial	SOX2
GRSF1	0.519672061	0.000506425	0.007178523	47.21126079	22.01541314	Superficial	SOX2
ARID2	0.518042147	0.015054848	0.038009884	26.3492912	11.03379219	Superficial	SOX2
CUEDC1	0.517765462	0.002315114	0.020052792	16.95961156	8.258408028	Basal	SOX2
KLF3	0.517679593	0.007423146	0.030449933	83.92570547	65.51864617	Suprabasal	SOX2 + KLF5
MTRF1L	0.51714331	0.010644787	0.029507311	27.58706894	13.04922908	Superficial	SOX2
ELP4	0.516927801	0.010644787	0.029507311	14.78220504	6.588135394	Superficial	SOX2
CDC42EP5	0.515557091	0.008904627	0.040392504	51.64386602	28.76256482	Basal	SOX2
RFWD3	0.515309713	0.004211401	0.021879278	11.70454255	6.736320405	Suprabasal	SOX2
AQR	0.514022203	0.015054848	0.038009884	31.19422048	12.82419177	Superficial	SOX2
SDE2	0.512741406	0.006166656	0.027303057	23.7675373	12.72793341	Suprabasal	SOX2
PNKP	0.511632792	0.008904627	0.025990329	23.42211961	10.10092883	Superficial	SOX2
UPF1	0.511286401	0.003462059	0.024854712	37.07981162	19.42343512	Basal	SOX2
PTK6	0.510135249	0.012681065	0.049425568	19.27144799	9.872191405	Basal	SOX2
USP6NL	0.509593969	0.008904627	0.040392504	17.68897498	8.50089317	Basal	SOX2
MPZL1	0.508728407	0.008904627	0.025990329	43.3369847	22.76864965	Superficial	SOX2 + KLF5, SOX2
BUB3	0.508714222	0.007423146	0.022959455	45.69659583	21.03149772	Superficial	SOX2
PURA	0.507065933	0.000401828	0.007176147	44.46355188	21.71574662	Superficial	SOX2
FRS2	0.506244634	0.021001058	0.04834985	18.7799487	7.366259053	Superficial	SOX2
FRMD6	0.505534122	0.002315114	0.011381143	72.31695849	41.61355295	Superficial	SOX2
CCND1	0.505169811	0.021001058	0.04834985	55.07589057	29.22761701	Superficial	SOX2
GNB2	0.50420714	0.005105016	0.029792864	78.3157134	55.61594237	Basal	SOX2
SH2B1	0.503568083	0.015054848	0.048140298	17.07369447	9.923961829	Suprabasal	SOX2
ASPH	0.501403718	0.004211401	0.021879278	70.82171402	48.84771551	Suprabasal	SOX2
PPP6R1	0.5009654	0.001232925	0.015721789	29.47347937	15.12279427	Basal	SOX2
NAPG	0.499670728	0.003462059	0.014108599	57.91755322	29.49803192	Superficial	SOX2
MNAT1	0.499035232	0.001526458	0.009387421	33.82642552	15.08818925	Superficial	SOX2
HNRNPA2B1	0.497371459	0.015054848	0.038009884	74.51684124	46.06121545	Superficial	SOX2
CEP44	0.497034882	0.021001058	0.04834985	13.30912046	5.031562544	Superficial	SOX2
FAM111A	0.496459518	0.005105016	0.024262694	17.72632172	10.20689672	Suprabasal	SOX2
GUCD1	0.49643982	0.001883197	0.018415354	31.74971695	16.31569375	Basal	SOX2
CLUAP1	0.496336728	0.008904627	0.034252903	21.28641366	11.54826649	Suprabasal	SOX2
KCTD3	0.494652574	0.000992312	0.014320968	39.57678112	21.59878832	Basal	SOX2
SAP30	0.493979071	0.007423146	0.036548425	45.00566094	26.41014799	Basal	SOX2
HNRNPR	0.491063799	0.017811616	0.043066688	47.55427304	22.21570082	Superficial	SOX2
NRDC	0.490991214	0.021001058	0.04834985	55.72038228	30.60547076	Superficial	SOX2
TRIM33	0.489450207	0.017811616	0.043066688	39.7606089	17.51831737	Superficial	SOX2 + KLF5, SOX2
SEC61A1	0.488628097	0.015054848	0.038009884	45.15063283	22.414793	Superficial	SOX2
WDHD1	0.488116912	0.001526458	0.016792744	24.01800884	13.26388537	Basal	SOX2

SPAG9	0.48675745	0.000795824	0.0136425	51.34410616	30.18405598	Basal	SOX2
SEC14L2	0.486212927	0.001883197	0.018415354	12.00086909	5.227538256	Basal	SOX2
MRPS31	0.486202719	0.017811616	0.043066688	38.70154943	17.3136704	Superficial	SOX2
BDKRB2	0.485062961	0.006166656	0.032981404	17.36183784	8.801648887	Basal	SOX2
SRPK2	0.482498645	0.003462059	0.014108599	44.41141015	20.7636501	Superficial	SOX2
PSMA1	0.482165624	0.008904627	0.025990329	65.97719582	36.27990299	Superficial	SOX2
TPGS1	0.481782703	0.017811616	0.043066688	41.59724017	19.97718233	Superficial	SOX2
CACTIN	0.481764839	0.010644787	0.038255467	9.797951605	5.480509165	Suprabasal	SOX2
CTTN	0.481760114	0.002315114	0.020052792	68.34703483	44.04458385	Basal	SOX2
ZDHHC5	0.481638674	0.002836074	0.022562695	45.41187793	26.26191115	Basal	SOX2
SH3GL1	0.481509796	0.000317696	0.012455402	40.9722635	22.65329046	Basal	SOX2
FMR1	0.481142069	0.005105016	0.024262694	43.2968248	26.86475631	Suprabasal	SOX2
NADK	0.480684707	0.010644787	0.044687249	20.85555924	10.89633778	Basal	SOX2
RALY	0.479004374	0.017811616	0.043066688	71.36994745	45.26189074	Superficial	SOX2
SPECC1	0.478790004	0.010644787	0.044687249	16.64152372	8.452011622	Basal	SOX2
DNAJB14	0.47843695	0.007423146	0.022959455	35.23391723	15.120162	Superficial	SOX2
TRIM16	0.475968725	0.006166656	0.032981404	45.63964355	28.50255283	Basal	SOX2
DVL3	0.475846201	0.000250281	0.010489143	34.21125811	21.10756577	Suprabasal	SOX2
ARMC1	0.474807714	0.008904627	0.025990329	27.30242392	11.60866805	Superficial	SOX2
R3HDM4	0.474476837	0.000992312	0.014320968	34.1889281	18.11556037	Basal	SOX2
TWF1	0.473939191	0.001883197	0.018415354	57.49152249	35.50918611	Basal	SOX2
DENND2D	0.47280977	0.000317696	0.012455402	36.61631128	20.5686023	Basal	SOX2
AHNAK	0.472580773	0.000992312	0.014320968	94.70051847	83.04168059	Basal	SOX2, SOX2 + KLF5
SIK3	0.471835245	0.003462059	0.024854712	16.02132565	7.698144507	Basal	SOX2
EHD1	0.471129417	0.006166656	0.032981404	18.87141849	9.712745193	Basal	SOX2
SCO1	0.469811368	0.002315114	0.011381143	27.81739673	11.84774497	Superficial	SOX2
RCN1	0.469437498	0.007423146	0.030449933	39.25339792	22.71572912	Suprabasal	SOX2
THUMPD3	0.469358263	0.021001058	0.04834985	30.94173887	12.9790936	Superficial	SOX2
RYK	0.469142284	0.002315114	0.011381143	30.19491679	13.27920261	Superficial	SOX2
PHF20L1	0.468437348	0.007423146	0.022959455	61.30332804	34.85572051	Superficial	SOX2
SHCBP1	0.467837807	0.005105016	0.029792864	22.36528606	12.86186587	Basal	SOX2
DENND1A	0.467812365	0.012681065	0.042921737	6.96267468	4.220814957	Suprabasal	SOX2 + KLF5
PPM1D	0.467316307	0.002836074	0.022562695	13.17120466	6.699147999	Basal	SOX2
ZNF598	0.466855393	0.002315114	0.020052792	20.47352294	10.30751396	Basal	SOX2
PGAP2	0.466709668	0.012681065	0.033478436	35.43820831	16.82219589	Superficial	SOX2
PELP1	0.466601935	0.005105016	0.024262694	27.07148126	15.08417818	Suprabasal	SOX2
MAP4K4	0.464946588	0.002315114	0.020052792	29.0491676	15.70885276	Basal	SOX2
USP22	0.463671294	0.012681065	0.042921737	44.5022348	27.78089775	Suprabasal	SOX2
MGAT4B	0.463578307	0.012681065	0.033478436	63.79064886	35.34931688	Superficial	SOX2
DUSP16	0.463416326	0.010644787	0.044687249	12.4917934	5.563776748	Basal	SOX2
FAAP100	0.462850974	0.003462059	0.024854712	14.96658424	7.878325253	Basal	SOX2
ERICH1	0.462223974	0.015054848	0.038009884	25.89510034	10.06784504	Superficial	SOX2
SLC22A23	0.461757582	0.006166656	0.027303057	39.49224725	24.87529787	Suprabasal	SOX2
STAM2	0.459897509	0.002836074	0.0176927	43.15189679	27.25445502	Suprabasal	SOX2
STRN4	0.459425666	0.000506425	0.012898798	25.0584803	12.69442914	Basal	SOX2
PRKCI	0.459071509	0.012681065	0.042921737	51.41691073	33.04730749	Suprabasal	SOX2
GATAD2A	0.458863107	0.003462059	0.024854712	35.79161866	18.9186545	Basal	SOX2
TMEM68	0.458247895	0.015054848	0.038009884	16.45128655	6.283049534	Superficial	SOX2
EIF5B	0.45771753	0.017811616	0.043066688	63.11019127	36.34802617	Superficial	SOX2
NUP98	0.457010854	0.000992312	0.014320968	32.6907407	17.7336952	Basal	SOX2
DYNC1LI2	0.456769397	0.000635975	0.007274975	61.99864339	35.22299681	Superficial	SOX2
CAND1	0.454883616	0.015054848	0.048140298	51.67717794	32.39796423	Suprabasal	SOX2
SEC16A	0.454482179	0.010644787	0.044687249	21.18626829	10.61205209	Basal	SOX2
ADAM17	0.453611455	0.010644787	0.029507311	30.19171051	11.82663939	Superficial	SOX2
THUMPD2	0.45263715	0.021001058	0.04834985	15.97784169	6.56066906	Superficial	SOX2 + KLF5
ACACA	0.452417245	0.008904627	0.040392504	29.6471181	15.65104834	Basal	SOX2
PPP2R5E	0.452064751	0.021001058	0.04834985	55.92045393	30.5051335	Superficial	SOX2
SLK	0.451135179	0.000401828	0.012455402	60.34261778	38.60359737	Basal	SOX2
MOCOS	0.450507041	0.007423146	0.036548425	11.66780025	6.100869399	Basal	SOX2
TCOF1	0.449688868	0.001526458	0.016792744	41.03802722	24.1069045	Basal	SOX2
PANX1	0.449051913	0.012681065	0.049425568	9.645531418	4.874082636	Basal	SOX2
ZFP90	0.448202743	0.002315114	0.016074975	15.3130742	8.801039518	Suprabasal	SOX2
FBXO46	0.447624189	0.000992312	0.014320968	16.59204452	8.127326468	Basal	SOX2
STRN	0.447432023	0.010644787	0.044687249	38.84829394	21.44867213	Basal	SOX2
NOL6	0.446672582	0.010644787	0.044687249	13.11910862	6.729699791	Basal	SOX2
MDN1	0.446383845	0.005105016	0.029792864	24.44692351	12.62374257	Basal	SOX2
CNN2	0.445861687	0.006166656	0.032981404	56.8142339	32.70563775	Basal	SOX2
ADA	0.445206613	0.005105016	0.029792864	22.03751847	11.78920222	Basal	SOX2
FBRS	0.444705702	0.012681065	0.049425568	30.07899109	15.75416998	Basal	SOX2
STON2	0.443577293	0.008904627	0.034252903	31.50370257	18.35266099	Suprabasal	SOX2
ACOX1	0.442951284	0.004211401	0.027185186	30.32328526	15.83888351	Basal	SOX2 + KLF5
NEDD1	0.442012152	0.008904627	0.034252903	14.81115727	7.965198757	Suprabasal	SOX2
ARAF	0.44188733	0.001883197	0.018415354	23.96647628	12.83259466	Basal	SOX2
GTF3C4	0.438299723	0.010644787	0.038255467	20.14127445	12.16362485	Suprabasal	SOX2
ARMT1	0.437711806	0.010644787	0.029507311	27.61166468	11.64950814	Superficial	SOX2
ZNF513	0.437149719	0.002836074	0.0176927	13.08879921	8.269687283	Suprabasal	SOX2
GPC1	0.4335995	0.005105016	0.024262694	58.84720048	40.39056084	Suprabasal	SOX2
PLS1	0.433501833	0.007423146	0.036548425	23.59769785	12.01671039	Basal	SOX2 + KLF5
TOPBP1	0.432977178	0.003462059	0.019729589	26.22138818	15.40008064	Suprabasal	SOX2
CTSB	0.43282805	0.002315114	0.020052792	79.26851537	55.67357313	Basal	SOX2

CRABP2	0.432650393	0.008904627	0.040392504	83.33089204	72.13197537	Basal	SOX2
RAD54L2	0.431128164	0.006166656	0.027303057	13.54171258	7.65361492	Suprabasal	SOX2
PCBP4	0.430029248	0.012681065	0.042921737	12.17578092	7.218205687	Suprabasal	SOX2
SMCHD1	0.429808244	0.006166656	0.027303057	37.02295778	22.63417127	Suprabasal	SOX2
SNX1	0.429491131	0.002315114	0.016074975	63.4365984	43.72910637	Suprabasal	SOX2
DHX32	0.427564306	0.005105016	0.029792864	29.98898918	16.15208417	Basal	SOX2
PTPN9	0.426795876	0.015054848	0.048140298	12.96039252	7.098054443	Suprabasal	SOX2
SRPRA	0.426396935	0.002315114	0.020052792	47.1307841	26.94631004	Basal	SOX2
MAPRE3	0.426209732	0.012681065	0.042921737	6.598992634	3.77396755	Suprabasal	SOX2
STK24	0.423981945	0.004211401	0.027185186	66.7561237	42.88101471	Basal	SOX2
PPIL4	0.423935678	0.001526458	0.009387421	33.29502682	14.07107106	Superficial	SOX2
HDAC2	0.423736835	0.012681065	0.033478436	46.77046386	22.13070123	Superficial	SOX2
DONSON	0.422694654	0.002315114	0.020052792	18.01339736	10.3784105	Basal	SOX2
ATAD2	0.422335996	0.007423146	0.036548425	52.37566144	34.75413718	Basal	SOX2
EIF4G3	0.421871801	0.012681065	0.033478436	41.23540469	19.80500389	Superficial	SOX2 + KLF5
KIF1B	0.421858334	0.001232925	0.015721789	28.04042735	15.11331966	Basal	SOX2
CSNK1G2	0.421806566	0.001526458	0.016792744	41.06864299	23.48149592	Basal	SOX2
AP1S1	0.421228877	0.015054848	0.038009884	48.96038672	26.48981535	Superficial	SOX2
TAF13	0.420823645	0.007423146	0.036548425	21.98425883	10.86907487	Basal	SOX2
RPRD1B	0.420555262	0.004211401	0.027185186	24.94256673	13.10438258	Basal	SOX2
PSME3	0.419754849	0.003462059	0.024854712	36.95658387	21.39527241	Basal	SOX2
EIF4G2	0.419134212	0.005105016	0.029792864	89.21513224	71.25553618	Basal	SOX2
ABHD17C	0.418557758	0.007423146	0.036548425	29.43653927	15.59754586	Basal	SOX2
BAG3	0.418424075	0.001526458	0.016792744	57.19094076	35.78013283	Basal	SOX2
DCTN1	0.418072859	0.002315114	0.016074975	51.97888715	35.8322889	Suprabasal	SOX2
SOS1	0.416701949	0.004211401	0.021879278	38.80402609	24.64701172	Suprabasal	SOX2
PLD1	0.416621216	0.010644787	0.044687249	26.55414004	14.95657525	Basal	SOX2 + KLF5
CSNK2A2	0.416250629	0.007423146	0.030449933	49.98222445	31.77717767	Suprabasal	SOX2
SIGMAR1	0.415664039	0.010644787	0.044687249	45.53288418	27.00160704	Basal	SOX2
BAIAP2L1	0.414981604	0.000992312	0.014320968	23.04935728	12.37712073	Basal	SOX2, SOX2 + KLF5
RRAGD	0.411940318	0.008904627	0.025990329	53.16936183	28.17200598	Superficial	SOX2
LARP4B	0.411512699	0.015054848	0.048140298	44.28576258	28.30531636	Suprabasal	SOX2
RRP1B	0.411164292	0.000795824	0.010838271	32.97235215	19.66653476	Suprabasal	SOX2
MAPK1	0.410899477	0.005105016	0.029792864	48.74884732	28.8759107	Basal	SOX2
OXSR1	0.410887757	0.012681065	0.049425568	31.81485499	17.55439172	Basal	SOX2
PPP4R3A	0.409837121	0.012681065	0.042921737	46.70037185	29.32635926	Suprabasal	SOX2
ABR	0.40963197	0.008904627	0.040392504	28.23171008	14.95736728	Basal	SOX2 + KLF5
TULP4	0.409453896	0.000992312	0.01142308	35.80588971	22.82858622	Suprabasal	SOX2 + KLF5
DARS2	0.408800324	0.007423146	0.036548425	16.0927049	8.816252156	Basal	SOX2
TULP3	0.40868924	0.004211401	0.021879278	33.2829504	21.53384324	Suprabasal	SOX2 + KLF5, SOX2
RAB6A	0.408482401	0.012681065	0.033478436	64.19858162	38.33939519	Superficial	SOX2
NF2	0.407599037	0.000401828	0.012455402	24.32252414	13.08445788	Basal	SOX2
HNRNPUL1	0.404922839	0.000795824	0.010838271	60.58384221	41.805875	Suprabasal	SOX2
UBE3A	0.404126921	0.008904627	0.025990329	52.68022712	27.47703569	Superficial	SOX2
DNAJC21	0.40361818	0.000992312	0.008042817	59.66645697	34.72447459	Superficial	SOX2 + KLF5, SOX2
CREBBP	0.402571645	0.015054848	0.048140298	39.79409462	25.00942118	Suprabasal	SOX2
SMYD4	0.402425291	0.008904627	0.034252903	7.546562152	4.396569188	Suprabasal	SOX2
FAM204A	0.399857747	0.003462059	0.014108599	44.29048694	20.2946133	Superficial	SOX2
SRBD1	0.399456503	0.007423146	0.030449933	14.79261982	9.197075729	Suprabasal	SOX2
HLA-E	0.399386809	0.002315114	0.020052792	91.46666117	75.79158387	Basal	SOX2
CUL1	0.398192813	0.007423146	0.030449933	40.65802972	25.80310235	Suprabasal	SOX2
PSMA5	0.398145724	0.021001058	0.04834985	65.47278703	39.48031861	Superficial	SOX2
KAT5	0.396068142	0.005105016	0.018023407	28.64432152	13.29728154	Superficial	SOX2
HDLBP	0.394837607	0.000401828	0.012455402	79.95998073	57.10289635	Basal	SOX2
ABCB7	0.392337334	0.003462059	0.014108599	21.58574896	8.963636351	Superficial	SOX2
LRRFIP1	0.391779281	0.000401828	0.012455402	76.39416407	54.39210723	Basal	SOX2
CAB39	0.391085547	0.008904627	0.040392504	38.52456553	21.76720296	Basal	SOX2
ZNF740	0.387806449	0.003462059	0.019729589	11.0501489	6.429114307	Suprabasal	SOX2
DNAJC5	0.387002103	0.006166656	0.032981404	30.47029786	16.50597108	Basal	SOX2
VBP1	0.386752618	0.021001058	0.04834985	48.30199504	24.9791918	Superficial	SOX2
VGLL4	0.386286071	0.006166656	0.027303057	69.20123638	49.11019076	Suprabasal	SOX2
PPARD	0.385509275	0.005105016	0.029792864	13.50772511	7.152754309	Basal	SOX2
RAVER2	0.385020215	0.015054848	0.048140298	11.98122369	7.225062747	Suprabasal	SOX2
IQCK	0.384096874	0.015054848	0.048140298	7.59743852	4.233400157	Suprabasal	SOX2
CAP1	0.383283453	0.004211401	0.027185186	70.38203023	48.7778019	Basal	SOX2
PRCC	0.382064623	0.001883197	0.010234018	33.12701812	15.6260195	Superficial	SOX2
SPINT2	0.381507478	0.017811616	0.043066688	89.89260612	77.91907541	Superficial	SOX2
ATP6V1C1	0.38106856	0.004211401	0.021879278	29.29032455	18.23094114	Suprabasal	SOX2
HNRNPD	0.379637837	0.008904627	0.025990329	66.22805206	39.57404479	Superficial	SOX2
EZH2	0.379576368	0.004211401	0.027185186	40.42867394	25.49765124	Basal	SOX2
ZFP64	0.37948406	0.010644787	0.038255467	12.40083125	7.455865285	Suprabasal	SOX2
GNL1	0.37843941	0.006166656	0.020290909	31.11718831	14.43086912	Superficial	SOX2
CDK12	0.378351456	0.001526458	0.013290882	48.53275386	31.62280627	Suprabasal	SOX2
NCKAP1	0.378170379	0.010644787	0.044687249	58.11956911	37.04242786	Basal	SOX2
ZNF672	0.376681299	0.010644787	0.044687249	19.56555888	10.59380964	Basal	SOX2
POLR2B	0.37636934	0.012681065	0.042921737	41.3802777	24.66201886	Suprabasal	SOX2
GMCL1	0.375873197	0.007423146	0.022959455	31.99316097	14.72130798	Superficial	SOX2
KCTD5	0.375183782	0.004211401	0.027185186	23.89015549	13.41836054	Basal	SOX2
GDE1	0.372793116	0.021001058	0.04834985	46.2836854	23.83445075	Superficial	SOX2
HGS	0.372464258	0.002836074	0.022562695	37.66991891	21.6422198	Basal	SOX2

ACTR1A	0.372227417	0.004211401	0.027185186	36.13204977	21.11663527	Basal	SOX2
SRRT	0.371491622	0.001883197	0.014679007	37.76365529	23.85917939	Suprabasal	SOX2
TCFL5	0.371359147	0.000506425	0.010489143	8.353413253	4.994880006	Suprabasal	SOX2
ASB6	0.367944847	0.003462059	0.024854712	12.57515056	6.43489909	Basal	SOX2
DSN1	0.367401907	0.006166656	0.027303057	14.87864595	9.062953013	Suprabasal	SOX2
KLF5	0.366989473	0.004211401	0.016013117	90.73767968	78.42717769	Superficial	KLF5, SOX2 + KLF5
UBE3B	0.365018038	0.010644787	0.044687249	22.00039008	11.50400949	Basal	SOX2
CLSPN	0.364721605	0.012681065	0.049425568	28.84436189	17.50717111	Basal	SOX2
EIF4ENIF1	0.364297387	0.000635975	0.010733065	22.31296889	13.97106774	Suprabasal	SOX2
C6orf132	0.363808744	0.001232925	0.015721789	47.99793227	30.24246367	Basal	SOX2
TMCC1	0.361299072	0.002836074	0.022562695	15.70166472	8.402344171	Basal	SOX2
SPATS2L	0.360680694	0.000992312	0.01142308	80.43917619	62.40270925	Suprabasal	KLF5
CDC26	0.359851754	0.005105016	0.018023407	53.12997972	29.84803495	Superficial	SOX2
HOOK2	0.359210707	0.021001058	0.04834985	48.76542349	25.87326508	Superficial	SOX2
RASAL2	0.357863099	0.012681065	0.049425568	28.13204675	15.36324284	Basal	SOX2
GALE	0.357551358	0.010644787	0.044687249	38.86560774	23.09276026	Basal	SOX2
MASTL	0.355928529	0.010644787	0.044687249	22.26375576	12.5982032	Basal	SOX2
CUL4A	0.354110326	0.003462059	0.024854712	38.8403561	23.05650822	Basal	SOX2
TMOD3	0.353471008	0.010644787	0.044687249	73.12027102	52.13714682	Basal	SOX2
NUDT4	0.353268938	0.008904627	0.034252903	30.43480114	18.89608606	Suprabasal	SOX2, SOX2 + KLF5
XPO1	0.352059571	0.015054848	0.048140298	60.17473282	39.24143377	Suprabasal	SOX2
ZNF330	0.352054942	0.021001058	0.04834985	33.66922418	16.39634589	Superficial	SOX2
TLK2	0.351292132	0.007423146	0.030449933	37.89732037	23.84071245	Suprabasal	SOX2
LONP1	0.351176435	0.010644787	0.044687249	27.96313576	15.97710597	Basal	SOX2
TADA2A	0.350900882	0.004211401	0.021879278	16.73317655	10.39759773	Suprabasal	SOX2 + KLF5, SOX2
UBAP1	0.348148108	0.012681065	0.049425568	28.3949319	15.61827932	Basal	SOX2
ETF1	0.347930763	0.012681065	0.049425568	64.85346179	43.8817305	Basal	SOX2
SIN3B	0.345189095	0.010644787	0.038255467	21.68181576	14.24138181	Suprabasal	SOX2
EHMT1	0.344749701	0.001232925	0.015721789	34.67135042	19.58732315	Basal	SOX2
NOM1	0.341602586	0.004211401	0.027185186	29.52543453	17.88515075	Basal	SOX2
DCAF7	0.341384602	0.021001058	0.04834985	34.97799652	16.46057478	Superficial	SOX2
HECTD1	0.341197856	0.008904627	0.040392504	55.19918194	35.45256668	Basal	SOX2
ETAA1	0.34086341	0.002836074	0.0176927	18.78995792	11.89426174	Suprabasal	SOX2
FOXK2	0.340257957	0.000635975	0.012925379	28.52080745	15.8799137	Basal	SOX2
MPRIP	0.339903944	0.012681065	0.049425568	29.41008776	16.52158519	Basal	SOX2
ACTN4	0.337577641	0.010644787	0.044687249	82.9768807	62.57568151	Basal	SOX2
UBQLN1	0.337087049	0.007423146	0.036548425	47.23144812	28.48757739	Basal	SOX2
ZNF398	0.335352651	0.012681065	0.049425568	11.78243869	6.596489406	Basal	SOX2
PGRMC2	0.335234595	0.021001058	0.04834985	52.81530241	27.35480933	Superficial	SOX2
BAG6	0.334098072	0.000635975	0.012925379	45.49164076	27.55833727	Basal	SOX2
CD2AP	0.328979616	0.007423146	0.036548425	55.36500458	35.22370908	Basal	SOX2
MARK3	0.328532891	0.001232925	0.015721789	39.36693965	23.67234087	Basal	SOX2
BTBD10	0.324843477	0.012681065	0.049425568	37.09246296	21.95821711	Basal	SOX2
RAF1	0.324610208	0.010644787	0.044687249	37.95147784	22.23152954	Basal	SOX2
SNX9	0.323080903	0.003462059	0.024854712	31.57020056	18.17767224	Basal	SOX2
RNF126	0.321893513	0.012681065	0.049425568	56.94156687	36.51439612	Basal	SOX2
NCAPD3	0.319375719	0.005105016	0.029792864	37.33730953	23.54175813	Basal	SOX2
TPD52L1	0.319203489	0.012681065	0.049425568	70.8735422	49.83127217	Basal	SOX2
TADA2B	0.318377235	0.007423146	0.036548425	11.44702194	6.08819351	Basal	SOX2
RAD21	0.317255801	0.007423146	0.036548425	72.37467016	51.73760027	Basal	SOX2
DPP9	0.316281208	0.005105016	0.029792864	24.5146002	13.78317029	Basal	SOX2
DHX15	0.315489463	0.010644787	0.038255467	50.30688361	33.87821156	Suprabasal	SOX2
PRRC2C	0.314883273	0.007423146	0.030449933	87.62866402	72.69746995	Suprabasal	SOX2
AKAP8	0.314009175	0.005105016	0.024262694	26.62854161	16.76378779	Suprabasal	SOX2
DNAAF5	0.313393642	0.004211401	0.027185186	25.39436291	14.69899341	Basal	SOX2
CSNK1A1	0.311878468	0.002836074	0.0176927	93.53449428	84.71976112	Suprabasal	SOX2
MIS18BP1	0.311634917	0.005105016	0.024262694	39.65562411	24.88374556	Suprabasal	SOX2
ANKRD11	0.311535163	0.005105016	0.024262694	77.75687683	59.74175098	Suprabasal	SOX2
SUPT5H	0.311462565	0.010644787	0.044687249	41.30614259	23.88312125	Basal	SOX2
PRKCH	0.310775043	0.010644787	0.044687249	12.78922278	7.133312113	Basal	SOX2, SOX2 + KLF5
ANXA11	0.309510653	0.007423146	0.036548425	80.1395934	59.90618267	Basal	SOX2 + KLF5
SMARCA2	0.30736207	0.012681065	0.042921737	50.47896445	33.01055583	Suprabasal	SOX2
RBM26	0.306807667	0.004211401	0.021879278	45.24969731	28.33994468	Suprabasal	SOX2
EDC3	0.305267304	0.003462059	0.024854712	13.40354087	7.180247317	Basal	SOX2
ACTB	0.304849735	0.004211401	0.027185186	99.71378693	97.52395914	Basal	SOX2
RNASEL	0.304812706	0.008904627	0.034252903	20.4978733	13.22398589	Suprabasal	SOX2
PRRG4	0.303894969	0.006166656	0.027303057	83.56882003	69.16067751	Suprabasal	SOX2, SOX2 + KLF5
CNDP2	0.301775633	0.007423146	0.036548425	41.87890185	25.30966965	Basal	SOX2
PPM1A	0.297552231	0.010644787	0.044687249	31.80766749	17.79182567	Basal	SOX2
PCM1	0.29663167	0.012681065	0.042921737	62.41064601	43.03776245	Suprabasal	SOX2
XRCC1	0.296049831	0.012681065	0.042921737	16.1532907	10.76767722	Suprabasal	SOX2
PTBP1	0.294116815	0.005105016	0.029792864	65.2690628	44.63874797	Basal	SOX2
RNF40	0.292210922	0.002836074	0.022562695	23.01616376	12.95846387	Basal	SOX2
INTS14	0.291042279	0.012681065	0.042921737	21.96252873	13.69680143	Suprabasal	SOX2
UBTF	0.289426243	0.004211401	0.021879278	55.33188696	37.74718139	Suprabasal	SOX2
BDH1	0.287800194	0.001526458	0.016792744	37.68427329	23.60581804	Basal	SOX2
ZNF174	0.285899464	0.006166656	0.027303057	6.81185006	4.65851935	Suprabasal	SOX2
TAX1BP1	0.28569555	0.006166656	0.032981404	70.90543279	49.84024475	Basal	SOX2
OPA3	0.285098991	0.015054848	0.048140298	18.78897384	12.76558836	Suprabasal	SOX2
KPNA4	0.284385351	0.008904627	0.040392504	53.03150064	33.71170895	Basal	SOX2

CSNK1D	0.282246278	0.006166656	0.032981404	38.6253149	23.71317908	Basal	SOX2
C6orf136	0.282069153	0.003462059	0.024854712	25.12174217	14.50978536	Basal	SOX2 + KLF5
KDM2A	0.281821164	0.005105016	0.029792864	44.65559244	26.94707233	Basal	SOX2
NFX1	0.281778885	0.008904627	0.034252903	30.94818377	19.69308165	Suprabasal	SOX2
KAT7	0.280513646	0.003462059	0.019729589	31.96355226	20.50682651	Suprabasal	SOX2
ZNF623	0.278474992	0.005105016	0.024262694	10.25246344	6.504611864	Suprabasal	SOX2
PSMD2	0.278455513	0.017811616	0.043066688	64.95891017	40.28973587	Superficial	SOX2
SYNRG	0.277856083	0.008904627	0.040392504	27.18815891	15.59211998	Basal	SOX2
CDKN2AIP	0.27405597	0.003462059	0.024854712	48.78322567	32.15435898	Basal	SOX2
APLP2	0.270292897	0.012681065	0.042921737	73.82533826	57.93670449	Suprabasal	SOX2
SF3A1	0.268950282	0.002315114	0.020052792	40.45425161	24.11803561	Basal	SOX2
DNAJC9	0.267541983	0.008904627	0.034252903	36.74656251	23.59342056	Suprabasal	SOX2
PAWR	0.266458622	0.010644787	0.044687249	63.09732871	43.42629148	Basal	SOX2
CTCF	0.266102688	0.010644787	0.038255467	50.85548206	33.85027116	Suprabasal	SOX2
SMC3	0.265657661	0.010644787	0.038255467	66.24412926	45.72828955	Suprabasal	SOX2
PAFAH1B2	0.265099423	0.006166656	0.032981404	49.89534049	31.20887734	Basal	SOX2
DDA1	0.264148216	0.004211401	0.027185186	39.59917761	24.07286753	Basal	SOX2
BRD4	0.260587942	0.010644787	0.044687249	59.06233998	38.69743774	Basal	SOX2
EPN1	0.259066852	0.004211401	0.027185186	52.19066592	33.714443	Basal	SOX2
NFKBIB	0.258046044	0.010644787	0.044687249	27.74931509	16.39772077	Basal	SOX2
ELF1	0.257772111	0.006166656	0.027303057	78.78518903	61.89883688	Suprabasal	SOX2
SAFB2	0.256603266	0.006166656	0.027303057	33.63209571	21.6889824	Suprabasal	SOX2
SPINT1	0.251117707	0.008904627	0.040392504	62.93750767	43.57976501	Basal	SOX2

Supplemental Table 8. Gene expression profile of EoE DEGs known to be regulated by the SOX2-KLF5 interaction.

gene	Log2FC	Pval	FDR_Pval	Top Changed Compartment
NRXN1	5.011064578	0.000184184	0.010398392	Suprabasal
NFATC2	4.981012295	0.000989444	0.008042817	Superficial
KCNJ2	4.494243322	0.000506425	0.010398392	Suprabasal
BCL2L15	4.350031654	0.000153669	0.010398392	Suprabasal
RUNX2	3.769129479	0.000504805	0.007178523	Superficial
LYPD6B	3.712117954	0.004211401	0.016013117	Superficial
SGK1	3.680085691	0.000153669	0.007176147	Superficial
ST8SIA6	3.586099976	0.000634007	0.007274975	Superficial
CMYA5	3.552817397	0.001526458	0.009387421	Superficial
HRH1	3.502634807	0.000992312	0.011395032	Suprabasal
TNS4	2.993108132	0.001232925	0.008679903	Superficial
RASGRP1	2.869246892	0.000506425	0.010398392	Suprabasal
LY75	2.675558265	5.55E-05	0.007176147	Superficial
HS3ST3A1	2.549605795	0.003462059	0.014108599	Superficial
HS3ST1	2.544170249	0.000153669	0.010398392	Suprabasal
RARB	2.530013416	0.000992312	0.008042817	Superficial
CLDN1	2.418800238	0.000506425	0.007178523	Superficial
LITAF	2.418477626	0.000401828	0.010398392	Suprabasal
NRP2	2.366104366	0.001232925	0.01240258	Suprabasal
SOX21	2.296941678	0.000506425	0.007178523	Superficial
GGH	2.23883892	0.000317696	0.007176147	Superficial
UTRN	2.217242242	0.000196466	0.007176147	Superficial
MYOF	2.181332839	0.000795824	0.007615337	Superficial
GIPC2	2.130796901	0.000119764	0.007176147	Superficial
MYH9	2.016181739	0.000401828	0.007176147	Superficial
PDLIM4	1.989624172	0.000317696	0.007176147	Superficial
NUAK2	1.98293209	0.001232925	0.008679903	Superficial
LPCAT4	1.972938051	0.000795824	0.007615337	Superficial
LRRC8B	1.948757418	0.001526458	0.009387421	Superficial
TLE1	1.923101892	0.000119764	0.007176147	Superficial
SGMS2	1.870786282	0.000401828	0.007176147	Superficial
FMN1	1.866940741	0.000992312	0.008042817	Superficial
GCNT3	1.859066598	0.002315114	0.016076911	Suprabasal
SERPINB5	1.819859014	0.000506425	0.007178523	Superficial
ARHGAP26	1.751173085	7.20E-05	0.007176147	Superficial
ADAM9	1.72837896	0.001232925	0.01240258	Suprabasal
IGF2R	1.719942816	0.000992312	0.008042817	Superficial
ATP8B1	1.711098498	9.30E-05	0.010398392	Suprabasal
FAM3C	1.6929743	0.000992312	0.008042817	Superficial
CD9	1.664880927	0.000317696	0.007176147	Superficial
DSG3	1.659685097	0.000401828	0.007176147	Superficial
SERPINB2	1.591511492	0.000635975	0.010421015	Suprabasal
MACF1	1.585172836	0.001883197	0.010234018	Superficial

TMPRSS11D	1.540082196	0.008904627	0.040392504	Basal
NRIP1	1.527774608	0.000992312	0.008042817	Superficial
SETBP1	1.526392337	0.000795824	0.007615337	Superficial
MYH14	1.506036602	0.000635975	0.007274975	Superficial
MCC	1.495164205	0.001232925	0.008679903	Superficial
SLC16A7	1.492659835	0.000317696	0.007176147	Superficial
EEA1	1.479639763	0.000196466	0.007176147	Superficial
KLF4	1.451647249	0.000317696	0.007176147	Superficial
RAB11FIP1	1.442200578	0.000317696	0.012455402	Basal
ETS2	1.440460005	0.000317696	0.010398392	Suprabasal
TJP2	1.428786705	0.000250281	0.012455402	Basal
FAM83B	1.409210634	0.005105016	0.018023407	Superficial
ANGPTL4	1.390767663	9.30E-05	0.012455402	Basal
OCIAD2	1.31691942	0.000795824	0.007615337	Superficial
ARHGAP18	1.313208605	0.001526458	0.009387421	Superficial
ERC1	1.308852525	0.000992312	0.008042817	Superficial
SOX13	1.301876207	0.004211401	0.016013117	Superficial
TMPRSS4	1.291648313	0.000317696	0.012455402	Basal
PDE4D	1.288243	0.003462059	0.014108599	Superficial
SHROOM2	1.258271405	0.000635975	0.007274975	Superficial
MREG	1.217754559	0.000401828	0.012455402	Basal
MBOAT2	1.21344202	0.000795824	0.0136425	Basal
ROCK2	1.20861768	0.001232925	0.008679903	Superficial
LIMA1	1.205072658	0.001232925	0.008679903	Superficial
SLC25A24	1.202476433	0.002836074	0.012662188	Superficial
MYO10	1.19168863	0.000635975	0.007274975	Superficial
AFF1	1.191217771	0.001526458	0.009387421	Superficial
MLLT3	1.18449493	0.002836074	0.012662188	Superficial
MEIS1	1.17701975	0.000992312	0.008042817	Superficial
PARP12	1.170055421	0.000635975	0.012925379	Basal
RAPGEF5	1.161160462	0.000401828	0.007176147	Superficial
BARX2	1.154142342	0.000317696	0.007176147	Superficial
TP63	1.146853343	0.006166656	0.020290909	Superficial
B4GALNT3	1.142848614	0.012681065	0.033478436	Superficial
RPS6KA3	1.139760282	0.001232925	0.008679903	Superficial
ROBO2	1.132994815	0.007423146	0.022959455	Superficial
FOXQ1	1.123032095	0.000992312	0.011395032	Suprabasal
ACVR1	1.11208563	0.010644787	0.029507311	Superficial
SLC5A3	1.108546111	0.017811616	0.043066688	Superficial
EPHB6	1.098421093	0.002315114	0.011381143	Superficial
CRYZ	1.092201992	0.001883197	0.010234018	Superficial
TGFBR1	1.082552543	0.017811616	0.043066688	Superficial
VCL	1.068576576	0.000401828	0.010398392	Suprabasal
IFIH1	1.055273435	0.002315114	0.016076911	Suprabasal
RAB11FIP2	1.051539922	0.004211401	0.016013117	Superficial
CLDN4	1.046614095	0.003462059	0.024854712	Basal
GBP2	1.043537437	0.006166656	0.020290909	Superficial

PDZRN3	1.038845423	0.005105016	0.018023407	Superficial
GJB5	1.036529192	0.012681065	0.033478436	Superficial
USP12	1.026388333	0.001232925	0.01240258	Suprabasal
LMTK2	0.992632835	0.002315114	0.016076911	Suprabasal
ITGB8	0.982346099	0.012681065	0.04332227	Suprabasal
RNF19A	0.979825429	0.000250281	0.010398392	Suprabasal
EFNB2	0.971028932	0.002315114	0.011381143	Superficial
CCDC88C	0.960891309	0.008904627	0.025990329	Superficial
MBNL3	0.951778334	0.005105016	0.018023407	Superficial
GPHN	0.950352459	0.005105016	0.018023407	Superficial
PKP1	0.940526247	0.005105016	0.018023407	Superficial
ATXN1	0.939973269	0.008904627	0.025990329	Superficial
TNK2	0.921895637	0.005105016	0.024729592	Suprabasal
SMPD3	0.91883153	0.012681065	0.033478436	Superficial
ANK3	0.913274645	0.012681065	0.033478436	Superficial
SLC4A7	0.899999926	0.017811616	0.043066688	Superficial
ST14	0.89536654	7.20E-05	0.012455402	Basal
EPN2	0.893135704	0.000795824	0.007615337	Superficial
KRT19	0.892179709	0.012681065	0.04332227	Suprabasal
BMPR2	0.892138295	0.001883197	0.010234018	Superficial
TNRC18	0.889205782	0.017811616	0.043066688	Superficial
FAM110C	0.886645228	0.000506425	0.010398392	Suprabasal
LMO7	0.886093098	0.002315114	0.016076911	Suprabasal
NOL10	0.882088665	0.015054848	0.038009884	Superficial
RNF19B	0.881491342	0.000506425	0.012898798	Basal
UGCG	0.880956022	0.000317696	0.012455402	Basal
BTG1	0.874641303	7.20E-05	0.007176147	Superficial
ALCAM	0.848190649	0.007423146	0.022959455	Superficial
DTNBP1	0.846794959	0.021001058	0.04834985	Superficial
MAP3K8	0.845745236	0.004211401	0.027185186	Basal
MACC1	0.834417685	0.001883197	0.018415354	Basal
DNMBP	0.834004899	0.017811616	0.043066688	Superficial
NDFIP2	0.829957287	0.000635975	0.010421015	Suprabasal
DSC3	0.82685136	0.021001058	0.04834985	Superficial
TWF2	0.823549219	0.000992312	0.008042817	Superficial
PPL	0.822801611	0.000317696	0.012455402	Basal
ACAD9	0.822605788	0.000401828	0.007176147	Superficial
PIM1	0.816682613	0.000506425	0.012898798	Basal
TNIP1	0.799214391	0.000506425	0.010398392	Suprabasal
BACE2	0.792110852	0.000506425	0.010398392	Suprabasal
PLS1	0.792063723	0.002836074	0.018003296	Suprabasal
KCTD1	0.790819901	0.000795824	0.007615337	Superficial
KLF3	0.788073544	0.000401828	0.010398392	Suprabasal
DSP	0.78419116	0.000506425	0.012898798	Basal
RASSF6	0.780974466	0.021001058	0.04834985	Superficial
IL15	0.766980121	0.007423146	0.030890563	Suprabasal
CRADD	0.748667913	0.007423146	0.022959455	Superficial

FAM53B	0.742226226	0.002836074	0.018003296	Suprabasal
ACOX1	0.738898578	0.007423146	0.030890563	Suprabasal
ELF3	0.738836064	0.003462059	0.019935309	Suprabasal
ARFGAP3	0.737414745	0.004211401	0.016013117	Superficial
ZFAND5	0.732780617	0.000635975	0.010421015	Suprabasal
EZR	0.72742657	0.002315114	0.020052792	Basal
KHDRBS3	0.710487783	0.000635975	0.012925379	Basal
MIPEP	0.707166754	0.021001058	0.04834985	Superficial
SHROOM3	0.695335913	0.007423146	0.036548425	Basal
FCHSD2	0.691175748	0.007423146	0.030890563	Suprabasal
TXNRD1	0.681192793	0.004211401	0.022365923	Suprabasal
SGMS1	0.679915708	0.000250281	0.010398392	Suprabasal
ANXA11	0.668315198	0.000401828	0.010398392	Suprabasal
MAL2	0.666971971	0.000992312	0.014320968	Basal
B3GNT2	0.659308899	0.015054848	0.038009884	Superficial
ILVBL	0.648089774	0.021001058	0.04834985	Superficial
PACS1	0.624993882	0.000635975	0.012925379	Basal
RIN2	0.62340617	0.002836074	0.018003296	Suprabasal
TTC39C	0.619828384	0.017811616	0.043066688	Superficial
NDUFA4	0.617621009	0.007423146	0.022959455	Superficial
LMNA	0.616183225	0.002315114	0.011381143	Superficial
JUN	0.612848095	0.010644787	0.029507311	Superficial
NCOR2	0.603984188	0.005105016	0.024729592	Suprabasal
RAP2B	0.602604508	0.012681065	0.04332227	Suprabasal
MGAT4A	0.589001621	0.000250281	0.010398392	Suprabasal
HS6ST1	0.587523478	0.001883197	0.014590393	Suprabasal
IPMK	0.585907313	0.008904627	0.034887819	Suprabasal
PAIP2B	0.580739387	0.021001058	0.04834985	Superficial
SPPL3	0.573634654	0.003462059	0.014108599	Superficial
TULP3	0.561441423	0.000153669	0.010398392	Suprabasal
RAB27B	0.555355604	0.002315114	0.011381143	Superficial
RERE	0.553739866	0.002315114	0.016076911	Suprabasal
RRP12	0.553439503	0.001232925	0.015721789	Basal
JUP	0.54864085	0.000795824	0.0136425	Basal
TRIP10	0.527179997	0.000795824	0.0136425	Basal
ANXA1	0.526354665	0.010644787	0.044687249	Basal
MPZL1	0.508728407	0.008904627	0.025990329	Superficial
EHF	0.492196041	0.010644787	0.038910009	Suprabasal
TRIM33	0.489450207	0.017811616	0.043066688	Superficial
AHNAK	0.472580773	0.000992312	0.014320968	Basal
THUMPD2	0.45263715	0.021001058	0.04834985	Superficial
STK38	0.448032161	0.001526458	0.013165538	Suprabasal
FNBP1	0.441580425	0.003462059	0.019935309	Suprabasal
EIF4G3	0.421871801	0.012681065	0.033478436	Superficial
PLD1	0.416621216	0.010644787	0.044687249	Basal
BAIAP2L1	0.414981604	0.000992312	0.014320968	Basal
SMG7	0.408190956	0.015054848	0.048244165	Suprabasal

DNAJC21	0.40361818	0.000992312	0.008042817	Superficial
N4BP1	0.398063677	0.000992312	0.011395032	Suprabasal
TULP4	0.387329406	0.001526458	0.013165538	Suprabasal
SETD5	0.377610281	0.005105016	0.024729592	Suprabasal
CAPN2	0.36891271	0.015054848	0.048244165	Suprabasal
KLF5	0.366989473	0.004211401	0.016013117	Superficial
PARD3	0.323072656	0.002836074	0.018003296	Suprabasal
PHACTR4	0.32155259	0.015054848	0.048244165	Suprabasal
PRKCH	0.310775043	0.010644787	0.044687249	Basal
C6orf136	0.282069153	0.003462059	0.024854712	Basal
ASAH1	-0.265541854	0.008904627	0.040392504	Basal
IL20RB	-0.323659215	0.012681065	0.04332227	Suprabasal
CTSZ	-0.330853459	0.010644787	0.044687249	Basal
SLC1A4	-0.543851368	0.004211401	0.027185186	Basal
MPPED2	-0.688191794	0.012681065	0.049425568	Basal
PTGR1	-0.715961366	0.001883197	0.018415354	Basal
TGFBI	-0.82611949	0.001526458	0.016792744	Basal
SPRY1	-0.831851276	0.008904627	0.034887819	Suprabasal
FHL2	-0.954787117	0.000317696	0.012455402	Basal
EPCAM	-1.00287481	0.021001058	0.04834985	Superficial
LFNG	-1.036801606	0.000992312	0.014320968	Basal
ANKH	-1.06813916	0.000119764	0.012455402	Basal
ARMCX1	-1.100766717	0.000317696	0.012455402	Basal
SORBS2	-1.125942002	0.000635975	0.012925379	Basal
CYYR1	-1.222114319	0.001883197	0.018415354	Basal
PFN2	-1.249675777	0.000795824	0.0136425	Basal
EFEMP1	-1.268342154	0.000196466	0.012455402	Basal
TGM3	-1.398053373	0.001883197	0.014590393	Suprabasal
SYT17	-1.411928301	0.008904627	0.025990329	Superficial
GHR	-1.424445437	0.000250281	0.012455402	Basal
STXBP1	-1.605053536	0.006166656	0.020290909	Superficial
ALOX12	-1.646262518	0.012681065	0.04332227	Suprabasal
NGEF	-1.697118232	0.000992312	0.011395032	Suprabasal
ZNF365	-1.723119785	0.008904627	0.025990329	Superficial
PLB1	-1.805722624	0.001883197	0.010234018	Superficial
DHRS9	-2.379799313	0.000250281	0.007176147	Superficial
TGM7	-2.419284932	0.01017129	0.029507311	Superficial
IL1A	-2.497686606	0.003462059	0.014108599	Superficial
RAI14	-2.779671032	0.000317696	0.007176147	Superficial
SULT1B1	-3.339062081	0.000182524	0.007176147	Superficial

Supplemental Table 9. List of antibodies used in the study.

Antibody	Company	Clone	Catalog #	Assay	Concentration	Secondary / Conjugate
anti-KI-67	Abcam	SP6	16667	IHC	1:100	goat anti-rabbit
anti-SOX2	Cell Signaling	D1C7J	14962S	IHC	1:500	goat anti-rabbit
anti-KLF5	Sigma-Aldrich	Polyclonal	HPA040398	IHC	1:150	goat anti-rabbit
anti-KRT14	Cell Signaling	LL002	48020	Multispectral IF	1:200	Opal 540
anti-P63	ABclonal	ARC0131	A19652	Multispectral IF	1:100	Opal 690
anti-IVL	Santa Cruz Biotechnology	SY5	sc-21748	Multispectral IF	1:500	Opal 520
anti-CNFN	Novus Biologicals	Polyclonal	NBP2-14668	Multispectral IF	1:200	Opal 570
